# Supplementary material for: Cost-effectiveness of pessary therapy versus surgery for symptomatic pelvic organ prolapse: an economic evaluation alongside a randomised non-inferiority controlled trial
Source: BMJ Open. 2024 May 1;14(5):e075016. doi: 10.1136/bmjopen-2023-075016 (PMC11086579; doi:10.1136/bmjopen-2023-075016)
Supplement: Supplementary data [file bmjopen-2023-075016supp001.pdf]

Study protocol

PEOPLE study

Pessary or Surgery for a Symptomatic Pelvic Organ Prolapse

Study protocol

Ben ÂJ, *et al. BMJ Open* 2024; 14:e075016. doi: 10.1136/bmjopen-2023-075016

Study protocol

Table of contents

1. Study protocols 3

1.1 Original study protocol..4

1.2 Final study protocol 55

1.3 Summary of amendments to study protocol.. 107

1.3.1 Detailed summary of all amendments 107

1.3.2 Table with amendments and corresponding section 109

Ben ÂJ, *et al. BMJ Open* 2024; 14:e075016. doi: 10.1136/bmjopen-2023-075016

1. Study protocols

Original study protocol:	Version 1.5, November 2014
Final study protocol:	Version 1.22, February 2018

Study protocol

1.1 Original study protocol

Pessary or surgery for symptomatic pelvic organ prolapse

Version 1.~~4~~ 5 ~~October~~ November 2014

Ben ÂJ, *et al. BMJ Open* 2024; 14:e075016. doi: 10.1136/bmjopen-2023-075016

Study protocol

PROTOCOL TITLE **'Pessary or surgery for symptomatic pelvic organ prolapse'**

**Protocol ID**	**2014 / 1.4**
**Short title**	**Pessary or surgery for symptomatic pelvic organ prolapse**
**EudraCT number**	***Not applicable***
**Version**	**1.~~4~~5**
**Date**	**O~~ctober~~ November 2014**
**Coordinating investigator/project leader**	**Prof. Dr. C.H. van der Vaart, gynaecologist**<br>**University Medical Centre Utrecht**
**Principal investigator(s) (in Dutch: hoofdonderzoeker/ uitvoerder)**	**~~Dr. A. Vollebregt, gynaecologist~~**<br>**~~Spaarne Hospital~~**<br><br>**M.K. van de Waarsenburg, MD**<br>**University Medical Centre Utrecht**
**Multicenter: per site**	**Prof. Dr. C.H. van der Vaart, UMC Utrecht**<br>**Dr. A.L. Milani, Reinier de Graaf Gasthuis**<br>**Dr. A. Vollebregt, Spaarne Hospital Hoofddorp**<br>**Dr. J.P.W.R. Roovers, Academic Medical Center**<br>**Dr. K.B. Kluivers, Radboud UMC Nijmegen**<br>**Dr. V. Dietz, Catharina Hospital Eindhoven**<br>**Dr. H.W.F. van Eijndhoven, Isala Clinic Zwolle**<br>**Dr. H.S. The, Sint Antonius Hospital Nieuwegein**<br>**Dr. R.P. Schellart, Kennemer Gasthuis Haarlem**<br>**Drs. A.M.W. Broekman, Sint Franciscus Gasthuis Rotterdam**<br>**Drs. J. van Bavel, Amphia Hospital Breda**
**Sponsor (in Dutch: verrichter/opdrachtgever)**	**Prof. Dr. C.H. van der Vaart, gynaecologist**
**Subsidising party**	**ZonMw Project nr 837002525**
**Independent expert (s)**	**Dr. R.P. Zweemer**<br><br>**University Medical Centre Utrecht**

**Laboratory sites <*if applicable*>**	***Not applicable***
**Pharmacy <*if applicable*>**	***Not applicable***

PROTOCOL SIGNATURE SHEET

Name	Signature	Date
**Head of Department:**<br><br>**Prof. Dr. B.C.J.M. Fauser**<br>**Department of Reproductive Medicine and Gynaecology**<br>**University Medical Centre Utrecht**		
**[Coordinating Investigator/Project leader/Principal Investigator]:**<br><br>**Prof. Dr. C.H. van der Vaart, gynaecologist**<br>**University Medical Centre Utrecht**		

peOp/e Study protocol nvog consortium 2.0

TABLE OF CONTENTS

1. INTRODUCTION AND RATIONALE 9
2. OBJECTIVES 13
3. STUDY DESIGN 14
4. STUDY POPULATION 15
   4.1 Population (base) 15
   4.2 Inclusion criteria 15
   4.3 Exclusion criteria 15
   4.4 Sample size calculation 15
5. TREATMENT OF SUBJECTS 17
   5.1 Investigational product/treatment 17
   5.2 Use of co-intervention (if applicable) 17
   5.3 Escape medication (if applicable) 18
6. INVESTIGATIONAL PRODUCT 19
   6.1 Name and description of investigational product(s) 19
   6.2 Summary of findings from non-clinical studies 19
   6.3 Summary of findings from clinical studies 19
   6.4 Summary of known and potential risks and benefits 20
   6.5 Description and justification of route of administration and dosage 20
   6.6 Dosages, dosage modifications and method of administration 20
   6.7 Preparation and labelling of Investigational Medicinal Product 20
   6.8 Drug accountability 20
7. NON-INVESTIGATIONAL PRODUCT 21
   7.1 Name and description of non-investigational product(s) 21
   7.2 Summary of findings from non-clinical studies 21
   7.3 Summary of findings from clinical studies 21
   7.4 Summary of known and potential risks and benefits 21
   7.5 Description and justification of route of administration and dosage 21
   7.6 Dosages, dosage modifications and method of administration 21
   7.7 Preparation and labelling of Non Investigational Medicinal Product 21
   7.8 Drug accountability 21
8. METHODS 22
   8.1 Study parameters/endpoints 22
      8.1.1 Main study parameter/endpoint 22
      8.1.2 Secondary study parameters/endpoints (if applicable) 22
      8.1.3 Other study parameters (if applicable) 25
   8.2 Randomisation, blinding and treatment allocation 25
   8.3 Study procedures 26
   8.4 Withdrawal of individual subjects 27
      8.4.1 Specific criteria for withdrawal (if applicable) 27

8.5 Replacement of individual subjects after withdrawal 27
8.6 Follow-up of subjects withdrawn from treatment 27
8.7 Premature termination of the study 27
9. SAFETY REPORTING 28
9.1 Section 10 WMO event 28
9.2 AEs, SAEs and SUSARs 28
9.2.1 Adverse events (AEs) 28
9.2.2 Serious adverse events (SAEs) 28
9.2.3 Suspected unexpected serious adverse reactions (SUSARs) 29
9.3 Annual safety report 29
9.4 Follow-up of adverse events 29
9.5 [Data Safety Monitoring Board (DSMB) / Safety Committee] 29
10. STATISTICAL ANALYSIS 30
10.1 Primary study parameter(s) 30
10.2 Secondary study parameter(s) 30
10.3 Other study parameters 31
10.4 Interim analysis (if applicable) 32
11. ETHICAL CONSIDERATIONS 33
11.1 Regulation statement 33
11.2 Recruitment and consent 33
11.3 Objection by minors or incapacitated subjects (if applicable) 33
11.4 Benefits and risks assessment, group relatedness 33
11.5 Compensation for injury 33
11.6 Incentives (if applicable) 33
12. ADMINISTRATIVE ASPECTS, MONITORING AND PUBLICATION 35
12.1 Handling and storage of data and documents 35
12.2 Monitoring and Quality Assurance 35
12.3 Amendments 35
12.4 Annual progress report 35
12.5 End of study report 35
12.6 Public disclosure and publication policy 36
13. STRUCTURED RISK ANALYSIS 37
13.1 Potential issues of concern 37
13.2 Synthesis 37
14. REFERENCES 38
Appendix 1… 42
Appendix 2… 43
Appendix 3… 45
Appendix 4… 46
Appendix 5… 47

Study protocol

LIST OF ABBREVIATIONS AND RELEVANT DEFINITIONS

**ABR**	**ABR form, General Assessment and Registration form, is the application form that is required for submission to the accredited Ethics Committee (In Dutch, ABR = Algemene Beoordeling en Registratie)**
**AE**	**Adverse Event**
**AR**	**Adverse Reaction**
**CA**	**Competent Authority**
**CCMO**	**Central Committee on Research Involving Human Subjects; in Dutch: Centrale Commissie Mensgebonden Onderzoek**
**CV**	**Curriculum Vitae**
**DSMB**	**Data Safety Monitoring Board**
**EU**	**European Union**
**EudraCT**	**European drug regulatory affairs Clinical Trials**
**GCP**	**Good Clinical Practice**
**IB**	**Investigator's Brochure**
**IC**	**Informed Consent**
**IMP**	**Investigational Medicinal Product**
**IMPD**	**Investigational Medicinal Product Dossier**
**METC**	**Medical research ethics committee (MREC); in Dutch: medisch ethische toetsing commissie (METC)**
**(S)AE**	**(Serious) Adverse Event**
**SPC**	**Summary of Product Characteristics (in Dutch: officiële productinfomatie IB1-tekst)**
**Sponsor**	**The sponsor is the party that commissions the organisation or performance of the research, for example a pharmaceutical company, academic hospital, scientific organisation or investigator. A party that provides funding for a study but does not commission it is not regarded as the sponsor, but referred to as a subsidising party.**
**SUSAR**	**Suspected Unexpected Serious Adverse Reaction**
**Wbp**	**Personal Data Protection Act (in Dutch: Wet Bescherming Persoonsgevens)**
**WMO**	**Medical Research Involving Human Subjects Act (in Dutch: Wet Medisch-wetenschappelijk Onderzoek met Mensen**

Study protocol

SUMMARY

Rationale: Moderate to severe pelvic organ prolapse symptoms can be treated with pessary or surgery. Both treatments appear to be effective, but have not been compared directly.

Hypothesis: The strategy of pessary as initial therapy is as effective as direct surgery for moderate to severe POP, but it is associated with lower costs.

Objective: The primary objective is to compare the effectiveness and cost-effectiveness of pessary versus surgery as initial treatment for moderate to severe symptomatic pelvic organ prolapse (POP) in women at two year after initiation of treatment. The secondary objective is the development of a prediction model for failure of pessary use and surgery within 2 years.

Study design: Cohort study with embedded randomized controlled trial.

Study population: Treatment naïve women with POP who present with moderate to severe symptoms.

Intervention (if applicable): Pessary therapy or vaginal POP surgery.

Main study parameters/endpoints:

Primary outcome: Global impression of improvement of POP symptoms at 24 months measured with PGI-I

Secondary outcomes:

- Changes in symptom bother and disease-specific quality of life at 12 and 24 months follow-up
- Changes of sexual function at 12 and 24 months follow-up
- Changes in general quality of life at 3, 6, 12 and 24 months of follow up
- Adverse events/complications related to both treatment strategies during the study period
- Development of prediction model to identify factors for failing of pessary and surgery.
- Costs-effectiveness analyses

Nature and extent of the burden and risks associated with participation, benefit and group relatedness: Both treatment arms are routine treatments in the Netherlands. Patients in the RCT can have the risks of surgery instead of the risks from pessary therapy.

Study protocol

1. INTRODUCTION AND RATIONALE

Problem definition

Female pelvic organ prolapse (POP) is a common problem in women that negatively affects quality of life. The estimated prevalence of symptomatic POP among women between 45-85 years of age is 8.3 - 11% [1,2]. It is current practice in the Netherlands that the general practitioner (GP) treats the majority of women with POP symptoms. Women with moderate to severe POP symptoms are often referred to a gynecologist for treatment. This study focuses at the subgroup of moderate to severe POP.

Known effective treatment options for moderate to severe POP are pessary or surgery. A pessary has proven its effectiveness in the treatment of symptomatic POP, mainly in cystocele and uterine descent. However, studies are mainly observational in nature and inherently subject to selection and indication bias [3]. In literature, outcomes of pessary therapy are mainly recorded in terms of (dis-) continuation of therapy and to a much lesser extent in terms of symptom relief. The pessary continuation rate is 60% [3]. This is confirmed by a Dutch pilot study in 65 women that showed a satisfaction with pessary in 57% of women and an operation rate of 43% at 12 months follow up [4]. In this study, 80% of women who continued pessary therapy reported much to very much improvement of their POP symptoms at 1 year follow up [4]. Reasons of discontinuation are pressure ulcer, vaginal discharge, discomfort or loss of fitting. These complications are reported to occur in up to 53% of women [5]. Half of them will decide to stop using pessary, but it is unclear which characteristics predict this outcome. Check-up of pessary therapy can be performed by either a general practitioner (GP), gynecologist or by self-management. According to a recent survey 50% percent of gynecologists involved in urogynaecology always offer self-management 40% on indication, and 10% never. Pessary therapy is inexpensive and costs are mainly related to doctor visits and treatment of side effects. In case of self-management costs might even be lower,

Surgery for POP results in much to very much improvement of symptoms in 80% of women and improvement of quality of life [6-9]. An anterior colporraphia is considered the standard procedure for a cystocele, as is the posterior colporraphia for a rectocele. For uterine descent uterus sparing techniques, like sacrospinous hysteropexy (SH) and modified Manchester-Fothergill procedure, or vaginal hysterectomy can be performed [10-12]]. Complications of POP surgery are temporary urinary retention, temporary buttock pain in case of sacrospinous hysteropexy, urinary tract infection, hematoma or dyspareunia [11]. These complications seldom lead to persistent morbidity. The most

common “complication” is the recurrence of symptomatic POP or de novo stress-incontinence that may lead to additional surgery, pessary therapy, or pelvic floor physiotherapy. As part of a RCT, comparing mesh with fascia plication, we found that
11% of women needed additional surgery after anterior colporraphia at 24 months follow up [7,9]. As in pessary therapy, the characteristics that predict successful or unsuccessful surgical therapy are largely unknown.

The decision which treatment option to choose depends on both patient and doctor’s preferences. In our pilot survey 70% of gynecologists informed their patients about the possibility of pessary therapy, but it is unknown how many women actually received a pessary. A recent Dutch study showed that 48% of treatment-naive women preferred surgery, 36% a pessary and 16% had no preference [28]. It is therefore reasonable to assume that at least 50% of treatment naïve women with moderate to severe prolapse symptoms will have surgery as primary treatment.

Although clinical efficacy appears to favor surgery [3], the large variation in study design, outcome measurements and loss to follow up makes any comment on the best treatment option speculative. This is recognized in two recent reviews on the subject that both urge the need for randomized trials comparing surgery and pessary for POP [13,14]. Efficacy can be expressed in terms of clinical outcome but also in terms of cost-effectiveness. It is obvious that surgery (especially hospital costs) is much more expensive than pessary therapy, but the cost-effectiveness of the surgical or pessary strategy has never been assessed. Based on current cohort and case-control studies we hypothesize that a strategy of initial pessary therapy for moderate to severe POP, is more cost-effective than surgery.
We propose to perform a randomized controlled trial to generate evidence for the optimal and most cost-effective primary treatment for moderate to severe POP, including a better a priori patient selection for treatment by identifying factors of failure for pessary therapy or surgery.

Relevance

At present a national multidisciplinary guideline on the diagnosis and treatment of POP is completed. The guideline identifies the lack of evidence with respect to the best treatment option for moderate to severe prolapse, a conclusion that is confirmed by the 2013 Cochrane Collaboration review [13]. In this evidence “vacuum” both doctors and patient
preferences rule, but unfortunately these are not supported by facts. If we look at the available data the following calculation emerges.

About 60% of women who start pessary therapy in the specialist care setting will continue using it at one year [4,15]. Eighty percent of them will report much to very much improvement, resulting in an overall 48% much to very much improvement. The majority of the 40% of women who are not satisfied with pessary therapy will request or are offered additional surgery. After surgery 80% of women report much to very much improvement of POP symptoms [6]. Combining these percentages, women who originally started with pessary therapy may also expect 80% (48% after initial pessary treatment + 32% after additional surgery =) much to very much improvement. Based on these estimates it is expected that the outcome of both treatment strategies will eventually result in a global improvement of symptoms in 80% of women. With equal clinical outcomes of both strategies the costs needed to obtain these outcomes become crucial. With the exception of a cost calculation based on a Markov model, no direct cost-effectiveness studies on the use of pessary or surgery for POP have been performed [16]. The relevance of this project, with the high prevalence of POP worldwide, associated costs and insufficient evidence, is high. We have searched the www.clinicaltrials.gov database (3th March 2014) on similar studies (comparing pessary with surgery) but none were found.

However, if we were to prove that pessary therapy is more cost-effective then surgical treatment, this does not imply that a trial of pessary should always be undertaken. There is also insufficient evidence on which patient characteristics are associated with failure of pessary treatment or surgery (systematic review). The knowledge on how to predict which women will have a very low chance of success with pessary therapy can further improve effective treatment strategy management. This will contribute to treatment efficacy. This is not only very relevant for the hospital specialist care setting, but this knowledge can also be implemented in general practitioner practice units.

There is very limited evidence on the optimal management strategy for pessary cleaning, both in time interval as well as in who should perform the cleaning. Our study is unique and therefore relevant since self-management is advocated in the study setting. This will not only allow it to obtain data in a standardized way, but also involves the woman in her own management. This involvement is strongly advocated by two major gynecologic patient organizations (‘Patienten Gynaecologie Nederland’ and the ‘Stichting Bekkenbodem Patienten’). These two organizations, as well as the Dutch urogynaecological consortium have identified this study to be highly relevant.

In line with the report "Medisch Specialistische zorg 20/20" we are heading towards integral health care in which the general physician and medical specialist will work more closely together, using the same treatment protocol for various illnesses. The information and conclusions of this trial will add level I scientific evidence to such an integral protocol and guideline for women with symptomatic POP. This will aid in a better patient selection that will need referral to the specialist. The data on patient's self-management of pessary treatment will supply information for patient instructions, which are relevant for information leaflets on the subject.

2. OBJECTIVES

The aim of this multicenter pragmatic cohort study with embedded randomized controlled non-inferiority trial comparing pessary therapy versus surgery is twofold:

1. To prospectively compare the effectiveness and cost-effectiveness of pessary therapy or surgery as primary treatment of moderate to severe symptomatic cystocele, uterine descent and/or rectocele in women at two year after initiation of treatment.
2. To develop a prediction model for failure of pessary use and surgery within the first 2 years.

3. STUDY DESIGN

Multicenter pragmatic cohort study with an embedded randomized controlled non-inferiority trial comparing pessary [CE 0086] therapy versus surgery including an economic evaluation. The follow up will be 24 months.

After a short (30 minutes) trial of pessary fitting before randomization into our protocol. This ensures that only women who fit both treatment options enter the randomization procedure. The trial is short and only aims at fitting, not symptom relief. For those women with an unsuccessful pessary fitting baseline characteristics will be recorded to allow analyses of this group.

See also appendix 1.

4. STUDY POPULATION

4.1 Population (base)

All women with a symptomatic POP will be included.

4.2 Inclusion criteria

In order to be eligible to participate in this study, a subject must meet all of the following criteria:

1. Women with a prolapse stage 2 or more.
2. Women with moderate to severe POP symptoms. Moderate to severe POP symptoms is defined as a prolapse domain score > 33 on the validated Dutch version of the Pelvic Floor Distress Inventory (PFDI-20) [8, 23, 24].
3. Women who have had a successful pessary fitting procedure: for the RCT.
4. Written informed consent.

4.3 Exclusion criteria

A potential subject who meets any of the following criteria will be excluded from participation in this study:

1. Prior urogynaecological (prolapse or incontinence) surgery
2. Probability of future childbearing
3. Insufficient knowledge of the Dutch language
4. Co-morbidity causing increased surgical risks at the discretion of the surgeon
5. Major psychiatric illness
6. Prior pessary use

4.4 Sample size calculation

With 198 women per group, we will have 80% power to reject the null hypothesis that pessary therapy is inferior to surgery, with a 1-sided alpha of 0.05, a non-inferiority margin of 10% and the proportion in the standard group is 80% (NQueryAdvisor). Accounting for 10% loss to follow-up we plan to randomize 436 patients.

The sample size calculation for prediction models is based on the number of failures of pessary or surgical therapy. For each potential predictor in the model we need 10-15 failures. Our pessary group sample size is 198 women. An estimated 40% (80 women) will cross over to surgery and can be regarded as failures. Our sample size is therefore sufficient

to develop the prediction model for failure of pessary therapy for 6 to 8 items. In the surgery group 20% of women will not be satisfied with the result of treatment. With the same sample size of 198 women, the 40 women who are dissatisfied allow us to study up to 4 potential predictive factors.

5. TREATMENT OF SUBJECTS

5.1 Investigational product/treatment

Pessary [CE 0086] therapy and surgery both are options for the treatment of a symptomatic POP. Ten large urogynecological units (university hospitals or teaching hospitals) that have worked together in previous consortium studies will participate in this multicenter trial. All participating gynaecologists have fitted at least 100 pessaries and performed more than 100 surgical POP procedures prior to the start of this study.

All pessaries are made of modern silicon material. All types of pessaries, both supportive and occlusive/space filling are allowed according to the judgment of the gynaecologist. A recent randomized trial comparing supportive (ring) and occlusive (Gelhorn) showed no differences [17]. After placing the pessary, all women will receive verbal and written instructions on the self-management of pessary therapy.

The first pessary follow up visit will always be performed by the gynaecologist. In case of self-management the frequency of cleaning is left to her personal judgment, but may not exceed 1 month. If self-management is not possible, women will be seen at 4 months intervals for pessary cleaning and vaginal inspection, preferable by their GP. In case of vaginal atrophy topical estrogens will be advised according to pharmaceutical guidelines. The diagnosis of atrophy is left to the judgment of the treating physician, since no clear definition for atrophy is available yet [18].

All surgical procedures will be performed according to our national guidelines. In this pragmatic trial the decision which technique to use is left, to the discretion of the gynaecologist, within the limitations below [19]. Cystocele repair will consist of conventional anterior colporrhaphy [9]. For uterine descent different techniques are allowed [20]. These techniques can either be uterus sparing (sacrospinous hysteropexy [10], modified Manchester-Fothergill procedure [12] or a abdominal sacrocolpopexy [9]) or a vaginal hysterectomy. Recent studies showed similar effectiveness on both anatomical and functional outcomes for these different techniques [10, 12, 21]. A coexistent stage 2 rectocele repair will be a conventional colporrhaphia posterior. All procedures are performed under general or spinal anesthesia and under antibiotics and thrombosis prophylaxis according to local protocols.

5.2 Use of co-intervention (if applicable)

Not applicable.

5.3 Escape medication (if applicable)

Not applicable.

6. INVESTIGATIONAL PRODUCT

6.1 Name and description of investigational product(s)

6.2 Summary of findings from non-clinical studies

Not applicable.

6.3 Summary of findings from clinical studies

Three systematic reviews of the literature were performed by four members of our Dutch urogynaecology consortium (details in appendix 2-4) that concluded:

1. Systematic review on the (cost)effectiveness of pessary use compared to surgery

There are a very limited number of comparative studies on the efficacy of surgery or pessary use for POP. The differences in study population, inclusion criteria, follow-up period, large numbers of loss to follow-up, different outcome measures makes interpretation difficult if not impossible. The two studies that presented data on functional outcome in terms of prolapsed symptom reduction were favorable for surgery (appendix 2).

2. Systematic review of factors influencing pessary fitting and continuation

A systematic review was performed to identify the satisfactory pessary fitting rate and the continuation rate of pessary use. The factors influencing these rates as well as the cross over to prolapse surgery were identified from previous studies (appendix 3).

Summarizing the results show that an estimated 75% of women will have a successful fitting and 59% will continue pessary use at variable follow-up between 3 months and 5 years. In these 18 studies, 8 factors have been tested more than 4 times as prognostic factor of successful pessary use: Stress urinary incontinence was found associated with discontinuation of pessary in 5 out of 7 studies. In 7 out of 10 studies previous prolapse surgery or hysterectomy was associated with less continuation of pessary use. Higher age was related to continuation of pessary use in 3 out of 6 studies, whereas no correlation was found in the other studies. In 1 out of 4 studies sexual activity was related to longer pessary use, whereas in 1 out of 4 related to the choice for surgery. In the two other studies no correlation was found. In one study where the prolapse in a specific vaginal compartment was related to outcome, nor cystocele was related to longer pessary use.

Parity en menopausal status and hormonal replacement were mostly not related to continued pessary use.

3. Review of factors influencing failure of POP surgery.

A systematic review of factors influencing failure of POP surgery was performed concerning recurrence after surgery (surgery failure). There were 1 case control study, 3 prospective studies and 6 retrospective studies. There were 2298 women included in the studies. Forty-four (44) potential risk factors have been studied, of which 12 risk factors have at least once been identified as statistically significant risk factors in a multivariate logistic regression analysis (appendix 4).

6.4 Summary of known and potential risks and benefits

The present study carries low risks for the participant. Pessary [CE 0086] or surgery is standard care for symptomatic pelvic organ prolapse. Known risks for surgery are blood loss, risk of infection, dyspareunia, urine incontinence or a recurrence of a symptomatic pelvic organ prolapse.

The benefit of the study lies in a better understanding of satisfaction and cost effectiveness.

6.5 Description and justification of route of administration and dosage

Not applicable

6.6 Dosages, dosage modifications and method of administration

Not applicable

6.7 Preparation and labelling of Investigational Medicinal Product

Not applicable

6.8 Drug accountability

Not applicable

7. NON-INVESTIGATIONAL PRODUCT

Not applicable.

7.1 Name and description of non-investigational product(s)

Not applicable.

7.2 Summary of findings from non-clinical studies

Not applicable.

7.3 Summary of findings from clinical studies

Not applicable.

7.4 Summary of known and potential risks and benefits

Not applicable.

7.5 Description and justification of route of administration and dosage

Not applicable.

7.6 Dosages, dosage modifications and method of administration

Not applicable.

7.7 Preparation and labelling of Non Investigational Medicinal Product

Not applicable.

7.8 Drug accountability

Not applicable.

8. METHODS

8.1 Study parameters/endpoints

8.1.1 Main study parameter/endpoint

The primary outcome of this study is the percentage of women with much or very much improvement of POP symptoms at 2 years follow-up, as measured with the Patient Global Impression of Improvement (PGI-I)scale [22].
PGI-I is a 7-point Likert scale, with scores ranging from very much worse to very much improved. Success is defined as 'much or very
much' improvement.

8.1.2 Secondary study parameters/endpoints (if applicable)

1. Changes in symptom bother and quality of life at 12 and 24 months follow up.
2. Changes in sexual function at 12 and 24 months follow up.
3. Changes in general quality of life at 3, 6, 12 and 24 months.
4. Adverse events/complications related to both treatment strategies.
5. Development of prediction model to identify fail factors for pessary and surgery
6. Cost-effectiveness

8.1.3 Other study parameters (if applicable)

Baseline characteristics: Age; ethnicity; alcohol; smoking; number and mode of deliveries; menopausal status; hormone use; drug use; height; weight; co-morbidity (hypertension, diabetes mellitus, COPD, neurological disease, depression, cardiovascular disease); history of gynaecological operations; family history of prolapse; allergies, incontinence and sexual activity.
Physical examination: time, POP-Q, atrophy, stress test, blood loss, excessive discharge.

8.2 Randomisation, blinding and treatment allocation

After written informed consent is obtained, and inclusion and exclusion criteria are assessed, women will be randomly allocated in a 1:1 ratio to either treatment with a pessary or surgical treatment. Randomization will be done web based using ALEA, the software for randomization in clinical trials currently used by most studies in the Dutch consortium for studies on women's health and reproduction studies. The

randomization sequence will be computer generated using variable blocks of two and four, stratified for centre.

After entering the woman's initials and confirming inclusion criteria on the website, a unique number for randomization will be generated and the allocation code will be disclosed. This unique number cannot be deleted afterwards. This study will be open label because the nature of the intervention meant that masking to the intervention was not possible.

Women who attend the cohort will also be registered in ALEA.

8.3 Study procedures (see also appendix 5)

This study will be performed within the Dutch Urogynaecology Research Consortium, a subdivision of the Dutch Consortium for studies on women's health. Infrastructure (research nurses for counseling and data-monitoring, the use of web-based data entry), expertise on methodology and cost-effectiveness is shared.

1. Symptom bother and disease-specific quality of life are measured with the Pelvic Floor Distress Inventory (PFDI-20) and Pelvic Floor Impact Questionnaire (PFIQ) [23,24]. The validated version of the Dutch PFDI consists of five domains: pelvic organ prolapse, urinary incontinence, overactive bladder, pain, and obstructive micturition. The PFIQ measures the impact of urogenital symptoms on quality of life and consist of five domains: physical functioning, mobility, emotional health, embarrassment and social functioning.

2. Sexual function is measured with the PISQ-R. It is an international disease-specific questionnaire that measures sexual functioning in sexually active and inactive participants [26]. At this time, the Dutch translation is in progress, which will be finished in 2014.

3. Generic quality of life is measured with the EQ-5D and a questionnaire "doktersbezoek".

4. The adverse events of surgery recorded will consist of; direct peri-and postoperative complications (bleeding, pain and infection); interventions for complications; recurrent prolapse; de novo stress urinary incontinence. The adverse events of pessary recorded will consist of; discharge; pain; discomfort; bleeding; involuntary loss of pessary; de novo stress urinary incontinence.

5. The development of a prediction model is separately described in paragraph "data analyses".

6. The economic evaluation is described below.

ANTICIPATED COST-EFFECTIVENESS/BUDGET IMPACT

Cost differences between the two strategies are mainly the result from differences in costs associated with the initial intervention. Cost of a POP procedure is estimated at 4000 euros direct medical costs, and 4000 euros associated with lost productivity (indirect costs) if a societal perspective is used. The direct cost of pessary use is estimated at 200 euros, including costs for the pessary itself (50 euros) and consultations in the first year (150 euros). The estimated cost differences between the two strategies depend on the extent that women are (and remain) satisfied with the initial procedure (surgical or pessary): in case of dissatisfaction with the procedure, additional costs are generated by a subsequent intervention ((re-)operation, pessary, or pelvic physiotherapy). The flowchart (see appendix 1) illustrates the expected outcomes for each strategy. Based on the assumptions reflected in this flowchart, combined with approximate estimates for unit costs for POP surgery, pessary, GP and specialist visits, the anticipated impact on the annual health care budget as well as societal costs were estimated.

At present, the primary therapy for women presenting with moderate to severe POP is either surgery or pessary. The exact ratio is unknown, but is probably 50/50. If 50% women would receive primary surgery the current medical costs amount to 34 million Euros. If all women would start with pessary therapy, these costs would be 20 million euros, and the potential budget impact would be 14 million Euros/year. As it is not realistic that all women will start with pessary if this strategy proves to be successful, at 85% implementation of the pessary strategy, the annual budget impact will be around 10 million euros. The economic impact to society (including indirect (productivity) costs) will be 28 million euros and 20 million euros, at 100% and 85% implementation, respectively.

Sensitivity analyses showed, that these “base case” results are affected by estimated unit costs for POP surgery (direct and indirect costs) and the satisfaction rate for pessary, relative to surgery, but even the most conservative assumptions would lead to major cost savings for the health care budget (5 million euros) and society (15 million euros).

ECONOMIC EVALUATION

Considering the non-inferiority design of the study, we will not be able to rule out a small but acceptable difference in favor of POP surgery. Consequently, the economic

evaluation will be setup as a cost-effectiveness analysis, where cost-effectiveness will be expressed as costs per improvement outcome (much or very much improvement on the Patient Global Impression of Improvement (PGI-I)), and the incremental cost-effectiveness ratio as costs saved per additional case of unsatisfactory outcome. We will also perform a cost-effectiveness analysis using QALYs as health outcome, to express the difference between the two strategies in terms of costs (saved) per QALY (lost).

The economic evaluation will therefore encompass a cost-effectiveness analysis (CEA), a cost-utility analysis (CUA) as well as a budget impact analysis (BIA) from a health care budget and a societal perspective, with a time horizon between randomization and 2 years follow up. The primary outcome in the cost-effectiveness analysis will be costs per satisfactory outcome (primary clinical outcome), and the incremental cost-effectiveness ratio will reflect the costs saved per additional case of unsatisfactory outcome. As we hypothesize that pessary as a primary strategy in these patients does not result in more unsatisfactory outcomes, increased use of pessary will result in a decrease in the number of POP surgeries, and associated costs of hospital stay, recovery and (from a societal perspective) productivity loss (non-inferior strategy at lower costs).

Based on data actually observed in the trial, total costs associated with both surgery and pessary as a primary strategy will be estimated. Total costs can be divided into direct medical costs, non-medical costs and indirect costs. Direct medical costs are generated by utilization of primary or secondary health care services (including POP surgery, hospital stay, diagnostic procedures, medication). Non-medical costs are generated by travel expenses, and informal care; and indirect costs result from lost productivity due to absence from work or lost opportunity for non-paid activities. Non-medical and indirect costs are only included in the analysis from a societal perspective.

Resource utilization will be documented in the clinical report form (CRF) and complementary patient questionnaires, based on the Medical Consumption Questionnaire (MCQ) and Productivity Costs Questionnaire (PCQ) [29,30]. In patients for whom complete follow-up is not available, cost and quality-of-life data will be extrapolated using multiple imputations. Unit costs will be based on Dutch guideline

prices (for primary and secondary health services, informal care and lost productivity), and market prices (for medication)[31,32]

Similarly, the incremental costs per QALY gained will be estimated over a period of two years. Health state utilities to estimate QALYs will be derived from an EQ-5D measurement at discharge, as well as at follow-up assessments. Utility values for EQ-5D scores will be based on UK-estimates (Dolan, 1997). Utility scores will be linearly interpolated, assuming constant increase/decrease between subsequent assessments.

Robustness for sampling uncertainty as well as uncertainty associated with cost estimates and assumptions will be assessed in sensitivity analyses, including: Dutch health states (Lamers, 2005) instead of the UK based model in the main analyses; and varying unit costs for pertinent volumes of health care utilization (e.g. costs of POP surgery, pessary use, productivity costs).

The incremental costs and effects will be depicted in a cost effectiveness plane and cost-effectiveness acceptability curves providing information directly interpretable as the probability of one intervention being cost-effective compared to the alternative given a ceiling ratio that policy makers are willing to invest.

BIA

In a budget impact analysis, study results will be extrapolated to the national level to estimate the total impact on the health care budget per annum for the Netherlands in terms of cost reduction and health outcomes (satisfactory outcomes as well as QALYs). As economic consequences of the intervention are expected to span multiple years, this accumulation of cost (savings) will be reflected in the budget impact analyses.

The Budget Impact Analysis will be executed according to the international ISPOR guidelines [33]. This framework for creating a budget impact model includes formalized guidance about the acquisition and use of data in order to make budget projections. In addition to the societal perspective, the BIA will therefore be also report economic consequences from the perspective of the Dutch budgetary health care framework (BKZ). If the probability of an unsatisfactory outcome exceeds the non-inferiority limit, recommending pessary as primary treatment for all women is not

feasible, and an economic evaluation/budget impact analysis is not sensible. To estimate costs, we will follow the Handleiding Kosten onderzoek CVZ 2010.

8.4 Withdrawal of individual subjects

Subjects can leave the study at any time for any reason if they wish to do so without any consequences. The investigator can decide to withdraw a subject from the study for urgent medical reasons.

8.4.1 Specific criteria for withdrawal (if applicable)

Not applicable.

8.5 Replacement of individual subjects after withdrawal

We will not replace patients who withdrew informed consent. We will replace patients that are randomized by mistake, for example because of technical errors with online randomization.

8.6 Follow-up of subjects withdrawn from treatment

Patients withdrawn from the intervention but not from informed consent will be followed up.

8.7 Premature termination of the study

This study includes standard care, therefore it is very unlikely that unexpected complications will occur. Therefore premature termination is not applicable.

9. SAFETY REPORTING

9.1 Section 10 WMO event

In accordance to section 10, subsection 1, of the WMO, the investigator will inform the subjects and the reviewing accredited METC if anything occurs, on the basis of which it appears that the disadvantages of participation may be significantly greater than was foreseen in the research proposal. The study will be suspended pending further review by the accredited METC, except insofar as suspension would jeopardise the subjects' health. The investigator will take care that all subjects are kept informed.

9.2 AEs, SAEs and SUSARs

9.2.1 Adverse events (AEs)

Adverse events are defined as any undesirable experience occurring to a subject during the study, whether or not considered related to the study. All adverse events reported spontaneously by the subject or observed by the investigator or his staff will be recorded. During visits complaints will be questioned systematically.

9.2.2 Serious adverse events (SAEs)

A serious adverse event is any untoward medical occurrence or effect that at any dose:

- results in death;
- is life threatening (at the time of the event);
- requires hospitalisation or prolongation of existing inpatients' hospitalisation (>4 days);
- results in persistent or significant disability or incapacity;
- is a congenital anomaly or birth defect;
- Any other important medical event that may not result in death, be life threatening, or require hospitalization, may be considered a serious adverse experience when, based upon appropriate medical judgement, the event may jeopardize the subject or may require an intervention to prevent one of the outcomes listed above.

SAEs have to be reported when its occurrence appears in two days after the study operations. The investigators in participating centres should inform the coordinating investigator as soon as possible but at least the next working day.

The sponsor will report the SAEs through the web portal *ToetsingOnline* to the accredited METC that approved the protocol, within 15 days after the sponsor has first knowledge of the serious adverse events.

SAEs that result in death or are life threatening should be reported expedited. The expedited reporting will occur not later than 7 days after the responsible investigator has first knowledge of the adverse event. This is for a preliminary report with another 8 days for completion of the report.

9.2.3 Suspected unexpected serious adverse reactions (SUSARs)

Not applicable.

9.3 Annual safety report

The annual safety report will be combined with the annual progress report (see chapter 12.4).

9.4 Follow-up of adverse events

All AEs will be followed until they have abated, or until a stable situation has been reached. Depending on the event, follow up may require additional tests or medical procedures as indicated, and/or referral to the general physician or a medical specialist.
SAEs need to be reported till end of study within the Netherlands, as defined in the protocol

9.5 Data Safety Monitoring Board (DSMB)

Since both techniques are standard practice, and no major unexpected complications are foreseen, no interim analysis is planned. A Data Safety and Monitoring Board will not be installed, as both procedures are regularly used and acceptable options in current clinical practice.

10. STATISTICAL ANALYSIS

The results of the study will be reported according to the CONSORT statement.

The main outcomes will be analysed and presented according the intention-to-treat principle. Since in this pragmatic trial we expect that in the pessary strategy group 40% of women will cross over to surgery an additional per protocol analyses is foreseen. This will provide more insight in the effect of surgery after pessary therapy as compared to surgery or pessary therapy alone.

We plan a subgroup analysis for the location of the prolapse: anterior prolapse versus posterior prolapse.

10.1 Primary study parameter(s)

The primary outcome, success (much or very much improvement) or no success (a little better, no change, a little worse, much worse or very much worse) on the PGI-I will be expressed in percentage point differences. Differences between the percentages will be tested using a chi-square test. A p-value <0.05 will be considered statistically significant.

10.2 Secondary study parameter(s)

The PFDI, PFIQ and PISQ-r are all interval scales. Differences between baseline and 12 and 24 months follow up will be assessed using an independent t-test when normality can be assumed, or by non-parametric tests when the data are not normally distributed. Effect sizes will be calculated to estimate the magnitude of changes.

Differences in EQ5-D scores and “ziekteverzuim” between baseline and at 3, 6, 12 and 24 months will be assessed using t-test and further incorporated in the cost-effectiveness analyses.

Imputation statistics will be used or missing data.

(Serious) adverse events will be categorized and chi-square statistics, with calculation of relative risks when appropriate, will be applied in analyses.

Prediction model

A prediction model that uses predefined variables, as potential predictors of failure of pessary therapy, will be developed using multivariable regression analysis. Missing data will be imputed.

Predictors for failure derived from literature are a large genital hiatus (gh > 4 cm), women being sexually active, age > 65 years, prolapse POP-Q stage 3, previous hysterectomy. If applicable, a prediction rule for the chance of failure of pessary therapy will be constructed, which could be presented as a normogram which could be used to determine the chance of failure on pessary therapy.

Internal validity will be assessed using bootstrapping techniques; shrinkage will be applied to the parameter estimates. Model performance will be assessed with discriminative capacity and calibration. Calibration will be assessed by comparing the mean predicted probability that patients failed on pessary therapy with the mean observed probability that patients failed on pessary therapy. To do so, the total cohort will be split into ten groups based on the deciles of the predicted probability. Per group the mean predicted probability will be calculated as well as the mean observed predicted probability. Discriminative capacity of the model will be assessed with receiver operation characteristics (ROC) analysis and the area under the ROC curve (AUC).

We will also look at factors that could explain failure of surgery. Our systematic review on POP and recurrent POP after surgery showed that 6 preoperative items, eg. POP stage, age, family history, preoperative incontinence, previous POP or incontinence surgery, previous hysterectomy seems to be predictive for recurrence.

Women with previous POP, incontinence surgery or previous hysterectomy are excluded from our study, leaving 4 predefined potential predictive factors. After the 2 year follow-up has been performed, we will reconsider which factors to include in a prognostic model, based on the current literature. We will select predictors from literature with the highest predictive value, where about 1 predictor could be selected for each 10 surgery failures.

Using interaction terms the effect of a differential effect in women with a higher age (>median) or a lower age (<=median), a higher (>25) or lower BMI(<=25) will be assessed for both pessary as well as surgery failures prediction.

10.3 Other study parameters

Not applicable.

10.4 Interim analysis (if applicable)

Not applicable, because of the non-inferiority design with low risk and the possibility of cross over.

11. ETHICAL CONSIDERATIONS

11.1 Regulation statement

This study will be conducted according to the principles of the Declaration of Helsinki (version 10, October 2013) and in accordance with the Medical Research Involving Human Subjects Act (WMO) and other guidelines, regulations and Acts.

11.2 Recruitment and consent

Women with symptomatic pelvic organ prolapse who attend the outpatient clinic will be informed about the study by the gynaecologist or nurse. After checking the in- and exclusion criteria the women will receive verbal and written information about the study. If the woman is willing to participate she is asked to sign the informed consent. All women will undergo the pessary fitting test which is part of the standard evaluation during the first visit. All women will be contacted at a minimum interval of 1 week. Those women who failed the initial fitting will be offered surgery and attend the cohort, the women with a succesfull initial fitting will be asked to enroll in the RCT. In case the woman is willing to participate but actively opts for pessary therapy she will be provided with a pessary and enter the cohort.

11.3 Objection by minors or incapacitated subjects (if applicable)

Not applicable.

11.4 Benefits and risks assessment, group relatedness

The present study carries no risks for the participant. Pessary or surgery are standard care for symptomatic pelvic organ prolapse. The benefit of the study lies in a better understanding of satisfaction and cost effectiveness.

11.5 Compensation for injury

The sponsor/investigator has a liability insurance which is in accordance with article 7, subsection 9 of the WMO.

The sponsor (also) has an insurance which is in accordance with the legal requirements in the Netherlands (Article 7 WMO and the Measure regarding Compulsory Insurance for Clinical Research in Humans of 23th June 2003). This insurance provides cover for damage to research subjects through injury or death caused by the study.

1. € 450.000,-- (i.e. four hundred and fifty thousand Euro) for death or injury for each subject who participates in the Research;
2. € 3.500.000,-- (i.e. three million five hundred thousand Euro) for death or injury for all subjects who participate in the Research;
3. € 5.000.000,-- (i.e. five million Euro) for the total damage incurred by the organisation for all damage disclosed by scientific research for the Sponsor as 'verrichter' in the meaning of said Act in each year of insurance coverage.

The insurance applies to the damage that becomes apparent during the study or within 4 years after the end of the study.

11.6 Incentives (if applicable)

Not applicable.

12. ADMINISTRATIVE ASPECTS, MONITORING AND PUBLICATION

12.1 Handling and storage of data and documents

All data will be stored and will be coded. Only the researchers will be able to link patient ID and research code. The handling of personal data complies with the Dutch Personal Data Protection Act. After cessation of the study, patient material will be stored for a maximum of 15 years.

The case report forms and questionnaires will be filled in online. The head investigator will be able to check all the completed forms and questionnaires.

12.2 Monitoring and Quality Assurance

The monitoring will be coordinated by the Staff Member Clinical Research, quality coordinator of division women and baby en will be executed by a qualified intern monitor. This person is not involved in design and output of this research. The frequency of checking will be every year. The monitoring plan is discussed in section K of the METC dossier.

12.3 Amendments

All substantial amendments will be notified to the METC.

Non-substantial amendments will not be notified to the accredited METC, but will be recorded and filed by the sponsor.

12.4 Annual progress report

The sponsor/investigator will submit a summary of the progress of the trial to the accredited METC once a year. Information will be provided on the date of inclusion of the first subject, numbers of subjects included and numbers of subjects that have completed the trial, serious adverse events/ serious adverse reactions, other problems, and amendments.

12.5 End of study report

The investigator will notify the accredited METC of the end of the study within a period of 8 weeks. The end of the study is defined as the last patient’s last visit.

In case the study is ended prematurely, the investigator will notify the accredited METC within 15 days, including the reasons for the premature termination.

Within one year after the end of the study, the investigator/sponsor will submit a final study report with the results of the study, including any publications/abstracts of the study, to the accredited METC.

12.6 Public disclosure and publication policy

The research findings will be published in peer reviewed journals.

13. STRUCTURED RISK ANALYSIS

Not applicable because this study is a low risk study concerning standard care.

13.1 Potential issues of concern

Not applicable.

13.2 Synthesis

Not applicable.

14. REFERENCES

1: Slieker-ten Hove MC et al. The prevalence of pelvic organ prolapse symptoms and signs and their relation with bladder and bowel disorders in a general female population. Int Urogynecol J. 2009;20(9):1037-45
2. Tegerstedt G, Maehle-Schmidt M, Nyrén O, Hammarström M. Prevalence of symptomatic pelvic organ prolapse in a Swedish population. Int Urogynecol J Pelvic Floor Dysfunct. 2005 Nov-Dec;16(6):497-503
3: Lamers BH et al. Pessary treatment for pelvic organ prolapse and health-related quality of life: a review. Int Urogynecol J. 2011;22(6):637-44
4: Broekman BM et al. Prospective pilot study in 65 women on pessary treatment as primary treatment, unpublished data
5:Broens-Oostveen WC, Mom RM, Lagro-Janssen ALM. De genitale prolaps: behnadleing en beloop in vier huisartspraktijken. Ned Tijdschr Geneeskd 2004, 148 (29); 1444-1448
6: Withagen M et al. Sacrospinous hysteropexy or modified manchester for the treatment of pelvic organ prolapse? Int J Gynaecol Obstet. 2013; Abstract Supplement
7: Vollebregt A et al. Cost-utility analysis of trocar-guided transobturator mesh versus anterior colporrhaphy for primary cystocele repair: a randomised trial. [in preparation]
8: Barber MD[1], Walters MD, Bump RC. Short forms of two condition-specific quality-of- life questionnaires for women with pelvic floor disorders (PFDI-20 and PFIQ-7). Am J Obstet Gynecol. 2005 Jul;193(1):103-13.
9: Pollard ME, Eilber KS, Anger JT. Abdominal approaches to pelvic prolapse repairs. Curr Opin Urol. 2013;23(4):306-11.
10: Dietz V et al. Vaginal surgery for uterine descent; which options do we have? A review of the literature. Int J Gynaecol Obstet. 2009;20(3):349-56
11: C. Maher, Feiner B, Baessler K, Schmid C. Surgical management of pelvic organ prolapse in women. Cochrane 2013
12: de Boer TA, Milani AL, Kluivers KB, Withagen MI, Vierhout ME. The effectiveness of surgical correction of uterine prolapse: cervical amputation with uterosacral ligament plication (modified Manchester) versus vaginal hysterectomy with high uterosacral ligament plication. Int Urogynecol J Pelvic Floor Dysfunct. 2009 Nov;20(11):1313-9
13: Bugge C et al. Pessaries (mechanical devices) for pelvic organ prolapse in women. Cochrane database syst rev, 2013;28;2:CD004010
14: van Geelen JM, Dwyer PL. Where to for pelvic organ prolapse treatment after the FDA pronouncements? A systematic review of the recent literature. Int Urogynecol J. 2013 May;24(5):707-18
15: Abdool Z et al. Prospective evaluation of outcome of vaginal pessaries versus surgery in women with symptomatic pelvic organ prolapse. Int Urogynecol J. 2011;22(3):273-8
16: Hullfish KL, Trowbridge ER, Stukenborg GJ. Treatment strategies for pelvic organ prolapse: a cost-effectiveness analysis. Int Urogynecol J. 2011 May;22(5):507-15
17: Cundiff GW, Amundsen CL, Bent AE, Coates KW, Schaffer JI, Strohbehn K, Handa VL. The PESSRI study: symptom relief outcomes of a randomized crossover trial of the ring and Gellhorn pessaries. Am J Obstet Gynecol. 2007 Apr;196(4):405.e1-8
18: Weber MA, Limpens J, Roovers JPWR. Assessment of vaginal atrophy: a review [submitted BJOG]
19: Cobra Ok-Klapper 2012. DCHG 2012
20: Detollenaere RJ, den Boon J, Kluivers KB, Vierhout ME, van Eijndhoven HW.Surgical management of pelvic organ prolapse and uterine descent in the Netherlands. Int Urogynecol J. 2013 May;24(5):781-8
21.Detollenaere RJ, den Boon J, Vierhout ME, van Eijndhoven HW. Uterus preserving surgery versus vaginal hysterectomy in treatment of uterine descent: a systematic review]. Ned Tijdschr Geneeskd. 2011;155(42):A3623

22: Yalcin I, Bump RC. Validation of two global impression questionnaires for incontinence. Am J Obstet Gynecol. 2003 Jul;189(1):98-101
23: Barber MD, Kuchibhatla MN, Pieper CF, Bump RC. Psychometric evaluation of 2 comprehensive condition-specific quality of life instruments for women with pelvic floor disorders. Am J Obstet Gynecol 2001;185(6):1388–1395.
24: Utomo E, Blok BF, Steensma AB, Korfage IJ. Validation of the Pelvic Floor Distress Inventory (PFDI-20) and Pelvic Floor Impact Questionnaire (PFIQ-7) in a Dutch population. Int Urogynecol J. 2014;25(4):531-44.
25: Euroquol—a new facility for the measurement of health-related quality of life. The EuroQol Group. Health Policy. 1990;16:199-208
26: Rogers RG, Rockwood TH, Constantine ML, Thakar R, Kammerer-Doak DN, Pauls RN, Parekh M, Ridgeway B, Jha S, Pitkin J, Reid F, Sutherland SE, Lukacz ES, Domoney C, Sand P, Davila GW, Espuna Pons ME. A new measure of sexual function in women with pelvic floor disorders (PFD): the Pelvic Organ Prolapse/Incontinence Sexual Questionnaire, IUGA-Revised (PISQ-IR). Int Urogynecol J. 2013 Jul;24(7):1091-103
27: Cabana MD, Rand CS, Powe NR, Wu AW, Wilson MH, Abboud PA, Rubin HR. Why don't physicians follow clinical practice guidelines? A framework for improvement. JAMA. 1999 Oct 20;282(15):1458-65
28: Thys SD, Roovers JP, Geomini PM, Bongers MY. Do patients prefer a pessary or surgery as primary treatment for pelvic organ prolapse. Gynecol Obstet Invest. 2012;74(1):6-12
29: www.imta.nl (C. Bouwmans et al. Handleiding iMTA Medical Cost Questionnaire (iMCQ). Rotterdam: iMTA, Erasmus Universiteit Rotterdam, 2013)
30: www.imta.nl (C. Bouwmans et al. Handleiding iMTA Productivity Cost Questionnaire (iPCQ). Rotterdam: iMTA, Erasmus Universiteit, 2013)
31:Hakkaart- van Roijen, S.S. Tan, C.A.M. Bouwmans: Handleiding voor Kostenonderzoek. Methoden en standaard kostprijzen voor economische evaluaties in de gezondheidszorg. CVZ 2010
32: Oostenbrink JB, Koopmanschap MA, Rutten FF. Standardisation of costs: the Dutch Manual for Costing in economic evaluations. Pharmacoeconomics 2002;20(7):443-454
33: Mauskopf JA, Sullivan SD, Annemans L et al. Principles of good practice for budget impact analysis: report of the ISPOR Task Force on good research practices--budget impact analysis. Value Health 2007;10(5):336-347 -> In BIA, klopt niet, nog vervangen door:

References (Cost) effectiveness of pessary use as compared to surgery systematic review

1: Mamik MM, Rogers RG, Qualls CR, Komesu YM. Goal attainment after treatment in patients with symptomatic pelvic organ prolapse. Am J Obstet Gynecol. 2013 Nov;209(5):488.e1-5.
2: Abdool Z, Thakar R, Sultan AH, Oliver RS. Prospective evaluation of outcome of vaginal pessaries versus surgery in women with symptomatic pelvic organ prolapse. Int Urogynecol J. 2011 Mar;22(3):273-8.
3: Lowenstein L, Gamble T, Sanses TV, van Raalte H, Carberry C, Jakus S, Pham T, Nguyen A, Hoskey K, Kenton K; Fellow's Pelvic Research Network. Changes in sexual function after treatment for prolapse are related to the improvement in body image perception. J Sex Med. 2010 Feb;7(2 Pt 2):1023-8.
4: Barber MD, Walters MD, Cundiff GW; PESSRI Trial Group. Responsiveness of the Pelvic Floor Distress Inventory (PFDI) and Pelvic Floor Impact Questionnaire (PFIQ) in women undergoing vaginal surgery and pessary treatment for pelvic organ prolapse. Am J Obstet Gynecol. 2006 May;194(5):1492-8.

References Systematic review of factors influencing pessary fitting and continuation

1. Abdool Z, Thakar R, Sultan AH, Oliver RS (2010) Prospective evaluation of outcome of vaginal pessaries versus surgery in women with symptomatic pelvic organ prolapse. Int Urogynecol J Pelvic Floor Dysfunct 16
2. Alperin M, Khan A, Dubina E, Tarnay C, Wu N, Pashos CL, Anger JT.Female Pelvic Med Reconstr Surg. 2013 May-Jun;19(3):142-7.Patterns of pessary care and outcomes for medicare beneficiaries with pelvic organ prolapse.
3. Bai SW, Yoon BS, Kwon JY, Shin JS, Kim SK, Park KH (2005) Survey of the characteristics and satisfaction degree of the patients using a pessary. Int Urogynecol J Pelvic Floor Dysfunct 16 (3):182–186
4. Barber MD, Walters MD, Cundiff GW (2006) Responsiveness of the Pelvic Floor Distress Inventory (PFDI) and Pelvic Floor Impact Questionnaire (PFIQ) in women undergoing vaginal surgery and pessary treatment for pelvic organ prolapse. Am JObstet Gynecol 194(5):1492–149
5. Brincat C, Kenton K, Pat FM, Brubaker L (2004) Sexual activity predicts continued pessary use. Am J Obstet Gynecol 191(1):198–200
6. Broens-Oostveen MC, Mom RM, Lagro-Janssen AL (2004)Genital prolapse; treatment and course in four general practices.Ned Tijdschr Geneeskd 148(29):1444–1448, 17
7. Chan SS, Cheung RY, Yiu KW, Lee LL, Pang AW, Chung TK. Int Urogynecol J. 2012 Aug;23(8):1027-33. Symptoms, quality of life, and factors affecting women's treatment decisions regarding pelvic organ prolapse.
8. Clemons JL, Aguilar VC, Sokol ER, Jackson ND, Myers DL (2004) Patient characteristics that are associated with continued pessary use versus surgery after 1 year. Am J Obstet Gynecol 191(1):159–164
9. Cundiff GW, Amundsen CL, Bent AE, Coates KW, Schaffer JI, Strohbehn K et al (2007) The PESSRI study: symptom relief outcomes of a randomized crossover trial of the ring and Gellhorn pessaries. Am J Obstet Gynecol 196(4):405–408
10. Fernando RJ, Thakar R, Sultan AH, Shah SM, Jones PW (2006) Effect of vaginal pessaries on symptoms associated with pelvic organ prolapse. Obstet Gynecol 108(1):93–99
11. Friedman S, Sandhu KS, Wang C, Mikhail MS, Banks E (2010) Factors influencing long-term pessary use. Int Urogynecol J Pelvic Floor Dysfunct 21(6):673–678
12. Hanson LA, Schulz JA, Flood CG, Cooley B, Tam F (2006) Vaginal pessaries in managing women with pelvic organ prolapse and urinary incontinence: patient characteristics and factors contributing to success. Int Urogynecol J Pelvic Floor Dysfunct 17(2):155–159
13. Jones K,Yang L, Lowder JL, Meyn L, Ellison R, ZyczynskiHMet al (2008) Effect of pessary use on genital hiatus measurements in women with pelvic organ prolapse. Obstet Gynecol 112(3):630–636
14. Geoffrion R, Zhang T, Lee T, Cundiff GW.Female Pelvic Med Reconstr Surg. 2013 Nov-Dec;19(6):339-45. Clinical characteristics associated with unsuccessful pessary fitting outcomes.
15. Komesu YM, Rogers RG, Rode MA, Craig EC, Gallegos KA, Montoya AR et al (2007) Pelvic floor symptom changes incpessary users. Am J Obstet Gynecol 197(6):620–626
16. Kuhn A, Bapst D, Stadlmayr W, Vits K, Mueller MD (2009) Sexual and organ function in patients with symptomatic prolapse: are pessaries helpful? Fertil Steril 91(5):1914–1918
17. Lone F1, Thakar R, Sultan AH, Karamalis G. A 5-year prospective study of vaginal pessary use for pelvic organ prolapse. Int J Gynaecol Obstet. 2011 Jul;114(1):56-9.
18. Maito JM, Quam ZA, Craig E, Danner KA, Rogers RG (2006) Predictors of successful pessary fitting and continued use in a nurse-midwifery pessary clinic. J Midwifery Womens Health 51 (2):78–84
19. Mamik MM, Rogers RG, Qualls CR, Komesu YM. Goal attainment after treatment in patients with symptomatic pelvic organ prolapse. Am J Obstet Gynecol. 2013 Nov;209(5):488.e1-5.
20. Manchana T, Bunyavejchevin S. Impact on quality of life after ring pessary use for pelvic organ prolapse. Int Urogynecol J. 2012 Jul;23(7):873-7.

21. Markle D, Skoczylas L, Goldsmith C, Noblett K. Patient characteristics associated with a successful pessary fitting. Female Pelvic Med Reconstr Surg. 2011 Sep;17(5):249-52.
22. Mokrzycki ML, Hatangadi SB, Zaccardi JE, Cox S. Preexisting stress urinary incontinence: a predictor of discontinuation with pessary management. J Low Genit Tract Dis. 2001 Oct;5(4):204-7.
23. Mutone MF, Terry C, Hale DS, Benson JT (2005) Factors which influence the short-term success of pessary management of pelvic organ prolapse. Am J Obstet Gynecol 193(1):89–94
24. Nguyen JN, Jones CR. Pessary treatment of pelvic relaxation: factors affecting successful fitting and continued use. J Wound Ostomy Continence Nurs. 2005;32(4):255-61; quiz 262-3.
25. Patel M, Mellen C, O'Sullivan DM, Lasala CA. (2010) Impact ofpessary use on prolapse symptoms, quality of life, and bodyimage. Am J Obstet Gynecol 18
26. Powers K, Lazarou G, Wang A, LaCombe J, Bensinger G.Greston WM et al (2006) Pessary use in advanced pelvic organprolapse. Int Urogynecol J Pelvic Floor Dysfunct 17(2):160–164.

Reference list Systematic review of factors influencing failure of POP surgery

1. Whiteside JL, Weber AM, Meyn LA, Walters MD. Am J Obstet Gynecol. 2004 Nov;191(5):1533-8.factors for prolapse recurrence after vaginal repair.
2. Diez-Itza I, Aizpitarte I, Becerro A. Int Urogynecol J Pelvic Floor Dysfunct. 2007 Nov;18(11):1317-24. Risk factors for the recurrence of pelvic organ prolapse after vaginal surgery: a review at 5 years after surgery.
3. Fialkow MF, Newton KM, Weiss NS. Int Urogynecol J Pelvic Floor Dysfunct. 2008 Nov;19(11):1483-7.Incidence of recurrent pelvic organ prolapse 10 years following primary surgical management: a retrospective cohort study.
4. Jeon MJ, Chung SM, Jung HJ, Kim SK, Bai SW. Gynecol Obstet Invest. 2008;66(4):268-73 Risk factors for the recurrence of pelvic organ prolapse.
5. Dällenbach P, Jungo Nancoz C, Eperon I, Dubuisson JB, Boulvain M. Int Urogynecol J. 2012 Jan;23(1):35-41. Incidence and risk factors for reoperation of surgically treated pelvic organ prolapse.
6. Weemhoff M, Vergeldt TF, Notten K, Serroyen J, Kampschoer PH, Roumen FJ. Int Urogynecol J. 2012 Jan;23(1):65-71. Avulsion of puborectalis muscle and other risk factors for cystocele recurrence: a 2-year follow-up study.
7. Tegerstedt G1, Hammarström M. Acta Obstet Gynecol Scand. 2004 Aug;83(8):758-63. Operation for pelvic organ prolapse: a follow-up study.
8. Medina CA, Candiotti K, Takacs P. Int J Gynaecol Obstet. 2008 May;101(2):184-7. Wide genital hiatus is a risk factor for recurrence following anterior vaginal repair.
9. Salvatore S1, Athanasiou S, Digesu GA, Soligo M, Sotiropoulou M, Serati M, Antsaklis A, Milani R. Neurourol Urodyn. 2009;28(4):301-4. doi: 10.1002/nau.20639. Identification of risk factors for genital prolapse recurrence.
10. Kawasaki A, Corey EG, Laskey RA, Weidner AC, Siddiqui NY, Wu JM. J Reprod Med. 2013 May-Jun;58(5-6):195-9. Obesity as a risk for the recurrence of anterior vaginal wall prolaps after anterior colporrhaphy.

Appendix 1:

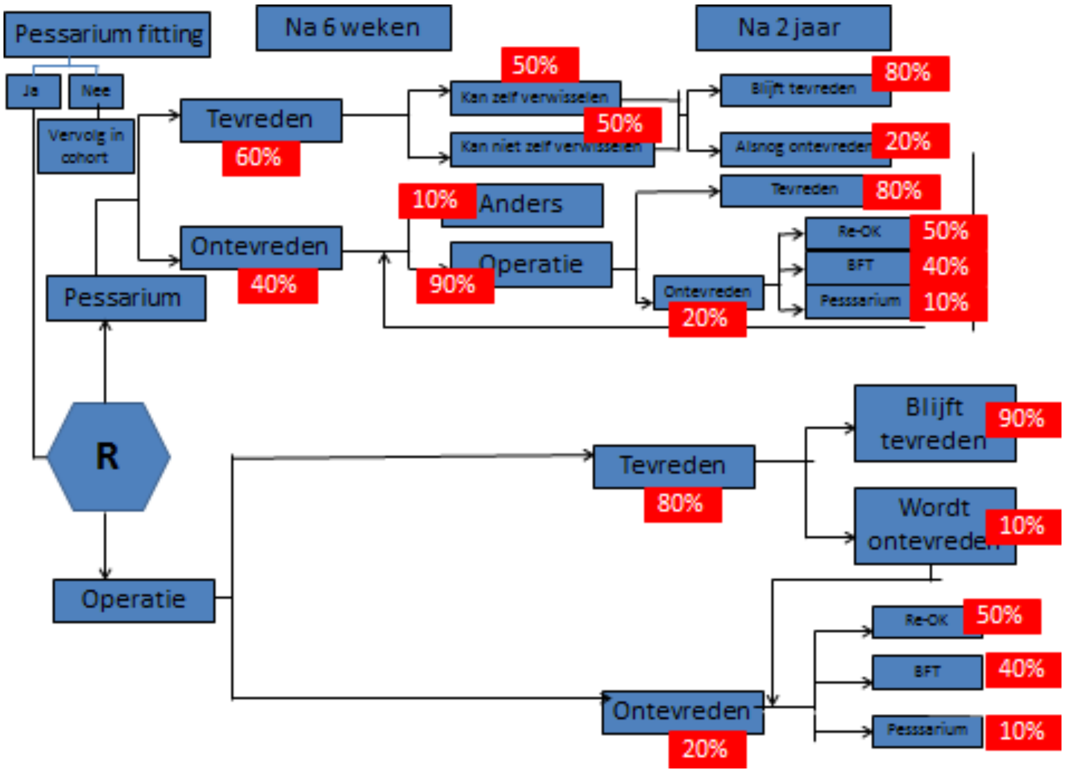
Pessarium fitting
Na 6 weken
Na 2 jaar
Ja
Nee
Vervolg in cohort
Tevreden
60%
Kan zelf verwisselen
50%
Kan niet zelf verwisselen
50%
Blijft tevreden
80%
Alsnog ontevreden
20%
10%
Anders
Tevreden
80%
Ontevreden
40%
Operatie
90%
Re-OK
50%
BFT
40%
Pessarium
10%
Ontevreden
20%
Pessarium
R
Blijft tevreden
90%
Tevreden
80%
Wordt ontevreden
10%
Operatie
Re-OK
50%
BFT
40%
Ontevreden
20%
Pessarium
10%

Appendix 2 Review on (Cost) effectiveness of pessary use as compared to surgery:

Study protocol

Reference	Study type	Characteristics	Intervention (I)	Controls (C)	Outcome measures and follow-up time	Results
**Mamik, 2012**<br><br>**AJOG 2013:209:488**	**Design**<br>**Case-control**<br><br>**N = 100**<br><br>**Country**<br>**US**	**Aim: compare goal achievement and global improvement between pessary and surgery for POP stage ≥2.**<br><br>**Inclusion criteria: >18 year old, read and write in English**<br><br>**Exclusion: not given**	**Vaginal pessary**<br>**N = 50**	**Prolapse surgery**<br>**N = 50**	**Primary outcome:**<br>**Goal attainment**<br><br>**Secondary:**<br>**PGI-I**<br>**PFDI-20**<br>**PISQ-12**<br>**Body Image scale**<br><br>**Follow-up:**<br>**3 months**	**Primary outcome:**<br>**Goal attainment sign. higher score after surgery (8.6 vs 6.4)**<br><br>**Secondary outcomes**<br>**PGI-I sign (p=0.04) better improvement after surgery (2.4 vs 1.9 points)**<br>**PFDI-20 sign (p=0.02) higher change (89 vs 43 points)**<br>**PISQ-12 and BIS no sign difference**<br><br>**Additional: 10% crossed over from pessary to surgery within 3 months and 10% referred from surgery after they had been selected as eligible.**<br>**No follow-up in pessary group is 40% (20/50) and surgery 30% (15/50)**
**Abdool, 2011**	**Design**<br>**Cohort study**<br><br>**N total = 554**<br><br>**Country:**<br>**UK**	**Aim of the study**<br>**to evaluate and compare the effectiveness of pessaries and surgery in women with symptomatic pelvic organ prolapse.**<br><br>**Inclusion criteria**<br>**- Women referred to a specialist urogynaecology unit with symptomatic POP between June 2002 and May 2007**<br><br>**Exclusion criteria**<br>**- Subjects fitted with pessaries for urinary incontinence and those who had concomitant urinary incontinence surgery (e.g. TVT)**<br>**- Subjects who started in the pessary group but subsequently requested surgery were excluded from**	**Intervention**<br>**vaginal pessary**<br><br>**N = 359**	**Controls**<br>**surgery**<br><br>**N = 195**	**Primary outcomes:**<br>**Sheffield POP questionnaire (SPS-Q)**<br><br>**Secundary outcomes:**<br>**None**<br><br>**Follow up:**<br>**For the surgery and pessary groups 14 months (SD 6.14)**<br>**and 12 months (SD 3.1), respectively.**	**Primary outcomes:**<br>**No difference in functional outcome after 1 year follow-up between groups**<br><br>**Additional:**<br>**Only 45% in pessary group en 55% in surgery group responded at 12 months**<br>**In pessary group 24.7% (89/359) crossed to surgery but were not analyzed**<br>**In pessary group 7.3% stopped because of other reasons.**<br>**Selection and patient preference bias**<br>**The mean age was significantly higher in the pessary group compared to the surgery group (68.4 +/− 13.08 vs 60.4 +/−12.25 years, respectively).**

		**analysis in both the surgery and pessary group.**				

Study protocol

**Lowenstein 2010**<br><br>**J Sex Med 2010; 7: 1023-28**	**Design**<br>**Cohort study**<br>**N= 235**<br><br>**Country**<br>**US**	**Aim of the study**<br>**First to evaluate patient-reported outcome, POP symptoms, sexual functioning and body image following treatment of POP. Second to compare surgery with pessary**<br><br>**Inclusion: ≥18 year, ≥ satge 2 POP, complete questionnaire at baseline and at ≥6 months follow up**<br><br>**Exclusion: recurrent UTI, peripheral neuropathy, using pessary at initial presentation or POP surgery < 6 months prior to presentation**	**Intervention**<br>**N = 202 surgery**	**Controls**<br>**N = 33 pessary**	**Primary outcomes**<br>**PFDI-20**<br>**PISQ-12**<br>**Modified Body Image scale**<br><br>**All at six months follow-up**	**Results**<br>**After multivariate analyses, including type of intervention, BMI and difference in Body image were associated with change in total PISQ (sexual functioning) score**<br><br>**In the pessary group there was no significant improvement in sexual functioning as compared to surgery (-2.5 versus +11.5)**<br><br>**Additional:**<br>**No figures presented for pessary and surgery group, with exemption of the Sexual functioning (PISQ-12) result above.**
**Barber, 2006**	**Design**<br>Case-control study<br><br>**N total = 106**<br><br>**Country:** USA	**Aim of the study**<br>**to evaluate the responsiveness of the Pelvic Floor Distress Inventory (PFDI) and Pelvic Floor Impact Questionnaire (PFIQ) in women with pelvic organ prolapse undergoing surgical and nonsurgical management.**<br><br>**Inclusion criteria**<br>**Surgery group:**<br>**Stage III or IV prolapse, were at least 18 years, and scheduled for vaginal prolapse repair.**<br>**Pessary group:**<br>**women with symptomatic pelvic organ prolapse of stage II or greater. (Pessri trial)**<br><br>**Exclusion criteria**<br>**Surgery group:**<br>**- mentally or physically incapable of completing the questionnaires.**<br>**Pessary group:**<br>**- were pregnant, were currently using a pessary, or had vaginal agglutination**	**Intervention**<br>**Pessary in women with stage II or greater POP**<br><br>**N = 42**	**Controls**<br>**Surgery in women with stage III or greater POP**<br><br>**N = 64**	**Primary outcomes:**<br>**PFDI and PFIQ**<br><br>**Secundary outcomes:**<br><br>**Follow up:**<br>**3 months (Pessary group) or 6 months (Surgery group) after initiation of treatment.**	**Primary outcomes:**<br><br>**After controlling for preoperative prolapse stage and baseline HRQOL scores, subjects in the Surgery group had significantly greater improvement in each of the scales of the PFDI and the prolapse and urinary scales of the PFIQ than did the Pessary group.**<br><br>**Scores from each of the scales of the PFDI improved by 14 to 15 points more on average after treatment in the Surgery group than those of the Pessary group (P < .01 for each) after adjusting for the above baseline differences.**<br><br>**Similarly, for the prolapse and urinary scales of the PFIQ, scores improved 13 and 17 points more, respectively, in the Surgery group than the Pessary group after treatment. (P < .05 for each).**<br><br>**Four of 64 (6%) of subjects in the Surgery group had recurrent prolapse develop beyond the hymen by 6 months after surgery. No subjects underwent reoperation for recurrent prolapse during the study period.**<br><br>**Additional:**<br><br>**Difference in follow up**<br>**Selection bias**

		**that precluded pessary insertion.**				

Study protocol

Appendix 3 Review on risk factors for failure of pessaries:

study		n	FU period	success initial	continued pes	stopped pes	cross over surgery	predict fit	predict contin
Wu 1997	retrospective	110	3 yrs	81 (74%)	43 (39%)		at least 5	nee	ja
Mokrzycki 2001	retrospective	42	3 mth		24 (57%)	18 (43%)	nr	nee	ja
Clemons 2004	prospective	100	1 yr	73 (73%)	43 (43%)		16/73 (27%)	nee	ja
Brincat 2004	retrospective	169	2,5 yr	144 (85%)	82 (60%)	54 (40%)	32/144 (22%)	nee	ja
Mutone 2004	retrospective	407	3 wks	168 (41%)		96 (57%)	nr	ja	ja
Powers 2006	retrospective	32	1 yr			20 (62%)	12 (38%)	nee	ja
Broens-Oostveen 2004	retrospective	192	9 yr			107 (52%)	nr	nee	ja
Nguyen 2005	retrospective	130	1 yr	111 (82%)	85 (65%)		20 (15%)	ja	ja
Hanson 2005	retrospective	1216	3 mth	1043 (86%)	744 (71%)		nr	nee	ja
Fernando 2006	prospective	203	4 mth	nr	153 (75%)		28 (14%)	nee	ja
Barber 2006	prospective	42	3 mth	nr		0	nr	nee	nee
Maito 2006	retrospective	120	6 mth	106 (88%)	92 (77%)		9 (8%)	ja	ja
Cundiff 2007	prospective	85	1 yr	65/71 (92%)			5 (6%)	nee	nee
Komesu 2007	prospective	64	6-12 mth		36 (56%)	28 (44%)	2 (3%)	nee	ja
Jones 2008	prospective	90	3 mth		42 (47%)		nr	nee	ja
Kuhn 2009	prospective	71	3 mth		32 (45%)		nr	nee	nee
Sarma 2009	retrospective	120	1 mth		45 (38%)		18 (15%)	nee	nee
Friedman 2010	retrospective	150	1 yr		115 (77%)	35 (23%)	25 (17%)	nee	ja
Patel 2010	prospective	65	3 mth	54 (83%)	54 (83%)		5 (8%)	nee	nee
Abdool 2010	prospective	359	1 yr	296 (83%)	243 (68%)		31 (9%)	nee	nee
Lone 2011	prospective	246	5 yr	187 (76%)	130 (53%)		70 (28%)	nee	nee
Markle 2011	retrospective	158	1 week	92 (59%)			nr	ja	nee
Chan 2012	prospective	197	1 yr	138 (70%)		59 (30%)	nr	nee	ja
Manchana 2012	retrospective	126	1 yr	77 (61%)	64 (51%)		5 (4%)	nee	nee
Mamik 2013	prospective	50	3 mth				5 (10%)	nee	nee
Geoffrion 2013	retrospective	101	4 wks	79 (78%)			nr	nee	ja
Alperin 2013	retrospective	4019	9 years				12% 1 yr, 24% 9 yrs	nee	nee
Succesfull fitting		3589		2714(76%)					
Continuation		3381			2027 (60%)				

Study protocol

Appendix 4 Review on risk factors for failure of surgery:

Risk factor	Investigated:	Significant:
Preoperative stage	8	5
Age	8	2
Obesity	7	0
Parity	5	0
Constipation	5	0
Pulmonary disease	5	0
Number of sites involved preoperative	4	1
Menopausal status	4	0
Hysterectomy status	4	0
Concomitent surgery	3	1
Family history	3	1
Complicated delivery	3	0
Diabetes	3	0
Smoking	3	0
Previous incontinence and/or prolapse surgery	2	2
Hiatus genitalis	2	1
Weight	2	1
Any incontinence preoperative	2	1
Delivery mode	2	0
Vaginal delivery	2	0
Hormone replacement therapy	2	0
Previous prolapse surgery	2	0
Surgeons experience	2	0
Abcense of posterior repair	1	1
Sexual activity	1	1
Levator defect	1	1
Height	1	0
Birth weight	1	0
Age at last delivery	1	0
Site of most advanced prolapse	1	0
Surgical approach	1	0
Use of Mesh	1	0
Previous incontinence surgery	1	0
Previous pelvic floor surgery or hysterectomy	1	0
Abdominal hernias	1	0
Cardiovascular disease	1	0
Intense physical exercise	1	0
Heavy lifting	1	0
Heavy lifting or constipation	1	0
Levator muscle contraction	1	0
Weight of the uterus	1	0
Postoperative complications	1	0
Incomplete emptying of bladder	1	0
Fecal incontinence	1	0

Ben ÂJ, *et al. BMJ Open* 2024; 14:e075016. doi: 10.1136/bmjopen-2023-075016

Appendix 5 tabel bezoeken, tijdstippen, onderzoeken

Chirurgie en cohort

Contact	Bezoek arts	POPQ	Eq5d doktersbezoek werkverzuim	PFIQ PFDI PISQ PGII PGIS
1. Eerste bezoek	X	X	Eq5D	X (zonder PGII)
2. 6 weken	X	X		
3. 3 maanden			X	
4. 6 maanden			X	
5. 12 maanden	X	X	X	X
6. 24 maanden	X	X	X	X

Pessarium met zelfmanagement

Contact	Bezoek arts	POPQ	Eq5d doktersbezoek werkverzuim	PFIQ PFDI PISQ PGII PGIS
1. Eerste bezoek	X	X	Eq5D	X (zonder PGII)
2. 6 weken	X	X		
3. 3 maanden			X	
4. 6 maanden			X	
5. 12 maanden	X	X	X	X
6. 24 maanden	X	X	X	X

Pessarium zonder zelfmanagement

Contact	Bezoek arts	POPQ	Ringcontrole	Eq5d doktersbezoek werkverzuim	PFIQ PFDI PISQ PGII PGIS
1. Eerste bezoek	X	X		Eq5D	X (zonder PGII)
2. 6 weken	X	X			
3. 3 maanden				X	
4. 4 maanden	X		X		
5. 6 maanden				X	
6. 8 maanden	X		X		
7. 12 maanden	X	X	X	X	X
8. 16 maanden	X		X		
9. 20 maanden	X		X		
10. 24 maanden	X	X	X	X	X

Study protocol

1.2 Final study protocol

Pessary or surgery for symptomatic pelvic organ prolapse

Version 1.~~21~~ 22 ~~April 2017~~February 2018

Study protocol

PROTOCOL TITLE ‘Pessary or surgery for symptomatic pelvic organ prolapse’

**Protocol ID**	**~~2017~~ 2018 / 1.~~21~~22**
**Short title**	**Pessary or surgery for symptomatic pelvic organ prolapse**
**EudraCT number**	***Not applicable***
**Version**	**1.~~21~~22**
**Date**	**April 2017**
**Coordinating investigator/project leader**	**Prof. Dr. C.H. van der Vaart, gynaecologist<br>University Medical Centre Utrecht**
**Principal investigator(s) (in Dutch: hoofdonderzoeker/ uitvoerder)**	**Dr. A. Vollebregt, gynaecologist<br>Spaarne Hospital<br><br>M.K. van de Waarsenburg, MD<br>University Medical Centre Utrecht**
**Multicenter: per site**	**Prof. Dr. C.H. van der Vaart, UMC Utrecht / Bergman Clinics Bilthoven<br>Dr. A.L. Milani, Reinier de Graaf Gasthuis<br>Dr. A. Vollebregt, Spaarne Hospital Hoofddorp<br>Dr. J.P.W.R. Roovers, Academic Medical Center / Bergman Clinics Amsterdam<br>Dr. K.B. Kluivers, Radboud UMC Nijmegen<br>Dr. V. Dietz, Catharina Hospital Eindhoven<br>Dr. H.W.F. van Eijndhoven, Isala Clinic Zwolle<br>Dr. M.M.A. Vernooij, Sint Antonius Hospital**

	Nieuwegein<br>Dr. R.P. Schellart, Kennemer Gasthuis Haarlem<br>Drs. A.M.W. Broekman, Sint Franciscus Gasthuis Rotterdam<br>Drs. J. van Bavel, Amphia Hospital Breda<br>Dr. M. Weemhoff, Atrium MC Heerlen<br>Dr. G. LinkW.A. Spaans, Academisch ziekenhuis Maastricht<br>Dr. J.M. van der Ploeg, Martini ziekenhuis Groningen<br>Dr. D. Massop-Helmink, MST Enschede<br>Dr. J.M. Sikkema, ZGT Almelo / Hengelo<br>Dr. L. van der Voet, Deventer ziekenhuis<br>Dr. J.P. de Bruin, Jeroen Bosch ziekenhuis<br>Dr. C. Zeijl, Amstelland ziekenhuis<br>Dr. G.G. Bon, Tergooi ziekenhuis<br>Dr. S. de Vos – Stekelenburg, Albert Schweitzer ziekenhuis<br>Drs. K.L. Bos, Canisius Wilhelmina ziekenhuis<br>Prof. Dr. M.Y. Bongers – Maxima Medisch Centrum<br>Dr. W. Hermes – MCH-Bronovo<br>Dr. E. Janszen – OLVG<br>Dr. T. Huisman - HAGA
Sponsor (in Dutch: verrichter/opdrachtgever)	Prof. Dr. C.H. van der Vaart, gynaecologist<br>University Medical Centre Utrecht
Subsidising party	ZonMw Project nr 837002525
Independent expert (s)	Dr. R.P. Zweemer<br>University Medical Centre Utrecht

**Laboratory sites <*if applicable*>**	***Not applicable***
**Pharmacy <*if applicable*>**	***Not applicable***

Study protocol

PROTOCOL SIGNATURE SHEET

Name	Signature	Date
**Head of Department:**<br><br>**Prof. Dr. B. Veersema**<br>**Department of Reproductive Medicine and Gynaecology**<br>**University Medical Centre Utrecht**		
**Coordinating Investigator/Project leader:**<br><br>**Prof. Dr. C.H. van der Vaart, gynaecologist**		
**M.K. van de Waarsenburg, MD**<br>**University Medical Centre Utrecht**		

Ben ÂJ, *et al. BMJ Open* 2024; 14:e075016. doi: 10.1136/bmjopen-2023-075016

Study protocol

TABLE OF CONTENTS

1. INTRODUCTION AND RATIONALE 64
Problem definition 64
Relevance 65
2. OBJECTIVES 68
3. STUDY DESIGN 69
4. STUDY POPULATION 70
4.1 Population (base) 70
4.2 Inclusion criteria 70
4.3 Exclusion criteria 70
4.4 Sample size calculation 70
5. TREATMENT OF SUBJECTS 72
5.1 Investigational product/treatment 72
5.2 Use of co-intervention (if applicable) 73
5.3 Escape medication (if applicable) 73
6. INVESTIGATIONAL PRODUCT 74
6.1 Name and description of investigational product(s) 74
6.2 Summary of findings from non-clinical studies 74
6.3 Summary of findings from clinical studies 74
6.4 Summary of known and potential risks and benefits 75
6.5 Description and justification of route of administration and dosage 75
6.6 Dosages, dosage modifications and method of administration 75
6.7 Preparation and labelling of Investigational Medicinal Product 75
6.8 Drug accountability 75
7. NON-INVESTIGATIONAL PRODUCT 76
7.1 Name and description of non-investigational product(s) 76
7.2 Summary of findings from non-clinical studies 76
7.3 Summary of findings from clinical studies 76
7.4 Summary of known and potential risks and benefits 76
7.5 Description and justification of route of administration and dosage 76
7.6 Dosages, dosage modifications and method of administration 76
7.7 Preparation and labelling of Non Investigational Medicinal Product 76
7.8 Drug accountability 76
8. METHODS 77
8.1 Study parameters/endpoints 77
8.1.1 Main study parameter/endpoint 77
8.1.2 Secondary study parameters/endpoints (if applicable) 77
8.1.3 Other study parameters (if applicable) 77
8.2 Randomisation, blinding and treatment allocation 77
8.3 Study procedures (see also appendix 5) 78

8.4 Withdrawal of individual subjects 82
8.4.1 Specific criteria for withdrawal (if applicable) 82
8.5 Replacement of individual subjects after withdrawal 82
8.6 Follow-up of subjects withdrawn from treatment 82
8.7 Premature termination of the study 82
9. SAFETY REPORTING9.1 Section 10 WMO event 83
9.2 AEs, SAEs and SUSARs 83
9.2.1 Adverse events (AEs) 83
9.2.2 Serious adverse events (SAEs) 83
9.2.3 Suspected unexpected serious adverse reactions (SUSARs) 84
9.3 Annual safety report 84
9.4 Follow-up of adverse events 84
9.5 Data Safety Monitoring Board (DSMB) 84
10. STATISTICAL ANALYSIS 85
10.1 Primary study parameter(s) 85
10.2 Secondary study parameter(s) 85
10.3 Other study parameters 87
10.4 Interim analysis (if applicable) 87
11. ETHICAL CONSIDERATIONS 88
11.1 Regulation statement 88
11.2 Recruitment and consent 88
11.3 Objection by minors or incapacitated subjects (if applicable) 88
11.4 Benefits and risks assessment, group relatedness 88
11.5 Compensation for injury 88
11.6 Incentives (if applicable) 89
12. ADMINISTRATIVE ASPECTS, MONITORING AND PUBLICATION 90
12.1 Handling and storage of data and documents 90
12.2 Monitoring and Quality Assurance 90
12.3 Amendments 90
12.4 Annual progress report 90
12.5 End of study report 90
12.6 Public disclosure and publication policy 91
13. STRUCTURED RISK ANALYSIS 92
13.1 Potential issues of concern 92
13.2 Synthesis 92
14. REFERENCES 93
Appendix 1: 97
Appendix 2 98
Appendix 3: 102
Appendix 4 103
Appendix 5: 104

Study protocol

LIST OF ABBREVIATIONS AND RELEVANT DEFINITIONS

ABR	ABR form, General Assessment and Registration form, is the application form that is required for submission to the accredited Ethics Committee (In Dutch, ABR = Algemene Beoordeling en Registratie)
AE	Adverse Event
AR	Adverse Reaction
CA	Competent Authority
CCMO	Central Committee on Research Involving Human Subjects; in Dutch: Centrale Commissie Mensgebonden Onderzoek
CV	Curriculum Vitae
DSMB	Data Safety Monitoring Board
EU	European Union
EudraCT	European drug regulatory affairs Clinical Trials
GCP	Good Clinical Practice
IB	Investigator's Brochure
IC	Informed Consent
IMP	Investigational Medicinal Product
IMPD	Investigational Medicinal Product Dossier
METC	Medical research ethics committee (MREC); in Dutch: medisch ethische toetsing commissie (METC)
(S)AE	(Serious) Adverse Event
SPC	Summary of Product Characteristics (in Dutch: officiële productinfomatie IB1-tekst)
Sponsor	The sponsor is the party that commissions the organisation or performance of the research, for example a pharmaceutical company, academic hospital, scientific organisation or investigator. A party that provides funding for a study but does not commission it is not regarded as the sponsor, but referred to as a subsidising party.
SUSAR	Suspected Unexpected Serious Adverse Reaction
Wbp	Personal Data Protection Act (in Dutch: Wet Bescherming Persoonsgevens)
WMO	Medical Research Involving Human Subjects Act (in Dutch: Wet Medisch-wetenschappelijk Onderzoek met Mensen

Study protocol

SUMMARY

Rationale: Moderate to severe pelvic organ prolapse symptoms can be treated with pessary or surgery. Both treatments appear to be effective, but have not been compared directly.

Hypothesis: The strategy of pessary as initial therapy is as effective as direct surgery for moderate to severe POP, but it is associated with lower costs.

Objective: The primary objective is to compare the effectiveness and cost-effectiveness of pessary versus surgery as initial treatment for moderate to severe symptomatic pelvic organ prolapse (POP) in women at two year after initiation of treatment. The secondary objective is the development of a prediction model for failure of pessary use and surgery within 2 years.

Study design: Cohort study with embedded randomized controlled trial.

Study population: Treatment naïve women with POP who present with moderate to severe symptoms.

Intervention (if applicable): Pessary therapy or vaginal POP surgery.

Main study parameters/endpoints:

Primary outcome: Global impression of improvement of POP symptoms at 24 months measured with PGI-I

Secondary outcomes:

- Changes in symptom bother and disease-specific quality of life at 12 and 24 months follow-up
- Changes of sexual function at 12 and 24 months follow-up
- Changes in general quality of life at 3, 6, 12 and 24 months of follow up
- Adverse events/complications related to both treatment strategies during the study period
- Development of prediction model to identify factors for failing of pessary and surgery.
- Costs-effectiveness analyses

Nature and extent of the burden and risks associated with participation, benefit and group relatedness: Both treatment arms are routine treatments in the Netherlands. Patients in the RCT can have the risks of surgery instead of the risks from pessary therapy.

63

Study protocol

1. INTRODUCTION AND RATIONALE

Problem definition

Female pelvic organ prolapse (POP) is a common problem in women that negatively affects quality of life. The estimated prevalence of symptomatic POP among women between 45-85 years of age is 8.3 - 11% [1,2]. It is current practice in the Netherlands that the general practitioner (GP) treats the majority of women with POP symptoms. Women with moderate to severe POP symptoms are often referred to a gynecologist for treatment. This study focuses at the subgroup of moderate to severe POP.

Known effective treatment options for moderate to severe POP are pessary or surgery. A pessary has proven its effectiveness in the treatment of symptomatic POP, mainly in cystocele and uterine descent. However, studies are mainly observational in nature and inherently subject to selection and indication bias [3]. In literature, outcomes of pessary therapy are mainly recorded in terms of (dis-) continuation of therapy and to a much lesser extent in terms of symptom relief. The pessary continuation rate is 60% [3]. This is confirmed by a Dutch pilot study in 65 women that showed a satisfaction with pessary in 57% of womenand an operation rate of 43% at 12 months follow up [4]. In this study, 80% of women who continued pessary therapy reported much to very much improvement of their POP symptomsat 1 year follow up [4]. Reasons of discontinuation are pressure ulcer, vaginal discharge, discomfort or loss of fitting. These complications are reported to occur in up to 53% of women [5]. Half of them will decide to stop using pessary, but it is unclear which characteristics predict this outcome. Check-up of pessary therapy can be performed by either a general practitioner (GP), gynecologist or by self-management. According to a recent survey 50% percent of gynecologists involved in urogynaecology always offer self- management 40% on indication, and 10% never. Pessary therapy is inexpensive and costs are mainly related to doctor visits and treatment of side effects. In case of self-management costs might even be lower,

Surgery for POP results in much to very much improvement of symptoms in 80% of women and improvement of quality of life [6-9]. An anterior colporraphia is considered the standard procedure for a cystocele, as is the posterior colporraphia for a rectocele. For uterinedescent uterus sparing techniques, like sacrospinous hysteropexy (SH) and modified Manchester-Fothergill procedure, or vaginal hysterectomy can be performed [10-12]]. Complications of POP surgery are temporary urinary retention, temporary buttock pain in case of sacrospinous hysteropexy, urinary tract infection, hematoma or dyspareunia [11]. These complications seldom lead to persistent morbidity. The most

common "complication" is the recurrence of symptomatic POP or de novo stress- incontinence that may lead to additional surgery, pessary therapy, or pelvic floor physiotherapy. As part of a RCT, comparing mesh with fascia plication, we found that
11% of women needed additional surgery after anterior colporraphia at 24 months follow up [7,9]. As in pessary therapy, the characteristics that predict successful or unsuccessfulsurgical therapy are largely unknown.

The decision which treatment option to choose depends on both patient and doctor's preferences. In our pilot survey 70% of gynecologists informed their patients about the possibility of pessary therapy, but it is unknown how many women actually received a pessary. A recent Dutch study showed that 48% of treatment-naive women preferred surgery, 36% a pessary and 16% had no preference [28]. It is therefore reasonable to assume that at least 50% of treatment naïve women with moderate to severe prolapse symptoms will have surgery as primary treatment.

Although clinical efficacy appears to favor surgery [3], the large variation in study design, outcome measurements and loss to follow up makes any comment on the best treatment option speculative. This is recognized in two recent reviews on the subject that both urge the need for randomized trials comparing surgery and pessary for POP [13,14]. Efficacy can be expressed in terms of clinical outcome but also in terms of cost-effectiveness. It is obvious that surgery (especially hospital costs) is much more expensive than pessary therapy, butthe cost-effectiveness of the surgical or pessary strategy has never been assessed. Based on current cohort and case-control studies we hypothesize that a strategy of initial pessary therapy for moderate to severe POP, is more cost-effective than surgery.
We propose to perform a randomized controlled trial to generate evidence for the optimal and most cost-effective primary treatment for moderate to severe POP, including a better a priori patient selection for treatment by identifying factors of failure for pessary therapy or surgery.

Relevance

At present a national multidisciplinary guideline on the diagnosis and treatment of POP is completed. The guideline identifies the lack of evidence with respect to the best treatment option for moderate to severe prolapse, a conclusion that is confirmed by the 2013 Cochrane Collaboration review [13]. In this evidence "vacuum" both doctors and patient preferences rule, but unfortunately these are not supported by facts. If we look at the available data the following

calculation emerges.

About 60% of women who start pessary therapy in the specialist care setting will continue using it at one year [4,15]. Eighty percent of them will report much to very much improvement, resulting in an overall 48% much to very much improvement. The majority ofthe 40% of women who are not satisfied with pessary therapy will request or are offered additional surgery. After surgery 80% of women report much to very much improvement ofPOP symptoms [6]. Combining these percentages, women who originally started withpessary therapy may also expect 80% (48% after initial pessary treatment + 32% afteradditional surgery =) much to very much improvement. Based on these estimates it isexpected that the outcome of both treatment strategies will eventually result in a globalimprovement of symptoms in 80% of women. With equal clinical outcomes of both strategiesthe costs needed to obtain these outcomes become crucial. With the exception of a cost calculation based on a Markov model, no direct cost-effectiveness studies on the use ofpessary or surgery for POP have been performed [16]. The relevance of this project, with the high prevalence of POP worldwide, associated costs and insufficient evidence, is high. Wehave searched the www.clinicaltrials.gov database (3th March 2014) on similar studies(comparing pessary with surgery) but none were found.

However, if we were to prove that pessary therapy is more cost-effective then surgical treatment, this does not imply that a trial of pessary should always be undertaken. There is also insufficient evidence on which patient characteristics are associated with failure of pessary treatment or surgery (systematic review). The knowledge on how to predict which women will have a very low chance of success with pessary therapy can further improve effective treatment strategy management. This will contribute to treatment efficacy. This is not only very relevant for the hospital specialist care setting, but this knowledge can also be implemented in general practitioner practice units.

There is very limited evidence on the optimal management strategy for pessary cleaning, both in time interval as well as in who should perform the cleaning. Our study is unique and therefore relevant since self-management is advocated in the study setting. This will not only allow it to obtain data in a standardized way, but also involves the woman in her own management. This involvement is strongly advocated by two major gynecologic patient organizations (‘Patienten Gynaecologie Nederland’ and the ‘Stichting Bekkenbodem Patienten’). These two organizations, as well as the Dutch urogynaecological consortium have identified this study to be highly relevant.

In line with the report "Medisch Specialistische zorg 20/20" we are heading towards integral health care in which the general physician and medical specialist will work more closely together, using the same treatment protocol for various illnesses. The information and conclusions of this trial will add level I scientific evidence to such an integral protocol and guideline for women with symptomatic POP. This will aid in a better patient selection that will need referral to the specialist. The data on patient's self-management of pessary treatment will supply information for patient instructions, which are relevant for information leaflets on the subject.

2. OBJECTIVES

The aim of this multicenter pragmatic cohort study with embedded randomized controlled non-inferiority trial comparing pessary therapy versus surgery is twofold:

1. To prospectively compare the effectiveness and cost-effectiveness of pessary therapy or surgery as primary treatment of moderate to severe symptomatic cystocele, uterine descent and/or rectocele in women at two year after initiation of treatment, in randomized trial embedded in a preference cohort.
2. To compare the effectiveness between the cohort and randomized trial.
3. To develop a prediction model for failure of pessary use and surgery within the first years.

3. STUDY DESIGN

Multicenter pragmatic cohort study with an embedded randomized controlled non-inferiority trial comparing pessary [CE 0086] therapy and surgery including an economic evaluation. The follow up will be 24 months.

A short (30 minutes) trial of pessary fitting is part of our protocol. This ensures that only women who fit both treatment options enter the randomization procedure. The trial is short and only aims at fitting, not symptom relief. Women with an unsuccessful pessary fitting will be followed in the cohort fitting failure. In case the woman is willing to participate but actively opts for one of both treatments, she will be followed in a cohort.

See also appendix 1 and 5.

4. STUDY POPULATION

4.1 Population (base)

All women with a symptomatic POP will be included.

4.2 Inclusion criteria

In order to be eligible to participate in this study, a subject must meet all of the following criteria:

1. Women with a prolapse stage 2 or more.
2. Women with moderate to severe POP symptoms. Moderate to severe POP symptoms is defined as a prolapse domain score > 33 on the validated Dutch version of the Pelvic Floor Distress Inventory (PFDI-20) [8, 23, 24].
3. For the RCT: Women who have had a successful pessary fitting procedure.
4. Written informed consent.

4.3 Exclusion criteria

A potential subject who meets any of the following criteria will be excluded from participation in this study:

1. Prior urogynaecological (prolapse or incontinence) surgery
2. Probability of future childbearing
3. Insufficient knowledge of the Dutch language
4. Co-morbidity causing increased surgical risks at the discretion of the surgeon
5. Major psychiatric illness
6. Prior pessary use

4.4 Sample size calculation

With 198 women per group, we will have 80% power to reject the null hypothesis that pessary therapy is inferior to surgery, with a 1-sided alpha of 0.05, a non-inferiority margin of 10% and the proportion in the standard group is 80% (NQueryAdvisor). Accounting for 10% loss to follow-up we plan to randomize 436 patients.

The sample size calculation for prediction models is based on the number of failures of pessary or surgical therapy. For each potential predictor in the model we need 10-15 failures. Our pessary group sample size is 198 women. An estimated 40% (80 women) will cross over to surgery and can be regarded as failures. Our sample size is therefore sufficient

to develop the prediction model for failure of pessary therapy for 6 to 8 items. In the surgery group 20% of women will not be satisfied with the result of treatment. With the same sample size of 198 women, the 40 women who are dissatisfied allow us to study up to 4 potential predictive factors.

In the cohort we include all patients who are willing to collaborate on this research but have a preference for one of both therapies. We now assume that 70% of the eligible patients object participation in the RCT, and that 90% of them is nevertheless willing to participate in the cohort.

5. TREATMENT OF SUBJECTS

5.1 Investigational product/treatment

Pessary [CE 0086] therapy and surgery both are options for the treatment of a symptomatic POP. Ten large urogynecological units (university hospitals or teaching hospitals) that have worked together in previous consortium studies will participate in this multicenter trial. All participating gynaecologists have fitted at least 100 pessaries and performed more than 100 surgical POP procedures prior to the start of this study.

All pessaries are made of modern silicon material. All types of pessaries, both supportive and occlusive/space filling are allowed according to the judgment of the gynaecologist. A recent randomized trial comparing supportive (ring) and occlusive (Gelhorn) showed no differences [17]. After placing the pessary, all women will receive verbal and written instructions on the self-management of pessary therapy.

The first pessary follow up visit will always be performed by the gynaecologist. In case of self-management the frequency of cleaning is left to her personal judgment, but may not exceed 4 months. If self-management is not possible, women will be seen at 4 months intervals for pessary cleaning and vaginal inspection, preferable by their GP. In case of vaginal atrophy topical estrogens will be advised according to pharmaceutical guidelines. The diagnosis of atrophy is left to the judgment of the treating physician, since no clear definition for atrophy is available yet [18].

All surgical procedures will be performed according to our national guidelines. In this pragmatic trial the decision which technique to use is left, to the discretion of the gynaecologist, within the limitations below [19]. Cystocele repair will consist of conventional anterior colporrhaphy [9]. For uterine descent different techniques are allowed [20]. These techniques can either be uterus sparing (sacrospinous hysteropexy [10], modified Manchester-Fothergill procedure [12] or a abdominal sacrocolpopexy [9]) or a vaginal hysterectomy. Recent studies showed similar effectiveness on both anatomical and functional outcomes for these different techniques [10, 12, 21]. A coexistent stage 2 rectocele repair will be a conventional colporrhaphia posterior. All procedures are performed under general or spinal anesthesia and under antibiotics and thrombosis prophylaxis according to local protocols.

5.2 Use of co-intervention (if applicable)

Not applicable.

5.3 Escape medication (if applicable)

Not applicable.

6. INVESTIGATIONAL PRODUCT

6.1 Name and description of investigational product(s)

6.2 Summary of findings from non-clinical studies

Not applicable.

6.3 Summary of findings from clinical studies

Three systematic reviews of the literature were performed by four members of our Dutch urogynaecology consortium (details in appendix 2-4) that concluded:

1. Systematic review on the (cost)effectiveness of pessary use compared to surgery

There are a very limited number of comparative studies on the efficacy of surgery or pessary use for POP. The differences in study population, inclusion criteria, follow-up period, large numbers of loss to follow-up, different outcome measures makes interpretation difficult if not impossible. The two studies that presented data on functional outcome in terms of prolapsed symptom reduction were favorable for surgery (appendix 2).

2. Systematic review of factors influencing pessary fitting and continuation

A systematic review was performed to identify the satisfactory pessary fitting rate and the continuation rate of pessary use. The factors influencing these rates as well as the cross over to prolapse surgery were identified from previous studies (appendix 3).

Summarizing the results show that an estimated 75% of women will have a successful fitting and 59% will continue pessary use at variable follow-up between 3 months and 5 years. In these 18 studies, 8 factors have been tested more than 4 times as prognostic factor of successful pessary use: Stress urinary incontinence was found associated with discontinuation of pessary in 5 out of 7 studies. In 7 out of 10 studies previous prolapse surgery or hysterectomy was associated with less continuation of pessary use. Higher age was related to continuation of pessary use in 3 out of 6 studies, whereas no correlation was found in the other studies. In 1 out of 4 studies sexual activity was related to longer pessary use, whereas in 1 out of 4 related to the choice for surgery. In the two other studies no correlation was found. In one study where the prolapse in a specific vaginal compartment was related to outcome, nor cystocele was related to longer pessary use.

Parity en menopausal status and hormonal replacement were mostly not related to continued pessary use.

3. Review of factors influencing failure of POP surgery.

A systematic review of factors influencing failure of POP surgery was performed concerning recurrence after surgery (surgery failure). There were 1 case control study, 3 prospective studies and 6 retrospective studies. There were 2298 women included in the studies. Forty-four (44) potential risk factors have been studied, of which 12 risk factors have at least once been identified as statistically significant risk factors in a multivariate logistic regression analysis (appendix 4).

6.4 Summary of known and potential risks and benefits

The present study carries low risks for the participant. Pessary [CE 0086] or surgery is standard care for symptomatic pelvic organ prolapse. Known risks for surgery are blood loss, risk of infection, dyspareunia, urine incontinence or a recurrence of a symptomatic pelvic organ prolapse.

The benefit of the study lies in a better understanding of satisfaction and cost effectiveness.

6.5 Description and justification of route of administration and dosage

Not applicable

6.6 Dosages, dosage modifications and method of administration

Not applicable

6.7 Preparation and labelling of Investigational Medicinal Product

Not applicable

6.8 Drug accountability

Not applicable

7. NON-INVESTIGATIONAL PRODUCT

Not applicable.

7.1 Name and description of non-investigational product(s)

Not applicable.

7.2 Summary of findings from non-clinical studies

Not applicable.

7.3 Summary of findings from clinical studies

Not applicable.

7.4 Summary of known and potential risks and benefits

Not applicable.

7.5 Description and justification of route of administration and dosage

Not applicable.

7.6 Dosages, dosage modifications and method of administration

Not applicable.

7.7 Preparation and labelling of Non Investigational Medicinal Product

Not applicable.

7.8 Drug accountability

Not applicable.

Ben ÂJ, *et al. BMJ Open* 2024; 14:e075016. doi: 10.1136/bmjopen-2023-075016

8. METHODS

8.1 Study parameters/endpoints

8.1.1 Main study parameter/endpoint

The primary outcome of this study is the percentage of women with much or very much improvement of POP symptoms at 2 years follow-up, as measured with the Patient Global Impression of Improvement (PGI-I) scale [22].
PGI-I is a 7-point Likert scale, with scores ranging from very much worse to very much improved. Success is defined as ‘much or very much’ improvement.

8.1.2 Secondary study parameters/endpoints (if applicable)

1. Changes in symptom bother and quality of life at 12 and 24 months follow up.
2. Changes in sexual function at 12 and 24 months follow up.
3. Changes in general quality of life at 3, 6, 12 and 24 months.
4. Adverse events/complications related to both treatment strategies.
5. Development of prediction model to identify fail factors for pessary and surgery
6. Cost-effectiveness

8.1.3 Other study parameters (if applicable)

Baseline characteristics: Age; ethnicity; allergies; smoking; obstetric history including number and mode of deliveries; menopausal status; hormone use; use of medication; height; weight; co-morbidity (diabetes mellitus, COPD); history of gynaecological operations; family history of prolapse; duration of complaints;.
Physical examination: time, POP-Q, atrophy, vulvar deviations, stress test.
Brand pessary, type of surgery.

8.2 Randomisation, blinding and treatment allocation

After written informed consent is obtained, and inclusion and exclusion criteria are assessed, women will be randomly allocated in a 1:1 ratio to either treatment with a pessary or surgical treatment. Randomization will be done web based using ALEA, the software for randomization in clinical trials currently used by most studies in the Dutch consortium for studies on women’s health and reproduction studies. The randomization sequence will be computer generated using variable blocks of two and four, stratified for centre.
After entering the woman’s initials and confirming inclusion criteria on the website, a

unique number for randomization will be generated and the allocation code will be disclosed. This unique number cannot be deleted afterwards. This study will be open label because the nature of the intervention meant that masking to the intervention was not possible. Women who attend the cohort fitting failure will also be registered in ALEA.

In case the woman is willing to participate but actively opts for one of both treatments, she will also be registered in ALEA.

All groups will have the same data collection and follow up as displayed in appendix 5. We expect differences in the study parameters between RCT and cohort, in effectivity, satisfaction and cost effectivity.

8.3 Study procedures (see also appendix 5)

This study will be performed within the Dutch Urogynaecology Research Consortium, a subdivision of the Dutch Consortium for studies on women's health. Infrastructure (research nurses for counseling and data-monitoring, the use of web-based data entry), expertise on methodology and cost-effectiveness is shared.

1. Symptom bother and disease-specific quality of life are measured with the Pelvic Floor Distress Inventory (PFDI-20) and Pelvic Floor Impact Questionnaire (PFIQ) [23,24]. The validated version of the Dutch PFDI consists of five domains: pelvic organ prolapse, urinary incontinence, overactive bladder, pain, and obstructive micturition. The PFIQ measures the impact of urogenital symptoms on quality of life and consist of five domains: physical functioning, mobility, emotional health, embarrassment and social functioning.

2. Sexual function is measured with the PISQ-R. It is an international disease-specific questionnaire that measures sexual functioning in sexually active and inactive participants [26].

3. Generic quality of life is measured with the EQ-5D and a questionnaire "doktersbezoek".

4. The adverse events of surgery recorded will consist of; direct peri-and postoperative complications (bleeding, pain and infection); interventions for complications; recurrent prolapse; de novo stress urinary incontinence. The adverse events of pessary recorded will consist of; discharge; pain; discomfort; bleeding; involuntary loss of pessary; de novo stress urinary incontinence.

5. The development of a prediction model is separately described in paragraph "data analyses".

6. The economic evaluation is described below.

ANTICIPATED COST-EFFECTIVENESS/BUDGET IMPACT

Cost differences between the two strategies are mainly the result from differences in costs associated with the initial intervention. Cost of a POP procedure is estimated at 4000 euros direct medical costs, and 4000 euros associated with lost productivity (indirect costs) if a societal perspective is used. The direct cost of pessary use is estimated at 200 euros, including costs for the pessary itself (50 euros) and consultations in the first year (150 euros). The estimated cost differences between the two strategies depend on the extent that women are (and remain) satisfied with the initial procedure (surgical or pessary): in case of dissatisfaction with the procedure, additional costs are generated by a subsequent intervention ((re-)operation, pessary, or pelvic physiotherapy). The flowchart (see appendix 1) illustrates the expected outcomes for each strategy. Based on the assumptions reflected in this flowchart, combined with approximate estimates for unit costs for POP surgery, pessary, GP and specialist visits, the anticipated impact on the annualhealth care budget as well as societal costs were estimated.

At present, the primary therapy for women presenting with moderate to severe POP is either surgery or pessary. The exact ratio is unknown, but is probably 50/50. If 50% women would receive primary surgery the current medical costs amount to 34 million Euros. If all women would start with pessary therapy, these costs would be 20 million euros, and the potential budget impact would be 14 million Euros/year. Asit is not realistic that all women will start with pessary if this strategy proves to be successful, at 85% implementation of the pessary strategy, the annual budgetimpact will be around 10 million euros. The economic impact to society (including indirect (productivity) costs) will be 28 million euros and 20 million euros, at 100% and 85% implementation, respectively.

Sensitivity analyses showed, that these “base case” results are affected by estimated unit costs for POP surgery (direct and indirect costs) and the satisfaction rate for pessary, relative to surgery, but even the most conservative assumptions would lead to major cost savings for the health care budget (5 million euros) and society (15 million euros).

ECONOMIC EVALUATION

The economic evaluation will be based on the randomized trial. Considering the non-inferiority design of the study, we will not be able to rule out a small but acceptable

difference in favor of POP surgery. Consequently, the economic evaluation will be setup as a cost-effectiveness analysis, where cost-effectiveness will be expressed as costs per improvement outcome (much or very much improvement on the Patient Global Impression of Improvement (PGI-I)), and the incremental cost-effectiveness ratio as costs saved per additional case of unsatisfactory outcome. We will also perform a cost-effectiveness analysis using QALYs as health outcome, to express the difference between the two strategies in terms of costs (saved) per QALY (lost).

The economic evaluation will therefore encompass a cost-effectiveness analysis (CEA), a cost-utility analysis (CUA) as well as a budget impact analysis (BIA) from a health care budget and a societal perspective, with a time horizon between randomization and 2 years follow up. The primary outcome in the cost-effectiveness analysis will be costs per satisfactory outcome (primary clinical outcome), and the incremental cost-effectiveness ratio will reflect the costs saved per additional case of unsatisfactory outcome. As we hypothesize that pessary as a primary strategy in these patients does not result in more unsatisfactory outcomes, increased use of pessary will result in a decrease in the number of POP surgeries, and associated costs of hospital stay, recovery and (from a societal perspective) productivity loss (non-inferior strategy at lower costs).

Based on data actually observed in the trial, total costs associated with both surgery and pessary as a primary strategy will be estimated. Total costs can be divided into direct medical costs, non-medical costs and indirect costs. Direct medical costs are generated by utilization of primary or secondary health care services (including POP surgery, hospital stay, diagnostic procedures, medication). Non-medical costs are generated by travel expenses, and informal care; and indirect costs result from lost productivity due to absence from work or lost opportunity for non-paid activities. Non-medical and indirect costs are only included in the analysis from a societal perspective.

Resource utilization will be documented in the clinical report form (CRF) and complementary patient questionnaires, based on the Medical Consumption Questionnaire (MCQ) and Productivity Costs Questionnaire (PCQ) [29,30]. In patients for whom complete follow-up is not available, cost and quality-of-life datawill be extrapolated using multiple imputations. Unit costs will be based on Dutch guideline prices (for primary and secondary health services, informal care and lost productivity),

and market prices (for medication)[31,32]

Similarly, the incremental costs per QALY gained will be estimated over a period of two years. Health state utilities to estimate QALYs will be derived from an EQ-5D measurement at discharge, as well as at follow-up assessments. Utility values for EQ-5D scores will be based on UK-estimates (Dolan, 1997). Utility scores will be linearly interpolated, assuming constant increase/decrease between subsequent assessments.

Robustness for sampling uncertainty as well as uncertainty associated with cost estimates and assumptions will be assessed in sensitivity analyses, including: Dutch health states (Lamers, 2005) instead of the UK based model in the main analyses; and varying unit costs for pertinent volumes of health care utilization (e.g. costs of POP surgery, pessary use, productivity costs).
The incremental costs and effects will be depicted in a cost effectiveness plane and cost-effectiveness acceptability curves providing information directly interpretable as the probability of one intervention being cost-effective compared to the alternative given a ceiling ratio that policy makers are willing to invest.

BIA
In a budget impact analysis, study results will be extrapolated to the national level to estimate the total impact on the health care budget per annum for the Netherlands in terms of cost reduction and health outcomes (satisfactory outcomes as well as QALYs). As economic consequences of the intervention are expected to spanmultiple years, this accumulation of cost (savings) will be reflected in the budget impact analyses.

The Budget Impact Analysis will be executed according to the international ISPOR guidelines [33]. This framework for creating a budget impact model includes formalized guidance about the acquisition and use of data in order to make budget projections. In addition to the societal perspective, the BIA will therefore be also report economic consequences from the perspective of the Dutch budgetary health care framework (BKZ). If the probability of an unsatisfactory outcome exceeds the non-inferiority limit, recommending pessary as primary treatment for all women isnot feasible, and an economic evaluation/budget impact analysis is not sensible. To estimate costs, we will follow the Handleiding Kosten onderzoek CVZ 2010.

8.4 Withdrawal of individual subjects

Subjects can leave the study at any time for any reason if they wish to do so without any consequences. The investigator can decide to withdraw a subject from the study for urgent medical reasons.

8.4.1 Specific criteria for withdrawal (if applicable)

Not applicable.

8.5 Replacement of individual subjects after withdrawal

We will not replace patients who withdrew informed consent. We will replace patients that are randomized by mistake, for example because of technical errors with online randomization.

8.6 Follow-up of subjects withdrawn from treatment

Patients withdrawn from the intervention but not from informed consent will be followed up.

8.7 Premature termination of the study

This study includes standard care, therefore it is very unlikely that unexpected complications will occur. Therefore premature termination is not applicable.

9. SAFETY REPORTING

9.1

Temporary halt for reasons of subject safety (section 9.1, CCMO Template Research Protocol)

In accordance to section 10, subsection 4, of the WMO, the sponsor will suspend the study if there is sufficient ground that continuation of the study will jeopardise subject health or safety. The sponsor will notify the accredited METC with undue delay of a temporary halt including the reason for such an action. The study will be suspended pending further review by the accredited METC. The investigator will take care that all subjects are kept informed.

Temporary halt and (prematurely) end of study report (section 12.5, CCMO Template Research Protocol)

The sponsor will notify the METC immediately of a temporary halt of the study, including the reason of such an action.

9.2 AEs, SAEs and SUSARs

9.2.1 Adverse events (AEs)

Adverse events are defined as any undesirable experience occurring to a subject during the study, whether or not considered related to the study. All adverse events reported spontaneously by the subject or observed by the investigator or his staff will be recorded. During visits complaints will be questioned systematically.

9.2.2 Serious adverse events (SAEs)

A serious adverse event is any untoward medical occurrence or effect that at any dose:

- results in death;
- is life threatening (at the time of the event);
- requires hospitalisation or prolongation of existing inpatients' hospitalisation (>4 days);
- results in persistent or significant disability or incapacity;
- is a congenital anomaly or birth defect;
- Any other important medical event that may not result in death, be life threatening, or require hospitalization, may be considered a serious adverse experience when,

based upon appropriate medical judgement, the event may jeopardize the subject or may require an intervention to prevent one of the outcomes listed above.

SAEs have to be reported when its occurrence appears in two days after the study operations. The investigators in participating centres should inform the coordinating investigator as soon as possible but at least the next working day.

The sponsor will report the SAEs through the web portal *ToetsingOnline* to the accredited METC that approved the protocol, within 15 days after the sponsor has first knowledge of the serious adverse events.

SAEs that result in death or are life threatening should be reported expedited. The expedited reporting will occur not later than 7 days after the responsible investigator has first knowledge of the adverse event. This is for a preliminary report with another 8 days for completion of the report.

9.2.3 Suspected unexpected serious adverse reactions (SUSARs)

Not applicable.

9.3 Annual safety report

The annual safety report will be combined with the annual progress report (see chapter 12.4).

9.4 Follow-up of adverse events

All AEs will be followed until they have abated, or until a stable situation has been reached. Depending on the event, follow up may require additional tests or medical procedures as indicated, and/or referral to the general physician or a medical specialist.

SAEs need to be reported till end of study within the Netherlands, as defined in the protocol

9.5 Data Safety Monitoring Board (DSMB)

Since both techniques are standard practice, and no major unexpected complications are foreseen, no interim analysis is planned. A Data Safety and Monitoring Board will not be installed, as both procedures are regularly used and acceptable options in current clinical practice.

10. STATISTICAL ANALYSIS

The results of the study will be reported according to the CONSORT statement.

The main outcomes will be analysed and presented according the intention-to-treat principle. Since in this pragmatic trial we expect that in the pessary strategy group 40% of women will cross over to surgery an additional per protocol analyses is foreseen. This will provide more insight in the effect of surgery after pessary therapy as compared to surgery or pessary therapy alone.

We plan a subgroup analysis for the location of the prolapse: anterior prolapse versus posterior prolapse.

The cohort with patients treated according their preference will be analysed separately from the randomized trial, and presented in the same manuscript, which will provide insight into the generalizability of the results.

10.1 Primary study parameter(s)

The primary outcome, success (much or very much improvement) or no success (a little better, no change, a little worse, much worse or very much worse) on the PGI-I will be expressed in percentage point differences. Differences between the percentages will be tested using a chi-square test. A p-value <0.05 will be considered statistically significant.

For the cohort study results will be presented separately, and the same analyses will be done. Differences between the trial arm and the cohort arm will be tested using the chi-square test, to determine the generalizability of the results.

10.2 Secondary study parameter(s)

The PFDI, PFIQ and PISQ-r are all interval scales. Differences between baseline and 12 and 24 months follow up will be assessed using an independent t-test when normality can be assumed, or by non-parametric tests when the data are not normally distributed. Effect sizes will be calculated to estimate the magnitude of changes.

Differences in EQ5-D scores and “ziekteverzuim” between baseline and at 3, 6, 12 and 24 months will be assessed using t-test and further incorporated in the cost-effectiveness analyses.

Imputation statistics will be used or missing data.

(Serious) adverse events will be categorized and chi-square statistics, with calculation of relative risks when appropriate, will be applied in analyses.

Prediction model

A prediction model that uses predefined variables, as potential predictors of failure of pessary therapy, will be developed using multivariable regression analysis.Missing data will be imputed.

Predictors for failure derived from literature are a large genital hiatus (gh > 4 cm), women being sexually active, age > 65 years, prolapse POP-Q stage 3, previous hysterectomy. If applicable, a prediction rule for the chance of failure of pessary therapy will be constructed, which could be presented as a normogram which could be used to determine the chance of failure on pessary therapy.

Internal validity will be assessed using bootstrapping techniques; shrinkage will be applied to the parameter estimates. Model performance will be assessed with discriminative capacity and calibration. Calibration will be assessed by comparing the mean predicted probability that patients failed on pessary therapy with the mean observed probability that patients failed on pessary therapy. To do so, the total cohort will be split into ten groups based on the deciles of the predicted probability. Per group the mean predicted probability will be calculated as well as the mean observed predicted probability. Discriminative capacity of the model will be assessed with receiver operation characteristics (ROC) analysis and the area under the ROC curve (AUC).

We will also look at factors that could explain failure of surgery. Our systematic review on POP and recurrent POP after surgery showed that 6 preoperative items, eg. POP stage, age, family history, preoperative incontinence, previous POP or incontinence surgery, previous hysterectomy seems to be predictive for recurrence.

Women with previous POP or incontinence surgery are excluded from our study, leaving 4 predefined potential predictive factors. After the 2 year follow-up has been performed, we will reconsider which factors to include in a prognostic model, based on the current literature. We will select predictors from literature with the highest predictive value, where about 1 predictor could be selected for each 10 surgery failures.

Using interaction terms the effect of a differential effect in women with a higher age (>median) or a lower age (<=median), a higher (>25) or lower BMI(<=25) will be assessed for both pessary as well as surgery failures prediction.

10.3 Other study parameters

Not applicable.

10.4 Interim analysis (if applicable)

Not applicable, because of the non-inferiority design with low risk and the possibility of cross over.

11. ETHICAL CONSIDERATIONS

11.1 Regulation statement

This study will be conducted according to the principles of the Declaration of Helsinki (version 10, October 2013) and in accordance with the Medical Research InvolvingHuman Subjects Act (WMO) and other guidelines, regulations and Acts.

11.2 Recruitment and consent

Women with symptomatic pelvic organ prolapse who attend the outpatient clinic will be informed about the study by the gynaecologist or nurse. After checking the in- and exclusion criteria the women will receive verbal and written information about the study. If the woman is willing to participate she is asked to sign the informed consent. All women will undergo the pessary fitting test which is part of the standard evaluation. All women will be contacted at a minimum interval of 1 week. Those women who failed the initial fitting will be offered surgery and attend the cohort fitting failure, the women with a succesfull initial fitting will be asked to enroll in the RCT. In case the woman is willing to participate but actively opts for one of both treatments, she can attend the cohort. Her motivation is requested. In case the women is not willing to participate, she will be registred as “refuser”.

11.3 Objection by minors or incapacitated subjects (if applicable)

Not applicable.

11.4 Benefits and risks assessment, group relatedness

The present study carries no risks for the participant. Pessary or surgery are standard care for symptomatic pelvic organ prolapse. The benefit of the study lies in a better understanding of satisfaction and cost effectiveness.

11.5 Compensation for injury

The sponsor/investigator has a liability insurance which is in accordance with article 7, subsection 9 of the WMO.

The sponsor (also) has an insurance which is in accordance with the legal requirements in the Netherlands (Article 7 WMO and the Measure regarding Compulsory Insurance for Clinical Research in Humans of 23th June 2003). This insurance provides cover for

damage to research subjects through injury or death caused by the study.

1. € 450.000,-- (i.e. four hundred and fifty thousand Euro) for death or injury for each subject who participates in the Research;
2. € 3.500.000,-- (i.e. three million five hundred thousand Euro) for death or injury for all subjects who participate in the Research;
3. € 5.000.000,-- (i.e. five million Euro) for the total damage incurred by the organisation for all damage disclosed by scientific research for the Sponsor as 'verrichter' in the meaning of said Act in each year of insurance coverage.

The insurance applies to the damage that becomes apparent during the study or within 4 years after the end of the study.

11.6 Incentives (if applicable)

Not applicable.

12. ADMINISTRATIVE ASPECTS, MONITORING AND PUBLICATION

12.1 Handling and storage of data and documents

All data will be stored and will be coded. Only the researchers will be able to link patient ID and research code. The handling of personal data complies with the Dutch Personal Data Protection Act. After cessation of the study, patient material will be stored for a maximum of 15 years.

The case report forms and questionnaires will be filled in online. The head investigator will be able to check all the completed forms and questionnaires.

12.2 Monitoring and Quality Assurance

The monitoring will be coordinated by the Dutch Consortium and will be executed by a qualified intern monitor. This person is not involved in design and output of this research. The frequency of checking will be every year. The monitoring plan is discussed in section K of the METC dossier.

12.3 Amendments

All substantial amendments will be notified to the METC.

Non-substantial amendments will not be notified to the accredited METC, but will be recorded and filed by the sponsor.

12.4 Annual progress report

The sponsor/investigator will submit a summary of the progress of the trial to the accredited METC once a year. Information will be provided on the date of inclusion of the first subject, numbers of subjects included and numbers of subjects that have completed the trial, serious adverse events/ serious adverse reactions, other problems, and amendments.

12.5 End of study report

The investigator will notify the accredited METC of the end of the study within a period of 8 weeks. The end of the study is defined as the last patient's last visit.

In case the study is ended prematurely, the investigator will notify the accredited METC within 15 days, including the reasons for the premature termination.

Within one year after the end of the study, the investigator/sponsor will submit a final study report with the results of the study, including any publications/abstracts of the study,to the accredited METC.

12.6 Public disclosure and publication policy

The research findings will be published in peer reviewed journals.

13. STRUCTURED RISK ANALYSIS

Not applicable because this study is a low risk study concerning standard care.

13.1 Potential issues of concern

Not applicable.

13.2 Synthesis

Not applicable.

Study protocol

14. REFERENCES

1: Slieker-ten Hove MC et al. The prevalence of pelvic organ prolapse symptoms and signs and their relation with bladder and bowel disorders in a general female population. Int Urogynecol J. 2009;20(9):1037-45
2. Tegerstedt G, Maehle-Schmidt M, Nyrén O, Hammarström M. Prevalence of symptomatic pelvic organ prolapse in a Swedish population. Int Urogynecol J Pelvic Floor Dysfunct. 2005 Nov-Dec;16(6):497-503
3: Lamers BH et al. Pessary treatment for pelvic organ prolapse and health-related quality of life: a review. Int Urogynecol J. 2011;22(6):637-44
4: Broekman BM et al. Prospective pilot study in 65 women on pessary treatment as primary treatment, unpublished data
5:Broens-Oostveen WC, Mom RM, Lagro-Janssen ALM. De genitale prolaps: behnadleingen beloop in vier huisartspraktijken. Ned Tijdschr Geneeskd 2004, 148 (29); 1444-1448
6: Withagen M et al. Sacrospinous hysteropexy or modified manchester for the treatment of pelvic organ prolapse? Int J Gynaecol Obstet. 2013; Abstract Supplement
7: Vollebregt A et al. Cost-utility analysis of trocar-guided transobturator mesh versus anterior colporrhaphy for primary cystocele repair: a randomised trial. [in preparation]
8: Barber MD[1], Walters MD, Bump RC. Short forms of two condition-specific quality-of-life questionnaires for women with pelvic floor disorders (PFDI-20 and PFIQ-7). Am J Obstet Gynecol. 2005 Jul;193(1):103-13.
9: Pollard ME, Eilber KS, Anger JT. Abdominal approaches to pelvic prolapse repairs. Curr Opin Urol. 2013;23(4):306-11.
10: Dietz V et al. Vaginal surgery for uterine descent; which options do we have? A review of the literature. Int J Gynaecol Obstet. 2009;20(3):349-56
11: C. Maher, Feiner B, Baessler K, Schmid C. Surgical management of pelvic organprolapse in women. Cochrane 2013
12: de Boer TA, Milani AL, Kluivers KB, Withagen MI, Vierhout ME. The effectiveness of surgical correction of uterine prolapse: cervical amputation with uterosacral ligament plication (modified Manchester) versus vaginal hysterectomy with high uterosacral ligament plication. Int Urogynecol J Pelvic Floor Dysfunct. 2009 Nov;20(11):1313-9
13: Bugge C et al. Pessaries (mechanical devices) for pelvic organ prolapse in women. Cochrane database syst rev, 2013;28;2:CD004010
14: van Geelen JM, Dwyer PL. Where to for pelvic organ prolapse treatment after the FDA pronouncements? A systematic review of the recent literature. Int Urogynecol J. 2013 May;24(5):707-18
15: Abdool Z et al. Prospective evaluation of outcome of vaginal pessaries versus surgery in women with symptomatic pelvic organ prolapse. Int Urogynecol J. 2011;22(3):273-8
16: Hullfish KL, Trowbridge ER, Stukenborg GJ. Treatment strategies for pelvic organ prolapse: a cost-effectiveness analysis. Int Urogynecol J. 2011 May;22(5):507-15
17: Cundiff GW, Amundsen CL, Bent AE, Coates KW, Schaffer JI, Strohbehn K, Handa VL. The PESSRI study: symptom relief outcomes of a randomized crossover trial of the ring and Gellhorn pessaries. Am J Obstet Gynecol. 2007 Apr;196(4):405.e1-8
18: Weber MA, Limpens J, Roovers JPWR. Assessment of vaginal atrophy: a review [submitted BJOG]
19: Cobra Ok-Klapper 2012. DCHG 2012
20: Detollenaere RJ, den Boon J, Kluivers KB, Vierhout ME, van Eijndhoven HW.Surgical management of pelvic organ prolapse and uterine descent in the Netherlands. Int Urogynecol J. 2013 May;24(5):781-8
21.Detollenaere RJ, den Boon J, Vierhout ME, van Eijndhoven HW. Uterus preservingsurgery versus vaginal hysterectomy in treatment of uterine descent: a systematic review]. Ned

Tijdschr Geneeskd. 2011;155(42):A3623
22: Yalcin I, Bump RC. Validation of two global impression questionnaires for incontinence. Am J Obstet Gynecol. 2003 Jul;189(1):98-101

23: Barber MD, Kuchibhatla MN, Pieper CF, Bump RC. Psychometric evaluation of 2 comprehensive condition-specific quality of life instruments for women with pelvic floor disorders. Am J Obstet Gynecol 2001;185(6):1388–1395.

24: Utomo E, Blok BF, Steensma AB, Korfage IJ. Validation of the Pelvic Floor Distress Inventory (PFDI-20) and Pelvic Floor Impact Questionnaire (PFIQ-7) in a Dutch population. Int Urogynecol J. 2014;25(4):531-44.

25: Euroquol—a new facility for the measurement of health-related quality of life. The EuroQol Group. Health Policy. 1990;16:199-208

26: Rogers RG, Rockwood TH, Constantine ML, Thakar R, Kammerer-Doak DN, Pauls RN, Parekh M, Ridgeway B, Jha S, Pitkin J, Reid F, Sutherland SE, Lukacz ES, Domoney C, Sand P, Davila GW, Espuna Pons ME. A new measure of sexual function in women with pelvic floor disorders (PFD): the Pelvic Organ Prolapse/Incontinence Sexual Questionnaire, IUGA-Revised (PISQ-IR). Int Urogynecol J. 2013 Jul;24(7):1091-103

27: Cabana MD, Rand CS, Powe NR, Wu AW, Wilson MH, Abboud PA, Rubin HR. Why don't physicians follow clinical practice guidelines? A framework for improvement. JAMA. 1999 Oct 20;282(15):1458-65

28: Thys SD, Roovers JP, Geomini PM, Bongers MY. Do patients prefer a pessary or surgery as primary treatment for pelvic organ prolapse. Gynecol Obstet Invest. 2012;74(1):6-12

29: www.imta.nl (C. Bouwmans et al. Handleiding iMTA Medical Cost Questionnaire (iMCQ). Rotterdam: iMTA, Erasmus Universiteit Rotterdam, 2013)

30: www.imta.nl (C. Bouwmans et al. Handleiding iMTA Productivity Cost Questionnaire (iPCQ). Rotterdam: iMTA, Erasmus Universiteit, 2013)

31:Hakkaart- van Roijen, S.S. Tan, C.A.M. Bouwmans: Handleiding voor Kostenonderzoek. Methoden en standaard kostprijzen voor economische evaluaties in de gezondheidszorg. CVZ 2010

32: Oostenbrink JB, Koopmanschap MA, Rutten FF. Standardisation of costs: the Dutch Manual for Costing in economic evaluations. Pharmacoeconomics 2002;20(7):443-454

33: Mauskopf JA, Sullivan SD, Annemans L et al. Principles of good practice for budget impact analysis: report of the ISPOR Task Force on good research practices--budget impact analysis. Value Health 2007;10(5):336-347 -> In BIA, klopt niet, nog vervangen door:

References (Cost) effectiveness of pessary use as compared to surgery systematic review

1: Mamik MM, Rogers RG, Qualls CR, Komesu YM. Goal attainment after treatment in patients with symptomatic pelvic organ prolapse. Am J Obstet Gynecol. 2013 Nov;209(5):488.e1-5.

2: Abdool Z, Thakar R, Sultan AH, Oliver RS. Prospective evaluation of outcome of vaginal pessaries versus surgery in women with symptomatic pelvic organ prolapse. Int Urogynecol J. 2011 Mar;22(3):273-8.

3: Lowenstein L, Gamble T, Sanses TV, van Raalte H, Carberry C, Jakus S, Pham T, Nguyen A, Hoskey K, Kenton K; Fellow's Pelvic Research Network. Changes in sexual function after treatment for prolapse are related to the improvement in body image perception. J Sex Med. 2010 Feb;7(2 Pt 2):1023-8.

4: Barber MD, Walters MD, Cundiff GW; PESSRI Trial Group. Responsiveness of the Pelvic Floor Distress Inventory (PFDI) and Pelvic Floor Impact Questionnaire (PFIQ) in women undergoing vaginal surgery and pessary treatment for pelvic organ prolapse. Am J Obstet Gynecol. 2006 May;194(5):1492-8.

References Systematic review of factors influencing pessary fitting and continuation

1. Abdool Z, Thakar R, Sultan AH, Oliver RS (2010) Prospective evaluation of outcome of

vaginal pessaries versus surgery in women with symptomatic pelvic organ prolapse. Int Urogynecol J Pelvic Floor Dysfunct 16

2. Alperin M, Khan A, Dubina E, Tarnay C, Wu N, Pashos CL, Anger JT.Female Pelvic Med Reconstr Surg. 2013 May-Jun;19(3):142-7.Patterns of pessary care and outcomes for medicare beneficiaries with pelvic organ prolapse.
3. Bai SW, Yoon BS, Kwon JY, Shin JS, Kim SK, Park KH (2005) Survey of the characteristics and satisfaction degree of the patients using a pessary. Int Urogynecol J Pelvic Floor Dysfunct 16 (3):182–186
4. Barber MD, Walters MD, Cundiff GW (2006) Responsiveness of the Pelvic Floor Distress Inventory (PFDI) and Pelvic Floor Impact Questionnaire (PFIQ) in women undergoing vaginal surgery and pessary treatment for pelvic organ prolapse. Am JObstet Gynecol 194(5):1492–149
5. Brincat C, Kenton K, Pat FM, Brubaker L (2004) Sexual activity predicts continued pessary use. Am J Obstet Gynecol 191(1):198–200
6. Broens-Oostveen MC, Mom RM, Lagro-Janssen AL (2004)Genital prolapse; treatment and course in four general practices.Ned Tijdschr Geneeskd 148(29):1444–1448, 17
7. Chan SS, Cheung RY, Yiu KW, Lee LL, Pang AW, Chung TK. Int Urogynecol J. 2012 Aug;23(8):1027-33. Symptoms, quality of life, and factors affecting women's treatment decisions regarding pelvic organ prolapse.
8. Clemons JL, Aguilar VC, Sokol ER, Jackson ND, Myers DL (2004) Patient characteristics that are associated with continued pessary use versus surgery after 1 year. Am J Obstet Gynecol 191(1):159–164
9. Cundiff GW, Amundsen CL, Bent AE, Coates KW, Schaffer JI, Strohbehn K et al (2007) The PESSRI study: symptom relief outcomes of a randomized crossover trial of the ring and Gellhorn pessaries. Am J Obstet Gynecol 196(4):405–408
10. Fernando RJ, Thakar R, Sultan AH, Shah SM, Jones PW (2006) Effect of vaginal pessaries on symptoms associated with pelvic organ prolapse. Obstet Gynecol 108(1):93–99
11. Friedman S, Sandhu KS, Wang C, Mikhail MS, Banks E (2010) Factors influencing long-term pessary use. Int Urogynecol J Pelvic Floor Dysfunct 21(6):673–678
12. Hanson LA, Schulz JA, Flood CG, Cooley B, Tam F (2006) Vaginal pessaries in managing women with pelvic organ prolapse and urinary incontinence: patient characteristics and factors contributing to success. Int Urogynecol J Pelvic Floor Dysfunct 17(2):155–159
13. Jones K,Yang L, Lowder JL, Meyn L, Ellison R, ZyczynskiHMet al (2008) Effect of pessary use on genital hiatus measurements in women with pelvic organ prolapse. Obstet Gynecol 112(3):630–636
14. Geoffrion R, Zhang T, Lee T, Cundiff GW.Female Pelvic Med Reconstr Surg. 2013 Nov-Dec;19(6):339-45. Clinical characteristics associated with unsuccessful pessary fitting outcomes.
15. Komesu YM, Rogers RG, Rode MA, Craig EC, Gallegos KA, Montoya AR et al (2007) Pelvic floor symptom changes incpessary users. Am J Obstet Gynecol 197(6):620–626
16. Kuhn A, Bapst D, Stadlmayr W, Vits K, Mueller MD (2009) Sexual and organ function in patients with symptomatic prolapse: are pessaries helpful? Fertil Steril 91(5):1914–1918
17. Lone F1, Thakar R, Sultan AH, Karamalis G. A 5-year prospective study of vaginal pessary use for pelvic organ prolapse. Int J Gynaecol Obstet. 2011 Jul;114(1):56-9.
18. Maito JM, Quam ZA, Craig E, Danner KA, Rogers RG (2006) Predictors of successful pessary fitting and continued use in a nurse-midwifery pessary clinic. J Midwifery Womens Health 51 (2):78–84
19. Mamik MM, Rogers RG, Qualls CR, Komesu YM. Goal attainment after treatment in patients with symptomatic pelvic organ prolapse. Am J Obstet Gynecol. 2013 Nov;209(5):488.e1-5.
20. Manchana T, Bunyavejchevin S. Impact on quality of life after ring pessary use for pelvic organ prolapse. Int Urogynecol J. 2012 Jul;23(7):873-7.
21. Markle D, Skoczylas L, Goldsmith C, Noblett K. Patient characteristics associated with a

successful pessary fitting. Female Pelvic Med Reconstr Surg. 2011 Sep;17(5):249-52.
22. Mokrzycki ML, Hatangadi SB, Zaccardi JE, Cox S. Preexisting stress urinary incontinence: a predictor of discontinuation with pessary management. J Low Genit TractDis. 2001 Oct;5(4):204-7.
23. Mutone MF, Terry C, Hale DS, Benson JT (2005) Factors which influence the short-term success of pessary management of pelvic organ prolapse. Am J Obstet Gynecol 193(1):89–94
24. Nguyen JN, Jones CR. Pessary treatment of pelvic relaxation: factors affecting successful fitting and continued use. J Wound Ostomy Continence Nurs. 2005;32(4):255-61; quiz 262-3.
25. Patel M, Mellen C, O'Sullivan DM, Lasala CA. (2010) Impact ofpessary use on prolapse symptoms, quality of life, and bodyimage. Am J Obstet Gynecol 18
26. Powers K, Lazarou G, Wang A, LaCombe J, Bensinger G.Greston WM et al (2006) Pessary use in advanced pelvic organprolapse. Int Urogynecol J Pelvic Floor Dysfunct17(2):160–164.

Reference list Systematic review of factors influencing failure of POP surgery

1. Whiteside JL, Weber AM, Meyn LA, Walters MD. Am J Obstet Gynecol. 2004 Nov;191(5):1533-8.factors for prolapse recurrence after vaginal repair.
2. Diez-Itza I, Aizpitarte I, Becerro A. Int Urogynecol J Pelvic Floor Dysfunct. 2007 Nov;18(11):1317-24. Risk factors for the recurrence of pelvic organ prolapse after vaginal surgery: a review at 5 years after surgery.
3. Fialkow MF, Newton KM, Weiss NS. Int Urogynecol J Pelvic Floor Dysfunct. 2008 Nov;19(11):1483-7.Incidence of recurrent pelvic organ prolapse 10 years following primary surgical management: a retrospective cohort study.
4. Jeon MJ, Chung SM, Jung HJ, Kim SK, Bai SW. Gynecol Obstet Invest. 2008;66(4):268- 73 Risk factors for the recurrence of pelvic organ prolapse.
5. Dällenbach P, Jungo Nancoz C, Eperon I, Dubuisson JB, Boulvain M. Int Urogynecol J. 2012 Jan;23(1):35-41. Incidence and risk factors for reoperation of surgically treated pelvic organ prolapse.
6. Weemhoff M, Vergeldt TF, Notten K, Serroyen J, Kampschoer PH, Roumen FJ. Int Urogynecol J. 2012 Jan;23(1):65-71. Avulsion of puborectalis muscle and other risk factors for cystocele recurrence: a 2-year follow-up study.
7. Tegerstedt G1, Hammarström M. Acta Obstet Gynecol Scand. 2004 Aug;83(8):758-63. Operation for pelvic organ prolapse: a follow-up study.
8. Medina CA, Candiotti K, Takacs P. Int J Gynaecol Obstet. 2008 May;101(2):184-7. Wide genital hiatus is a risk factor for recurrence following anterior vaginal repair.
9. Salvatore S1, Athanasiou S, Digesu GA, Soligo M, Sotiropoulou M, Serati M, Antsaklis A, Milani R. Neurourol Urodyn. 2009;28(4):301-4. doi: 10.1002/nau.20639. Identification of risk factors for genital prolapse recurrence.
10. Kawasaki A, Corey EG, Laskey RA, Weidner AC, Siddiqui NY, Wu JM. J Reprod Med. 2013 May-Jun;58(5-6):195-9. Obesity as a risk for the recurrence of anterior vaginal wall prolaps after anterior colporrhaphy.

Study protocol

Appendix 1:

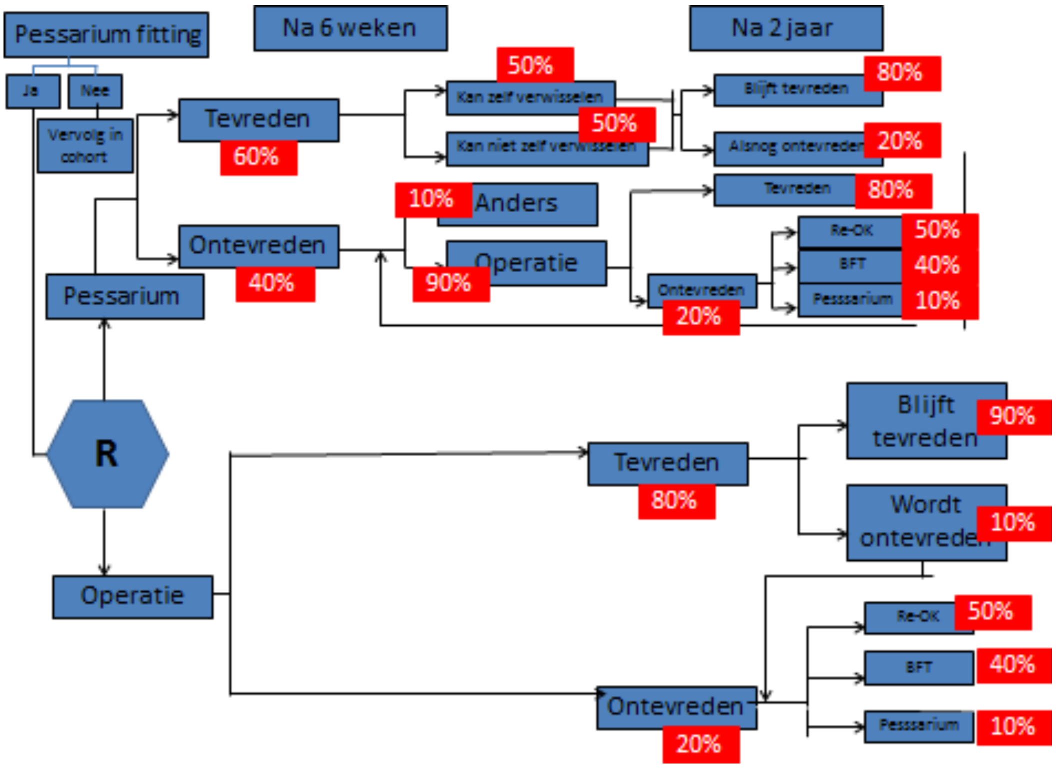

Study protocol

Appendix 2 Review on (Cost) effectiveness of pessary use as compared to surgery:

Reference	Study type	Characteristics	Intervention (I)	Controls (C)	Outcome measures and follow-uptime	Results
**Mamik, 2012**<br><br>**AJOG 2013:209:488**	**Design**<br>**Case-control**<br>**N = 100**<br>**Country**<br>**US**	**Aim: compare goal achievement and global improvement between pessary and surgery for [illegible] stage ≥2.**<br><br>**Inclusion criteria: >18 year old, read and write in English**<br><br>**Exclusion: not given**	**Vaginal pessary**<br>**N = 50**	**Prolapse surgery**<br>**N = 50**	**Primary outcome:**<br>**Goal attainment**<br><br>**Secondary:**<br>**PGI-I**<br>**PFDI-20**<br>**PISQ-12**<br>**Body Image scale**<br><br>**Follow-up: 3 months**	**Primary outcome:**<br>**Goal attainment sign. higher score after surgery (8.6 vs 6.4)**<br>**Secondary outcomes**<br>**PGI-I sign (p=0.04) better improvement after surgery (2.4vs 1.9 points)**<br>**PFDI-20 sign (p=0.02) higher change (89 vs 43 points)**<br>**PISQ-12 and BIS no sign difference**<br><br>**Additional: 10% crossed over from pessary to surgery within 3 months [illegible]% referred from surgery after they had been selected as eligible.**<br>**No follow-up in pessary group is 40% (20/50) and surgery 30% [illegible]**
**Abdool, 2011**	**Design**<br>**Cohort study**<br><br>**N total = [illegible]**<br><br>**Country:**<br>**UK**	**Aim of the study**<br>**to evaluate and compare the effectiveness of pessariesand surgery in women with symptomatic pelvic organ prolapse.**<br>**Inclusion criteria**<br>**- Women referred to a specialist urogynaecology unit with symptomatic [illegible] between June 2002 andMay 2007**	**Intervention vaginal pessary**<br>**N = 359**	**Controls surgery**<br>**N = 195**	**Primary outcomes:**<br>**Sheffield POP questionnaire [illegible]**<br><br>**Secundary outcomes:**<br>**None**<br>**Follow up:**<br>**For the surgery and pessary groups 14 months(SD 6.14) and 12 months (SD 3.1), respectively.**	**Primary outcomes:**<br>**No difference in functional outcome after 1 year follow-up between groups**<br>**Additional:**<br>**Only 45% in pessary group en 55% in surgery group responded at 12monthsIn pessary group 24.7% (89/359) crossed to surgery but were not analyzed In pessary group 7.3% stopped because of other reasons.**<br>**Selection and patient preference bias**<br>**The mean age was significantly higher in the pessary group compared to the surgerygroup (68.4 +/− 13.08 vs 60.4 +/−12.25 years, respectively).**

Study protocol

		**Exclusion criteria**<br>· **Subjects fitted with pessaries for urinary incontinence and those who had concomitant urinary incontinence surgery (e.g. TVT)**<br>· **Subjects who started inthe pessary group but subsequently requested surgery were excluded from analysis in both the surgery and pessary group.**				

Ben ÂJ, *et al. BMJ Open* 2024; 14:e075016. doi: 10.1136/bmjopen-2023-075016

Study protocol

**Lowenstein 2010**<br><br>**J Sex Med 2010; 7:-1023**<br><br>**28**	**Design**<br>**Cohort study**<br>**N= 235**<br><br>**Country**<br>**US**	**Aim of the study**<br>**First to evaluate patient-reported outcome, POP symptoms, sexual functioning and body image following treatment of P**<br>**Second to compare surgery with pessary**<br><br>**Inclusion: ≥18 year, ≥ satge 2 POP, complete questionnaire at baseline and at ≥6 months follow up**<br><br>**Exclusion: recurrent UTI, peripheral neuropathy, using pessary at initial presentationor POP surgery < 6 months prior to presentation**	**Intervention**<br>**N = 202 surgery**	**Controls**<br>**N = 33 pessary**	**Primary outcomes**<br>**PFDI-20**<br>**PISQ-12**<br>**Modified Body Image scale**<br><br>**All at six months follow-up**	**Results**<br>**After multivariate analyses, including type of intervention, BMI and differencein Body image were associated with change in total PISQ (sexual functioning) score**<br><br>**In the pessary group there was no significant improvement in sexual functioningas compared to surgery (-2.5 versus +11.5)**<br><br>**Additional:**<br>**No figures presented for pessary and surgery group, with exemption oftheSexual functioning (PISQ-12) result above.**

Study protocol

**Barber, 2006**	**Design**<br>Case-control study<br>**Ntotal=106Country:**<br>USA	Aim of the studyto evaluate the responsiveness of the PelvicFloor Distress Inventory (PFDI) andelicvPFloor Impact Questionnaire (PFIQ) in women with pelvic organ prolapse undergoingsurgical and nonsurgical management.<br><br>Inclusion criteria<br>Surgery group:<br>Stage III or IV prolapse, wereat least 18 years, and scheduled for vaginal prolapse repair.<br>Pessary group:<br>women with symptomatic pelvic organ prolapse ofstageII or greater. (Pessri trial)<br><br>Exclusion criteriaSurgery group:<br>· mentally or physically incapable of completingthe questionnaires.<br>Pessary group:<br>· were pregnant, were currently using a pessary, orhad vaginal agglutination that precluded pessary insertion.	**Intervention**<br>**Pessary in women with stage II or greater POP**<br>**N = 42**	**Controls**<br>**Surgery in womenwithstage III or greater POP**<br>**N = 64**	**Primary outcomes:**<br>**PFDI and PFIQ**<br>**Secundary outcomes:**<br><br>**Follow up:**<br>**3 months (Pessary group)or 6 months (Surgery group) after initiationoftreatment.**	**Primary outcomes:**<br><br>**After controlling for preoperative prolapse stage and baseline HRQOLscores,subjects in the Surgery group had significantly greater improvement in each of the scales of the PFDI and the prolapse and urinary scales of the PFIQ than did the Pessary group.**<br>**Scores from each of the scales of the PFDI improved by 14 to 15**<br>**points more on average after treatment in the Surgery group than those ofthe Pessary group (P < .01 for each) after adjusting for the above baseline differences.**<br><br>**Similarly, for the prolapse and urinary scales of the PFIQ, scores improved13and 17 points more, respectively, in the Surgery group than the Pessary group after treatment. (P < .05 for each).**<br><br>**Four of 64 (6%) of subjects in the Surgery group had recurrent prolapsedevelopbeyond the hymen by 6 months after surgery. No subjects underwent reoperation for recurrent prolapse during the study period.**<br>**Additional:**<br><br>**Difference in followup**<br>**Selection bias**

101

Appendix 3 Review on risk factors for failure of pessaries:

study		n	FU period	success initial	continued pes	stopped pes	cross over surgery	predict fit	predict contin
Wu 1997	retrospective	110	3 yrs	81 (74%)	43 (39%)		at least 5	nee	ja
Mokrzycki 2001	retrospective	42	3 mth		24 (57%)	18 (43%)	nr	nee	ja
Clemons 2004	prospective	100	1 yr	73 (73%)	43 (43%)		16/73 (27%)	nee	ja
Brincat 2004	retrospective	169	2,5 yr	144 (85%)	82 (60%)	54 (40%)	32/144 (22%)	nee	ja
Mutone 2004	retrospective	407	3 wks	168 (41%)		96 (57%)	nr	ja	ja
Powers 2006	retrospective	32	1 yr			20 (62%)	12 (38%)	nee	ja
Broens-Oostveen 2004	retrospective	192	9 yr			107 (52%)	nr	nee	ja
Nguyen 2005	retrospective	130	1 yr	111 (82%)	85 (65%)		20 (15%)	ja	ja
Hanson 2005	retrospective	1216	3 mth	1043 (86%)	744 (71%)		nr	nee	ja
Fernando 2006	prospective	203	4 mth	nr	153 (75%)		28 (14%)	nee	ja
Barber 2006	prospective	42	3 mth	nr		0	nr	nee	nee
Maito 2006	retrospective	120	6 mth	106 (88%)	92 (77%)		9 (8%)	ja	ja
Cundiff 2007	prospective	85	1 yr	65/71 (92%)			5 (6%)	nee	nee
Komesu 2007	prospective	64	6-12 mth		36 (56%)	28 (44%)	2 (3%)	nee	ja
Jones 2008	prospective	90	3 mth		42 (47%)		nr	nee	ja
Kuhn 2009	prospective	71	3 mth		32 (45%)		nr	nee	nee
Sarma 2009	retrospective	120	1 mth		45 (38%)		18 (15%)	nee	nee
Friedman 2010	retrospective	150	1 yr		115 (77%)	35 (23%)	25 (17%)	nee	ja
Patel 2010	prospective	65	3 mth	54 (83%)	54 (83%)		5 (8%)	nee	nee
Abdool 2010	prospective	359	1 yr	296 (83%)	243 (68%)		31 (9%)	nee	nee
Lone 2011	prospective	246	5 yr	187 (76%)	130 (53%)		70 (28%)	nee	nee
Markle 2011	retrospective	158	1 week	92 (59%)			nr	ja	nee
Chan 2012	prospective	197	1 yr	138 (70%)		59 (30%)	nr	nee	ja
Manchana 2012	retrospective	126	1 yr	77 (61%)	64 (51%)		5 (4%)	nee	nee
Mamik 2013	prospective	50	3 mth				5 (10%)	nee	nee
Geoffrion 2013	retrospective	101	4 wks	79 (78%)			nr	nee	ja
Alperin 2013	retrospective	4019	9 years				12% 1 yr, 24% 9 yrs	nee	nee
Succesfull fitting		3589		2714(76%)					
Continuation		3381			2027 (60%)				

Appendix 4 Review on risk factors for failure of surgery:

Risk factor	Investigated:	Significant:
Preoperative stage	8	5
Age	8	2
Obesity	7	0
Parity	5	0
Constipation	5	0
Pulmonary disease	5	0
Number of sites involved preoperative	4	1
Menopausal status	4	0
Hysterectomy status	4	0
Concomitent surgery	3	1
Family history	3	1
Complicated delivery	3	0
Diabetes	3	0
Smoking	3	0
Previous incontinence and/or prolapse surgery	2	2
Hiatus genitalis	2	1
Weight	2	1
Any incontinence preoperative	2	1
Delivery mode	2	0
Vaginal delivery	2	0
Hormone replacement therapy	2	0
Previous prolapse surgery	2	0
Surgeons experience	2	0
Abcense of posterior repair	1	1
Sexual activity	1	1
Levator defect	1	1
Height	1	0
Birth weight	1	0
Age at last delivery	1	0
Site of most advanced prolapse	1	0
Surgical approach	1	0
Use of Mesh	1	0
Previous incontinence surgery	1	0
Previous pelvic floor surgery or hysterectomy	1	0
Abdominal hernias	1	0
Cardiovascular disease	1	0
Intense physical exercise	1	0
Heavy lifting	1	0
Heavy lifting or constipation	1	0
Levator muscle contraction	1	0
Weight of the uterus	1	0
Postoperative complications	1	0
Incomplete emptying of bladder	1	0
Fecal incontinence	1	0

Study protocol

Appendix 5 diagram/tabel bezoeken, tijdstippen, onderzoeken

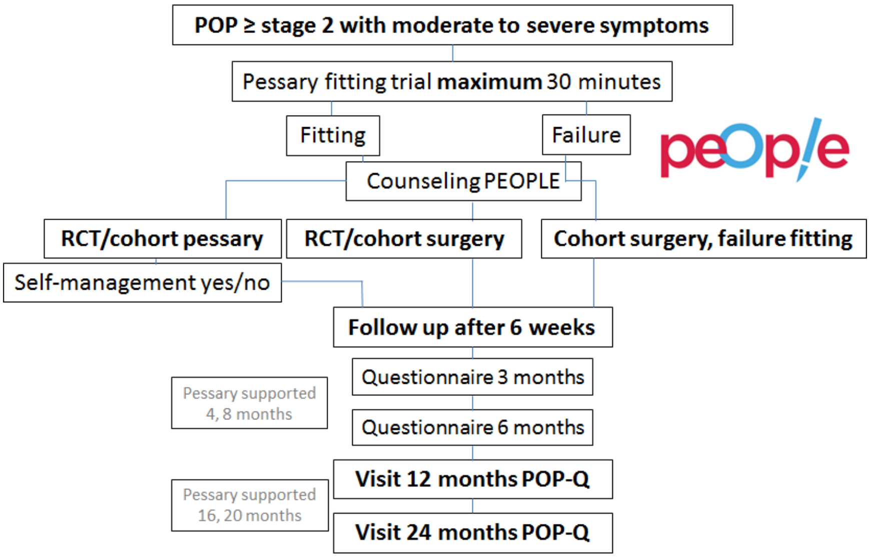

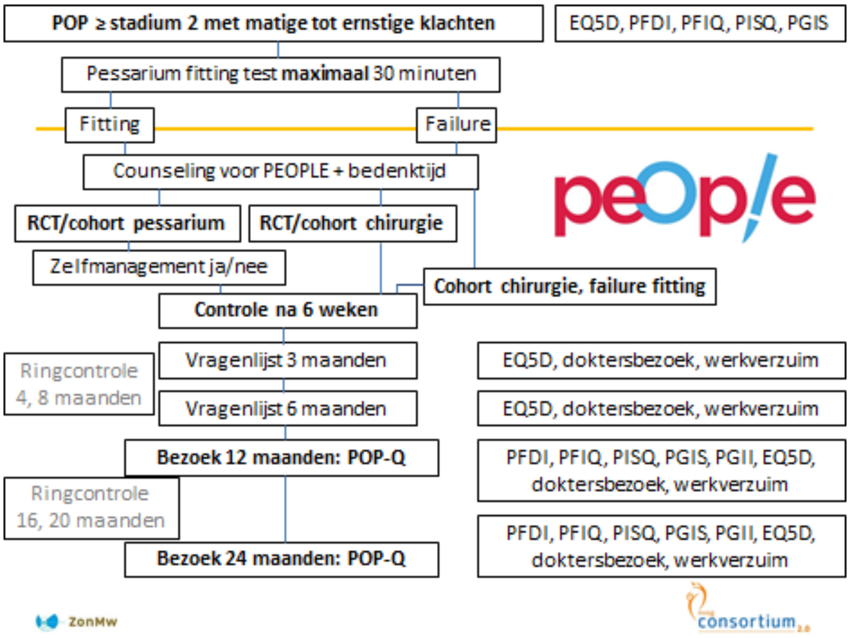

Chirurgie en cohort fitting failure

Contact	Bezoek arts	POPQ	Eq5d<br>doktersbezoek<br>werkverzuim	PFIQ<br>PFDI<br>PISQ<br>PGII<br>PGIS

1. Eerste bezoek	X	X	Eq5D	X<br>(zonder PGII)
2. 6 weken	X			
3. 3 maanden			X	
4. 6 maanden			X	
5. 12 maanden	X	X	X	X
6. 24 maanden	X	X	X	X

Pessarium met zelfmanagement

**Contact**	**Bezoek arts**	**POPQ**	**Eq5d<br>doktersbezoek<br>werkverzuim**	**PFIQ<br>PFDI<br>PISQ<br>PGII<br>PGIS**
1. Eerste bezoek	X	X	Eq5D	X<br>(zonder PGII)
2. 6 weken	X			
3. 3 maanden			X	
4. 6 maanden			X	
5. 12 maanden	X	X	X	X
6. 24 maanden	X	X	X	X

Study protocol

Pessarium zonder zelfmanagement

Contact	Bezoek arts	POPQ	Ringcontrole	Eq5d doktersbezoek werkverzuim	PFIQ PFDI PISQ PGII PGIS
1. Eerste bezoek	X	X		Eq5D	X (zonder PGII)
2. 6 weken	X				
3. 3 maanden				X	
4. 4 maanden	X		X		
5. 6 maanden				X	
6. 8 maanden	X		X		
7. 12 maanden	X	X	X	X	X
8. 16 maanden	X		X		
9. 20 maanden	X		X		
10. 24 maanden	X	X	X	X	X

1.3 Summary of amendment to study protocol

The main change in the final version is the addition of an observational cohort performed alongside the RCT. We added this observational cohort since many women refused to participate in the RCT due to treatment preference. In case a woman was willing to participate in the study but actively opted for one of two treatment options she was followed in the observational cohort. The same study parameters and follow-up were used in both the trial and observational cohort. See section 2, section 3, section 4.4, section 8.2, section 10, section 11.2

1.3.1 Detailed summary of all amendments

1. Addition of multiple centers for participation.

Added centers:

- Atrium MC Heerlen
- Academisch ziekenhuis Maastricht
- Martini ziekenhuis Groningen
- MST Enschede
- ZGT Almelo / Hengelo
- Deventer ziekenhuis
- Jeroen Bosch ziekenhuis
- Amstelland ziekenhuis
- Tergooi ziekenhuis
- Albert Schweitzer ziekenhuis
- Canisius Wilhelmina ziekenhuis
- Maxima Medisch Centrum
- MCH-Bronovo
- OLVG
- HAGA

2. Change in investigators at the following participating centers:
    - St. Antonius hospital. S. The was replaced by E. Vernooij
    - Canisius hospital. C.F. van Heteren was replaced by K.L. Bos
    - Maastricht University center (MUMC): G. Link was replaced by W.A. Spaans

3. Change in Head of Department of Reproductive Medicine and Gynaecology.

4. Change in Objective.

An observational cohort was added since many women refused to participate in the trial due to treatment preference. At first, women were asked to participate in the trial. In case the woman is willing to participate but actively opts for one of both treatments, she will be followed in a cohort ‘own choice’.

5. Change in study design.

In the first version it is noted that for women with an unsuccessful pessary fitting only baseline characteristics will be recorded. However, these women will be followed in the cohort fitting failure with the same follow-up as for the trial (24-months). Appendix 5 has been noted in more detail.

6. Addition in sample size calculation for observational cohort.

Since we added an observational cohort with women who made their own choice of treatment, we added this to the section sample size calculation. In the cohort we include all patients who are willing to collaborate on this research but have a preference for one of both therapies. We now assume that 70% of the eligible patients object participation in the RCT, and that 90% of them is nevertheless willing to participate in the cohort.

7. Change in self-management of pessary treatment.

In case self-management was performed, women were advised to change their pessary every 4-months, instead of every 1 month.

8. Observational cohort is added in randomization section.

In case the woman is willing to participate but actively opts for one of both treatments, she will also be registered in ALEA. All groups will have the same data collection and follow up as displayed in appendix 5.

9. Observational cohort added in statistical analysis section.

The cohort with patients treated according their preference will be analyzed separately from the randomized trial. The same analysis will be done.

10. Change in exclusion criteria.

Women with a previous hysterectomy were only excluded in case the indication for the hysterectomy was a prolapse.

11. Observational cohort added in recruitment.

In case the woman is willing to participate but actively opts for one of both treatments, she can attend the cohort.

12. Change in monitoring

At first, the monitoring was coordinated by the Staff Member Clinical Research, quality coordinator of division women and baby. Later on, the monitoring was conducted by the Dutch consortium and was executed by a qualified intern monitor.

13. POP-Q only performed at 12- and 24-months follow-up, not at 6 weeks visit.

Demonstrated in the tables listed in appendix 5.

1.3.2 Table with amendments and corresponding section

Amendment	Corresponding section in the final version 1.22
1. Addition of multiple centers for participation	First table with project information
2. Change in investigators	First table with project information
3. Change in Head of Department	Protocol signature sheet
4. Change in objective	Section 2
5. Change in study design	Section 3
6. Addition in sample size calculation for observational cohort	Section 4.4
7. Change in self-management of pessary treatment	Section 5.1
8. Observational cohort is added in randomization section	Section 8.2
9. Observational cohort added in statistical analysis section	Section 10
10. Change in exclusion criteria	Section 10.2
11. Observational cohort added in recruitment	Section 11.2
12. Change in monitoring	Section 12.2
13. POP-Q only performed at 12- and 24-months	Appendix 5
