# Supplementary material for: Cost-effectiveness of pessary therapy versus surgery for symptomatic pelvic organ prolapse: an economic evaluation alongside a randomised non-inferiority controlled trial
Source: BMJ Open. 2024 May 1;14(5):e075016. doi: 10.1136/bmjopen-2023-075016 (PMC11086579; doi:10.1136/bmjopen-2023-075016)
Supplement: Supplementary data [file bmjopen-2023-075016supp002.pdf]

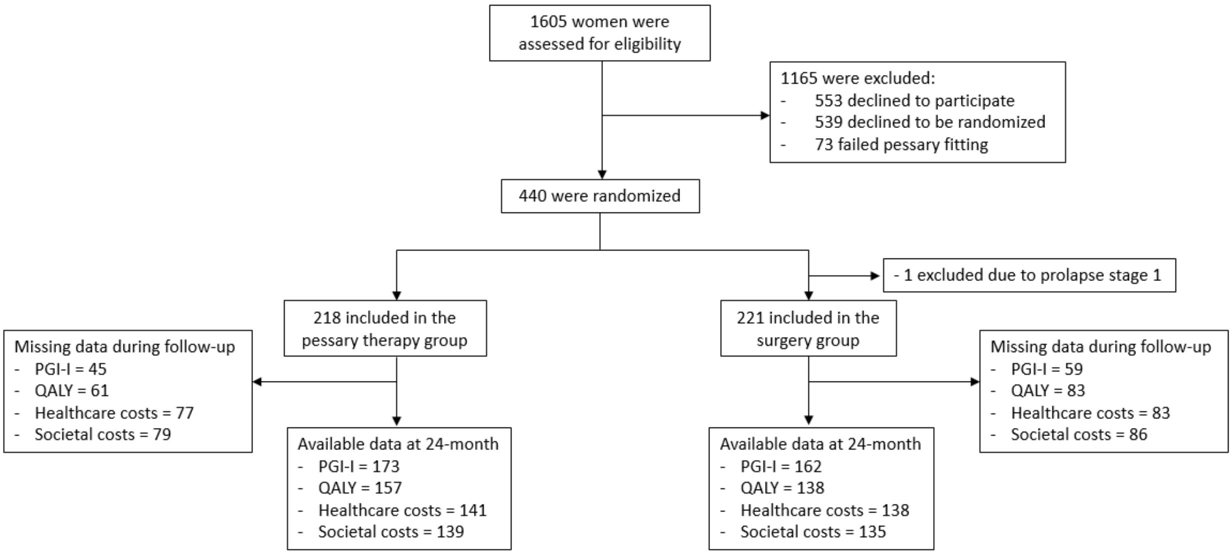

SUPPLEMENTARY Figure 1. FLOW DIAGRAM. Inclusion and available data at 24-month follow-up.

SUPPLEMENTARY TABLE 1. RESOURCES AND UNIT COSTS

Resources	Unit costs	Year	Reference
Pessary device			
Milex®	€64	2022	Market price: bol.com
Arabin®	€73	2022	Market price: bol.com
Other brand (average)	€68	2022	Market price: bol.com
Pessary placement	€109	2022	Dutch costing manual[1]
Surgery			
Sacrospinous hysteropexy (care product 149999033)	€5835	2022	DBC[2]
Sacrospinous fixation (care product 149999047)	€4640	2022	DBC[2]
Manchester–Fothergill procedure (care product 149999047)	€4640	2022	DBC[2]
Abdominal sacrocolpopexy (care product 149999033)	€5835	2022	DBC[2]
Sacrocervicopexy care product 149999033)	€5835	2022	DBC[2]
Vaginal hysterectomy (care product 149999047)	€4640	2022	DBC[2]
Average surgical procedures costs (used as WTP threshold)	€5237	2022	DBC[2]
**Other resources**			
General practitioner consultation	€39	2022	Dutch costing manual[1]
Other healthcare professional consultation at primary care	€39	2022	Dutch costing manual[1]
Medical specialist consultation at secondary care	€109	2022	Dutch costing manual[1]
Hospital readmission (1 day)	€568	2022	Dutch costing manual[1]
Paid working hour for women	€38	2022	Dutch costing manual[1]

DBC: Diagnosis Treatment Combination, in Dutch *Diagnose Behandeling CombinatieI.*

References:

1 Kanters TA, Bouwmans CAM, van der Linden N, *et al.* Update of the Dutch manual for costing studies in health care. *PLoS One* 2017;**12**. doi:10.1371/journal.pone.0187477

2 Diagnose Behandeling Combinatie (DBC) open data - NZa. https://www.opendisdata.nl/ (accessed 3 Sep 2022).
