# Supplementary material for: Cost-effectiveness of pessary therapy versus surgery for symptomatic pelvic organ prolapse: an economic evaluation alongside a randomised non-inferiority controlled trial
Source: BMJ Open. 2024 May 1;14(5):e075016. doi: 10.1136/bmjopen-2023-075016 (PMC11086579; doi:10.1136/bmjopen-2023-075016)
Supplement: Author's manuscript [file bmjopen-2023-075016.draft_revisions.pdf]

BMJ Open

BMJ Open is committed to open peer review. As part of this commitment we make the peer review history of every article we publish publicly available.

When an article is published we post the peer reviewers' comments and the authors' responses online. We also post the versions of the paper that were used during peer review. These are the versions that the peer review comments apply to.

The versions of the paper that follow are the versions that were submitted during the peer review process. They are not the versions of record or the final published versions. They should not be cited or distributed as the published version of this manuscript.

BMJ Open is an open access journal and the full, final, typeset and author-corrected version of record of the manuscript is available on our site with no access controls, subscription charges or pay-per-view fees (http://bmjopen.bmj.com).

If you have any questions on BMJ Open's open peer review process please email info.bmjopen@bmj.com

BMJ Open

Cost-effectiveness of pessary therapy versus surgery for symptomatic pelvic organ prolapse: an economic evaluation alongside a randomized non-inferiority controlled trial

Journal:	*BMJ Open*
Manuscript ID	bmjopen-2023-075016
Article Type:	Original research
Date Submitted by the Author:	25-Apr-2023

Complete List of Authors:	Ben, Ângela; Vrije Universiteit Amsterdam, Health Sciences<br>van der Vaart, Lisa R.; University of Amsterdam<br>Bosmans, J; VU University Amsterdam<br>Roovers, Jan-Paul W.R.; University of Amsterdam, Department of Obstetrics and Gynecology; Women's Health Bergman Clinics, Department of Gynecology<br>Lagro-Janssen, Antoinette; Radboud university medical center, Department of Primary and Community Care<br>van der Vaart, Carl H.; University Medical Center Utrecht, Department of Obstetrics and Gynecology; Women's Health Bergman Clinics, Department of Gynecology<br>Vollebregt, Astrid; Spaarne Hospital, Department of Obstetrics and Gynecology

Keywords:	Pelvic Pain, Urogynaecology < GYNAECOLOGY, Health economics < HEALTH SERVICES ADMINISTRATION & MANAGEMENT

SCHOLARONE™
Manuscripts

I, the Submitting Author has the right to grant and does grant on behalf of all authors of the Work (as defined in the below author licence), an exclusive licence and/or a non-exclusive licence for contributions from authors who are: i) UK Crown employees; ii) where BMJ has agreed a CC-BY licence shall apply, and/or iii) in accordance with the terms applicable for US Federal Government officers or employees acting as part of their official duties; on a worldwide, perpetual, irrevocable, royalty-free basis to BMJ Publishing Group Ltd ("BMJ") its licensees and where the relevant Journal is co-owned by BMJ to the co-owners of the Journal, to publish the Work in this journal and any other BMJ products and to exploit all rights, as set out in our licence.

The Submitting Author accepts and understands that any supply made under these terms is made by BMJ to the Submitting Author unless you are acting as an employee on behalf of your employer or a postgraduate student of an affiliated institution which is paying any applicable article publishing charge ("APC") for Open Access articles. Where the Submitting Author wishes to make the Work available on an Open Access basis (and intends to pay the relevant APC), the terms of reuse of such Open Access shall be governed by a Creative Commons licence – details of these licences and which Creative Commons licence will apply to this Work are set out in our licence referred to above.

Other than as permitted in any relevant BMJ Author's Self Archiving Policies, I confirm this Work has not been accepted for publication elsewhere, is not being considered for publication elsewhere and does not duplicate material already published. I confirm all authors consent to publication of this Work and authorise the granting of this licence.

For peer review only

Cost-effectiveness of pessary therapy versus surgery for symptomatic pelvic organ prolapse: an economic evaluation alongside a randomized non-inferiority controlled trial

Ângela Jornada Ben MD, Ph.D.[1], Lisa R. van der Vaart MD [2], Judith E. Bosmans Ph.D.[1], Jan-Paul W.R. Roovers MD, Ph.D [2,3], Antoine L. Lagro-Janssen MD, Ph.D [4], Carl H. van der Vaart MD, Ph.D [5,6] Astrid Vollebregt MD, Ph.D.[7]

[1] Department of Health Sciences, Faculty of Science, Vrije Universiteit Amsterdam, Amsterdam Public Health research institute, Amsterdam, The Netherlands.

[2] Department of Obstetrics and Gynecology, Amsterdam Reproduction & Development Research Institute, Amsterdam UMC, University of Amsterdam, Amsterdam, The Netherlands.

[3] Department of Gynecology, Women's Health, Bergman Clinics, Amsterdam, the Netherlands.

[4] Department of General Practice/ Women's studies Medicine, University Medical Center Radboud, Nijmegen, The Netherlands

[5] Department of Obstetrics and Gynecology, University Medical Center Utrecht, Utrecht, The Netherlands.

[6] Department of Gynecology, Women's Health, Bergman Clinics, Hilversum, the Netherlands.

[7] Department of Obstetrics and Gynecology, Spaarne Hospital, Haarlem, The Netherlands.

Corresponding author:

Ângela Jornada Ben. ORCID: 0000-0003-4793-9026. Department of Health Sciences, Faculty Science, Vrije Universiteit Amsterdam, Van der Boechorststraat 7, 1081 BT Amsterdam, The Netherlands. E-mail: a.jornadaben@vu.nl. Telephone: +31 6 25304906.

Orchid numbers:

Ângela J. Ben: 0000-0003-4793-9026

Lisa R van der Vaart: 0000-0003-1195-0047

Judith E. Bosmans: 0000-0002-1443-1026

Jan-Paul W.R. Roovers: 0000-0002-3069-6382

Antoine L. Lagro-Janssen: 0000-0003-2339-3743

Carl H. van der Vaart: 0000-0003-0890-5368

Astrid Vollebregt: 0000-0002-4704-1710

For peer review only - http://bmjopen.bmj.com/site/about/guidelines.xhtml

ABSTRACT

Objective: To evaluate the cost-effectiveness of pessary therapy as an initial treatment option compared to surgery for moderate to severe POP symptoms in secondary care from a healthcare and a societal perspective.

Design: Economic evaluation alongside a multicenter randomized controlled non-inferiority trial with a 24-month follow-up.

Setting: 21 hospitals in the Netherlands, recruitment conducted between 2015 – 2022.

Participants: 1605 women referred to secondary care with symptomatic prolapse stage ≥ 2 were requested to participate. Of them, 440 women gave informed consent and were randomized to pessary therapy (n=218) or to surgery (n=222) in a 1:1 ratio stratified by hospital.

Interventions: Pessary therapy and surgery.

Primary and secondary outcome measures: The Patient Global Impression of Improvement (PGI-I), a 7-point scale dichotomized into successful *vs.* unsuccessful, with a non-inferiority margin of -10%; Quality-Adjusted Life-Years (QALYs) measured by the EQ-5D-3L; healthcare and societal costs were based on medical records and the institute for Medical Technology Assessment (iMTA) questionnaires.

Results: For the PGI-I, the mean difference between pessary therapy and surgery was -0.03 (95% CI, -0.11; 0.06), and -0.01 (95% CI, -0.05; 0.03) for QALYs. In total, 54.1% women randomized to pessary therapy crossed over to surgery, and 3.6% underwent recurrent surgery. Healthcare and societal costs were significantly lower in the pessary therapy (mean difference=-€1780, 95% CI, -€2148; -€1422 and mean difference=-€1826, 95% CI, -€2328; -€1322 respectively). The probability that pessary therapy is cost-effective compared to surgery was 1 at willingness-to-pay thresholds between €0 and €20000/QALY gained from both perspectives.

Conclusions: Non-inferiority of pessary therapy regarding the PGI-I could not be shown and no statistically significant differences in QALYs between interventions were found. Due to significantly lower costs, pessary therapy is likely to be cost-effective compared to surgery as an initial treatment option for women with symptomatic POP treated in secondary care.

Trial registration number: https://trialsearch.who.int/ Identifier: NTR4883.

For peer review only - http://bmjopen.bmj.com/site/about/guidelines.xhtml

Strengths and limitations of this study

- This economic evaluation was performed alongside a multicenter pragmatic randomized controlled trial. The randomization process ensures that groups are comparable and decrease the likelihood of selection bias while the multicenter pragmatic design improves generalizability of results and transferability to clinical practice.
- Validated outcome measures were used and the trial had a long-term follow-up of 2 years.
- Consultations related to both interventions were provided by gynecologists, which may overestimate intervention costs, as these consultations may be provided by trained general practitioners at lower costs.
- Resource utilization related to the specific medical treatment of interventions' complications (e.g., medications), productivity costs related to unpaid work, and informal care costs were not available and, thus, not included in the analysis, which may underestimate total costs.
- Costs were estimated based on the Dutch reimbursement system and can differ from countries which may hamper the generalizability of results to healthcare systems in other countries.

Funding statement: Financial support was provided through a personal grant (receiver: Carl H. van der Vaart) issued by the ZonMW, a Dutch governmental healthcare organization. This study was funded on 26 June 2014 (project no. 837002525).

Competing interests statement: Prof. Dr. C.H. van der Vaart reports grants from ZonMW Dutch government institution grant during the conduct of the study.

Patient consent forms: patient consent forms cannot be obtained because the patient cannot be traced due to anonymization of the data.

Data sharing statement: Data is available through **Lisa R van der Vaart (l.r.vdvaart@gmail.com)** upon reasonable request. To gain access, requesters will need to sign an agreement form and confirm that data will be used for the purpose for which access was granted. Stata code are available through the corresponding author upon reasonable request.

Word count: 3937 words.

For peer review only - http://bmjopen.bmj.com/site/about/guidelines.xhtml

INTRODUCTION

Pelvic organ prolapse (POP) is a gynecological condition in which one or more of the pelvic organs (i.e., uterus, rectum, bladder, small bowel) herniate into the vagina due to weakness or damaging of the pelvic floor muscles and ligaments[1,2]. POP symptoms (e.g., urinary, bowel, and sexual dysfunction) are associated with decreased quality of life[3]. The estimated prevalence of patient-reported POP symptoms ranges from 3 to 17.7% and is expected to increase with an aging population. As a result, the demand for care and associated costs are also expected to increase[4].

Effective treatment options for moderate to severe POP symptoms include pessary therapy and surgery[5,6]. However, both treatment options are not equally effective since non-inferiority of pessary therapy compared to surgery has not been shown[7]. A pessary is a silicone flexible device that is inserted into the vagina to support the pelvic organs (i.e., uterus and bladder)[8]. An advantage of pessary therapy is its minimally invasive nature. However, adverse effects (e.g., discomfort, pain, or excessive discharge) may occur in up to 49% of women within 12 to 24 months after fitting a pessary[9,10]. As for the surgery procedure, side-effects may include urinary tract infection and urinary bladder retention which may lead to longer admission hospital stay[7]. A recent observational study in women with a strong treatment preference and a randomized trial (RCT) in women without a preference found a high crossover rate from pessary therapy to surgery of 24% and 54%, respectively[7,9]. Consequently, using pessary therapy as an initial treatment option might delay effective treatment, thereby increasing the demand for care and, thus, healthcare costs. However, using a pessary as a first treatment step would prevent expensive surgery if the pessary therapy relieves women symptoms adequately, making the initial use of pessary therapy potentially cost-effective compared to immediate surgery.

According to a recent systematic review[8], only one model-based economic evaluation based on data from United States conducted more than 10 years ago compared the cost-effectiveness of expectant management, pessary therapy and surgery for POP symptoms [11]. This review reported that both pessary therapy and surgery were cost-effective compared to expectant management[11]. The aim of this study was to further investigate the cost-effectiveness of initial pessary therapy compared to immediate surgery from a healthcare and a societal perspective for moderate to severe POP symptoms with 2 years of follow-up. This study was performed alongside a non-inferiority randomized trial, of which the results have recently been published[7].

For peer review only - http://bmjopen.bmj.com/site/about/guidelines.xhtml

METHODS

Study design

An economic evaluation was conducted alongside a non-inferiority randomized controlled trial (RCT) comparing pessary therapy and surgery as an initial treatment for moderate to severe POP in secondary care, the PEOPLE project. Participants were recruited between March 2015 and November 2019, the follow-up ended in June 2022. Detailed information about the PEOPLE project is published elsewhere[7,9,12]. This study was approved by the Medical Ethical Committee of the University Medical Center Utrecht (METC protocol number 14-533/M). No substantial changes were made to the protocol after the commencement of the RCT[7,12]. This economic evaluation is reported according to the Consolidated Health Economic Evaluating Reporting Standards statement[13]. All participants provided written informed consent.

Study population

Women with POP symptoms who were referred by their general practitioner (GP) to secondary care, were eligible for participation[7]. Inclusion criteria were POP stage ≥2 according to the Pelvic Organ Prolapse Quantification (POP-Q) system[14] and moderate to severe POP symptoms, defined as a prolapse domain score of >33 on the validated original Urinary Distress Inventory[15]. Exclusion criteria were prior prolapse or incontinence surgery, probability of future childbearing, insufficient knowledge of the Dutch language, comorbidity causing increased surgical risks, major psychiatric illness and prior pessary use[7]. Participants had to successfully complete a 30-minute pessary fitting trial to be eligible for randomization. After informed consent was signed, participants were randomly allocated to either pessary therapy or surgery in a 1:1 ratio[7]. Randomization used random permuted block sizes of 2 and 4 and was stratified by center. Due to the nature of the treatment, treatment allocation was not concealed. Women who actively opted for a treatment were asked to participate in an observational cohort performed alongside the RCT, their data were not included in economic evaluation, but published in another article[9]. Detailed information about study design and randomization can be found elsewhere[7,12].

For peer review only - http://bmjopen.bmj.com/site/about/guidelines.xhtml

Setting and location

Twenty-one Dutch hospitals participated in this multicenter RCT. In the Netherlands, women with moderate to severe POP symptoms are generally referred to secondary care. Treatment options in secondary care include pessary therapy or surgery, which are both reimbursed by the Dutch healthcare system. All gynecologist fitted at least 100 pessaries and performed 100 POP surgeries prior to study initiation.

Comparators

Pessary therapy

Two main types of pessary therapy were offered to participants, namely, supportive (i.e., ring) and occlusive (i.e., space filling)[16]. The pessary fitting was considered successful if the patient felt comfortable with the pessary in situ and if there was no pessary expulsion 30 minutes after fitting[7]. All women received verbal and written instructions on self-management of pessary therapy[7]. If self-management was not possible or preferred, an additional follow-up consultation with their gynecologist or GP was scheduled every four months for pessary cleaning and vaginal inspection[7]. In case women performed self-management, the frequency of cleaning was left to their personal preference, however it was advised to clean their pessary at least every 4 months. Women were instructed to return to the hospital if they experienced any complaint or adverse events due to pessary therapy[7].

Surgery

Surgical intervention included a range of surgical procedures for the correction of three main types of prolapse that can occur individually or simultaneously, namely, 1) uterine descent 2) cystocele, and/or 3) rectocele[7]. For a cystocele or rectocele, respectively a conventional anterior- or posterior colporrhaphy was the standard technique. For a uterine descent, uterine preserving techniques or a vaginal hysterectomy was performed[7]. All surgical interventions were performed following Dutch guidelines recommendations[7,17]. Decisions on which surgical technique was performed was decided in a shared-decision manner between gynecologist and participant[7]. Women were instructed to return to the hospital if they experienced any complaint or adverse events.

For peer review only - http://bmjopen.bmj.com/site/about/guidelines.xhtml

For peer review only

Study perspective, time horizon, and discount rate

This economic evaluation was conducted from a healthcare and a societal perspective over a time horizon of 24 months. The healthcare perspective included costs related to interventions (pessary therapy and surgery) and healthcare utilization costs. The societal perspective included costs related to absenteeism from paid work in addition to the interventions' costs and healthcare utilization costs. Discount rates of 1.5% and 4% were applied to QALY and costs, respectively after the first year of the RCT as recommended by the Dutch Guideline for Economic Evaluations in healthcare[18].

For peer review only

Outcomes

Health outcomes

Two health outcomes were used for the trial-based economic evaluation: patient-reported subjective improvement and Quality-Adjusted Life-Years (QALYs). Subjective improvement was measured with the Patient Global Impression of Improvement (PGI-I)[19] scale at 12- and 24-month follow-up. The PGI-I is a single question, seven-point Likert response scale ranging from '*very much worse*' to '*very much better*'[19]. Subjective improvement was defined as a response of '*much better*' or '*very much better*'[20]. The PGI-I is a validated, easy to apply questionnaire, and it strongly correlates with other validated outcome measures such as the POP-Q system[14,19].

The QALY incorporates the impact of interventions on both the quantity and quality of life[21]. It is a routinely used health outcome measure in economic evaluations because it allows decision-makers to compare the cost-effectiveness of a range of interventions for different health conditions[21]. In this study, QALYs were calculated based on the EQ-5D-3L data collected at baseline, 3-, 6-, 12-, and 24-month follow-up. The EQ-5D-3L includes five dimensions of quality of life (i.e., mobility, self-care, usual activities, pain/discomfort and anxiety/depression) with three response levels (i.e., no problems, some problems or extreme problems/ unable to) describing 243 health states[22]. The participants' health states obtained from EQ-5D-3L responses were converted into utility values using the Dutch tariff[23]. The utility values were used to calculate QALYs by means of linear interpolation (i.e., the duration of a health state is multiplied by the utility related to that health state)[24].

For peer review only - http://bmjopen.bmj.com/site/about/guidelines.xhtml

Cost outcomes

Intervention costs

Intervention costs of the pessary therapy included those related to the pessary device and one gynecologist consultation for the pessary placement at baseline. Unit prices of pessary therapy were based on the Dutch costing guideline[25] and on market prices (Supplementary Table 1). For the surgery group, intervention costs consisted of the surgical procedures conducted at baseline. Unit prices of surgical procedures was based on the Diagnosis Treatment Combination (in Dutch *Diagnose Behandeling Combinatie*, DBC)[26]. The DBC is a care path that includes diagnostic procedures and care activities delivered at hospital and immediate follow-up up to 6 weeks (42 days)[26]. The average national prices are calculated for each DBC code based on all declared reimbursements that have been submitted to the DBC Information System (DIS) by healthcare providers in hospital care. A detailed description of the resources used in the interventions and their respective unit costs is presented in Supplementary Table 1.

Healthcare utilization costs

Healthcare utilization was collected during follow-up visits at hospital centers including information on the number of scheduled consultations with gynecologists and extra consultations due to complications, the number of days of hospital readmissions due to complications, the type/number of surgeries after pessary, the type/number of re-surgeries, the number of times a pessary device was changed, and the use of a pessary after initial surgery. Additionally, an adapted version of the iMTA Medical Consumption Questionnaire (iMCQ)[27] was used to measure non-intervention related healthcare utilization at 3-, 6-, 12-, and 24-month follow-up. Healthcare utilization included resources used in primary care (i.e., the number of GP consultations and other healthcare professionals due to POP complaints), and in secondary care apart from study scheduled consultations (i.e., the number of extra consultations with other medical specialists due to POP complaints). The number of healthcare resources used was then multiplied by their respective unit prices. Unit of prices of healthcare resources were based on the Dutch costing guideline[25] (Supplementary Table 1).

For peer review only - http://bmjopen.bmj.com/site/about/guidelines.xhtml

Lost productivity costs

Absenteeism from paid work due to POP symptoms was measured using a adapted version of the iMTA Productivity Cost Questionnaire[28] at 3-, 6-, 12-, and 24-month follow-up. The friction cost approach (FCA) was used to calculate sickness absenteeism costs related to paid work[29]. The FCA assumes that sickness absenteeism costs are limited to the period needed to replace an absent, sick worker (the friction period), which has been estimated to be 12 weeks (85 days) in the Netherlands[29]. Gender-specific estimates of the mean wages of the Dutch population were used to calculate sickness absenteeism costs from paid work[25]. All costs were indexed to 2022 using the consumer price index in the Netherlands(www.cbs.nl)[30].

Cost-effectiveness analysis

Analyses were performed according to the intention-to-treat principle using StataSE V.17. As recommended by Faria et al,[31] mean imputation was used to impute missing values at baseline (i.e., parity, Patient Global Impression of Severity [PGIS], Pelvic Floor Distress Inventory [PFDI-20], Pelvic Organ Prolapse Distress Inventory [POPDI-6], Colorectal-Anal Distress Inventory [CRADI-8], Urinary Distress Inventory [UDI-6], and EQ-5D utility values). Subsequently, multiple imputation by Chained Equations (MICE) was used to impute follow-up missing data. The multiple imputation model included treatment group and hospital center, variables associated with missingness (i.e., Body Mass Index [BMI], number of re-surgeries, number of consultations, and family history of prolapse), outcomes, and potential confounders (i.e., age, history of gynecological operations, prolapse stage, menopausal state, and risk-increasing aspects)[32]. Risk-increasing aspects was a combined variable that included at least one of the following comorbidities: smoking status, antidepressants use, obesity, diabetes mellitus, and chronic pulmonary disease. Predictive Mean Matching was used in the imputation procedure to account for the skewed distribution of the costs[33]. Missing cost data were imputed at the level of resource use by time point (i.e., number of consultations, working hours and absenteeism hours). The number of imputations was increased until there was a loss of efficiency of ≤5%, resulting in ten imputed datasets[34]. The ten imputed datasets were analyzed separately and estimates were pooled using Rubin's rules[35].

Multilevel regression models were used to estimate the difference in costs and effects between the groups to account for the fact that randomization was stratified by hospital center[36]. For cost and effect outcomes, a

For peer review only - http://bmjopen.bmj.com/site/about/guidelines.xhtml

two-level structure was used where participants and hospital center represented the first and second level, respectively. All analysis models were adjusted for relevant confounders. The PGI-I model was adjusted for PGI-I at 12-month, risk-increasing aspects, and prolapse stage. The QALY model was adjusted for baseline utility values[37], PGI-I at 12-month, risk-increasing aspects, prolapse stage, and number of extra consultations due to complications. Healthcare and societal costs models were adjusted for age, PGI-I at 24-month, menopause state, risk-increasing aspects, and prolapse stage. A non-inferiority margin of 10% risk difference (one-sided 95% CI) was set for the PGI-I outcome based on the expectation that 80% of women would report successful treatment (either pessary therapy or surgery) after 2 years[12,38,39].

Bias-corrected accelerated bootstrapping with 5000 replications was used to estimate the joint uncertainty surrounding differences in costs and effects. Bootstrapped cost-effect pairs were plotted on cost-effectiveness planes (CE-planes)[40]. Non-inferiority with regard to cost-effectiveness was demonstrated using a one-sided α of 2.5%, meaning that 97.5% of the cost-effect pairs have to lie right of the non-inferiority margin for effects[41]. Cost-effectiveness acceptability curves (CEACs) were estimated to show the probability of the pessary therapy being cost-effective compared to surgery for a range of willingness-to-pay (WTP) thresholds (i.e., the maximum amount of money the healthcare system is willing to pay for a unit of effect gained)[42]. For QALY, we used a WTP threshold of 20000 €/QALY gained recommended by the Dutch Health Care Institute[43]. As there is no specific WTP threshold for PGI-I, we used a maximum WTP of €5237 per PGI-I gained. This threshold was based on the average DBC costs of surgical procedures performed for POP symptoms as reported in Supplementary Table 1.

Sensitivity Analysis

Two sensitive analyses (SA) were performed to assess the robustness of the results. SA1 was a complete case analysis, meaning that only observations with complete data were included in the main analysis. Because we expected some participants to crossover from pessary to surgery, a per protocol analysis (SA2) was performed to compare treatment groups including women who completed the treatment to which they were originally allocated.

For peer review only - http://bmjopen.bmj.com/site/about/guidelines.xhtml

Patient and Public Involvement

One major gynecological patient organization in the Netherlands (i.e., BekkenBodem4All) as well as the Dutch Urogynecology Consortium fully agreed on the study protocol and identified the study as highly relevant[12].

For peer review only

For peer review only - http://bmjopen.bmj.com/site/about/guidelines.xhtml

RESULTS

Participants

Of the 1605 women assessed for eligibility, 440 were randomized to either pessary therapy (n=218) or surgery (n=222) as shown in Figure 1. After randomization, one participant was excluded from the surgery group due to prolapse stage 1 resulting in a total of 221 women in this group (Supplementary Figure 1). Baseline incomplete data were imputed for parity (n=4, 0.9%), PFDI-20 (n=22, 5.0%), POPDI-6 (n=21, 4.8%%), CRADI-8 (n=21, 4.8%), UDI-6 (n=22, 5.0%) and utility values (n=24, 5.5%) (Table 1). Follow-up missing data at 24-months were multiply imputed for PGI-I (n=104, 23.7%), QALY (n=144, 32.8%), healthcare costs (n=160, 36.4%), and societal costs (n=165, 37.6%) (Figure 1). A total of 118 of 218 (54.1%) women randomized to pessary therapy crossed over to surgery, and a total of 8 women out of 221 (3.6%) underwent recurrent surgery. At baseline, no meaningful differences were found between both groups (Table 1).

< Insert Table 1 here >

Effectiveness

In the unadjusted analysis, the lower 95%CI bound of the PGI-I outcome surpassed the non-inferiority margin of -10% (mean difference -0.06, 95% CI, -0.15; 0.04), meaning that non-inferiority of pessary therapy compared to surgery could not be shown (Table 2). After adjusting for confounders, the lower 95% CI bound of the PGI-I outcome still surpassed the non-inferiority margin (mean difference -0.03, 95% CI, -0.11; 0.06, Table 3). There was no statistically significant difference in QALYs between groups neither in the unadjusted analysis (mean difference -0.02, 95% CI, -0.06; 0.02, Table 2) nor the adjusted analysis (mean difference -0.01, 95% CI -0.05; 0.03, Table 3).

< Insert Table 2 here >

For peer review only - http://bmjopen.bmj.com/site/about/guidelines.xhtml

Costs

After 24 months, unadjusted analyses showed there were statistically significant savings in the pessary therapy group compared to the surgery for both total healthcare costs (mean difference -€1850, 95% CI, -€2228; -€1476) and societal costs (mean difference -€1878, 95% CI, -€2395; -€1345) (Table 2). The main cost driver in the surgery group was the intervention costs (€4640, SE=0), while in the pessary therapy group this was secondary costs (€3736, SE=174) (Table 2). Given that half of patients in the pessary group crossed over to surgery (54.1%) and a small proportion of women underwent recurrent surgery in the surgery group (3.6%), secondary costs during follow-up were statistically significantly higher in the pessary therapy group compared to surgery (mean difference €2609, 95% CI, €2232; €2982, Table 2). In the adjusted analysis, mean differences in healthcare and societal costs between groups slightly decreased compared to the unadjusted analysis (Table 3). However, both healthcare and societal costs in the pessary group were still statistically significantly lower than in the surgery group.

< Insert Table 3 here >

Cost-effectiveness analysis

For the PGI-I outcome, from both perspectives, the main analysis showed that most bootstrapped cost-effect pairs were situated on the right of the non-inferiority margin for effects (95.5%) (Figure 1[1A] and [2A]). Due to statistically significant lower healthcare and societal costs in the pessary therapy group compared to surgery, the probability of the pessary therapy being cost-effective compared to surgery was 1 at different WTP per an additional participant that improved (Figure 1 [1B] and [2B]). This means that the pessary therapy as an initial treatment option has a 100% probability of being cost-effective compared to immediate surgery.

< Insert Figure 1 here >

For QALYs, the majority of the bootstrapped cost-effect pairs was in the South-West quadrant of the CE-plane (70%) meaning that on average the pessary was less costly but less effective in terms of QALY gained (Figure 2 [1A] and [2A]). The probability that pessary therapy is cost-effective compared to surgery at all WTP thresholds was 1 from both perspectives (Figure 2 [1B] and [2B]).

For peer review only - http://bmjopen.bmj.com/site/about/guidelines.xhtml

< Insert Figure 2 here >

Sensitivity analysis

SA1 including only complete cases showed similar results compared to the main analysis, although the direction of the difference in QALYs turned around (Table 3). However, the difference was still small and neither statistically significant nor clinically relevant. This explains the negative ICER and the shift in the distribution of the bootstrapped cost-effect pairs between South-West and South-East quadrants of the CE-plane compared to the main analysis. In SA2, which included women that received their originally allocated intervention with fully imputed data on the PGI-I, (pessary therapy n=81, surgery n=190), the differences in costs and PGI-I between pessary and surgery increased and in QALY decreased compared to the main analysis (Table 3). However, this did not affect the cost-effectiveness results.

For peer review only

For peer review only - http://bmjopen.bmj.com/site/about/guidelines.xhtml

DISCUSSION

Main findings

This economic evaluation showed that although non-inferiority of pessary therapy with regard to subjective improvement could not be shown and there were no statistically significant differences in QALY gained, a strategy of initial pessary therapy in women with symptomatic POP is likely to be cost-effective compared to immediate surgery from a healthcare and a societal perspective. These findings were confirmed by sensitivity analyses.

Explanation of the findings and comparison with the literature

For both effect outcomes, the high probability of pessary therapy being cost-effective compared to surgery is explained by the fact that total healthcare and societal costs in the pessary group were statistically significant lower than in the surgery group, despite the high proportion of crossover (54.1%) from participants in the pessary group to surgery.

Recently, Bugge et al. (2022)[8] systematically reviewed the (cost-)effectiveness of pessary therapy for managing POP symptoms and found only two economic evaluations[11,44]. Of those, only Hullfish et al. (2011)[11] directly compared pessary therapy with surgery. They developed a model-based economic evaluation with 12-month follow-up based on data from the literature, local experience of a single institution, and expert opinion. Results showed that for lower WTP thresholds pessary is cost-effective compared to surgery and for higher WTP thresholds not anymore. Our results, based on randomized data, showed that pessary therapy is cost-effective compared to surgery at all WTP thresholds.

Strengths and Limitations

One of the strengths of this study is that it was performed alongside a multicenter pragmatic randomized controlled trial. The randomization process ensures that groups are comparable and decrease the likelihood of selection bias[45] while the multicenter pragmatic design improves generalizability of results and transferability to clinical practice. Validated outcome measures were used and the trial had a long-term follow-up of 2 years. This study has a number of limitations. First, productivity costs related to unpaid work such as number of

For peer review only - http://bmjopen.bmj.com/site/about/guidelines.xhtml

hours spent in unpaid activities (e.g., voluntary and housework) and informal care (e.g., care provided by family and friends while being sick) were not collected. Since the mean age of the participants is 65 years (the retirement age in the Netherlands until 2024), these costs are likely to be more relevant than lost productivity related to paid work. Second, consultations related to both interventions were provided by gynecologists, which may overestimate intervention costs, as these consultations may be provided by trained GPs at lower costs (i.e., €39 by a GP vs €109 by a medical specialist). Third, healthcare resource utilization related to the specific medical treatment of complications (e.g., medications) was not collected. Only costs related to readmissions and extra complications due to complications were included in the analysis. This may underestimate healthcare utilization costs. Fourth, the proportion of missing data on the outcomes was between 24 to 38%. To deal with this issue, multiple imputation of missing values were performed which is the recommended method to handle missing data in trial-based economic evaluations to produce valid estimates[31,46,47]. In addition, a sensitivity analysis including complete cases was performed to evaluate the robustness of findings, showing that results were not affected. Fifth, costs were estimated based on the Dutch reimbursement system and can differ from countries which may hamper the generalizability of results to healthcare systems in other countries.

Implications for practice and future research

A considerable number of women declined to participate in the RCT (n=553, Figure 1). These women were offered the possibility to participate in a prospective cohort[9]. The majority of participants in the prospective cohort opted for a pessary therapy as initial treatment option (62.2%)[9]. Compared to participants of the RCT[7], participants in the cohort less often crossed over to surgery (24% vs 54%). In addition, in this cohort, more women reported successful improvement after surgery compared to pessary[9]. This suggests that it is important to consider women's preferences when deciding about the most suitable treatment for their POP symptoms. Future studies should measure costs from a broader perspective than this study did, as relevant costs were not considered in the analysis, that is, costs related to follow-up medical treatment, informal care costs and lost productivity costs related to unpaid work (e.g., housework, voluntary work).

For peer review only - http://bmjopen.bmj.com/site/about/guidelines.xhtml

CONCLUSION

Non-inferiority of pessary therapy with regard to the PGI-I could not be shown and there were no statistically significant differences in QALYs between interventions. Due to significantly lower costs, pessary therapy is likely to be cost-effective compared to surgery from a healthcare and a societal perspective as an initial treatment option for women with moderate to severe POP symptoms treated in secondary care compared to immediate surgery. However, considering the high crossover rate from pessary to surgery it is important to consider women's preferences regarding the treatment of their POP systems.

For peer review only

For peer review only - http://bmjopen.bmj.com/site/about/guidelines.xhtml

REFERENCES

1. Aboseif C, Liu P. *Pelvic Organ Prolapse*. StatPearls Publishing; 2022. Accessed August 22, 2022. https://www.ncbi.nlm.nih.gov/books/NBK563229/

2. Harrison BP, Cespedes RD. PELVIC ORGAN PROLAPSE*. *Emergency Medicine Clinics of North America*. 2001;19(3):781-798. doi:10.1016/S0733-8627(05)70215-7

3. Doaee M, Moradi-Lakeh M, Nourmohammadi A, Razavi-Ratki SK, Nojomi M. Management of pelvic organ prolapse and quality of life: a systematic review and meta-analysis. *Int Urogynecol J*. 2014;25(2):153-163. doi:10.1007/s00192-013-2141-8

4. Dieter AA, Wilkins MF, Wu JM. Epidemiological trends and future care needs for pelvic floor disorders. *Current Opinion in Obstetrics and Gynecology*. 2015;27(5):380-384. doi:10.1097/GCO.0000000000000200

5. Brown HW, Hegde A, Huebner M, et al. International urogynecology consultation chapter 1 committee 2: Epidemiology of pelvic organ prolapse: prevalence, incidence, natural history, and service needs. *Int Urogynecol J*. 2022;33(2):173-187. doi:10.1007/s00192-021-05018-z

6. van Geelen JM, Dwyer PL. Where to for pelvic organ prolapse treatment after the FDA pronouncements? A systematic review of the recent literature. *Int Urogynecol J*. 2013;24(5):707-718. doi:10.1007/s00192-012-2025-3

7. van der Vaart LR, Vollebregt A, Milani AL, et al. Effect of Pessary vs Surgery on Patient-Reported Improvement in Patients With Symptomatic Pelvic Organ Prolapse: A Randomized Clinical Trial. *JAMA*. 2022;328(23):2312-2323. doi:10.1001/jama.2022.22385

8. Bugge C, Adams EJ, Gopinath D, et al. Pessaries (mechanical devices) for managing pelvic organ prolapse in women. *Cochrane Database of Systematic Reviews*. 2020;(11). doi:10.1002/14651858.CD004010.pub4

9. van der Vaart L, Vollebregt A, Milani A, et al. Pessary or surgery for a symptomatic pelvic organ prolapse: the PEOPLE study, a multicentre prospective cohort study. *BJOG*. 2022;129(5):820-829. doi:10.1111/1471-0528.16950

10. de Albuquerque Coelho SC, de Castro EB, Juliato CRT. Female pelvic organ prolapse using pessaries: systematic review. *Int Urogynecol J*. 2016;27(12):1797-1803. doi:10.1007/s00192-016-2991-y

11. Hullfish KL, Trowbridge ER, Stukenborg GJ. Treatment strategies for pelvic organ prolapse: a cost-effectiveness analysis. *Int Urogynecol J*. 2011;22(5):507-515. doi:10.1007/s00192-011-1383-6

12. PEOPLE group. Healthcare evaluation in the Netherlands. - PEOPLE Project. Published 2015. Accessed August 11, 2022. https://www.zorgevaluatienederland.nl/evaluations/people

13. Husereau D, Drummond M, Augustovski F, et al. Consolidated Health Economic Evaluation Reporting Standards 2022 (CHEERS 2022) Statement: Updated Reporting Guidance for Health Economic Evaluations. *Value in Health*. 2022;25(1):3-9. doi:10.1016/j.jval.2021.11.1351

14. Bump RC, Mattiasson A, Bø K, et al. The standardization of terminology of female pelvic organ prolapse and pelvic floor dysfunction. *American Journal of Obstetrics and Gynecology*. 1996;175(1):10-17. doi:10.1016/S0002-9378(96)70243-0

15. Shumaker SA, Wyman JF, Uebersax JS, McClish D, Fantl JA. Health-related quality of life measures for women with urinary incontinence: the Incontinence Impact Questionnaire and the Urogenital Distress Inventory. Continence Program in Women (CPW) Research Group. *Qual Life Res*. 1994;3(5):291-306. doi:10.1007/BF00451721

For peer review only - http://bmjopen.bmj.com/site/about/guidelines.xhtml

16. Cundiff GW, Amundsen CL, Bent AE, et al. The PESSRI study: symptom relief outcomes of a randomized crossover trial of the ring and Gellhorn pessaries. *American Journal of Obstetrics and Gynecology*. 2007;196(4):405.e1-405.e8. doi:10.1016/j.ajog.2007.02.018

17. Federatie Medisch Specialisten. Prolapse: guideline about the best care for patients with prolapse according to current standards. Utrecht, the Netherlands. Published 2014. Accessed March 22, 2023. https://richtlijnendatabase.nl/richtlijn/prolaps/prolaps_-_startpagina.html

18. VWS. Guideline for economic evaluations in healthcare - Dutch National Health Care Institute - Ministerie van Volksgezondheid, Welzijn en Sport. Published June 16, 2016. Accessed August 23, 2018. https://english.zorginstituutnederland.nl/publications/reports/2016/06/16/guideline-for-economic-evaluations-in-healthcare

19. Srikrishna S, Robinson D, Cardozo L. Validation of the Patient Global Impression of Improvement (PGI-I) for urogenital prolapse. *Int Urogynecol J*. 2010;21(5):523-528. doi:10.1007/s00192-009-1069-5

20. Labrie J, Berghmans BLCM, Fischer K, et al. Surgery versus Physiotherapy for Stress Urinary Incontinence. *N Engl J Med*. 2013;369(12):1124-1133. doi:10.1056/NEJMoa1210627

21. Drummond MF, Sculpher MJ, Torranc GW. *Methods for the Economic Evaluation of Health Care Programmes*. 3rd ed. Oxford University Press; 2005.

22. EuroQol Group. EuroQol - a new facility for the measurement of health-related quality of life. *Health Policy*. 1990;16(3):199-208. doi:10.1016/0168-8510(90)90421-9

23. Lamers LM, McDonnell J, Stalmeier PFM, Krabbe PFM, Busschbach JJV. The Dutch tariff: results and arguments for an effective design for national EQ-5D valuation studies. *Health Economics*. 2006;15(10):1121-1132. doi:10.1002/hec.1124

24. Whitehead SJ, Ali S. Health outcomes in economic evaluation: the QALY and utilities. *Br Med Bull*. 2010;96(1):5-21. doi:10.1093/bmb/ldq033

25. Kanters TA, Bouwmans CAM, van der Linden N, Tan SS, Hakkaart-van Roijen L. Update of the Dutch manual for costing studies in health care. *PLoS One*. 2017;12(11). doi:10.1371/journal.pone.0187477

26. Diagnose Behandeling Combinatie (DBC) open data - NZa. Accessed September 3, 2022. https://www.opendisdata.nl/

27. iMTA. iMTA Medical Consumption Questionnaire. iMTA. Accessed February 6, 2022. https://www.imta.nl/questionnaires/imcq/

28. Bouwmans C, Krol M, Severens H, Koopmanschap M, Brouwer W, Roijen LH van. The iMTA Productivity Cost Questionnaire: A Standardized Instrument for Measuring and Valuing Health-Related Productivity Losses. *Value in Health*. 2015;18(6):753-758. doi:10.1016/j.jval.2015.05.009

29. Koopmanschap MA, Rutten FFH, van Ineveld BM, van Roijen L. The friction cost method for measuring indirect costs of disease. *Journal of Health Economics*. 1995;14(2):171-189. doi:10.1016/0167-6296(94)00044-5

30. CBS. StatLine - Consumentenprijzen. Accessed August 21, 2022. https://opendata.cbs.nl/#/CBS/nl/dataset/83131NED/table?ts=1660927150933

31. Faria R, Gomes M, Epstein D, White IR. A Guide to Handling Missing Data in Cost-Effectiveness Analysis Conducted Within Randomised Controlled Trials. *Pharmacoeconomics*. 2014;32(12):1157. doi:10.1007/s40273-014-0193-3

For peer review only - http://bmjopen.bmj.com/site/about/guidelines.xhtml

32. Schulten SFM, Claas-Quax MJ, Weemhoff M, et al. Risk factors for primary pelvic organ prolapse and prolapse recurrence: an updated systematic review and meta-analysis. *American Journal of Obstetrics and Gynecology*. 2022;227(2):192-208. doi:10.1016/j.ajog.2022.04.046

33. White IR, Royston P, Wood AM. Multiple imputation using chained equations: Issues and guidance for practice. *Statistics in Medicine*. 2011;30(4):377-399. doi:10.1002/sim.4067

34. Madley-Dowd P, Hughes R, Tilling K, Heron J. The proportion of missing data should not be used to guide decisions on multiple imputation. *Journal of Clinical Epidemiology*. 2019;110:63-73. doi:10.1016/j.jclinepi.2019.02.016

35. Little RJA, Rubin DB. *Statistical Analysis with Missing Data*. John Wiley & Sons, Inc.; 2014.

36. Gomes M, Grieve R, Nixon R, Edmunds WJ. Statistical Methods for Cost-Effectiveness Analyses That Use Data from Cluster Randomized Trials: A Systematic Review and Checklist for Critical Appraisal. *Med Decis Making*. 2012;32(1):209-220. doi:10.1177/0272989X11407341

37. Manca A, Hawkins N, Sculpher MJ. Estimating mean QALYs in trial-based cost-effectiveness analysis: the importance of controlling for baseline utility. *Health Economics*. 2005;14(5):487-496. doi:10.1002/hec.944

38. Koullali B, van Kempen LEM, van Zijl MD, et al. A multi-centre, non-inferiority, randomised controlled trial to compare a cervical pessary with a cervical cerclage in the prevention of preterm delivery in women with short cervical length and a history of preterm birth – PC study. *BMC Pregnancy Childbirth*. 2017;17:215. doi:10.1186/s12884-017-1393-6

39. Non-Inferiority Clinical Trials to Establish Effectiveness Guidance for Industry. *US Department of Health and Human Services Food and Drug Administration Center for Drug Evaluation and Research (CDER) Center for Biologics Evaluation and Research (CBER)*.

40. Black WC. The CE plane: a graphic representation of cost-effectiveness. *Med Decis Making*. 1990;10(3):212-214. doi:10.1177/0272989X9001000308

41. Bosmans JE, de Bruijne MC, van Hout HPJ, Hermens MLM, Adèr HJ, van Tulder MW. Practical guidelines for economic evaluations alongside equivalence trials. *Value Health*. 2008;11(2):251-258. doi:10.1111/j.1524-4733.2007.00245.x

42. Fenwick E, Claxton K, Sculpher M. Representing uncertainty: the role of cost-effectiveness acceptability curves. *Health Econ*. 2001;10(8):779-787. doi:10.1002/hec.635

43. ZIN. Zorginstituut Nederland - Richtlijn voor het uitvoeren van economische evaluaties in de gezondheidszorg. Ministerie van Volksgezondheid, Welzijn en Sport. Published February 29, 2016. Accessed March 28, 2018. https://www.zorginstituutnederland.nl/publicaties/publicatie/2016/02/29/richtlijn-voor-het-uitvoeren-van-economische-evaluaties-in-de-gezondheidszorg

44. Panman C, Wiegersma M, Kollen B, et al. Two-year effects and cost-effectiveness of pelvic floor muscle training in mild pelvic organ prolapse: a randomised controlled trial in primary care. *BJOG: An International Journal of Obstetrics & Gynaecology*. 2017;124(3):511-520. doi:10.1111/1471-0528.13992

45. Randomization-Based Evaluations. In: *Multiple Imputation for Nonresponse in Surveys*. Wiley-Blackwell; 2008:113-153. doi:10.1002/9780470316696.ch4

46. MacNeil Vroomen J, Eekhout I, Dijkgraaf MG, et al. Multiple imputation strategies for zero-inflated cost data in economic evaluations: which method works best? *Eur J Health Econ*. 2016;17(8):939-950. doi:10.1007/s10198-015-0734-5

For peer review only - http://bmjopen.bmj.com/site/about/guidelines.xhtml

47. Ben ÂJ, van Dongen JM, Alili ME, et al. The handling of missing data in trial-based economic evaluations: should data be multiply imputed prior to longitudinal linear mixed-model analyses? *Eur J Health Econ*. Published online September 26, 2022. doi:10.1007/s10198-022-01525-y

Authors' contributions: **Ângela Jornada Ben:** Methodology, Data Curation, Formal analysis, and Writing - Original Draft. **Lisa R van der Vaart:** Methodology, Data Curation, Writing - Review & Editing, and Project administration. **Judith E. Bosmans:** Methodology, Writing - Review & Editing, Supervision. **Jan-Paul W.R. Roovers:** Writing - Review & Editing. **Antoine L. Lagro-Janssen:** Writing - Review & Editing. **Carl H. van der Vaart**: Conceptualization, Methodology, Resources, Funding acquisition, and Writing - Review & Editing. **Astrid Vollebregt**: Conceptualization, Methodology, and Writing - Review & Editing, Supervision. All authors have agreed to the submission of the final paper.

Acknowledgments: PEOPLE group: Milani, F, van Bavel, J., Bon G.G., Bongers M.Y., Bos K., Broekman A.M.W., Dietz, V., van Eijndhoven H.W.F., Hakvoort R.A., Janszen E.W.M., Kluivers K., Link G., Massop-Helmink D.S., van der Ploeg J.M., Sikkema F.M., Spaans W.A., van der Ster A., Verheijen-van de Waarsenburg, K., Vernooij M.M.A., Weemhoff, M., A.E. Weis-Potters.

For peer review only

TABLE 1. BASELINE CHARACTERISTICS OF PARTICIPANTS

**Baseline characteristic**	**Pessary therapy** ***n = 218***	**Surgery** ***n = 221***
Age (mean (SD))	64.8 (9.5), n=218	64.7(9.2), n=221
Risk-increasing aspects [¥] (n, %)	71 (32.6), n=221	58 (26.2), n=218
History of gynecological surgery (n, %)	22 (10.1), n=218	28 (12.7), n=221
Family history of prolapse (n, %)	106 (48.6), n=218	107 (49.5), n=216
Parity (median (IQR)	2.0 (2-3), n=215	2.0 (2-3), n=220
Postmenopausal (n, %)	186 (92.5), n=201	185 (90.2), n=205
Duration of symptoms in months (median (IQR)	6 (2-24), n=211	6 (3-24), n=216
Vaginal atrophy (n, %)	106 (56.7), n=187	110 (57.3), n=192
Prolapse stage (n, %)		
II (Moderate)	85 (39.0), n=218	102 (46.2), n=221
≥III (Severe)	133 (61.0), n=218	119 (53.9), n=221
PGI-S score[a] (n, %)		
I (Not severe)	13 (6.3), n=205	9 (4.4), n=205
II (Mild)	48 (23.4), n=205	50 (24.4), n=205
III (Moderate)	99 (48.3), n=205	112 (54.6), n=205
IV (Severe)	45 (22.0), n=205	34 (16.6), n=205
PFDI-20 score[b] (n, %)		
POPDI-6 score	29.5 (19.2), n=210	28.7 (15.6), n=208
CRADI-8 score	13.9 (15.1), n=210	12.1 (12.6), n=208
UDI-6 score	26.0 (22.0), n=209	25.2 (20.0), n=208
PFDI-20 total score	69.3 (45.7), n=209	65.9 (37.7), n=208
EQ-5D utility value[c] (mean (SD))	0.87 (0.15), n=209	0.85 (0.15), n=206

SD = standard deviation. n = number of women. % = proportion. IQR = interquartile range. [a]PGIS = Patient Global Impression of Severity: I (not severe), II (mild), III (moderate), IV (severe). [b]PFDI-20 = Pelvic Floor Distress Inventory: the subscale scores range from 0-100 and the total score ranges from 0 to 300. Higher scores indicate more symptom distress. POPDI-6 = Pelvic Organ Prolapse Distress Inventory. CRADI-8 = Colorectal-Anal Distress Inventory. UDI-6 = Urinary Distress Inventory. [c]EQ-5D utility values: the Dutch EQ-5D tariffs range from -0.33 to 1. [¥]presence of 1 or more comorbidities: smoking, use of antidepressants, obesity, diabetes mellitus, chronic pulmonary disease.

For peer review only - http://bmjopen.bmj.com/site/about/guidelines.xhtml

TABLE 2. EFFECTS AND COSTS BY TREATMENT GROUP AND DIFFERENCE AT 24-MONTH FOLLOW-UP

	**Pessary therapy** *n = 218*	**Surgery** *n = 221*	**Unadjusted Difference (95% CI)**
**Effects**			
PGI-I, n (%)	164 (75.1%)	179 (80.8%)	-0.06 (-0.15; 0.04)
QALY, mean (SE)	1.80 (0.02)	1.82 (0.01)	-0.02 (-0.06; 0.02)
**Costs, mean (SE)**			
Intervention costs	178 (0.2)	4640 (0)	-4462 (-4463; -4462)
Primary care costs	18 (2)	15 (2)	3 (-3; 8)
Secondary care costs	3736 (174)	1127 (80)	2609 (2232; 2982)
Healthcare costs	3932 (174)	5782 (80)	-1850 (-2228; -1476)
Absenteeism from paid work	362 (117)	390 (120)	-28 (-338; 290)
Societal costs	4294 (227)	6172 (150)	-1878 (-2395, -1345)

PGI-I = Patient Global Impression of Improvement (1=improvement; 0= no improvement). n = number of participants. % = proportion. SE = standard error. Intervention costs in the pessary group = costs of pessary device and pessary placement consultation at baseline. Intervention costs in the surgery group = DBC costs of surgery at baseline which included one follow-up consultation at 6 weeks. Primary care costs = costs of general practitioner or other healthcare professional consultations apart from the pre-scheduled follow-up consultations because of complaints related to pelvic organ prolapse (POP) symptoms. Secondary care costs = costs of follow-up scheduled consultations with gynecologists attended by patients and extra consultations due to complications, costs of hospital readmissions due to complications, surgeries after pessary, re-surgeries, and costs of pessary change.

For peer review only - http://bmjopen.bmj.com/site/about/guidelines.xhtml

TABLE 3. RESULTS OF THE COST-EFFECTIVENESS AND COST-UTILITY ANALYSIS

Effect outcome	ΔE (95% CI)	ΔC (95% CI)	ICER	Cost-effectiveness plane			
				NE	SE	SW	NW
**Main analysis – Healthcare Perspective**							
PGI-I, n=439	-0.03 (-0.11; 0.06)	-1780 (-2148; -1422)	65525	0%	24%	76%	0%
QALY, n=439	-0.01 (-0.05; 0.03)	-1780 (-2148; -1422)	154939	0%	30%	70%	0%
**Main analysis – Societal Perspective**							
PGI-I, n=439	-0.03 (-0.11; 0.06)	-1826 (-2328; -1322)	67203	0%	24%	76%	0%
QALY, n=439	-0.01 (-0.05; 0.03)	-1826 (-2328; -1322)	158905	0%	30%	70%	0%
**Sensitivity analysis 1 – Complete Case Analysis – Healthcare Perspective**							
PGI-I, n=259	-0.01 (-0.09; 0.08)	-1961 (-2453; -1585)	283377	0%	38%	62%	0%
QALY, n=256	0.02 (-0.03; 0.06)	-1947 (-2450; -1571)	-119365	0%	79%	21%	0%
**Sensitivity analysis 1 – Complete Case Analysis – Societal Perspective**							
PGI-I, n=254	-0.005 (-0.08; 0.09)	-1872 (-2479; -1243)	389260	0%	38%	62%	0%
QALY, n=252	0.02 (-0.03; 0.06)	-1846 (-2475; -1224)	-99342	0%	81%	19%	0%
**Sensitivity analysis 2 – Per Protocol Analysis – Healthcare Perspective**							
PGI-I, n=271	-0.06 (-0.18; 0.05)	-4413 (-4597; -4326)	69585	0%	12%	88%	0%
QALY, n=271	-0.0001 (-0.04; 0.04)	-4413 (-4597; -4326)	41586588	0%	53%	47%	0%
**Sensitivity analysis 2 – Per Protocol Analysis – Societal Perspective**							
PGI-I, n=271	-0.06 (-0.18; 0.05)	-4772 (-5236; -4495)	75249	0%	12%	88%	0%
QALY, n=271	-0.0001 (-0.04; 0.04)	-4772 (-5236; -4495)	22594796	0%	53%	47%	0%

ΔC= difference in costs; 95% CI = 95% confidence interval; ΔE= difference in effects; ICER = Incremental Cost-Effectiveness Ratio; NE = northeast; SE = southeast; SW = southwest; NW = northwest. The PGI-I model was adjusted by PGI-I at 12-month, risk-increasing aspects, and prolapse stage. The QALY model was adjusted by baseline utility values, PGI-I at 12-month, risk-increasing aspects, prolapse stage, and number of extra consultations due complications. Healthcare and societal costs models were adjusted by age, PGI-I at 24-month, menopause state, risk-increasing aspects, and prolapse stage.

For peer review only

For peer review only - http://bmjopen.bmj.com/site/about/guidelines.xhtml

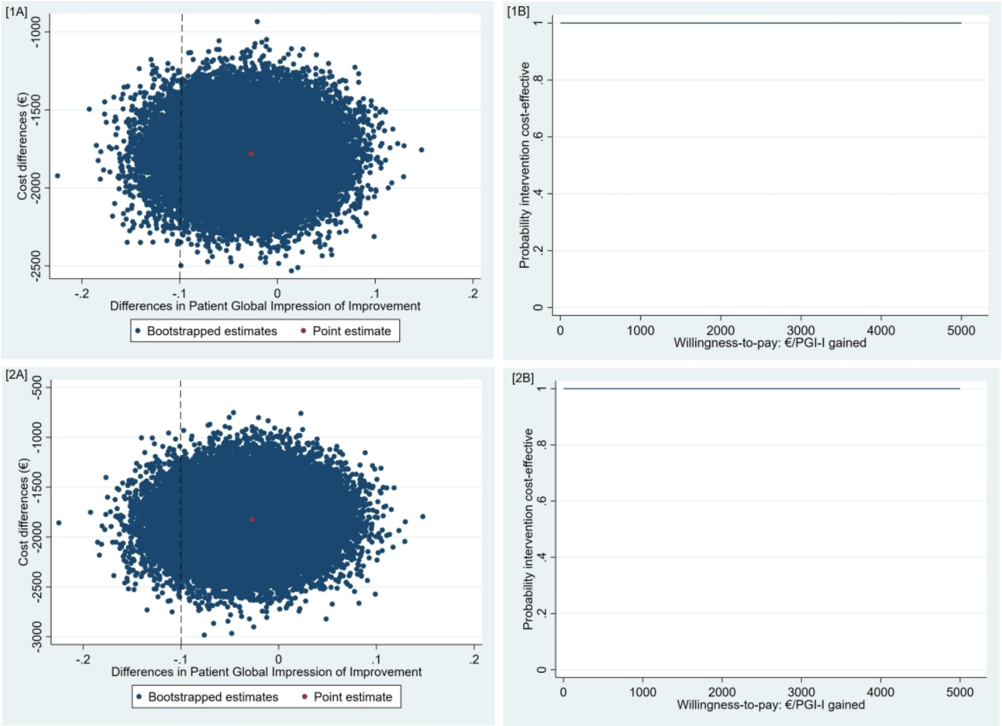

FIGURE 1. COST-EFFECTIVENESS PLANES AND COST-EFFECTIVENESS ACCEPTABILITY CURVES FOR PATIENT GLOBAL IMPRESSION IMPROVEMENT (PGI-I). Cost-effectiveness planes (CE-planes [1A] and [2A]) and cost-effectiveness acceptability curves (CEACs [2A] and [2B]) comparing pessary therapy with surgery for the PGI-I outcome from a healthcare and a societal perspective, respectively. CE-planes show the incremental cost-effectiveness ratio point estimate (ICER, red dot) and the distribution of the 5000 replications of the bootstrapped cost-effective pairs (blue dots). CEACs indicate the probability of pessary therapy being cost-effective compared with surgery (y-axis) for different willingness-to-pay (WTP) thresholds per unit of PGI-I gained (x-axis). The dashed line represents the non-inferiority margin of 10%. [1A] and [2A] show that all of bootstrapped cost-effect pairs were distributed in the southern quadrants of the CE-planed meaning that the pessary therapy is less costly but could also be less and more effective. 95.5% bootstrapped cost-effect pairs are situated on the right of the non-inferiority margin for effects.[1B] and [2B] indicate a steady probability of 1 that the pessary therapy is cost-effective compared with surgery for different WTP thresholds per PGI-I gained.

853x618mm (96 x 96 DPI)

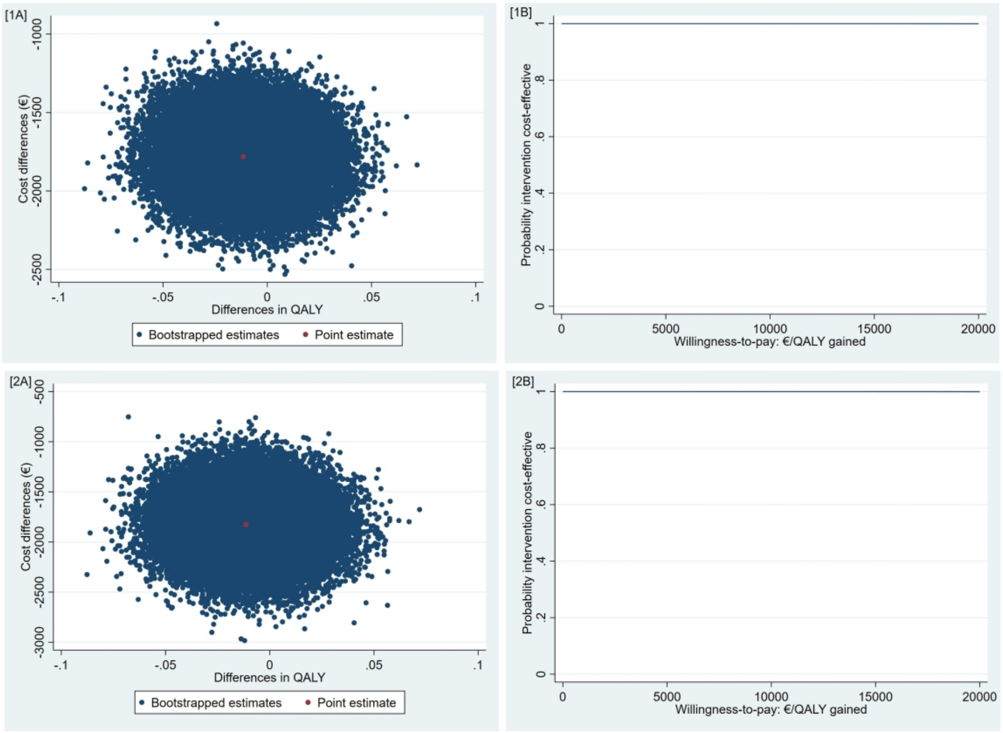

FIGURE 2. COST-EFFECTIVENESS PLANES AND COST-EFFECTIVENESS ACCEPTABILITY CURVES FOR QUALITY-ADJUSTED LIFE-YEARS (QALY). Cost-effectiveness planes (CE-planes [1A] and [2A]) and cost-effectiveness acceptability curves (CEACs [2A] and [2B]) comparing pessary therapy with surgery for QALY from a healthcare and a societal perspective, respectively. CE-planes show the incremental cost-effectiveness ratio point estimate (ICER, red dot) and the distribution of the 5000 replications of the bootstrapped cost-effective pairs (blue dots). CEACs indicate the probability of pessary therapy being cost-effective compared with surgery (y-axis) for different willingness-to-pay (WTP) thresholds per QALY gained (x-axis). [1A] and [2A] show that all of bootstrapped cost-effect pairs were distributed in the southern quadrants of the CE-planed meaning that the pessary therapy is less costly but could also be less and more effective. [1B] and [2B] indicate a steady probability of 1 that the pessary therapy is cost-effective compared with surgery for different WTP thresholds per QALY gained.

850x618mm (96 x 96 DPI)

SUPPLEMENTARY TABLE 1. RESOURCES AND UNIT COSTS

**Resources**	**Unit costs**	**Year**	**Reference**
Pessary device			
Milex®	€64	2022	Market price: bol.com
Arabin®	€73	2022	Market price: bol.com
Other brand (average)	€68	2022	Market price: bol.com
Pessary placement	€109	2022	Dutch costing manual[1]
Surgery			
Sacrospinous hysteropexy (care product 149999033)	€5835	2022	DBC[2]
Sacrospinous fixation (care product 149999047)	€4640	2022	DBC[2]
Manchester–Fothergill procedure (care product 149999047)	€4640	2022	DBC[2]
Abdominal sacrocolpopexy (care product 149999033)	€5835	2022	DBC[2]
Sacrocervicopexy care product 149999033)	€5835	2022	DBC[2]
Vaginal hysterectomy (care product 149999047)	€4640	2022	DBC[2]
Average surgical procedures costs (used as WTP threshold)	€5237	2022	DBC[2]
**Other resources**			
General practitioner consultation	€39	2022	Dutch costing manual[1]
Other healthcare professional consultation at primary care	€39	2022	Dutch costing manual[1]
Medical specialist consultation at secondary care	€109	2022	Dutch costing manual[1]
Hospital readmission (1 day)	€568	2022	Dutch costing manual[1]
Paid working hour for women	€38	2022	Dutch costing manual[1]

DBC: Diagnosis Treatment Combination, in Dutch *Diagnose Behandeling CombinatieI.*

References:

1 Kanters TA, Bouwmans CAM, van der Linden N, *et al.* Update of the Dutch manual for costing studies in health care. *PLoS One* 2017;**12**. doi:10.1371/journal.pone.0187477
2 Diagnose Behandeling Combinatie (DBC) open data - NZa. https://www.opendisdata.nl/ (accessed 3 Sep 2022).

For peer review only

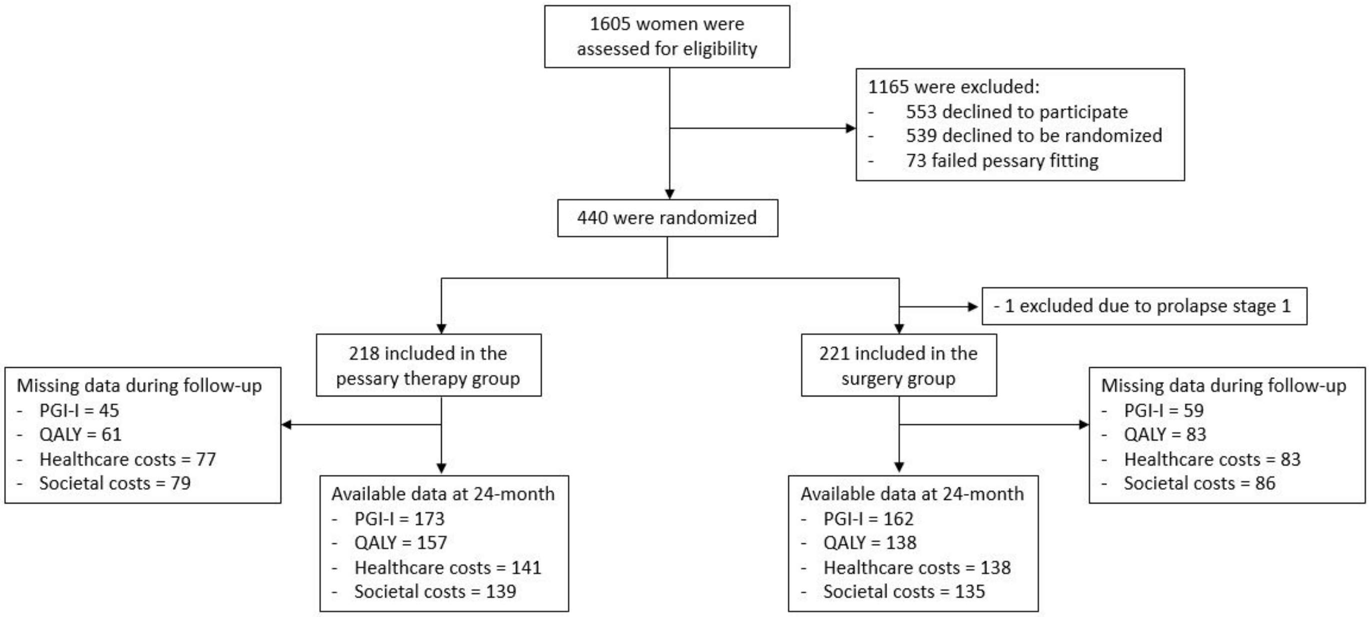

SUPPLEMENTARY Figure 1. FLOW DIAGRAM. Inclusion and available data at 24-month follow-up.

For peer review only

CHEERS 2022 Checklist

Topic	No.	Item	Location where item is reported
**Title**			
	1	Identify the study as an economic evaluation and specify the interventions being compared.	Page 1, 1st paragraph
**Abstract**			
	2	Provide a structured summary that highlights context, key methods, results, and alternative analyses.	Page 2
**Introduction**			
**Background and objectives**	3	Give the context for the study, the study question, and its practical relevance for decision making in policy or practice.	Page 4
**Methods**			
**Health economic analysis plan**	4	Indicate whether a health economic analysis plan was developed and where available.	Page 5, Study design, 1st paragraph
**Study population**	5	Describe characteristics of the study population (such as age range, demographics, socioeconomic, or clinical characteristics).	Page 5, Study population, 2nd paragraph
**Setting and location**	6	Provide relevant contextual information that may influence findings.	Page 6, Setting and location, 1st paragraph
**Comparators**	7	Describe the interventions or strategies being compared and why chosen.	Page 6, Comparators, Pessary therapy, 2nd paragraph and Surgery, 3rd paragraph
**Perspective**	8	State the perspective(s) adopted by the study and why chosen.	Page 7, Study perspective, time horizon, and discount rate, 1st paragraph
**Time horizon**	9	State the time horizon for the study and why appropriate.	Page 7, Study perspective, time horizon, and discount rate, 1st paragraph

Topic	No.	Item	Location where item is reported
**Discount rate**	10	Report the discount rate(s) and reason chosen.	Page 7, Study perspective, time horizon, and discount rate, 1st paragraph
**Selection of outcomes**	11	Describe what outcomes were used as the measure(s) of benefit(s) and harm(s).	Page 7, Outcomes, Health outcomes, 2nd and 3rd paragraph
**Measurement of outcomes**	12	Describe how outcomes used to capture benefit(s) and harm(s) were measured.	Page 7, Outcomes, Health outcomes, 2nd and 3rd paragraph
**Valuation of outcomes**	13	Describe the population and methods used to measure and value outcomes.	Page 7, Outcomes, Health outcomes, 2nd and 3rd paragraph
**Measurement and valuation of resources and costs**	14	Describe how costs were valued.	Page 8-9, Cost outcomes
**Currency, price date, and conversion**	15	Report the dates of the estimated resource quantities and unit costs, plus the currency and year of conversion.	Page 9, 1st paragraph, last sentence
**Rationale and description of model**	16	If modelling is used, describe in detail and why used. Report if the model is publicly available and where it can be accessed.	Page 9, Cost-effectiveness analysis, 3rd paragraph
**Analytics and assumptions**	17	Describe any methods for analysing or statistically transforming data, any extrapolation methods, and approaches for validating any model used.	Page 9-10, Cost-effectiveness analysis
**Characterising heterogeneity**	18	Describe any methods used for estimating how the results of the study vary for subgroups.	Page 10, Sensitivity analysis
**Characterising distributional effects**	19	Describe how impacts are distributed across different individuals or adjustments made to reflect priority populations.	Page 9, 3rd paragraph and Page 10, 1st paragraph
**Characterising uncertainty**	20	Describe methods to characterise any sources of uncertainty in the analysis.	Page 10, Sensitivity analysis
**Approach to engagement with patients and others affected by the study**	21	Describe any approaches to engage patients or service recipients, the general public, communities, or stakeholders (such as clinicians or payers) in the design of the study.	Page 11, Patient and Public Involvement

Topic	No.	Item	Location where item is reported
**Results**			
**Study parameters**	22	Report all analytic inputs (such as values, ranges, references) including uncertainty or distributional assumptions.	Page 12, Participants, 1st paragraph
**Summary of main results**	23	Report the mean values for the main categories of costs and outcomes of interest and summarise them in the most appropriate overall measure.	Page 12-13, Effectiveness, Costs
**Effect of uncertainty**	24	Describe how uncertainty about analytic judgments, inputs, or projections affect findings. Report the effect of choice of discount rate and time horizon, if applicable.	Page 13, Cost-effectiveness analysis. Page 14, Sensitivity analysis
**Effect of engagement with patients and others affected by the study**	25	Report on any difference patient/service recipient, general public, community, or stakeholder involvement made to the approach or findings of the study	Not Applicable
**Discussion**			
**Study findings, limitations, generalisability, and current knowledge**	26	Report key findings, limitations, ethical or equity considerations not captured, and how these could affect patients, policy, or practice.	Page 15-17
**Other relevant information**			
**Source of funding**	27	Describe how the study was funded and any role of the funder in the identification, design, conduct, and reporting of the analysis	Page 3
**Conflicts of interest**	28	Report authors conflicts of interest according to journal or International Committee of Medical Journal Editors requirements.	Page 3

From: Husereau D, Drummond M, Augustovski F, et al. Consolidated Health Economic Evaluation Reporting Standards 2022 (CHEERS 2022) Explanation and Elaboration: A Report of the ISPOR CHEERS II Good Practices Task Force. Value Health 2022;25. doi:10.1016/j.jval.2021.10.008

PEOPLE study

Pessary or Surgery for a Symptomatic Pelvic Organ Prolapse

Study protocol

For peer review only

For peer review only - http://bmjopen.bmj.com/site/about/guidelines.xhtml

Table of contents

1. Study protocols 3

1.1 Original study protocol..4

1.2 Final study protocol 55

1.3 Summary of amendments to study protocol.. 107

1.3.1 Detailed summary of all amendments 107

1.3.2 Table with amendments and corresponding section 109

For peer review only

For peer review only - http://bmjopen.bmj.com/site/about/guidelines.xhtml

1. Study protocols

Original study protocol:	Version 1.5, November 2014
Final study protocol:	Version 1.22, February 2018

For peer review only

1.1 Original study protocol

Pessary or surgery for symptomatic pelvic organ prolapse

Version 1.~~4~~ 5 ~~October~~ November 2014

For peer review only

For peer review only - http://bmjopen.bmj.com/site/about/guidelines.xhtml

PROTOCOL TITLE **'Pessary or surgery for symptomatic pelvic organ prolapse'**

**Protocol ID**	**2014 / 1.4**
**Short title**	**Pessary or surgery for symptomatic pelvic organ prolapse**
**EudraCT number**	***Not applicable***
**Version**	**1.~~4~~5**
**Date**	**O~~ctober~~ November 2014**
**Coordinating investigator/project leader**	**Prof. Dr. C.H. van der Vaart, gynaecologist**<br>**University Medical Centre Utrecht**
**Principal investigator(s) (in Dutch: hoofdonderzoeker/ uitvoerder)**	**Dr. A. Vollebregt, gynaecologist**<br>**Spaarne Hospital**<br><br>**M.K. van de Waarsenburg, MD**<br>**University Medical Centre Utrecht**
**Multicenter: per site**	**Prof. Dr. C.H. van der Vaart, UMC Utrecht**<br>**Dr. A.L. Milani, Reinier de Graaf Gasthuis**<br>**Dr. A. Vollebregt, Spaarne Hospital Hoofddorp**<br>**Dr. J.P.W.R. Roovers, Academic Medical Center**<br>**Dr. K.B. Kluivers, Radboud UMC Nijmegen**<br>**Dr. V. Dietz, Catharina Hospital Eindhoven**<br>**Dr. H.W.F. van Eijndhoven, Isala Clinic Zwolle**<br>**Dr. H.S. The, Sint Antonius Hospital Nieuwegein**<br>**Dr. R.P. Schellart, Kennemer Gasthuis Haarlem**<br>**Drs. A.M.W. Broekman, Sint Franciscus Gasthuis Rotterdam**<br>**Drs. J. van Bavel, Amphia Hospital Breda**
**Sponsor (in Dutch: verrichter/opdrachtgever)**	**Prof. Dr. C.H. van der Vaart, gynaecologist**
**Subsidising party**	**ZonMw Project nr 837002525**
**Independent expert (s)**	**Dr. R.P. Zweemer**<br><br>**University Medical Centre Utrecht**

For peer review only

**Laboratory sites <*if applicable*>**	***Not applicable***
**Pharmacy <*if applicable*>**	***Not applicable***

For peer review only

PROTOCOL SIGNATURE SHEET

Name	Signature	Date
**Head of Department:**<br><br>**Prof. Dr. B.C.J.M. Fauser**<br>**Department of Reproductive Medicine and Gynaecology**<br>**University Medical Centre Utrecht**		
**[Coordinating Investigator/Project leader/Principal Investigator]:**<br><br>**Prof. Dr. C.H. van der Vaart, gynaecologist**<br>**University Medical Centre Utrecht**		

For peer review only

For peer review only - http://bmjopen.bmj.com/site/about/guidelines.xhtml

TABLE OF CONTENTS

1. INTRODUCTION AND RATIONALE 9
2. OBJECTIVES 13
3. STUDY DESIGN 14
4. STUDY POPULATION 15
4.1 Population (base) 15
4.2 Inclusion criteria 15
4.3 Exclusion criteria 15
4.4 Sample size calculation 15
5. TREATMENT OF SUBJECTS 17
5.1 Investigational product/treatment 17
5.2 Use of co-intervention (if applicable) 17
5.3 Escape medication (if applicable) 18
6. INVESTIGATIONAL PRODUCT 19
6.1 Name and description of investigational product(s) 19
6.2 Summary of findings from non-clinical studies 19
6.3 Summary of findings from clinical studies 19
6.4 Summary of known and potential risks and benefits 20
6.5 Description and justification of route of administration and dosage 20
6.6 Dosages, dosage modifications and method of administration 20
6.7 Preparation and labelling of Investigational Medicinal Product 20
6.8 Drug accountability 20
7. NON-INVESTIGATIONAL PRODUCT 21
7.1 Name and description of non-investigational product(s) 21
7.2 Summary of findings from non-clinical studies 21
7.3 Summary of findings from clinical studies 21
7.4 Summary of known and potential risks and benefits 21
7.5 Description and justification of route of administration and dosage 21
7.6 Dosages, dosage modifications and method of administration 21
7.7 Preparation and labelling of Non Investigational Medicinal Product 21
7.8 Drug accountability 21
8. METHODS 22
8.1 Study parameters/endpoints 22
8.1.1 Main study parameter/endpoint 22
8.1.2 Secondary study parameters/endpoints (if applicable) 22
8.1.3 Other study parameters (if applicable) 25
8.2 Randomisation, blinding and treatment allocation 25
8.3 Study procedures 26
8.4 Withdrawal of individual subjects 27
8.4.1 Specific criteria for withdrawal (if applicable) 27

For peer review only - http://bmjopen.bmj.com/site/about/guidelines.xhtml

8.5 Replacement of individual subjects after withdrawal 27
8.6 Follow-up of subjects withdrawn from treatment 27
8.7 Premature termination of the study 27
9. SAFETY REPORTING 28
9.1 Section 10 WMO event 28
9.2 AEs, SAEs and SUSARs 28
9.2.1 Adverse events (AEs) 28
9.2.2 Serious adverse events (SAEs) 28
9.2.3 Suspected unexpected serious adverse reactions (SUSARs) 29
9.3 Annual safety report 29
9.4 Follow-up of adverse events 29
9.5 [Data Safety Monitoring Board (DSMB) / Safety Committee] 29
10. STATISTICAL ANALYSIS 30
10.1 Primary study parameter(s) 30
10.2 Secondary study parameter(s) 30
10.3 Other study parameters 31
10.4 Interim analysis (if applicable) 32
11. ETHICAL CONSIDERATIONS 33
11.1 Regulation statement 33
11.2 Recruitment and consent 33
11.3 Objection by minors or incapacitated subjects (if applicable) 33
11.4 Benefits and risks assessment, group relatedness 33
11.5 Compensation for injury 33
11.6 Incentives (if applicable) 33
12. ADMINISTRATIVE ASPECTS, MONITORING AND PUBLICATION 35
12.1 Handling and storage of data and documents 35
12.2 Monitoring and Quality Assurance 35
12.3 Amendments 35
12.4 Annual progress report 35
12.5 End of study report 35
12.6 Public disclosure and publication policy 36
13. STRUCTURED RISK ANALYSIS 37
13.1 Potential issues of concern 37
13.2 Synthesis 37
14. REFERENCES 38
Appendix 1… 42
Appendix 2… 43
Appendix 3… 45
Appendix 4… 46
Appendix 5… 47

For peer review only - http://bmjopen.bmj.com/site/about/guidelines.xhtml

LIST OF ABBREVIATIONS AND RELEVANT DEFINITIONS

**ABR**	**ABR form, General Assessment and Registration form, is the application form that is required for submission to the accredited Ethics Committee (In Dutch, ABR = Algemene Beoordeling en Registratie)**
**AE**	**Adverse Event**
**AR**	**Adverse Reaction**
**CA**	**Competent Authority**
**CCMO**	**Central Committee on Research Involving Human Subjects; in Dutch: Centrale Commissie Mensgebonden Onderzoek**
**CV**	**Curriculum Vitae**
**DSMB**	**Data Safety Monitoring Board**
**EU**	**European Union**
**EudraCT**	**European drug regulatory affairs Clinical Trials**
**GCP**	**Good Clinical Practice**
**IB**	**Investigator's Brochure**
**IC**	**Informed Consent**
**IMP**	**Investigational Medicinal Product**
**IMPD**	**Investigational Medicinal Product Dossier**
**METC**	**Medical research ethics committee (MREC); in Dutch: medisch ethische toetsing commissie (METC)**
**(S)AE**	**(Serious) Adverse Event**
**SPC**	**Summary of Product Characteristics (in Dutch: officiële productinfomatie IB1-tekst)**
**Sponsor**	**The sponsor is the party that commissions the organisation or performance of the research, for example a pharmaceutical company, academic hospital, scientific organisation or investigator. A party that provides funding for a study but does not commission it is not regarded as the sponsor, but referred to as a subsidising party.**
**SUSAR**	**Suspected Unexpected Serious Adverse Reaction**
**Wbp**	**Personal Data Protection Act (in Dutch: Wet Bescherming Persoonsgevens)**
**WMO**	**Medical Research Involving Human Subjects Act (in Dutch: Wet Medisch-wetenschappelijk Onderzoek met Mensen**

For peer review only - http://bmjopen.bmj.com/site/about/guidelines.xhtml

SUMMARY

Rationale: Moderate to severe pelvic organ prolapse symptoms can be treated with pessary or surgery. Both treatments appear to be effective, but have not been compared directly.

Hypothesis: The strategy of pessary as initial therapy is as effective as direct surgery for moderate to severe POP, but it is associated with lower costs.

Objective: The primary objective is to compare the effectiveness and cost-effectiveness of pessary versus surgery as initial treatment for moderate to severe symptomatic pelvic organ prolapse (POP) in women at two year after initiation of treatment. The secondary objective is the development of a prediction model for failure of pessary use and surgery within 2 years.

Study design: Cohort study with embedded randomized controlled trial.

Study population: Treatment naïve women with POP who present with moderate to severe symptoms.

Intervention (if applicable): Pessary therapy or vaginal POP surgery.

Main study parameters/endpoints:

Primary outcome: Global impression of improvement of POP symptoms at 24 months measured with PGI-I

Secondary outcomes:

- Changes in symptom bother and disease-specific quality of life at 12 and 24 months follow-up
- Changes of sexual function at 12 and 24 months follow-up
- Changes in general quality of life at 3, 6, 12 and 24 months of follow up
- Adverse events/complications related to both treatment strategies during the study period
- Development of prediction model to identify factors for failing of pessary and surgery.
- Costs-effectiveness analyses

Nature and extent of the burden and risks associated with participation, benefit and group relatedness: Both treatment arms are routine treatments in the Netherlands. Patients in the RCT can have the risks of surgery instead of the risks from pessary therapy.

For peer review only - http://bmjopen.bmj.com/site/about/guidelines.xhtml

1. INTRODUCTION AND RATIONALE

Problem definition

Female pelvic organ prolapse (POP) is a common problem in women that negatively affects quality of life. The estimated prevalence of symptomatic POP among women between 45-85 years of age is 8.3 - 11% [1,2]. It is current practice in the Netherlands that the general practitioner (GP) treats the majority of women with POP symptoms. Women with moderate to severe POP symptoms are often referred to a gynecologist for treatment. This study focuses at the subgroup of moderate to severe POP.

Known effective treatment options for moderate to severe POP are pessary or surgery. A pessary has proven its effectiveness in the treatment of symptomatic POP, mainly in cystocele and uterine descent. However, studies are mainly observational in nature and inherently subject to selection and indication bias [3]. In literature, outcomes of pessary therapy are mainly recorded in terms of (dis-) continuation of therapy and to a much lesser extent in terms of symptom relief. The pessary continuation rate is 60% [3]. This is confirmed by a Dutch pilot study in 65 women that showed a satisfaction with pessary in 57% of women and an operation rate of 43% at 12 months follow up [4]. In this study, 80% of women who continued pessary therapy reported much to very much improvement of their POP symptoms at 1 year follow up [4]. Reasons of discontinuation are pressure ulcer, vaginal discharge, discomfort or loss of fitting. These complications are reported to occur in up to 53% of women [5]. Half of them will decide to stop using pessary, but it is unclear which characteristics predict this outcome. Check-up of pessary therapy can be performed by either a general practitioner (GP), gynecologist or by self-management. According to a recent survey 50% percent of gynecologists involved in urogynaecology always offer self-management 40% on indication, and 10% never. Pessary therapy is inexpensive and costs are mainly related to doctor visits and treatment of side effects. In case of self-management costs might even be lower,

Surgery for POP results in much to very much improvement of symptoms in 80% of women and improvement of quality of life [6-9]. An anterior colporraphia is considered the standard procedure for a cystocele, as is the posterior colporraphia for a rectocele. For uterine descent uterus sparing techniques, like sacrospinous hysteropexy (SH) and modified Manchester-Fothergill procedure, or vaginal hysterectomy can be performed [10-12]]. Complications of POP surgery are temporary urinary retention, temporary buttock pain in case of sacrospinous hysteropexy, urinary tract infection, hematoma or dyspareunia [11]. These complications seldom lead to persistent morbidity. The most

For peer review only - http://bmjopen.bmj.com/site/about/guidelines.xhtml

common “complication” is the recurrence of symptomatic POP or de novo stress-incontinence that may lead to additional surgery, pessary therapy, or pelvic floor physiotherapy. As part of a RCT, comparing mesh with fascia plication, we found that
11% of women needed additional surgery after anterior colporraphia at 24 months follow up [7,9]. As in pessary therapy, the characteristics that predict successful or unsuccessful surgical therapy are largely unknown.

The decision which treatment option to choose depends on both patient and doctor’s preferences. In our pilot survey 70% of gynecologists informed their patients about the possibility of pessary therapy, but it is unknown how many women actually received a pessary. A recent Dutch study showed that 48% of treatment-naive women preferred surgery, 36% a pessary and 16% had no preference [28]. It is therefore reasonable to assume that at least 50% of treatment naïve women with moderate to severe prolapse symptoms will have surgery as primary treatment.

Although clinical efficacy appears to favor surgery [3], the large variation in study design, outcome measurements and loss to follow up makes any comment on the best treatment option speculative. This is recognized in two recent reviews on the subject that both urge the need for randomized trials comparing surgery and pessary for POP [13,14]. Efficacy can be expressed in terms of clinical outcome but also in terms of cost-effectiveness. It is obvious that surgery (especially hospital costs) is much more expensive than pessary therapy, but the cost-effectiveness of the surgical or pessary strategy has never been assessed. Based on current cohort and case-control studies we hypothesize that a strategy of initial pessary therapy for moderate to severe POP, is more cost-effective than surgery.
We propose to perform a randomized controlled trial to generate evidence for the optimal and most cost-effective primary treatment for moderate to severe POP, including a better a priori patient selection for treatment by identifying factors of failure for pessary therapy or surgery.

Relevance
At present a national multidisciplinary guideline on the diagnosis and treatment of POP is completed. The guideline identifies the lack of evidence with respect to the best treatment option for moderate to severe prolapse, a conclusion that is confirmed by the 2013 Cochrane Collaboration review [13]. In this evidence “vacuum” both doctors and patient
preferences rule, but unfortunately these are not supported by facts. If we look at the available data the following calculation emerges.

For peer review only - http://bmjopen.bmj.com/site/about/guidelines.xhtml

About 60% of women who start pessary therapy in the specialist care setting will continue using it at one year [4,15]. Eighty percent of them will report much to very much improvement, resulting in an overall 48% much to very much improvement. The majority of the 40% of women who are not satisfied with pessary therapy will request or are offered additional surgery. After surgery 80% of women report much to very much improvement of POP symptoms [6]. Combining these percentages, women who originally started with pessary therapy may also expect 80% (48% after initial pessary treatment + 32% after additional surgery =) much to very much improvement. Based on these estimates it is expected that the outcome of both treatment strategies will eventually result in a global improvement of symptoms in 80% of women. With equal clinical outcomes of both strategies the costs needed to obtain these outcomes become crucial. With the exception of a cost calculation based on a Markov model, no direct cost-effectiveness studies on the use of pessary or surgery for POP have been performed [16]. The relevance of this project, with the high prevalence of POP worldwide, associated costs and insufficient evidence, is high. We have searched the www.clinicaltrials.gov database (3th March 2014) on similar studies (comparing pessary with surgery) but none were found.

However, if we were to prove that pessary therapy is more cost-effective then surgical treatment, this does not imply that a trial of pessary should always be undertaken. There is also insufficient evidence on which patient characteristics are associated with failure of pessary treatment or surgery (systematic review). The knowledge on how to predict which women will have a very low chance of success with pessary therapy can further improve effective treatment strategy management. This will contribute to treatment efficacy. This is not only very relevant for the hospital specialist care setting, but this knowledge can also be implemented in general practitioner practice units.

There is very limited evidence on the optimal management strategy for pessary cleaning, both in time interval as well as in who should perform the cleaning. Our study is unique and therefore relevant since self-management is advocated in the study setting. This will not only allow it to obtain data in a standardized way, but also involves the woman in her own management. This involvement is strongly advocated by two major gynecologic patient organizations ('Patienten Gynaecologie Nederland' and the 'Stichting Bekkenbodem Patienten'). These two organizations, as well as the Dutch urogynaecological consortium have identified this study to be highly relevant.

For peer review only - http://bmjopen.bmj.com/site/about/guidelines.xhtml

In line with the report "Medisch Specialistische zorg 20/20" we are heading towards integral health care in which the general physician and medical specialist will work more closely together, using the same treatment protocol for various illnesses. The information and conclusions of this trial will add level I scientific evidence to such an integral protocol and guideline for women with symptomatic POP. This will aid in a better patient selection that will need referral to the specialist. The data on patient's self-management of pessary treatment will supply information for patient instructions, which are relevant for information leaflets on the subject.

For peer review only

2. OBJECTIVES

The aim of this multicenter pragmatic cohort study with embedded randomized controlled non-inferiority trial comparing pessary therapy versus surgery is twofold:

1. To prospectively compare the effectiveness and cost-effectiveness of pessary therapy or surgery as primary treatment of moderate to severe symptomatic cystocele, uterine descent and/or rectocele in women at two year after initiation of treatment.
2. To develop a prediction model for failure of pessary use and surgery within the first 2 years.

For peer review only

For peer review only - http://bmjopen.bmj.com/site/about/guidelines.xhtml

3. STUDY DESIGN

Multicenter pragmatic cohort study with an embedded randomized controlled non-inferiority trial comparing pessary [CE 0086] therapy versus surgery including an economic evaluation. The follow up will be 24 months.

After a short (30 minutes) trial of pessary fitting before randomization into our protocol. This ensures that only women who fit both treatment options enter the randomization procedure. The trial is short and only aims at fitting, not symptom relief. For those women with an unsuccessful pessary fitting baseline characteristics will be recorded to allow analyses of this group.

See also appendix 1.

For peer review only

For peer review only - http://bmjopen.bmj.com/site/about/guidelines.xhtml

4. STUDY POPULATION

4.1 Population (base)

All women with a symptomatic POP will be included.

4.2 Inclusion criteria

In order to be eligible to participate in this study, a subject must meet all of the following criteria:

1. Women with a prolapse stage 2 or more.
2. Women with moderate to severe POP symptoms. Moderate to severe POP symptoms is defined as a prolapse domain score > 33 on the validated Dutch version of the Pelvic Floor Distress Inventory (PFDI-20) [8, 23, 24].
3. Women who have had a successful pessary fitting procedure: for the RCT.
4. Written informed consent.

4.3 Exclusion criteria

A potential subject who meets any of the following criteria will be excluded from participation in this study:

1. Prior urogynaecological (prolapse or incontinence) surgery
2. Probability of future childbearing
3. Insufficient knowledge of the Dutch language
4. Co-morbidity causing increased surgical risks at the discretion of the surgeon
5. Major psychiatric illness
6. Prior pessary use

4.4 Sample size calculation

With 198 women per group, we will have 80% power to reject the null hypothesis that pessary therapy is inferior to surgery, with a 1-sided alpha of 0.05, a non-inferiority margin of 10% and the proportion in the standard group is 80% (NQueryAdvisor). Accounting for 10% loss to follow-up we plan to randomize 436 patients.

The sample size calculation for prediction models is based on the number of failures of pessary or surgical therapy. For each potential predictor in the model we need 10-15 failures. Our pessary group sample size is 198 women. An estimated 40% (80 women) will cross over to surgery and can be regarded as failures. Our sample size is therefore sufficient

For peer review only - http://bmjopen.bmj.com/site/about/guidelines.xhtml

to develop the prediction model for failure of pessary therapy for 6 to 8 items. In the surgery group 20% of women will not be satisfied with the result of treatment. With the same sample size of 198 women, the 40 women who are dissatisfied allow us to study up to 4 potential predictive factors.

For peer review only

For peer review only - http://bmjopen.bmj.com/site/about/guidelines.xhtml

5. TREATMENT OF SUBJECTS

5.1 Investigational product/treatment

Pessary [CE 0086] therapy and surgery both are options for the treatment of a symptomatic POP. Ten large urogynecological units (university hospitals or teaching hospitals) that have worked together in previous consortium studies will participate in this multicenter trial. All participating gynaecologists have fitted at least 100 pessaries and performed more than 100 surgical POP procedures prior to the start of this study.

All pessaries are made of modern silicon material. All types of pessaries, both supportive and occlusive/space filling are allowed according to the judgment of the gynaecologist. A recent randomized trial comparing supportive (ring) and occlusive (Gelhorn) showed no differences [17]. After placing the pessary, all women will receive verbal and written instructions on the self-management of pessary therapy.

The first pessary follow up visit will always be performed by the gynaecologist. In case of self-management the frequency of cleaning is left to her personal judgment, but may not exceed 1 month. If self-management is not possible, women will be seen at 4 months intervals for pessary cleaning and vaginal inspection, preferable by their GP. In case of vaginal atrophy topical estrogens will be advised according to pharmaceutical guidelines. The diagnosis of atrophy is left to the judgment of the treating physician, since no clear definition for atrophy is available yet [18].

All surgical procedures will be performed according to our national guidelines. In this pragmatic trial the decision which technique to use is left, to the discretion of the gynaecologist, within the limitations below [19]. Cystocele repair will consist of conventional anterior colporrhaphy [9]. For uterine descent different techniques are allowed [20]. These techniques can either be uterus sparing (sacrospinous hysteropexy [10], modified Manchester-Fothergill procedure [12] or a abdominal sacrocolpopexy [9]) or a vaginal hysterectomy. Recent studies showed similar effectiveness on both anatomical and functional outcomes for these different techniques [10, 12, 21]. A coexistent stage 2 rectocele repair will be a conventional colporrhaphia posterior. All procedures are performed under general or spinal anesthesia and under antibiotics and thrombosis prophylaxis according to local protocols.

5.2 Use of co-intervention (if applicable)

Not applicable.

For peer review only - http://bmjopen.bmj.com/site/about/guidelines.xhtml

5.3 Escape medication (if applicable)

Not applicable.

For peer review only

For peer review only - http://bmjopen.bmj.com/site/about/guidelines.xhtml

6. INVESTIGATIONAL PRODUCT

6.1 Name and description of investigational product(s)

6.2 Summary of findings from non-clinical studies

Not applicable.

6.3 Summary of findings from clinical studies

Three systematic reviews of the literature were performed by four members of our Dutch urogynaecology consortium (details in appendix 2-4) that concluded:

1. Systematic review on the (cost)effectiveness of pessary use compared to surgery

There are a very limited number of comparative studies on the efficacy of surgery or pessary use for POP. The differences in study population, inclusion criteria, follow-up period, large numbers of loss to follow-up, different outcome measures makes interpretation difficult if not impossible. The two studies that presented data on functional outcome in terms of prolapsed symptom reduction were favorable for surgery (appendix 2).

2. Systematic review of factors influencing pessary fitting and continuation

A systematic review was performed to identify the satisfactory pessary fitting rate and the continuation rate of pessary use. The factors influencing these rates as well as the cross over to prolapse surgery were identified from previous studies (appendix 3).

Summarizing the results show that an estimated 75% of women will have a successful fitting and 59% will continue pessary use at variable follow-up between 3 months and 5 years. In these 18 studies, 8 factors have been tested more than 4 times as prognostic factor of successful pessary use: Stress urinary incontinence was found associated with discontinuation of pessary in 5 out of 7 studies. In 7 out of 10 studies previous prolapse surgery or hysterectomy was associated with less continuation of pessary use. Higher age was related to continuation of pessary use in 3 out of 6 studies, whereas no correlation was found in the other studies. In 1 out of 4 studies sexual activity was related to longer pessary use, whereas in 1 out of 4 related to the choice for surgery. In the two other studies no correlation was found. In one study where the prolapse in a specific vaginal compartment was related to outcome, nor cystocele was related to longer pessary use.

Parity en menopausal status and hormonal replacement were mostly not related to continued pessary use.

For peer review only - http://bmjopen.bmj.com/site/about/guidelines.xhtml

3. Review of factors influencing failure of POP surgery.

A systematic review of factors influencing failure of POP surgery was performed concerning recurrence after surgery (surgery failure). There were 1 case control study, 3 prospective studies and 6 retrospective studies. There were 2298 women included in the studies. Forty-four (44) potential risk factors have been studied, of which 12 risk factors have at least once been identified as statistically significant risk factors in a multivariate logistic regression analysis (appendix 4).

6.4 Summary of known and potential risks and benefits

The present study carries low risks for the participant. Pessary [CE 0086] or surgery is standard care for symptomatic pelvic organ prolapse. Known risks for surgery are blood loss, risk of infection, dyspareunia, urine incontinence or a recurrence of a symptomatic pelvic organ prolapse.

The benefit of the study lies in a better understanding of satisfaction and cost effectiveness.

6.5 Description and justification of route of administration and dosage

Not applicable

6.6 Dosages, dosage modifications and method of administration

Not applicable

6.7 Preparation and labelling of Investigational Medicinal Product

Not applicable

6.8 Drug accountability

Not applicable

For peer review only

7. NON-INVESTIGATIONAL PRODUCT

Not applicable.

7.1 Name and description of non-investigational product(s)

Not applicable.

7.2 Summary of findings from non-clinical studies

Not applicable.

7.3 Summary of findings from clinical studies

Not applicable.

7.4 Summary of known and potential risks and benefits

Not applicable.

7.5 Description and justification of route of administration and dosage

Not applicable.

7.6 Dosages, dosage modifications and method of administration

Not applicable.

7.7 Preparation and labelling of Non Investigational Medicinal Product

Not applicable.

7.8 Drug accountability

Not applicable.

For peer review only

8. METHODS

8.1 Study parameters/endpoints

8.1.1 Main study parameter/endpoint

The primary outcome of this study is the percentage of women with much or very much improvement of POP symptoms at 2 years follow-up, as measured with the Patient Global Impression of Improvement (PGI-I)scale [22].
PGI-I is a 7-point Likert scale, with scores ranging from very much worse to very much improved. Success is defined as 'much or very
much' improvement.

8.1.2 Secondary study parameters/endpoints (if applicable)

1. Changes in symptom bother and quality of life at 12 and 24 months follow up.
2. Changes in sexual function at 12 and 24 months follow up.
3. Changes in general quality of life at 3, 6, 12 and 24 months.
4. Adverse events/complications related to both treatment strategies.
5. Development of prediction model to identify fail factors for pessary and surgery
6. Cost-effectiveness

8.1.3 Other study parameters (if applicable)

Baseline characteristics: Age; ethnicity; alcohol; smoking; number and mode of deliveries; menopausal status; hormone use; drug use; height; weight; co-morbidity (hypertension, diabetes mellitus, COPD, neurological disease, depression, cardiovascular disease); history of gynaecological operations; family history of prolapse; allergies, incontinence and sexual activity.
Physical examination: time, POP-Q, atrophy, stress test, blood loss, excessive discharge.

8.2 Randomisation, blinding and treatment allocation

After written informed consent is obtained, and inclusion and exclusion criteria are assessed, women will be randomly allocated in a 1:1 ratio to either treatment with a pessary or surgical treatment. Randomization will be done web based using ALEA, the software for randomization in clinical trials currently used by most studies in the Dutch consortium for studies on women's health and reproduction studies. The

For peer review only - http://bmjopen.bmj.com/site/about/guidelines.xhtml

randomization sequence will be computer generated using variable blocks of two and four, stratified for centre.

After entering the woman's initials and confirming inclusion criteria on the website, a unique number for randomization will be generated and the allocation code will be disclosed. This unique number cannot be deleted afterwards. This study will be open label because the nature of the intervention meant that masking to the intervention was not possible.

Women who attend the cohort will also be registered in ALEA.

8.3 Study procedures (see also appendix 5)

This study will be performed within the Dutch Urogynaecology Research Consortium, a subdivision of the Dutch Consortium for studies on women's health. Infrastructure (research nurses for counseling and data-monitoring, the use of web-based data entry), expertise on methodology and cost-effectiveness is shared.

1. Symptom bother and disease-specific quality of life are measured with the Pelvic Floor Distress Inventory (PFDI-20) and Pelvic Floor Impact Questionnaire (PFIQ) [23,24]. The validated version of the Dutch PFDI consists of five domains: pelvic organ prolapse, urinary incontinence, overactive bladder, pain, and obstructive micturition. The PFIQ measures the impact of urogenital symptoms on quality of life and consist of five domains: physical functioning, mobility, emotional health, embarrassment and social functioning.

2. Sexual function is measured with the PISQ-R. It is an international disease-specific questionnaire that measures sexual functioning in sexually active and inactive participants [26]. At this time, the Dutch translation is in progress, which will be finished in 2014.

3. Generic quality of life is measured with the EQ-5D and a questionnaire "doktersbezoek".

4. The adverse events of surgery recorded will consist of; direct peri-and postoperative complications (bleeding, pain and infection); interventions for complications; recurrent prolapse; de novo stress urinary incontinence. The adverse events of pessary recorded will consist of; discharge; pain; discomfort; bleeding; involuntary loss of pessary; de novo stress urinary incontinence.

5. The development of a prediction model is separately described in paragraph "data analyses".

6. The economic evaluation is described below.

For peer review only - http://bmjopen.bmj.com/site/about/guidelines.xhtml

ANTICIPATED COST-EFFECTIVENESS/BUDGET IMPACT

Cost differences between the two strategies are mainly the result from differences in costs associated with the initial intervention. Cost of a POP procedure is estimated at 4000 euros direct medical costs, and 4000 euros associated with lost productivity (indirect costs) if a societal perspective is used. The direct cost of pessary use is estimated at 200 euros, including costs for the pessary itself (50 euros) and consultations in the first year (150 euros). The estimated cost differences between the two strategies depend on the extent that women are (and remain) satisfied with the initial procedure (surgical or pessary): in case of dissatisfaction with the procedure, additional costs are generated by a subsequent intervention ((re-)operation, pessary, or pelvic physiotherapy). The flowchart (see appendix 1) illustrates the expected outcomes for each strategy. Based on the assumptions reflected in this flowchart, combined with approximate estimates for unit costs for POP surgery, pessary, GP and specialist visits, the anticipated impact on the annual health care budget as well as societal costs were estimated.

At present, the primary therapy for women presenting with moderate to severe POP is either surgery or pessary. The exact ratio is unknown, but is probably 50/50. If 50% women would receive primary surgery the current medical costs amount to 34 million Euros. If all women would start with pessary therapy, these costs would be 20 million euros, and the potential budget impact would be 14 million Euros/year. As it is not realistic that all women will start with pessary if this strategy proves to be successful, at 85% implementation of the pessary strategy, the annual budget impact will be around 10 million euros. The economic impact to society (including indirect (productivity) costs) will be 28 million euros and 20 million euros, at 100% and 85% implementation, respectively.

Sensitivity analyses showed, that these “base case” results are affected by estimated unit costs for POP surgery (direct and indirect costs) and the satisfaction rate for pessary, relative to surgery, but even the most conservative assumptions would lead to major cost savings for the health care budget (5 million euros) and society (15 million euros).

ECONOMIC EVALUATION

Considering the non-inferiority design of the study, we will not be able to rule out a small but acceptable difference in favor of POP surgery. Consequently, the economic

For peer review only - http://bmjopen.bmj.com/site/about/guidelines.xhtml

evaluation will be setup as a cost-effectiveness analysis, where cost-effectiveness will be expressed as costs per improvement outcome (much or very much improvement on the Patient Global Impression of Improvement (PGI-I)), and the incremental cost-effectiveness ratio as costs saved per additional case of unsatisfactory outcome. We will also perform a cost-effectiveness analysis using QALYs as health outcome, to express the difference between the two strategies in terms of costs (saved) per QALY (lost).

The economic evaluation will therefore encompass a cost-effectiveness analysis (CEA), a cost-utility analysis (CUA) as well as a budget impact analysis (BIA) from a health care budget and a societal perspective, with a time horizon between randomization and 2 years follow up. The primary outcome in the cost-effectiveness analysis will be costs per satisfactory outcome (primary clinical outcome), and the incremental cost-effectiveness ratio will reflect the costs saved per additional case of unsatisfactory outcome. As we hypothesize that pessary as a primary strategy in these patients does not result in more unsatisfactory outcomes, increased use of pessary will result in a decrease in the number of POP surgeries, and associated costs of hospital stay, recovery and (from a societal perspective) productivity loss (non-inferior strategy at lower costs).

Based on data actually observed in the trial, total costs associated with both surgery and pessary as a primary strategy will be estimated. Total costs can be divided into direct medical costs, non-medical costs and indirect costs. Direct medical costs are generated by utilization of primary or secondary health care services (including POP surgery, hospital stay, diagnostic procedures, medication). Non-medical costs are generated by travel expenses, and informal care; and indirect costs result from lost productivity due to absence from work or lost opportunity for non-paid activities. Non-medical and indirect costs are only included in the analysis from a societal perspective.

Resource utilization will be documented in the clinical report form (CRF) and complementary patient questionnaires, based on the Medical Consumption Questionnaire (MCQ) and Productivity Costs Questionnaire (PCQ) [29,30]. In patients for whom complete follow-up is not available, cost and quality-of-life data will be extrapolated using multiple imputations. Unit costs will be based on Dutch guideline

For peer review only - http://bmjopen.bmj.com/site/about/guidelines.xhtml

prices (for primary and secondary health services, informal care and lost productivity), and market prices (for medication)[31,32]

Similarly, the incremental costs per QALY gained will be estimated over a period of two years. Health state utilities to estimate QALYs will be derived from an EQ-5D measurement at discharge, as well as at follow-up assessments. Utility values for EQ-5D scores will be based on UK-estimates (Dolan, 1997). Utility scores will be linearly interpolated, assuming constant increase/decrease between subsequent assessments.

Robustness for sampling uncertainty as well as uncertainty associated with cost estimates and assumptions will be assessed in sensitivity analyses, including: Dutch health states (Lamers, 2005) instead of the UK based model in the main analyses; and varying unit costs for pertinent volumes of health care utilization (e.g. costs of POP surgery, pessary use, productivity costs).

The incremental costs and effects will be depicted in a cost effectiveness plane and cost-effectiveness acceptability curves providing information directly interpretable as the probability of one intervention being cost-effective compared to the alternative given a ceiling ratio that policy makers are willing to invest.

BIA

In a budget impact analysis, study results will be extrapolated to the national level to estimate the total impact on the health care budget per annum for the Netherlands in terms of cost reduction and health outcomes (satisfactory outcomes as well as QALYs). As economic consequences of the intervention are expected to span multiple years, this accumulation of cost (savings) will be reflected in the budget impact analyses.

The Budget Impact Analysis will be executed according to the international ISPOR guidelines [33]. This framework for creating a budget impact model includes formalized guidance about the acquisition and use of data in order to make budget projections. In addition to the societal perspective, the BIA will therefore be also report economic consequences from the perspective of the Dutch budgetary health care framework (BKZ). If the probability of an unsatisfactory outcome exceeds the non-inferiority limit, recommending pessary as primary treatment for all women is not

For peer review only - http://bmjopen.bmj.com/site/about/guidelines.xhtml

feasible, and an economic evaluation/budget impact analysis is not sensible. To estimate costs, we will follow the Handleiding Kosten onderzoek CVZ 2010.

8.4 Withdrawal of individual subjects

Subjects can leave the study at any time for any reason if they wish to do so without any consequences. The investigator can decide to withdraw a subject from the study for urgent medical reasons.

8.4.1 Specific criteria for withdrawal (if applicable)

Not applicable.

8.5 Replacement of individual subjects after withdrawal

We will not replace patients who withdrew informed consent. We will replace patients that are randomized by mistake, for example because of technical errors with online randomization.

8.6 Follow-up of subjects withdrawn from treatment

Patients withdrawn from the intervention but not from informed consent will be followed up.

8.7 Premature termination of the study

This study includes standard care, therefore it is very unlikely that unexpected complications will occur. Therefore premature termination is not applicable.

For peer review only

9. SAFETY REPORTING

9.1 Section 10 WMO event

In accordance to section 10, subsection 1, of the WMO, the investigator will inform the subjects and the reviewing accredited METC if anything occurs, on the basis of which it appears that the disadvantages of participation may be significantly greater than was foreseen in the research proposal. The study will be suspended pending further review by the accredited METC, except insofar as suspension would jeopardise the subjects' health. The investigator will take care that all subjects are kept informed.

9.2 AEs, SAEs and SUSARs

9.2.1 Adverse events (AEs)

Adverse events are defined as any undesirable experience occurring to a subject during the study, whether or not considered related to the study. All adverse events reported spontaneously by the subject or observed by the investigator or his staff will be recorded. During visits complaints will be questioned systematically.

9.2.2 Serious adverse events (SAEs)

A serious adverse event is any untoward medical occurrence or effect that at any dose:

- results in death;
- is life threatening (at the time of the event);
- requires hospitalisation or prolongation of existing inpatients' hospitalisation (>4 days);
- results in persistent or significant disability or incapacity;
- is a congenital anomaly or birth defect;
- Any other important medical event that may not result in death, be life threatening, or require hospitalization, may be considered a serious adverse experience when, based upon appropriate medical judgement, the event may jeopardize the subject or may require an intervention to prevent one of the outcomes listed above.

SAEs have to be reported when its occurrence appears in two days after the study operations. The investigators in participating centres should inform the coordinating investigator as soon as possible but at least the next working day.

For peer review only - http://bmjopen.bmj.com/site/about/guidelines.xhtml
For peer review only

The sponsor will report the SAEs through the web portal *ToetsingOnline* to the accredited METC that approved the protocol, within 15 days after the sponsor has first knowledge of the serious adverse events.

SAEs that result in death or are life threatening should be reported expedited. The expedited reporting will occur not later than 7 days after the responsible investigator has first knowledge of the adverse event. This is for a preliminary report with another 8 days for completion of the report.

9.2.3 Suspected unexpected serious adverse reactions (SUSARs)

Not applicable.

9.3 Annual safety report

The annual safety report will be combined with the annual progress report (see chapter 12.4).

9.4 Follow-up of adverse events

All AEs will be followed until they have abated, or until a stable situation has been reached. Depending on the event, follow up may require additional tests or medical procedures as indicated, and/or referral to the general physician or a medical specialist.

SAEs need to be reported till end of study within the Netherlands, as defined in the protocol

9.5 Data Safety Monitoring Board (DSMB)

Since both techniques are standard practice, and no major unexpected complications are foreseen, no interim analysis is planned. A Data Safety and Monitoring Board will not be installed, as both procedures are regularly used and acceptable options in current clinical practice.

For peer review only

For peer review only - http://bmjopen.bmj.com/site/about/guidelines.xhtml

10. STATISTICAL ANALYSIS

The results of the study will be reported according to the CONSORT statement.

The main outcomes will be analysed and presented according the intention-to-treat principle. Since in this pragmatic trial we expect that in the pessary strategy group 40% of women will cross over to surgery an additional per protocol analyses is foreseen. This will provide more insight in the effect of surgery after pessary therapy as compared to surgery or pessary therapy alone.

We plan a subgroup analysis for the location of the prolapse: anterior prolapse versus posterior prolapse.

10.1 Primary study parameter(s)

The primary outcome, success (much or very much improvement) or no success (a little better, no change, a little worse, much worse or very much worse) on the PGI-I will be expressed in percentage point differences. Differences between the percentages will be tested using a chi-square test. A p-value <0.05 will be considered statistically significant.

10.2 Secondary study parameter(s)

The PFDI, PFIQ and PISQ-r are all interval scales. Differences between baseline and 12 and 24 months follow up will be assessed using an independent t-test when normality can be assumed, or by non-parametric tests when the data are not normally distributed. Effect sizes will be calculated to estimate the magnitude of changes.

Differences in EQ5-D scores and “ziekteverzuim” between baseline and at 3, 6, 12 and 24 months will be assessed using t-test and further incorporated in the cost-effectiveness analyses.

Imputation statistics will be used or missing data.

(Serious) adverse events will be categorized and chi-square statistics, with calculation of relative risks when appropriate, will be applied in analyses.

Prediction model

A prediction model that uses predefined variables, as potential predictors of failure of pessary therapy, will be developed using multivariable regression analysis. Missing data will be imputed.

For peer review only - http://bmjopen.bmj.com/site/about/guidelines.xhtml

Predictors for failure derived from literature are a large genital hiatus (gh > 4 cm), women being sexually active, age > 65 years, prolapse POP-Q stage 3, previous hysterectomy. If applicable, a prediction rule for the chance of failure of pessary therapy will be constructed, which could be presented as a normogram which could be used to determine the chance of failure on pessary therapy.

Internal validity will be assessed using bootstrapping techniques; shrinkage will be applied to the parameter estimates. Model performance will be assessed with discriminative capacity and calibration. Calibration will be assessed by comparing the mean predicted probability that patients failed on pessary therapy with the mean observed probability that patients failed on pessary therapy. To do so, the total cohort will be split into ten groups based on the deciles of the predicted probability. Per group the mean predicted probability will be calculated as well as the mean observed predicted probability. Discriminative capacity of the model will be assessed with receiver operation characteristics (ROC) analysis and the area under the ROC curve (AUC).

We will also look at factors that could explain failure of surgery. Our systematic review on POP and recurrent POP after surgery showed that 6 preoperative items, eg. POP stage, age, family history, preoperative incontinence, previous POP or incontinence surgery, previous hysterectomy seems to be predictive for recurrence.

Women with previous POP, incontinence surgery or previous hysterectomy are excluded from our study, leaving 4 predefined potential predictive factors. After the 2 year follow-up has been performed, we will reconsider which factors to include in a prognostic model, based on the current literature. We will select predictors from literature with the highest predictive value, where about 1 predictor could be selected for each 10 surgery failures.

Using interaction terms the effect of a differential effect in women with a higher age (>median) or a lower age (<=median), a higher (>25) or lower BMI(<=25) will be assessed for both pessary as well as surgery failures prediction.

10.3 Other study parameters

Not applicable.

For peer review only - http://bmjopen.bmj.com/site/about/guidelines.xhtml

10.4 Interim analysis (if applicable)

Not applicable, because of the non-inferiority design with low risk and the possibility of cross over.

For peer review only

For peer review only - http://bmjopen.bmj.com/site/about/guidelines.xhtml

11. ETHICAL CONSIDERATIONS

11.1 Regulation statement

This study will be conducted according to the principles of the Declaration of Helsinki (version 10, October 2013) and in accordance with the Medical Research Involving Human Subjects Act (WMO) and other guidelines, regulations and Acts.

11.2 Recruitment and consent

Women with symptomatic pelvic organ prolapse who attend the outpatient clinic will be informed about the study by the gynaecologist or nurse. After checking the in- and exclusion criteria the women will receive verbal and written information about the study. If the woman is willing to participate she is asked to sign the informed consent. All women will undergo the pessary fitting test which is part of the standard evaluation during the first visit. All women will be contacted at a minimum interval of 1 week. Those women who failed the initial fitting will be offered surgery and attend the cohort, the women with a succesfull initial fitting will be asked to enroll in the RCT. In case the woman is willing to participate but actively opts for pessary therapy she will be provided with a pessary and enter the cohort.

11.3 Objection by minors or incapacitated subjects (if applicable)

Not applicable.

11.4 Benefits and risks assessment, group relatedness

The present study carries no risks for the participant. Pessary or surgery are standard care for symptomatic pelvic organ prolapse. The benefit of the study lies in a better understanding of satisfaction and cost effectiveness.

11.5 Compensation for injury

The sponsor/investigator has a liability insurance which is in accordance with article 7, subsection 9 of the WMO.

The sponsor (also) has an insurance which is in accordance with the legal requirements in the Netherlands (Article 7 WMO and the Measure regarding Compulsory Insurance for Clinical Research in Humans of 23th June 2003). This insurance provides cover for damage to research subjects through injury or death caused by the study.

For peer review only - http://bmjopen.bmj.com/site/about/guidelines.xhtml

1. € 450.000,-- (i.e. four hundred and fifty thousand Euro) for death or injury for each subject who participates in the Research;
2. € 3.500.000,-- (i.e. three million five hundred thousand Euro) for death or injury for all subjects who participate in the Research;
3. € 5.000.000,-- (i.e. five million Euro) for the total damage incurred by the organisation for all damage disclosed by scientific research for the Sponsor as 'verrichter' in the meaning of said Act in each year of insurance coverage.

The insurance applies to the damage that becomes apparent during the study or within 4 years after the end of the study.

11.6 Incentives (if applicable)

Not applicable.

For peer review only - http://bmjopen.bmj.com/site/about/guidelines.xhtml

12. ADMINISTRATIVE ASPECTS, MONITORING AND PUBLICATION

12.1 Handling and storage of data and documents

All data will be stored and will be coded. Only the researchers will be able to link patient ID and research code. The handling of personal data complies with the Dutch Personal Data Protection Act. After cessation of the study, patient material will be stored for a maximum of 15 years.

The case report forms and questionnaires will be filled in online. The head investigator will be able to check all the completed forms and questionnaires.

12.2 Monitoring and Quality Assurance

The monitoring will be coordinated by the Staff Member Clinical Research, quality coordinator of division women and baby en will be executed by a qualified intern monitor. This person is not involved in design and output of this research. The frequency of checking will be every year. The monitoring plan is discussed in section K of the METC dossier.

12.3 Amendments

All substantial amendments will be notified to the METC.

Non-substantial amendments will not be notified to the accredited METC, but will be recorded and filed by the sponsor.

12.4 Annual progress report

The sponsor/investigator will submit a summary of the progress of the trial to the accredited METC once a year. Information will be provided on the date of inclusion of the first subject, numbers of subjects included and numbers of subjects that have completed the trial, serious adverse events/ serious adverse reactions, other problems, and amendments.

12.5 End of study report

The investigator will notify the accredited METC of the end of the study within a period of 8 weeks. The end of the study is defined as the last patient's last visit.

In case the study is ended prematurely, the investigator will notify the accredited METC within 15 days, including the reasons for the premature termination.

For peer review only - http://bmjopen.bmj.com/site/about/guidelines.xhtml

Within one year after the end of the study, the investigator/sponsor will submit a final study report with the results of the study, including any publications/abstracts of the study, to the accredited METC.

12.6 Public disclosure and publication policy

The research findings will be published in peer reviewed journals.

For peer review only

For peer review only - http://bmjopen.bmj.com/site/about/guidelines.xhtml

13. STRUCTURED RISK ANALYSIS

Not applicable because this study is a low risk study concerning standard care.

13.1 Potential issues of concern

Not applicable.

13.2 Synthesis

Not applicable.

For peer review only

14. REFERENCES

1: Slieker-ten Hove MC et al. The prevalence of pelvic organ prolapse symptoms and signs and their relation with bladder and bowel disorders in a general female population. Int Urogynecol J. 2009;20(9):1037-45
2. Tegerstedt G, Maehle-Schmidt M, Nyrén O, Hammarström M. Prevalence of symptomatic pelvic organ prolapse in a Swedish population. Int Urogynecol J Pelvic Floor Dysfunct. 2005 Nov-Dec;16(6):497-503
3: Lamers BH et al. Pessary treatment for pelvic organ prolapse and health-related quality of life: a review. Int Urogynecol J. 2011;22(6):637-44
4: Broekman BM et al. Prospective pilot study in 65 women on pessary treatment as primary treatment, unpublished data
5:Broens-Oostveen WC, Mom RM, Lagro-Janssen ALM. De genitale prolaps: behnadleing en beloop in vier huisartspraktijken. Ned Tijdschr Geneeskd 2004, 148 (29); 1444-1448
6: Withagen M et al. Sacrospinous hysteropexy or modified manchester for the treatment of pelvic organ prolapse? Int J Gynaecol Obstet. 2013; Abstract Supplement
7: Vollebregt A et al. Cost-utility analysis of trocar-guided transobturator mesh versus anterior colporrhaphy for primary cystocele repair: a randomised trial. [in preparation]
8: Barber MD[1], Walters MD, Bump RC. Short forms of two condition-specific quality-of- life questionnaires for women with pelvic floor disorders (PFDI-20 and PFIQ-7). Am J Obstet Gynecol. 2005 Jul;193(1):103-13.
9: Pollard ME, Eilber KS, Anger JT. Abdominal approaches to pelvic prolapse repairs. Curr Opin Urol. 2013;23(4):306-11.
10: Dietz V et al. Vaginal surgery for uterine descent; which options do we have? A review of the literature. Int J Gynaecol Obstet. 2009;20(3):349-56
11: C. Maher, Feiner B, Baessler K, Schmid C. Surgical management of pelvic organ prolapse in women. Cochrane 2013
12: de Boer TA, Milani AL, Kluivers KB, Withagen MI, Vierhout ME. The effectiveness of surgical correction of uterine prolapse: cervical amputation with uterosacral ligament plication (modified Manchester) versus vaginal hysterectomy with high uterosacral ligament plication. Int Urogynecol J Pelvic Floor Dysfunct. 2009 Nov;20(11):1313-9
13: Bugge C et al. Pessaries (mechanical devices) for pelvic organ prolapse in women. Cochrane database syst rev, 2013;28;2:CD004010
14: van Geelen JM, Dwyer PL. Where to for pelvic organ prolapse treatment after the FDA pronouncements? A systematic review of the recent literature. Int Urogynecol J. 2013 May;24(5):707-18
15: Abdool Z et al. Prospective evaluation of outcome of vaginal pessaries versus surgery in women with symptomatic pelvic organ prolapse. Int Urogynecol J. 2011;22(3):273-8
16: Hullfish KL, Trowbridge ER, Stukenborg GJ. Treatment strategies for pelvic organ prolapse: a cost-effectiveness analysis. Int Urogynecol J. 2011 May;22(5):507-15
17: Cundiff GW, Amundsen CL, Bent AE, Coates KW, Schaffer JI, Strohbehn K, Handa VL. The PESSRI study: symptom relief outcomes of a randomized crossover trial of the ring and Gellhorn pessaries. Am J Obstet Gynecol. 2007 Apr;196(4):405.e1-8
18: Weber MA, Limpens J, Roovers JPWR. Assessment of vaginal atrophy: a review [submitted BJOG]
19: Cobra Ok-Klapper 2012. DCHG 2012
20: Detollenaere RJ, den Boon J, Kluivers KB, Vierhout ME, van Eijndhoven HW.Surgical management of pelvic organ prolapse and uterine descent in the Netherlands. Int Urogynecol J. 2013 May;24(5):781-8
21.Detollenaere RJ, den Boon J, Vierhout ME, van Eijndhoven HW. Uterus preserving surgery versus vaginal hysterectomy in treatment of uterine descent: a systematic review]. Ned Tijdschr Geneeskd. 2011;155(42):A3623

For peer review only - http://bmjopen.bmj.com/site/about/guidelines.xhtml

22: Yalcin I, Bump RC. Validation of two global impression questionnaires for incontinence. Am J Obstet Gynecol. 2003 Jul;189(1):98-101
23: Barber MD, Kuchibhatla MN, Pieper CF, Bump RC. Psychometric evaluation of 2 comprehensive condition-specific quality of life instruments for women with pelvic floor disorders. Am J Obstet Gynecol 2001;185(6):1388–1395.
24: Utomo E, Blok BF, Steensma AB, Korfage IJ. Validation of the Pelvic Floor Distress Inventory (PFDI-20) and Pelvic Floor Impact Questionnaire (PFIQ-7) in a Dutch population. Int Urogynecol J. 2014;25(4):531-44.
25: Euroquol—a new facility for the measurement of health-related quality of life. The EuroQol Group. Health Policy. 1990;16:199-208
26: Rogers RG, Rockwood TH, Constantine ML, Thakar R, Kammerer-Doak DN, Pauls RN, Parekh M, Ridgeway B, Jha S, Pitkin J, Reid F, Sutherland SE, Lukacz ES, Domoney C, Sand P, Davila GW, Espuna Pons ME. A new measure of sexual function in women with pelvic floor disorders (PFD): the Pelvic Organ Prolapse/Incontinence Sexual Questionnaire, IUGA-Revised (PISQ-IR). Int Urogynecol J. 2013 Jul;24(7):1091-103
27: Cabana MD, Rand CS, Powe NR, Wu AW, Wilson MH, Abboud PA, Rubin HR. Why don't physicians follow clinical practice guidelines? A framework for improvement. JAMA. 1999 Oct 20;282(15):1458-65
28: Thys SD, Roovers JP, Geomini PM, Bongers MY. Do patients prefer a pessary or surgery as primary treatment for pelvic organ prolapse. Gynecol Obstet Invest. 2012;74(1):6-12
29: www.imta.nl (C. Bouwmans et al. Handleiding iMTA Medical Cost Questionnaire (iMCQ). Rotterdam: iMTA, Erasmus Universiteit Rotterdam, 2013)
30: www.imta.nl (C. Bouwmans et al. Handleiding iMTA Productivity Cost Questionnaire (iPCQ). Rotterdam: iMTA, Erasmus Universiteit, 2013)
31:Hakkaart- van Roijen, S.S. Tan, C.A.M. Bouwmans: Handleiding voor Kostenonderzoek. Methoden en standaard kostprijzen voor economische evaluaties in de gezondheidszorg. CVZ 2010
32: Oostenbrink JB, Koopmanschap MA, Rutten FF. Standardisation of costs: the Dutch Manual for Costing in economic evaluations. Pharmacoeconomics 2002;20(7):443-454
33: Mauskopf JA, Sullivan SD, Annemans L et al. Principles of good practice for budget impact analysis: report of the ISPOR Task Force on good research practices--budget impact analysis. Value Health 2007;10(5):336-347 -> In BIA, klopt niet, nog vervangen door:

References (Cost) effectiveness of pessary use as compared to surgery systematic review

1: Mamik MM, Rogers RG, Qualls CR, Komesu YM. Goal attainment after treatment in patients with symptomatic pelvic organ prolapse. Am J Obstet Gynecol. 2013 Nov;209(5):488.e1-5.
2: Abdool Z, Thakar R, Sultan AH, Oliver RS. Prospective evaluation of outcome of vaginal pessaries versus surgery in women with symptomatic pelvic organ prolapse. Int Urogynecol J. 2011 Mar;22(3):273-8.
3: Lowenstein L, Gamble T, Sanses TV, van Raalte H, Carberry C, Jakus S, Pham T, Nguyen A, Hoskey K, Kenton K; Fellow's Pelvic Research Network. Changes in sexual function after treatment for prolapse are related to the improvement in body image perception. J Sex Med. 2010 Feb;7(2 Pt 2):1023-8.
4: Barber MD, Walters MD, Cundiff GW; PESSRI Trial Group. Responsiveness of the Pelvic Floor Distress Inventory (PFDI) and Pelvic Floor Impact Questionnaire (PFIQ) in women undergoing vaginal surgery and pessary treatment for pelvic organ prolapse. Am J Obstet Gynecol. 2006 May;194(5):1492-8.

References Systematic review of factors influencing pessary fitting and continuation

For peer review only - http://bmjopen.bmj.com/site/about/guidelines.xhtml

1. Abdool Z, Thakar R, Sultan AH, Oliver RS (2010) Prospective evaluation of outcome of vaginal pessaries versus surgery in women with symptomatic pelvic organ prolapse. Int Urogynecol J Pelvic Floor Dysfunct 16
2. Alperin M, Khan A, Dubina E, Tarnay C, Wu N, Pashos CL, Anger JT.Female Pelvic Med Reconstr Surg. 2013 May-Jun;19(3):142-7.Patterns of pessary care and outcomes for medicare beneficiaries with pelvic organ prolapse.
3. Bai SW, Yoon BS, Kwon JY, Shin JS, Kim SK, Park KH (2005) Survey of the characteristics and satisfaction degree of the patients using a pessary. Int Urogynecol J Pelvic Floor Dysfunct 16 (3):182–186
4. Barber MD, Walters MD, Cundiff GW (2006) Responsiveness of the Pelvic Floor Distress Inventory (PFDI) and Pelvic Floor Impact Questionnaire (PFIQ) in women undergoing vaginal surgery and pessary treatment for pelvic organ prolapse. Am JObstet Gynecol 194(5):1492–149
5. Brincat C, Kenton K, Pat FM, Brubaker L (2004) Sexual activity predicts continued pessary use. Am J Obstet Gynecol 191(1):198–200
6. Broens-Oostveen MC, Mom RM, Lagro-Janssen AL (2004)Genital prolapse; treatment and course in four general practices.Ned Tijdschr Geneeskd 148(29):1444–1448, 17
7. Chan SS, Cheung RY, Yiu KW, Lee LL, Pang AW, Chung TK. Int Urogynecol J. 2012 Aug;23(8):1027-33. Symptoms, quality of life, and factors affecting women's treatment decisions regarding pelvic organ prolapse.
8. Clemons JL, Aguilar VC, Sokol ER, Jackson ND, Myers DL (2004) Patient characteristics that are associated with continued pessary use versus surgery after 1 year. Am J Obstet Gynecol 191(1):159–164
9. Cundiff GW, Amundsen CL, Bent AE, Coates KW, Schaffer JI, Strohbehn K et al (2007) The PESSRI study: symptom relief outcomes of a randomized crossover trial of the ring and Gellhorn pessaries. Am J Obstet Gynecol 196(4):405–408
10. Fernando RJ, Thakar R, Sultan AH, Shah SM, Jones PW (2006) Effect of vaginal pessaries on symptoms associated with pelvic organ prolapse. Obstet Gynecol 108(1):93–99
11. Friedman S, Sandhu KS, Wang C, Mikhail MS, Banks E (2010) Factors influencing long-term pessary use. Int Urogynecol J Pelvic Floor Dysfunct 21(6):673–678
12. Hanson LA, Schulz JA, Flood CG, Cooley B, Tam F (2006) Vaginal pessaries in managing women with pelvic organ prolapse and urinary incontinence: patient characteristics and factors contributing to success. Int Urogynecol J Pelvic Floor Dysfunct 17(2):155–159
13. Jones K,Yang L, Lowder JL, Meyn L, Ellison R, ZyczynskiHMet al (2008) Effect of pessary use on genital hiatus measurements in women with pelvic organ prolapse. Obstet Gynecol 112(3):630–636
14. Geoffrion R, Zhang T, Lee T, Cundiff GW.Female Pelvic Med Reconstr Surg. 2013 Nov-Dec;19(6):339-45. Clinical characteristics associated with unsuccessful pessary fitting outcomes.
15. Komesu YM, Rogers RG, Rode MA, Craig EC, Gallegos KA, Montoya AR et al (2007) Pelvic floor symptom changes incpessary users. Am J Obstet Gynecol 197(6):620–626
16. Kuhn A, Bapst D, Stadlmayr W, Vits K, Mueller MD (2009) Sexual and organ function in patients with symptomatic prolapse: are pessaries helpful? Fertil Steril 91(5):1914–1918
17. Lone F1, Thakar R, Sultan AH, Karamalis G. A 5-year prospective study of vaginal pessary use for pelvic organ prolapse. Int J Gynaecol Obstet. 2011 Jul;114(1):56-9.
18. Maito JM, Quam ZA, Craig E, Danner KA, Rogers RG (2006) Predictors of successful pessary fitting and continued use in a nurse-midwifery pessary clinic. J Midwifery Womens Health 51 (2):78–84
19. Mamik MM, Rogers RG, Qualls CR, Komesu YM. Goal attainment after treatment in patients with symptomatic pelvic organ prolapse. Am J Obstet Gynecol. 2013 Nov;209(5):488.e1-5.
20. Manchana T, Bunyavejchevin S. Impact on quality of life after ring pessary use for pelvic organ prolapse. Int Urogynecol J. 2012 Jul;23(7):873-7.

For peer review only - http://bmjopen.bmj.com/site/about/guidelines.xhtml

21. Markle D, Skoczylas L, Goldsmith C, Noblett K. Patient characteristics associated with a successful pessary fitting. Female Pelvic Med Reconstr Surg. 2011 Sep;17(5):249-52.
22. Mokrzycki ML, Hatangadi SB, Zaccardi JE, Cox S. Preexisting stress urinary incontinence: a predictor of discontinuation with pessary management. J Low Genit Tract Dis. 2001 Oct;5(4):204-7.
23. Mutone MF, Terry C, Hale DS, Benson JT (2005) Factors which influence the short-term success of pessary management of pelvic organ prolapse. Am J Obstet Gynecol 193(1):89–94
24. Nguyen JN, Jones CR. Pessary treatment of pelvic relaxation: factors affecting successful fitting and continued use. J Wound Ostomy Continence Nurs. 2005;32(4):255-61; quiz 262-3.
25. Patel M, Mellen C, O'Sullivan DM, Lasala CA. (2010) Impact ofpessary use on prolapse symptoms, quality of life, and bodyimage. Am J Obstet Gynecol 18
26. Powers K, Lazarou G, Wang A, LaCombe J, Bensinger G.Greston WM et al (2006) Pessary use in advanced pelvic organprolapse. Int Urogynecol J Pelvic Floor Dysfunct 17(2):160–164.

Reference list Systematic review of factors influencing failure of POP surgery

1. Whiteside JL, Weber AM, Meyn LA, Walters MD. Am J Obstet Gynecol. 2004 Nov;191(5):1533-8.factors for prolapse recurrence after vaginal repair.
2. Diez-Itza I, Aizpitarte I, Becerro A. Int Urogynecol J Pelvic Floor Dysfunct. 2007 Nov;18(11):1317-24. Risk factors for the recurrence of pelvic organ prolapse after vaginal surgery: a review at 5 years after surgery.
3. Fialkow MF, Newton KM, Weiss NS. Int Urogynecol J Pelvic Floor Dysfunct. 2008 Nov;19(11):1483-7.Incidence of recurrent pelvic organ prolapse 10 years following primary surgical management: a retrospective cohort study.
4. Jeon MJ, Chung SM, Jung HJ, Kim SK, Bai SW. Gynecol Obstet Invest. 2008;66(4):268-73 Risk factors for the recurrence of pelvic organ prolapse.
5. Dällenbach P, Jungo Nancoz C, Eperon I, Dubuisson JB, Boulvain M. Int Urogynecol J. 2012 Jan;23(1):35-41. Incidence and risk factors for reoperation of surgically treated pelvic organ prolapse.
6. Weemhoff M, Vergeldt TF, Notten K, Serroyen J, Kampschoer PH, Roumen FJ. Int Urogynecol J. 2012 Jan;23(1):65-71. Avulsion of puborectalis muscle and other risk factors for cystocele recurrence: a 2-year follow-up study.
7. Tegerstedt G1, Hammarström M. Acta Obstet Gynecol Scand. 2004 Aug;83(8):758-63. Operation for pelvic organ prolapse: a follow-up study.
8. Medina CA, Candiotti K, Takacs P. Int J Gynaecol Obstet. 2008 May;101(2):184-7. Wide genital hiatus is a risk factor for recurrence following anterior vaginal repair.
9. Salvatore S1, Athanasiou S, Digesu GA, Soligo M, Sotiropoulou M, Serati M, Antsaklis A, Milani R. Neurourol Urodyn. 2009;28(4):301-4. doi: 10.1002/nau.20639. Identification of risk factors for genital prolapse recurrence.
10. Kawasaki A, Corey EG, Laskey RA, Weidner AC, Siddiqui NY, Wu JM. J Reprod Med. 2013 May-Jun;58(5-6):195-9. Obesity as a risk for the recurrence of anterior vaginal wall prolaps after anterior colporrhaphy.

For peer review only - http://bmjopen.bmj.com/site/about/guidelines.xhtml

Appendix 1:

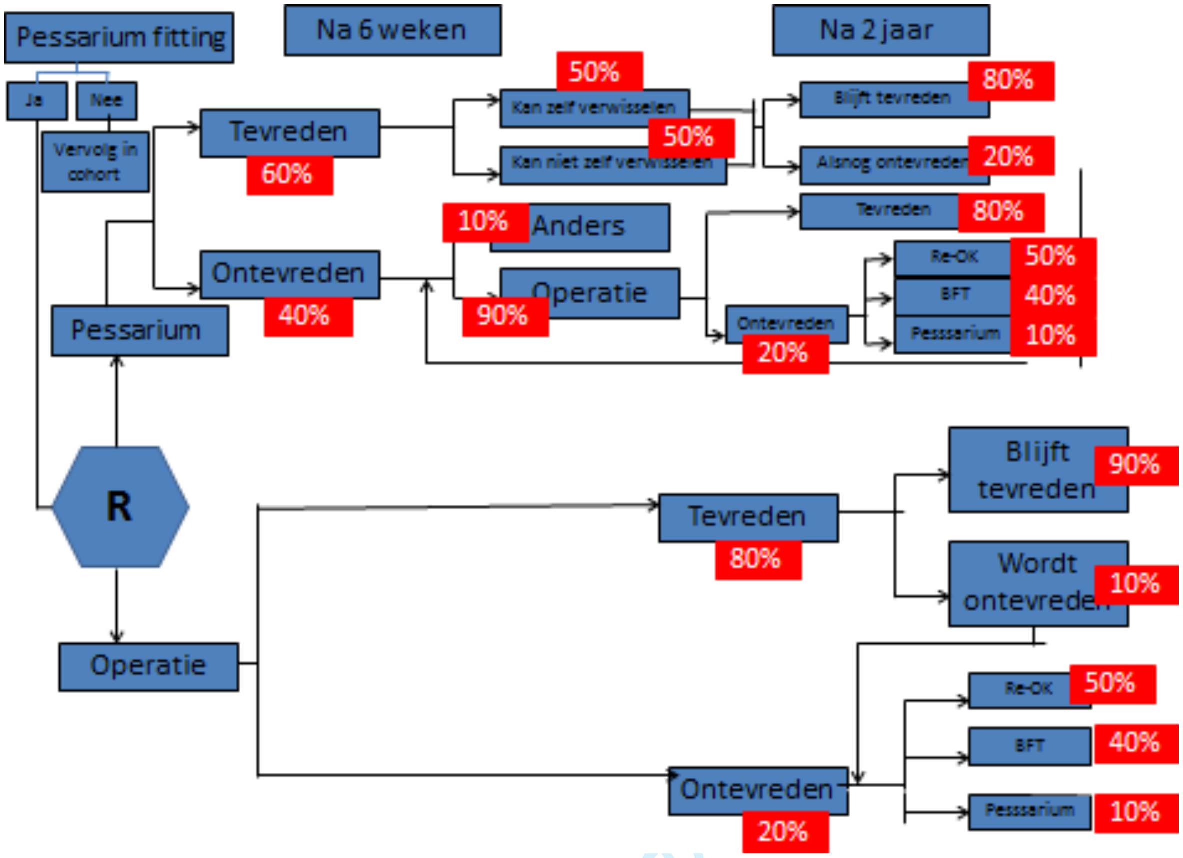

ew only

Appendix 2 Review on (Cost) effectiveness of pessary use as compared to surgery:

For peer review only

For peer review only - http://bmjopen.bmj.com/site/about/guidelines.xhtml

Reference	Study type	Characteristics	Intervention (I)	Controls (C)	Outcome measures and follow-up time	Results
Mamik, 2012<br><br>AJOG 2013:209:488	Design<br>Case-control<br><br>N = 100<br><br>Country<br>US	Aim: compare goal achievement and global improvement between pessary and surgery for POP stage ≥2.<br><br>Inclusion criteria: >18 year old, read and write in English<br><br>Exclusion: not given	Vaginal pessary<br>N = 50	Prolapse surgery<br>N = 50	Primary outcome:<br>Goal attainment<br><br>Secondary:<br>PGI-I<br>PFDI-20<br>PISQ-12<br>Body Image scale<br><br>Follow-up:<br>3 months	Primary outcome:<br>Goal attainment sign. higher score after surgery (8.6 vs 6.4)<br><br>Secondary outcomes<br>PGI-I sign (p=0.04) better improvement after surgery (2.4 vs 1.9 points)<br>PFDI-20 sign (p=0.02) higher change (89 vs 43 points)<br>PISQ-12 and BIS no sign difference<br><br>Additional: 10% crossed over from pessary to surgery within 3 months and 10% referred from surgery after they had been selected as eligible.<br>No follow-up in pessary group is 40% (20/50) and surgery 30% (15/50)
Abdool, 2011	Design<br>Cohort study<br><br>N total = 554<br><br>Country:<br>UK	Aim of the study<br>to evaluate and compare the effectiveness of pessaries and surgery in women with symptomatic pelvic organ prolapse.<br><br>Inclusion criteria<br>- Women referred to a specialist urogynaecology unit with symptomatic POP between June 2002 and May 2007<br><br>Exclusion criteria<br>- Subjects fitted with pessaries for urinary incontinence and those who had concomitant urinary incontinence surgery (e.g. TVT)<br>- Subjects who started in the pessary group but subsequently requested surgery were excluded from	Intervention<br>vaginal pessary<br><br>N = 359	Controls<br>surgery<br><br>N = 195	Primary outcomes:<br>Sheffield POP questionnaire (SPS-Q)<br><br>Secundary outcomes:<br>None<br><br>Follow up:<br>For the surgery and pessary groups 14 months (SD 6.14)<br>and 12 months (SD 3.1), respectively.	Primary outcomes:<br>No difference in functional outcome after 1 year follow-up between groups<br><br>Additional:<br>Only 45% in pessary group en 55% in surgery group responded at 12 months<br>In pessary group 24.7% (89/359) crossed to surgery but were not analyzed<br>In pessary group 7.3% stopped because of other reasons.<br>Selection and patient preference bias<br>The mean age was significantly higher in the pessary group compared to the surgery group (68.4 +/− 13.08 vs 60.4 +/−12.25 years, respectively).

For peer review only - http://bmjopen.bmj.com/site/about/guidelines.xhtml

		**analysis in both the surgery and pessary group.**				

For peer review only - http://bmjopen.bmj.com/site/about/guidelines.xhtml

Lowenstein 2010<br><br>J Sex Med 2010; 7: 1023-<br><br>28	Design<br>Cohort study<br>N= 235<br><br>Country<br>US	Aim of the study<br>First to evaluate patient-reported outcome, POP symptoms, sexual functioning and body image following treatment of POP. Second to compare surgery with pessary<br><br>Inclusion: ≥18 year, ≥ satge 2 POP, complete questionnaire at baseline and at ≥6 months follow up<br><br>Exclusion: recurrent UTI, peripheral neuropathy, using pessary at initial presentation or POP surgery < 6 months prior to presentation	Intervention<br>N = 202 surgery	Controls<br>N = 33 pessary	Primary outcomes<br>PFDI-20<br>PISQ-12<br>Modified Body Image scale<br><br>All at six months follow-up	Results<br>After multivariate analyses, including type of intervention, BMI and difference in Body image were associated with change in total PISQ (sexual functioning) score<br><br>In the pessary group there was no significant improvement in sexual functioning as compared to surgery (-2.5 versus +11.5)<br><br>Additional:<br>No figures presented for pessary and surgery group, with exemption of the Sexual functioning (PISQ-12) result above.
Barber, 2006	Design<br>Case-control study<br><br>N total = 106<br><br>Country: USA	Aim of the study<br>to evaluate the responsiveness of the Pelvic Floor Distress Inventory (PFDI) and Pelvic Floor Impact Questionnaire (PFIQ) in women with pelvic organ prolapse undergoing surgical and nonsurgical management.<br><br>Inclusion criteria<br>Surgery group:<br>Stage III or IV prolapse, were at least 18 years, and scheduled for vaginal prolapse repair.<br>Pessary group:<br>women with symptomatic pelvic organ prolapse of stage II or greater. (Pessri trial)<br><br>Exclusion criteria<br>Surgery group:<br>- mentally or physically incapable of completing the questionnaires.<br>Pessary group:<br>- were pregnant, were currently using a pessary, or had vaginal agglutination	Intervention<br>Pessary in women with stage II or greater POP<br><br>N = 42	Controls<br>Surgery in women with stage III or greater POP<br><br>N = 64	Primary outcomes:<br>PFDI and PFIQ<br><br>Secundary outcomes:<br><br>Follow up:<br>3 months (Pessary group) or 6 months (Surgery group) after initiation of treatment.	Primary outcomes:<br><br>After controlling for preoperative prolapse stage and baseline HRQOL scores, subjects in the Surgery group had significantly greater improvement in each of the scales of the PFDI and the prolapse and urinary scales of the PFIQ than did the Pessary group.<br><br>Scores from each of the scales of the PFDI improved by 14 to 15 points more on average after treatment in the Surgery group than those of the Pessary group (P < .01 for each) after adjusting for the above baseline differences.<br><br>Similarly, for the prolapse and urinary scales of the PFIQ, scores improved 13 and 17 points more, respectively, in the Surgery group than the Pessary group after treatment. (P < .05 for each).<br><br>Four of 64 (6%) of subjects in the Surgery group had recurrent prolapse develop beyond the hymen by 6 months after surgery. No subjects underwent reoperation for recurrent prolapse during the study period.<br><br>Additional:<br><br>Difference in follow up<br>Selection bias

For peer review only - http://bmjopen.bmj.com/site/about/guidelines.xhtml

		**that precluded pessary insertion.**				

For peer review only - http://bmjopen.bmj.com/site/about/guidelines.xhtml

Appendix 3 Review on risk factors for failure of pessaries:

study		n	FU period	success initial	continued pes	stopped pes	cross over surgery	predict fit	predict contin
Wu 1997	retrospective	110	3 yrs	81 (74%)	43 (39%)		at least 5	nee	ja
Mokrzycki 2001	retrospective	42	3 mth		24 (57%)	18 (43%)	nr	nee	ja
Clemons 2004	prospective	100	1 yr	73 (73%)	43 (43%)		16/73 (27%)	nee	ja
Brincat 2004	retrospective	169	2,5 yr	144 (85%)	82 (60%)	54 (40%)	32/144 (22%)	nee	ja
Mutone 2004	retrospective	407	3 wks	168 (41%)		96 (57%)	nr	ja	ja
Powers 2006	retrospective	32	1 yr			20 (62%)	12 (38%)	nee	ja
Broens-Oostveen 2004	retrospective	192	9 yr			107 (52%)	nr	nee	ja
Nguyen 2005	retrospective	130	1 yr	111 (82%)	85 (65%)		20 (15%)	ja	ja
Hanson 2005	retrospective	1216	3 mth	1043 (86%)	744 (71%)		nr	nee	ja
Fernando 2006	prospective	203	4 mth	nr	153 (75%)		28 (14%)	nee	ja
Barber 2006	prospective	42	3 mth	nr		0	nr	nee	nee
Maito 2006	retrospective	120	6 mth	106 (88%)	92 (77%)		9 (8%)	ja	ja
Cundiff 2007	prospective	85	1 yr	65/71 (92%)			5 (6%)	nee	nee
Komesu 2007	prospective	64	6-12 mth		36 (56%)	28 (44%)	2 (3%)	nee	ja
Jones 2008	prospective	90	3 mth		42 (47%)		nr	nee	ja
Kuhn 2009	prospective	71	3 mth		32 (45%)		nr	nee	nee
Sarma 2009	retrospective	120	1 mth		45 (38%)		18 (15%)	nee	nee
Friedman 2010	retrospective	150	1 yr		115 (77%)	35 (23%)	25 (17%)	nee	ja
Patel 2010	prospective	65	3 mth	54 (83%)	54 (83%)		5 (8%)	nee	nee
Abdool 2010	prospective	359	1 yr	296 (83%)	243 (68%)		31 (9%)	nee	nee
Lone 2011	prospective	246	5 yr	187 (76%)	130 (53%)		70 (28%)	nee	nee
Markle 2011	retrospective	158	1 week	92 (59%)			nr	ja	nee
Chan 2012	prospective	197	1 yr	138 (70%)		59 (30%)	nr	nee	ja
Manchana 2012	retrospective	126	1 yr	77 (61%)	64 (51%)		5 (4%)	nee	nee
Mamik 2013	prospective	50	3 mth				5 (10%)	nee	nee
Geoffrion 2013	retrospective	101	4 wks	79 (78%)			nr	nee	ja
Alperin 2013	retrospective	4019	9 years				12% 1 yr, 24% 9 yrs	nee	nee
Succesfull fitting		3589		2714(76%)					
Continuation		3381			2027 (60%)				

nly

For peer review only - http://bmjopen.bmj.com/site/about/guidelines.xhtml

Appendix 4 Review on risk factors for failure of surgery:

Risk factor	Investigated:	Significant:
Preoperative stage	8	5
Age	8	2
Obesity	7	0
Parity	5	0
Constipation	5	0
Pulmonary disease	5	0
Number of sites involved preoperative	4	1
Menopausal status	4	0
Hysterectomy status	4	0
Concomitent surgery	3	1
Family history	3	1
Complicated delivery	3	0
Diabetes	3	0
Smoking	3	0
Previous incontinence and/or prolapse surgery	2	2
Hiatus genitalis	2	1
Weight	2	1
Any incontinence preoperative	2	1
Delivery mode	2	0
Vaginal delivery	2	0
Hormone replacement therapy	2	0
Previous prolapse surgery	2	0
Surgeons experience	2	0
Abcense of posterior repair	1	1
Sexual activity	1	1
Levator defect	1	1
Height	1	0
Birth weight	1	0
Age at last delivery	1	0
Site of most advanced prolapse	1	0
Surgical approach	1	0
Use of Mesh	1	0
Previous incontinence surgery	1	0
Previous pelvic floor surgery or hysterectomy	1	0
Abdominal hernias	1	0
Cardiovascular disease	1	0
Intense physical exercise	1	0
Heavy lifting	1	0
Heavy lifting or constipation	1	0
Levator muscle contraction	1	0
Weight of the uterus	1	0
Postoperative complications	1	0
Incomplete emptying of bladder	1	0
Fecal incontinence	1	0

For peer review only

Study protocol

Appendix 5 tabel bezoeken, tijdstippen, onderzoeken

Chirurgie en cohort

Contact	Bezoek arts	POPQ	Eq5d<br>doktersbezoek<br>werkverzuim	PFIQ<br>PFDI<br>PISQ<br>PGII<br>PGIS
1. Eerste bezoek	X	X	Eq5D	X<br>(zonder PGII)
2. 6 weken	X	X		
3. 3 maanden			X	
4. 6 maanden			X	
5. 12 maanden	X	X	X	X
6. 24 maanden	X	X	X	X

Pessarium met zelfmanagement

Contact	Bezoek arts	POPQ	Eq5d<br>doktersbezoek<br>werkverzuim	PFIQ<br>PFDI<br>PISQ<br>PGII<br>PGIS
1. Eerste bezoek	X	X	Eq5D	X<br>(zonder PGII)
2. 6 weken	X	X		
3. 3 maanden			X	
4. 6 maanden			X	
5. 12 maanden	X	X	X	X
6. 24 maanden	X	X	X	X

For peer review only

Pessarium zonder zelfmanagement

Contact	Bezoek arts	POPQ	Ringcontrole	Eq5d doktersbezoek werkverzuim	PFIQ PFDI PISQ PGII PGIS
1. Eerste bezoek	X	X		Eq5D	X (zonder PGII)
2. 6 weken	X	X			
3. 3 maanden				X	
4. 4 maanden	X		X		
5. 6 maanden				X	
6. 8 maanden	X		X		
7. 12 maanden	X	X	X	X	X
8. 16 maanden	X		X		
9. 20 maanden	X		X		
10. 24 maanden	X	X	X	X	X

For peer review only

1.2 Final study protocol

Pessary or surgery for symptomatic pelvic organ prolapse

Version 1.~~21~~ 22 ~~April 2017~~February 2018

For peer review only

Study protocol

PROTOCOL TITLE 'Pessary or surgery for symptomatic pelvic organ prolapse'

**Protocol ID**	**~~2017~~ 2018 / 1.~~21~~22**
**Short title**	**Pessary or surgery for symptomatic pelvic organ prolapse**
**EudraCT number**	***Not applicable***
**Version**	**1.~~21~~22**
**Date**	**April 2017**
**Coordinating investigator/project leader**	**Prof. Dr. C.H. van der Vaart, gynaecologist**<br>**University Medical Centre Utrecht**
**Principal investigator(s) (in Dutch: hoofdonderzoeker/ uitvoerder)**	**Dr. A. Vollebregt, gynaecologist**<br>**Spaarne Hospital**<br><br>**M.K. van de Waarsenburg, MD**<br>**University Medical Centre Utrecht**
**Multicenter: per site**	**Prof. Dr. C.H. van der Vaart, UMC Utrecht / Bergman Clinics Bilthoven**<br>**Dr. A.L. Milani, Reinier de Graaf Gasthuis**<br>**Dr. A. Vollebregt, Spaarne Hospital Hoofddorp**<br>**Dr. J.P.W.R. Roovers, Academic Medical Center / Bergman Clinics Amsterdam**<br>**Dr. K.B. Kluivers, Radboud UMC Nijmegen**<br>**Dr. V. Dietz, Catharina Hospital Eindhoven**<br>**Dr. H.W.F. van Eijndhoven, Isala Clinic Zwolle**<br>**Dr. M.M.A. Vernooij, Sint Antonius Hospital**

For peer review only

For peer review only - http://bmjopen.bmj.com/site/about/guidelines.xhtml

	**Nieuwegein**<br>**Dr. R.P. Schellart, Kennemer Gasthuis Haarlem**<br>**Drs. A.M.W. Broekman, Sint Franciscus Gasthuis Rotterdam**<br>**Drs. J. van Bavel, Amphia Hospital Breda**<br>**Dr. M. Weemhoff, Atrium MC Heerlen**<br>**Dr. ~~G. Link~~W.A. Spaans, Academisch ziekenhuis Maastricht**<br>**Dr. J.M. van der Ploeg, Martini ziekenhuis Groningen**<br>**Dr. D. Massop-Helmink, MST Enschede**<br>**Dr. J.M. Sikkema, ZGT Almelo / Hengelo**<br>**Dr. L. van der Voet, Deventer ziekenhuis**<br>**Dr. J.P. de Bruin, Jeroen Bosch ziekenhuis**<br>**Dr. C. Zeijl, Amstelland ziekenhuis**<br>**Dr. G.G. Bon, Tergooi ziekenhuis**<br>**Dr. S. de Vos – Stekelenburg, Albert Schweitzer ziekenhuis**<br>**Drs. K.L. Bos, Canisius Wilhelmina ziekenhuis**<br>**Prof. Dr. M.Y. Bongers – Maxima Medisch Centrum**<br>**Dr. W. Hermes – MCH-Bronovo**<br>**Dr. E. Janszen – OLVG**<br>**Dr. T. Huisman - HAGA**
**Sponsor (in Dutch: verrichter/opdrachtgever)**	**Prof. Dr. C.H. van der Vaart, gynaecologist**<br>**University Medical Centre Utrecht**
**Subsidising party**	**ZonMw Project nr 837002525**
**Independent expert (s)**	**Dr. R.P. Zweemer**<br>**University Medical Centre Utrecht**

For peer review only

**Laboratory sites <*if applicable*>**	***Not applicable***
**Pharmacy <*if applicable*>**	***Not applicable***

For peer review only

PROTOCOL SIGNATURE SHEET

Name	Signature	Date
**Head of Department:**<br><br>**Prof. Dr. B. Veersema**<br>**Department of Reproductive Medicine and Gynaecology**<br>**University Medical Centre Utrecht**		
**Coordinating Investigator/Project leader:**<br><br>**Prof. Dr. C.H. van der Vaart, gynaecologist**		
**M.K. van de Waarsenburg, MD**<br>**University Medical Centre Utrecht**		

For peer review only

TABLE OF CONTENTS

1. INTRODUCTION AND RATIONALE 64
Problem definition 64
Relevance 65
2. OBJECTIVES 68
3. STUDY DESIGN 69
4. STUDY POPULATION 70
4.1 Population (base) 70
4.2 Inclusion criteria 70
4.3 Exclusion criteria 70
4.4 Sample size calculation 70
5. TREATMENT OF SUBJECTS 72
5.1 Investigational product/treatment 72
5.2 Use of co-intervention (if applicable) 73
5.3 Escape medication (if applicable) 73
6. INVESTIGATIONAL PRODUCT 74
6.1 Name and description of investigational product(s) 74
6.2 Summary of findings from non-clinical studies 74
6.3 Summary of findings from clinical studies 74
6.4 Summary of known and potential risks and benefits 75
6.5 Description and justification of route of administration and dosage 75
6.6 Dosages, dosage modifications and method of administration 75
6.7 Preparation and labelling of Investigational Medicinal Product 75
6.8 Drug accountability 75
7. NON-INVESTIGATIONAL PRODUCT 76
7.1 Name and description of non-investigational product(s) 76
7.2 Summary of findings from non-clinical studies 76
7.3 Summary of findings from clinical studies 76
7.4 Summary of known and potential risks and benefits 76
7.5 Description and justification of route of administration and dosage 76
7.6 Dosages, dosage modifications and method of administration 76
7.7 Preparation and labelling of Non Investigational Medicinal Product 76
7.8 Drug accountability 76
8. METHODS 77
8.1 Study parameters/endpoints 77
8.1.1 Main study parameter/endpoint 77
8.1.2 Secondary study parameters/endpoints (if applicable) 77
8.1.3 Other study parameters (if applicable) 77
8.2 Randomisation, blinding and treatment allocation 77
8.3 Study procedures (see also appendix 5) 78

For peer review only - http://bmjopen.bmj.com/site/about/guidelines.xhtml

8.4 Withdrawal of individual subjects 82
8.4.1 Specific criteria for withdrawal (if applicable) 82
8.5 Replacement of individual subjects after withdrawal 82
8.6 Follow-up of subjects withdrawn from treatment 82
8.7 Premature termination of the study 82
9. SAFETY REPORTING9.1 Section 10 WMO event 83
9.2 AEs, SAEs and SUSARs 83
9.2.1 Adverse events (AEs) 83
9.2.2 Serious adverse events (SAEs) 83
9.2.3 Suspected unexpected serious adverse reactions (SUSARs) 84
9.3 Annual safety report 84
9.4 Follow-up of adverse events 84
9.5 Data Safety Monitoring Board (DSMB) 84
10. STATISTICAL ANALYSIS 85
10.1 Primary study parameter(s) 85
10.2 Secondary study parameter(s) 85
10.3 Other study parameters 87
10.4 Interim analysis (if applicable) 87
11. ETHICAL CONSIDERATIONS 88
11.1 Regulation statement 88
11.2 Recruitment and consent 88
11.3 Objection by minors or incapacitated subjects (if applicable) 88
11.4 Benefits and risks assessment, group relatedness 88
11.5 Compensation for injury 88
11.6 Incentives (if applicable) 89
12. ADMINISTRATIVE ASPECTS, MONITORING AND PUBLICATION 90
12.1 Handling and storage of data and documents 90
12.2 Monitoring and Quality Assurance 90
12.3 Amendments 90
12.4 Annual progress report 90
12.5 End of study report 90
12.6 Public disclosure and publication policy 91
13. STRUCTURED RISK ANALYSIS 92
13.1 Potential issues of concern 92
13.2 Synthesis 92
14. REFERENCES 93
Appendix 1: 97
Appendix 2 98
Appendix 3: 102
Appendix 4 103
Appendix 5: 104

For peer review only - http://bmjopen.bmj.com/site/about/guidelines.xhtml

LIST OF ABBREVIATIONS AND RELEVANT DEFINITIONS

ABR	ABR form, General Assessment and Registration form, is the application form that is required for submission to the accredited Ethics Committee (In Dutch, ABR = Algemene Beoordeling en Registratie)
AE	Adverse Event
AR	Adverse Reaction
CA	Competent Authority
CCMO	Central Committee on Research Involving Human Subjects; in Dutch: Centrale Commissie Mensgebonden Onderzoek
CV	Curriculum Vitae
DSMB	Data Safety Monitoring Board
EU	European Union
EudraCT	European drug regulatory affairs Clinical Trials
GCP	Good Clinical Practice
IB	Investigator's Brochure
IC	Informed Consent
IMP	Investigational Medicinal Product
IMPD	Investigational Medicinal Product Dossier
METC	Medical research ethics committee (MREC); in Dutch: medisch ethische toetsing commissie (METC)
(S)AE	(Serious) Adverse Event
SPC	Summary of Product Characteristics (in Dutch: officiële productinfomatie IB1-tekst)
Sponsor	The sponsor is the party that commissions the organisation or performance of the research, for example a pharmaceutical company, academic hospital, scientific organisation or investigator. A party that provides funding for a study but does not commission it is not regarded as the sponsor, but referred to as a subsidising party.
SUSAR	Suspected Unexpected Serious Adverse Reaction
Wbp	Personal Data Protection Act (in Dutch: Wet Bescherming Persoonsgevens)
WMO	Medical Research Involving Human Subjects Act (in Dutch: Wet Medisch-wetenschappelijk Onderzoek met Mensen

For peer review only - http://bmjopen.bmj.com/site/about/guidelines.xhtml

SUMMARY

Rationale: Moderate to severe pelvic organ prolapse symptoms can be treated with pessary or surgery. Both treatments appear to be effective, but have not been compared directly.

Hypothesis: The strategy of pessary as initial therapy is as effective as direct surgery for moderate to severe POP, but it is associated with lower costs.

Objective: The primary objective is to compare the effectiveness and cost-effectiveness of pessary versus surgery as initial treatment for moderate to severe symptomatic pelvic organ prolapse (POP) in women at two year after initiation of treatment. The secondary objective is the development of a prediction model for failure of pessary use and surgery within 2 years.

Study design: Cohort study with embedded randomized controlled trial.

Study population: Treatment naïve women with POP who present with moderate to severe symptoms.

Intervention (if applicable): Pessary therapy or vaginal POP surgery.

Main study parameters/endpoints:

Primary outcome: Global impression of improvement of POP symptoms at 24 months measured with PGI-I

Secondary outcomes:

- Changes in symptom bother and disease-specific quality of life at 12 and 24 months follow-up
- Changes of sexual function at 12 and 24 months follow-up
- Changes in general quality of life at 3, 6, 12 and 24 months of follow up
- Adverse events/complications related to both treatment strategies during the study period
- Development of prediction model to identify factors for failing of pessary and surgery.
- Costs-effectiveness analyses

Nature and extent of the burden and risks associated with participation, benefit and group relatedness: Both treatment arms are routine treatments in the Netherlands. Patients in the RCT can have the risks of surgery instead of the risks from pessary therapy.

For peer review only - http://bmjopen.bmj.com/site/about/guidelines.xhtml

1. INTRODUCTION AND RATIONALE

Problem definition

Female pelvic organ prolapse (POP) is a common problem in women that negatively affects quality of life. The estimated prevalence of symptomatic POP among women between 45-85 years of age is 8.3 - 11% [1,2]. It is current practice in the Netherlands that the general practitioner (GP) treats the majority of women with POP symptoms. Women with moderate to severe POP symptoms are often referred to a gynecologist for treatment. This study focuses at the subgroup of moderate to severe POP.

Known effective treatment options for moderate to severe POP are pessary or surgery. A pessary has proven its effectiveness in the treatment of symptomatic POP, mainly in cystocele and uterine descent. However, studies are mainly observational in nature and inherently subject to selection and indication bias [3]. In literature, outcomes of pessary therapy are mainly recorded in terms of (dis-) continuation of therapy and to a much lesser extent in terms of symptom relief. The pessary continuation rate is 60% [3]. This is confirmed by a Dutch pilot study in 65 women that showed a satisfaction with pessary in 57% of womenand an operation rate of 43% at 12 months follow up [4]. In this study, 80% of women who continued pessary therapy reported much to very much improvement of their POP symptomsat 1 year follow up [4]. Reasons of discontinuation are pressure ulcer, vaginal discharge, discomfort or loss of fitting. These complications are reported to occur in up to 53% of women [5]. Half of them will decide to stop using pessary, but it is unclear which characteristics predict this outcome. Check-up of pessary therapy can be performed by either a general practitioner (GP), gynecologist or by self-management. According to a recent survey 50% percent of gynecologists involved in urogynaecology always offer self- management 40% on indication, and 10% never. Pessary therapy is inexpensive and costs are mainly related to doctor visits and treatment of side effects. In case of self-management costs might even be lower,

Surgery for POP results in much to very much improvement of symptoms in 80% of women and improvement of quality of life [6-9]. An anterior colporraphia is considered the standard procedure for a cystocele, as is the posterior colporraphia for a rectocele. For uterinedescent uterus sparing techniques, like sacrospinous hysteropexy (SH) and modified Manchester-Fothergill procedure, or vaginal hysterectomy can be performed [10-12]]. Complications of POP surgery are temporary urinary retention, temporary buttock pain in case of sacrospinous hysteropexy, urinary tract infection, hematoma or dyspareunia [11]. These complications seldom lead to persistent morbidity. The most

For peer review only - http://bmjopen.bmj.com/site/about/guidelines.xhtml

common “complication” is the recurrence of symptomatic POP or de novo stress- incontinence that may lead to additional surgery, pessary therapy, or pelvic floor physiotherapy. As part of a RCT, comparing mesh with fascia plication, we found that
11% of women needed additional surgery after anterior colporraphia at 24 months follow up [7,9]. As in pessary therapy, the characteristics that predict successful or unsuccessfulsurgical therapy are largely unknown.

The decision which treatment option to choose depends on both patient and doctor’s preferences. In our pilot survey 70% of gynecologists informed their patients about the possibility of pessary therapy, but it is unknown how many women actually received a pessary. A recent Dutch study showed that 48% of treatment-naive women preferred surgery, 36% a pessary and 16% had no preference [28]. It is therefore reasonable to assume that at least 50% of treatment naïve women with moderate to severe prolapse symptoms will have surgery as primary treatment.

Although clinical efficacy appears to favor surgery [3], the large variation in study design, outcome measurements and loss to follow up makes any comment on the best treatment option speculative. This is recognized in two recent reviews on the subject that both urge the need for randomized trials comparing surgery and pessary for POP [13,14]. Efficacy can be expressed in terms of clinical outcome but also in terms of cost-effectiveness. It is obvious that surgery (especially hospital costs) is much more expensive than pessary therapy, butthe cost-effectiveness of the surgical or pessary strategy has never been assessed. Based on current cohort and case-control studies we hypothesize that a strategy of initial pessary therapy for moderate to severe POP, is more cost-effective than surgery.
We propose to perform a randomized controlled trial to generate evidence for the optimal and most cost-effective primary treatment for moderate to severe POP, including a better a priori patient selection for treatment by identifying factors of failure for pessary therapy or surgery.

Relevance
At present a national multidisciplinary guideline on the diagnosis and treatment of POP is completed. The guideline identifies the lack of evidence with respect to the best treatment option for moderate to severe prolapse, a conclusion that is confirmed by the 2013 Cochrane Collaboration review [13]. In this evidence “vacuum” both doctors and patient preferences rule, but unfortunately these are not supported by facts. If we look at the available data the following

For peer review only - http://bmjopen.bmj.com/site/about/guidelines.xhtml

calculation emerges.

About 60% of women who start pessary therapy in the specialist care setting will continue using it at one year [4,15]. Eighty percent of them will report much to very much improvement, resulting in an overall 48% much to very much improvement. The majority ofthe 40% of women who are not satisfied with pessary therapy will request or are offered additional surgery. After surgery 80% of women report much to very much improvement ofPOP symptoms [6]. Combining these percentages, women who originally started withpessary therapy may also expect 80% (48% after initial pessary treatment + 32% afteradditional surgery =) much to very much improvement. Based on these estimates it isexpected that the outcome of both treatment strategies will eventually result in a globalimprovement of symptoms in 80% of women. With equal clinical outcomes of both strategiesthe costs needed to obtain these outcomes become crucial. With the exception of a cost calculation based on a Markov model, no direct cost-effectiveness studies on the use ofpessary or surgery for POP have been performed [16]. The relevance of this project, with the high prevalence of POP worldwide, associated costs and insufficient evidence, is high. Wehave searched the www.clinicaltrials.gov database (3th March 2014) on similar studies(comparing pessary with surgery) but none were found.

However, if we were to prove that pessary therapy is more cost-effective then surgical treatment, this does not imply that a trial of pessary should always be undertaken. There is also insufficient evidence on which patient characteristics are associated with failure of pessary treatment or surgery (systematic review). The knowledge on how to predict which women will have a very low chance of success with pessary therapy can further improve effective treatment strategy management. This will contribute to treatment efficacy. This is not only very relevant for the hospital specialist care setting, but this knowledge can also be implemented in general practitioner practice units.

There is very limited evidence on the optimal management strategy for pessary cleaning, both in time interval as well as in who should perform the cleaning. Our study is unique and therefore relevant since self-management is advocated in the study setting. This will not only allow it to obtain data in a standardized way, but also involves the woman in her own management. This involvement is strongly advocated by two major gynecologic patient organizations ('Patienten Gynaecologie Nederland' and the 'Stichting Bekkenbodem Patienten'). These two organizations, as well as the Dutch urogynaecological consortium have identified this study to be highly relevant.

For peer review only - http://bmjopen.bmj.com/site/about/guidelines.xhtml

In line with the report "Medisch Specialistische zorg 20/20" we are heading towards integral health care in which the general physician and medical specialist will work more closely together, using the same treatment protocol for various illnesses. The information and conclusions of this trial will add level I scientific evidence to such an integral protocol and guideline for women with symptomatic POP. This will aid in a better patient selection that will need referral to the specialist. The data on patient's self-management of pessary treatment will supply information for patient instructions, which are relevant for information leaflets on the subject.

For peer review only

For peer review only - http://bmjopen.bmj.com/site/about/guidelines.xhtml

2. OBJECTIVES

The aim of this multicenter pragmatic cohort study with embedded randomized controlled non-inferiority trial comparing pessary therapy versus surgery is twofold:

1. To prospectively compare the effectiveness and cost-effectiveness of pessary therapy or surgery as primary treatment of moderate to severe symptomatic cystocele, uterine descent and/or rectocele in women at two year after initiation of treatment, in randomized trial embedded in a preference cohort.
2. To compare the effectiveness between the cohort and randomized trial.
3. To develop a prediction model for failure of pessary use and surgery within the first years.

For peer review only - http://bmjopen.bmj.com/site/about/guidelines.xhtml

3. STUDY DESIGN

Multicenter pragmatic cohort study with an embedded randomized controlled non-inferiority trial comparing pessary [CE 0086] therapy and surgery including an economic evaluation. The follow up will be 24 months.

A short (30 minutes) trial of pessary fitting is part of our protocol. This ensures that only women who fit both treatment options enter the randomization procedure. The trial is short and only aims at fitting, not symptom relief. Women with an unsuccessful pessary fitting will be followed in the cohort fitting failure. In case the woman is willing to participate but actively opts for one of both treatments, she will be followed in a cohort.

See also appendix 1 and 5.

For peer review only

4. STUDY POPULATION

4.1 Population (base)

All women with a symptomatic POP will be included.

4.2 Inclusion criteria

In order to be eligible to participate in this study, a subject must meet all of the following criteria:

1. Women with a prolapse stage 2 or more.
2. Women with moderate to severe POP symptoms. Moderate to severe POP symptoms is defined as a prolapse domain score > 33 on the validated Dutch version of the Pelvic Floor Distress Inventory (PFDI-20) [8, 23, 24].
3. For the RCT: Women who have had a successful pessary fitting procedure.
4. Written informed consent.

4.3 Exclusion criteria

A potential subject who meets any of the following criteria will be excluded from participation in this study:

1. Prior urogynaecological (prolapse or incontinence) surgery
2. Probability of future childbearing
3. Insufficient knowledge of the Dutch language
4. Co-morbidity causing increased surgical risks at the discretion of the surgeon
5. Major psychiatric illness
6. Prior pessary use

4.4 Sample size calculation

With 198 women per group, we will have 80% power to reject the null hypothesis that pessary therapy is inferior to surgery, with a 1-sided alpha of 0.05, a non-inferiority margin of 10% and the proportion in the standard group is 80% (NQueryAdvisor). Accounting for 10% loss to follow-up we plan to randomize 436 patients.

The sample size calculation for prediction models is based on the number of failures of pessary or surgical therapy. For each potential predictor in the model we need 10-15 failures. Our pessary group sample size is 198 women. An estimated 40% (80 women) will cross over to surgery and can be regarded as failures. Our sample size is therefore sufficient

For peer review only - http://bmjopen.bmj.com/site/about/guidelines.xhtml

to develop the prediction model for failure of pessary therapy for 6 to 8 items. In the surgery group 20% of women will not be satisfied with the result of treatment. With the same sample size of 198 women, the 40 women who are dissatisfied allow us to study up to 4 potential predictive factors.

In the cohort we include all patients who are willing to collaborate on this research but have a preference for one of both therapies. We now assume that 70% of the eligible patients object participation in the RCT, and that 90% of them is nevertheless willing to participate in the cohort.

For peer review only

5. TREATMENT OF SUBJECTS

5.1 Investigational product/treatment

Pessary [CE 0086] therapy and surgery both are options for the treatment of a symptomatic POP. Ten large urogynecological units (university hospitals or teaching hospitals) that have worked together in previous consortium studies will participate in this multicenter trial. All participating gynaecologists have fitted at least 100 pessaries and performed more than 100 surgical POP procedures prior to the start of this study.

All pessaries are made of modern silicon material. All types of pessaries, both supportive and occlusive/space filling are allowed according to the judgment of the gynaecologist. A recent randomized trial comparing supportive (ring) and occlusive (Gelhorn) showed no differences [17]. After placing the pessary, all women will receive verbal and written instructions on the self-management of pessary therapy.

The first pessary follow up visit will always be performed by the gynaecologist. In case of self-management the frequency of cleaning is left to her personal judgment, but may not exceed 4 months. If self-management is not possible, women will be seen at 4 months intervals for pessary cleaning and vaginal inspection, preferable by their GP. In case of vaginal atrophy topical estrogens will be advised according to pharmaceutical guidelines. The diagnosis of atrophy is left to the judgment of the treating physician, since no clear definition for atrophy is available yet [18].

All surgical procedures will be performed according to our national guidelines. In this pragmatic trial the decision which technique to use is left, to the discretion of the gynaecologist, within the limitations below [19]. Cystocele repair will consist of conventional anterior colporrhaphy [9]. For uterine descent different techniques are allowed [20]. These techniques can either be uterus sparing (sacrospinous hysteropexy [10], modified Manchester-Fothergill procedure [12] or a abdominal sacrocolpopexy [9]) or a vaginal hysterectomy. Recent studies showed similar effectiveness on both anatomical and functional outcomes for these different techniques [10, 12, 21]. A coexistent stage 2 rectocele repair will be a conventional colporrhaphia posterior. All procedures are performed under general or spinal anesthesia and under antibiotics and thrombosis prophylaxis according to local protocols.

For peer review only - http://bmjopen.bmj.com/site/about/guidelines.xhtml

5.2 Use of co-intervention (if applicable)

Not applicable.

5.3 Escape medication (if applicable)

Not applicable.

For peer review only

For peer review only - http://bmjopen.bmj.com/site/about/guidelines.xhtml

6. INVESTIGATIONAL PRODUCT

6.1 Name and description of investigational product(s)

6.2 Summary of findings from non-clinical studies

Not applicable.

6.3 Summary of findings from clinical studies

Three systematic reviews of the literature were performed by four members of our Dutch urogynaecology consortium (details in appendix 2-4) that concluded:

1. Systematic review on the (cost)effectiveness of pessary use compared to surgery

There are a very limited number of comparative studies on the efficacy of surgery or pessary use for POP. The differences in study population, inclusion criteria, follow-up period, large numbers of loss to follow-up, different outcome measures makes interpretation difficult if not impossible. The two studies that presented data on functional outcome in terms of prolapsed symptom reduction were favorable for surgery (appendix 2).

2. Systematic review of factors influencing pessary fitting and continuation

A systematic review was performed to identify the satisfactory pessary fitting rate and the continuation rate of pessary use. The factors influencing these rates as well as the cross over to prolapse surgery were identified from previous studies (appendix 3).

Summarizing the results show that an estimated 75% of women will have a successful fitting and 59% will continue pessary use at variable follow-up between 3 months and 5 years. In these 18 studies, 8 factors have been tested more than 4 times as prognostic factor of successful pessary use: Stress urinary incontinence was found associated with discontinuation of pessary in 5 out of 7 studies. In 7 out of 10 studies previous prolapse surgery or hysterectomy was associated with less continuation of pessary use. Higher age was related to continuation of pessary use in 3 out of 6 studies, whereas no correlation was found in the other studies. In 1 out of 4 studies sexual activity was related to longer pessary use, whereas in 1 out of 4 related to the choice for surgery. In the two other studies no correlation was found. In one study where the prolapse in a specific vaginal compartment was related to outcome, nor cystocele was related to longer pessary use.

Parity en menopausal status and hormonal replacement were mostly not related to continued pessary use.

For peer review only - http://bmjopen.bmj.com/site/about/guidelines.xhtml

3. Review of factors influencing failure of POP surgery.

A systematic review of factors influencing failure of POP surgery was performed concerning recurrence after surgery (surgery failure). There were 1 case control study, 3 prospective studies and 6 retrospective studies. There were 2298 women included in the studies. Forty-four (44) potential risk factors have been studied, of which 12 risk factors have at least once been identified as statistically significant risk factors in a multivariate logistic regression analysis (appendix 4).

6.4 Summary of known and potential risks and benefits

The present study carries low risks for the participant. Pessary [CE 0086] or surgery is standard care for symptomatic pelvic organ prolapse. Known risks for surgery are blood loss, risk of infection, dyspareunia, urine incontinence or a recurrence of a symptomatic pelvic organ prolapse.

The benefit of the study lies in a better understanding of satisfaction and cost effectiveness.

6.5 Description and justification of route of administration and dosage

Not applicable

6.6 Dosages, dosage modifications and method of administration

Not applicable

6.7 Preparation and labelling of Investigational Medicinal Product

Not applicable

6.8 Drug accountability

Not applicable

For peer review only - http://bmjopen.bmj.com/site/about/guidelines.xhtml

7. NON-INVESTIGATIONAL PRODUCT

Not applicable.

7.1 Name and description of non-investigational product(s)

Not applicable.

7.2 Summary of findings from non-clinical studies

Not applicable.

7.3 Summary of findings from clinical studies

Not applicable.

7.4 Summary of known and potential risks and benefits

Not applicable.

7.5 Description and justification of route of administration and dosage

Not applicable.

7.6 Dosages, dosage modifications and method of administration

Not applicable.

7.7 Preparation and labelling of Non Investigational Medicinal Product

Not applicable.

7.8 Drug accountability

Not applicable.

For peer review only

For peer review only - http://bmjopen.bmj.com/site/about/guidelines.xhtml

8. METHODS

8.1 Study parameters/endpoints

8.1.1 Main study parameter/endpoint

The primary outcome of this study is the percentage of women with much or very much improvement of POP symptoms at 2 years follow-up, as measured with the Patient Global Impression of Improvement (PGI-I) scale [22].
PGI-I is a 7-point Likert scale, with scores ranging from very much worse to very much improved. Success is defined as 'much or very much' improvement.

8.1.2 Secondary study parameters/endpoints (if applicable)

1. Changes in symptom bother and quality of life at 12 and 24 months follow up.
2. Changes in sexual function at 12 and 24 months follow up.
3. Changes in general quality of life at 3, 6, 12 and 24 months.
4. Adverse events/complications related to both treatment strategies.
5. Development of prediction model to identify fail factors for pessary and surgery
6. Cost-effectiveness

8.1.3 Other study parameters (if applicable)

Baseline characteristics: Age; ethnicity; allergies; smoking; obstetric history including number and mode of deliveries; menopausal status; hormone use; use of medication; height; weight; co-morbidity (diabetes mellitus, COPD); history of gynaecological operations; family history of prolapse; duration of complaints;.
Physical examination: time, POP-Q, atrophy, vulvar deviations, stress test.
Brand pessary, type of surgery.

8.2 Randomisation, blinding and treatment allocation

After written informed consent is obtained, and inclusion and exclusion criteria are assessed, women will be randomly allocated in a 1:1 ratio to either treatment with a pessary or surgical treatment. Randomization will be done web based using ALEA, the software for randomization in clinical trials currently used by most studies in the Dutch consortium for studies on women's health and reproduction studies. The randomization sequence will be computer generated using variable blocks of two and four, stratified for centre.
After entering the woman's initials and confirming inclusion criteria on the website, a

For peer review only - http://bmjopen.bmj.com/site/about/guidelines.xhtml

unique number for randomization will be generated and the allocation code will be disclosed. This unique number cannot be deleted afterwards. This study will be open label because the nature of the intervention meant that masking to the intervention was not possible. Women who attend the cohort fitting failure will also be registered in ALEA.

In case the woman is willing to participate but actively opts for one of both treatments, she will also be registered in ALEA.

All groups will have the same data collection and follow up as displayed in appendix 5. We expect differences in the study parameters between RCT and cohort, in effectivity, satisfaction and cost effectivity.

8.3 Study procedures (see also appendix 5)

This study will be performed within the Dutch Urogynaecology Research Consortium, a subdivision of the Dutch Consortium for studies on women's health. Infrastructure (research nurses for counseling and data-monitoring, the use of web-based data entry), expertise on methodology and cost-effectiveness is shared.

1. Symptom bother and disease-specific quality of life are measured with the Pelvic Floor Distress Inventory (PFDI-20) and Pelvic Floor Impact Questionnaire (PFIQ) [23,24]. The validated version of the Dutch PFDI consists of five domains: pelvic organ prolapse, urinary incontinence, overactive bladder, pain, and obstructive micturition. The PFIQ measures the impact of urogenital symptoms on quality of life and consist of five domains: physical functioning, mobility, emotional health, embarrassment and social functioning.

2. Sexual function is measured with the PISQ-R. It is an international disease-specific questionnaire that measures sexual functioning in sexually active and inactive participants [26].

3. Generic quality of life is measured with the EQ-5D and a questionnaire "doktersbezoek".

4. The adverse events of surgery recorded will consist of; direct peri-and postoperative complications (bleeding, pain and infection); interventions for complications; recurrent prolapse; de novo stress urinary incontinence. The adverse events of pessary recorded will consist of; discharge; pain; discomfort; bleeding; involuntary loss of pessary; de novo stress urinary incontinence.

5. The development of a prediction model is separately described in paragraph "data analyses".

6. The economic evaluation is described below.

For peer review only - http://bmjopen.bmj.com/site/about/guidelines.xhtml

ANTICIPATED COST-EFFECTIVENESS/BUDGET IMPACT

Cost differences between the two strategies are mainly the result from differences in costs associated with the initial intervention. Cost of a POP procedure is estimated at 4000 euros direct medical costs, and 4000 euros associated with lost productivity (indirect costs) if a societal perspective is used. The direct cost of pessary use is estimated at 200 euros, including costs for the pessary itself (50 euros) and consultations in the first year (150 euros). The estimated cost differences between the two strategies depend on the extent that women are (and remain) satisfied with the initial procedure (surgical or pessary): in case of dissatisfaction with the procedure, additional costs are generated by a subsequent intervention ((re-)operation, pessary, or pelvic physiotherapy). The flowchart (see appendix 1) illustrates the expected outcomes for each strategy. Based on the assumptions reflected in this flowchart, combined with approximate estimates for unit costs for POP surgery, pessary, GP and specialist visits, the anticipated impact on the annualhealth care budget as well as societal costs were estimated.

At present, the primary therapy for women presenting with moderate to severe POP is either surgery or pessary. The exact ratio is unknown, but is probably 50/50. If 50% women would receive primary surgery the current medical costs amount to 34 million Euros. If all women would start with pessary therapy, these costs would be 20 million euros, and the potential budget impact would be 14 million Euros/year. Asit is not realistic that all women will start with pessary if this strategy proves to be successful, at 85% implementation of the pessary strategy, the annual budgetimpact will be around 10 million euros. The economic impact to society (including indirect (productivity) costs) will be 28 million euros and 20 million euros, at 100% and 85% implementation, respectively.

Sensitivity analyses showed, that these “base case” results are affected by estimated unit costs for POP surgery (direct and indirect costs) and the satisfaction rate for pessary, relative to surgery, but even the most conservative assumptions would lead to major cost savings for the health care budget (5 million euros) and society (15 million euros).

ECONOMIC EVALUATION

The economic evaluation will be based on the randomized trial. Considering the non-inferiority design of the study, we will not be able to rule out a small but acceptable

For peer review only - http://bmjopen.bmj.com/site/about/guidelines.xhtml

difference in favor of POP surgery. Consequently, the economic evaluation will be setup as a cost-effectiveness analysis, where cost-effectiveness will be expressed as costs per improvement outcome (much or very much improvement on the Patient Global Impression of Improvement (PGI-I)), and the incremental cost-effectiveness ratio as costs saved per additional case of unsatisfactory outcome. We will also perform a cost-effectiveness analysis using QALYs as health outcome, to express the difference between the two strategies in terms of costs (saved) per QALY (lost).

The economic evaluation will therefore encompass a cost-effectiveness analysis (CEA), a cost-utility analysis (CUA) as well as a budget impact analysis (BIA) from a health care budget and a societal perspective, with a time horizon between randomization and 2 years follow up. The primary outcome in the cost-effectiveness analysis will be costs per satisfactory outcome (primary clinical outcome), and the incremental cost-effectiveness ratio will reflect the costs saved per additional case of unsatisfactory outcome. As we hypothesize that pessary as a primary strategy in these patients does not result in more unsatisfactory outcomes, increased use of pessary will result in a decrease in the number of POP surgeries, and associated costs of hospital stay, recovery and (from a societal perspective) productivity loss (non-inferior strategy at lower costs).

Based on data actually observed in the trial, total costs associated with both surgery and pessary as a primary strategy will be estimated. Total costs can be divided into direct medical costs, non-medical costs and indirect costs. Direct medical costs are generated by utilization of primary or secondary health care services (including POP surgery, hospital stay, diagnostic procedures, medication). Non-medical costs are generated by travel expenses, and informal care; and indirect costs result from lost productivity due to absence from work or lost opportunity for non-paid activities. Non-medical and indirect costs are only included in the analysis from a societal perspective.

Resource utilization will be documented in the clinical report form (CRF) and complementary patient questionnaires, based on the Medical Consumption Questionnaire (MCQ) and Productivity Costs Questionnaire (PCQ) [29,30]. In patients for whom complete follow-up is not available, cost and quality-of-life datawill be extrapolated using multiple imputations. Unit costs will be based on Dutch guideline prices (for primary and secondary health services, informal care and lost productivity),

For peer review only - http://bmjopen.bmj.com/site/about/guidelines.xhtml

and market prices (for medication)[31,32]

Similarly, the incremental costs per QALY gained will be estimated over a period of two years. Health state utilities to estimate QALYs will be derived from an EQ-5D measurement at discharge, as well as at follow-up assessments. Utility values for EQ-5D scores will be based on UK-estimates (Dolan, 1997). Utility scores will be linearly interpolated, assuming constant increase/decrease between subsequent assessments.

Robustness for sampling uncertainty as well as uncertainty associated with cost estimates and assumptions will be assessed in sensitivity analyses, including: Dutch health states (Lamers, 2005) instead of the UK based model in the main analyses; and varying unit costs for pertinent volumes of health care utilization (e.g. costs of POP surgery, pessary use, productivity costs).
The incremental costs and effects will be depicted in a cost effectiveness plane and cost-effectiveness acceptability curves providing information directly interpretable as the probability of one intervention being cost-effective compared to the alternative given a ceiling ratio that policy makers are willing to invest.

BIA

In a budget impact analysis, study results will be extrapolated to the national level to estimate the total impact on the health care budget per annum for the Netherlands in terms of cost reduction and health outcomes (satisfactory outcomes as well as QALYs). As economic consequences of the intervention are expected to spanmultiple years, this accumulation of cost (savings) will be reflected in the budget impact analyses.

The Budget Impact Analysis will be executed according to the international ISPOR guidelines [33]. This framework for creating a budget impact model includes formalized guidance about the acquisition and use of data in order to make budget projections. In addition to the societal perspective, the BIA will therefore be also report economic consequences from the perspective of the Dutch budgetary health care framework (BKZ). If the probability of an unsatisfactory outcome exceeds the non-inferiority limit, recommending pessary as primary treatment for all women isnot feasible, and an economic evaluation/budget impact analysis is not sensible. To estimate costs, we will follow the Handleiding Kosten onderzoek CVZ 2010.

For peer review only - http://bmjopen.bmj.com/site/about/guidelines.xhtml

8.4 Withdrawal of individual subjects

Subjects can leave the study at any time for any reason if they wish to do so without any consequences. The investigator can decide to withdraw a subject from the study for urgent medical reasons.

8.4.1 Specific criteria for withdrawal (if applicable)

Not applicable.

8.5 Replacement of individual subjects after withdrawal

We will not replace patients who withdrew informed consent. We will replace patients that are randomized by mistake, for example because of technical errors with online randomization.

8.6 Follow-up of subjects withdrawn from treatment

Patients withdrawn from the intervention but not from informed consent will be followed up.

8.7 Premature termination of the study

This study includes standard care, therefore it is very unlikely that unexpected complications will occur. Therefore premature termination is not applicable.

For peer review only

For peer review only - http://bmjopen.bmj.com/site/about/guidelines.xhtml

9. SAFETY REPORTING

9.1

Temporary halt for reasons of subject safety (section 9.1, CCMO Template Research Protocol)

In accordance to section 10, subsection 4, of the WMO, the sponsor will suspend the study if there is sufficient ground that continuation of the study will jeopardise subject health or safety. The sponsor will notify the accredited METC with undue delay of a temporary halt including the reason for such an action. The study will be suspended pending further review by the accredited METC. The investigator will take care that all subjects are kept informed.

Temporary halt and (prematurely) end of study report (section 12.5, CCMO Template Research Protocol)

The sponsor will notify the METC immediately of a temporary halt of the study, including the reason of such an action.

9.2 AEs, SAEs and SUSARs

9.2.1 Adverse events (AEs)

Adverse events are defined as any undesirable experience occurring to a subject during the study, whether or not considered related to the study. All adverse events reported spontaneously by the subject or observed by the investigator or his staff will be recorded. During visits complaints will be questioned systematically.

9.2.2 Serious adverse events (SAEs)

A serious adverse event is any untoward medical occurrence or effect that at any dose:

- results in death;
- is life threatening (at the time of the event);
- requires hospitalisation or prolongation of existing inpatients' hospitalisation (>4 days);
- results in persistent or significant disability or incapacity;
- is a congenital anomaly or birth defect;
- Any other important medical event that may not result in death, be life threatening, or require hospitalization, may be considered a serious adverse experience when,

For peer review only - http://bmjopen.bmj.com/site/about/guidelines.xhtml

based upon appropriate medical judgement, the event may jeopardize the subject or may require an intervention to prevent one of the outcomes listed above.

SAEs have to be reported when its occurrence appears in two days after the study operations. The investigators in participating centres should inform the coordinating investigator as soon as possible but at least the next working day.

The sponsor will report the SAEs through the web portal *ToetsingOnline* to the accredited METC that approved the protocol, within 15 days after the sponsor has first knowledge of the serious adverse events.

SAEs that result in death or are life threatening should be reported expedited. The expedited reporting will occur not later than 7 days after the responsible investigator has first knowledge of the adverse event. This is for a preliminary report with another 8 days for completion of the report.

9.2.3 Suspected unexpected serious adverse reactions (SUSARs)

Not applicable.

9.3 Annual safety report

The annual safety report will be combined with the annual progress report (see chapter 12.4).

9.4 Follow-up of adverse events

All AEs will be followed until they have abated, or until a stable situation has been reached. Depending on the event, follow up may require additional tests or medical procedures as indicated, and/or referral to the general physician or a medical specialist.
SAEs need to be reported till end of study within the Netherlands, as defined in the protocol

9.5 Data Safety Monitoring Board (DSMB)

Since both techniques are standard practice, and no major unexpected complications are foreseen, no interim analysis is planned. A Data Safety and Monitoring Board will not be installed, as both procedures are regularly used and acceptable options in current clinical practice.

For peer review only - http://bmjopen.bmj.com/site/about/guidelines.xhtml

10. STATISTICAL ANALYSIS

The results of the study will be reported according to the CONSORT statement.

The main outcomes will be analysed and presented according the intention-to-treat principle. Since in this pragmatic trial we expect that in the pessary strategy group 40% of women will cross over to surgery an additional per protocol analyses is foreseen. This will provide more insight in the effect of surgery after pessary therapy as compared to surgery or pessary therapy alone.

We plan a subgroup analysis for the location of the prolapse: anterior prolapse versus posterior prolapse.

The cohort with patients treated according their preference will be analysed separately from the randomized trial, and presented in the same manuscript, which will provide insight into the generalizability of the results.

10.1 Primary study parameter(s)

The primary outcome, success (much or very much improvement) or no success (a little better, no change, a little worse, much worse or very much worse) on the PGI-I will be expressed in percentage point differences. Differences between the percentages will be tested using a chi-square test. A p-value <0.05 will be considered statistically significant.

For the cohort study results will be presented separately, and the same analyses will be done. Differences between the trial arm and the cohort arm will be tested using the chi-square test, to determine the generalizability of the results.

10.2 Secondary study parameter(s)

The PFDI, PFIQ and PISQ-r are all interval scales. Differences between baseline and 12 and 24 months follow up will be assessed using an independent t-test when normality can be assumed, or by non-parametric tests when the data are not normally distributed. Effect sizes will be calculated to estimate the magnitude of changes.

Differences in EQ5-D scores and “ziekteverzuim” between baseline and at 3, 6, 12 and 24 months will be assessed using t-test and further incorporated in the cost-effectiveness analyses.

Imputation statistics will be used or missing data.

(Serious) adverse events will be categorized and chi-square statistics, with calculation of relative risks when appropriate, will be applied in analyses.

For peer review only - http://bmjopen.bmj.com/site/about/guidelines.xhtml

Prediction model

A prediction model that uses predefined variables, as potential predictors of failure of pessary therapy, will be developed using multivariable regression analysis.Missing data will be imputed.

Predictors for failure derived from literature are a large genital hiatus (gh > 4 cm), women being sexually active, age > 65 years, prolapse POP-Q stage 3, previous hysterectomy. If applicable, a prediction rule for the chance of failure of pessary therapy will be constructed, which could be presented as a normogram which could be used to determine the chance of failure on pessary therapy.

Internal validity will be assessed using bootstrapping techniques; shrinkage will be applied to the parameter estimates. Model performance will be assessed with discriminative capacity and calibration. Calibration will be assessed by comparing the mean predicted probability that patients failed on pessary therapy with the mean observed probability that patients failed on pessary therapy. To do so, the total cohort will be split into ten groups based on the deciles of the predicted probability. Per group the mean predicted probability will be calculated as well as the mean observed predicted probability. Discriminative capacity of the model will be assessed with receiver operation characteristics (ROC) analysis and the area under the ROC curve (AUC).

We will also look at factors that could explain failure of surgery. Our systematic review on POP and recurrent POP after surgery showed that 6 preoperative items, eg. POP stage, age, family history, preoperative incontinence, previous POP or incontinence surgery, previous hysterectomy seems to be predictive for recurrence.

Women with previous POP or incontinence surgery are excluded from our study, leaving 4 predefined potential predictive factors. After the 2 year follow-up has been performed, we will reconsider which factors to include in a prognostic model, based on the current literature. We will select predictors from literature with the highest predictive value, where about 1 predictor could be selected for each 10 surgery failures.

Using interaction terms the effect of a differential effect in women with a higher age (>median) or a lower age (<=median), a higher (>25) or lower BMI(<=25) will be assessed for both pessary as well as surgery failures prediction.

For peer review only - http://bmjopen.bmj.com/site/about/guidelines.xhtml

10.3 Other study parameters

Not applicable.

10.4 Interim analysis (if applicable)

Not applicable, because of the non-inferiority design with low risk and the possibility of cross over.

For peer review only

For peer review only - http://bmjopen.bmj.com/site/about/guidelines.xhtml

11. ETHICAL CONSIDERATIONS

11.1 Regulation statement

This study will be conducted according to the principles of the Declaration of Helsinki (version 10, October 2013) and in accordance with the Medical Research InvolvingHuman Subjects Act (WMO) and other guidelines, regulations and Acts.

11.2 Recruitment and consent

Women with symptomatic pelvic organ prolapse who attend the outpatient clinic will be informed about the study by the gynaecologist or nurse. After checking the in- and exclusion criteria the women will receive verbal and written information about the study. If the woman is willing to participate she is asked to sign the informed consent. All women will undergo the pessary fitting test which is part of the standard evaluation. All women will be contacted at a minimum interval of 1 week. Those women who failed the initial fitting will be offered surgery and attend the cohort fitting failure, the women with a succesfull initial fitting will be asked to enroll in the RCT. In case the woman is willing to participate but actively opts for one of both treatments, she can attend the cohort. Her motivation is requested. In case the women is not willing to participate, she will be registred as “refuser”.

11.3 Objection by minors or incapacitated subjects (if applicable)

Not applicable.

11.4 Benefits and risks assessment, group relatedness

The present study carries no risks for the participant. Pessary or surgery are standard care for symptomatic pelvic organ prolapse. The benefit of the study lies in a better understanding of satisfaction and cost effectiveness.

11.5 Compensation for injury

The sponsor/investigator has a liability insurance which is in accordance with article 7, subsection 9 of the WMO.

The sponsor (also) has an insurance which is in accordance with the legal requirements in the Netherlands (Article 7 WMO and the Measure regarding Compulsory Insurance for Clinical Research in Humans of 23th June 2003). This insurance provides cover for

For peer review only - http://bmjopen.bmj.com/site/about/guidelines.xhtml

damage to research subjects through injury or death caused by the study.

1. € 450.000,-- (i.e. four hundred and fifty thousand Euro) for death or injury for each subject who participates in the Research;
2. € 3.500.000,-- (i.e. three million five hundred thousand Euro) for death or injury for all subjects who participate in the Research;
3. € 5.000.000,-- (i.e. five million Euro) for the total damage incurred by the organisation for all damage disclosed by scientific research for the Sponsor as ‘verrichter’ in the meaning of said Act in each year of insurance coverage.

The insurance applies to the damage that becomes apparent during the study or within 4 years after the end of the study.

11.6 Incentives (if applicable)

Not applicable.

For peer review only

12. ADMINISTRATIVE ASPECTS, MONITORING AND PUBLICATION

12.1 Handling and storage of data and documents

All data will be stored and will be coded. Only the researchers will be able to link patient ID and research code. The handling of personal data complies with the Dutch Personal Data Protection Act. After cessation of the study, patient material will be stored for a maximum of 15 years.

The case report forms and questionnaires will be filled in online. The head investigator will be able to check all the completed forms and questionnaires.

12.2 Monitoring and Quality Assurance

The monitoring will be coordinated by the Dutch Consortium and will be executed by a qualified intern monitor. This person is not involved in design and output of this research. The frequency of checking will be every year. The monitoring plan is discussed in section K of the METC dossier.

12.3 Amendments

All substantial amendments will be notified to the METC.

Non-substantial amendments will not be notified to the accredited METC, but will be recorded and filed by the sponsor.

12.4 Annual progress report

The sponsor/investigator will submit a summary of the progress of the trial to the accredited METC once a year. Information will be provided on the date of inclusion of the first subject, numbers of subjects included and numbers of subjects that have completed the trial, serious adverse events/ serious adverse reactions, other problems, and amendments.

12.5 End of study report

The investigator will notify the accredited METC of the end of the study within a period of 8 weeks. The end of the study is defined as the last patient's last visit.

In case the study is ended prematurely, the investigator will notify the accredited METC within 15 days, including the reasons for the premature termination.

For peer review only - http://bmjopen.bmj.com/site/about/guidelines.xhtml

Within one year after the end of the study, the investigator/sponsor will submit a final study report with the results of the study, including any publications/abstracts of the study,to the accredited METC.

12.6 Public disclosure and publication policy

The research findings will be published in peer reviewed journals.

For peer review only

For peer review only - http://bmjopen.bmj.com/site/about/guidelines.xhtml

13. STRUCTURED RISK ANALYSIS

Not applicable because this study is a low risk study concerning standard care.

13.1 Potential issues of concern

Not applicable.

13.2 Synthesis

Not applicable.

For peer review only

For peer review only - http://bmjopen.bmj.com/site/about/guidelines.xhtml

14. REFERENCES

1: Slieker-ten Hove MC et al. The prevalence of pelvic organ prolapse symptoms and signs and their relation with bladder and bowel disorders in a general female population. Int Urogynecol J. 2009;20(9):1037-45
2. Tegerstedt G, Maehle-Schmidt M, Nyrén O, Hammarström M. Prevalence of symptomatic pelvic organ prolapse in a Swedish population. Int Urogynecol J Pelvic Floor Dysfunct. 2005 Nov-Dec;16(6):497-503
3: Lamers BH et al. Pessary treatment for pelvic organ prolapse and health-related quality of life: a review. Int Urogynecol J. 2011;22(6):637-44
4: Broekman BM et al. Prospective pilot study in 65 women on pessary treatment as primary treatment, unpublished data
5:Broens-Oostveen WC, Mom RM, Lagro-Janssen ALM. De genitale prolaps: behnadleingen beloop in vier huisartspraktijken. Ned Tijdschr Geneeskd 2004, 148 (29); 1444-1448
6: Withagen M et al. Sacrospinous hysteropexy or modified manchester for the treatment of pelvic organ prolapse? Int J Gynaecol Obstet. 2013; Abstract Supplement
7: Vollebregt A et al. Cost-utility analysis of trocar-guided transobturator mesh versus anterior colporrhaphy for primary cystocele repair: a randomised trial. [in preparation]
8: Barber MD[1], Walters MD, Bump RC. Short forms of two condition-specific quality-of-life questionnaires for women with pelvic floor disorders (PFDI-20 and PFIQ-7). Am J Obstet Gynecol. 2005 Jul;193(1):103-13.
9: Pollard ME, Eilber KS, Anger JT. Abdominal approaches to pelvic prolapse repairs. Curr Opin Urol. 2013;23(4):306-11.
10: Dietz V et al. Vaginal surgery for uterine descent; which options do we have? A review of the literature. Int J Gynaecol Obstet. 2009;20(3):349-56
11: C. Maher, Feiner B, Baessler K, Schmid C. Surgical management of pelvic organprolapse in women. Cochrane 2013
12: de Boer TA, Milani AL, Kluivers KB, Withagen MI, Vierhout ME. The effectiveness of surgical correction of uterine prolapse: cervical amputation with uterosacral ligament plication (modified Manchester) versus vaginal hysterectomy with high uterosacral ligament plication. Int Urogynecol J Pelvic Floor Dysfunct. 2009 Nov;20(11):1313-9
13: Bugge C et al. Pessaries (mechanical devices) for pelvic organ prolapse in women. Cochrane database syst rev, 2013;28;2:CD004010
14: van Geelen JM, Dwyer PL. Where to for pelvic organ prolapse treatment after the FDA pronouncements? A systematic review of the recent literature. Int Urogynecol J. 2013 May;24(5):707-18
15: Abdool Z et al. Prospective evaluation of outcome of vaginal pessaries versus surgery in women with symptomatic pelvic organ prolapse. Int Urogynecol J. 2011;22(3):273-8
16: Hullfish KL, Trowbridge ER, Stukenborg GJ. Treatment strategies for pelvic organ prolapse: a cost-effectiveness analysis. Int Urogynecol J. 2011 May;22(5):507-15
17: Cundiff GW, Amundsen CL, Bent AE, Coates KW, Schaffer JI, Strohbehn K, Handa VL. The PESSRI study: symptom relief outcomes of a randomized crossover trial of the ring and Gellhorn pessaries. Am J Obstet Gynecol. 2007 Apr;196(4):405.e1-8
18: Weber MA, Limpens J, Roovers JPWR. Assessment of vaginal atrophy: a review [submitted BJOG]
19: Cobra Ok-Klapper 2012. DCHG 2012
20: Detollenaere RJ, den Boon J, Kluivers KB, Vierhout ME, van Eijndhoven HW.Surgical management of pelvic organ prolapse and uterine descent in the Netherlands. Int Urogynecol J. 2013 May;24(5):781-8
21.Detollenaere RJ, den Boon J, Vierhout ME, van Eijndhoven HW. Uterus preservingsurgery versus vaginal hysterectomy in treatment of uterine descent: a systematic review]. Ned

For peer review only - http://bmjopen.bmj.com/site/about/guidelines.xhtml

Tijdschr Geneeskd. 2011;155(42):A3623
22: Yalcin I, Bump RC. Validation of two global impression questionnaires for incontinence. Am J Obstet Gynecol. 2003 Jul;189(1):98-101
23: Barber MD, Kuchibhatla MN, Pieper CF, Bump RC. Psychometric evaluation of 2 comprehensive condition-specific quality of life instruments for women with pelvic floor disorders. Am J Obstet Gynecol 2001;185(6):1388–1395.
24: Utomo E, Blok BF, Steensma AB, Korfage IJ. Validation of the Pelvic Floor Distress Inventory (PFDI-20) and Pelvic Floor Impact Questionnaire (PFIQ-7) in a Dutch population. Int Urogynecol J. 2014;25(4):531-44.
25: Euroquol—a new facility for the measurement of health-related quality of life. The EuroQol Group. Health Policy. 1990;16:199-208
26: Rogers RG, Rockwood TH, Constantine ML, Thakar R, Kammerer-Doak DN, Pauls RN, Parekh M, Ridgeway B, Jha S, Pitkin J, Reid F, Sutherland SE, Lukacz ES, Domoney C, Sand P, Davila GW, Espuna Pons ME. A new measure of sexual function in women with pelvic floor disorders (PFD): the Pelvic Organ Prolapse/Incontinence Sexual Questionnaire, IUGA-Revised (PISQ-IR). Int Urogynecol J. 2013 Jul;24(7):1091-103
27: Cabana MD, Rand CS, Powe NR, Wu AW, Wilson MH, Abboud PA, Rubin HR. Why don't physicians follow clinical practice guidelines? A framework for improvement. JAMA. 1999 Oct 20;282(15):1458-65
28: Thys SD, Roovers JP, Geomini PM, Bongers MY. Do patients prefer a pessary or surgery as primary treatment for pelvic organ prolapse. Gynecol Obstet Invest. 2012;74(1):6-12
29: www.imta.nl (C. Bouwmans et al. Handleiding iMTA Medical Cost Questionnaire (iMCQ). Rotterdam: iMTA, Erasmus Universiteit Rotterdam, 2013)
30: www.imta.nl (C. Bouwmans et al. Handleiding iMTA Productivity Cost Questionnaire (iPCQ). Rotterdam: iMTA, Erasmus Universiteit, 2013)
31:Hakkaart- van Roijen, S.S. Tan, C.A.M. Bouwmans: Handleiding voor Kostenonderzoek. Methoden en standaard kostprijzen voor economische evaluaties in de gezondheidszorg. CVZ 2010
32: Oostenbrink JB, Koopmanschap MA, Rutten FF. Standardisation of costs: the Dutch Manual for Costing in economic evaluations. Pharmacoeconomics 2002;20(7):443-454
33: Mauskopf JA, Sullivan SD, Annemans L et al. Principles of good practice for budget impact analysis: report of the ISPOR Task Force on good research practices--budget impact analysis. Value Health 2007;10(5):336-347 -> In BIA, klopt niet, nog vervangen door:

References (Cost) effectiveness of pessary use as compared to surgery systematic review

1: Mamik MM, Rogers RG, Qualls CR, Komesu YM. Goal attainment after treatment in patients with symptomatic pelvic organ prolapse. Am J Obstet Gynecol. 2013 Nov;209(5):488.e1-5.
2: Abdool Z, Thakar R, Sultan AH, Oliver RS. Prospective evaluation of outcome of vaginal pessaries versus surgery in women with symptomatic pelvic organ prolapse. Int Urogynecol J. 2011 Mar;22(3):273-8.
3: Lowenstein L, Gamble T, Sanses TV, van Raalte H, Carberry C, Jakus S, Pham T, Nguyen A, Hoskey K, Kenton K; Fellow's Pelvic Research Network. Changes in sexual function after treatment for prolapse are related to the improvement in body image perception. J Sex Med. 2010 Feb;7(2 Pt 2):1023-8.
4: Barber MD, Walters MD, Cundiff GW; PESSRI Trial Group. Responsiveness of the Pelvic Floor Distress Inventory (PFDI) and Pelvic Floor Impact Questionnaire (PFIQ) in women undergoing vaginal surgery and pessary treatment for pelvic organ prolapse. Am J Obstet Gynecol. 2006 May;194(5):1492-8.

References Systematic review of factors influencing pessary fitting and continuation

1.Abdool Z, Thakar R, Sultan AH, Oliver RS (2010) Prospective evaluation of outcome of

For peer review only - http://bmjopen.bmj.com/site/about/guidelines.xhtml

vaginal pessaries versus surgery in women with symptomatic pelvic organ prolapse. Int Urogynecol J Pelvic Floor Dysfunct 16

2. Alperin M, Khan A, Dubina E, Tarnay C, Wu N, Pashos CL, Anger JT.Female Pelvic Med Reconstr Surg. 2013 May-Jun;19(3):142-7.Patterns of pessary care and outcomes for medicare beneficiaries with pelvic organ prolapse.
3. Bai SW, Yoon BS, Kwon JY, Shin JS, Kim SK, Park KH (2005) Survey of the characteristics and satisfaction degree of the patients using a pessary. Int Urogynecol J Pelvic Floor Dysfunct 16 (3):182–186
4. Barber MD, Walters MD, Cundiff GW (2006) Responsiveness of the Pelvic Floor Distress Inventory (PFDI) and Pelvic Floor Impact Questionnaire (PFIQ) in women undergoing vaginal surgery and pessary treatment for pelvic organ prolapse. Am JObstet Gynecol 194(5):1492–149
5. Brincat C, Kenton K, Pat FM, Brubaker L (2004) Sexual activity predicts continued pessary use. Am J Obstet Gynecol 191(1):198–200
6. Broens-Oostveen MC, Mom RM, Lagro-Janssen AL (2004)Genital prolapse; treatment and course in four general practices.Ned Tijdschr Geneeskd 148(29):1444–1448, 17
7. Chan SS, Cheung RY, Yiu KW, Lee LL, Pang AW, Chung TK. Int Urogynecol J. 2012 Aug;23(8):1027-33. Symptoms, quality of life, and factors affecting women's treatment decisions regarding pelvic organ prolapse.
8. Clemons JL, Aguilar VC, Sokol ER, Jackson ND, Myers DL (2004) Patient characteristics that are associated with continued pessary use versus surgery after 1 year. Am J Obstet Gynecol 191(1):159–164
9. Cundiff GW, Amundsen CL, Bent AE, Coates KW, Schaffer JI, Strohbehn K et al (2007) The PESSRI study: symptom relief outcomes of a randomized crossover trial of the ring and Gellhorn pessaries. Am J Obstet Gynecol 196(4):405–408
10. Fernando RJ, Thakar R, Sultan AH, Shah SM, Jones PW (2006) Effect of vaginal pessaries on symptoms associated with pelvic organ prolapse. Obstet Gynecol 108(1):93–99
11. Friedman S, Sandhu KS, Wang C, Mikhail MS, Banks E (2010) Factors influencing long-term pessary use. Int Urogynecol J Pelvic Floor Dysfunct 21(6):673–678
12. Hanson LA, Schulz JA, Flood CG, Cooley B, Tam F (2006) Vaginal pessaries in managing women with pelvic organ prolapse and urinary incontinence: patient characteristics and factors contributing to success. Int Urogynecol J Pelvic Floor Dysfunct 17(2):155–159
13. Jones K,Yang L, Lowder JL, Meyn L, Ellison R, ZyczynskiHMet al (2008) Effect of pessary use on genital hiatus measurements in women with pelvic organ prolapse. Obstet Gynecol 112(3):630–636
14. Geoffrion R, Zhang T, Lee T, Cundiff GW.Female Pelvic Med Reconstr Surg. 2013 Nov-Dec;19(6):339-45. Clinical characteristics associated with unsuccessful pessary fitting outcomes.
15. Komesu YM, Rogers RG, Rode MA, Craig EC, Gallegos KA, Montoya AR et al (2007) Pelvic floor symptom changes incpessary users. Am J Obstet Gynecol 197(6):620–626
16. Kuhn A, Bapst D, Stadlmayr W, Vits K, Mueller MD (2009) Sexual and organ function in patients with symptomatic prolapse: are pessaries helpful? Fertil Steril 91(5):1914–1918
17. Lone F1, Thakar R, Sultan AH, Karamalis G. A 5-year prospective study of vaginal pessary use for pelvic organ prolapse. Int J Gynaecol Obstet. 2011 Jul;114(1):56-9.
18. Maito JM, Quam ZA, Craig E, Danner KA, Rogers RG (2006) Predictors of successful pessary fitting and continued use in a nurse-midwifery pessary clinic. J Midwifery Womens Health 51 (2):78–84
19. Mamik MM, Rogers RG, Qualls CR, Komesu YM. Goal attainment after treatment in patients with symptomatic pelvic organ prolapse. Am J Obstet Gynecol. 2013 Nov;209(5):488.e1-5.
20. Manchana T, Bunyavejchevin S. Impact on quality of life after ring pessary use for pelvic organ prolapse. Int Urogynecol J. 2012 Jul;23(7):873-7.
21. Markle D, Skoczylas L, Goldsmith C, Noblett K. Patient characteristics associated with a

For peer review only - http://bmjopen.bmj.com/site/about/guidelines.xhtml

successful pessary fitting. Female Pelvic Med Reconstr Surg. 2011 Sep;17(5):249-52.
22. Mokrzycki ML, Hatangadi SB, Zaccardi JE, Cox S. Preexisting stress urinary incontinence: a predictor of discontinuation with pessary management. J Low Genit TractDis. 2001 Oct;5(4):204-7.
23. Mutone MF, Terry C, Hale DS, Benson JT (2005) Factors which influence the short-term success of pessary management of pelvic organ prolapse. Am J Obstet Gynecol 193(1):89–94
24. Nguyen JN, Jones CR. Pessary treatment of pelvic relaxation: factors affecting successful fitting and continued use. J Wound Ostomy Continence Nurs. 2005;32(4):255-61; quiz 262-3.
25. Patel M, Mellen C, O'Sullivan DM, Lasala CA. (2010) Impact ofpessary use on prolapse symptoms, quality of life, and bodyimage. Am J Obstet Gynecol 18
26. Powers K, Lazarou G, Wang A, LaCombe J, Bensinger G.Greston WM et al (2006) Pessary use in advanced pelvic organprolapse. Int Urogynecol J Pelvic Floor Dysfunct17(2):160–164.

Reference list Systematic review of factors influencing failure of POP surgery

1. Whiteside JL, Weber AM, Meyn LA, Walters MD. Am J Obstet Gynecol. 2004 Nov;191(5):1533-8.factors for prolapse recurrence after vaginal repair.
2. Diez-Itza I, Aizpitarte I, Becerro A. Int Urogynecol J Pelvic Floor Dysfunct. 2007 Nov;18(11):1317-24. Risk factors for the recurrence of pelvic organ prolapse after vaginal surgery: a review at 5 years after surgery.
3. Fialkow MF, Newton KM, Weiss NS. Int Urogynecol J Pelvic Floor Dysfunct. 2008 Nov;19(11):1483-7.Incidence of recurrent pelvic organ prolapse 10 years following primary surgical management: a retrospective cohort study.
4. Jeon MJ, Chung SM, Jung HJ, Kim SK, Bai SW. Gynecol Obstet Invest. 2008;66(4):268- 73 Risk factors for the recurrence of pelvic organ prolapse.
5. Dällenbach P, Jungo Nancoz C, Eperon I, Dubuisson JB, Boulvain M. Int Urogynecol J. 2012 Jan;23(1):35-41. Incidence and risk factors for reoperation of surgically treated pelvic organ prolapse.
6. Weemhoff M, Vergeldt TF, Notten K, Serroyen J, Kampschoer PH, Roumen FJ. Int Urogynecol J. 2012 Jan;23(1):65-71. Avulsion of puborectalis muscle and other risk factors for cystocele recurrence: a 2-year follow-up study.
7. Tegerstedt G1, Hammarström M. Acta Obstet Gynecol Scand. 2004 Aug;83(8):758-63. Operation for pelvic organ prolapse: a follow-up study.
8. Medina CA, Candiotti K, Takacs P. Int J Gynaecol Obstet. 2008 May;101(2):184-7. Wide genital hiatus is a risk factor for recurrence following anterior vaginal repair.
9. Salvatore S1, Athanasiou S, Digesu GA, Soligo M, Sotiropoulou M, Serati M, Antsaklis A, Milani R. Neurourol Urodyn. 2009;28(4):301-4. doi: 10.1002/nau.20639. Identification of risk factors for genital prolapse recurrence.
10. Kawasaki A, Corey EG, Laskey RA, Weidner AC, Siddiqui NY, Wu JM. J Reprod Med. 2013 May-Jun;58(5-6):195-9. Obesity as a risk for the recurrence of anterior vaginal wall prolaps after anterior colporrhaphy.

For peer review only - http://bmjopen.bmj.com/site/about/guidelines.xhtml

Appendix 1:

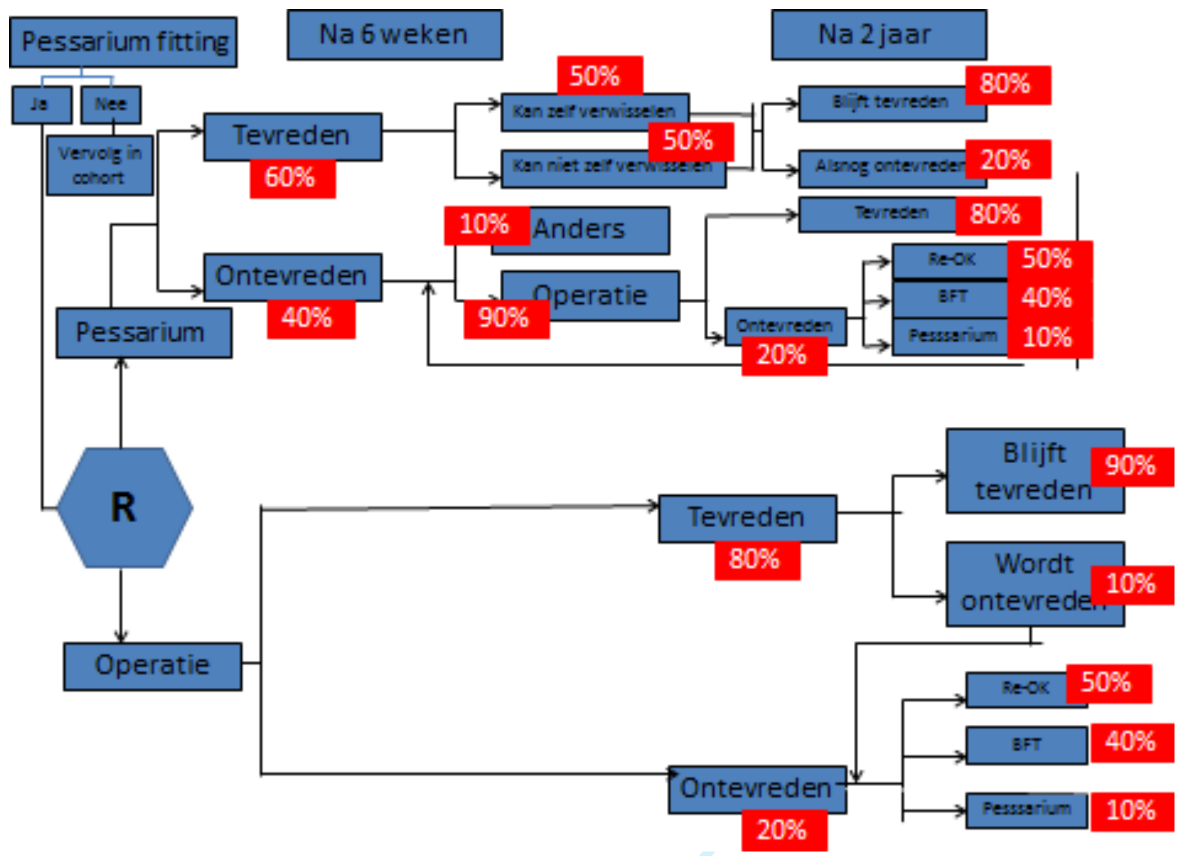

For peer review only - http://bmjopen.bmj.com/site/about/guidelines.xhtml

Appendix 2 Review on (Cost) effectiveness of pessary use as compared to surgery:

Reference	Study type	Characteristics	Intervention (I)	Controls (C)	Outcome measures and follow-uptime	Results
Mamik, 2012<br><br>AJOG 2013:209:488	Design<br>Case-control<br>N = 100<br>Country<br>US	Aim: compare goal achievement and global improvement between pessary and surgery for [illegible] stage ≥2.<br><br>Inclusion criteria: >18 year old, read and write in English<br><br>Exclusion: not given	Vaginal pessary<br>N = 50	Prolapse surgery<br>N = 50	Primary outcome:<br>Goal attainment<br><br>Secondary:<br>PGI-I<br>PFDI-20<br>PISQ-12<br>Body Image scale<br><br>Follow-up: 3 months	Primary outcome:<br>Goal attainment sign. higher score after surgery (8.6 vs 6.4)<br>Secondary outcomes<br>PGI-I sign (p=0.04) better improvement after surgery (2.4vs 1.9 points)<br>PFDI-20 sign (p=0.02) higher change (89 vs 43 points)<br>PISQ-12 and BIS no sign difference<br><br>Additional: 10% crossed over from pessary to surgery within 3 months [illegible]% referred from surgery after they had been selected as eligible.<br>No follow-up in pessary group is 40% (20/50) and surgery 30% [illegible]
Abdool, 2011	Design<br>Cohort study<br><br>N total = [illegible]<br><br>Country:<br>UK	Aim of the study<br>to evaluate and compare the effectiveness of pessariesand surgery in women with symptomatic pelvic organ prolapse.<br>Inclusion criteria<br>- Women referred to a specialist urogynaecology unit with symptomatic [illegible] between June 2002 andMay 2007	Intervention vaginal pessary<br>N = 359	Controls surgery<br>N = 195	Primary outcomes:<br>Sheffield POP questionnaire [illegible]<br><br>Secundary outcomes:<br>None<br>Follow up:<br>For the surgery and pessary groups 14 months(SD 6.14) and 12 months (SD 3.1), respectively.	Primary outcomes:<br>No difference in functional outcome after 1 year follow-up between groups<br>Additional:<br>Only 45% in pessary group en 55% in surgery group responded at 12monthsIn pessary group 24.7% (89/359) crossed to surgery but were not analyzed In pessary group 7.3% stopped because of other reasons.<br>Selection and patient preference bias<br>The mean age was significantly higher in the pessary group compared to the surgerygroup (68.4 +/− 13.08 vs 60.4 +/−12.25 years, respectively).

		**Exclusion criteria**<br>· Subjects fitted with pessaries for urinary incontinence and those who had concomitant urinary incontinence surgery (e.g. TVT)<br>· Subjects who started inthe pessary group but subsequently requested surgery were excluded from analysis in both the surgery and pessary group.				

For peer review only

Study protocol

**Lowenstein 2010**<br><br>**J Sex Med 2010; 7:-1023**<br><br>**28**	**Design**<br>**Cohort study**<br>**N= 235**<br><br>**Country**<br>**US**	**Aim of the study**<br>**First to evaluate patient-reported outcome, POP symptoms, sexual functioning and body image following treatment of P Second to compare surgery with pessary**<br><br>**Inclusion: ≥18 year, ≥ satge 2 POP, complete questionnaire at baseline and at ≥6 months follow up**<br><br>**Exclusion: recurrent UTI, peripheral neuropathy, using pessary at initial presentationor POP surgery < 6 months prior to presentation**	**Intervention**<br>**N = 202 surgery**	**Controls**<br>**N = 33 pessary**	**Primary outcomes**<br>**PFDI-20**<br>**PISQ-12**<br>**Modified Body Image scale**<br>**All at six months follow-up**	**Results**<br>**After multivariate analyses, including type of intervention, BMI and differencein Body image were associated with change in total PISQ (sexual functioning) score**<br><br>**In the pessary group there was no significant improvement in sexual functioningas compared to surgery (-2.5 versus +11.5)**<br>**Additional:**<br>**No figures presented for pessary and surgery group, with exemption oftheSexual functioning (PISQ-12) result above.**

For peer review only - http://bmjopen.bmj.com/site/about/guidelines.xhtml

Study protocol

**Barber, 2006**	**Design**<br>Case-c ontrol study<br>**Ntotal=106Country:**<br>USA	Aim of the studyto evaluate the responsiveness of the PelvicFloor Distress Inventory (PFDI) andelicvPFloor Impact Questionnaire (PFIQ) in women with pelvic organ prolapse undergoingsurgical and nonsurgical management.<br><br>Inclusion criteria<br>Surgery group:<br>Stage III or IV prolapse, wereat least 18 years, and scheduled for vaginal prolapse repair.<br>Pessary group:<br>women with symptomatic pelvic organ prolapse oftsageII or greater. (Pessri trial)<br><br>Exclusion criteriaSurgery group:<br>· mentally or physically incapable of completingthe questionnaires.<br>Pessary group:<br>· were pregnant, were currently using a pessary, orhad vaginal agglutination that precluded pessary insertion.	**Intervention**<br>**Pessary in women with stage II or greater POP**<br>**N = 42**	**Controls**<br>**Surgery in womenwithstage III or greater POP**<br>**N = 64**	**Primary outcomes:**<br>**PFDI and PFIQ**<br>**Secundary outcomes:**<br><br>**Follow up:**<br>**3 months (Pessary group)or 6 months (Surgery group) after initiationoftreatment.**	**Primary outcomes:**<br><br>**After controlling for preoperative prolapse stage and baseline HRQOLscores,subjects in the Surgery group had significantly greater improvement in each of the scales of the PFDI and the prolapse and urinary scales of the PFIQ than did the Pessary group.**<br>**Scores from each of the scales of the PFDI improved by 14 to 15**<br>**points more on average after treatment in the Surgery group than those ofthe Pessary group (P < .01 for each) after adjusting for the above baseline differences.**<br><br>**Similarly, for the prolapse and urinary scales of the PFIQ, scores improved13and 17 points more, respectively, in the Surgery group than the Pessary group after treatment. (P < .05 for each).**<br><br>**Four of 64 (6%) of subjects in the Surgery group had recurrent prolapsedevelopbeyond the hymen by 6 months after surgery. No subjects underwent reoperation for recurrent prolapse during the study period.**<br>**Additional:**<br><br>**Difference in followup**<br>**Selection bias**

For peer review only

Appendix 3 Review on risk factors for failure of pessaries:

study		n	FU period	success initial	continued pes	stopped pes	cross over surgery	predict fit	predict contin
Wu 1997	retrospective	110	3 yrs	81 (74%)	43 (39%)		at least 5	nee	ja
Mokrzycki 2001	retrospective	42	3 mth		24 (57%)	18 (43%)	nr	nee	ja
Clemons 2004	prospective	100	1 yr	73 (73%)	43 (43%)		16/73 (27%)	nee	ja
Brincat 2004	retrospective	169	2,5 yr	144 (85%)	82 (60%)	54 (40%)	32/144 (22%)	nee	ja
Mutone 2004	retrospective	407	3 wks	168 (41%)		96 (57%)	nr	ja	ja
Powers 2006	retrospective	32	1 yr			20 (62%)	12 (38%)	nee	ja
Broens-Oostveen 2004	retrospective	192	9 yr			107 (52%)	nr	nee	ja
Nguyen 2005	retrospective	130	1 yr	111 (82%)	85 (65%)		20 (15%)	ja	ja
Hanson 2005	retrospective	1216	3 mth	1043 (86%)	744 (71%)		nr	nee	ja
Fernando 2006	prospective	203	4 mth	nr	153 (75%)		28 (14%)	nee	ja
Barber 2006	prospective	42	3 mth	nr		0	nr	nee	nee
Maito 2006	retrospective	120	6 mth	106 (88%)	92 (77%)		9 (8%)	ja	ja
Cundiff 2007	prospective	85	1 yr	65/71 (92%)			5 (6%)	nee	nee
Komesu 2007	prospective	64	6-12 mth		36 (56%)	28 (44%)	2 (3%)	nee	ja
Jones 2008	prospective	90	3 mth		42 (47%)		nr	nee	ja
Kuhn 2009	prospective	71	3 mth		32 (45%)		nr	nee	nee
Sarma 2009	retrospective	120	1 mth		45 (38%)		18 (15%)	nee	nee
Friedman 2010	retrospective	150	1 yr		115 (77%)	35 (23%)	25 (17%)	nee	ja
Patel 2010	prospective	65	3 mth	54 (83%)	54 (83%)		5 (8%)	nee	nee
Abdool 2010	prospective	359	1 yr	296 (83%)	243 (68%)		31 (9%)	nee	nee
Lone 2011	prospective	246	5 yr	187 (76%)	130 (53%)		70 (28%)	nee	nee
Markle 2011	retrospective	158	1 week	92 (59%)			nr	ja	nee
Chan 2012	prospective	197	1 yr	138 (70%)		59 (30%)	nr	nee	ja
Manchana 2012	retrospective	126	1 yr	77 (61%)	64 (51%)		5 (4%)	nee	nee
Mamik 2013	prospective	50	3 mth				5 (10%)	nee	nee
Geoffrion 2013	retrospective	101	4 wks	79 (78%)			nr	nee	ja
Alperin 2013	retrospective	4019	9 years				12% 1 yr, 24% 9 yrs	nee	nee
Succesfull fitting		3589		2714(76%)					
Continuation		3381			2027 (60%)				

For peer review only - http://bmjopen.bmj.com/site/about/guidelines.xhtml

Appendix 4 Review on risk factors for failure of surgery:

Risk factor	Investigated:	Significant:
Preoperative stage	8	5
Age	8	2
Obesity	7	0
Parity	5	0
Constipation	5	0
Pulmonary disease	5	0
Number of sites involved preoperative	4	1
Menopausal status	4	0
Hysterectomy status	4	0
Concomitent surgery	3	1
Family history	3	1
Complicated delivery	3	0
Diabetes	3	0
Smoking	3	0
Previous incontinence and/or prolapse surgery	2	2
Hiatus genitalis	2	1
Weight	2	1
Any incontinence preoperative	2	1
Delivery mode	2	0
Vaginal delivery	2	0
Hormone replacement therapy	2	0
Previous prolapse surgery	2	0
Surgeons experience	2	0
Abcense of posterior repair	1	1
Sexual activity	1	1
Levator defect	1	1
Height	1	0
Birth weight	1	0
Age at last delivery	1	0
Site of most advanced prolapse	1	0
Surgical approach	1	0
Use of Mesh	1	0
Previous incontinence surgery	1	0
Previous pelvic floor surgery or hysterectomy	1	0
Abdominal hernias	1	0
Cardiovascular disease	1	0
Intense physical exercise	1	0
Heavy lifting	1	0
Heavy lifting or constipation	1	0
Levator muscle contraction	1	0
Weight of the uterus	1	0
Postoperative complications	1	0
Incomplete emptying of bladder	1	0
Fecal incontinence	1	0

For peer review only

Appendix 5 diagram/tabel bezoeken, tijdstippen, onderzoeken

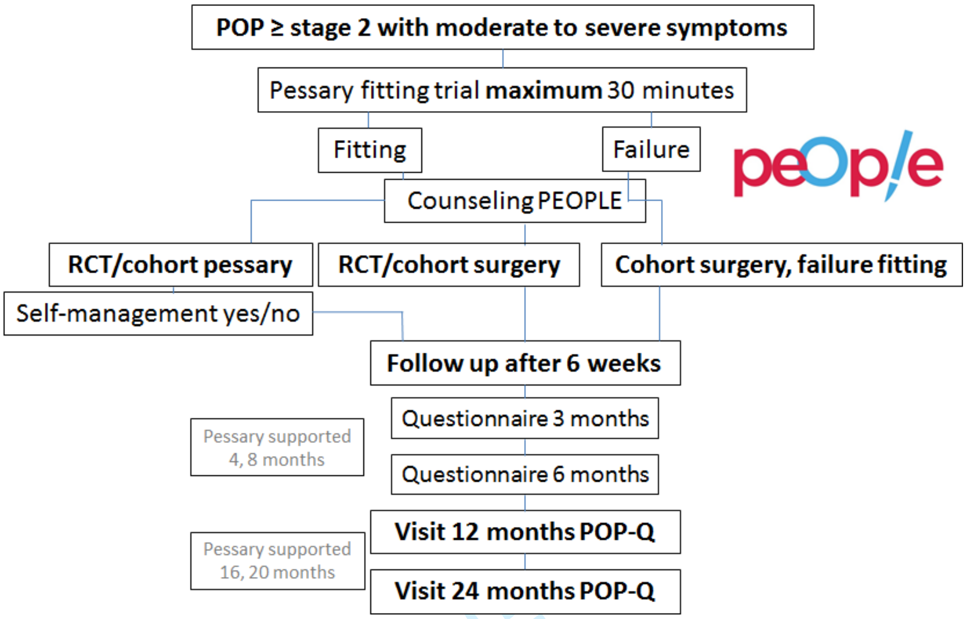

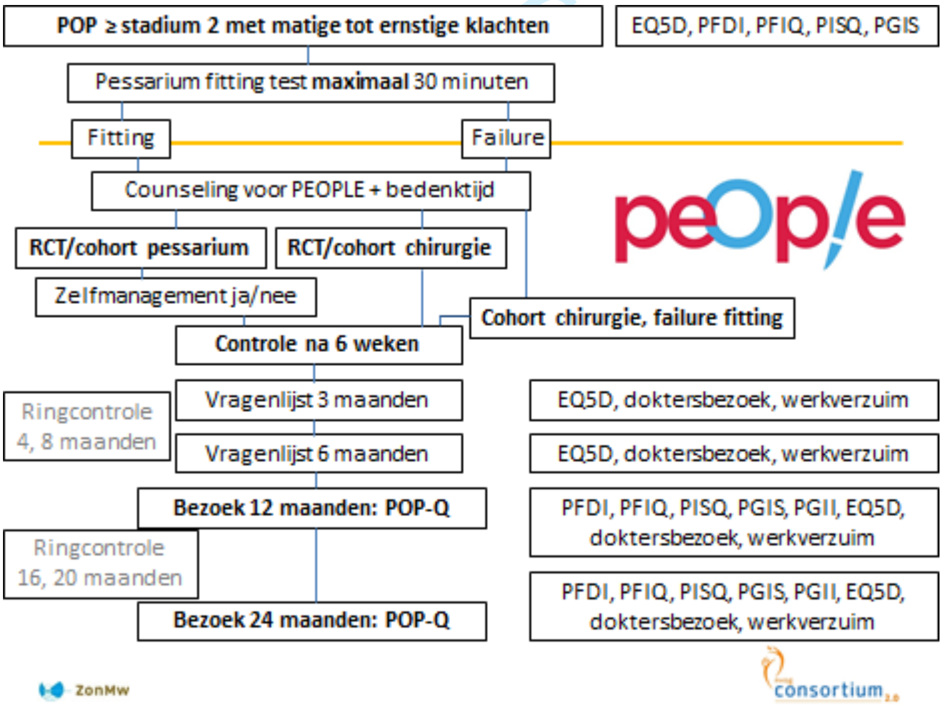

Chirurgie en cohort fitting failure

Contact	Bezoek arts	POPQ	Eq5d doktersbezoek werkverzuim	PFIQ PFDI PISQ PGII PGIS

1. Eerste bezoek	X	X	Eq5D	X (zonder PGII)
2. 6 weken	X			
3. 3 maanden			X	
4. 6 maanden			X	
5. 12 maanden	X	X	X	X
6. 24 maanden	X	X	X	X

Pessarium met zelfmanagement

**Contact**	**Bezoek arts**	**POPQ**	**Eq5d doktersbezoek werkverzuim**	**PFIQ PFDI PISQ PGII PGIS**
1. Eerste bezoek	X	X	Eq5D	X (zonder PGII)
2. 6 weken	X			
3. 3 maanden			X	
4. 6 maanden			X	
5. 12 maanden	X	X	X	X
6. 24 maanden	X	X	X	X

For peer review only

Pessarium zonder zelfmanagement

Contact	Bezoek arts	POPQ	Ringcontrole	Eq5d doktersbezoek werkverzuim	PFIQ PFDI PISQ PGII PGIS
1. Eerste bezoek	X	X		Eq5D	X (zonder PGII)
2. 6 weken	X				
3. 3 maanden				X	
4. 4 maanden	X		X		
5. 6 maanden				X	
6. 8 maanden	X		X		
7. 12 maanden	X	X	X	X	X
8. 16 maanden	X		X		
9. 20 maanden	X		X		
10. 24 maanden	X	X	X	X	X

For peer review only

For peer review only - http://bmjopen.bmj.com/site/about/guidelines.xhtml

1.3 Summary of amendment to study protocol

The main change in the final version is the addition of an observational cohort performed alongside the RCT. We added this observational cohort since many women refused to participate in the RCT due to treatment preference. In case a woman was willing to participate in the study but actively opted for one of two treatment options she was followed in the observational cohort. The same study parameters and follow-up were used in both the trial and observational cohort. See section 2, section 3, section 4.4, section 8.2, section 10, section 11.2

1.3.1 Detailed summary of all amendments

1. Addition of multiple centers for participation.

Added centers:

- Atrium MC Heerlen
- Academisch ziekenhuis Maastricht
- Martini ziekenhuis Groningen
- MST Enschede
- ZGT Almelo / Hengelo
- Deventer ziekenhuis
- Jeroen Bosch ziekenhuis
- Amstelland ziekenhuis
- Tergooi ziekenhuis
- Albert Schweitzer ziekenhuis
- Canisius Wilhelmina ziekenhuis
- Maxima Medisch Centrum
- MCH-Bronovo
- OLVG
- HAGA

2. Change in investigators at the following participating centers:
    - St. Antonius hospital. S. The was replaced by E. Vernooij
    - Canisius hospital. C.F. van Heteren was replaced by K.L. Bos
    - Maastricht University center (MUMC): G. Link was replaced by W.A. Spaans

3. Change in Head of Department of Reproductive Medicine and Gynaecology.

4. Change in Objective.

An observational cohort was added since many women refused to participate in the trial due to treatment preference. At first, women were asked to participate in the trial. In case the woman is willing to participate but actively opts for one of both treatments, she will be followed in a cohort ‘own choice’.

For peer review only

5. Change in study design.

In the first version it is noted that for women with an unsuccessful pessary fitting only baseline characteristics will be recorded. However, these women will be followed in the cohort fitting failure with the same follow-up as for the trial (24-months). Appendix 5 has been noted in more detail.

6. Addition in sample size calculation for observational cohort.

Since we added an observational cohort with women who made their own choice of treatment, we added this to the section sample size calculation. In the cohort we include all patients who are willing to collaborate on this research but have a preference for one of both therapies. We now assume that 70% of the eligible patients object participation in the RCT, and that 90% of them is nevertheless willing to participate in the cohort.

7. Change in self-management of pessary treatment.

In case self-management was performed, women were advised to change their pessary every 4-months, instead of every 1 month.

8. Observational cohort is added in randomization section.

In case the woman is willing to participate but actively opts for one of both treatments, she will also be registered in ALEA. All groups will have the same data collection and follow up as displayed in appendix 5.

9. Observational cohort added in statistical analysis section.

The cohort with patients treated according their preference will be analyzed separately from the randomized trial. The same analysis will be done.

10. Change in exclusion criteria.

Women with a previous hysterectomy were only excluded in case the indication for the hysterectomy was a prolapse.

11. Observational cohort added in recruitment.

In case the woman is willing to participate but actively opts for one of both treatments, she can attend the cohort.

12. Change in monitoring

At first, the monitoring was coordinated by the Staff Member Clinical Research, quality coordinator of division women and baby. Later on, the monitoring was conducted by the Dutch consortium and was executed by a qualified intern monitor.

13. POP-Q only performed at 12- and 24-months follow-up, not at 6 weeks visit.

Demonstrated in the tables listed in appendix 5.

For peer review only - http://bmjopen.bmj.com/site/about/guidelines.xhtml

1.3.2 Table with amendments and corresponding section

**Amendment**	**Corresponding section in the final version 1.22**
1. Addition of multiple centers for participation	First table with project information
2. Change in investigators	First table with project information
3. Change in Head of Department	Protocol signature sheet
4. Change in objective	Section 2
5. Change in study design	Section 3
6. Addition in sample size calculation for observational cohort	Section 4.4
7. Change in self-management of pessary treatment	Section 5.1
8. Observational cohort is added in randomization section	Section 8.2
9. Observational cohort added in statistical analysis section	Section 10
10. Change in exclusion criteria	Section 10.2
11. Observational cohort added in recruitment	Section 11.2
12. Change in monitoring	Section 12.2
13. POP-Q only performed at 12- and 24-months	Appendix 5

For peer review only

For peer review only

BMJ Open

Cost-effectiveness of pessary therapy versus surgery for symptomatic pelvic organ prolapse: an economic evaluation alongside a randomized non-inferiority controlled trial

Journal:	*BMJ Open*
Manuscript ID	bmjopen-2023-075016.R1
Article Type:	Original research
Date Submitted by the Author:	22-Dec-2023

Complete List of Authors:	Ben, Ângela; Vrije Universiteit Amsterdam, Health Sciences<br>van der Vaart, Lisa R.; University of Amsterdam<br>Bosmans, J; VU University Amsterdam<br>Roovers, Jan-Paul W.R.; University of Amsterdam, Department of Obstetrics and Gynecology; Women's Health Bergman Clinics, Department of Gynecology<br>Lagro-Janssen, Antoinette; Radboud university medical center, Department of Primary and Community Care<br>van der Vaart, Carl H.; University Medical Center Utrecht, Department of Obstetrics and Gynecology; Women's Health Bergman Clinics, Department of Gynecology<br>Vollebregt, Astrid; Spaarne Hospital, Department of Obstetrics and Gynecology

**Primary Subject Heading**:	Health economics
Secondary Subject Heading:	Obstetrics and gynaecology
Keywords:	Pelvic Pain, Urogynaecology < GYNAECOLOGY, Health economics < HEALTH SERVICES ADMINISTRATION & MANAGEMENT

SCHOLARONE™
Manuscripts

I, the Submitting Author has the right to grant and does grant on behalf of all authors of the Work (as defined in the below author licence), an exclusive licence and/or a non-exclusive licence for contributions from authors who are: i) UK Crown employees; ii) where BMJ has agreed a CC-BY licence shall apply, and/or iii) in accordance with the terms applicable for US Federal Government officers or employees acting as part of their official duties; on a worldwide, perpetual, irrevocable, royalty-free basis to BMJ Publishing Group Ltd ("BMJ") its licensees and where the relevant Journal is co-owned by BMJ to the co-owners of the Journal, to publish the Work in this journal and any other BMJ products and to exploit all rights, as set out in our licence.

The Submitting Author accepts and understands that any supply made under these terms is made by BMJ to the Submitting Author unless you are acting as an employee on behalf of your employer or a postgraduate student of an affiliated institution which is paying any applicable article publishing charge ("APC") for Open Access articles. Where the Submitting Author wishes to make the Work available on an Open Access basis (and intends to pay the relevant APC), the terms of reuse of such Open Access shall be governed by a Creative Commons licence – details of these licences and which Creative Commons licence will apply to this Work are set out in our licence referred to above.

Other than as permitted in any relevant BMJ Author's Self Archiving Policies, I confirm this Work has not been accepted for publication elsewhere, is not being considered for publication elsewhere and does not duplicate material already published. I confirm all authors consent to publication of this Work and authorise the granting of this licence.

For peer review only

Cost-effectiveness of pessary therapy versus surgery for symptomatic pelvic organ prolapse: an economic evaluation alongside a randomized non-inferiority controlled trial

Ângela Jornada Ben MD, Ph.D.[1], Lisa R. van der Vaart MD [2], Judith E. Bosmans Ph.D.[1], Jan-Paul W.R. Roovers MD, Ph.D [2,3], Antoine L. Lagro-Janssen MD, Ph.D [4], Carl H. van der Vaart MD, Ph.D [5,6] Astrid Vollebregt MD, Ph.D.[7]

[1] Department of Health Sciences, Faculty of Science, Vrije Universiteit Amsterdam, Amsterdam Public Health research institute, Amsterdam, The Netherlands.

[2] Department of Obstetrics and Gynecology, Amsterdam Reproduction & Development Research Institute, Amsterdam UMC, University of Amsterdam, Amsterdam, The Netherlands.

[3] Department of Gynecology, Women's Health, Bergman Clinics, Amsterdam, the Netherlands.

[4] Department of General Practice/ Women's studies Medicine, University Medical Center Radboud, Nijmegen, The Netherlands

[5] Department of Obstetrics and Gynecology, University Medical Center Utrecht, Utrecht, The Netherlands.

[6] Department of Gynecology, Women's Health, Bergman Clinics, Hilversum, the Netherlands.

[7] Department of Obstetrics and Gynecology, Spaarne Hospital, Haarlem, The Netherlands.

Corresponding author:

Ângela Jornada Ben. ORCID: 0000-0003-4793-9026. Department of Health Sciences, Faculty Science, Vrije Universiteit Amsterdam, Van der Boechorststraat 7, 1081 BT Amsterdam, The Netherlands. E-mail: a.jornadaben@vu.nl. Telephone: +31 6 25304906.

Orchid numbers:

Ângela J. Ben: 0000-0003-4793-9026

Lisa R van der Vaart: 0000-0003-1195-0047

Judith E. Bosmans: 0000-0002-1443-1026

Jan-Paul W.R. Roovers: 0000-0002-3069-6382

Antoine L. Lagro-Janssen: 0000-0003-2339-3743

Carl H. van der Vaart: 0000-0003-0890-5368

Astrid Vollebregt: 0000-0002-4704-1710

For peer review only - http://bmjopen.bmj.com/site/about/guidelines.xhtml

ABSTRACT

Objective: To evaluate the cost-effectiveness of pessary therapy as an initial treatment option compared to surgery for moderate to severe POP symptoms in secondary care from a healthcare and a societal perspective.

Design: Economic evaluation alongside a multicenter randomized controlled non-inferiority trial with a 24-month follow-up.

Setting: 21 hospitals in the Netherlands, recruitment conducted between 2015 – 2022.

Participants: 1605 women referred to secondary care with symptomatic prolapse stage ≥ 2 were requested to participate. Of them, 440 women gave informed consent and were randomized to pessary therapy (n=218) or to surgery (n=222) in a 1:1 ratio stratified by hospital.

Interventions: Pessary therapy and surgery.

Primary and secondary outcome measures: The Patient Global Impression of Improvement (PGI-I), a 7-point scale dichotomized into successful *vs.* unsuccessful, with a non-inferiority margin of -10%; Quality-Adjusted Life-Years (QALYs) measured by the EQ-5D-3L; healthcare and societal costs were based on medical records and the institute for Medical Technology Assessment (iMTA) questionnaires.

Results: For the PGI-I, the mean difference between pessary therapy and surgery was -0.03 (95% CI, -0.11; 0.06), and -0.01 (95% CI, -0.05; 0.03) for QALYs. In total, 54.1% women randomized to pessary therapy crossed over to surgery, and 3.6% underwent recurrent surgery. Healthcare and societal costs were significantly lower in the pessary therapy (mean difference=-€1780, 95% CI, -€2148; -€1422 and mean difference=-€1826, 95% CI, -€2328; -€1322 respectively). The probability that pessary therapy is cost-effective compared to surgery was 1 at willingness-to-pay thresholds between €0 and €20000/QALY gained from both perspectives.

Conclusions: Non-inferiority of pessary therapy regarding the PGI-I could not be shown and no statistically significant differences in QALYs between interventions were found. Due to significantly lower costs, pessary therapy is likely to be cost-effective compared to surgery as an initial treatment option for women with symptomatic POP treated in secondary care.

Trial registration number: https://trialsearch.who.int/ Identifier: NTR4883.

For peer review only - http://bmjopen.bmj.com/site/about/guidelines.xhtml

Strengths and limitations of this study

- This economic evaluation was performed alongside a multicenter pragmatic randomized controlled trial. The randomization process ensures that groups are comparable and decrease the likelihood of selection bias while the multicenter pragmatic design improves generalizability of results and transferability to clinical practice.
- Validated outcome measures were used and the trial had a long-term follow-up of 2 years.
- Consultations related to both interventions were provided by gynecologists, which may overestimate intervention costs, as these consultations may be provided by trained general practitioners at lower costs.
- Resource utilization related to the specific medical treatment of interventions' complications (e.g., medications), productivity costs related to unpaid work, and informal care costs were not available and, thus, not included in the analysis, which may underestimate total costs.
- Costs were estimated based on the Dutch reimbursement system and can differ from countries which may hamper the generalizability of results to healthcare systems in other countries.

Funding statement: Financial support was provided through a personal grant (receiver: Carl H. van der Vaart) issued by the ZonMW, a Dutch governmental healthcare organization. This study was funded on 26 June 2014 (project no. 837002525).

Competing interests statement: Prof. Dr. C.H. van der Vaart reports grants from ZonMW Dutch government institution grant during the conduct of the study.

Patient consent forms: patient consent forms cannot be obtained because the patient cannot be traced due to anonymization of the data.

Data sharing statement: Data is available through **Lisa R van der Vaart (l.r.vdvaart@gmail.com)** upon reasonable request. To gain access, requesters will need to sign an agreement form and confirm that data will be used for the purpose for which access was granted. Stata code are available through the corresponding author upon reasonable request.

Word count: 4259 words.

For peer review only - http://bmjopen.bmj.com/site/about/guidelines.xhtml

INTRODUCTION

Pelvic organ prolapse (POP) is a gynecological condition in which one or more of the pelvic organs (i.e., uterus, rectum, bladder, small bowel) herniate into the vagina due to weakness or damaging of the pelvic floor muscles and ligaments[1,2]. POP symptoms (e.g., urinary, bowel, and sexual dysfunction) are associated with decreased quality of life[3]. The estimated prevalence of patient-reported POP symptoms ranges from 3 to 17.7% and is expected to increase with an aging population. As a result, the demand for care and associated costs are also expected to increase[4].

Effective treatment options for moderate to severe POP symptoms include pessary therapy and surgery[5,6]. However, both treatment options are not equally effective since non-inferiority of pessary therapy compared to surgery has not been shown[7]. A pessary is a silicone flexible device that is inserted into the vagina to support the pelvic organs (i.e., uterus and bladder)[8]. An advantage of pessary therapy is its minimally invasive nature. However, adverse effects (e.g., discomfort, pain, or excessive discharge) may occur in up to 49% of women within 12 to 24 months after fitting a pessary[9,10]. As for the surgery procedure, side-effects may include urinary tract infection and urinary bladder retention which may lead to longer admission hospital stay[7]. A recent observational study in women with a strong treatment preference and a randomized trial (RCT) in women without a preference found a high crossover rate from pessary therapy to surgery of 24% and 54%, respectively[7,9]. Consequently, using pessary therapy as an initial treatment option might delay effective treatment, thereby increasing the demand for care and, thus, healthcare costs. However, using a pessary as a first treatment step would prevent expensive surgery if the pessary therapy relieves women symptoms adequately, making the initial use of pessary therapy potentially cost-effective compared to immediate surgery.

According to a recent systematic review[8], only one model-based economic evaluation based on data from United States conducted more than 10 years ago compared the cost-effectiveness of expectant management, pessary therapy and surgery for POP symptoms [11]. This review reported that both pessary therapy and surgery were cost-effective compared to expectant management[11]. The aim of this study was to further investigate the cost-effectiveness of initial pessary therapy compared to immediate surgery from a healthcare and a societal perspective for moderate to severe POP symptoms with 2 years of follow-up. This study was performed alongside a non-inferiority randomized trial, of which the results have recently been published[7].

For peer review only

For peer review only - http://bmjopen.bmj.com/site/about/guidelines.xhtml

METHODS

Study design

An economic evaluation was conducted alongside a non-inferiority randomized controlled trial (RCT) comparing pessary therapy and surgery as an initial treatment for moderate to severe POP in secondary care, the PEOPLE project. The health economic analysis plan is available in the study protocol provided as Supplementary file 1. Participants were recruited between March 2015 and November 2019, the follow-up ended in June 2022. Detailed information about the PEOPLE project is published elsewhere[7,9,12]. This study was approved by the Medical Ethical Committee of the University Medical Center Utrecht (METC protocol number 14-533/M). No substantial changes were made to the protocol after the commencement of the RCT[7,12]. This economic evaluation is reported according to the Consolidated Health Economic Evaluating Reporting Standards statement[13]. All participants provided written informed consent.

Study population

Women with POP symptoms who were referred by their general practitioner (GP) to secondary care, were eligible for participation[7]. Inclusion criteria were POP stage ≥2 according to the Pelvic Organ Prolapse Quantification (POP-Q) system[14] and moderate to severe POP symptoms, defined as a prolapse domain score of >33 on the validated original Urinary Distress Inventory[15]. Exclusion criteria were prior prolapse or incontinence surgery, probability of future childbearing, insufficient knowledge of the Dutch language, comorbidity causing increased surgical risks, major psychiatric illness and prior pessary use[7]. Participants had to successfully complete a 30-minute pessary fitting trial to be eligible for randomization. After informed consent was signed, participants were randomly allocated to either pessary therapy or surgery in a 1:1 ratio[7]. Randomization used random permuted block sizes of 2 and 4 and was stratified by center. Due to the nature of the treatment, treatment allocation was not concealed. Women who actively opted for a treatment were asked to participate in an observational cohort performed alongside the RCT, their data were not included in economic evaluation, but published in another article[9]. Detailed information about study design and randomization can be found elsewhere[7,12].

For peer review only - http://bmjopen.bmj.com/site/about/guidelines.xhtml

Setting and location

Twenty-one Dutch hospitals participated in this multicenter RCT. In the Netherlands, women with moderate to severe POP symptoms are generally referred to secondary care. Treatment options in secondary care include pessary therapy or surgery, which are both reimbursed by the Dutch healthcare system. All gynecologist fitted at least 100 pessaries and performed 100 POP surgeries prior to study initiation.

Comparators

Pessary therapy

Two main types of pessary therapy were offered to participants, namely, supportive (i.e., ring) and occlusive (i.e., space filling)[16]. The pessary fitting was considered successful if the patient felt comfortable with the pessary in situ and if there was no pessary expulsion 30 minutes after fitting[7]. All women received verbal and written instructions on self-management of pessary therapy[7]. If self-management was not possible or preferred, an additional follow-up consultation with their gynecologist or GP was scheduled every four months for pessary cleaning and vaginal inspection[7]. In case women performed self-management, the frequency of cleaning was left to their personal preference, however it was advised to clean their pessary at least every 4 months. Women were instructed to return to the hospital if they experienced any complaint or adverse events due to pessary therapy[7].

Surgery

Surgical intervention included a range of surgical procedures for the correction of three main types of prolapse that can occur individually or simultaneously, namely, 1) uterine descent 2) cystocele, and/or 3) rectocele[7]. For a cystocele or rectocele, respectively a conventional anterior- or posterior colporrhaphy was the standard technique. For a uterine descent, uterine preserving techniques or a vaginal hysterectomy was performed[7]. All surgical interventions were performed following Dutch guidelines recommendations[7,17]. Decisions on which surgical technique was performed was decided in a shared-decision manner between gynecologist and participant[7]. Women were instructed to return to the hospital if they experienced any complaint or adverse events.

For peer review only - http://bmjopen.bmj.com/site/about/guidelines.xhtml

Study perspective, time horizon, and discount rate

This economic evaluation was conducted from a healthcare and a societal perspective over a time horizon of 24 months based on the literature and as recommended by the National Institute for Health and Clinical Excellence[6,8,18]. The healthcare perspective included costs related to interventions (pessary therapy and surgery) and healthcare utilization costs. The societal perspective included costs related to absenteeism from paid work in addition to the interventions' costs and healthcare utilization costs. Discount rates of 1.5% and 4% were applied to QALY and costs, respectively after the first year of the RCT as recommended by the Dutch Guideline for Economic Evaluations in healthcare[19].

Outcomes

Health outcomes

Two health outcomes were used for the trial-based economic evaluation: patient-reported subjective improvement and Quality-Adjusted Life-Years (QALYs). Subjective improvement was measured with the Patient Global Impression of Improvement (PGI-I)[20] scale at 12- and 24-month follow-up. The PGI-I is a single question, seven-point Likert response scale ranging from '*very much worse*' to '*very much better*'[20]. Subjective improvement was defined as a response of '*much better*' or '*very much better*'[21]. The PGI-I is a validated, easy to apply questionnaire, and it strongly correlates with other validated outcome measures such as the POP-Q system[14,20]. The primary analysis of PGI-I compared with surgery was presented in a previous publication in which its non-inferiority could not be shown[7]. This secondary analysis was performed as planned in the study protocol (Supplementary file 1)[22].

The QALY incorporates the impact of interventions on both the quantity and quality of life[23]. It is a routinely used health outcome measure in economic evaluations because it allows decision-makers to compare the cost-effectiveness of a range of interventions for different health conditions[23]. In this study, QALYs were calculated based on the EQ-5D-3L data collected at baseline, 3-, 6-, 12-, and 24-month follow-up. The EQ-5D-3L includes five dimensions of quality of life (i.e., mobility, self-care, usual activities, pain/discomfort and anxiety/depression) with three response levels (i.e., no problems, some problems or extreme problems/ unable to) describing 243 health states[24]. The participants' health states obtained from EQ-5D-3L responses

For peer review only - http://bmjopen.bmj.com/site/about/guidelines.xhtml

were converted into utility values using the Dutch tariff[25]. The utility values were used to calculate QALYs by means of linear interpolation (i.e., the duration of a health state is multiplied by the utility related to that health state)[26].

Cost outcomes

All costs were indexed to 2022 using the consumer price index in the Netherlands(www.cbs.nl)[27].

Intervention costs

Intervention costs of the pessary therapy included those related to the pessary device and one gynecologist consultation for the pessary placement at baseline. Unit prices of pessary therapy were based on the Dutch costing guideline[28] and on market prices (Supplementary file 2). For the surgery group, intervention costs consisted of the surgical procedures conducted at baseline. Unit prices of surgical procedures was based on the Diagnosis Treatment Combination (in Dutch *Diagnose Behandeling Combinatie*, DBC)[29]. The DBC is a care path that includes diagnostic procedures and care activities delivered at hospital and immediate follow-up up to 6 weeks (42 days)[29]. The average national prices are calculated for each DBC code based on all declared reimbursements that have been submitted to the DBC Information System (DIS) by healthcare providers in hospital care. A detailed description of the resources used in the interventions and their respective unit costs is presented in Supplementary file 2.

Healthcare utilization costs

Healthcare utilization was collected during follow-up visits at hospital centers including information on the number of scheduled consultations with gynecologists and extra consultations due to complications, the number of days of hospital readmissions due to complications, the type/number of surgeries after pessary, the type/number of re-surgeries, the number of times a pessary device was changed, and the use of a pessary after initial surgery. Additionally, an adapted version of the iMTA Medical Consumption Questionnaire (iMCQ)[30] was used to measure non-intervention related healthcare utilization at 3-, 6-, 12-, and 24-month follow-up. Healthcare utilization included resources used in primary care (i.e., the number of GP consultations and other healthcare professionals due to POP complaints), and in secondary care apart from study scheduled consultations (i.e., the number of extra consultations with other medical specialists due to POP complaints).

For peer review only - http://bmjopen.bmj.com/site/about/guidelines.xhtml
For peer review only

The number of healthcare resources used was then multiplied by their respective unit prices. Unit of prices of healthcare resources were based on the Dutch costing guideline[28] (Supplementary file 2).

Lost productivity costs

Absenteeism from paid work due to POP symptoms was measured using a adapted version of the iMTA Productivity Cost Questionnaire[31] at 3-, 6-, 12-, and 24-month follow-up. The friction cost approach (FCA) was used to calculate sickness absenteeism costs related to paid work[32]. The FCA assumes that sickness absenteeism costs are limited to the period needed to replace an absent, sick worker (the friction period), which has been estimated to be 12 weeks (85 days) in the Netherlands[32]. Gender-specific estimates of the mean wages of the Dutch population were used to calculate sickness absenteeism costs from paid work[28].

Cost-effectiveness analysis

Analyses were performed according to the intention-to-treat principle using StataSE V.17. As recommended by Faria et al,[33] mean imputation was used to impute missing values at baseline (i.e., parity, Patient Global Impression of Severity [PGIS], Pelvic Floor Distress Inventory [PFDI-20], Pelvic Organ Prolapse Distress Inventory [POPDI-6], Colorectal-Anal Distress Inventory [CRADI-8], Urinary Distress Inventory [UDI-6], and EQ-5D utility values). Subsequently, multiple imputation by Chained Equations (MICE) was used to impute follow-up missing data. The multiple imputation model included treatment group and hospital center, variables associated with missingness (i.e., Body Mass Index [BMI], number of re-surgeries, number of consultations, and family history of prolapse), outcomes, and potential confounders (i.e., age, history of gynecological operations, prolapse stage, menopausal state, and risk-increasing aspects)[34]. Risk-increasing aspects was a combined variable that included at least one of the following comorbidities: smoking status, antidepressants use, obesity, diabetes mellitus, and chronic pulmonary disease. Predictive Mean Matching was used in the imputation procedure to account for the skewed distribution of the costs[35]. Missing cost data were imputed at the level of resource use by time point (i.e., number of consultations, working hours and absenteeism hours). The number of imputations was increased until there was a loss of efficiency of ≤5%, resulting in ten imputed datasets[36]. The ten imputed datasets were analyzed separately and estimates were pooled using Rubin's rules[37].

For peer review only - http://bmjopen.bmj.com/site/about/guidelines.xhtml

Multilevel regression models were used to estimate the difference in costs and effects between the groups to account for the fact that randomization was stratified by hospital center[38]. For cost and effect outcomes, a two-level structure was used where participants and hospital center represented the first and second level, respectively. All analysis models were adjusted for relevant confounders. The PGI-I model was adjusted for PGI-I at 12-month, risk-increasing aspects, and prolapse stage. The QALY model was adjusted for baseline utility values[39], PGI-I at 12-month, risk-increasing aspects, prolapse stage, and number of extra consultations due to complications. Healthcare and societal costs models were adjusted for age, PGI-I at 24-month, menopause state, risk-increasing aspects, and prolapse stage. PGI-I at 12-month and extra consultations due to complications, were included in the models as confounders as they were related to the outcomes and treatment allocation. A non-inferiority margin of 10% risk difference (one-sided 95% CI) was set for the PGI-I outcome based on the expectation that 80% of women would report successful treatment (either pessary therapy or surgery) after 2 years[12,40,41].

Incremental cost-effectiveness ratios (ICERs) were calculated by dividing the difference in costs between the pessary therapy and surgery by their difference in effects resulting in an estimate of the costs per unit of effect gained. Bias-corrected accelerated bootstrapping with 5000 replications was used to estimate the joint uncertainty surrounding differences in costs and effects. Bootstrapped cost-effect pairs were described and plotted on cost-effectiveness planes (CE-planes)[42]. Non-inferiority with regard to cost-effectiveness was demonstrated using a one-sided α of 2.5%, meaning that 97.5% of the cost-effect pairs have to lie right of the non-inferiority margin for effects[43]. Cost-effectiveness acceptability curves (CEACs) were estimated to show the probability of the pessary therapy being cost-effective compared to surgery for a range of willingness-to-pay (WTP) thresholds (i.e., the maximum amount of money society is willing to pay for a unit of effect)[44]. For QALY, we used a WTP threshold of 20000 €/QALY gained recommended by the Dutch Health Care Institute[45]. As there is no specific WTP threshold for PGI-I, we used a maximum WTP of 5237 €/PGI-I gained. This threshold was based on the average DBC costs of surgical procedures performed for POP symptoms as reported in Supplementary file 2.

For peer review only - http://bmjopen.bmj.com/site/about/guidelines.xhtml

Sensitivity Analysis

Two sensitive analyses (SA) were performed to assess the robustness of the results. SA1 was a complete case analysis, meaning that only observations with complete data were included in the main analysis. A per protocol analysis (SA2) was performed to compare treatment groups including women who completed the treatment to which they were originally allocated.

For peer review only

For peer review only - http://bmjopen.bmj.com/site/about/guidelines.xhtml

Patient and Public Involvement

One major gynecological patient organization in the Netherlands (i.e., BekkenBodem4All) as well as the Dutch Urogynecology Consortium fully agreed on the study protocol and identified the study as highly relevant[12].

For peer review only

For peer review only - http://bmjopen.bmj.com/site/about/guidelines.xhtml

RESULTS

Participants

Of the 1605 women assessed for eligibility, 440 were randomized to either pessary therapy (n=218) or surgery (n=222) as shown in Supplementary file 2. After randomization, one participant was excluded from the surgery group due to prolapse stage 1 resulting in a total of 221 women in this group (Supplementary file 2). Baseline incomplete data were imputed for parity (n=4, 0.9%), PFDI-20 (n=22, 5.0%), POPDI-6 (n=21, 4.8%), CRADI-8 (n=21, 4.8%), UDI-6 (n=22, 5.0%) and utility values (n=24, 5.5%) (Table 1). Follow-up missing data at 24-months were multiply imputed for PGI-I (n=104, 23.7%), QALY (n=144, 32.8%), healthcare costs (n=160, 36.4%), and societal costs (n=165, 37.6%) (Figure 1). A total of 118 of 218 (54.1%) women randomized to pessary therapy crossed over to surgery, and a total of 8 women out of 221 (3.6%) underwent recurrent surgery. At baseline, no meaningful differences were found between both groups (Table 1).

< Insert Table 1 here >

Effectiveness

In the unadjusted analysis, the lower 95%CI bound of the PGI-I outcome surpassed the non-inferiority margin of -10% (mean difference -0.06, 95% CI, -0.15; 0.04), meaning that non-inferiority of pessary therapy compared to surgery could not be shown (Table 2). After adjusting for confounders, the lower 95% CI bound of the PGI-I outcome still surpassed the non-inferiority margin (mean difference -0.03, 95% CI, -0.11; 0.06, Table 3). There was no statistically significant difference in QALYs between groups neither in the unadjusted analysis (mean difference -0.02, 95% CI, -0.06; 0.02, Table 2) nor the adjusted analysis (mean difference -0.01, 95% CI -0.05; 0.03, Table 3).

< Insert Table 2 here >

For peer review only - http://bmjopen.bmj.com/site/about/guidelines.xhtml

Costs

After 24 months, unadjusted analyses showed there were statistically significant savings in the pessary therapy group compared to the surgery for both total healthcare costs (mean difference -€1850, 95% CI, -€2228; -€1476) and societal costs (mean difference -€1878, 95% CI, -€2395; -€1345) (Table 2). Despite having other surgery options (Supplementary file 2), we used a fixed price of €4640 considering the surgical procedures conducted in the trial. The main cost driver in the surgery group was the intervention costs (€4640, SE=0), while in the pessary therapy group this was secondary costs (€3736, SE=174) (Table 2). Given that half of patients in the pessary group crossed over to surgery (54.1%) and a small proportion of women underwent recurrent surgery in the surgery group (3.6%), secondary costs during follow-up were statistically significantly higher in the pessary therapy group compared to surgery (mean difference €2609, 95% CI, €2232; €2982, Table 2). In the adjusted analysis, mean differences in healthcare and societal costs between groups slightly decreased compared to the unadjusted analysis (Table 3). However, both healthcare and societal costs in the pessary group were still statistically significantly lower than in the surgery group.

< Insert Table 3 here >

Cost-effectiveness analysis

For the PGI-I outcome, the main analysis showed ICERs of 65525 and 67203 from a healthcare and a societal perspective, respectively (Table 3). The positive ICERs are situated in the SW quadrant of the CE plane and indicate that while pessary therapy incurred significantly lower costs (healthcare mean difference -€1780, 95% CI -€2148; -€1422 and societal mean difference -€1826, 95% CI -€2328; -€1322), it was also less effective compared to surgery (mean difference = -0.03, 95% CI -0.11; 0.06), although not statistically significantly so. Most bootstrapped cost-effect pairs were situated on the right of the non-inferiority margin for effects (95.5%) and in the southern quadrants of the CE-Plane meaning that pessary therapy would save costs at an acceptable loss of effect in terms of PGI-I (Figure 1[1A] and [2A]). Due to statistically significant lower healthcare and societal costs in the pessary therapy group compared to surgery, CEACs showed that the probability of the pessary therapy being cost-effective compared to surgery was 1 at relevant WTP values (Figure 1 [1B] and [2B]). This means that the pessary therapy as an initial treatment option has a 100% probability of being cost-effective compared to immediate surgery.

For peer review only - http://bmjopen.bmj.com/site/about/guidelines.xhtml

< Insert Figure 1 here >

For QALYs, similar to PGI-I the positive, ICERs indicate that pessary therapy is less expensive and less effective (mean difference -0.01, 95% CI -0.05; 0.03) than surgery. However, the difference in QALYs was small and less than the commonly used minimally clinically important difference (i.e., 0.06)[46,47] meaning that pessary therapy would save costs without considerably reducing health-related quality of life. The majority of the bootstrapped cost-effect pairs was in the southern quadrants of the CE-plane (70%) meaning that on average the pessary therapy was less costly than surgery (Figure 2 [1A] and [2A]). The probability that pessary therapy being cost-effective compared to surgery at all WTP thresholds was 1 from both perspectives (Figure 2 [1B] and [2B]).

< Insert Figure 2 here >

Sensitivity analysis

SA1 including only complete cases showed similar results compared to the main analysis, although the direction of the difference in QALYs turned around (Table 3). However, the difference was still small and neither statistically significant nor clinically relevant. This explains the negative ICER and the shift in the distribution of the bootstrapped cost-effect pairs between South-West and South-East quadrants of the CE-plane compared to the main analysis. In SA2, which included women that received their originally allocated intervention with fully imputed data on the PGI-I, (pessary therapy n=81, surgery n=190), the differences in costs and PGI-I between pessary and surgery increased and in QALY decreased compared to the main analysis (Table 3). However, this did not affect the cost-effectiveness results.

For peer review only - http://bmjopen.bmj.com/site/about/guidelines.xhtml

DISCUSSION

Main findings

This economic evaluation showed that although non-inferiority of pessary therapy with regard to subjective improvement could not be shown which was consistent with primary analysis of PGI-I[7]. Also, there were no statistically significant differences in QALY gained. Despite this, a strategy of initial pessary therapy in women with symptomatic POP is likely to be cost-effective compared to immediate surgery from a healthcare and a societal perspective due to lower costs associated with pessary therapy."

Explanation of the findings and comparison with the literature

For both effect outcomes, the high probability of pessary therapy being cost-effective compared to surgery is explained by the fact that total healthcare and societal costs in the pessary group were statistically significant lower than in the surgery group, despite the high proportion of crossover (54.1%) from participants in the pessary group to surgery.

Recently, Bugge et al. (2022)[8] systematically reviewed the (cost-)effectiveness of pessary therapy for managing POP symptoms and found only two economic evaluations[11,48]. Of those, only Hullfish et al. (2011)[11] directly compared pessary therapy with surgery. They developed a model-based economic evaluation with 12-month follow-up based on data from the literature, local experience of a single institution, and expert opinion. Results showed that for lower WTP thresholds (i.e. from 0 to 5600 $/QALY gained) pessary is cost-effective compared to surgery and for higher WTP thresholds (i.e., from 5600 to roughly 20000 $/QALY gained) not anymore. Our results, based on randomized data, showed that pessary therapy is cost-effective compared to surgery at similar WTP thresholds (i.e. 0 to 20000 €/QALY gained).

Strengths and Limitations

One of the strengths of this study is that it was performed alongside a multicenter pragmatic randomized controlled trial. The randomization process ensures that groups are comparable and decrease the likelihood of selection bias[49] while the multicenter pragmatic design improves generalizability of results and transferability to clinical practice. Validated outcome measures were used and the trial had a ~~long-term~~ follow-

For peer review only - http://bmjopen.bmj.com/site/about/guidelines.xhtml

up of 2 years. However, since POP symptoms can relapse over time, studies including a longer follow-up (e.g., more than 2 years) are needed. This study has a number of limitations. First, productivity costs related to unpaid work such as number of hours spent in unpaid activities (e.g., voluntary and housework) and informal care (e.g., care provided by family and friends while being sick) were not collected. Since the mean age of the participants is 65 years (the retirement age in the Netherlands until 2024), these costs are likely to be more relevant than lost productivity related to paid work. Second, consultations related to both interventions were provided by gynecologists, which may result in an overestimation of intervention costs. This may not be representative for healthcare systems in other countries, as these consultations may be provided by trained GPs at lower costs (i.e., €39 by a GP vs €109 by a medical specialist). Third, healthcare resource utilization related to the specific medical treatment of complications (e.g., medications) was not collected. Only costs related to readmissions and extra complications due to complications were included in the analysis. This may underestimate healthcare utilization costs. Fourth, the proportion of missing data on the outcomes was between 24 to 38%. To deal with this issue, multiple imputation of missing values were performed which is the recommended method to handle missing data in trial-based economic evaluations to produce valid estimates[33,50,51]. In addition, a sensitivity analysis including complete cases was performed to evaluate the robustness of findings, showing that results were not affected. Fifth, costs were estimated based on the Dutch reimbursement system and can differ from countries which may hamper the generalizability of results to healthcare systems in other countries.

Implications for practice and future research

A considerable number of women declined to participate in the RCT (n=553, Figure 1). These women were offered the possibility to participate in a prospective cohort[9]. The majority of participants in the prospective cohort opted for a pessary therapy as initial treatment option (62.2%)[9]. Compared to participants of the RCT[7], participants in the cohort less often crossed over to surgery (24% vs 54%). In addition, in this cohort, more women reported successful improvement after surgery compared to pessary[9]. This suggests that it is important to consider women's preferences when deciding about the most suitable treatment for their POP symptoms. Future studies should measure costs from a broader perspective than this study did, as relevant

For peer review only - http://bmjopen.bmj.com/site/about/guidelines.xhtml

costs were not considered in the analysis, that is, costs related to follow-up medical treatment, informal care costs and lost productivity costs related to unpaid work (e.g., housework, voluntary work).

For peer review only

For peer review only - http://bmjopen.bmj.com/site/about/guidelines.xhtml

CONCLUSION

Non-inferiority of pessary therapy with regard to the PGI-I could not be shown and there were no statistically significant differences in QALYs between interventions. Due to significantly lower costs, pessary therapy is likely to be cost-effective compared to surgery from a healthcare and a societal perspective as an initial treatment option for women with moderate to severe POP symptoms treated in secondary care compared to immediate surgery. However, considering the high crossover rate from pessary to surgery it is important to consider women's preferences regarding the treatment of their POP systems.

FIGURE 1. COST-EFFECTIVENESS PLANES AND COST-EFFECTIVENESS ACCEPTABILITY CURVES FOR PATIENT GLOBAL IMPRESSION IMPROVEMENT (PGI-I). Cost-effectiveness planes (CE-planes [1A] and [2A]) and cost-effectiveness acceptability curves (CEACs [1B] and [2B]) comparing pessary therapy with surgery for the PGI-I outcome from a healthcare and a societal perspective, respectively. CE-planes show the incremental cost-effectiveness ratio point estimate (ICER, red dot) and the distribution of the 5000 replications of the bootstrapped cost-effective pairs (blue dots). CEACs indicate the probability of pessary therapy being cost-effective compared with surgery (y-axis) for different willingness-to-pay (WTP) thresholds per unit of PGI-I gained (x-axis). The dashed line represents the non-inferiority margin of 10%. [1A] and [2A] show that all of bootstrapped cost-effect pairs were distributed in the southern quadrants of the CE-planed meaning that the pessary therapy is less costly but could also be less and more effective. 95.5% bootstrapped cost-effect pairs are situated on the right of the non-inferiority margin for effects.[1B] and [2B] indicate a steady probability of 1 that the pessary therapy is cost-effective compared with surgery for different WTP thresholds per PGI-I gained. PGI-I is presented as the difference between groups in the proportion of participants reporting improvement.

FIGURE 2. COST-EFFECTIVENESS PLANES AND COST-EFFECTIVENESS ACCEPTABILITY CURVES FOR QUALITY-ADJUSTED LIFE-YEARS (QALY). Cost-effectiveness planes (CE-planes [1A] and [2A]) and cost-effectiveness acceptability curves (CEACs [1B] and [2B]) comparing pessary therapy with surgery for QALY from a healthcare and a societal perspective, respectively. CE-planes show the incremental cost-effectiveness ratio point estimate (ICER, red dot) and the distribution of the 5000 replications of the bootstrapped cost-effective pairs (blue dots). CEACs indicate the probability of pessary therapy being cost-effective compared with surgery (y-axis) for different willingness-to-pay (WTP) thresholds per QALY gained (x-axis). [1A] and [2A] show that all of bootstrapped cost-effect pairs were distributed in the southern quadrants of the CE-planed meaning that the pessary therapy is less costly but could also be less and more effective. [1B] and [2B] indicate a steady probability of 1 that the pessary therapy is cost-effective compared with surgery for different WTP thresholds per QALY gained. PGI-I is presented as the difference between groups in the proportion of participants reporting improvement.

For peer review only - http://bmjopen.bmj.com/site/about/guidelines.xhtml

REFERENCES

1 Aboseif C, Liu P. *Pelvic Organ Prolapse*. StatPearls Publishing 2022. https://www.ncbi.nlm.nih.gov/books/NBK563229/ (accessed 22 August 2022)

2 Harrison BP, Cespedes RD. PELVIC ORGAN PROLAPSE*. *Emergency Medicine Clinics of North America*. 2001;19:781–98.

3 Doaee M, Moradi-Lakeh M, Nourmohammadi A, *et al.* Management of pelvic organ prolapse and quality of life: a systematic review and meta-analysis. *Int Urogynecol J*. 2014;25:153–63.

4 Dieter AA, Wilkins MF, Wu JM. Epidemiological trends and future care needs for pelvic floor disorders. *Current Opinion in Obstetrics and Gynecology*. 2015;27:380–4.

5 Brown HW, Hegde A, Huebner M, *et al.* International urogynecology consultation chapter 1 committee 2: Epidemiology of pelvic organ prolapse: prevalence, incidence, natural history, and service needs. *Int Urogynecol J*. 2022;33:173–87.

6 van Geelen JM, Dwyer PL. Where to for pelvic organ prolapse treatment after the FDA pronouncements? A systematic review of the recent literature. *Int Urogynecol J*. 2013;24:707–18.

7 van der Vaart LR, Vollebregt A, Milani AL, *et al.* Effect of Pessary vs Surgery on Patient-Reported Improvement in Patients With Symptomatic Pelvic Organ Prolapse: A Randomized Clinical Trial. *JAMA*. 2022;328:2312–23.

8 Bugge C, Adams EJ, Gopinath D, *et al.* Pessaries (mechanical devices) for managing pelvic organ prolapse in women. *Cochrane Database of Systematic Reviews*. Published Online First: 2020. doi: 10.1002/14651858.CD004010.pub4

9 van der Vaart L, Vollebregt A, Milani A, *et al.* Pessary or surgery for a symptomatic pelvic organ prolapse: the PEOPLE study, a multicentre prospective cohort study. *BJOG*. 2022;129:820–9.

10 de Albuquerque Coelho SC, de Castro EB, Juliato CRT. Female pelvic organ prolapse using pessaries: systematic review. *Int Urogynecol J*. 2016;27:1797–803.

11 Hullfish KL, Trowbridge ER, Stukenborg GJ. Treatment strategies for pelvic organ prolapse: a cost-effectiveness analysis. *Int Urogynecol J*. 2011;22:507–15.

12 PEOPLE group. Healthcare evaluation in the Netherlands. - PEOPLE Project. 2015. https://www.zorgevaluatienederland.nl/evaluations/people (accessed 11 August 2022)

13 Husereau D, Drummond M, Augustovski F, *et al.* Consolidated Health Economic Evaluation Reporting Standards 2022 (CHEERS 2022) Statement: Updated Reporting Guidance for Health Economic Evaluations. *Value in Health*. 2022;25:3–9.

14 Bump RC, Mattiasson A, Bø K, *et al.* The standardization of terminology of female pelvic organ prolapse and pelvic floor dysfunction. *American Journal of Obstetrics and Gynecology*. 1996;175:10–7.

15 Shumaker SA, Wyman JF, Uebersax JS, *et al.* Health-related quality of life measures for women with urinary incontinence: the Incontinence Impact Questionnaire and the Urogenital Distress Inventory. Continence Program in Women (CPW) Research Group. *Qual Life Res*. 1994;3:291–306.

16 Cundiff GW, Amundsen CL, Bent AE, *et al.* The PESSRI study: symptom relief outcomes of a randomized crossover trial of the ring and Gellhorn pessaries. *American Journal of Obstetrics and Gynecology*. 2007;196:405.e1-405.e8.

For peer review only

17 Federatie Medisch Specialisten. Prolapse: guideline about the best care for patients with prolapse according to current standards. Utrecht, the Netherlands. 2014. https://richtlijnendatabase.nl/richtlijn/prolaps/prolaps_-_startpagina.html (accessed 22 March 2023)

18 Overview | Urinary incontinence and pelvic organ prolapse in women: management | Guidance | NICE. 2019. https://www.nice.org.uk/guidance/ng123 (accessed 19 December 2023)

19 VWS. Guideline for economic evaluations in healthcare - Dutch National Health Care Institute - Ministerie van Volksgezondheid, Welzijn en Sport. 2016. https://english.zorginstituutnederland.nl/publications/reports/2016/06/16/guideline-for-economic-evaluations-in-healthcare (accessed 23 August 2018)

20 Srikrishna S, Robinson D, Cardozo L. Validation of the Patient Global Impression of Improvement (PGI-I) for urogenital prolapse. *Int Urogynecol J*. 2010;21:523–8.

21 Labrie J, Berghmans BLCM, Fischer K, *et al.* Surgery versus Physiotherapy for Stress Urinary Incontinence. *N Engl J Med*. 2013;369:1124–33.

22 ZonMw. ZonMw. https://www.zonmw.nl/nl/ (accessed 18 June 2022)

23 Drummond MF, Sculpher MJ, Torranc GW. *Methods for the economic evaluation of health care programmes*. 3rd ed. Oxford: Oxford University Press 2005.

24 EuroQol Group. EuroQol - a new facility for the measurement of health-related quality of life. *Health Policy*. 1990;16:199–208.

25 Lamers LM, McDonnell J, Stalmeier PFM, *et al.* The Dutch tariff: results and arguments for an effective design for national EQ-5D valuation studies. *Health Economics*. 2006;15:1121–32.

26 Whitehead SJ, Ali S. Health outcomes in economic evaluation: the QALY and utilities. *Br Med Bull*. 2010;96:5–21.

27 CBS. StatLine - Consumentenprijzen. https://opendata.cbs.nl/#/CBS/nl/dataset/83131NED/table?ts=1660927150933 (accessed 21 August 2022)

28 Kanters TA, Bouwmans CAM, van der Linden N, *et al.* Update of the Dutch manual for costing studies in health care. *PLoS One*. 2017;12. doi: 10.1371/journal.pone.0187477

29 Diagnose Behandeling Combinatie (DBC) open data - NZa. https://www.opendisdata.nl/ (accessed 3 September 2022)

30 iMTA. iMTA Medical Consumption Questionnaire. iMTA. https://www.imta.nl/questionnaires/imcq/ (accessed 6 February 2022)

31 Bouwmans C, Krol M, Severens H, *et al.* The iMTA Productivity Cost Questionnaire: A Standardized Instrument for Measuring and Valuing Health-Related Productivity Losses. *Value in Health*. 2015;18:753–8.

32 Koopmanschap MA, Rutten FFH, van Ineveld BM, *et al.* The friction cost method for measuring indirect costs of disease. *Journal of Health Economics*. 1995;14:171–89.

33 Faria R, Gomes M, Epstein D, *et al.* A Guide to Handling Missing Data in Cost-Effectiveness Analysis Conducted Within Randomised Controlled Trials. *Pharmacoeconomics*. 2014;32:1157.

For peer review only - http://bmjopen.bmj.com/site/about/guidelines.xhtml

34 Schulten SFM, Claas-Quax MJ, Weemhoff M, *et al.* Risk factors for primary pelvic organ prolapse and prolapse recurrence: an updated systematic review and meta-analysis. *American Journal of Obstetrics and Gynecology*. 2022;227:192–208.

35 White IR, Royston P, Wood AM. Multiple imputation using chained equations: Issues and guidance for practice. *Statistics in Medicine*. 2011;30:377–99.

36 Madley-Dowd P, Hughes R, Tilling K, *et al.* The proportion of missing data should not be used to guide decisions on multiple imputation. *Journal of Clinical Epidemiology*. 2019;110:63–73.

37 Little RJA, Rubin DB. *Statistical Analysis with Missing Data*. New York, NY, USA: John Wiley & Sons, Inc. 2014.

38 Gomes M, Grieve R, Nixon R, *et al.* Statistical Methods for Cost-Effectiveness Analyses That Use Data from Cluster Randomized Trials: A Systematic Review and Checklist for Critical Appraisal. *Med Decis Making*. 2012;32:209–20.

39 Manca A, Hawkins N, Sculpher MJ. Estimating mean QALYs in trial-based cost-effectiveness analysis: the importance of controlling for baseline utility. *Health Economics*. 2005;14:487–96.

40 Koullali B, van Kempen LEM, van Zijl MD, *et al.* A multi-centre, non-inferiority, randomised controlled trial to compare a cervical pessary with a cervical cerclage in the prevention of preterm delivery in women with short cervical length and a history of preterm birth – PC study. *BMC Pregnancy Childbirth*. 2017;17:215.

41 Non-Inferiority Clinical Trials to Establish Effectiveness Guidance for Industry. *US Department of Health and Human Services Food and Drug Administration Center for Drug Evaluation and Research (CDER) Center for Biologics Evaluation and Research (CBER)*.

42 Black WC. The CE plane: a graphic representation of cost-effectiveness. *Med Decis Making*. 1990;10:212–4.

43 Bosmans JE, de Bruijne MC, van Hout HPJ, *et al.* Practical guidelines for economic evaluations alongside equivalence trials. *Value Health*. 2008;11:251–8.

44 Fenwick E, Claxton K, Sculpher M. Representing uncertainty: the role of cost-effectiveness acceptability curves. *Health Econ*. 2001;10:779–87.

45 ZIN. Zorginstituut Nederland - Richtlijn voor het uitvoeren van economische evaluaties in de gezondheidszorg. Ministerie van Volksgezondheid, Welzijn en Sport. 2016. https://www.zorginstituutnederland.nl/publicaties/publicatie/2016/02/29/richtlijn-voor-het-uitvoeren-van-economische-evaluaties-in-de-gezondheidszorg (accessed 28 March 2018)

46 Walters SJ, Brazier JE. Comparison of the minimally important difference for two health state utility measures: EQ-5D and SF-6D. *Qual Life Res*. 2005;14:1523–32.

47 Luo N, Johnson JA, Coons SJ. Using Instrument-Defined Health State Transitions to Estimate Minimally Important Differences for Four Preference-Based Health-Related Quality of Life Instruments: *Medical Care*. 2010;48:365–71.

48 Panman C, Wiegersma M, Kollen B, *et al.* Two-year effects and cost-effectiveness of pelvic floor muscle training in mild pelvic organ prolapse: a randomised controlled trial in primary care. *BJOG: An International Journal of Obstetrics & Gynaecology*. 2017;124:511–20.

49 Randomization-Based Evaluations. *Multiple Imputation for Nonresponse in Surveys*. Wiley-Blackwell 2008:113–53. https://doi.org/10.1002/9780470316696.ch4

For peer review only - http://bmjopen.bmj.com/site/about/guidelines.xhtml

50 MacNeil Vroomen J, Eekhout I, Dijkgraaf MG, *et al.* Multiple imputation strategies for zero-inflated cost data in economic evaluations: which method works best? *Eur J Health Econ*. 2016;17:939–50.

51 Ben ÂJ, van Dongen JM, Alili ME, *et al.* The handling of missing data in trial-based economic evaluations: should data be multiply imputed prior to longitudinal linear mixed-model analyses? *Eur J Health Econ*. Published Online First: 26 September 2022. doi: 10.1007/s10198-022-01525-y

Authors' contributions: **Ângela Jornada Ben:** Methodology, Data Curation, Formal analysis, and Writing - Original Draft. **Lisa R van der Vaart:** Methodology, Data Curation, Writing - Review & Editing, and Project administration. **Judith E. Bosmans:** Methodology, Writing - Review & Editing, Supervision. **Jan-Paul W.R. Roovers:** Writing - Review & Editing. **Antoine L. Lagro-Janssen:** Writing - Review & Editing. **Carl H. van der Vaart**: Conceptualization, Methodology, Resources, Funding acquisition, and Writing - Review & Editing. **Astrid Vollebregt**: Conceptualization, Methodology, and Writing - Review & Editing, Supervision. All authors have agreed to the submission of the final paper.

Acknowledgments: PEOPLE group: Milani, F, van Bavel, J., Bon G.G., Bongers M.Y., Bos K., Broekman A.M.W., Dietz, V., van Eijndhoven H.W.F., Hakvoort R.A., Janszen E.W.M., Kluivers K., Link G., Massop-Helmink D.S., van der Ploeg J.M., Sikkema F.M., Spaans W.A., van der Ster A., Verheijen-van de Waarsenburg, K., Vernooij M.M.A., Weemhoff, M., A.E. Weis-Potters.

For peer review only

For peer review only - http://bmjopen.bmj.com/site/about/guidelines.xhtml

TABLE 1. BASELINE CHARACTERISTICS OF PARTICIPANTS

Baseline characteristic	Pessary therapy *n = 218*	Surgery *n = 221*
Age (mean (SD))	64.8 (9.5), n=218	64.7(9.2), n=221
Risk-increasing aspects [¥] (n, %)	71 (32.6), n=218	58 (26.2), n=221
History of gynecological surgery (n, %)	22 (10.1), n=218	28 (12.7), n=221
Family history of prolapse (n, %)	106 (48.6), n=218	107 (49.5), n=216
Parity (median (IQR)	2.0 (2-3), n=215	2.0 (2-3), n=220
Postmenopausal (n, %)	186 (92.5), n=201	185 (90.2), n=205
Duration of symptoms in months (median (IQR)	6 (2-24), n=211	6 (3-24), n=216
Vaginal atrophy (n, %)	106 (56.7), n=187	110 (57.3), n=192
Prolapse stage (n, %)		
II (Moderate)	85 (39.0), n=218	102 (46.2), n=221
≥III (Severe)	133 (61.0), n=218	119 (53.9), n=221
PGI-S score[a] (n, %)		
I (Not severe)	13 (6.3), n=205	9 (4.4), n=205
II (Mild)	48 (23.4), n=205	50 (24.4), n=205
III (Moderate)	99 (48.3), n=205	112 (54.6), n=205
IV (Severe)	45 (22.0), n=205	34 (16.6), n=205
PFDI-20 score[b] (n, %)		
POPDI-6 score	29.5 (19.2), n=210	28.7 (15.6), n=208
CRADI-8 score	13.9 (15.1), n=210	12.1 (12.6), n=208
UDI-6 score	26.0 (22.0), n=209	25.2 (20.0), n=208
PFDI-20 total score	69.3 (45.7), n=209	65.9 (37.7), n=208
EQ-5D utility value[c] (mean (SD))	0.87 (0.15), n=209	0.85 (0.15), n=206

SD = standard deviation. n = number of women. % = proportion. IQR = interquartile range. [a]PGIS = Patient Global Impression of Severity: I (not severe), II (mild), III (moderate), IV (severe). [b]PFDI-20 = Pelvic Floor Distress Inventory: the subscale scores range from 0-100 and the total score ranges from 0 to 300. Higher scores indicate more symptom distress. POPDI-6 = Pelvic Organ Prolapse Distress Inventory. CRADI-8 = Colorectal-Anal Distress Inventory. UDI-6 = Urinary Distress Inventory. [c]EQ-5D utility values: the Dutch EQ-5D tariffs range from -0.33 to 1. [¥]presence of 1 or more comorbidities: smoking, use of antidepressants, obesity, diabetes mellitus, chronic pulmonary disease.

For peer review only - http://bmjopen.bmj.com/site/about/guidelines.xhtml

TABLE 2. EFFECTS AND COSTS BY TREATMENT GROUP AND DIFFERENCE AT 24-MONTH FOLLOW-UP

	**Pessary therapy** ***n = 218***	**Surgery** ***n = 221***	**Unadjusted Difference (95% CI)**
**Effects**			
PGI-I, n (%)	164 (75.1%)	179 (80.8%)	-0.06 (-0.15; 0.04)
QALY, mean (SE)	1.80 (0.02)	1.82 (0.01)	-0.02 (-0.06; 0.02)
**Costs, mean (SE)**			
Intervention costs	178 (0.2)	4640 (0)	-4462 (-4463; -4462)
Primary care costs	18 (2)	15 (2)	3 (-3; 8)
Secondary care costs	3736 (174)	1127 (80)	2609 (2232; 2982)
Healthcare costs	3932 (174)	5782 (80)	-1850 (-2228; -1476)
Absenteeism from paid work	362 (117)	390 (120)	-28 (-338; 290)
Societal costs	4294 (227)	6172 (150)	-1878 (-2395, -1345)

PGI-I = Patient Global Impression of Improvement (1=improvement; 0= no improvement). PGI-I is presented as the difference between groups in the proportion of participants reporting improvement. n = number of participants. % = proportion. SE = standard error. Intervention costs in the pessary group = costs of pessary device and pessary placement consultation at baseline. Intervention costs in the surgery group = DBC costs of surgery at baseline which included one follow-up consultation at 6 weeks. Primary care costs = costs of general practitioner or other healthcare professional consultations apart from the pre-scheduled follow-up consultations because of complaints related to pelvic organ prolapse (POP) symptoms. Secondary care costs = costs of follow-up scheduled consultations with gynecologists attended by patients and extra consultations due to complications, costs of hospital readmissions due to complications, surgeries after pessary, re-surgeries, and costs of pessary change

For peer review only - http://bmjopen.bmj.com/site/about/guidelines.xhtml

TABLE 3. RESULTS OF THE COST-EFFECTIVENESS AND COST-UTILITY ANALYSIS

Effect outcome	ΔE (95% CI)	ΔC (95% CI)	ICER	Proportion of bootstrapped cost-effect pairs in the CE-plane			
				NE	SE	SW	NW
**Main analysis – Healthcare Perspective**							
PGI-I, n=439	-0.03 (-0.11; 0.06)	-1780 (-2148; -1422)	65525	0%	24%	76%	0%
QALY, n=439	-0.01 (-0.05; 0.03)	-1780 (-2148; -1422)	154939	0%	30%	70%	0%
**Main analysis – Societal Perspective**							
PGI-I, n=439	-0.03 (-0.11; 0.06)	-1826 (-2328; -1322)	67203	0%	24%	76%	0%
QALY, n=439	-0.01 (-0.05; 0.03)	-1826 (-2328; -1322)	158905	0%	30%	70%	0%
**Sensitivity analysis 1 – Complete Case Analysis – Healthcare Perspective**							
PGI-I, n=259	-0.01 (-0.09; 0.08)	-1961 (-2453; -1585)	283377	0%	38%	62%	0%
QALY, n=256	0.02 (-0.03; 0.06)	-1947 (-2450; -1571)	-119365	0%	79%	21%	0%
**Sensitivity analysis 1 – Complete Case Analysis – Societal Perspective**							
PGI-I, n=254	-0.005 (-0.08; 0.09)	-1872 (-2479; -1243)	389260	0%	38%	62%	0%
QALY, n=252	0.02 (-0.03; 0.06)	-1846 (-2475; -1224)	-99342	0%	81%	19%	0%
**Sensitivity analysis 2 – Per Protocol Analysis – Healthcare Perspective**							
PGI-I, n=271	-0.06 (-0.18; 0.05)	-4413 (-4597; -4326)	69585	0%	12%	88%	0%
QALY, n=271	-0.0001 (-0.04; 0.04)	-4413 (-4597; -4326)	41586588	0%	53%	47%	0%
**Sensitivity analysis 2 – Per Protocol Analysis – Societal Perspective**							
PGI-I, n=271	-0.06 (-0.18; 0.05)	-4772 (-5236; -4495)	75249	0%	12%	88%	0%
QALY, n=271	-0.0001 (-0.04; 0.04)	-4772 (-5236; -4495)	22594796	0%	53%	47%	0%

ΔC= difference in costs in Euros; 95% CI = 95% confidence interval; ΔE= difference in effects; ICER = Incremental Cost-Effectiveness Ratio (€ per unit of effect gained); CE-plane = cost-effectiveness plane showing the difference in costs between pessary therapy and surgery on the y-axis and the difference in effects on the x-axis resulting in four quadrants namely, NE = northeast (pessary therapy more expensive and more effective than surgery); SE = southeast (pessary therapy less expensive and more effective than surgery); SW = southwest (pessary therapy less expensive and less effective than surgery); NW = northwest (pessary therapy more expensive and less effective than surgery).The PGI-I model was adjusted by PGI-I at 12-month, risk-increasing aspects, and prolapse stage. The QALY model was adjusted by baseline utility values, PGI-I at 12-month, risk-increasing aspects, prolapse stage, and number of extra consultations due complications. Healthcare and societal costs models were adjusted by age, PGI-I at 24-month, menopause state, risk-increasing aspects, and prolapse stage. PGI-I is presented as the difference between groups in the proportion of participants reporting improvement.

For peer review only

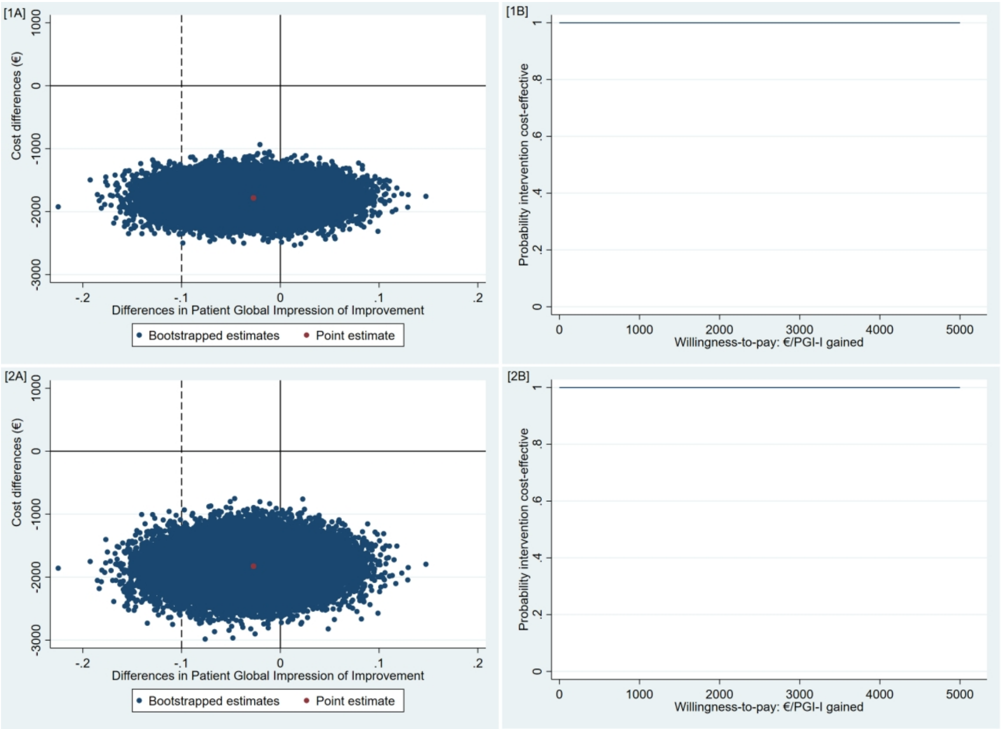

FIGURE 1. COST-EFFECTIVENESS PLANES AND COST-EFFECTIVENESS ACCEPTABILITY CURVES FOR PATIENT GLOBAL IMPRESSION IMPROVEMENT (PGI-I). Cost-effectiveness planes (CE-planes [1A] and [2A]) and cost-effectiveness acceptability curves (CEACs [1B] and [2B]) comparing pessary therapy with surgery for the PGI-I outcome from a healthcare and a societal perspective, respectively. CE-planes show the incremental cost-effectiveness ratio point estimate (ICER, red dot) and the distribution of the 5000 replications of the bootstrapped cost-effective pairs (blue dots). CEACs indicate the probability of pessary therapy being cost-effective compared with surgery (y-axis) for different willingness-to-pay (WTP) thresholds per unit of PGI-I gained (x-axis). The dashed line represents the non-inferiority margin of 10%. [1A] and [2A] show that all of bootstrapped cost-effect pairs were distributed in the southern quadrants of the CE-planed meaning that the pessary therapy is less costly but could also be less and more effective. 95.5% bootstrapped cost-effect pairs are situated on the right of the non-inferiority margin for effects.[1B] and [2B] indicate a steady probability of 1 that the pessary therapy is cost-effective compared with surgery for different WTP thresholds per PGI-I gained. PGI-I is presented as the difference between groups in the proportion of participants reporting improvement.

1715x1247mm (38 x 38 DPI)

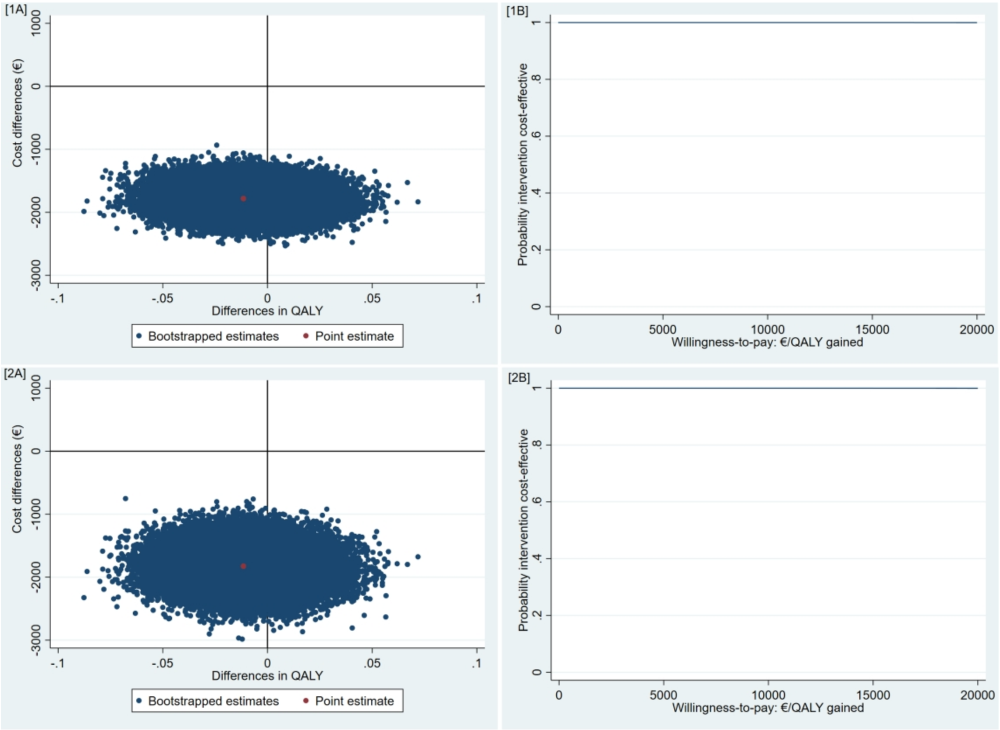

FIGURE 2. COST-EFFECTIVENESS PLANES AND COST-EFFECTIVENESS ACCEPTABILITY CURVES FOR QUALITY-ADJUSTED LIFE-YEARS (QALY). Cost-effectiveness planes (CE-planes [1A] and [2A]) and cost-effectiveness acceptability curves (CEACs [1B] and [2B]) comparing pessary therapy with surgery for QALY from a healthcare and a societal perspective, respectively. CE-planes show the incremental cost-effectiveness ratio point estimate (ICER, red dot) and the distribution of the 5000 replications of the bootstrapped cost-effective pairs (blue dots). CEACs indicate the probability of pessary therapy being cost-effective compared with surgery (y-axis) for different willingness-to-pay (WTP) thresholds per QALY gained (x-axis). [1A] and [2A] show that all of bootstrapped cost-effect pairs were distributed in the southern quadrants of the CE-planed meaning that the pessary therapy is less costly but could also be less and more effective. [1B] and [2B] indicate a steady probability of 1 that the pessary therapy is cost-effective compared with surgery for different WTP thresholds per QALY gained. PGI-I is presented as the difference between groups in the proportion of participants reporting improvement.

1715x1252mm (38 x 38 DPI)

PEOPLE study

Pessary or Surgery for a Symptomatic Pelvic Organ Prolapse

Study protocol

For peer review only

For peer review only - http://bmjopen.bmj.com/site/about/guidelines.xhtml

Table of contents

1. Study protocols 3

1.1 Original study protocol...4

1.2 Final study protocol 55

1.3 Summary of amendments to study protocol.. 107

1.3.1 Detailed summary of all amendments 107

1.3.2 Table with amendments and corresponding section 109

For peer review only - http://bmjopen.bmj.com/site/about/guidelines.xhtml

1. Study protocols

Original study protocol:	Version 1.5, November 2014
Final study protocol:	Version 1.22, February 2018

For peer review only

For peer review only - http://bmjopen.bmj.com/site/about/guidelines.xhtml

1.1 Original study protocol

Pessary or surgery for symptomatic pelvic organ prolapse

Version 1.~~4~~ 5 ~~October~~ November 2014

For peer review only - http://bmjopen.bmj.com/site/about/guidelines.xhtml

PROTOCOL TITLE 'Pessary or surgery for symptomatic pelvic organ prolapse'

**Protocol ID**	**2014 / 1.4**
**Short title**	**Pessary or surgery for symptomatic pelvic organ prolapse**
**EudraCT number**	***Not applicable***
**Version**	**1.45**
**Date**	**October November 2014**
**Coordinating investigator/project leader**	**Prof. Dr. C.H. van der Vaart, gynaecologist**<br>**University Medical Centre Utrecht**
**Principal investigator(s) (in Dutch: hoofdonderzoeker/ uitvoerder)**	**Dr. A. Vollebregt, gynaecologist**<br>**Spaarne Hospital**<br><br>**M.K. van de Waarsenburg, MD**<br>**University Medical Centre Utrecht**
**Multicenter: per site**	**Prof. Dr. C.H. van der Vaart, UMC Utrecht**<br>**Dr. A.L. Milani, Reinier de Graaf Gasthuis**<br>**Dr. A. Vollebregt, Spaarne Hospital Hoofddorp**<br>**Dr. J.P.W.R. Roovers, Academic Medical Center**<br>**Dr. K.B. Kluivers, Radboud UMC Nijmegen**<br>**Dr. V. Dietz, Catharina Hospital Eindhoven**<br>**Dr. H.W.F. van Eijndhoven, Isala Clinic Zwolle**<br>**Dr. H.S. The, Sint Antonius Hospital Nieuwegein**<br>**Dr. R.P. Schellart, Kennemer Gasthuis Haarlem**<br>**Drs. A.M.W. Broekman, Sint Franciscus Gasthuis Rotterdam**<br>**Drs. J. van Bavel, Amphia Hospital Breda**
**Sponsor (in Dutch: verrichter/opdrachtgever)**	**Prof. Dr. C.H. van der Vaart, gynaecologist**
**Subsidising party**	**ZonMw Project nr 837002525**
**Independent expert (s)**	**Dr. R.P. Zweemer**<br><br>**University Medical Centre Utrecht**

For peer review only - http://bmjopen.bmj.com/site/about/guidelines.xhtml

**Laboratory sites <*if applicable*>**	***Not applicable***
**Pharmacy <*if applicable*>**	***Not applicable***

For peer review only

PROTOCOL SIGNATURE SHEET

Name	Signature	Date
**Head of Department:**<br><br>**Prof. Dr. B.C.J.M. Fauser**<br>**Department of Reproductive Medicine and Gynaecology**<br>**University Medical Centre Utrecht**		
**[Coordinating Investigator/Project leader/Principal Investigator]:**<br><br>**Prof. Dr. C.H. van der Vaart, gynaecologist**<br>**University Medical Centre Utrecht**		

For peer review only - http://bmjopen.bmj.com/site/about/guidelines.xhtml

TABLE OF CONTENTS

1. INTRODUCTION AND RATIONALE 9
2. OBJECTIVES 13
3. STUDY DESIGN 14
4. STUDY POPULATION 15
4.1 Population (base) 15
4.2 Inclusion criteria 15
4.3 Exclusion criteria 15
4.4 Sample size calculation 15
5. TREATMENT OF SUBJECTS 17
5.1 Investigational product/treatment 17
5.2 Use of co-intervention (if applicable) 17
5.3 Escape medication (if applicable) 18
6. INVESTIGATIONAL PRODUCT 19
6.1 Name and description of investigational product(s) 19
6.2 Summary of findings from non-clinical studies 19
6.3 Summary of findings from clinical studies 19
6.4 Summary of known and potential risks and benefits 20
6.5 Description and justification of route of administration and dosage 20
6.6 Dosages, dosage modifications and method of administration 20
6.7 Preparation and labelling of Investigational Medicinal Product 20
6.8 Drug accountability 20
7. NON-INVESTIGATIONAL PRODUCT 21
7.1 Name and description of non-investigational product(s) 21
7.2 Summary of findings from non-clinical studies 21
7.3 Summary of findings from clinical studies 21
7.4 Summary of known and potential risks and benefits 21
7.5 Description and justification of route of administration and dosage 21
7.6 Dosages, dosage modifications and method of administration 21
7.7 Preparation and labelling of Non Investigational Medicinal Product 21
7.8 Drug accountability 21
8. METHODS 22
8.1 Study parameters/endpoints 22
8.1.1 Main study parameter/endpoint 22
8.1.2 Secondary study parameters/endpoints (if applicable) 22
8.1.3 Other study parameters (if applicable) 25
8.2 Randomisation, blinding and treatment allocation 25
8.3 Study procedures 26
8.4 Withdrawal of individual subjects 27
8.4.1 Specific criteria for withdrawal (if applicable) 27

For peer review only - http://bmjopen.bmj.com/site/about/guidelines.xhtml

8.5 Replacement of individual subjects after withdrawal ... 27
8.6 Follow-up of subjects withdrawn from treatment ... 27
8.7 Premature termination of the study ... 27
9. SAFETY REPORTING ... 28
9.1 Section 10 WMO event ... 28
9.2 AEs, SAEs and SUSARs ... 28
9.2.1 Adverse events (AEs) ... 28
9.2.2 Serious adverse events (SAEs) ... 28
9.2.3 Suspected unexpected serious adverse reactions (SUSARs) ... 29
9.3 Annual safety report ... 29
9.4 Follow-up of adverse events ... 29
9.5 [Data Safety Monitoring Board (DSMB) / Safety Committee] ... 29
10. STATISTICAL ANALYSIS ... 30
10.1 Primary study parameter(s) ... 30
10.2 Secondary study parameter(s) ... 30
10.3 Other study parameters ... 31
10.4 Interim analysis (if applicable) ... 32
11. ETHICAL CONSIDERATIONS ... 33
11.1 Regulation statement ... 33
11.2 Recruitment and consent ... 33
11.3 Objection by minors or incapacitated subjects (if applicable) ... 33
11.4 Benefits and risks assessment, group relatedness ... 33
11.5 Compensation for injury ... 33
11.6 Incentives (if applicable) ... 33
12. ADMINISTRATIVE ASPECTS, MONITORING AND PUBLICATION ... 35
12.1 Handling and storage of data and documents ... 35
12.2 Monitoring and Quality Assurance ... 35
12.3 Amendments ... 35
12.4 Annual progress report ... 35
12.5 End of study report ... 35
12.6 Public disclosure and publication policy ... 36
13. STRUCTURED RISK ANALYSIS ... 37
13.1 Potential issues of concern ... 37
13.2 Synthesis ... 37
14. REFERENCES ... 38
Appendix 1… ... 42
Appendix 2… ... 43
Appendix 3… ... 45
Appendix 4… ... 46
Appendix 5… ... 47

For peer review only

For peer review only - http://bmjopen.bmj.com/site/about/guidelines.xhtml

LIST OF ABBREVIATIONS AND RELEVANT DEFINITIONS

ABR	ABR form, General Assessment and Registration form, is the application form that is required for submission to the accredited Ethics Committee (In Dutch, ABR = Algemene Beoordeling en Registratie)
AE	Adverse Event
AR	Adverse Reaction
CA	Competent Authority
CCMO	Central Committee on Research Involving Human Subjects; in Dutch: Centrale Commissie Mensgebonden Onderzoek
CV	Curriculum Vitae
DSMB	Data Safety Monitoring Board
EU	European Union
EudraCT	European drug regulatory affairs Clinical Trials
GCP	Good Clinical Practice
IB	Investigator's Brochure
IC	Informed Consent
IMP	Investigational Medicinal Product
IMPD	Investigational Medicinal Product Dossier
METC	Medical research ethics committee (MREC); in Dutch: medisch ethische toetsing commissie (METC)
(S)AE	(Serious) Adverse Event
SPC	Summary of Product Characteristics (in Dutch: officiële productinfomatie IB1-tekst)
Sponsor	The sponsor is the party that commissions the organisation or performance of the research, for example a pharmaceutical company, academic hospital, scientific organisation or investigator. A party that provides funding for a study but does not commission it is not regarded as the sponsor, but referred to as a subsidising party.
SUSAR	Suspected Unexpected Serious Adverse Reaction
Wbp	Personal Data Protection Act (in Dutch: Wet Bescherming Persoonsgevens)
WMO	Medical Research Involving Human Subjects Act (in Dutch: Wet Medisch-wetenschappelijk Onderzoek met Mensen

For peer review only - http://bmjopen.bmj.com/site/about/guidelines.xhtml

SUMMARY

Rationale: Moderate to severe pelvic organ prolapse symptoms can be treated with pessary or surgery. Both treatments appear to be effective, but have not been compared directly.

Hypothesis: The strategy of pessary as initial therapy is as effective as direct surgery for moderate to severe POP, but it is associated with lower costs.

Objective: The primary objective is to compare the effectiveness and cost-effectiveness of pessary versus surgery as initial treatment for moderate to severe symptomatic pelvic organ prolapse (POP) in women at two year after initiation of treatment. The secondary objective is the development of a prediction model for failure of pessary use and surgery within 2 years.

Study design: Cohort study with embedded randomized controlled trial.

Study population: Treatment naïve women with POP who present with moderate to severe symptoms.

Intervention (if applicable): Pessary therapy or vaginal POP surgery.

Main study parameters/endpoints:

Primary outcome: Global impression of improvement of POP symptoms at 24 months measured with PGI-I

Secondary outcomes:

- Changes in symptom bother and disease-specific quality of life at 12 and 24 months follow-up
- Changes of sexual function at 12 and 24 months follow-up
- Changes in general quality of life at 3, 6, 12 and 24 months of follow up
- Adverse events/complications related to both treatment strategies during the study period
- Development of prediction model to identify factors for failing of pessary and surgery.
- Costs-effectiveness analyses

Nature and extent of the burden and risks associated with participation, benefit and group relatedness: Both treatment arms are routine treatments in the Netherlands. Patients in the RCT can have the risks of surgery instead of the risks from pessary therapy.

For peer review only - http://bmjopen.bmj.com/site/about/guidelines.xhtml

1. INTRODUCTION AND RATIONALE

Problem definition

Female pelvic organ prolapse (POP) is a common problem in women that negatively affects quality of life. The estimated prevalence of symptomatic POP among women between 45-85 years of age is 8.3 - 11% [1,2]. It is current practice in the Netherlands that the general practitioner (GP) treats the majority of women with POP symptoms. Women with moderate to severe POP symptoms are often referred to a gynecologist for treatment. This study focuses at the subgroup of moderate to severe POP.

Known effective treatment options for moderate to severe POP are pessary or surgery. A pessary has proven its effectiveness in the treatment of symptomatic POP, mainly in cystocele and uterine descent. However, studies are mainly observational in nature and inherently subject to selection and indication bias [3]. In literature, outcomes of pessary therapy are mainly recorded in terms of (dis-) continuation of therapy and to a much lesser extent in terms of symptom relief. The pessary continuation rate is 60% [3]. This is confirmed by a Dutch pilot study in 65 women that showed a satisfaction with pessary in 57% of women and an operation rate of 43% at 12 months follow up [4]. In this study, 80% of women who continued pessary therapy reported much to very much improvement of their POP symptoms at 1 year follow up [4]. Reasons of discontinuation are pressure ulcer, vaginal discharge, discomfort or loss of fitting. These complications are reported to occur in up to 53% of women [5]. Half of them will decide to stop using pessary, but it is unclear which characteristics predict this outcome. Check-up of pessary therapy can be performed by either a general practitioner (GP), gynecologist or by self-management. According to a recent survey 50% percent of gynecologists involved in urogynaecology always offer self-management 40% on indication, and 10% never. Pessary therapy is inexpensive and costs are mainly related to doctor visits and treatment of side effects. In case of self-management costs might even be lower,

Surgery for POP results in much to very much improvement of symptoms in 80% of women and improvement of quality of life [6-9]. An anterior colporraphia is considered the standard procedure for a cystocele, as is the posterior colporraphia for a rectocele. For uterine descent uterus sparing techniques, like sacrospinous hysteropexy (SH) and modified Manchester-Fothergill procedure, or vaginal hysterectomy can be performed [10-12]]. Complications of POP surgery are temporary urinary retention, temporary buttock pain in case of sacrospinous hysteropexy, urinary tract infection, hematoma or dyspareunia [11]. These complications seldom lead to persistent morbidity. The most

For peer review only - http://bmjopen.bmj.com/site/about/guidelines.xhtml

common “complication” is the recurrence of symptomatic POP or de novo stress-incontinence that may lead to additional surgery, pessary therapy, or pelvic floor physiotherapy. As part of a RCT, comparing mesh with fascia plication, we found that
11% of women needed additional surgery after anterior colporraphia at 24 months follow up [7,9]. As in pessary therapy, the characteristics that predict successful or unsuccessful surgical therapy are largely unknown.

The decision which treatment option to choose depends on both patient and doctor’s preferences. In our pilot survey 70% of gynecologists informed their patients about the possibility of pessary therapy, but it is unknown how many women actually received a pessary. A recent Dutch study showed that 48% of treatment-naive women preferred surgery, 36% a pessary and 16% had no preference [28]. It is therefore reasonable to assume that at least 50% of treatment naïve women with moderate to severe prolapse symptoms will have surgery as primary treatment.

Although clinical efficacy appears to favor surgery [3], the large variation in study design, outcome measurements and loss to follow up makes any comment on the best treatment option speculative. This is recognized in two recent reviews on the subject that both urge the need for randomized trials comparing surgery and pessary for POP [13,14]. Efficacy can be expressed in terms of clinical outcome but also in terms of cost-effectiveness. It is obvious that surgery (especially hospital costs) is much more expensive than pessary therapy, but the cost-effectiveness of the surgical or pessary strategy has never been assessed. Based on current cohort and case-control studies we hypothesize that a strategy of initial pessary therapy for moderate to severe POP, is more cost-effective than surgery.
We propose to perform a randomized controlled trial to generate evidence for the optimal and most cost-effective primary treatment for moderate to severe POP, including a better a priori patient selection for treatment by identifying factors of failure for pessary therapy or surgery.

Relevance
At present a national multidisciplinary guideline on the diagnosis and treatment of POP is completed. The guideline identifies the lack of evidence with respect to the best treatment option for moderate to severe prolapse, a conclusion that is confirmed by the 2013 Cochrane Collaboration review [13]. In this evidence “vacuum” both doctors and patient
preferences rule, but unfortunately these are not supported by facts. If we look at the available data the following calculation emerges.

For peer review only - http://bmjopen.bmj.com/site/about/guidelines.xhtml

About 60% of women who start pessary therapy in the specialist care setting will continue using it at one year [4,15]. Eighty percent of them will report much to very much improvement, resulting in an overall 48% much to very much improvement. The majority of the 40% of women who are not satisfied with pessary therapy will request or are offered additional surgery. After surgery 80% of women report much to very much improvement of POP symptoms [6]. Combining these percentages, women who originally started with pessary therapy may also expect 80% (48% after initial pessary treatment + 32% after additional surgery =) much to very much improvement. Based on these estimates it is expected that the outcome of both treatment strategies will eventually result in a global improvement of symptoms in 80% of women. With equal clinical outcomes of both strategies the costs needed to obtain these outcomes become crucial. With the exception of a cost calculation based on a Markov model, no direct cost-effectiveness studies on the use of pessary or surgery for POP have been performed [16]. The relevance of this project, with the high prevalence of POP worldwide, associated costs and insufficient evidence, is high. We have searched the www.clinicaltrials.gov database (3th March 2014) on similar studies (comparing pessary with surgery) but none were found.

However, if we were to prove that pessary therapy is more cost-effective then surgical treatment, this does not imply that a trial of pessary should always be undertaken. There is also insufficient evidence on which patient characteristics are associated with failure of pessary treatment or surgery (systematic review). The knowledge on how to predict which women will have a very low chance of success with pessary therapy can further improve effective treatment strategy management. This will contribute to treatment efficacy. This is not only very relevant for the hospital specialist care setting, but this knowledge can also be implemented in general practitioner practice units.

There is very limited evidence on the optimal management strategy for pessary cleaning, both in time interval as well as in who should perform the cleaning. Our study is unique and therefore relevant since self-management is advocated in the study setting. This will not only allow it to obtain data in a standardized way, but also involves the woman in her own management. This involvement is strongly advocated by two major gynecologic patient organizations ('Patienten Gynaecologie Nederland' and the 'Stichting Bekkenbodem Patienten'). These two organizations, as well as the Dutch urogynaecological consortium have identified this study to be highly relevant.

For peer review only - http://bmjopen.bmj.com/site/about/guidelines.xhtml

In line with the report "Medisch Specialistische zorg 20/20" we are heading towards integral health care in which the general physician and medical specialist will work more closely together, using the same treatment protocol for various illnesses. The information and conclusions of this trial will add level I scientific evidence to such an integral protocol and guideline for women with symptomatic POP. This will aid in a better patient selection that will need referral to the specialist. The data on patient's self-management of pessary treatment will supply information for patient instructions, which are relevant for information leaflets on the subject.

For peer review only

For peer review only - http://bmjopen.bmj.com/site/about/guidelines.xhtml

2. OBJECTIVES

The aim of this multicenter pragmatic cohort study with embedded randomized controlled non-inferiority trial comparing pessary therapy versus surgery is twofold:

1. To prospectively compare the effectiveness and cost-effectiveness of pessary therapy or surgery as primary treatment of moderate to severe symptomatic cystocele, uterine descent and/or rectocele in women at two year after initiation of treatment.
2. To develop a prediction model for failure of pessary use and surgery within the first 2 years.

For peer review only

For peer review only - http://bmjopen.bmj.com/site/about/guidelines.xhtml

3. STUDY DESIGN

Multicenter pragmatic cohort study with an embedded randomized controlled non-inferiority trial comparing pessary [CE 0086] therapy versus surgery including an economic evaluation. The follow up will be 24 months.

After a short (30 minutes) trial of pessary fitting before randomization into our protocol. This ensures that only women who fit both treatment options enter the randomization procedure. The trial is short and only aims at fitting, not symptom relief. For those women with an unsuccessful pessary fitting baseline characteristics will be recorded to allow analyses of this group.

See also appendix 1.

For peer review only

For peer review only - http://bmjopen.bmj.com/site/about/guidelines.xhtml

4. STUDY POPULATION

4.1 Population (base)

All women with a symptomatic POP will be included.

4.2 Inclusion criteria

In order to be eligible to participate in this study, a subject must meet all of the following criteria:

1. Women with a prolapse stage 2 or more.
2. Women with moderate to severe POP symptoms. Moderate to severe POP symptoms is defined as a prolapse domain score > 33 on the validated Dutch version of the Pelvic Floor Distress Inventory (PFDI-20) [8, 23, 24].
3. Women who have had a successful pessary fitting procedure: for the RCT.
4. Written informed consent.

4.3 Exclusion criteria

A potential subject who meets any of the following criteria will be excluded from participation in this study:

1. Prior urogynaecological (prolapse or incontinence) surgery
2. Probability of future childbearing
3. Insufficient knowledge of the Dutch language
4. Co-morbidity causing increased surgical risks at the discretion of the surgeon
5. Major psychiatric illness
6. Prior pessary use

4.4 Sample size calculation

With 198 women per group, we will have 80% power to reject the null hypothesis that pessary therapy is inferior to surgery, with a 1-sided alpha of 0.05, a non-inferiority margin of 10% and the proportion in the standard group is 80% (NQueryAdvisor). Accounting for 10% loss to follow-up we plan to randomize 436 patients.

The sample size calculation for prediction models is based on the number of failures of pessary or surgical therapy. For each potential predictor in the model we need 10-15 failures. Our pessary group sample size is 198 women. An estimated 40% (80 women) will cross over to surgery and can be regarded as failures. Our sample size is therefore sufficient

For peer review only - http://bmjopen.bmj.com/site/about/guidelines.xhtml

to develop the prediction model for failure of pessary therapy for 6 to 8 items. In the surgery group 20% of women will not be satisfied with the result of treatment. With the same sample size of 198 women, the 40 women who are dissatisfied allow us to study up to 4 potential predictive factors.

For peer review only

For peer review only - http://bmjopen.bmj.com/site/about/guidelines.xhtml

5. TREATMENT OF SUBJECTS

5.1 Investigational product/treatment

Pessary [CE 0086] therapy and surgery both are options for the treatment of a symptomatic POP. Ten large urogynecological units (university hospitals or teaching hospitals) that have worked together in previous consortium studies will participate in this multicenter trial. All participating gynaecologists have fitted at least 100 pessaries and performed more than 100 surgical POP procedures prior to the start of this study.

All pessaries are made of modern silicon material. All types of pessaries, both supportive and occlusive/space filling are allowed according to the judgment of the gynaecologist. A recent randomized trial comparing supportive (ring) and occlusive (Gelhorn) showed no differences [17]. After placing the pessary, all women will receive verbal and written instructions on the self-management of pessary therapy.

The first pessary follow up visit will always be performed by the gynaecologist. In case of self-management the frequency of cleaning is left to her personal judgment, but may not exceed 1 month. If self-management is not possible, women will be seen at 4 months intervals for pessary cleaning and vaginal inspection, preferable by their GP. In case of vaginal atrophy topical estrogens will be advised according to pharmaceutical guidelines. The diagnosis of atrophy is left to the judgment of the treating physician, since no clear definition for atrophy is available yet [18].

All surgical procedures will be performed according to our national guidelines. In this pragmatic trial the decision which technique to use is left, to the discretion of the gynaecologist, within the limitations below [19]. Cystocele repair will consist of conventional anterior colporrhaphy [9]. For uterine descent different techniques are allowed [20]. These techniques can either be uterus sparing (sacrospinous hysteropexy [10], modified Manchester-Fothergill procedure [12] or a abdominal sacrocolpopexy [9]) or a vaginal hysterectomy. Recent studies showed similar effectiveness on both anatomical and functional outcomes for these different techniques [10, 12, 21]. A coexistent stage 2 rectocele repair will be a conventional colporrhaphia posterior. All procedures are performed under general or spinal anesthesia and under antibiotics and thrombosis prophylaxis according to local protocols.

5.2 Use of co-intervention (if applicable)

Not applicable.

For peer review only - http://bmjopen.bmj.com/site/about/guidelines.xhtml

5.3 Escape medication (if applicable)

Not applicable.

For peer review only

For peer review only - http://bmjopen.bmj.com/site/about/guidelines.xhtml

6. INVESTIGATIONAL PRODUCT

6.1 Name and description of investigational product(s)

6.2 Summary of findings from non-clinical studies

Not applicable.

6.3 Summary of findings from clinical studies

Three systematic reviews of the literature were performed by four members of our Dutch urogynaecology consortium (details in appendix 2-4) that concluded:

1. Systematic review on the (cost)effectiveness of pessary use compared to surgery

There are a very limited number of comparative studies on the efficacy of surgery or pessary use for POP. The differences in study population, inclusion criteria, follow-up period, large numbers of loss to follow-up, different outcome measures makes interpretation difficult if not impossible. The two studies that presented data on functional outcome in terms of prolapsed symptom reduction were favorable for surgery (appendix 2).

2. Systematic review of factors influencing pessary fitting and continuation

A systematic review was performed to identify the satisfactory pessary fitting rate and the continuation rate of pessary use. The factors influencing these rates as well as the cross over to prolapse surgery were identified from previous studies (appendix 3).

Summarizing the results show that an estimated 75% of women will have a successful fitting and 59% will continue pessary use at variable follow-up between 3 months and 5 years. In these 18 studies, 8 factors have been tested more than 4 times as prognostic factor of successful pessary use: Stress urinary incontinence was found associated with discontinuation of pessary in 5 out of 7 studies. In 7 out of 10 studies previous prolapse surgery or hysterectomy was associated with less continuation of pessary use. Higher age was related to continuation of pessary use in 3 out of 6 studies, whereas no correlation was found in the other studies. In 1 out of 4 studies sexual activity was related to longer pessary use, whereas in 1 out of 4 related to the choice for surgery. In the two other studies no correlation was found. In one study where the prolapse in a specific vaginal compartment was related to outcome, nor cystocele was related to longer pessary use.

Parity en menopausal status and hormonal replacement were mostly not related to continued pessary use.

For peer review only - http://bmjopen.bmj.com/site/about/guidelines.xhtml

3. Review of factors influencing failure of POP surgery.

A systematic review of factors influencing failure of POP surgery was performed concerning recurrence after surgery (surgery failure). There were 1 case control study, 3 prospective studies and 6 retrospective studies. There were 2298 women included in the studies. Forty-four (44) potential risk factors have been studied, of which 12 risk factors have at least once been identified as statistically significant risk factors in a multivariate logistic regression analysis (appendix 4).

6.4 Summary of known and potential risks and benefits

The present study carries low risks for the participant. Pessary [CE 0086] or surgery is standard care for symptomatic pelvic organ prolapse. Known risks for surgery are blood loss, risk of infection, dyspareunia, urine incontinence or a recurrence of a symptomatic pelvic organ prolapse.

The benefit of the study lies in a better understanding of satisfaction and cost effectiveness.

6.5 Description and justification of route of administration and dosage

Not applicable

6.6 Dosages, dosage modifications and method of administration

Not applicable

6.7 Preparation and labelling of Investigational Medicinal Product

Not applicable

6.8 Drug accountability

Not applicable

For peer review only - http://bmjopen.bmj.com/site/about/guidelines.xhtml

7. NON-INVESTIGATIONAL PRODUCT

Not applicable.

7.1 Name and description of non-investigational product(s)

Not applicable.

7.2 Summary of findings from non-clinical studies

Not applicable.

7.3 Summary of findings from clinical studies

Not applicable.

7.4 Summary of known and potential risks and benefits

Not applicable.

7.5 Description and justification of route of administration and dosage

Not applicable.

7.6 Dosages, dosage modifications and method of administration

Not applicable.

7.7 Preparation and labelling of Non Investigational Medicinal Product

Not applicable.

7.8 Drug accountability

Not applicable.

For peer review only

For peer review only - http://bmjopen.bmj.com/site/about/guidelines.xhtml

8. METHODS

8.1 Study parameters/endpoints

8.1.1 Main study parameter/endpoint

The primary outcome of this study is the percentage of women with much or very much improvement of POP symptoms at 2 years follow-up, as measured with the Patient Global Impression of Improvement (PGI-I)scale [22].
PGI-I is a 7-point Likert scale, with scores ranging from very much worse to very much improved. Success is defined as 'much or very
much' improvement.

8.1.2 Secondary study parameters/endpoints (if applicable)

1. Changes in symptom bother and quality of life at 12 and 24 months follow up.
2. Changes in sexual function at 12 and 24 months follow up.
3. Changes in general quality of life at 3, 6, 12 and 24 months.
4. Adverse events/complications related to both treatment strategies.
5. Development of prediction model to identify fail factors for pessary and surgery
6. Cost-effectiveness

8.1.3 Other study parameters (if applicable)

Baseline characteristics: Age; ethnicity; alcohol; smoking; number and mode of deliveries; menopausal status; hormone use; drug use; height; weight; co-morbidity (hypertension, diabetes mellitus, COPD, neurological disease, depression, cardiovascular disease); history of gynaecological operations; family history of prolapse; allergies, incontinence and sexual activity.
Physical examination: time, POP-Q, atrophy, stress test, blood loss, excessive discharge.

8.2 Randomisation, blinding and treatment allocation

After written informed consent is obtained, and inclusion and exclusion criteria are assessed, women will be randomly allocated in a 1:1 ratio to either treatment with a pessary or surgical treatment. Randomization will be done web based using ALEA, the software for randomization in clinical trials currently used by most studies in the Dutch consortium for studies on women's health and reproduction studies. The

For peer review only - http://bmjopen.bmj.com/site/about/guidelines.xhtml

randomization sequence will be computer generated using variable blocks of two and four, stratified for centre.

After entering the woman's initials and confirming inclusion criteria on the website, a unique number for randomization will be generated and the allocation code will be disclosed. This unique number cannot be deleted afterwards. This study will be open label because the nature of the intervention meant that masking to the intervention was not possible.

Women who attend the cohort will also be registered in ALEA.

8.3 Study procedures (see also appendix 5)

This study will be performed within the Dutch Urogynaecology Research Consortium, a subdivision of the Dutch Consortium for studies on women's health. Infrastructure (research nurses for counseling and data-monitoring, the use of web-based data entry), expertise on methodology and cost-effectiveness is shared.

1. Symptom bother and disease-specific quality of life are measured with the Pelvic Floor Distress Inventory (PFDI-20) and Pelvic Floor Impact Questionnaire (PFIQ) [23,24]. The validated version of the Dutch PFDI consists of five domains: pelvic organ prolapse, urinary incontinence, overactive bladder, pain, and obstructive micturition. The PFIQ measures the impact of urogenital symptoms on quality of life and consist of five domains: physical functioning, mobility, emotional health, embarrassment and social functioning.

2. Sexual function is measured with the PISQ-R. It is an international disease-specific questionnaire that measures sexual functioning in sexually active and inactive participants [26]. At this time, the Dutch translation is in progress, which will be finished in 2014.

3. Generic quality of life is measured with the EQ-5D and a questionnaire "doktersbezoek".

4. The adverse events of surgery recorded will consist of; direct peri-and postoperative complications (bleeding, pain and infection); interventions for complications; recurrent prolapse; de novo stress urinary incontinence. The adverse events of pessary recorded will consist of; discharge; pain; discomfort; bleeding; involuntary loss of pessary; de novo stress urinary incontinence.

5. The development of a prediction model is separately described in paragraph "data analyses".

6. The economic evaluation is described below.

For peer review only - http://bmjopen.bmj.com/site/about/guidelines.xhtml

ANTICIPATED COST-EFFECTIVENESS/BUDGET IMPACT

Cost differences between the two strategies are mainly the result from differences in costs associated with the initial intervention. Cost of a POP procedure is estimated at 4000 euros direct medical costs, and 4000 euros associated with lost productivity (indirect costs) if a societal perspective is used. The direct cost of pessary use is estimated at 200 euros, including costs for the pessary itself (50 euros) and consultations in the first year (150 euros). The estimated cost differences between the two strategies depend on the extent that women are (and remain) satisfied with the initial procedure (surgical or pessary): in case of dissatisfaction with the procedure, additional costs are generated by a subsequent intervention ((re-)operation, pessary, or pelvic physiotherapy). The flowchart (see appendix 1) illustrates the expected outcomes for each strategy. Based on the assumptions reflected in this flowchart, combined with approximate estimates for unit costs for POP surgery, pessary, GP and specialist visits, the anticipated impact on the annual health care budget as well as societal costs were estimated.

At present, the primary therapy for women presenting with moderate to severe POP is either surgery or pessary. The exact ratio is unknown, but is probably 50/50. If 50% women would receive primary surgery the current medical costs amount to 34 million Euros. If all women would start with pessary therapy, these costs would be 20 million euros, and the potential budget impact would be 14 million Euros/year. As it is not realistic that all women will start with pessary if this strategy proves to be successful, at 85% implementation of the pessary strategy, the annual budget impact will be around 10 million euros. The economic impact to society (including indirect (productivity) costs) will be 28 million euros and 20 million euros, at 100% and 85% implementation, respectively.

Sensitivity analyses showed, that these "base case" results are affected by estimated unit costs for POP surgery (direct and indirect costs) and the satisfaction rate for pessary, relative to surgery, but even the most conservative assumptions would lead to major cost savings for the health care budget (5 million euros) and society (15 million euros).

ECONOMIC EVALUATION

Considering the non-inferiority design of the study, we will not be able to rule out a small but acceptable difference in favor of POP surgery. Consequently, the economic

For peer review only - http://bmjopen.bmj.com/site/about/guidelines.xhtml

evaluation will be setup as a cost-effectiveness analysis, where cost-effectiveness will be expressed as costs per improvement outcome (much or very much improvement on the Patient Global Impression of Improvement (PGI-I)), and the incremental cost-effectiveness ratio as costs saved per additional case of unsatisfactory outcome. We will also perform a cost-effectiveness analysis using QALYs as health outcome, to express the difference between the two strategies in terms of costs (saved) per QALY (lost).

The economic evaluation will therefore encompass a cost-effectiveness analysis (CEA), a cost-utility analysis (CUA) as well as a budget impact analysis (BIA) from a health care budget and a societal perspective, with a time horizon between randomization and 2 years follow up. The primary outcome in the cost-effectiveness analysis will be costs per satisfactory outcome (primary clinical outcome), and the incremental cost-effectiveness ratio will reflect the costs saved per additional case of unsatisfactory outcome. As we hypothesize that pessary as a primary strategy in these patients does not result in more unsatisfactory outcomes, increased use of pessary will result in a decrease in the number of POP surgeries, and associated costs of hospital stay, recovery and (from a societal perspective) productivity loss (non-inferior strategy at lower costs).

Based on data actually observed in the trial, total costs associated with both surgery and pessary as a primary strategy will be estimated. Total costs can be divided into direct medical costs, non-medical costs and indirect costs. Direct medical costs are generated by utilization of primary or secondary health care services (including POP surgery, hospital stay, diagnostic procedures, medication). Non-medical costs are generated by travel expenses, and informal care; and indirect costs result from lost productivity due to absence from work or lost opportunity for non-paid activities. Non-medical and indirect costs are only included in the analysis from a societal perspective.

Resource utilization will be documented in the clinical report form (CRF) and complementary patient questionnaires, based on the Medical Consumption Questionnaire (MCQ) and Productivity Costs Questionnaire (PCQ) [29,30]. In patients for whom complete follow-up is not available, cost and quality-of-life data will be extrapolated using multiple imputations. Unit costs will be based on Dutch guideline

For peer review only - http://bmjopen.bmj.com/site/about/guidelines.xhtml

prices (for primary and secondary health services, informal care and lost productivity), and market prices (for medication)[31,32]

Similarly, the incremental costs per QALY gained will be estimated over a period of two years. Health state utilities to estimate QALYs will be derived from an EQ-5D measurement at discharge, as well as at follow-up assessments. Utility values for EQ-5D scores will be based on UK-estimates (Dolan, 1997). Utility scores will be linearly interpolated, assuming constant increase/decrease between subsequent assessments.

Robustness for sampling uncertainty as well as uncertainty associated with cost estimates and assumptions will be assessed in sensitivity analyses, including: Dutch health states (Lamers, 2005) instead of the UK based model in the main analyses; and varying unit costs for pertinent volumes of health care utilization (e.g. costs of POP surgery, pessary use, productivity costs).
The incremental costs and effects will be depicted in a cost effectiveness plane and cost-effectiveness acceptability curves providing information directly interpretable as the probability of one intervention being cost-effective compared to the alternative given a ceiling ratio that policy makers are willing to invest.

BIA

In a budget impact analysis, study results will be extrapolated to the national level to estimate the total impact on the health care budget per annum for the Netherlands in terms of cost reduction and health outcomes (satisfactory outcomes as well as QALYs). As economic consequences of the intervention are expected to span multiple years, this accumulation of cost (savings) will be reflected in the budget impact analyses.

The Budget Impact Analysis will be executed according to the international ISPOR guidelines [33]. This framework for creating a budget impact model includes formalized guidance about the acquisition and use of data in order to make budget projections. In addition to the societal perspective, the BIA will therefore be also report economic consequences from the perspective of the Dutch budgetary health care framework (BKZ). If the probability of an unsatisfactory outcome exceeds the non-inferiority limit, recommending pessary as primary treatment for all women is not

For peer review only - http://bmjopen.bmj.com/site/about/guidelines.xhtml

feasible, and an economic evaluation/budget impact analysis is not sensible. To estimate costs, we will follow the Handleiding Kosten onderzoek CVZ 2010.

8.4 Withdrawal of individual subjects

Subjects can leave the study at any time for any reason if they wish to do so without any consequences. The investigator can decide to withdraw a subject from the study for urgent medical reasons.

8.4.1 Specific criteria for withdrawal (if applicable)

Not applicable.

8.5 Replacement of individual subjects after withdrawal

We will not replace patients who withdrew informed consent. We will replace patients that are randomized by mistake, for example because of technical errors with online randomization.

8.6 Follow-up of subjects withdrawn from treatment

Patients withdrawn from the intervention but not from informed consent will be followed up.

8.7 Premature termination of the study

This study includes standard care, therefore it is very unlikely that unexpected complications will occur. Therefore premature termination is not applicable.

For peer review only

9. SAFETY REPORTING

9.1 Section 10 WMO event

In accordance to section 10, subsection 1, of the WMO, the investigator will inform the subjects and the reviewing accredited METC if anything occurs, on the basis of which it appears that the disadvantages of participation may be significantly greater than was foreseen in the research proposal. The study will be suspended pending further review by the accredited METC, except insofar as suspension would jeopardise the subjects' health. The investigator will take care that all subjects are kept informed.

9.2 AEs, SAEs and SUSARs

9.2.1 Adverse events (AEs)

Adverse events are defined as any undesirable experience occurring to a subject during the study, whether or not considered related to the study. All adverse events reported spontaneously by the subject or observed by the investigator or his staff will be recorded. During visits complaints will be questioned systematically.

9.2.2 Serious adverse events (SAEs)

A serious adverse event is any untoward medical occurrence or effect that at any dose:

- results in death;
- is life threatening (at the time of the event);
- requires hospitalisation or prolongation of existing inpatients' hospitalisation (>4 days);
- results in persistent or significant disability or incapacity;
- is a congenital anomaly or birth defect;
- Any other important medical event that may not result in death, be life threatening, or require hospitalization, may be considered a serious adverse experience when, based upon appropriate medical judgement, the event may jeopardize the subject or may require an intervention to prevent one of the outcomes listed above.

SAEs have to be reported when its occurrence appears in two days after the study operations. The investigators in participating centres should inform the coordinating investigator as soon as possible but at least the next working day.

For peer review only - http://bmjopen.bmj.com/site/about/guidelines.xhtml

The sponsor will report the SAEs through the web portal *ToetsingOnline* to the accredited METC that approved the protocol, within 15 days after the sponsor has first knowledge of the serious adverse events.

SAEs that result in death or are life threatening should be reported expedited. The expedited reporting will occur not later than 7 days after the responsible investigator has first knowledge of the adverse event. This is for a preliminary report with another 8 days for completion of the report.

9.2.3 Suspected unexpected serious adverse reactions (SUSARs)

Not applicable.

9.3 Annual safety report

The annual safety report will be combined with the annual progress report (see chapter 12.4).

9.4 Follow-up of adverse events

All AEs will be followed until they have abated, or until a stable situation has been reached. Depending on the event, follow up may require additional tests or medical procedures as indicated, and/or referral to the general physician or a medical specialist.
SAEs need to be reported till end of study within the Netherlands, as defined in the protocol

9.5 Data Safety Monitoring Board (DSMB)

Since both techniques are standard practice, and no major unexpected complications are foreseen, no interim analysis is planned. A Data Safety and Monitoring Board will not be installed, as both procedures are regularly used and acceptable options in current clinical practice.

For peer review only

10. STATISTICAL ANALYSIS

The results of the study will be reported according to the CONSORT statement.

The main outcomes will be analysed and presented according the intention-to-treat principle. Since in this pragmatic trial we expect that in the pessary strategy group 40% of women will cross over to surgery an additional per protocol analyses is foreseen. This will provide more insight in the effect of surgery after pessary therapy as compared to surgery or pessary therapy alone.

We plan a subgroup analysis for the location of the prolapse: anterior prolapse versus posterior prolapse.

10.1 Primary study parameter(s)

The primary outcome, success (much or very much improvement) or no success (a little better, no change, a little worse, much worse or very much worse) on the PGI-I will be expressed in percentage point differences. Differences between the percentages will be tested using a chi-square test. A p-value <0.05 will be considered statistically significant.

10.2 Secondary study parameter(s)

The PFDI, PFIQ and PISQ-r are all interval scales. Differences between baseline and 12 and 24 months follow up will be assessed using an independent t-test when normality can be assumed, or by non-parametric tests when the data are not normally distributed. Effect sizes will be calculated to estimate the magnitude of changes.

Differences in EQ5-D scores and "ziekteverzuim" between baseline and at 3, 6, 12 and 24 months will be assessed using t-test and further incorporated in the cost-effectiveness analyses.

Imputation statistics will be used or missing data.

(Serious) adverse events will be categorized and chi-square statistics, with calculation of relative risks when appropriate, will be applied in analyses.

Prediction model

A prediction model that uses predefined variables, as potential predictors of failure of pessary therapy, will be developed using multivariable regression analysis. Missing data will be imputed.

For peer review only - http://bmjopen.bmj.com/site/about/guidelines.xhtml

Predictors for failure derived from literature are a large genital hiatus (gh > 4 cm), women being sexually active, age > 65 years, prolapse POP-Q stage 3, previous hysterectomy. If applicable, a prediction rule for the chance of failure of pessary therapy will be constructed, which could be presented as a normogram which could be used to determine the chance of failure on pessary therapy.

Internal validity will be assessed using bootstrapping techniques; shrinkage will be applied to the parameter estimates. Model performance will be assessed with discriminative capacity and calibration. Calibration will be assessed by comparing the mean predicted probability that patients failed on pessary therapy with the mean observed probability that patients failed on pessary therapy. To do so, the total cohort will be split into ten groups based on the deciles of the predicted probability. Per group the mean predicted probability will be calculated as well as the mean observed predicted probability. Discriminative capacity of the model will be assessed with receiver operation characteristics (ROC) analysis and the area under the ROC curve (AUC).

We will also look at factors that could explain failure of surgery. Our systematic review on POP and recurrent POP after surgery showed that 6 preoperative items, eg. POP stage, age, family history, preoperative incontinence, previous POP or incontinence surgery, previous hysterectomy seems to be predictive for recurrence.

Women with previous POP, incontinence surgery or previous hysterectomy are excluded from our study, leaving 4 predefined potential predictive factors. After the 2 year follow-up has been performed, we will reconsider which factors to include in a prognostic model, based on the current literature. We will select predictors from literature with the highest predictive value, where about 1 predictor could be selected for each 10 surgery failures.

Using interaction terms the effect of a differential effect in women with a higher age (>median) or a lower age (<=median), a higher (>25) or lower BMI(<=25) will be assessed for both pessary as well as surgery failures prediction.

10.3 Other study parameters

Not applicable.

For peer review only - http://bmjopen.bmj.com/site/about/guidelines.xhtml

10.4 Interim analysis (if applicable)

Not applicable, because of the non-inferiority design with low risk and the possibility of cross over.

For peer review only

For peer review only - http://bmjopen.bmj.com/site/about/guidelines.xhtml

11. ETHICAL CONSIDERATIONS

11.1 Regulation statement

This study will be conducted according to the principles of the Declaration of Helsinki (version 10, October 2013) and in accordance with the Medical Research Involving Human Subjects Act (WMO) and other guidelines, regulations and Acts.

11.2 Recruitment and consent

Women with symptomatic pelvic organ prolapse who attend the outpatient clinic will be informed about the study by the gynaecologist or nurse. After checking the in- and exclusion criteria the women will receive verbal and written information about the study. If the woman is willing to participate she is asked to sign the informed consent. All women will undergo the pessary fitting test which is part of the standard evaluation during the first visit. All women will be contacted at a minimum interval of 1 week. Those women who failed the initial fitting will be offered surgery and attend the cohort, the women with a succesfull initial fitting will be asked to enroll in the RCT. In case the woman is willing to participate but actively opts for pessary therapy she will be provided with a pessary and enter the cohort.

11.3 Objection by minors or incapacitated subjects (if applicable)

Not applicable.

11.4 Benefits and risks assessment, group relatedness

The present study carries no risks for the participant. Pessary or surgery are standard care for symptomatic pelvic organ prolapse. The benefit of the study lies in a better understanding of satisfaction and cost effectiveness.

11.5 Compensation for injury

The sponsor/investigator has a liability insurance which is in accordance with article 7, subsection 9 of the WMO.

The sponsor (also) has an insurance which is in accordance with the legal requirements in the Netherlands (Article 7 WMO and the Measure regarding Compulsory Insurance for Clinical Research in Humans of 23th June 2003). This insurance provides cover for damage to research subjects through injury or death caused by the study.

For peer review only - http://bmjopen.bmj.com/site/about/guidelines.xhtml

1. € 450.000,-- (i.e. four hundred and fifty thousand Euro) for death or injury for each subject who participates in the Research;
2. € 3.500.000,-- (i.e. three million five hundred thousand Euro) for death or injury for all subjects who participate in the Research;
3. € 5.000.000,-- (i.e. five million Euro) for the total damage incurred by the organisation for all damage disclosed by scientific research for the Sponsor as 'verrichter' in the meaning of said Act in each year of insurance coverage.

The insurance applies to the damage that becomes apparent during the study or within 4 years after the end of the study.

11.6 Incentives (if applicable)

Not applicable.

For peer review only

12. ADMINISTRATIVE ASPECTS, MONITORING AND PUBLICATION

12.1 Handling and storage of data and documents

All data will be stored and will be coded. Only the researchers will be able to link patient ID and research code. The handling of personal data complies with the Dutch Personal Data Protection Act. After cessation of the study, patient material will be stored for a maximum of 15 years.

The case report forms and questionnaires will be filled in online. The head investigator will be able to check all the completed forms and questionnaires.

12.2 Monitoring and Quality Assurance

The monitoring will be coordinated by the Staff Member Clinical Research, quality coordinator of division women and baby en will be executed by a qualified intern monitor. This person is not involved in design and output of this research. The frequency of checking will be every year. The monitoring plan is discussed in section K of the METC dossier.

12.3 Amendments

All substantial amendments will be notified to the METC.

Non-substantial amendments will not be notified to the accredited METC, but will be recorded and filed by the sponsor.

12.4 Annual progress report

The sponsor/investigator will submit a summary of the progress of the trial to the accredited METC once a year. Information will be provided on the date of inclusion of the first subject, numbers of subjects included and numbers of subjects that have completed the trial, serious adverse events/ serious adverse reactions, other problems, and amendments.

12.5 End of study report

The investigator will notify the accredited METC of the end of the study within a period of 8 weeks. The end of the study is defined as the last patient's last visit.

In case the study is ended prematurely, the investigator will notify the accredited METC within 15 days, including the reasons for the premature termination.

For peer review only - http://bmjopen.bmj.com/site/about/guidelines.xhtml

Within one year after the end of the study, the investigator/sponsor will submit a final study report with the results of the study, including any publications/abstracts of the study, to the accredited METC.

12.6 Public disclosure and publication policy

The research findings will be published in peer reviewed journals.

For peer review only

For peer review only - http://bmjopen.bmj.com/site/about/guidelines.xhtml

13. STRUCTURED RISK ANALYSIS

Not applicable because this study is a low risk study concerning standard care.

13.1 Potential issues of concern

Not applicable.

13.2 Synthesis

Not applicable.

For peer review only

14. REFERENCES

1: Slieker-ten Hove MC et al. The prevalence of pelvic organ prolapse symptoms and signs and their relation with bladder and bowel disorders in a general female population. Int Urogynecol J. 2009;20(9):1037-45
2. Tegerstedt G, Maehle-Schmidt M, Nyrén O, Hammarström M. Prevalence of symptomatic pelvic organ prolapse in a Swedish population. Int Urogynecol J Pelvic Floor Dysfunct. 2005 Nov-Dec;16(6):497-503
3: Lamers BH et al. Pessary treatment for pelvic organ prolapse and health-related quality of life: a review. Int Urogynecol J. 2011;22(6):637-44
4: Broekman BM et al. Prospective pilot study in 65 women on pessary treatment as primary treatment, unpublished data
5:Broens-Oostveen WC, Mom RM, Lagro-Janssen ALM. De genitale prolaps: behnadleing en beloop in vier huisartspraktijken. Ned Tijdschr Geneeskd 2004, 148 (29); 1444-1448
6: Withagen M et al. Sacrospinous hysteropexy or modified manchester for the treatment of pelvic organ prolapse? Int J Gynaecol Obstet. 2013; Abstract Supplement
7: Vollebregt A et al. Cost-utility analysis of trocar-guided transobturator mesh versus anterior colporrhaphy for primary cystocele repair: a randomised trial. [in preparation]
8: Barber MD[1], Walters MD, Bump RC. Short forms of two condition-specific quality-of- life questionnaires for women with pelvic floor disorders (PFDI-20 and PFIQ-7). Am J Obstet Gynecol. 2005 Jul;193(1):103-13.
9: Pollard ME, Eilber KS, Anger JT. Abdominal approaches to pelvic prolapse repairs. Curr Opin Urol. 2013;23(4):306-11.
10: Dietz V et al. Vaginal surgery for uterine descent; which options do we have? A review of the literature. Int J Gynaecol Obstet. 2009;20(3):349-56
11: C. Maher, Feiner B, Baessler K, Schmid C. Surgical management of pelvic organ prolapse in women. Cochrane 2013
12: de Boer TA, Milani AL, Kluivers KB, Withagen MI, Vierhout ME. The effectiveness of surgical correction of uterine prolapse: cervical amputation with uterosacral ligament plication (modified Manchester) versus vaginal hysterectomy with high uterosacral ligament plication. Int Urogynecol J Pelvic Floor Dysfunct. 2009 Nov;20(11):1313-9
13: Bugge C et al. Pessaries (mechanical devices) for pelvic organ prolapse in women. Cochrane database syst rev, 2013;28;2:CD004010
14: van Geelen JM, Dwyer PL. Where to for pelvic organ prolapse treatment after the FDA pronouncements? A systematic review of the recent literature. Int Urogynecol J. 2013 May;24(5):707-18
15: Abdool Z et al. Prospective evaluation of outcome of vaginal pessaries versus surgery in women with symptomatic pelvic organ prolapse. Int Urogynecol J. 2011;22(3):273-8
16: Hullfish KL, Trowbridge ER, Stukenborg GJ. Treatment strategies for pelvic organ prolapse: a cost-effectiveness analysis. Int Urogynecol J. 2011 May;22(5):507-15
17: Cundiff GW, Amundsen CL, Bent AE, Coates KW, Schaffer JI, Strohbehn K, Handa VL. The PESSRI study: symptom relief outcomes of a randomized crossover trial of the ring and Gellhorn pessaries. Am J Obstet Gynecol. 2007 Apr;196(4):405.e1-8
18: Weber MA, Limpens J, Roovers JPWR. Assessment of vaginal atrophy: a review [submitted BJOG]
19: Cobra Ok-Klapper 2012. DCHG 2012
20: Detollenaere RJ, den Boon J, Kluivers KB, Vierhout ME, van Eijndhoven HW.Surgical management of pelvic organ prolapse and uterine descent in the Netherlands. Int Urogynecol J. 2013 May;24(5):781-8
21.Detollenaere RJ, den Boon J, Vierhout ME, van Eijndhoven HW. Uterus preserving surgery versus vaginal hysterectomy in treatment of uterine descent: a systematic review]. Ned Tijdschr Geneeskd. 2011;155(42):A3623

For peer review only - http://bmjopen.bmj.com/site/about/guidelines.xhtml

22: Yalcin I, Bump RC. Validation of two global impression questionnaires for incontinence. Am J Obstet Gynecol. 2003 Jul;189(1):98-101
23: Barber MD, Kuchibhatla MN, Pieper CF, Bump RC. Psychometric evaluation of 2 comprehensive condition-specific quality of life instruments for women with pelvic floor disorders. Am J Obstet Gynecol 2001;185(6):1388–1395.
24: Utomo E, Blok BF, Steensma AB, Korfage IJ. Validation of the Pelvic Floor Distress Inventory (PFDI-20) and Pelvic Floor Impact Questionnaire (PFIQ-7) in a Dutch population. Int Urogynecol J. 2014;25(4):531-44.
25: Euroquol—a new facility for the measurement of health-related quality of life. The EuroQol Group. Health Policy. 1990;16:199-208
26: Rogers RG, Rockwood TH, Constantine ML, Thakar R, Kammerer-Doak DN, Pauls RN, Parekh M, Ridgeway B, Jha S, Pitkin J, Reid F, Sutherland SE, Lukacz ES, Domoney C, Sand P, Davila GW, Espuna Pons ME. A new measure of sexual function in women with pelvic floor disorders (PFD): the Pelvic Organ Prolapse/Incontinence Sexual Questionnaire, IUGA-Revised (PISQ-IR). Int Urogynecol J. 2013 Jul;24(7):1091-103
27: Cabana MD, Rand CS, Powe NR, Wu AW, Wilson MH, Abboud PA, Rubin HR. Why don't physicians follow clinical practice guidelines? A framework for improvement. JAMA. 1999 Oct 20;282(15):1458-65
28: Thys SD, Roovers JP, Geomini PM, Bongers MY. Do patients prefer a pessary or surgery as primary treatment for pelvic organ prolapse. Gynecol Obstet Invest. 2012;74(1):6-12
29: www.imta.nl (C. Bouwmans et al. Handleiding iMTA Medical Cost Questionnaire (iMCQ). Rotterdam: iMTA, Erasmus Universiteit Rotterdam, 2013)
30: www.imta.nl (C. Bouwmans et al. Handleiding iMTA Productivity Cost Questionnaire (iPCQ). Rotterdam: iMTA, Erasmus Universiteit, 2013)
31:Hakkaart- van Roijen, S.S. Tan, C.A.M. Bouwmans: Handleiding voor Kostenonderzoek. Methoden en standaard kostprijzen voor economische evaluaties in de gezondheidszorg. CVZ 2010
32: Oostenbrink JB, Koopmanschap MA, Rutten FF. Standardisation of costs: the Dutch Manual for Costing in economic evaluations. Pharmacoeconomics 2002;20(7):443-454
33: Mauskopf JA, Sullivan SD, Annemans L et al. Principles of good practice for budget impact analysis: report of the ISPOR Task Force on good research practices--budget impact analysis. Value Health 2007;10(5):336-347 -> In BIA, klopt niet, nog vervangen door:

References (Cost) effectiveness of pessary use as compared to surgery systematic review

1: Mamik MM, Rogers RG, Qualls CR, Komesu YM. Goal attainment after treatment in patients with symptomatic pelvic organ prolapse. Am J Obstet Gynecol. 2013 Nov;209(5):488.e1-5.
2: Abdool Z, Thakar R, Sultan AH, Oliver RS. Prospective evaluation of outcome of vaginal pessaries versus surgery in women with symptomatic pelvic organ prolapse. Int Urogynecol J. 2011 Mar;22(3):273-8.
3: Lowenstein L, Gamble T, Sanses TV, van Raalte H, Carberry C, Jakus S, Pham T, Nguyen A, Hoskey K, Kenton K; Fellow's Pelvic Research Network. Changes in sexual function after treatment for prolapse are related to the improvement in body image perception. J Sex Med. 2010 Feb;7(2 Pt 2):1023-8.
4: Barber MD, Walters MD, Cundiff GW; PESSRI Trial Group. Responsiveness of the Pelvic Floor Distress Inventory (PFDI) and Pelvic Floor Impact Questionnaire (PFIQ) in women undergoing vaginal surgery and pessary treatment for pelvic organ prolapse. Am J Obstet Gynecol. 2006 May;194(5):1492-8.

References Systematic review of factors influencing pessary fitting and continuation

For peer review only - http://bmjopen.bmj.com/site/about/guidelines.xhtml

1. Abdool Z, Thakar R, Sultan AH, Oliver RS (2010) Prospective evaluation of outcome of vaginal pessaries versus surgery in women with symptomatic pelvic organ prolapse. Int Urogynecol J Pelvic Floor Dysfunct 16
2. Alperin M, Khan A, Dubina E, Tarnay C, Wu N, Pashos CL, Anger JT.Female Pelvic Med Reconstr Surg. 2013 May-Jun;19(3):142-7.Patterns of pessary care and outcomes for medicare beneficiaries with pelvic organ prolapse.
3. Bai SW, Yoon BS, Kwon JY, Shin JS, Kim SK, Park KH (2005) Survey of the characteristics and satisfaction degree of the patients using a pessary. Int Urogynecol J Pelvic Floor Dysfunct 16 (3):182–186
4. Barber MD, Walters MD, Cundiff GW (2006) Responsiveness of the Pelvic Floor Distress Inventory (PFDI) and Pelvic Floor Impact Questionnaire (PFIQ) in women undergoing vaginal surgery and pessary treatment for pelvic organ prolapse. Am JObstet Gynecol 194(5):1492–149
5. Brincat C, Kenton K, Pat FM, Brubaker L (2004) Sexual activity predicts continued pessary use. Am J Obstet Gynecol 191(1):198–200
6. Broens-Oostveen MC, Mom RM, Lagro-Janssen AL (2004)Genital prolapse; treatment and course in four general practices.Ned Tijdschr Geneeskd 148(29):1444–1448, 17
7. Chan SS, Cheung RY, Yiu KW, Lee LL, Pang AW, Chung TK. Int Urogynecol J. 2012 Aug;23(8):1027-33. Symptoms, quality of life, and factors affecting women's treatment decisions regarding pelvic organ prolapse.
8. Clemons JL, Aguilar VC, Sokol ER, Jackson ND, Myers DL (2004) Patient characteristics that are associated with continued pessary use versus surgery after 1 year. Am J Obstet Gynecol 191(1):159–164
9. Cundiff GW, Amundsen CL, Bent AE, Coates KW, Schaffer JI, Strohbehn K et al (2007) The PESSRI study: symptom relief outcomes of a randomized crossover trial of the ring and Gellhorn pessaries. Am J Obstet Gynecol 196(4):405–408
10. Fernando RJ, Thakar R, Sultan AH, Shah SM, Jones PW (2006) Effect of vaginal pessaries on symptoms associated with pelvic organ prolapse. Obstet Gynecol 108(1):93–99
11. Friedman S, Sandhu KS, Wang C, Mikhail MS, Banks E (2010) Factors influencing long-term pessary use. Int Urogynecol J Pelvic Floor Dysfunct 21(6):673–678
12. Hanson LA, Schulz JA, Flood CG, Cooley B, Tam F (2006) Vaginal pessaries in managing women with pelvic organ prolapse and urinary incontinence: patient characteristics and factors contributing to success. Int Urogynecol J Pelvic Floor Dysfunct 17(2):155–159
13. Jones K,Yang L, Lowder JL, Meyn L, Ellison R, ZyczynskiHMet al (2008) Effect of pessary use on genital hiatus measurements in women with pelvic organ prolapse. Obstet Gynecol 112(3):630–636
14. Geoffrion R, Zhang T, Lee T, Cundiff GW.Female Pelvic Med Reconstr Surg. 2013 Nov-Dec;19(6):339-45. Clinical characteristics associated with unsuccessful pessary fitting outcomes.
15. Komesu YM, Rogers RG, Rode MA, Craig EC, Gallegos KA, Montoya AR et al (2007) Pelvic floor symptom changes incpessary users. Am J Obstet Gynecol 197(6):620–626
16. Kuhn A, Bapst D, Stadlmayr W, Vits K, Mueller MD (2009) Sexual and organ function in patients with symptomatic prolapse: are pessaries helpful? Fertil Steril 91(5):1914–1918
17. Lone F1, Thakar R, Sultan AH, Karamalis G. A 5-year prospective study of vaginal pessary use for pelvic organ prolapse. Int J Gynaecol Obstet. 2011 Jul;114(1):56-9.
18. Maito JM, Quam ZA, Craig E, Danner KA, Rogers RG (2006) Predictors of successful pessary fitting and continued use in a nurse-midwifery pessary clinic. J Midwifery Womens Health 51 (2):78–84
19. Mamik MM, Rogers RG, Qualls CR, Komesu YM. Goal attainment after treatment in patients with symptomatic pelvic organ prolapse. Am J Obstet Gynecol. 2013 Nov;209(5):488.e1-5.
20. Manchana T, Bunyavejchevin S. Impact on quality of life after ring pessary use for pelvic organ prolapse. Int Urogynecol J. 2012 Jul;23(7):873-7.

For peer review only - http://bmjopen.bmj.com/site/about/guidelines.xhtml

21. Markle D, Skoczylas L, Goldsmith C, Noblett K. Patient characteristics associated with a successful pessary fitting. Female Pelvic Med Reconstr Surg. 2011 Sep;17(5):249-52.
22. Mokrzycki ML, Hatangadi SB, Zaccardi JE, Cox S. Preexisting stress urinary incontinence: a predictor of discontinuation with pessary management. J Low Genit Tract Dis. 2001 Oct;5(4):204-7.
23. Mutone MF, Terry C, Hale DS, Benson JT (2005) Factors which influence the short-term success of pessary management of pelvic organ prolapse. Am J Obstet Gynecol 193(1):89–94
24. Nguyen JN, Jones CR. Pessary treatment of pelvic relaxation: factors affecting successful fitting and continued use. J Wound Ostomy Continence Nurs. 2005;32(4):255-61; quiz 262-3.
25. Patel M, Mellen C, O'Sullivan DM, Lasala CA. (2010) Impact ofpessary use on prolapse symptoms, quality of life, and bodyimage. Am J Obstet Gynecol 18
26. Powers K, Lazarou G, Wang A, LaCombe J, Bensinger G.Greston WM et al (2006) Pessary use in advanced pelvic organprolapse. Int Urogynecol J Pelvic Floor Dysfunct 17(2):160–164.

Reference list Systematic review of factors influencing failure of POP surgery

1. Whiteside JL, Weber AM, Meyn LA, Walters MD. Am J Obstet Gynecol. 2004 Nov;191(5):1533-8.factors for prolapse recurrence after vaginal repair.
2. Diez-Itza I, Aizpitarte I, Becerro A. Int Urogynecol J Pelvic Floor Dysfunct. 2007 Nov;18(11):1317-24. Risk factors for the recurrence of pelvic organ prolapse after vaginal surgery: a review at 5 years after surgery.
3. Fialkow MF, Newton KM, Weiss NS. Int Urogynecol J Pelvic Floor Dysfunct. 2008 Nov;19(11):1483-7.Incidence of recurrent pelvic organ prolapse 10 years following primary surgical management: a retrospective cohort study.
4. Jeon MJ, Chung SM, Jung HJ, Kim SK, Bai SW. Gynecol Obstet Invest. 2008;66(4):268-73 Risk factors for the recurrence of pelvic organ prolapse.
5. Dällenbach P, Jungo Nancoz C, Eperon I, Dubuisson JB, Boulvain M. Int Urogynecol J. 2012 Jan;23(1):35-41. Incidence and risk factors for reoperation of surgically treated pelvic organ prolapse.
6. Weemhoff M, Vergeldt TF, Notten K, Serroyen J, Kampschoer PH, Roumen FJ. Int Urogynecol J. 2012 Jan;23(1):65-71. Avulsion of puborectalis muscle and other risk factors for cystocele recurrence: a 2-year follow-up study.
7. Tegerstedt G1, Hammarström M. Acta Obstet Gynecol Scand. 2004 Aug;83(8):758-63. Operation for pelvic organ prolapse: a follow-up study.
8. Medina CA, Candiotti K, Takacs P. Int J Gynaecol Obstet. 2008 May;101(2):184-7. Wide genital hiatus is a risk factor for recurrence following anterior vaginal repair.
9. Salvatore S1, Athanasiou S, Digesu GA, Soligo M, Sotiropoulou M, Serati M, Antsaklis A, Milani R. Neurourol Urodyn. 2009;28(4):301-4. doi: 10.1002/nau.20639. Identification of risk factors for genital prolapse recurrence.
10. Kawasaki A, Corey EG, Laskey RA, Weidner AC, Siddiqui NY, Wu JM. J Reprod Med. 2013 May-Jun;58(5-6):195-9. Obesity as a risk for the recurrence of anterior vaginal wall prolaps after anterior colporrhaphy.

For peer review only - http://bmjopen.bmj.com/site/about/guidelines.xhtml

Appendix 1:

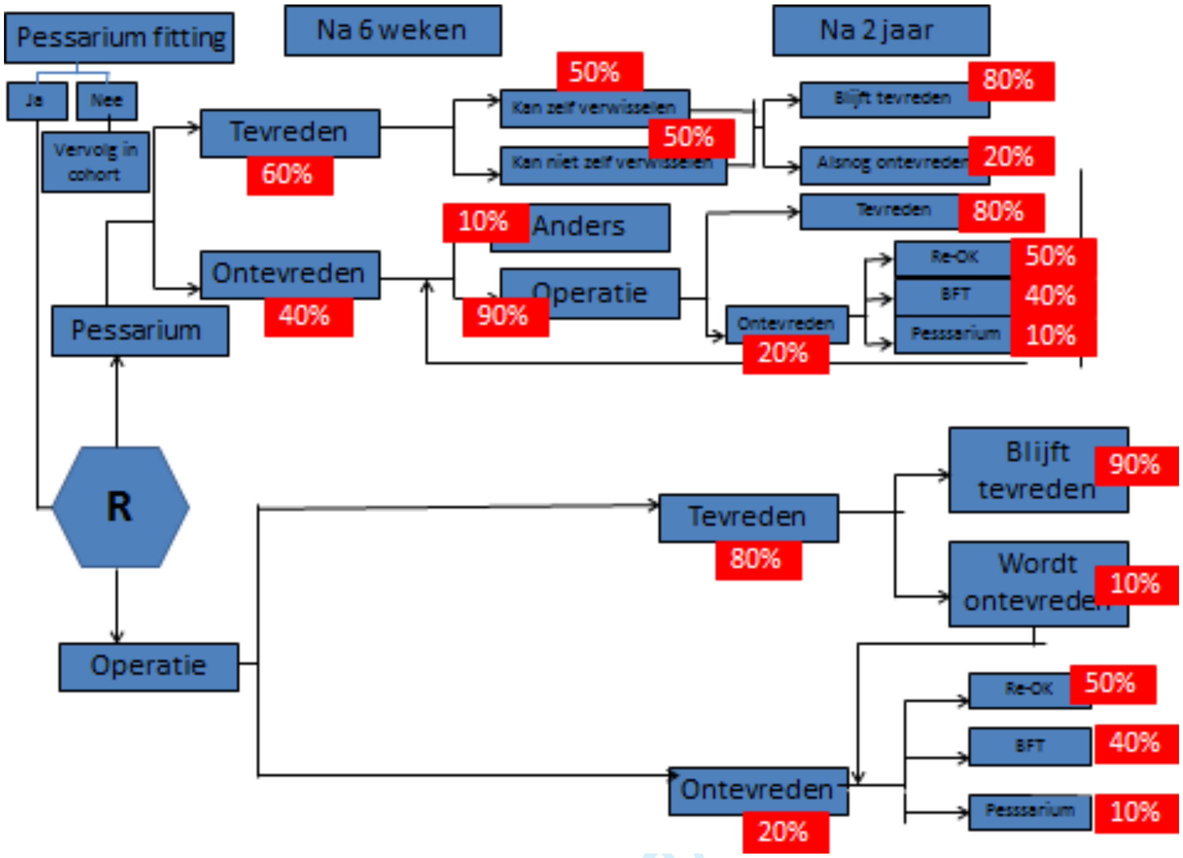

For peer review only - http://bmjopen.bmj.com/site/about/guidelines.xhtml

Appendix 2 Review on (Cost) effectiveness of pessary use as compared to surgery:

For peer review only

For peer review only - http://bmjopen.bmj.com/site/about/guidelines.xhtml

Reference	Study type	Characteristics	Intervention (I)	Controls (C)	Outcome measures and follow-up time	Results
**Mamik, 2012**<br><br>**AJOG 2013:209:488**	**Design**<br>**Case-control**<br><br>**N = 100**<br><br>**Country**<br>**US**	**Aim: compare goal achievement and global improvement between pessary and surgery for POP stage ≥2.**<br><br>**Inclusion criteria: >18 year old, read and write in English**<br><br>**Exclusion: not given**	**Vaginal pessary**<br>**N = 50**	**Prolapse surgery**<br>**N = 50**	**Primary outcome:**<br>**Goal attainment**<br><br>**Secondary:**<br>**PGI-I**<br>**PFDI-20**<br>**PISQ-12**<br>**Body Image scale**<br><br>**Follow-up:**<br>**3 months**	**Primary outcome:**<br>**Goal attainment sign. higher score after surgery (8.6 vs 6.4)**<br><br>**Secondary outcomes**<br>**PGI-I sign (p=0.04) better improvement after surgery (2.4 vs 1.9 points)**<br>**PFDI-20 sign (p=0.02) higher change (89 vs 43 points)**<br>**PISQ-12 and BIS no sign difference**<br><br>**Additional: 10% crossed over from pessary to surgery within 3 months and 10% referred from surgery after they had been selected as eligible.**<br>**No follow-up in pessary group is 40% (20/50) and surgery 30% (15/50)**
**Abdool, 2011**	**Design**<br>**Cohort study**<br><br>**N total = 554**<br><br>**Country:**<br>**UK**	**Aim of the study**<br>**to evaluate and compare the effectiveness of pessaries and surgery in women with symptomatic pelvic organ prolapse.**<br><br>**Inclusion criteria**<br>**- Women referred to a specialist urogynaecology unit with symptomatic POP between June 2002 and May 2007**<br><br>**Exclusion criteria**<br>**- Subjects fitted with pessaries for urinary incontinence and those who had concomitant urinary incontinence surgery (e.g. TVT)**<br>**- Subjects who started in the pessary group but subsequently requested surgery were excluded from**	**Intervention**<br>**vaginal pessary**<br><br>**N = 359**	**Controls**<br>**surgery**<br><br>**N = 195**	**Primary outcomes:**<br>**Sheffield POP questionnaire (SPS-Q)**<br><br>**Secundary outcomes:**<br>**None**<br><br>**Follow up:**<br>**For the surgery and pessary groups 14 months (SD 6.14)**<br>**and 12 months (SD 3.1), respectively.**	**Primary outcomes:**<br>**No difference in functional outcome after 1 year follow-up between groups**<br><br>**Additional:**<br>**Only 45% in pessary group en 55% in surgery group responded at 12 months**<br>**In pessary group 24.7% (89/359) crossed to surgery but were not analyzed**<br>**In pessary group 7.3% stopped because of other reasons.**<br>**Selection and patient preference bias**<br>**The mean age was significantly higher in the pessary group compared to the surgery group (68.4 +/− 13.08 vs 60.4 +/−12.25 years, respectively).**

For peer review only - http://bmjopen.bmj.com/site/about/guidelines.xhtml

		**analysis in both the surgery and pessary group.**				

For peer review only

**Lowenstein 2010**<br><br>**J Sex Med 2010; 7: 1023-**<br><br>**28**	**Design**<br>**Cohort study**<br>**N= 235**<br><br>**Country**<br>**US**	**Aim of the study**<br>**First to evaluate patient-reported outcome, POP symptoms, sexual functioning and body image following treatment of POP. Second to compare surgery with pessary**<br><br>**Inclusion: ≥18 year, ≥ satge 2 POP, complete questionnaire at baseline and at ≥6 months follow up**<br><br>**Exclusion: recurrent UTI, peripheral neuropathy, using pessary at initial presentation or POP surgery < 6 months prior to presentation**	**Intervention**<br>**N = 202 surgery**	**Controls**<br>**N = 33 pessary**	**Primary outcomes**<br>**PFDI-20**<br>**PISQ-12**<br>**Modified Body Image scale**<br><br>**All at six months follow-up**	**Results**<br>**After multivariate analyses, including type of intervention, BMI and difference in Body image were associated with change in total PISQ (sexual functioning) score**<br><br>**In the pessary group there was no significant improvement in sexual functioning as compared to surgery (-2.5 versus +11.5)**<br><br>**Additional:**<br>**No figures presented for pessary and surgery group, with exemption of the Sexual functioning (PISQ-12) result above.**
**Barber, 2006**	**Design**<br>Case-control study<br><br>**N total = 106**<br><br>**Country:** USA	**Aim of the study**<br>**to evaluate the responsiveness of the Pelvic Floor Distress Inventory (PFDI) and Pelvic Floor Impact Questionnaire (PFIQ) in women with pelvic organ prolapse undergoing surgical and nonsurgical management.**<br><br>**Inclusion criteria**<br>**Surgery group:**<br>**Stage III or IV prolapse, were at least 18 years, and scheduled for vaginal prolapse repair.**<br>**Pessary group:**<br>**women with symptomatic pelvic organ prolapse of stage II or greater. (Pessri trial)**<br><br>**Exclusion criteria**<br>**Surgery group:**<br>**- mentally or physically incapable of completing the questionnaires.**<br>**Pessary group:**<br>**- were pregnant, were currently using a pessary, or had vaginal agglutination**	**Intervention**<br>**Pessary in women with stage II or greater POP**<br><br>**N = 42**	**Controls**<br>**Surgery in women with stage III or greater POP**<br><br>**N = 64**	**Primary outcomes:**<br>**PFDI and PFIQ**<br><br>**Secundary outcomes:**<br><br>**Follow up:**<br>**3 months (Pessary group) or 6 months (Surgery group) after initiation of treatment.**	**Primary outcomes:**<br><br>**After controlling for preoperative prolapse stage and baseline HRQOL scores, subjects in the Surgery group had significantly greater improvement in each of the scales of the PFDI and the prolapse and urinary scales of the PFIQ than did the Pessary group.**<br><br>**Scores from each of the scales of the PFDI improved by 14 to 15 points more on average after treatment in the Surgery group than those of the Pessary group (P < .01 for each) after adjusting for the above baseline differences.**<br><br>**Similarly, for the prolapse and urinary scales of the PFIQ, scores improved 13 and 17 points more, respectively, in the Surgery group than the Pessary group after treatment. (P < .05 for each).**<br><br>**Four of 64 (6%) of subjects in the Surgery group had recurrent prolapse develop beyond the hymen by 6 months after surgery. No subjects underwent reoperation for recurrent prolapse during the study period.**<br><br>**Additional:**<br><br>**Difference in follow up**<br>**Selection bias**

For peer review only - http://bmjopen.bmj.com/site/about/guidelines.xhtml

		**that precluded pessary insertion.**				

For peer review only

For peer review only - http://bmjopen.bmj.com/site/about/guidelines.xhtml

Appendix 3 Review on risk factors for failure of pessaries:

study		n	FU period	success initial	continued pes	stopped pes	cross over surgery	predict fit	predict contin
Wu 1997	retrospective	110	3 yrs	81 (74%)	43 (39%)		at least 5	nee	ja
Mokrzycki 2001	retrospective	42	3 mth		24 (57%)	18 (43%)	nr	nee	ja
Clemons 2004	prospective	100	1 yr	73 (73%)	43 (43%)		16/73 (27%)	nee	ja
Brincat 2004	retrospective	169	2,5 yr	144 (85%)	82 (60%)	54 (40%)	32/144 (22%)	nee	ja
Mutone 2004	retrospective	407	3 wks	168 (41%)		96 (57%)	nr	ja	ja
Powers 2006	retrospective	32	1 yr			20 (62%)	12 (38%)	nee	ja
Broens-Oostveen 2004	retrospective	192	9 yr			107 (52%)	nr	nee	ja
Nguyen 2005	retrospective	130	1 yr	111 (82%)	85 (65%)		20 (15%)	ja	ja
Hanson 2005	retrospective	1216	3 mth	1043 (86%)	744 (71%)		nr	nee	ja
Fernando 2006	prospective	203	4 mth	nr	153 (75%)		28 (14%)	nee	ja
Barber 2006	prospective	42	3 mth	nr		0	nr	nee	nee
Maito 2006	retrospective	120	6 mth	106 (88%)	92 (77%)		9 (8%)	ja	ja
Cundiff 2007	prospective	85	1 yr	65/71 (92%)			5 (6%)	nee	nee
Komesu 2007	prospective	64	6-12 mth		36 (56%)	28 (44%)	2 (3%)	nee	ja
Jones 2008	prospective	90	3 mth		42 (47%)		nr	nee	ja
Kuhn 2009	prospective	71	3 mth		32 (45%)		nr	nee	nee
Sarma 2009	retrospective	120	1 mth		45 (38%)		18 (15%)	nee	nee
Friedman 2010	retrospective	150	1 yr		115 (77%)	35 (23%)	25 (17%)	nee	ja
Patel 2010	prospective	65	3 mth	54 (83%)	54 (83%)		5 (8%)	nee	nee
Abdool 2010	prospective	359	1 yr	296 (83%)	243 (68%)		31 (9%)	nee	nee
Lone 2011	prospective	246	5 yr	187 (76%)	130 (53%)		70 (28%)	nee	nee
Markle 2011	retrospective	158	1 week	92 (59%)			nr	ja	nee
Chan 2012	prospective	197	1 yr	138 (70%)		59 (30%)	nr	nee	ja
Manchana 2012	retrospective	126	1 yr	77 (61%)	64 (51%)		5 (4%)	nee	nee
Mamik 2013	prospective	50	3 mth				5 (10%)	nee	nee
Geoffrion 2013	retrospective	101	4 wks	79 (78%)			nr	nee	ja
Alperin 2013	retrospective	4019	9 years				12% 1 yr, 24% 9 yrs	nee	nee
Succesfull fitting		3589		2714(76%)					
Continuation		3381			2027 (60%)				

For peer review only - http://bmjopen.bmj.com/site/about/guidelines.xhtml

Appendix 4 Review on risk factors for failure of surgery:

Risk factor	Investigated:	Significant:
Preoperative stage	8	5
Age	8	2
Obesity	7	0
Parity	5	0
Constipation	5	0
Pulmonary disease	5	0
Number of sites involved preoperative	4	1
Menopausal status	4	0
Hysterectomy status	4	0
Concomitent surgery	3	1
Family history	3	1
Complicated delivery	3	0
Diabetes	3	0
Smoking	3	0
Previous incontinence and/or prolapse surgery	2	2
Hiatus genitalis	2	1
Weight	2	1
Any incontinence preoperative	2	1
Delivery mode	2	0
Vaginal delivery	2	0
Hormone replacement therapy	2	0
Previous prolapse surgery	2	0
Surgeons experience	2	0
Abcense of posterior repair	1	1
Sexual activity	1	1
Levator defect	1	1
Height	1	0
Birth weight	1	0
Age at last delivery	1	0
Site of most advanced prolapse	1	0
Surgical approach	1	0
Use of Mesh	1	0
Previous incontinence surgery	1	0
Previous pelvic floor surgery or hysterectomy	1	0
Abdominal hernias	1	0
Cardiovascular disease	1	0
Intense physical exercise	1	0
Heavy lifting	1	0
Heavy lifting or constipation	1	0
Levator muscle contraction	1	0
Weight of the uterus	1	0
Postoperative complications	1	0
Incomplete emptying of bladder	1	0
Fecal incontinence	1	0

For peer review only

Appendix 5 tabel bezoeken, tijdstippen, onderzoeken

Chirurgie en cohort

Contact	Bezoek arts	POPQ	Eq5d doktersbezoek werkverzuim	PFIQ PFDI PISQ PGII PGIS
1. Eerste bezoek	X	X	Eq5D	X (zonder PGII)
2. 6 weken	X	X		
3. 3 maanden			X	
4. 6 maanden			X	
5. 12 maanden	X	X	X	X
6. 24 maanden	X	X	X	X

Pessarium met zelfmanagement

Contact	Bezoek arts	POPQ	Eq5d doktersbezoek werkverzuim	PFIQ PFDI PISQ PGII PGIS
1. Eerste bezoek	X	X	Eq5D	X (zonder PGII)
2. 6 weken	X	X		
3. 3 maanden			X	
4. 6 maanden			X	
5. 12 maanden	X	X	X	X
6. 24 maanden	X	X	X	X

For peer review only

Study protocol

Pessarium zonder zelfmanagement

Contact	Bezoek arts	POPQ	Ringcontrole	Eq5d doktersbezoek werkverzuim	PFIQ PFDI PISQ PGII PGIS
1. Eerste bezoek	X	X		Eq5D	X (zonder PGII)
2. 6 weken	X	X			
3. 3 maanden				X	
4. 4 maanden	X		X		
5. 6 maanden				X	
6. 8 maanden	X		X		
7. 12 maanden	X	X	X	X	X
8. 16 maanden	X		X		
9. 20 maanden	X		X		
10. 24 maanden	X	X	X	X	X

For peer review only

1.2 Final study protocol

Pessary or surgery for symptomatic pelvic organ prolapse

Version 1.~~21~~ 22 ~~April 2017~~February 2018

For peer review only

Study protocol

PROTOCOL TITLE 'Pessary or surgery for symptomatic pelvic organ prolapse'

Protocol ID	~~2017~~ 2018 / 1.~~21~~22
Short title	Pessary or surgery for symptomatic pelvic organ prolapse
EudraCT number	*Not applicable*
Version	1.~~21~~22
Date	April 2017
Coordinating investigator/project leader	Prof. Dr. C.H. van der Vaart, gynaecologist<br>University Medical Centre Utrecht
Principal investigator(s) (in Dutch: hoofdonderzoeker/ uitvoerder)	Dr. A. Vollebregt, gynaecologist<br>Spaarne Hospital<br><br>M.K. van de Waarsenburg, MD<br>University Medical Centre Utrecht
Multicenter: per site	Prof. Dr. C.H. van der Vaart, UMC Utrecht / Bergman Clinics Bilthoven<br>Dr. A.L. Milani, Reinier de Graaf Gasthuis<br>Dr. A. Vollebregt, Spaarne Hospital Hoofddorp<br>Dr. J.P.W.R. Roovers, Academic Medical Center / Bergman Clinics Amsterdam<br>Dr. K.B. Kluivers, Radboud UMC Nijmegen<br>Dr. V. Dietz, Catharina Hospital Eindhoven<br>Dr. H.W.F. van Eijndhoven, Isala Clinic Zwolle<br>Dr. M.M.A. Vernooij, Sint Antonius Hospital

For peer review only

For peer review only - http://bmjopen.bmj.com/site/about/guidelines.xhtml

	Nieuwegein<br>Dr. R.P. Schellart, Kennemer Gasthuis Haarlem<br>Drs. A.M.W. Broekman, Sint Franciscus Gasthuis Rotterdam<br>Drs. J. van Bavel, Amphia Hospital Breda<br>Dr. M. Weemhoff, Atrium MC Heerlen<br>Dr. ~~G. Link~~W.A. Spaans, Academisch ziekenhuis Maastricht<br>Dr. J.M. van der Ploeg, Martini ziekenhuis Groningen<br>Dr. D. Massop-Helmink, MST Enschede<br>Dr. J.M. Sikkema, ZGT Almelo / Hengelo<br>Dr. L. van der Voet, Deventer ziekenhuis<br>Dr. J.P. de Bruin, Jeroen Bosch ziekenhuis<br>Dr. C. Zeijl, Amstelland ziekenhuis<br>Dr. G.G. Bon, Tergooi ziekenhuis<br>Dr. S. de Vos – Stekelenburg, Albert Schweitzer ziekenhuis<br>Drs. K.L. Bos, Canisius Wilhelmina ziekenhuis<br>Prof. Dr. M.Y. Bongers – Maxima Medisch Centrum<br>Dr. W. Hermes – MCH-Bronovo<br>Dr. E. Janszen – OLVG<br>Dr. T. Huisman - HAGA
**Sponsor (in Dutch: verrichter/opdrachtgever)**	**Prof. Dr. C.H. van der Vaart, gynaecologist<br>University Medical Centre Utrecht**
**Subsidising party**	**ZonMw Project nr 837002525**
**Independent expert (s)**	**Dr. R.P. Zweemer<br>University Medical Centre Utrecht**

For peer review only

**Laboratory sites <*if applicable*>**	***Not applicable***
**Pharmacy <*if applicable*>**	***Not applicable***

For peer review only

PROTOCOL SIGNATURE SHEET

Name	Signature	Date
**Head of Department:**<br><br>**Prof. Dr. B. Veersema**<br>**Department of Reproductive Medicine and Gynaecology**<br>**University Medical Centre Utrecht**		
**Coordinating Investigator/Project leader:**<br><br>**Prof. Dr. C.H. van der Vaart, gynaecologist**		
**M.K. van de Waarsenburg, MD**<br>**University Medical Centre Utrecht**		

For peer review only

TABLE OF CONTENTS

1. INTRODUCTION AND RATIONALE 64
Problem definition 64
Relevance 65
2. OBJECTIVES 68
3. STUDY DESIGN 69
4. STUDY POPULATION 70
4.1 Population (base) 70
4.2 Inclusion criteria 70
4.3 Exclusion criteria 70
4.4 Sample size calculation 70
5. TREATMENT OF SUBJECTS 72
5.1 Investigational product/treatment 72
5.2 Use of co-intervention (if applicable) 73
5.3 Escape medication (if applicable) 73
6. INVESTIGATIONAL PRODUCT 74
6.1 Name and description of investigational product(s) 74
6.2 Summary of findings from non-clinical studies 74
6.3 Summary of findings from clinical studies 74
6.4 Summary of known and potential risks and benefits 75
6.5 Description and justification of route of administration and dosage 75
6.6 Dosages, dosage modifications and method of administration 75
6.7 Preparation and labelling of Investigational Medicinal Product 75
6.8 Drug accountability 75
7. NON-INVESTIGATIONAL PRODUCT 76
7.1 Name and description of non-investigational product(s) 76
7.2 Summary of findings from non-clinical studies 76
7.3 Summary of findings from clinical studies 76
7.4 Summary of known and potential risks and benefits 76
7.5 Description and justification of route of administration and dosage 76
7.6 Dosages, dosage modifications and method of administration 76
7.7 Preparation and labelling of Non Investigational Medicinal Product 76
7.8 Drug accountability 76
8. METHODS 77
8.1 Study parameters/endpoints 77
8.1.1 Main study parameter/endpoint 77
8.1.2 Secondary study parameters/endpoints (if applicable) 77
8.1.3 Other study parameters (if applicable) 77
8.2 Randomisation, blinding and treatment allocation 77
8.3 Study procedures (see also appendix 5) 78

For peer review only - http://bmjopen.bmj.com/site/about/guidelines.xhtml

8.4 Withdrawal of individual subjects ... 82
8.4.1 Specific criteria for withdrawal (if applicable) ... 82
8.5 Replacement of individual subjects after withdrawal ... 82
8.6 Follow-up of subjects withdrawn from treatment ... 82
8.7 Premature termination of the study ... 82
9. SAFETY REPORTING9.1 Section 10 WMO event ... 83
9.2 AEs, SAEs and SUSARs ... 83
9.2.1 Adverse events (AEs) ... 83
9.2.2 Serious adverse events (SAEs) ... 83
9.2.3 Suspected unexpected serious adverse reactions (SUSARs) ... 84
9.3 Annual safety report ... 84
9.4 Follow-up of adverse events ... 84
9.5 Data Safety Monitoring Board (DSMB) ... 84
10. STATISTICAL ANALYSIS ... 85
10.1 Primary study parameter(s) ... 85
10.2 Secondary study parameter(s) ... 85
10.3 Other study parameters ... 87
10.4 Interim analysis (if applicable) ... 87
11. ETHICAL CONSIDERATIONS ... 88
11.1 Regulation statement ... 88
11.2 Recruitment and consent ... 88
11.3 Objection by minors or incapacitated subjects (if applicable) ... 88
11.4 Benefits and risks assessment, group relatedness ... 88
11.5 Compensation for injury ... 88
11.6 Incentives (if applicable) ... 89
12. ADMINISTRATIVE ASPECTS, MONITORING AND PUBLICATION ... 90
12.1 Handling and storage of data and documents ... 90
12.2 Monitoring and Quality Assurance ... 90
12.3 Amendments ... 90
12.4 Annual progress report ... 90
12.5 End of study report ... 90
12.6 Public disclosure and publication policy ... 91
13. STRUCTURED RISK ANALYSIS ... 92
13.1 Potential issues of concern ... 92
13.2 Synthesis ... 92
14. REFERENCES ... 93
Appendix 1: ... 97
Appendix 2 ... 98
Appendix 3: ... 102
Appendix 4 ... 103
Appendix 5: ... 104

For peer review only - http://bmjopen.bmj.com/site/about/guidelines.xhtml

LIST OF ABBREVIATIONS AND RELEVANT DEFINITIONS

ABR	ABR form, General Assessment and Registration form, is the application form that is required for submission to the accredited Ethics Committee (In Dutch, ABR = Algemene Beoordeling en Registratie)
AE	Adverse Event
AR	Adverse Reaction
CA	Competent Authority
CCMO	Central Committee on Research Involving Human Subjects; in Dutch: Centrale Commissie Mensgebonden Onderzoek
CV	Curriculum Vitae
DSMB	Data Safety Monitoring Board
EU	European Union
EudraCT	European drug regulatory affairs Clinical Trials
GCP	Good Clinical Practice
IB	Investigator's Brochure
IC	Informed Consent
IMP	Investigational Medicinal Product
IMPD	Investigational Medicinal Product Dossier
METC	Medical research ethics committee (MREC); in Dutch: medisch ethische toetsing commissie (METC)
(S)AE	(Serious) Adverse Event
SPC	Summary of Product Characteristics (in Dutch: officiële productinfomatie IB1-tekst)
Sponsor	The sponsor is the party that commissions the organisation or performance of the research, for example a pharmaceutical company, academic hospital, scientific organisation or investigator. A party that provides funding for a study but does not commission it is not regarded as the sponsor, but referred to as a subsidising party.
SUSAR	Suspected Unexpected Serious Adverse Reaction
Wbp	Personal Data Protection Act (in Dutch: Wet Bescherming Persoonsgevens)
WMO	Medical Research Involving Human Subjects Act (in Dutch: Wet Medisch-wetenschappelijk Onderzoek met Mensen

For peer review only - http://bmjopen.bmj.com/site/about/guidelines.xhtml

SUMMARY

Rationale: Moderate to severe pelvic organ prolapse symptoms can be treated with pessary or surgery. Both treatments appear to be effective, but have not been compared directly.

Hypothesis: The strategy of pessary as initial therapy is as effective as direct surgery for moderate to severe POP, but it is associated with lower costs.

Objective: The primary objective is to compare the effectiveness and cost-effectiveness of pessary versus surgery as initial treatment for moderate to severe symptomatic pelvic organ prolapse (POP) in women at two year after initiation of treatment. The secondary objective is the development of a prediction model for failure of pessary use and surgery within 2 years.

Study design: Cohort study with embedded randomized controlled trial.

Study population: Treatment naïve women with POP who present with moderate to severe symptoms.

Intervention (if applicable): Pessary therapy or vaginal POP surgery.

Main study parameters/endpoints:

Primary outcome: Global impression of improvement of POP symptoms at 24 months measured with PGI-I

Secondary outcomes:

- Changes in symptom bother and disease-specific quality of life at 12 and 24 months follow-up
- Changes of sexual function at 12 and 24 months follow-up
- Changes in general quality of life at 3, 6, 12 and 24 months of follow up
- Adverse events/complications related to both treatment strategies during the study period
- Development of prediction model to identify factors for failing of pessary and surgery.
- Costs-effectiveness analyses

Nature and extent of the burden and risks associated with participation, benefit and group relatedness: Both treatment arms are routine treatments in the Netherlands. Patients in the RCT can have the risks of surgery instead of the risks from pessary therapy.

For peer review only - http://bmjopen.bmj.com/site/about/guidelines.xhtml

Study protocol

1. INTRODUCTION AND RATIONALE

Problem definition

Female pelvic organ prolapse (POP) is a common problem in women that negatively affects quality of life. The estimated prevalence of symptomatic POP among women between 45-85 years of age is 8.3 - 11% [1,2]. It is current practice in the Netherlands that the general practitioner (GP) treats the majority of women with POP symptoms. Women with moderate to severe POP symptoms are often referred to a gynecologist for treatment. This study focuses at the subgroup of moderate to severe POP.

Known effective treatment options for moderate to severe POP are pessary or surgery. A pessary has proven its effectiveness in the treatment of symptomatic POP, mainly in cystocele and uterine descent. However, studies are mainly observational in nature and inherently subject to selection and indication bias [3]. In literature, outcomes of pessary therapy are mainly recorded in terms of (dis-) continuation of therapy and to a much lesser extent in terms of symptom relief. The pessary continuation rate is 60% [3]. This is confirmed by a Dutch pilot study in 65 women that showed a satisfaction with pessary in 57% of womenand an operation rate of 43% at 12 months follow up [4]. In this study, 80% of women who continued pessary therapy reported much to very much improvement of their POP symptomsat 1 year follow up [4]. Reasons of discontinuation are pressure ulcer, vaginal discharge, discomfort or loss of fitting. These complications are reported to occur in up to 53% of women [5]. Half of them will decide to stop using pessary, but it is unclear which characteristics predict this outcome. Check-up of pessary therapy can be performed by either a general practitioner (GP), gynecologist or by self-management. According to a recent survey 50% percent of gynecologists involved in urogynaecology always offer self- management 40% on indication, and 10% never. Pessary therapy is inexpensive and costs are mainly related to doctor visits and treatment of side effects. In case of self-management costs might even be lower,

Surgery for POP results in much to very much improvement of symptoms in 80% of women and improvement of quality of life [6-9]. An anterior colporraphia is considered the standard procedure for a cystocele, as is the posterior colporraphia for a rectocele. For uterinedescent uterus sparing techniques, like sacrospinous hysteropexy (SH) and modified Manchester-Fothergill procedure, or vaginal hysterectomy can be performed [10-12]]. Complications of POP surgery are temporary urinary retention, temporary buttock pain in case of sacrospinous hysteropexy, urinary tract infection, hematoma or dyspareunia [11]. These complications seldom lead to persistent morbidity. The most

For peer review only - http://bmjopen.bmj.com/site/about/guidelines.xhtml

common “complication” is the recurrence of symptomatic POP or de novo stress- incontinence that may lead to additional surgery, pessary therapy, or pelvic floor physiotherapy. As part of a RCT, comparing mesh with fascia plication, we found that
11% of women needed additional surgery after anterior colporraphia at 24 months follow up [7,9]. As in pessary therapy, the characteristics that predict successful or unsuccessfulsurgical therapy are largely unknown.

The decision which treatment option to choose depends on both patient and doctor’s preferences. In our pilot survey 70% of gynecologists informed their patients about the possibility of pessary therapy, but it is unknown how many women actually received a pessary. A recent Dutch study showed that 48% of treatment-naive women preferred surgery, 36% a pessary and 16% had no preference [28]. It is therefore reasonable to assume that at least 50% of treatment naïve women with moderate to severe prolapse symptoms will have surgery as primary treatment.

Although clinical efficacy appears to favor surgery [3], the large variation in study design, outcome measurements and loss to follow up makes any comment on the best treatment option speculative. This is recognized in two recent reviews on the subject that both urge the need for randomized trials comparing surgery and pessary for POP [13,14]. Efficacy can be expressed in terms of clinical outcome but also in terms of cost-effectiveness. It is obvious that surgery (especially hospital costs) is much more expensive than pessary therapy, butthe cost-effectiveness of the surgical or pessary strategy has never been assessed. Based on current cohort and case-control studies we hypothesize that a strategy of initial pessary therapy for moderate to severe POP, is more cost-effective than surgery.
We propose to perform a randomized controlled trial to generate evidence for the optimal and most cost-effective primary treatment for moderate to severe POP, including a better a priori patient selection for treatment by identifying factors of failure for pessary therapy or surgery.

Relevance

At present a national multidisciplinary guideline on the diagnosis and treatment of POP is completed. The guideline identifies the lack of evidence with respect to the best treatment option for moderate to severe prolapse, a conclusion that is confirmed by the 2013 Cochrane Collaboration review [13]. In this evidence “vacuum” both doctors and patient preferences rule, but unfortunately these are not supported by facts. If we look at the available data the following

For peer review only - http://bmjopen.bmj.com/site/about/guidelines.xhtml

calculation emerges.

About 60% of women who start pessary therapy in the specialist care setting will continue using it at one year [4,15]. Eighty percent of them will report much to very much improvement, resulting in an overall 48% much to very much improvement. The majority ofthe 40% of women who are not satisfied with pessary therapy will request or are offered additional surgery. After surgery 80% of women report much to very much improvement ofPOP symptoms [6]. Combining these percentages, women who originally started withpessary therapy may also expect 80% (48% after initial pessary treatment + 32% afteradditional surgery =) much to very much improvement. Based on these estimates it isexpected that the outcome of both treatment strategies will eventually result in a globalimprovement of symptoms in 80% of women. With equal clinical outcomes of both strategiesthe costs needed to obtain these outcomes become crucial. With the exception of a cost calculation based on a Markov model, no direct cost-effectiveness studies on the use ofpessary or surgery for POP have been performed [16]. The relevance of this project, with the high prevalence of POP worldwide, associated costs and insufficient evidence, is high. Wehave searched the www.clinicaltrials.gov database (3th March 2014) on similar studies(comparing pessary with surgery) but none were found.

However, if we were to prove that pessary therapy is more cost-effective then surgical treatment, this does not imply that a trial of pessary should always be undertaken. There is also insufficient evidence on which patient characteristics are associated with failure of pessary treatment or surgery (systematic review). The knowledge on how to predict which women will have a very low chance of success with pessary therapy can further improve effective treatment strategy management. This will contribute to treatment efficacy. This is not only very relevant for the hospital specialist care setting, but this knowledge can also be implemented in general practitioner practice units.

There is very limited evidence on the optimal management strategy for pessary cleaning, both in time interval as well as in who should perform the cleaning. Our study is unique and therefore relevant since self-management is advocated in the study setting. This will not only allow it to obtain data in a standardized way, but also involves the woman in her own management. This involvement is strongly advocated by two major gynecologic patient organizations ('Patienten Gynaecologie Nederland' and the 'Stichting Bekkenbodem Patienten'). These two organizations, as well as the Dutch urogynaecological consortium have identified this study to be highly relevant.

For peer review only - http://bmjopen.bmj.com/site/about/guidelines.xhtml

In line with the report "Medisch Specialistische zorg 20/20" we are heading towards integral health care in which the general physician and medical specialist will work more closely together, using the same treatment protocol for various illnesses. The information and conclusions of this trial will add level I scientific evidence to such an integral protocol and guideline for women with symptomatic POP. This will aid in a better patient selection that will need referral to the specialist. The data on patient's self-management of pessary treatment will supply information for patient instructions, which are relevant for information leaflets on the subject.

For peer review only

2. OBJECTIVES

The aim of this multicenter pragmatic cohort study with embedded randomized controlled non-inferiority trial comparing pessary therapy versus surgery is twofold:

1. To prospectively compare the effectiveness and cost-effectiveness of pessary therapy or surgery as primary treatment of moderate to severe symptomatic cystocele, uterine descent and/or rectocele in women at two year after initiation of treatment, in randomized trial embedded in a preference cohort.
2. To compare the effectiveness between the cohort and randomized trial.
3. To develop a prediction model for failure of pessary use and surgery within the first years.

For peer review only

3. STUDY DESIGN

Multicenter pragmatic cohort study with an embedded randomized controlled non-inferiority trial comparing pessary [CE 0086] therapy and surgery including an economic evaluation. The follow up will be 24 months.

A short (30 minutes) trial of pessary fitting is part of our protocol. This ensures that only women who fit both treatment options enter the randomization procedure. The trial is short and only aims at fitting, not symptom relief. Women with an unsuccessful pessary fitting will be followed in the cohort fitting failure. In case the woman is willing to participate but actively opts for one of both treatments, she will be followed in a cohort.

See also appendix 1 and 5.

For peer review only

For peer review only - http://bmjopen.bmj.com/site/about/guidelines.xhtml

4. STUDY POPULATION

4.1 Population (base)

All women with a symptomatic POP will be included.

4.2 Inclusion criteria

In order to be eligible to participate in this study, a subject must meet all of the following criteria:

1. Women with a prolapse stage 2 or more.
2. Women with moderate to severe POP symptoms. Moderate to severe POP symptoms is defined as a prolapse domain score > 33 on the validated Dutch version of the Pelvic Floor Distress Inventory (PFDI-20) [8, 23, 24].
3. For the RCT: Women who have had a successful pessary fitting procedure.
4. Written informed consent.

4.3 Exclusion criteria

A potential subject who meets any of the following criteria will be excluded from participation in this study:

1. Prior urogynaecological (prolapse or incontinence) surgery
2. Probability of future childbearing
3. Insufficient knowledge of the Dutch language
4. Co-morbidity causing increased surgical risks at the discretion of the surgeon
5. Major psychiatric illness
6. Prior pessary use

4.4 Sample size calculation

With 198 women per group, we will have 80% power to reject the null hypothesis that pessary therapy is inferior to surgery, with a 1-sided alpha of 0.05, a non-inferiority margin of 10% and the proportion in the standard group is 80% (NQueryAdvisor). Accounting for 10% loss to follow-up we plan to randomize 436 patients.

The sample size calculation for prediction models is based on the number of failures of pessary or surgical therapy. For each potential predictor in the model we need 10-15 failures. Our pessary group sample size is 198 women. An estimated 40% (80 women) will cross over to surgery and can be regarded as failures. Our sample size is therefore sufficient

For peer review only

For peer review only - http://bmjopen.bmj.com/site/about/guidelines.xhtml

to develop the prediction model for failure of pessary therapy for 6 to 8 items. In the surgery group 20% of women will not be satisfied with the result of treatment. With the same sample size of 198 women, the 40 women who are dissatisfied allow us to study up to 4 potential predictive factors.

In the cohort we include all patients who are willing to collaborate on this research but have a preference for one of both therapies. We now assume that 70% of the eligible patients object participation in the RCT, and that 90% of them is nevertheless willing to participate in the cohort.

For peer review only

5. TREATMENT OF SUBJECTS

5.1 Investigational product/treatment

Pessary [CE 0086] therapy and surgery both are options for the treatment of a symptomatic POP. Ten large urogynecological units (university hospitals or teaching hospitals) that have worked together in previous consortium studies will participate in this multicenter trial. All participating gynaecologists have fitted at least 100 pessaries and performed more than 100 surgical POP procedures prior to the start of this study.

All pessaries are made of modern silicon material. All types of pessaries, both supportive and occlusive/space filling are allowed according to the judgment of the gynaecologist. A recent randomized trial comparing supportive (ring) and occlusive (Gelhorn) showed no differences [17]. After placing the pessary, all women will receive verbal and written instructions on the self-management of pessary therapy.

The first pessary follow up visit will always be performed by the gynaecologist. In case of self-management the frequency of cleaning is left to her personal judgment, but may not exceed 4 months. If self-management is not possible, women will be seen at 4 months intervals for pessary cleaning and vaginal inspection, preferable by their GP. In case of vaginal atrophy topical estrogens will be advised according to pharmaceutical guidelines. The diagnosis of atrophy is left to the judgment of the treating physician, since no clear definition for atrophy is available yet [18].

All surgical procedures will be performed according to our national guidelines. In this pragmatic trial the decision which technique to use is left, to the discretion of the gynaecologist, within the limitations below [19]. Cystocele repair will consist of conventional anterior colporrhaphy [9]. For uterine descent different techniques are allowed [20]. These techniques can either be uterus sparing (sacrospinous hysteropexy [10], modified Manchester-Fothergill procedure [12] or a abdominal sacrocolpopexy [9]) or a vaginal hysterectomy. Recent studies showed similar effectiveness on both anatomical and functional outcomes for these different techniques [10, 12, 21]. A coexistent stage 2 rectocele repair will be a conventional colporrhaphia posterior. All procedures are performed under general or spinal anesthesia and under antibiotics and thrombosis prophylaxis according to local protocols.

For peer review only - http://bmjopen.bmj.com/site/about/guidelines.xhtml

5.2 Use of co-intervention (if applicable)

Not applicable.

5.3 Escape medication (if applicable)

Not applicable.

For peer review only

For peer review only - http://bmjopen.bmj.com/site/about/guidelines.xhtml

6. INVESTIGATIONAL PRODUCT

6.1 Name and description of investigational product(s)

6.2 Summary of findings from non-clinical studies

Not applicable.

6.3 Summary of findings from clinical studies

Three systematic reviews of the literature were performed by four members of our Dutch urogynaecology consortium (details in appendix 2-4) that concluded:

1. Systematic review on the (cost)effectiveness of pessary use compared to surgery

There are a very limited number of comparative studies on the efficacy of surgery or pessary use for POP. The differences in study population, inclusion criteria, follow-up period, large numbers of loss to follow-up, different outcome measures makes interpretation difficult if not impossible. The two studies that presented data on functional outcome in terms of prolapsed symptom reduction were favorable for surgery (appendix 2).

2. Systematic review of factors influencing pessary fitting and continuation

A systematic review was performed to identify the satisfactory pessary fitting rate and the continuation rate of pessary use. The factors influencing these rates as well as the cross over to prolapse surgery were identified from previous studies (appendix 3).

Summarizing the results show that an estimated 75% of women will have a successful fitting and 59% will continue pessary use at variable follow-up between 3 months and 5 years. In these 18 studies, 8 factors have been tested more than 4 times as prognostic factor of successful pessary use: Stress urinary incontinence was found associated with discontinuation of pessary in 5 out of 7 studies. In 7 out of 10 studies previous prolapse surgery or hysterectomy was associated with less continuation of pessary use. Higher age was related to continuation of pessary use in 3 out of 6 studies, whereas no correlation was found in the other studies. In 1 out of 4 studies sexual activity was related to longer pessary use, whereas in 1 out of 4 related to the choice for surgery. In the two other studies no correlation was found. In one study where the prolapse in a specific vaginal compartment was related to outcome, nor cystocele was related to longer pessary use.

Parity en menopausal status and hormonal replacement were mostly not related to continued pessary use.

For peer review only - http://bmjopen.bmj.com/site/about/guidelines.xhtml

3. Review of factors influencing failure of POP surgery.

A systematic review of factors influencing failure of POP surgery was performed concerning recurrence after surgery (surgery failure). There were 1 case control study, 3 prospective studies and 6 retrospective studies. There were 2298 women included in the studies. Forty-four (44) potential risk factors have been studied, of which 12 risk factors have at least once been identified as statistically significant risk factors in a multivariate logistic regression analysis (appendix 4).

6.4 Summary of known and potential risks and benefits

The present study carries low risks for the participant. Pessary [CE 0086] or surgery is standard care for symptomatic pelvic organ prolapse. Known risks for surgery are blood loss, risk of infection, dyspareunia, urine incontinence or a recurrence of a symptomatic pelvic organ prolapse.

The benefit of the study lies in a better understanding of satisfaction and cost effectiveness.

6.5 Description and justification of route of administration and dosage

Not applicable

6.6 Dosages, dosage modifications and method of administration

Not applicable

6.7 Preparation and labelling of Investigational Medicinal Product

Not applicable

6.8 Drug accountability

Not applicable

For peer review only - http://bmjopen.bmj.com/site/about/guidelines.xhtml

7. NON-INVESTIGATIONAL PRODUCT

Not applicable.

7.1 Name and description of non-investigational product(s)

Not applicable.

7.2 Summary of findings from non-clinical studies

Not applicable.

7.3 Summary of findings from clinical studies

Not applicable.

7.4 Summary of known and potential risks and benefits

Not applicable.

7.5 Description and justification of route of administration and dosage

Not applicable.

7.6 Dosages, dosage modifications and method of administration

Not applicable.

7.7 Preparation and labelling of Non Investigational Medicinal Product

Not applicable.

7.8 Drug accountability

Not applicable.

For peer review only

8. METHODS

8.1 Study parameters/endpoints

8.1.1 Main study parameter/endpoint

The primary outcome of this study is the percentage of women with much or very much improvement of POP symptoms at 2 years follow-up, as measured with the Patient Global Impression of Improvement (PGI-I) scale [22].
PGI-I is a 7-point Likert scale, with scores ranging from very much worse to very much improved. Success is defined as ‘much or very much’ improvement.

8.1.2 Secondary study parameters/endpoints (if applicable)

1. Changes in symptom bother and quality of life at 12 and 24 months follow up.
2. Changes in sexual function at 12 and 24 months follow up.
3. Changes in general quality of life at 3, 6, 12 and 24 months.
4. Adverse events/complications related to both treatment strategies.
5. Development of prediction model to identify fail factors for pessary and surgery
6. Cost-effectiveness

8.1.3 Other study parameters (if applicable)

Baseline characteristics: Age; ethnicity; allergies; smoking; obstetric history including number and mode of deliveries; menopausal status; hormone use; use of medication; height; weight; co-morbidity (diabetes mellitus, COPD); history of gynaecological operations; family history of prolapse; duration of complaints;.
Physical examination: time, POP-Q, atrophy, vulvar deviations, stress test.
Brand pessary, type of surgery.

8.2 Randomisation, blinding and treatment allocation

After written informed consent is obtained, and inclusion and exclusion criteria are assessed, women will be randomly allocated in a 1:1 ratio to either treatment with a pessary or surgical treatment. Randomization will be done web based using ALEA, the software for randomization in clinical trials currently used by most studies in the Dutch consortium for studies on women’s health and reproduction studies. The randomization sequence will be computer generated using variable blocks of two and four, stratified for centre.
After entering the woman’s initials and confirming inclusion criteria on the website, a

For peer review only - http://bmjopen.bmj.com/site/about/guidelines.xhtml

unique number for randomization will be generated and the allocation code will be disclosed. This unique number cannot be deleted afterwards. This study will be open label because the nature of the intervention meant that masking to the intervention was not possible. Women who attend the cohort fitting failure will also be registered in ALEA.

In case the woman is willing to participate but actively opts for one of both treatments, she will also be registered in ALEA.

All groups will have the same data collection and follow up as displayed in appendix 5. We expect differences in the study parameters between RCT and cohort, in effectivity, satisfaction and cost effectivity.

8.3 Study procedures (see also appendix 5)

This study will be performed within the Dutch Urogynaecology Research Consortium, a subdivision of the Dutch Consortium for studies on women's health. Infrastructure (research nurses for counseling and data-monitoring, the use of web-based data entry), expertise on methodology and cost-effectiveness is shared.

1. Symptom bother and disease-specific quality of life are measured with the Pelvic Floor Distress Inventory (PFDI-20) and Pelvic Floor Impact Questionnaire (PFIQ) [23,24]. The validated version of the Dutch PFDI consists of five domains: pelvic organ prolapse, urinary incontinence, overactive bladder, pain, and obstructive micturition. The PFIQ measures the impact of urogenital symptoms on quality of life and consist of five domains: physical functioning, mobility, emotional health, embarrassment and social functioning.

2. Sexual function is measured with the PISQ-R. It is an international disease-specific questionnaire that measures sexual functioning in sexually active and inactive participants [26].

3. Generic quality of life is measured with the EQ-5D and a questionnaire "doktersbezoek".

4. The adverse events of surgery recorded will consist of; direct peri-and postoperative complications (bleeding, pain and infection); interventions for complications; recurrent prolapse; de novo stress urinary incontinence. The adverse events of pessary recorded will consist of; discharge; pain; discomfort; bleeding; involuntary loss of pessary; de novo stress urinary incontinence.

5. The development of a prediction model is separately described in paragraph "data analyses".

6. The economic evaluation is described below.

For peer review only - http://bmjopen.bmj.com/site/about/guidelines.xhtml

ANTICIPATED COST-EFFECTIVENESS/BUDGET IMPACT

Cost differences between the two strategies are mainly the result from differences in costs associated with the initial intervention. Cost of a POP procedure is estimated at 4000 euros direct medical costs, and 4000 euros associated with lost productivity (indirect costs) if a societal perspective is used. The direct cost of pessary use is estimated at 200 euros, including costs for the pessary itself (50 euros) and consultations in the first year (150 euros). The estimated cost differences between the two strategies depend on the extent that women are (and remain) satisfied with the initial procedure (surgical or pessary): in case of dissatisfaction with the procedure, additional costs are generated by a subsequent intervention ((re-)operation, pessary, or pelvic physiotherapy). The flowchart (see appendix 1) illustrates the expected outcomes for each strategy. Based on the assumptions reflected in this flowchart, combined with approximate estimates for unit costs for POP surgery, pessary, GP and specialist visits, the anticipated impact on the annualhealth care budget as well as societal costs were estimated.

At present, the primary therapy for women presenting with moderate to severe POP is either surgery or pessary. The exact ratio is unknown, but is probably 50/50. If 50% women would receive primary surgery the current medical costs amount to 34 million Euros. If all women would start with pessary therapy, these costs would be 20 million euros, and the potential budget impact would be 14 million Euros/year. Asit is not realistic that all women will start with pessary if this strategy proves to be successful, at 85% implementation of the pessary strategy, the annual budgetimpact will be around 10 million euros. The economic impact to society (including indirect (productivity) costs) will be 28 million euros and 20 million euros, at 100% and 85% implementation, respectively.

Sensitivity analyses showed, that these “base case” results are affected by estimated unit costs for POP surgery (direct and indirect costs) and the satisfaction rate for pessary, relative to surgery, but even the most conservative assumptions would lead to major cost savings for the health care budget (5 million euros) and society (15 million euros).

ECONOMIC EVALUATION

The economic evaluation will be based on the randomized trial. Considering the non-inferiority design of the study, we will not be able to rule out a small but acceptable

For peer review only - http://bmjopen.bmj.com/site/about/guidelines.xhtml

difference in favor of POP surgery. Consequently, the economic evaluation will be setup as a cost-effectiveness analysis, where cost-effectiveness will be expressed as costs per improvement outcome (much or very much improvement on the Patient Global Impression of Improvement (PGI-I)), and the incremental cost-effectiveness ratio as costs saved per additional case of unsatisfactory outcome. We will also perform a cost-effectiveness analysis using QALYs as health outcome, to express the difference between the two strategies in terms of costs (saved) per QALY (lost).

The economic evaluation will therefore encompass a cost-effectiveness analysis (CEA), a cost-utility analysis (CUA) as well as a budget impact analysis (BIA) from a health care budget and a societal perspective, with a time horizon between randomization and 2 years follow up. The primary outcome in the cost-effectiveness analysis will be costs per satisfactory outcome (primary clinical outcome), and the incremental cost-effectiveness ratio will reflect the costs saved per additional case of unsatisfactory outcome. As we hypothesize that pessary as a primary strategy in these patients does not result in more unsatisfactory outcomes, increased use of pessary will result in a decrease in the number of POP surgeries, and associated costs of hospital stay, recovery and (from a societal perspective) productivity loss (non-inferior strategy at lower costs).

Based on data actually observed in the trial, total costs associated with both surgery and pessary as a primary strategy will be estimated. Total costs can be divided into direct medical costs, non-medical costs and indirect costs. Direct medical costs are generated by utilization of primary or secondary health care services (including POP surgery, hospital stay, diagnostic procedures, medication). Non-medical costs are generated by travel expenses, and informal care; and indirect costs result from lost productivity due to absence from work or lost opportunity for non-paid activities. Non-medical and indirect costs are only included in the analysis from a societal perspective.

Resource utilization will be documented in the clinical report form (CRF) and complementary patient questionnaires, based on the Medical Consumption Questionnaire (MCQ) and Productivity Costs Questionnaire (PCQ) [29,30]. In patients for whom complete follow-up is not available, cost and quality-of-life datawill be extrapolated using multiple imputations. Unit costs will be based on Dutch guideline prices (for primary and secondary health services, informal care and lost productivity),

For peer review only - http://bmjopen.bmj.com/site/about/guidelines.xhtml

and market prices (for medication)[31,32]

Similarly, the incremental costs per QALY gained will be estimated over a period of two years. Health state utilities to estimate QALYs will be derived from an EQ-5D measurement at discharge, as well as at follow-up assessments. Utility values for EQ-5D scores will be based on UK-estimates (Dolan, 1997). Utility scores will be linearly interpolated, assuming constant increase/decrease between subsequent assessments.

Robustness for sampling uncertainty as well as uncertainty associated with cost estimates and assumptions will be assessed in sensitivity analyses, including: Dutch health states (Lamers, 2005) instead of the UK based model in the main analyses; and varying unit costs for pertinent volumes of health care utilization (e.g. costs of POP surgery, pessary use, productivity costs).

The incremental costs and effects will be depicted in a cost effectiveness plane and cost-effectiveness acceptability curves providing information directly interpretable as the probability of one intervention being cost-effective compared to the alternative given a ceiling ratio that policy makers are willing to invest.

BIA

In a budget impact analysis, study results will be extrapolated to the national level to estimate the total impact on the health care budget per annum for the Netherlands in terms of cost reduction and health outcomes (satisfactory outcomes as well as QALYs). As economic consequences of the intervention are expected to spanmultiple years, this accumulation of cost (savings) will be reflected in the budget impact analyses.

The Budget Impact Analysis will be executed according to the international ISPOR guidelines [33]. This framework for creating a budget impact model includes formalized guidance about the acquisition and use of data in order to make budget projections. In addition to the societal perspective, the BIA will therefore be also report economic consequences from the perspective of the Dutch budgetary health care framework (BKZ). If the probability of an unsatisfactory outcome exceeds the non-inferiority limit, recommending pessary as primary treatment for all women isnot feasible, and an economic evaluation/budget impact analysis is not sensible. To estimate costs, we will follow the Handleiding Kosten onderzoek CVZ 2010.

For peer review only - http://bmjopen.bmj.com/site/about/guidelines.xhtml

8.4 Withdrawal of individual subjects

Subjects can leave the study at any time for any reason if they wish to do so without any consequences. The investigator can decide to withdraw a subject from the study for urgent medical reasons.

8.4.1 Specific criteria for withdrawal (if applicable)

Not applicable.

8.5 Replacement of individual subjects after withdrawal

We will not replace patients who withdrew informed consent. We will replace patients that are randomized by mistake, for example because of technical errors with online randomization.

8.6 Follow-up of subjects withdrawn from treatment

Patients withdrawn from the intervention but not from informed consent will be followed up.

8.7 Premature termination of the study

This study includes standard care, therefore it is very unlikely that unexpected complications will occur. Therefore premature termination is not applicable.

For peer review only

9. SAFETY REPORTING

9.1

Temporary halt for reasons of subject safety (section 9.1, CCMO Template Research Protocol)

In accordance to section 10, subsection 4, of the WMO, the sponsor will suspend the study if there is sufficient ground that continuation of the study will jeopardise subject health or safety. The sponsor will notify the accredited METC with undue delay of a temporary halt including the reason for such an action. The study will be suspended pending further review by the accredited METC. The investigator will take care that all subjects are kept informed.

Temporary halt and (prematurely) end of study report (section 12.5, CCMO Template Research Protocol)

The sponsor will notify the METC immediately of a temporary halt of the study, including the reason of such an action.

9.2 AEs, SAEs and SUSARs

9.2.1 Adverse events (AEs)

Adverse events are defined as any undesirable experience occurring to a subject during the study, whether or not considered related to the study. All adverse events reported spontaneously by the subject or observed by the investigator or his staff will be recorded. During visits complaints will be questioned systematically.

9.2.2 Serious adverse events (SAEs)

A serious adverse event is any untoward medical occurrence or effect that at any dose:

- results in death;
- is life threatening (at the time of the event);
- requires hospitalisation or prolongation of existing inpatients' hospitalisation (>4 days);
- results in persistent or significant disability or incapacity;
- is a congenital anomaly or birth defect;
- Any other important medical event that may not result in death, be life threatening, or require hospitalization, may be considered a serious adverse experience when,

For peer review only - http://bmjopen.bmj.com/site/about/guidelines.xhtml

based upon appropriate medical judgement, the event may jeopardize the subject or may require an intervention to prevent one of the outcomes listed above.

SAEs have to be reported when its occurrence appears in two days after the study operations. The investigators in participating centres should inform the coordinating investigator as soon as possible but at least the next working day.

The sponsor will report the SAEs through the web portal *ToetsingOnline* to the accredited METC that approved the protocol, within 15 days after the sponsor has first knowledge of the serious adverse events.

SAEs that result in death or are life threatening should be reported expedited. The expedited reporting will occur not later than 7 days after the responsible investigator has first knowledge of the adverse event. This is for a preliminary report with another 8 days for completion of the report.

9.2.3 Suspected unexpected serious adverse reactions (SUSARs)

Not applicable.

9.3 Annual safety report

The annual safety report will be combined with the annual progress report (see chapter 12.4).

9.4 Follow-up of adverse events

All AEs will be followed until they have abated, or until a stable situation has been reached. Depending on the event, follow up may require additional tests or medical procedures as indicated, and/or referral to the general physician or a medical specialist.

SAEs need to be reported till end of study within the Netherlands, as defined in the protocol

9.5 Data Safety Monitoring Board (DSMB)

Since both techniques are standard practice, and no major unexpected complications are foreseen, no interim analysis is planned. A Data Safety and Monitoring Board will not be installed, as both procedures are regularly used and acceptable options in current clinical practice.

For peer review only - http://bmjopen.bmj.com/site/about/guidelines.xhtml

10. STATISTICAL ANALYSIS

The results of the study will be reported according to the CONSORT statement.

The main outcomes will be analysed and presented according the intention-to-treat principle. Since in this pragmatic trial we expect that in the pessary strategy group 40% of women will cross over to surgery an additional per protocol analyses is foreseen. This will provide more insight in the effect of surgery after pessary therapy as compared to surgery or pessary therapy alone.

We plan a subgroup analysis for the location of the prolapse: anterior prolapse versus posterior prolapse.

The cohort with patients treated according their preference will be analysed separately from the randomized trial, and presented in the same manuscript, which will provide insight into the generalizability of the results.

10.1 Primary study parameter(s)

The primary outcome, success (much or very much improvement) or no success (a little better, no change, a little worse, much worse or very much worse) on the PGI-I will be expressed in percentage point differences. Differences between the percentages will be tested using a chi-square test. A p-value <0.05 will be considered statistically significant.

For the cohort study results will be presented separately, and the same analyses will be done. Differences between the trial arm and the cohort arm will be tested using the chi-square test, to determine the generalizability of the results.

10.2 Secondary study parameter(s)

The PFDI, PFIQ and PISQ-r are all interval scales. Differences between baseline and 12 and 24 months follow up will be assessed using an independent t-test when normality can be assumed, or by non-parametric tests when the data are not normally distributed. Effect sizes will be calculated to estimate the magnitude of changes.

Differences in EQ5-D scores and “ziekteverzuim” between baseline and at 3, 6, 12 and 24 months will be assessed using t-test and further incorporated in the cost-effectiveness analyses.

Imputation statistics will be used or missing data.

(Serious) adverse events will be categorized and chi-square statistics, with calculation of relative risks when appropriate, will be applied in analyses.

For peer review only - http://bmjopen.bmj.com/site/about/guidelines.xhtml

Prediction model

A prediction model that uses predefined variables, as potential predictors of failure of pessary therapy, will be developed using multivariable regression analysis.Missing data will be imputed.

Predictors for failure derived from literature are a large genital hiatus (gh > 4 cm), women being sexually active, age > 65 years, prolapse POP-Q stage 3, previous hysterectomy. If applicable, a prediction rule for the chance of failure of pessary therapy will be constructed, which could be presented as a normogram which could be used to determine the chance of failure on pessary therapy.

Internal validity will be assessed using bootstrapping techniques; shrinkage will be applied to the parameter estimates. Model performance will be assessed with discriminative capacity and calibration. Calibration will be assessed by comparing the mean predicted probability that patients failed on pessary therapy with the mean observed probability that patients failed on pessary therapy. To do so, the total cohort will be split into ten groups based on the deciles of the predicted probability. Per group the mean predicted probability will be calculated as well as the mean observed predicted probability. Discriminative capacity of the model will be assessed with receiver operation characteristics (ROC) analysis and the area under the ROC curve (AUC).

We will also look at factors that could explain failure of surgery. Our systematic review on POP and recurrent POP after surgery showed that 6 preoperative items, eg. POP stage, age, family history, preoperative incontinence, previous POP or incontinence surgery, previous hysterectomy seems to be predictive for recurrence.

Women with previous POP or incontinence surgery are excluded from our study, leaving 4 predefined potential predictive factors. After the 2 year follow-up has been performed, we will reconsider which factors to include in a prognostic model, based on the current literature. We will select predictors from literature with the highest predictive value, where about 1 predictor could be selected for each 10 surgery failures.

Using interaction terms the effect of a differential effect in women with a higher age (>median) or a lower age (<=median), a higher (>25) or lower BMI(<=25) will be assessed for both pessary as well as surgery failures prediction.

For peer review only - http://bmjopen.bmj.com/site/about/guidelines.xhtml

10.3 Other study parameters

Not applicable.

10.4 Interim analysis (if applicable)

Not applicable, because of the non-inferiority design with low risk and the possibility of cross over.

For peer review only

For peer review only - http://bmjopen.bmj.com/site/about/guidelines.xhtml

11. ETHICAL CONSIDERATIONS

11.1 Regulation statement

This study will be conducted according to the principles of the Declaration of Helsinki (version 10, October 2013) and in accordance with the Medical Research InvolvingHuman Subjects Act (WMO) and other guidelines, regulations and Acts.

11.2 Recruitment and consent

Women with symptomatic pelvic organ prolapse who attend the outpatient clinic will be informed about the study by the gynaecologist or nurse. After checking the in- and exclusion criteria the women will receive verbal and written information about the study. If the woman is willing to participate she is asked to sign the informed consent. All women will undergo the pessary fitting test which is part of the standard evaluation. All women will be contacted at a minimum interval of 1 week. Those women who failed the initial fitting will be offered surgery and attend the cohort fitting failure, the women with a succesfull initial fitting will be asked to enroll in the RCT. In case the woman is willing to participate but actively opts for one of both treatments, she can attend the cohort. Her motivation is requested. In case the women is not willing to participate, she will be registred as “refuser”.

11.3 Objection by minors or incapacitated subjects (if applicable)

Not applicable.

11.4 Benefits and risks assessment, group relatedness

The present study carries no risks for the participant. Pessary or surgery are standard care for symptomatic pelvic organ prolapse. The benefit of the study lies in a better understanding of satisfaction and cost effectiveness.

11.5 Compensation for injury

The sponsor/investigator has a liability insurance which is in accordance with article 7, subsection 9 of the WMO.

The sponsor (also) has an insurance which is in accordance with the legal requirements in the Netherlands (Article 7 WMO and the Measure regarding Compulsory Insurance for Clinical Research in Humans of 23th June 2003). This insurance provides cover for

For peer review only - http://bmjopen.bmj.com/site/about/guidelines.xhtml

damage to research subjects through injury or death caused by the study.

1. € 450.000,-- (i.e. four hundred and fifty thousand Euro) for death or injury for each subject who participates in the Research;
2. € 3.500.000,-- (i.e. three million five hundred thousand Euro) for death or injury for all subjects who participate in the Research;
3. € 5.000.000,-- (i.e. five million Euro) for the total damage incurred by the organisation for all damage disclosed by scientific research for the Sponsor as 'verrichter' in the meaning of said Act in each year of insurance coverage.

The insurance applies to the damage that becomes apparent during the study or within 4 years after the end of the study.

11.6 Incentives (if applicable)

Not applicable.

For peer review only

12. ADMINISTRATIVE ASPECTS, MONITORING AND PUBLICATION

12.1 Handling and storage of data and documents

All data will be stored and will be coded. Only the researchers will be able to link patient ID and research code. The handling of personal data complies with the Dutch Personal Data Protection Act. After cessation of the study, patient material will be stored for a maximum of 15 years.

The case report forms and questionnaires will be filled in online. The head investigator will be able to check all the completed forms and questionnaires.

12.2 Monitoring and Quality Assurance

The monitoring will be coordinated by the Dutch Consortium and will be executed by a qualified intern monitor. This person is not involved in design and output of this research. The frequency of checking will be every year. The monitoring plan is discussed in section K of the METC dossier.

12.3 Amendments

All substantial amendments will be notified to the METC.

Non-substantial amendments will not be notified to the accredited METC, but will be recorded and filed by the sponsor.

12.4 Annual progress report

The sponsor/investigator will submit a summary of the progress of the trial to the accredited METC once a year. Information will be provided on the date of inclusion of the first subject, numbers of subjects included and numbers of subjects that have completed the trial, serious adverse events/ serious adverse reactions, other problems, and amendments.

12.5 End of study report

The investigator will notify the accredited METC of the end of the study within a period of 8 weeks. The end of the study is defined as the last patient's last visit.

In case the study is ended prematurely, the investigator will notify the accredited METC within 15 days, including the reasons for the premature termination.

For peer review only - http://bmjopen.bmj.com/site/about/guidelines.xhtml

Within one year after the end of the study, the investigator/sponsor will submit a final study report with the results of the study, including any publications/abstracts of the study,to the accredited METC.

12.6 Public disclosure and publication policy

The research findings will be published in peer reviewed journals.

For peer review only

For peer review only - http://bmjopen.bmj.com/site/about/guidelines.xhtml

13. STRUCTURED RISK ANALYSIS

Not applicable because this study is a low risk study concerning standard care.

13.1 Potential issues of concern

Not applicable.

13.2 Synthesis

Not applicable.

For peer review only

14. REFERENCES

1: Slieker-ten Hove MC et al. The prevalence of pelvic organ prolapse symptoms and signs and their relation with bladder and bowel disorders in a general female population. Int Urogynecol J. 2009;20(9):1037-45
2. Tegerstedt G, Maehle-Schmidt M, Nyrén O, Hammarström M. Prevalence of symptomatic pelvic organ prolapse in a Swedish population. Int Urogynecol J Pelvic Floor Dysfunct. 2005 Nov-Dec;16(6):497-503
3: Lamers BH et al. Pessary treatment for pelvic organ prolapse and health-related quality of life: a review. Int Urogynecol J. 2011;22(6):637-44
4: Broekman BM et al. Prospective pilot study in 65 women on pessary treatment as primary treatment, unpublished data
5:Broens-Oostveen WC, Mom RM, Lagro-Janssen ALM. De genitale prolaps: behnadleingen beloop in vier huisartspraktijken. Ned Tijdschr Geneeskd 2004, 148 (29); 1444-1448
6: Withagen M et al. Sacrospinous hysteropexy or modified manchester for the treatment of pelvic organ prolapse? Int J Gynaecol Obstet. 2013; Abstract Supplement
7: Vollebregt A et al. Cost-utility analysis of trocar-guided transobturator mesh versus anterior colporrhaphy for primary cystocele repair: a randomised trial. [in preparation]
8: Barber MD[1], Walters MD, Bump RC. Short forms of two condition-specific quality-of-life questionnaires for women with pelvic floor disorders (PFDI-20 and PFIQ-7). Am J Obstet Gynecol. 2005 Jul;193(1):103-13.
9: Pollard ME, Eilber KS, Anger JT. Abdominal approaches to pelvic prolapse repairs. Curr Opin Urol. 2013;23(4):306-11.
10: Dietz V et al. Vaginal surgery for uterine descent; which options do we have? A review of the literature. Int J Gynaecol Obstet. 2009;20(3):349-56
11: C. Maher, Feiner B, Baessler K, Schmid C. Surgical management of pelvic organprolapse in women. Cochrane 2013
12: de Boer TA, Milani AL, Kluivers KB, Withagen MI, Vierhout ME. The effectiveness of surgical correction of uterine prolapse: cervical amputation with uterosacral ligament plication (modified Manchester) versus vaginal hysterectomy with high uterosacral ligament plication. Int Urogynecol J Pelvic Floor Dysfunct. 2009 Nov;20(11):1313-9
13: Bugge C et al. Pessaries (mechanical devices) for pelvic organ prolapse in women. Cochrane database syst rev, 2013;28;2:CD004010
14: van Geelen JM, Dwyer PL. Where to for pelvic organ prolapse treatment after the FDA pronouncements? A systematic review of the recent literature. Int Urogynecol J. 2013 May;24(5):707-18
15: Abdool Z et al. Prospective evaluation of outcome of vaginal pessaries versus surgery in women with symptomatic pelvic organ prolapse. Int Urogynecol J. 2011;22(3):273-8
16: Hullfish KL, Trowbridge ER, Stukenborg GJ. Treatment strategies for pelvic organ prolapse: a cost-effectiveness analysis. Int Urogynecol J. 2011 May;22(5):507-15
17: Cundiff GW, Amundsen CL, Bent AE, Coates KW, Schaffer JI, Strohbehn K, Handa VL. The PESSRI study: symptom relief outcomes of a randomized crossover trial of the ring and Gellhorn pessaries. Am J Obstet Gynecol. 2007 Apr;196(4):405.e1-8
18: Weber MA, Limpens J, Roovers JPWR. Assessment of vaginal atrophy: a review [submitted BJOG]
19: Cobra Ok-Klapper 2012. DCHG 2012
20: Detollenaere RJ, den Boon J, Kluivers KB, Vierhout ME, van Eijndhoven HW.Surgical management of pelvic organ prolapse and uterine descent in the Netherlands. Int Urogynecol J. 2013 May;24(5):781-8
21.Detollenaere RJ, den Boon J, Vierhout ME, van Eijndhoven HW. Uterus preservingsurgery versus vaginal hysterectomy in treatment of uterine descent: a systematic review]. Ned

For peer review only - http://bmjopen.bmj.com/site/about/guidelines.xhtml

Tijdschr Geneeskd. 2011;155(42):A3623
22: Yalcin I, Bump RC. Validation of two global impression questionnaires for incontinence. Am J Obstet Gynecol. 2003 Jul;189(1):98-101
23: Barber MD, Kuchibhatla MN, Pieper CF, Bump RC. Psychometric evaluation of 2 comprehensive condition-specific quality of life instruments for women with pelvic floor disorders. Am J Obstet Gynecol 2001;185(6):1388–1395.
24: Utomo E, Blok BF, Steensma AB, Korfage IJ. Validation of the Pelvic Floor Distress Inventory (PFDI-20) and Pelvic Floor Impact Questionnaire (PFIQ-7) in a Dutch population. Int Urogynecol J. 2014;25(4):531-44.
25: Euroquol—a new facility for the measurement of health-related quality of life. The EuroQol Group. Health Policy. 1990;16:199-208
26: Rogers RG, Rockwood TH, Constantine ML, Thakar R, Kammerer-Doak DN, Pauls RN, Parekh M, Ridgeway B, Jha S, Pitkin J, Reid F, Sutherland SE, Lukacz ES, Domoney C, Sand P, Davila GW, Espuna Pons ME. A new measure of sexual function in women with pelvic floor disorders (PFD): the Pelvic Organ Prolapse/Incontinence Sexual Questionnaire, IUGA-Revised (PISQ-IR). Int Urogynecol J. 2013 Jul;24(7):1091-103
27: Cabana MD, Rand CS, Powe NR, Wu AW, Wilson MH, Abboud PA, Rubin HR. Why don't physicians follow clinical practice guidelines? A framework for improvement. JAMA. 1999 Oct 20;282(15):1458-65
28: Thys SD, Roovers JP, Geomini PM, Bongers MY. Do patients prefer a pessary or surgery as primary treatment for pelvic organ prolapse. Gynecol Obstet Invest. 2012;74(1):6-12
29: www.imta.nl (C. Bouwmans et al. Handleiding iMTA Medical Cost Questionnaire (iMCQ). Rotterdam: iMTA, Erasmus Universiteit Rotterdam, 2013)
30: www.imta.nl (C. Bouwmans et al. Handleiding iMTA Productivity Cost Questionnaire (iPCQ). Rotterdam: iMTA, Erasmus Universiteit, 2013)
31:Hakkaart- van Roijen, S.S. Tan, C.A.M. Bouwmans: Handleiding voor Kostenonderzoek. Methoden en standaard kostprijzen voor economische evaluaties in de gezondheidszorg. CVZ 2010
32: Oostenbrink JB, Koopmanschap MA, Rutten FF. Standardisation of costs: the Dutch Manual for Costing in economic evaluations. Pharmacoeconomics 2002;20(7):443-454
33: Mauskopf JA, Sullivan SD, Annemans L et al. Principles of good practice for budget impact analysis: report of the ISPOR Task Force on good research practices--budget impact analysis. Value Health 2007;10(5):336-347 -> In BIA, klopt niet, nog vervangen door:

References (Cost) effectiveness of pessary use as compared to surgery systematic review

1: Mamik MM, Rogers RG, Qualls CR, Komesu YM. Goal attainment after treatment in patients with symptomatic pelvic organ prolapse. Am J Obstet Gynecol. 2013 Nov;209(5):488.e1-5.
2: Abdool Z, Thakar R, Sultan AH, Oliver RS. Prospective evaluation of outcome of vaginal pessaries versus surgery in women with symptomatic pelvic organ prolapse. Int Urogynecol J. 2011 Mar;22(3):273-8.
3: Lowenstein L, Gamble T, Sanses TV, van Raalte H, Carberry C, Jakus S, Pham T, Nguyen A, Hoskey K, Kenton K; Fellow's Pelvic Research Network. Changes in sexual function after treatment for prolapse are related to the improvement in body image perception. J Sex Med. 2010 Feb;7(2 Pt 2):1023-8.
4: Barber MD, Walters MD, Cundiff GW; PESSRI Trial Group. Responsiveness of the Pelvic Floor Distress Inventory (PFDI) and Pelvic Floor Impact Questionnaire (PFIQ) in women undergoing vaginal surgery and pessary treatment for pelvic organ prolapse. Am J Obstet Gynecol. 2006 May;194(5):1492-8.

References Systematic review of factors influencing pessary fitting and continuation

1.Abdool Z, Thakar R, Sultan AH, Oliver RS (2010) Prospective evaluation of outcome of

For peer review only - http://bmjopen.bmj.com/site/about/guidelines.xhtml

vaginal pessaries versus surgery in women with symptomatic pelvic organ prolapse. Int Urogynecol J Pelvic Floor Dysfunct 16

2. Alperin M, Khan A, Dubina E, Tarnay C, Wu N, Pashos CL, Anger JT.Female Pelvic Med Reconstr Surg. 2013 May-Jun;19(3):142-7.Patterns of pessary care and outcomes for medicare beneficiaries with pelvic organ prolapse.
3. Bai SW, Yoon BS, Kwon JY, Shin JS, Kim SK, Park KH (2005) Survey of the characteristics and satisfaction degree of the patients using a pessary. Int Urogynecol J Pelvic Floor Dysfunct 16 (3):182–186
4. Barber MD, Walters MD, Cundiff GW (2006) Responsiveness of the Pelvic Floor Distress Inventory (PFDI) and Pelvic Floor Impact Questionnaire (PFIQ) in women undergoing vaginal surgery and pessary treatment for pelvic organ prolapse. Am JObstet Gynecol 194(5):1492–149
5. Brincat C, Kenton K, Pat FM, Brubaker L (2004) Sexual activity predicts continued pessary use. Am J Obstet Gynecol 191(1):198–200
6. Broens-Oostveen MC, Mom RM, Lagro-Janssen AL (2004)Genital prolapse; treatment and course in four general practices.Ned Tijdschr Geneeskd 148(29):1444–1448, 17
7. Chan SS, Cheung RY, Yiu KW, Lee LL, Pang AW, Chung TK. Int Urogynecol J. 2012 Aug;23(8):1027-33. Symptoms, quality of life, and factors affecting women's treatment decisions regarding pelvic organ prolapse.
8. Clemons JL, Aguilar VC, Sokol ER, Jackson ND, Myers DL (2004) Patient characteristics that are associated with continued pessary use versus surgery after 1 year. Am J Obstet Gynecol 191(1):159–164
9. Cundiff GW, Amundsen CL, Bent AE, Coates KW, Schaffer JI, Strohbehn K et al (2007) The PESSRI study: symptom relief outcomes of a randomized crossover trial of the ring and Gellhorn pessaries. Am J Obstet Gynecol 196(4):405–408
10. Fernando RJ, Thakar R, Sultan AH, Shah SM, Jones PW (2006) Effect of vaginal pessaries on symptoms associated with pelvic organ prolapse. Obstet Gynecol 108(1):93–99
11. Friedman S, Sandhu KS, Wang C, Mikhail MS, Banks E (2010) Factors influencing long-term pessary use. Int Urogynecol J Pelvic Floor Dysfunct 21(6):673–678
12. Hanson LA, Schulz JA, Flood CG, Cooley B, Tam F (2006) Vaginal pessaries in managing women with pelvic organ prolapse and urinary incontinence: patient characteristics and factors contributing to success. Int Urogynecol J Pelvic Floor Dysfunct 17(2):155–159
13. Jones K,Yang L, Lowder JL, Meyn L, Ellison R, ZyczynskiHMet al (2008) Effect of pessary use on genital hiatus measurements in women with pelvic organ prolapse. Obstet Gynecol 112(3):630–636
14. Geoffrion R, Zhang T, Lee T, Cundiff GW.Female Pelvic Med Reconstr Surg. 2013 Nov-Dec;19(6):339-45. Clinical characteristics associated with unsuccessful pessary fitting outcomes.
15. Komesu YM, Rogers RG, Rode MA, Craig EC, Gallegos KA, Montoya AR et al (2007) Pelvic floor symptom changes incpessary users. Am J Obstet Gynecol 197(6):620–626
16. Kuhn A, Bapst D, Stadlmayr W, Vits K, Mueller MD (2009) Sexual and organ function in patients with symptomatic prolapse: are pessaries helpful? Fertil Steril 91(5):1914–1918
17. Lone F1, Thakar R, Sultan AH, Karamalis G. A 5-year prospective study of vaginal pessary use for pelvic organ prolapse. Int J Gynaecol Obstet. 2011 Jul;114(1):56-9.
18. Maito JM, Quam ZA, Craig E, Danner KA, Rogers RG (2006) Predictors of successful pessary fitting and continued use in a nurse-midwifery pessary clinic. J Midwifery Womens Health 51 (2):78–84
19. Mamik MM, Rogers RG, Qualls CR, Komesu YM. Goal attainment after treatment in patients with symptomatic pelvic organ prolapse. Am J Obstet Gynecol. 2013 Nov;209(5):488.e1-5.
20. Manchana T, Bunyavejchevin S. Impact on quality of life after ring pessary use for pelvic organ prolapse. Int Urogynecol J. 2012 Jul;23(7):873-7.
21. Markle D, Skoczylas L, Goldsmith C, Noblett K. Patient characteristics associated with a

For peer review only - http://bmjopen.bmj.com/site/about/guidelines.xhtml

successful pessary fitting. Female Pelvic Med Reconstr Surg. 2011 Sep;17(5):249-52.
22. Mokrzycki ML, Hatangadi SB, Zaccardi JE, Cox S. Preexisting stress urinary incontinence: a predictor of discontinuation with pessary management. J Low Genit TractDis. 2001 Oct;5(4):204-7.
23. Mutone MF, Terry C, Hale DS, Benson JT (2005) Factors which influence the short-term success of pessary management of pelvic organ prolapse. Am J Obstet Gynecol 193(1):89–94
24. Nguyen JN, Jones CR. Pessary treatment of pelvic relaxation: factors affecting successful fitting and continued use. J Wound Ostomy Continence Nurs. 2005;32(4):255-61; quiz 262-3.
25. Patel M, Mellen C, O'Sullivan DM, Lasala CA. (2010) Impact ofpessary use on prolapse symptoms, quality of life, and bodyimage. Am J Obstet Gynecol 18
26. Powers K, Lazarou G, Wang A, LaCombe J, Bensinger G.Greston WM et al (2006) Pessary use in advanced pelvic organprolapse. Int Urogynecol J Pelvic Floor Dysfunct17(2):160–164.

Reference list Systematic review of factors influencing failure of POP surgery

1. Whiteside JL, Weber AM, Meyn LA, Walters MD. Am J Obstet Gynecol. 2004 Nov;191(5):1533-8.factors for prolapse recurrence after vaginal repair.
2. Diez-Itza I, Aizpitarte I, Becerro A. Int Urogynecol J Pelvic Floor Dysfunct. 2007 Nov;18(11):1317-24. Risk factors for the recurrence of pelvic organ prolapse after vaginal surgery: a review at 5 years after surgery.
3. Fialkow MF, Newton KM, Weiss NS. Int Urogynecol J Pelvic Floor Dysfunct. 2008 Nov;19(11):1483-7.Incidence of recurrent pelvic organ prolapse 10 years following primary surgical management: a retrospective cohort study.
4. Jeon MJ, Chung SM, Jung HJ, Kim SK, Bai SW. Gynecol Obstet Invest. 2008;66(4):268- 73 Risk factors for the recurrence of pelvic organ prolapse.
5. Dällenbach P, Jungo Nancoz C, Eperon I, Dubuisson JB, Boulvain M. Int Urogynecol J. 2012 Jan;23(1):35-41. Incidence and risk factors for reoperation of surgically treated pelvic organ prolapse.
6. Weemhoff M, Vergeldt TF, Notten K, Serroyen J, Kampschoer PH, Roumen FJ. Int Urogynecol J. 2012 Jan;23(1):65-71. Avulsion of puborectalis muscle and other risk factors for cystocele recurrence: a 2-year follow-up study.
7. Tegerstedt G1, Hammarström M. Acta Obstet Gynecol Scand. 2004 Aug;83(8):758-63. Operation for pelvic organ prolapse: a follow-up study.
8. Medina CA, Candiotti K, Takacs P. Int J Gynaecol Obstet. 2008 May;101(2):184-7. Wide genital hiatus is a risk factor for recurrence following anterior vaginal repair.
9. Salvatore S1, Athanasiou S, Digesu GA, Soligo M, Sotiropoulou M, Serati M, Antsaklis A, Milani R. Neurourol Urodyn. 2009;28(4):301-4. doi: 10.1002/nau.20639. Identification of risk factors for genital prolapse recurrence.
10. Kawasaki A, Corey EG, Laskey RA, Weidner AC, Siddiqui NY, Wu JM. J Reprod Med. 2013 May-Jun;58(5-6):195-9. Obesity as a risk for the recurrence of anterior vaginal wall prolaps after anterior colporrhaphy.

For peer review only - http://bmjopen.bmj.com/site/about/guidelines.xhtml

Appendix 1:

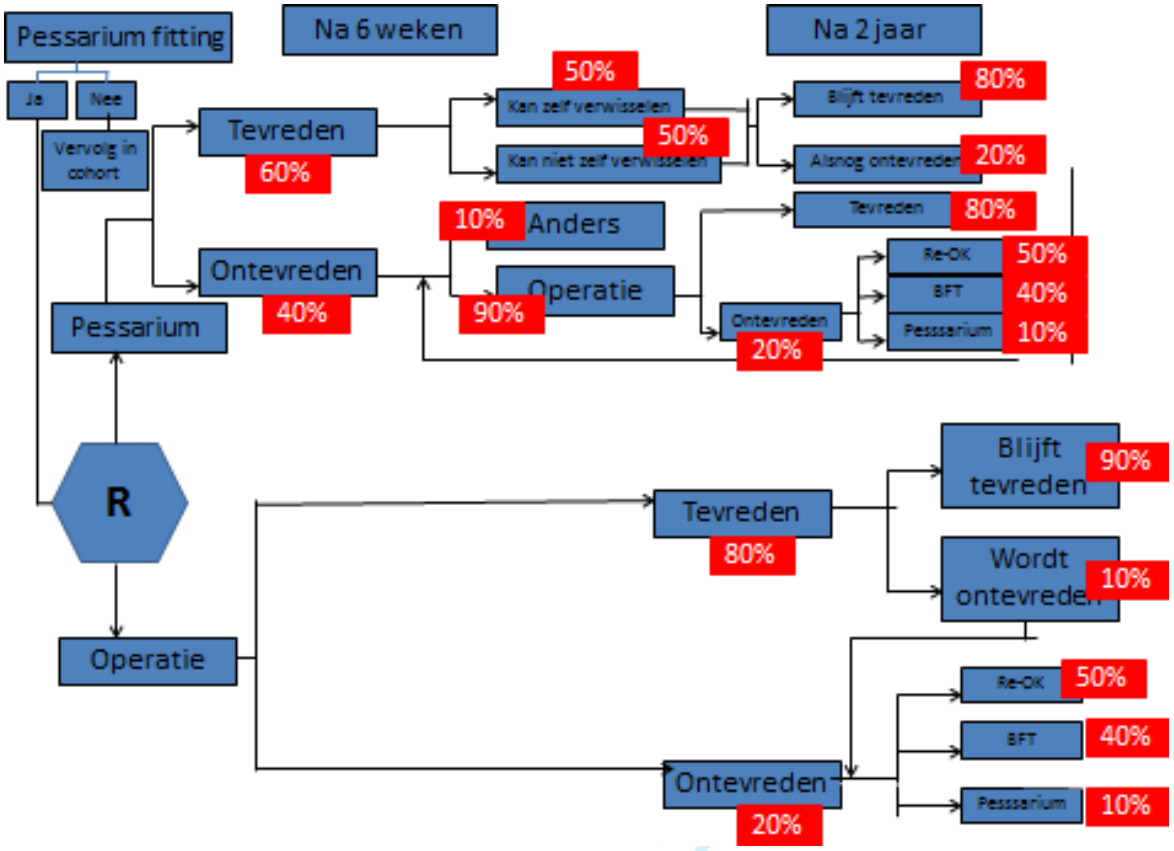

w only

For peer review only - http://bmjopen.bmj.com/site/about/guidelines.xhtml

Appendix 2 Review on (Cost) effectiveness of pessary use as compared to surgery:

Reference	Study type	Characteristics	Intervention (I)	Controls (C)	Outcome measures and follow-uptime	Results
Mamik, 2012<br><br>AJOG 2013:209:488	Design<br>Case-control<br>N = 100<br>Country<br>US	Aim: compare goal achievement and global improvement between pessary and surgery for [illegible] stage ≥2.<br><br>Inclusion criteria: >18 year old, read and write in English<br><br>Exclusion: not given	Vaginal pessary<br>N = 50	Prolapse surgery<br>N = 50	Primary outcome:<br>Goal attainment<br><br>Secondary:<br>PGI-I<br>PFDI-20<br>PISQ-12<br>Body Image scale<br><br>Follow-up: 3 months	Primary outcome:<br>Goal attainment sign. higher score after surgery (8.6 vs 6.4)<br>Secondary outcomes<br>PGI-I sign (p=0.04) better improvement after surgery (2.4vs 1.9 points)<br>PFDI-20 sign (p=0.02) higher change (89 vs 43 points)<br>PISQ-12 and BIS no sign difference<br><br>Additional: 10% crossed over from pessary to surgery within 3 months [illegible] referred from surgery after they had been selected as eligible.<br>No follow-up in pessary group is 40% (20/50) and surgery 30% [illegible]
Abdool, 2011	Design<br>Cohort study<br><br>N total = [illegible]<br><br>Country:<br>UK	Aim of the study<br>to evaluate and compare the effectiveness of pessariesand surgery in women with symptomatic pelvic organ prolapse.<br>Inclusion criteria<br>- Women referred to a specialist urogynaecology unit with symptomatic [illegible] between June 2002 andMay 2007	Intervention vaginal pessary<br>N = 359	Controls surgery<br>N = 195	Primary outcomes:<br>Sheffield POP questionnaire [illegible]<br><br>Secundary outcomes:<br>None<br>Follow up:<br>For the surgery and pessary groups 14 months(SD 6.14) and 12 months (SD 3.1), respectively.	Primary outcomes:<br>No difference in functional outcome after 1 year follow-up between groups<br>Additional:<br>Only 45% in pessary group en 55% in surgery group responded at 12monthsIn pessary group 24.7% (89/359) crossed to surgery but were not analyzed In pessary group 7.3% stopped because of other reasons.<br>Selection and patient preference bias<br>The mean age was significantly higher in the pessary group compared to the surgerygroup (68.4 +/− 13.08 vs 60.4 +/−12.25 years, respectively).

For peer review only - http://bmjopen.bmj.com/site/about/guidelines.xhtml

For peer review only

Study protocol

		**Exclusion criteria**<br>· Subjects fitted with pessaries for urinary incontinence and those who had concomitant urinary incontinence surgery (e.g. TVT)<br>· Subjects who started inthe pessary group but subsequently requested surgery were excluded from analysis in both the surgery and pessary group.				

For peer review only

Study protocol

**Lowenstein 2010**<br><br>**J Sex Med 2010; 7:-1023**<br><br>**28**	**Design**<br>**Cohort study**<br>**N= 235**<br><br>**Country**<br>**US**	**Aim of the study**<br>**First to evaluate patient-reported outcome, POP symptoms, sexual functioning and body image following treatment of PO**<br>**Second to compare surgery with pessary**<br><br>**Inclusion: ≥18 year, ≥ satge 2 POP, complete questionnaire at baseline and at ≥6 months follow up**<br><br>**Exclusion: recurrent UTI, peripheral neuropathy, using pessary at initial presentationor POP surgery < 6 months prior to presentation**	**Intervention**<br>**N = 202 surgery**	**Controls**<br>**N = 33 pessary**	**Primary outcomes**<br>**PFDI-20**<br>**PISQ-12**<br>**Modified Body Image scale**<br>**All at six months follow-up**	**Results**<br>**After multivariate analyses, including type of intervention, BMI and differencein Body image were associated with change in total PISQ (sexual functioning) score**<br><br>**In the pessary group there was no significant improvement in sexual functioningas compared to surgery (-2.5 versus +11.5)**<br>**Additional:**<br>**No figures presented for pessary and surgery group, with exemption oftheSexual functioning (PISQ-12) result above.**

For peer review only - http://bmjopen.bmj.com/site/about/guidelines.xhtml

Study protocol

**Barber, 2006**	**Design** Case-control study **Ntotal=106Country:** USA	Aim of the studyto evaluate the responsiveness of the PelvicFloor Distress Inventory (PFDI) andelicvPFloor Impact Questionnaire (PFIQ) in women with pelvic organ prolapse undergoingsurgical and nonsurgical management.<br><br>Inclusion criteria<br>Surgery group:<br>Stage III or IV prolapse, wereat least 18 years, and scheduled for vaginal prolapse repair.<br>Pessary group:<br>women with symptomatic pelvic organ prolapse ofstageII or greater. (Pessri trial)<br><br>Exclusion criteriaSurgery group:<br>· mentally or physically incapable of completingthe questionnaires.<br>Pessary group:<br>· were pregnant, were currently using a pessary, orhad vaginal agglutination that precluded pessary insertion.	**Intervention Pessary in women with stage II or greater POP N = 42**	**Controls Surgery in womenwithstage III or greater POP N = 64**	**Primary outcomes: PFDI and PFIQ**<br>**Secundary outcomes:**<br><br>**Follow up:**<br>**3 months (Pessary group)or 6 months (Surgery group) after initiationoftreatment.**	**Primary outcomes:**<br><br>**After controlling for preoperative prolapse stage and baseline HRQOLscores,subjects in the Surgery group had significantly greater improvement in each of the scales of the PFDI and the prolapse and urinary scales of the PFIQ than did the Pessary group.**<br>**Scores from each of the scales of the PFDI improved by 14 to 15 points more on average after treatment in the Surgery group than those ofthe Pessary group (P < .01 for each) after adjusting for the above baseline differences.**<br><br>**Similarly, for the prolapse and urinary scales of the PFIQ, scores improved13and 17 points more, respectively, in the Surgery group than the Pessary group after treatment. (P < .05 for each).**<br><br>**Four of 64 (6%) of subjects in the Surgery group had recurrent prolapsedevelopbeyond the hymen by 6 months after surgery. No subjects underwent reoperation for recurrent prolapse during the study period.**<br>**Additional:**<br><br>**Difference in followup**<br>**Selection bias**

For peer review only - http://bmjopen.bmj.com/site/about/guidelines.xhtml

Appendix 3 Review on risk factors for failure of pessaries:

study		n	FU period	success initial	continued pes	stopped pes	cross over surgery	predict fit	predict contin
Wu 1997	retrospective	110	3 yrs	81 (74%)	43 (39%)		at least 5	nee	ja
Mokrzycki 2001	retrospective	42	3 mth		24 (57%)	18 (43%)	nr	nee	ja
Clemons 2004	prospective	100	1 yr	73 (73%)	43 (43%)		16/73 (27%)	nee	ja
Brincat 2004	retrospective	169	2,5 yr	144 (85%)	82 (60%)	54 (40%)	32/144 (22%)	nee	ja
Mutone 2004	retrospective	407	3 wks	168 (41%)		96 (57%)	nr	ja	ja
Powers 2006	retrospective	32	1 yr			20 (62%)	12 (38%)	nee	ja
Broens-Oostveen 2004	retrospective	192	9 yr			107 (52%)	nr	nee	ja
Nguyen 2005	retrospective	130	1 yr	111 (82%)	85 (65%)		20 (15%)	ja	ja
Hanson 2005	retrospective	1216	3 mth	1043 (86%)	744 (71%)		nr	nee	ja
Fernando 2006	prospective	203	4 mth	nr	153 (75%)		28 (14%)	nee	ja
Barber 2006	prospective	42	3 mth	nr		0	nr	nee	nee
Maito 2006	retrospective	120	6 mth	106 (88%)	92 (77%)		9 (8%)	ja	ja
Cundiff 2007	prospective	85	1 yr	65/71 (92%)			5 (6%)	nee	nee
Komesu 2007	prospective	64	6-12 mth		36 (56%)	28 (44%)	2 (3%)	nee	ja
Jones 2008	prospective	90	3 mth		42 (47%)		nr	nee	ja
Kuhn 2009	prospective	71	3 mth		32 (45%)		nr	nee	nee
Sarma 2009	retrospective	120	1 mth		45 (38%)		18 (15%)	nee	nee
Friedman 2010	retrospective	150	1 yr		115 (77%)	35 (23%)	25 (17%)	nee	ja
Patel 2010	prospective	65	3 mth	54 (83%)	54 (83%)		5 (8%)	nee	nee
Abdool 2010	prospective	359	1 yr	296 (83%)	243 (68%)		31 (9%)	nee	nee
Lone 2011	prospective	246	5 yr	187 (76%)	130 (53%)		70 (28%)	nee	nee
Markle 2011	retrospective	158	1 week	92 (59%)			nr	ja	nee
Chan 2012	prospective	197	1 yr	138 (70%)		59 (30%)	nr	nee	ja
Manchana 2012	retrospective	126	1 yr	77 (61%)	64 (51%)		5 (4%)	nee	nee
Mamik 2013	prospective	50	3 mth				5 (10%)	nee	nee
Geoffrion 2013	retrospective	101	4 wks	79 (78%)			nr	nee	ja
Alperin 2013	retrospective	4019	9 years				12% 1 yr, 24% 9 yrs	nee	nee
Succesfull fitting		3589		2714(76%)					
Continuation		3381			2027 (60%)				

For peer review only - http://bmjopen.bmj.com/site/about/guidelines.xhtml

Appendix 4 Review on risk factors for failure of surgery:

Risk factor	Investigated:	Significant:
Preoperative stage	8	5
Age	8	2
Obesity	7	0
Parity	5	0
Constipation	5	0
Pulmonary disease	5	0
Number of sites involved preoperative	4	1
Menopausal status	4	0
Hysterectomy status	4	0
Concomitent surgery	3	1
Family history	3	1
Complicated delivery	3	0
Diabetes	3	0
Smoking	3	0
Previous incontinence and/or prolapse surgery	2	2
Hiatus genitalis	2	1
Weight	2	1
Any incontinence preoperative	2	1
Delivery mode	2	0
Vaginal delivery	2	0
Hormone replacement therapy	2	0
Previous prolapse surgery	2	0
Surgeons experience	2	0
Abcense of posterior repair	1	1
Sexual activity	1	1
Levator defect	1	1
Height	1	0
Birth weight	1	0
Age at last delivery	1	0
Site of most advanced prolapse	1	0
Surgical approach	1	0
Use of Mesh	1	0
Previous incontinence surgery	1	0
Previous pelvic floor surgery or hysterectomy	1	0
Abdominal hernias	1	0
Cardiovascular disease	1	0
Intense physical exercise	1	0
Heavy lifting	1	0
Heavy lifting or constipation	1	0
Levator muscle contraction	1	0
Weight of the uterus	1	0
Postoperative complications	1	0
Incomplete emptying of bladder	1	0
Fecal incontinence	1	0

For peer review only

For peer review only - http://bmjopen.bmj.com/site/about/guidelines.xhtml

Appendix 5 diagram/tabel bezoeken, tijdstippen, onderzoeken

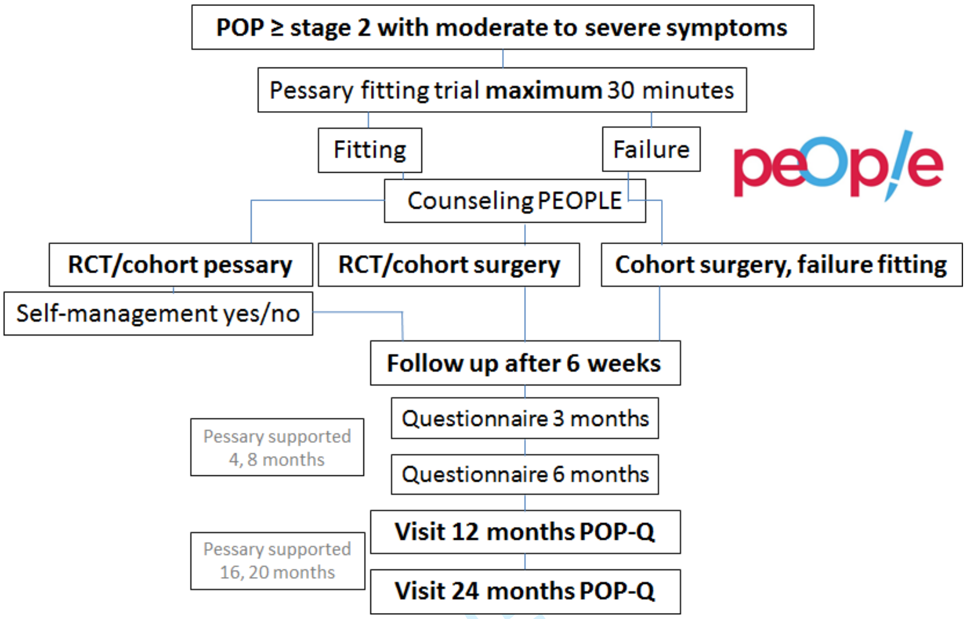

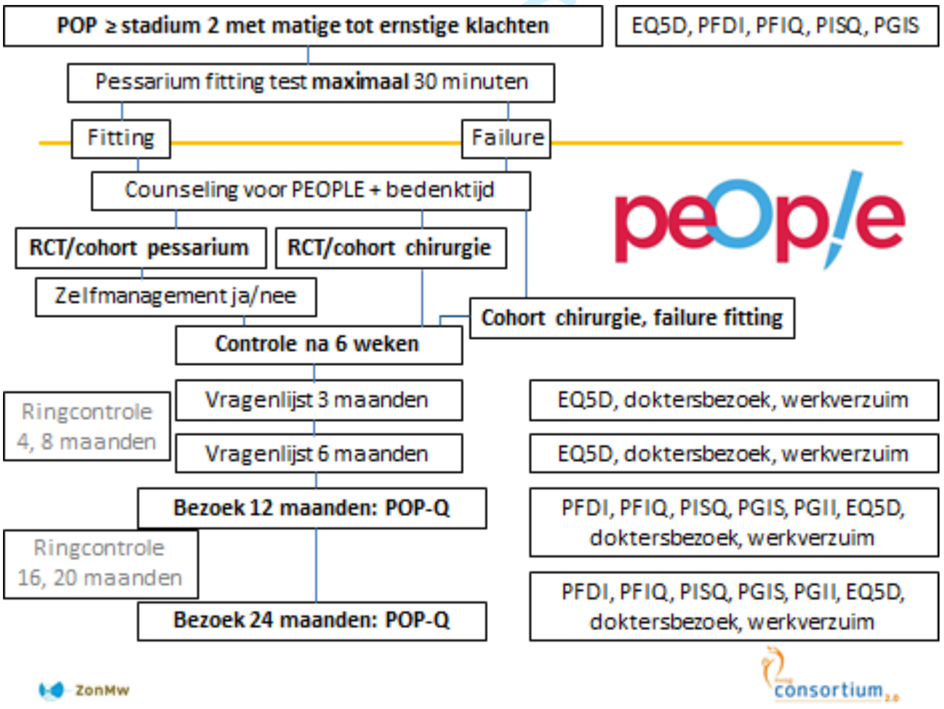

Chirurgie en cohort fitting failure

Contact	Bezoek arts	POPQ	Eq5d<br>doktersbezoek<br>werkverzuim	PFIQ<br>PFDI<br>PISQ<br>PGII<br>PGIS

1. Eerste bezoek	X	X	Eq5D	X (zonder PGII)
2. 6 weken	X			
3. 3 maanden			X	
4. 6 maanden			X	
5. 12 maanden	X	X	X	X
6. 24 maanden	X	X	X	X

Pessarium met zelfmanagement

Contact	Bezoek arts	POPQ	Eq5d doktersbezoek werkverzuim	PFIQ PFDI PISQ PGII PGIS
1. Eerste bezoek	X	X	Eq5D	X (zonder PGII)
2. 6 weken	X			
3. 3 maanden			X	
4. 6 maanden			X	
5. 12 maanden	X	X	X	X
6. 24 maanden	X	X	X	X

For peer review only

Pessarium zonder zelfmanagement

Contact	Bezoek arts	POPQ	Ringcontrole	Eq5d doktersbezoek werkverzuim	PFIQ PFDI PISQ PGII PGIS
1. Eerste bezoek	X	X		Eq5D	X (zonder PGII)
2. 6 weken	X				
3. 3 maanden				X	
4. 4 maanden	X		X		
5. 6 maanden				X	
6. 8 maanden	X		X		
7. 12 maanden	X	X	X	X	X
8. 16 maanden	X		X		
9. 20 maanden	X		X		
10. 24 maanden	X	X	X	X	X

For peer review only

1.3 Summary of amendment to study protocol

The main change in the final version is the addition of an observational cohort performed alongside the RCT. We added this observational cohort since many women refused to participate in the RCT due to treatment preference. In case a woman was willing to participate in the study but actively opted for one of two treatment options she was followed in the observational cohort. The same study parameters and follow-up were used in both the trial and observational cohort. See section 2, section 3, section 4.4, section 8.2, section 10, section 11.2

1.3.1 Detailed summary of all amendments

1. Addition of multiple centers for participation.

Added centers:

- Atrium MC Heerlen
- Academisch ziekenhuis Maastricht
- Martini ziekenhuis Groningen
- MST Enschede
- ZGT Almelo / Hengelo
- Deventer ziekenhuis
- Jeroen Bosch ziekenhuis
- Amstelland ziekenhuis
- Tergooi ziekenhuis
- Albert Schweitzer ziekenhuis
- Canisius Wilhelmina ziekenhuis
- Maxima Medisch Centrum
- MCH-Bronovo
- OLVG
- HAGA

2. Change in investigators at the following participating centers:
    - St. Antonius hospital. S. The was replaced by E. Vernooij
    - Canisius hospital. C.F. van Heteren was replaced by K.L. Bos
    - Maastricht University center (MUMC): G. Link was replaced by W.A. Spaans

3. Change in Head of Department of Reproductive Medicine and Gynaecology.

4. Change in Objective.

An observational cohort was added since many women refused to participate in the trial due to treatment preference. At first, women were asked to participate in the trial. In case the woman is willing to participate but actively opts for one of both treatments, she will be followed in a cohort ‘own choice’.

For peer review only - http://bmjopen.bmj.com/site/about/guidelines.xhtml

5. Change in study design.

In the first version it is noted that for women with an unsuccessful pessary fitting only baseline characteristics will be recorded. However, these women will be followed in the cohort fitting failure with the same follow-up as for the trial (24-months). Appendix 5 has been noted in more detail.

6. Addition in sample size calculation for observational cohort.

Since we added an observational cohort with women who made their own choice of treatment, we added this to the section sample size calculation. In the cohort we include all patients who are willing to collaborate on this research but have a preference for one of both therapies. We now assume that 70% of the eligible patients object participation in the RCT, and that 90% of them is nevertheless willing to participate in the cohort.

7. Change in self-management of pessary treatment.

In case self-management was performed, women were advised to change their pessary every 4-months, instead of every 1 month.

8. Observational cohort is added in randomization section.

In case the woman is willing to participate but actively opts for one of both treatments, she will also be registered in ALEA. All groups will have the same data collection and follow up as displayed in appendix 5.

9. Observational cohort added in statistical analysis section.

The cohort with patients treated according their preference will be analyzed separately from the randomized trial. The same analysis will be done.

10. Change in exclusion criteria.

Women with a previous hysterectomy were only excluded in case the indication for the hysterectomy was a prolapse.

11. Observational cohort added in recruitment.

In case the woman is willing to participate but actively opts for one of both treatments, she can attend the cohort.

12. Change in monitoring

At first, the monitoring was coordinated by the Staff Member Clinical Research, quality coordinator of division women and baby. Later on, the monitoring was conducted by the Dutch consortium and was executed by a qualified intern monitor.

13. POP-Q only performed at 12- and 24-months follow-up, not at 6 weeks visit.

Demonstrated in the tables listed in appendix 5.

For peer review only - http://bmjopen.bmj.com/site/about/guidelines.xhtml

1.3.2 Table with amendments and corresponding section

Amendment	Corresponding section in the final version 1.22
1. Addition of multiple centers for participation	First table with project information
2. Change in investigators	First table with project information
3. Change in Head of Department	Protocol signature sheet
4. Change in objective	Section 2
5. Change in study design	Section 3
6. Addition in sample size calculation for observational cohort	Section 4.4
7. Change in self-management of pessary treatment	Section 5.1
8. Observational cohort is added in randomization section	Section 8.2
9. Observational cohort added in statistical analysis section	Section 10
10. Change in exclusion criteria	Section 10.2
11. Observational cohort added in recruitment	Section 11.2
12. Change in monitoring	Section 12.2
13. POP-Q only performed at 12- and 24-months	Appendix 5

For peer review only

For peer review only - http://bmjopen.bmj.com/site/about/guidelines.xhtml

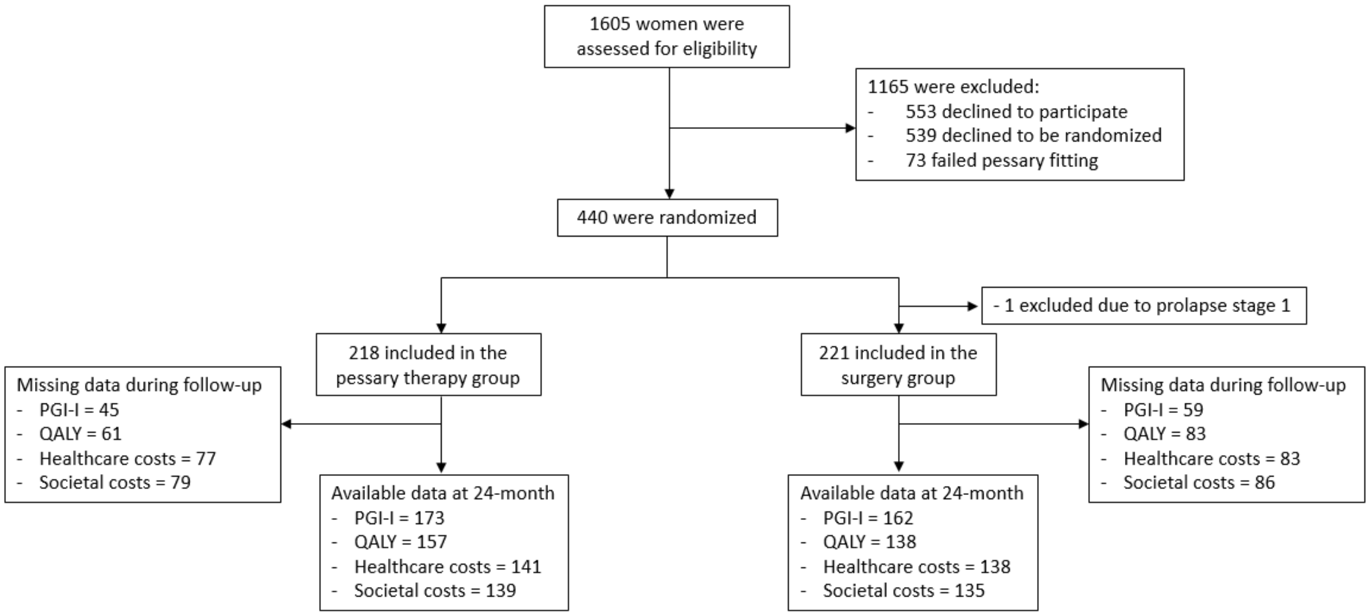

SUPPLEMENTARY Figure 1. FLOW DIAGRAM. Inclusion and available data at 24-month follow-up.

SUPPLEMENTARY TABLE 1. RESOURCES AND UNIT COSTS

Resources	Unit costs	Year	Reference
Pessary device			
Milex®	€64	2022	Market price: bol.com
Arabin®	€73	2022	Market price: bol.com
Other brand (average)	€68	2022	Market price: bol.com
Pessary placement	€109	2022	Dutch costing manual[1]
Surgery			
Sacrospinous hysteropexy (care product 149999033)	€5835	2022	DBC[2]
Sacrospinous fixation (care product 149999047)	€4640	2022	DBC[2]
Manchester–Fothergill procedure (care product 149999047)	€4640	2022	DBC[2]
Abdominal sacrocolpopexy (care product 149999033)	€5835	2022	DBC[2]
Sacrocervicopexy care product 149999033)	€5835	2022	DBC[2]
Vaginal hysterectomy (care product 149999047)	€4640	2022	DBC[2]
Average surgical procedures costs (used as WTP threshold)	€5237	2022	DBC[2]
**Other resources**			
General practitioner consultation	€39	2022	Dutch costing manual[1]
Other healthcare professional consultation at primary care	€39	2022	Dutch costing manual[1]
Medical specialist consultation at secondary care	€109	2022	Dutch costing manual[1]
Hospital readmission (1 day)	€568	2022	Dutch costing manual[1]
Paid working hour for women	€38	2022	Dutch costing manual[1]

DBC: Diagnosis Treatment Combination, in Dutch *Diagnose Behandeling CombinatieI.*

References:

1 Kanters TA, Bouwmans CAM, van der Linden N, *et al.* Update of the Dutch manual for costing studies in health care. *PLoS One* 2017;**12**. doi:10.1371/journal.pone.0187477

2 Diagnose Behandeling Combinatie (DBC) open data - NZa. https://www.opendisdata.nl/ (accessed 3 Sep 2022).

For peer review only

CHEERS 2022 Checklist

Topic	No.	Item	Location where item is reported
**Title**			
	1	Identify the study as an economic evaluation and specify the interventions being compared.	Page 1, 1st paragraph
**Abstract**			
	2	Provide a structured summary that highlights context, key methods, results, and alternative analyses.	Page 2
**Introduction**			
**Background and objectives**	3	Give the context for the study, the study question, and its practical relevance for decision making in policy or practice.	Page 4
**Methods**			
**Health economic analysis plan**	4	Indicate whether a health economic analysis plan was developed and where available.	Page 5, Study design, 1st paragraph
**Study population**	5	Describe characteristics of the study population (such as age range, demographics, socioeconomic, or clinical characteristics).	Page 5, Study population, 2nd paragraph
**Setting and location**	6	Provide relevant contextual information that may influence findings.	Page 6, Setting and location, 1st paragraph
**Comparators**	7	Describe the interventions or strategies being compared and why chosen.	Page 6, Comparators, Pessary therapy, 2nd paragraph and Surgery, 3rd paragraph
**Perspective**	8	State the perspective(s) adopted by the study and why chosen.	Page 7, Study perspective, time horizon, and discount rate, 1st paragraph
**Time horizon**	9	State the time horizon for the study and why appropriate.	Page 7, Study perspective, time horizon, and discount rate, 1st paragraph

Topic	No.	Item	Location where item is reported
**Discount rate**	10	Report the discount rate(s) and reason chosen.	Page 7, Study perspective, time horizon, and discount rate, 1st paragraph
**Selection of outcomes**	11	Describe what outcomes were used as the measure(s) of benefit(s) and harm(s).	Page 7, Outcomes, Health outcomes, 2nd and 3rd paragraph
**Measurement of outcomes**	12	Describe how outcomes used to capture benefit(s) and harm(s) were measured.	Page 7, Outcomes, Health outcomes, 2nd and 3rd paragraph
**Valuation of outcomes**	13	Describe the population and methods used to measure and value outcomes.	Page 7, Outcomes, Health outcomes, 2nd and 3rd paragraph
**Measurement and valuation of resources and costs**	14	Describe how costs were valued.	Page 8-9, Cost outcomes
**Currency, price date, and conversion**	15	Report the dates of the estimated resource quantities and unit costs, plus the currency and year of conversion.	Page 9, 1st paragraph, last sentence
**Rationale and description of model**	16	If modelling is used, describe in detail and why used. Report if the model is publicly available and where it can be accessed.	Page 9, Cost-effectiveness analysis, 3rd paragraph
**Analytics and assumptions**	17	Describe any methods for analysing or statistically transforming data, any extrapolation methods, and approaches for validating any model used.	Page 9-10, Cost-effectiveness analysis
**Characterising heterogeneity**	18	Describe any methods used for estimating how the results of the study vary for subgroups.	Page 10, Sensitivity analysis
**Characterising distributional effects**	19	Describe how impacts are distributed across different individuals or adjustments made to reflect priority populations.	Page 9, 3rd paragraph and Page 10, 1st paragraph
**Characterising uncertainty**	20	Describe methods to characterise any sources of uncertainty in the analysis.	Page 10, Sensitivity analysis
**Approach to engagement with patients and others affected by the study**	21	Describe any approaches to engage patients or service recipients, the general public, communities, or stakeholders (such as clinicians or payers) in the design of the study.	Page 11, Patient and Public Involvement

For peer review only - http://bmjopen.bmj.com/site/about/guidelines.xhtml

Topic	No.	Item	Location where item is reported
**Results**			
**Study parameters**	22	Report all analytic inputs (such as values, ranges, references) including uncertainty or distributional assumptions.	Page 12, Participants, 1st paragraph
**Summary of main results**	23	Report the mean values for the main categories of costs and outcomes of interest and summarise them in the most appropriate overall measure.	Page 12-13, Effectiveness, Costs
**Effect of uncertainty**	24	Describe how uncertainty about analytic judgments, inputs, or projections affect findings. Report the effect of choice of discount rate and time horizon, if applicable.	Page 13, Cost-effectiveness analysis. Page 14, Sensitivity analysis
**Effect of engagement with patients and others affected by the study**	25	Report on any difference patient/service recipient, general public, community, or stakeholder involvement made to the approach or findings of the study	Not Applicable
**Discussion**			
**Study findings, limitations, generalisability, and current knowledge**	26	Report key findings, limitations, ethical or equity considerations not captured, and how these could affect patients, policy, or practice.	Page 15-17
**Other relevant information**			
**Source of funding**	27	Describe how the study was funded and any role of the funder in the identification, design, conduct, and reporting of the analysis	Page 3
**Conflicts of interest**	28	Report authors conflicts of interest according to journal or International Committee of Medical Journal Editors requirements.	Page 3

From: Husereau D, Drummond M, Augustovski F, et al. Consolidated Health Economic Evaluation Reporting Standards 2022 (CHEERS 2022) Explanation and Elaboration: A Report of the ISPOR CHEERS II Good Practices Task Force. Value Health 2022;25. doi:10.1016/j.jval.2021.10.008

BMJ Open

Cost-effectiveness of pessary therapy versus surgery for symptomatic pelvic organ prolapse: an economic evaluation alongside a randomized non-inferiority controlled trial

Journal:	*BMJ Open*
Manuscript ID	bmjopen-2023-075016.R2
Article Type:	Original research
Date Submitted by the Author:	13-Mar-2024
Complete List of Authors:	Ben, Ângela; Vrije Universiteit Amsterdam, Department of Health Sciences<br>van der Vaart, Lisa R.; University of Amsterdam, Department of Obstetrics and Gynaecology, Amsterdam Reproduction & Development Research Institute<br>Bosmans, J; VU University Amsterdam, Department of Health Sciences<br>Roovers, Jan-Paul W.R.; University of Amsterdam, Department of Obstetrics and Gynecology; Women's Health Bergman Clinics, Department of Gynecology<br>Lagro-Janssen, Antoinette; Radboud university medical center, Department of Primary and Community Care<br>van der Vaart, Carl H.; University Medical Center Utrecht, Department of Obstetrics and Gynecology; Women's Health Bergman Clinics, Department of Gynecology<br>Vollebregt, Astrid; Spaarne Hospital, Department of Obstetrics and Gynecology
**Primary Subject Heading**:	Health economics
Secondary Subject Heading:	Obstetrics and gynaecology
Keywords:	Pelvic Pain, Urogynaecology < GYNAECOLOGY, Health economics < HEALTH SERVICES ADMINISTRATION & MANAGEMENT

SCHOLARONE™
Manuscripts

I, the Submitting Author has the right to grant and does grant on behalf of all authors of the Work (as defined in the below author licence), an exclusive licence and/or a non-exclusive licence for contributions from authors who are: i) UK Crown employees; ii) where BMJ has agreed a CC-BY licence shall apply, and/or iii) in accordance with the terms applicable for US Federal Government officers or employees acting as part of their official duties; on a worldwide, perpetual, irrevocable, royalty-free basis to BMJ Publishing Group Ltd ("BMJ") its licensees and where the relevant Journal is co-owned by BMJ to the co-owners of the Journal, to publish the Work in this journal and any other BMJ products and to exploit all rights, as set out in our licence.

The Submitting Author accepts and understands that any supply made under these terms is made by BMJ to the Submitting Author unless you are acting as an employee on behalf of your employer or a postgraduate student of an affiliated institution which is paying any applicable article publishing charge ("APC") for Open Access articles. Where the Submitting Author wishes to make the Work available on an Open Access basis (and intends to pay the relevant APC), the terms of reuse of such Open Access shall be governed by a Creative Commons licence – details of these licences and which Creative Commons licence will apply to this Work are set out in our licence referred to above.

Other than as permitted in any relevant BMJ Author's Self Archiving Policies, I confirm this Work has not been accepted for publication elsewhere, is not being considered for publication elsewhere and does not duplicate material already published. I confirm all authors consent to publication of this Work and authorise the granting of this licence.

For peer review only

Cost-effectiveness of pessary therapy versus surgery for symptomatic pelvic organ prolapse: an economic evaluation alongside a randomized non-inferiority controlled trial

Ângela Jornada Ben MD, Ph.D.[1], Lisa R. van der Vaart MD [2], Judith E. Bosmans Ph.D.[1], Jan-Paul W.R. Roovers MD, Ph.D [2,3], Antoine L. Lagro-Janssen MD, Ph.D [4], Carl H. van der Vaart MD, Ph.D [5,6] Astrid Vollebregt MD, Ph.D.[7]

[1] Department of Health Sciences, Faculty of Science, Vrije Universiteit Amsterdam, Amsterdam Public Health research institute, Amsterdam, The Netherlands.

[2] Department of Obstetrics and Gynecology, Amsterdam Reproduction & Development Research Institute, Amsterdam UMC, University of Amsterdam, Amsterdam, The Netherlands.

[3] Department of Gynecology, Women's Health, Bergman Clinics, Amsterdam, the Netherlands.

[4] Department of General Practice/ Women's studies Medicine, University Medical Center Radboud, Nijmegen, The Netherlands

[5] Department of Obstetrics and Gynecology, University Medical Center Utrecht, Utrecht, The Netherlands.

[6] Department of Gynecology, Women's Health, Bergman Clinics, Hilversum, the Netherlands.

[7] Department of Obstetrics and Gynecology, Spaarne Hospital, Haarlem, The Netherlands.

Corresponding author:

Ângela Jornada Ben. ORCID: 0000-0003-4793-9026. Department of Health Sciences, Faculty Science, Vrije Universiteit Amsterdam, Van der Boechorststraat 7, 1081 BT Amsterdam, The Netherlands. E-mail: a.jornadaben@vu.nl. Telephone: +31 6 25304906.

Orchid numbers:

Ângela J. Ben: 0000-0003-4793-9026

Lisa R van der Vaart: 0000-0003-1195-0047

Judith E. Bosmans: 0000-0002-1443-1026

Jan-Paul W.R. Roovers: 0000-0002-3069-6382

Antoine L. Lagro-Janssen: 0000-0003-2339-3743

Carl H. van der Vaart: 0000-0003-0890-5368

Astrid Vollebregt: 0000-0002-4704-1710

For peer review only - http://bmjopen.bmj.com/site/about/guidelines.xhtml

ABSTRACT

Objective: To evaluate the cost-effectiveness of pessary therapy as an initial treatment option compared to surgery for moderate to severe POP symptoms in secondary care from a healthcare and a societal perspective.

Design: Economic evaluation alongside a multicenter randomized controlled non-inferiority trial with a 24-month follow-up.

Setting: 21 hospitals in the Netherlands, recruitment conducted between 2015 – 2022.

Participants: 1605 women referred to secondary care with symptomatic prolapse stage ≥ 2 were requested to participate. Of them, 440 women gave informed consent and were randomized to pessary therapy (n=218) or to surgery (n=222) in a 1:1 ratio stratified by hospital.

Interventions: Pessary therapy and surgery.

Primary and secondary outcome measures: The Patient Global Impression of Improvement (PGI-I), a 7-point scale dichotomized into successful *vs.* unsuccessful, with a non-inferiority margin of -10%; Quality-Adjusted Life-Years (QALYs) measured by the EQ-5D-3L; healthcare and societal costs were based on medical records and the institute for Medical Technology Assessment (iMTA) questionnaires.

Results: For the PGI-I, the mean difference between pessary therapy and surgery was -0.05 (95% CI, -0.14; 0.03), and -0.03 (95% CI -0.07; 0.002) for QALYs. In total, 54.1% women randomized to pessary therapy crossed over to surgery, and 3.6% underwent recurrent surgery. Healthcare and societal costs were significantly lower in the pessary therapy (mean difference=-€1807, 95% CI -€2172; -€1446 and mean difference=-€1850, 95% CI -€2349; -€1341, respectively). The probability that pessary therapy is cost-effective compared to surgery was 1 at willingness-to-pay thresholds between €0 and €20000/QALY gained from both perspectives.

Conclusions: Non-inferiority of pessary therapy regarding the PGI-I could not be shown and no statistically significant differences in QALYs between interventions were found. Due to significantly lower costs, pessary therapy is likely to be cost-effective compared to surgery as an initial treatment option for women with symptomatic POP treated in secondary care.

Trial registration number: https://trialsearch.who.int/ Identifier: NTR4883.

For peer review only - http://bmjopen.bmj.com/site/about/guidelines.xhtml

Strengths and limitations of this study

- This economic evaluation was performed alongside a multicenter pragmatic randomized controlled trial. The randomization process ensures that groups are comparable and decrease the likelihood of selection bias while the multicenter pragmatic design improves generalizability of results and transferability to clinical practice.
- Validated outcome measures were used and the trial had a long-term follow-up of 2 years.
- Consultations related to both interventions were provided by gynecologists, which may overestimate intervention costs, as these consultations may be provided by trained general practitioners at lower costs.
- Resource utilization related to the specific medical treatment of interventions' complications (e.g., medications), productivity costs related to unpaid work, and informal care costs were not available and, thus, not included in the analysis, which may underestimate total costs.
- Costs were estimated based on the Dutch reimbursement system and can differ from countries which may hamper the generalizability of results to healthcare systems in other countries.

Funding statement: Financial support was provided through a personal grant (receiver: Carl H. van der Vaart) issued by the ZonMW, a Dutch governmental healthcare organization. This study was funded on 26 June 2014 (project no. 837002525).

Competing interests statement: Prof. Dr. C.H. van der Vaart reports grants from ZonMW Dutch government institution grant during the conduct of the study.

Patient consent forms: patient consent forms cannot be obtained because the patient cannot be traced due to anonymization of the data.

Data sharing statement: Data is available through **Lisa R van der Vaart (l.r.vdvaart@gmail.com)** upon reasonable request. To gain access, requesters will need to sign an agreement form and confirm that data will be used for the purpose for which access was granted. Stata code are available through the corresponding author upon reasonable request.

Word count: 4198 words.

For peer review only

INTRODUCTION

Pelvic organ prolapse (POP) is a gynecological condition in which one or more of the pelvic organs (i.e., uterus, rectum, bladder, small bowel) herniate into the vagina due to weakness or damaging of the pelvic floor muscles and ligaments[1,2]. POP symptoms (e.g., urinary, bowel, and sexual dysfunction) are associated with decreased quality of life[3]. The estimated prevalence of patient-reported POP symptoms ranges from 3 to 17.7% and is expected to increase with an aging population. As a result, the demand for care and associated costs are also expected to increase[4].

Effective treatment options for moderate to severe POP symptoms include pessary therapy and surgery[5,6]. However, both treatment options are not equally effective since non-inferiority of pessary therapy compared to surgery has not been shown[7]. A pessary is a silicone flexible device that is inserted into the vagina to support the pelvic organs (i.e., uterus and bladder)[8]. An advantage of pessary therapy is its minimally invasive nature. However, adverse effects (e.g., discomfort, pain, or excessive discharge) may occur in up to 49% of women within 12 to 24 months after fitting a pessary[9,10]. As for the surgery procedure, side-effects may include urinary tract infection and urinary bladder retention which may lead to longer admission hospital stay[7]. A recent observational study in women with a strong treatment preference and a randomized trial (RCT) in women without a preference found a high crossover rate from pessary therapy to surgery of 24% and 54%, respectively[7,9]. Consequently, using pessary therapy as an initial treatment option might delay effective treatment, thereby increasing the demand for care and, thus, healthcare costs. However, using a pessary as a first treatment step would prevent expensive surgery if the pessary therapy relieves women symptoms adequately, making the initial use of pessary therapy potentially cost-effective compared to immediate surgery.

According to a recent systematic review[8], only one model-based economic evaluation based on data from United States conducted more than 10 years ago compared the cost-effectiveness of expectant management, pessary therapy and surgery for POP symptoms [11]. This review reported that both pessary therapy and surgery were cost-effective compared to expectant management[11]. The aim of this study was to further investigate the cost-effectiveness of initial pessary therapy compared to immediate surgery from a healthcare and a societal perspective for moderate to severe POP symptoms with 2 years of follow-up. This study was performed alongside a non-inferiority randomized trial, of which the results have recently been published[7].

For peer review only - http://bmjopen.bmj.com/site/about/guidelines.xhtml

METHODS

Study design

An economic evaluation was conducted alongside a non-inferiority randomized controlled trial (RCT) comparing pessary therapy and surgery as an initial treatment for moderate to severe POP in secondary care, the PEOPLE project. The health economic analysis plan is available in the study protocol provided as Supplementary file 1. Participants were recruited between March 2015 and November 2019, the follow-up ended in June 2022. Detailed information about the PEOPLE project is published elsewhere[7,9,12]. This study was approved by the Medical Ethical Committee of the University Medical Center Utrecht (METC protocol number 14-533/M). No substantial changes were made to the protocol after the commencement of the RCT[7,12]. This economic evaluation is reported according to the Consolidated Health Economic Evaluating Reporting Standards statement[13]. All participants provided written informed consent.

Study population

Women with POP symptoms who were referred by their general practitioner (GP) to secondary care, were eligible for participation[7]. Inclusion criteria were POP stage ≥2 according to the Pelvic Organ Prolapse Quantification (POP-Q) system[14] and moderate to severe POP symptoms, defined as a prolapse domain score of >33 on the validated original Urinary Distress Inventory[15]. Exclusion criteria were prior prolapse or incontinence surgery, probability of future childbearing, insufficient knowledge of the Dutch language, comorbidity causing increased surgical risks, major psychiatric illness and prior pessary use[7]. Participants had to successfully complete a 30-minute pessary fitting trial to be eligible for randomization. After informed consent was signed, participants were randomly allocated to either pessary therapy or surgery in a 1:1 ratio[7]. Randomization used random permuted block sizes of 2 and 4 and was stratified by center. Due to the nature of the treatment, treatment allocation was not concealed. Women who actively opted for a treatment were asked to participate in an observational cohort performed alongside the RCT, their data were not included in economic evaluation, but published in another article[9]. Detailed information about study design and randomization can be found elsewhere[7,12].

For peer review only - http://bmjopen.bmj.com/site/about/guidelines.xhtml

Setting and location

Twenty-one Dutch hospitals participated in this multicenter RCT. In the Netherlands, women with moderate to severe POP symptoms are generally referred to secondary care. Treatment options in secondary care include pessary therapy or surgery, which are both reimbursed by the Dutch healthcare system. All gynecologist fitted at least 100 pessaries and performed 100 POP surgeries prior to study initiation.

Comparators

Pessary therapy

Two main types of pessary therapy were offered to participants, namely, supportive (i.e., ring) and occlusive (i.e., space filling)[16]. The pessary fitting was considered successful if the patient felt comfortable with the pessary in situ and if there was no pessary expulsion 30 minutes after fitting[7]. All women received verbal and written instructions on self-management of pessary therapy[7]. If self-management was not possible or preferred, an additional follow-up consultation with their gynecologist or GP was scheduled every four months for pessary cleaning and vaginal inspection[7]. In case women performed self-management, the frequency of cleaning was left to their personal preference, however it was advised to clean their pessary at least every 4 months. Women were instructed to return to the hospital if they experienced any complaint or adverse events due to pessary therapy[7].

Surgery

Surgical intervention included a range of surgical procedures for the correction of three main types of prolapse that can occur individually or simultaneously, namely, 1) uterine descent 2) cystocele, and/or 3) rectocele[7]. For a cystocele or rectocele, respectively a conventional anterior- or posterior colporrhaphy was the standard technique. For a uterine descent, uterine preserving techniques or a vaginal hysterectomy was performed[7]. All surgical interventions were performed following Dutch guidelines recommendations[7,17]. Decisions on which surgical technique was performed was decided in a shared-decision manner between gynecologist and participant[7]. Women were instructed to return to the hospital if they experienced any complaint or adverse events.

For peer review only - http://bmjopen.bmj.com/site/about/guidelines.xhtml

Study perspective, time horizon, and discount rate

This economic evaluation was conducted from a healthcare and a societal perspective over a time horizon of 24 months based on the literature and as recommended by the National Institute for Health and Clinical Excellence[6,8,18]. The healthcare perspective included costs related to interventions (pessary therapy and surgery) and healthcare utilization costs. The societal perspective included costs related to absenteeism from paid work in addition to the interventions' costs and healthcare utilization costs. Discount rates of 1.5% and 4% were applied to QALY and costs, respectively after the first year of the RCT as recommended by the Dutch Guideline for Economic Evaluations in healthcare[19].

Outcomes

Health outcomes

Two health outcomes were used for the trial-based economic evaluation: patient-reported subjective improvement and Quality-Adjusted Life-Years (QALYs). Subjective improvement was measured with the Patient Global Impression of Improvement (PGI-I)[20] scale at 12- and 24-month follow-up. The PGI-I is a single question, seven-point Likert response scale ranging from '*very much worse*' to '*very much better*'[20]. Subjective improvement was defined as a response of '*much better*' or '*very much better*'[21]. The PGI-I is a validated, easy to apply questionnaire, and it strongly correlates with other validated outcome measures such as the POP-Q system[14,20]. The primary analysis of PGI-I compared with surgery was presented in a previous publication in which its non-inferiority could not be shown[7]. This secondary analysis was performed as planned in the study protocol (Supplementary file 1)[22].

The QALY incorporates the impact of interventions on both the quantity and quality of life[23]. It is a routinely used health outcome measure in economic evaluations because it allows decision-makers to compare the cost-effectiveness of a range of interventions for different health conditions[23]. In this study, QALYs were calculated based on the EQ-5D-3L data collected at baseline, 3-, 6-, 12-, and 24-month follow-up. The EQ-5D-3L includes five dimensions of quality of life (i.e., mobility, self-care, usual activities, pain/discomfort and anxiety/depression) with three response levels (i.e., no problems, some problems or extreme problems/ unable to) describing 243 health states[24]. The participants' health states obtained from EQ-5D-3L responses

For peer review only - http://bmjopen.bmj.com/site/about/guidelines.xhtml

were converted into utility values using the Dutch tariff[25]. The utility values were used to calculate QALYs by means of linear interpolation (i.e., the duration of a health state is multiplied by the utility related to that health state)[26].

Cost outcomes

All costs were indexed to 2022 using the consumer price index in the Netherlands(www.cbs.nl)[27].

Intervention costs

Intervention costs of the pessary therapy included those related to the pessary device and one gynecologist consultation for the pessary placement at baseline. Unit prices of pessary therapy were based on the Dutch costing guideline[28] and on market prices (Supplementary file 2). For the surgery group, intervention costs consisted of the surgical procedures conducted at baseline. Unit prices of surgical procedures was based on the Diagnosis Treatment Combination (in Dutch *Diagnose Behandeling Combinatie*, DBC)[29]. The DBC is a care path that includes diagnostic procedures and care activities delivered at hospital and immediate follow-up up to 6 weeks (42 days)[29]. The average national prices are calculated for each DBC code based on all declared reimbursements that have been submitted to the DBC Information System (DIS) by healthcare providers in hospital care. A detailed description of the resources used in the interventions and their respective unit costs is presented in Supplementary file 2.

Healthcare utilization costs

Healthcare utilization was collected during follow-up visits at hospital centers including information on the number of scheduled consultations with gynecologists and extra consultations due to complications, the number of days of hospital readmissions due to complications, the type/number of surgeries after pessary, the type/number of re-surgeries, the number of times a pessary device was changed, and the use of a pessary after initial surgery. Additionally, an adapted version of the iMTA Medical Consumption Questionnaire (iMCQ)[30] was used to measure non-intervention related healthcare utilization at 3-, 6-, 12-, and 24-month follow-up. Healthcare utilization included resources used in primary care (i.e., the number of GP consultations and other healthcare professionals due to POP complaints), and in secondary care apart from study scheduled consultations (i.e., the number of extra consultations with other medical specialists due to POP complaints).

For peer review only - http://bmjopen.bmj.com/site/about/guidelines.xhtml

The number of healthcare resources used was then multiplied by their respective unit prices. Unit of prices of healthcare resources were based on the Dutch costing guideline[28] (Supplementary file 2).

Lost productivity costs

Absenteeism from paid work due to POP symptoms was measured using a adapted version of the iMTA Productivity Cost Questionnaire[31] at 3-, 6-, 12-, and 24-month follow-up. The friction cost approach (FCA) was used to calculate sickness absenteeism costs related to paid work[32]. The FCA assumes that sickness absenteeism costs are limited to the period needed to replace an absent, sick worker (the friction period), which has been estimated to be 12 weeks (85 days) in the Netherlands[32]. Gender-specific estimates of the mean wages of the Dutch population were used to calculate sickness absenteeism costs from paid work[28].

Cost-effectiveness analysis

Analyses were performed according to the intention-to-treat principle using StataSE V.17. As recommended by Faria et al,[33] mean imputation was used to impute missing values at baseline (i.e., parity, Patient Global Impression of Severity [PGIS], Pelvic Floor Distress Inventory [PFDI-20], Pelvic Organ Prolapse Distress Inventory [POPDI-6], Colorectal-Anal Distress Inventory [CRADI-8], Urinary Distress Inventory [UDI-6], and EQ-5D utility values). Subsequently, multiple imputation by Chained Equations (MICE) was used to impute follow-up missing data. The multiple imputation model included treatment group and hospital center, variables associated with missingness (i.e., Body Mass Index [BMI], number of re-surgeries, number of consultations, and family history of prolapse), outcomes, and potential confounders (i.e., age, history of gynecological operations, prolapse stage, menopausal state, and risk-increasing aspects)[34]. Risk-increasing aspects was a combined variable that included at least one of the following comorbidities: smoking status, antidepressants use, obesity, diabetes mellitus, and chronic pulmonary disease. Predictive Mean Matching was used in the imputation procedure to account for the skewed distribution of the costs[35]. Missing cost data were imputed at the level of resource use by time point (i.e., number of consultations, working hours and absenteeism hours). The number of imputations was increased until there was a loss of efficiency of ≤5%, resulting in ten imputed datasets[36]. The ten imputed datasets were analyzed separately and estimates were pooled using Rubin's rules[37].

For peer review only - http://bmjopen.bmj.com/site/about/guidelines.xhtml

For peer review only

Multilevel linear regression models were used to estimate the difference in costs and effects between the groups to account for the fact that randomization was stratified by hospital center[38]. For cost and effect outcomes, a two-level structure was used where participants and hospital center represented the first and second level, respectively. All analysis models were adjusted for relevant baseline confounders. The PGI-I model was adjusted for risk-increasing aspects and prolapse stage. The QALY model was adjusted for baseline utility values[39], risk-increasing aspects, and prolapse stage. Healthcare and societal costs models were adjusted for age, menopause state, risk-increasing aspects, and prolapse stage. A non-inferiority margin of 10% risk difference (one-sided 95% CI) was set for the PGI-I outcome based on the expectation that 80% of women would report successful treatment (either pessary therapy or surgery) after 2 years[12,40,41].

Incremental cost-effectiveness ratios (ICERs) were calculated by dividing the difference in costs between the pessary therapy and surgery by their difference in effects resulting in an estimate of the costs per unit of effect gained. Bias-corrected accelerated bootstrapping with 5000 replications was used to estimate the joint uncertainty surrounding differences in costs and effects. Bootstrapped cost-effect pairs were described and plotted on cost-effectiveness planes (CE-planes)[42]. Non-inferiority with regard to cost-effectiveness was demonstrated using a one-sided α of 2.5%, meaning that 97.5% of the cost-effect pairs have to lie right of the non-inferiority margin for effects[43]. Cost-effectiveness acceptability curves (CEACs) were estimated to show the probability of the pessary therapy being cost-effective compared to surgery for a range of willingness-to-pay (WTP) thresholds (i.e., the maximum amount of money society is willing to pay for a unit of effect)[44]. For QALY, we used a WTP threshold of 20000 €/QALY gained recommended by the Dutch Health Care Institute[45]. As there is no specific WTP threshold for PGI-I, we used a maximum WTP of 5237 €/PGI-I gained. This threshold was based on the average DBC costs of surgical procedures performed for POP symptoms as reported in Supplementary file 2.

Sensitivity Analysis

Two sensitive analyses (SA) were performed to assess the robustness of the results. SA1 was a complete case analysis, meaning that only observations with complete data were included in the main analysis. A per protocol analysis (SA2) was performed to compare treatment groups including women who completed the treatment to which they were originally allocated.

For peer review only - http://bmjopen.bmj.com/site/about/guidelines.xhtml

Patient and Public Involvement

One major gynecological patient organization in the Netherlands (i.e., BekkenBodem4All) as well as the Dutch Urogynecology Consortium fully agreed on the study protocol and identified the study as highly relevant[12].

For peer review only

For peer review only - http://bmjopen.bmj.com/site/about/guidelines.xhtml

RESULTS

Participants

Of the 1605 women assessed for eligibility, 440 were randomized to either pessary therapy (n=218) or surgery (n=222) as shown in Supplementary file 2. After randomization, one participant was excluded from the surgery group due to prolapse stage 1 resulting in a total of 221 women in this group (Supplementary file 2). Baseline incomplete data were imputed for parity (n=4, 0.9%), PFDI-20 (n=22, 5.0%), POPDI-6 (n=21, 4.8%), CRADI-8 (n=21, 4.8%), UDI-6 (n=22, 5.0%) and utility values (n=24, 5.5%) (Table 1). Follow-up missing data at 24-months were multiply imputed for PGI-I (n=104, 23.7%), QALY (n=144, 32.8%), healthcare costs (n=160, 36.4%), and societal costs (n=165, 37.6%) (Figure 1). A total of 118 of 218 (54.1%) women randomized to pessary therapy crossed over to surgery, and a total of 8 women out of 221 (3.6%) underwent recurrent surgery. At baseline, no meaningful differences were found between both groups (Table 1).

< Insert Table 1 here >

Effectiveness

In the unadjusted analysis, the lower 95%CI bound of the PGI-I outcome surpassed the non-inferiority margin of -10% (mean difference -0.06, 95% CI, -0.15; 0.04), meaning that non-inferiority of pessary therapy compared to surgery could not be shown (Table 2). After adjusting for confounders, the lower 95% CI bound of the PGI-I outcome still surpassed the non-inferiority margin (mean difference -0.05, 95% CI, -0.14; 0.03, Table 3). There was no statistically significant difference in QALYs between groups neither in the unadjusted analysis (mean difference -0.02, 95% CI, -0.06; 0.02, Table 2) nor the adjusted analysis (mean difference -0.03, 95% CI -0.07; 0.002, Table 3).

< Insert Table 2 here >

Costs

After 24 months, unadjusted analyses showed there were statistically significant savings in the pessary therapy group compared to the surgery for both total healthcare costs (mean difference -€1850, 95% CI, -€2228; -€1476) and societal costs (mean difference -€1878, 95% CI, -€2395; -€1345) (Table 2). Despite having other

For peer review only - http://bmjopen.bmj.com/site/about/guidelines.xhtml

surgery options (Supplementary file 2), we used a fixed price of €4640 considering the surgical procedures conducted in the trial. The main cost driver in the surgery group was the intervention costs (€4640, SE=0), while in the pessary therapy group this was secondary costs (€3736, SE=174) (Table 2). Given that half of patients in the pessary group crossed over to surgery (54.1%) and a small proportion of women underwent recurrent surgery in the surgery group (3.6%), secondary costs during follow-up were statistically significantly higher in the pessary therapy group compared to surgery (mean difference €2609, 95% CI, €2232; €2982, Table 2). In the adjusted analysis, mean differences in healthcare and societal costs between groups slightly decreased compared to the unadjusted analysis (Table 3). However, both healthcare and societal costs in the pessary group were still statistically significantly lower than in the surgery group.

< Insert Table 3 here >

Cost-effectiveness analysis

For the PGI-I outcome, the main analysis showed ICERs of 33509 and 34295 from a healthcare and a societal perspective, respectively (Table 3). The positive ICERs are situated in the SW quadrant of the CE plane and indicate that while pessary therapy incurred significantly lower costs (healthcare mean difference -€1807, 95% CI -€2172; -€1446 and societal mean difference -€1850, 95% CI -€2349; -€1341), it was also less effective compared to surgery (mean difference = -0.05, 95% CI, -0.14; 0.03), although not statistically significantly so. Most bootstrapped cost-effect pairs were situated on the right of the non-inferiority margin for effects (83.2%) and in the southern quadrants of the CE-Plane meaning that pessary therapy would save costs at an acceptable loss of effect in terms of PGI-I (Figure 1[1A] and [2A]). Due to statistically significant lower healthcare and societal costs in the pessary therapy group compared to surgery, CEACs showed that the probability of the pessary therapy being cost-effective compared to surgery was 1 at relevant WTP values (Figure 1 [1B] and [2B]). This means that the pessary therapy as an initial treatment option has a 100% probability of being cost-effective compared to immediate surgery.

< Insert Figure 1 here >

For QALYs, similar to PGI-I the positive, ICERs indicate that pessary therapy is less expensive and less effective (mean difference -0.03, 95% CI -0.07; 0.002) than surgery. However, the difference in QALYs was small and less

For peer review only - http://bmjopen.bmj.com/site/about/guidelines.xhtml

than the commonly used minimally clinically important difference (i.e., 0.06)[46,47] meaning that pessary therapy would save costs without considerably reducing health-related quality of life. The majority of the bootstrapped cost-effect pairs was in the southern quadrants of the CE-plane (100%) meaning that the pessary therapy was less costly than surgery (Figure 2 [1A] and [2A]). The probability that pessary therapy being cost-effective compared to surgery at all WTP thresholds was 1 from both perspectives (Figure 2 [1B] and [2B]).

< Insert Figure 2 here >

Sensitivity analysis

SA1 including only complete cases showed similar results compared to the main analysis (Table 3). In SA2, which included women that received their originally allocated intervention with fully imputed data on the PGI-I, (pessary therapy n=81, surgery n=190), the differences in costs and PGI-I between pessary and surgery increased and in QALY decreased compared to the main analysis (Table 3). However, this did not affect the cost-effectiveness results.

For peer review only

DISCUSSION

Main findings

This economic evaluation showed that non-inferiority of pessary therapy compared to surgery with regard to subjective improvement could not be shown, which was consistent with primary analysis of PGI-I[7]. Also, there were no statistically significant differences in QALY gained. Despite this, a strategy of initial pessary therapy in women with symptomatic POP is likely to be cost-effective compared to immediate surgery from a healthcare and a societal perspective due to lower costs associated with pessary therapy.

Explanation of the findings and comparison with the literature

For both effect outcomes, the high probability of pessary therapy being cost-effective compared to surgery is explained by the fact that total healthcare and societal costs in the pessary group were statistically significant lower than in the surgery group, despite the high proportion of crossover (54.1%) from participants in the pessary group to surgery.

Recently, Bugge et al. (2022)[8] systematically reviewed the (cost-)effectiveness of pessary therapy for managing POP symptoms and found only two economic evaluations[11,48]. Of those, only Hullfish et al. (2011)[11] directly compared pessary therapy with surgery. They developed a model-based economic evaluation with 12-month follow-up based on data from the literature, local experience of a single institution, and expert opinion. Results showed that for lower WTP thresholds (i.e. from 0 to 5600 $/QALY gained) pessary is cost-effective compared to surgery and for higher WTP thresholds (i.e., from 5600 to roughly 20000 $/QALY gained) not anymore. Our results, based on randomized data, showed that pessary therapy is cost-effective compared to surgery at similar WTP thresholds (i.e. 0 to 20000 €/QALY gained).

Strengths and Limitations

One of the strengths of this study is that it was performed alongside a multicenter pragmatic randomized controlled trial. The randomization process ensures that groups are comparable and decrease the likelihood of selection bias[49] while the multicenter pragmatic design improves generalizability of results and transferability to clinical practice. Validated outcome measures were used and the trial had a ~~long-term~~ follow-

For peer review only

For peer review only - http://bmjopen.bmj.com/site/about/guidelines.xhtml

up of 2 years. However, since POP symptoms can relapse over time, studies including a longer follow-up (e.g., more than 2 years) are needed. This study has a number of limitations. First, productivity costs related to unpaid work such as number of hours spent in unpaid activities (e.g., voluntary and housework) and informal care (e.g., care provided by family and friends while being sick) were not collected. Since the mean age of the participants is 65 years (the retirement age in the Netherlands until 2024), these costs are likely to be more relevant than lost productivity related to paid work. Second, consultations related to both interventions were provided by gynecologists, which may result in an overestimation of intervention costs. This may not be representative for healthcare systems in other countries, as these consultations may be provided by trained GPs at lower costs (i.e., €39 by a GP vs €109 by a medical specialist). Third, healthcare resource utilization related to the specific medical treatment of complications (e.g., medications) was not collected. Only costs related to readmissions and extra complications due to complications were included in the analysis. This may underestimate healthcare utilization costs. Fourth, the proportion of missing data on the outcomes was between 24 to 38%. To deal with this issue, multiple imputation of missing values were performed which is the recommended method to handle missing data in trial-based economic evaluations to produce valid estimates[33,50,51]. In addition, a sensitivity analysis including complete cases was performed to evaluate the robustness of findings, showing that results were not affected. Fifth, costs were estimated based on the Dutch reimbursement system and can differ from countries which may hamper the generalizability of results to healthcare systems in other countries.

Implications for practice and future research

A considerable number of women declined to participate in the RCT (n=553, Figure 1). These women were offered the possibility to participate in a prospective cohort[9]. The majority of participants in the prospective cohort opted for a pessary therapy as initial treatment option (62.2%)[9]. Compared to participants of the RCT[7], participants in the cohort less often crossed over to surgery (24% vs 54%). In addition, in this cohort, more women reported successful improvement after surgery compared to pessary[9]. This suggests that it is important to consider women's preferences when deciding about the most suitable treatment for their POP symptoms. Future studies should measure costs from a broader perspective than this study did, as relevant

For peer review only - http://bmjopen.bmj.com/site/about/guidelines.xhtml

costs were not considered in the analysis, that is, costs related to follow-up medical treatment, informal care costs and lost productivity costs related to unpaid work (e.g., housework, voluntary work).

For peer review only

For peer review only - http://bmjopen.bmj.com/site/about/guidelines.xhtml

CONCLUSION

Non-inferiority of pessary therapy with regard to the PGI-I could not be shown and there were no statistically significant differences in QALYs between interventions. Due to significantly lower costs, pessary therapy is likely to be cost-effective compared to immediate surgery from a healthcare and a societal perspective as an initial treatment option for women with moderate to severe POP symptoms treated in secondary care. However, considering the high crossover rate from pessary therapy to surgery it is important to consider women's preferences regarding the treatment of their POP systems.

FIGURE 1. COST-EFFECTIVENESS PLANES AND COST-EFFECTIVENESS ACCEPTABILITY CURVES FOR PATIENT GLOBAL IMPRESSION IMPROVEMENT (PGI-I). Cost-effectiveness planes (CE-planes [1A] and [2A]) and cost-effectiveness acceptability curves (CEACs [1B] and [2B]) comparing pessary therapy with surgery for the PGI-I outcome from a healthcare and a societal perspective, respectively. CE-planes show the incremental cost-effectiveness ratio point estimate (ICER, red dot) and the distribution of the 5000 replications of the bootstrapped cost-effective pairs (blue dots). CEACs indicate the probability of pessary therapy being cost-effective compared with surgery (y-axis) for different willingness-to-pay (WTP) thresholds per unit of PGI-I gained (x-axis). The dashed line represents the non-inferiority margin of 10%. [1A] and [2A] show that all of bootstrapped cost-effect pairs were distributed in the southern quadrants of the CE-planed meaning that the pessary therapy is less costly but could also be less and more effective. 83.2% bootstrapped cost-effect pairs are situated on the right of the non-inferiority margin for effects.[1B] and [2B] indicate a steady probability of 1 that the pessary therapy is cost-effective compared with surgery for different WTP thresholds per PGI-I gained. PGI-I is presented as the difference between groups in the proportion of participants reporting improvement.

FIGURE 2. COST-EFFECTIVENESS PLANES AND COST-EFFECTIVENESS ACCEPTABILITY CURVES FOR QUALITY-ADJUSTED LIFE-YEARS (QALY). Cost-effectiveness planes (CE-planes [1A] and [2A]) and cost-effectiveness acceptability curves (CEACs [1B] and [2B]) comparing pessary therapy with surgery for QALY from a healthcare and a societal perspective, respectively. CE-planes show the incremental cost-effectiveness ratio point estimate (ICER, red dot) and the distribution of the 5000 replications of the bootstrapped cost-effective pairs (blue dots). CEACs indicate the probability of pessary therapy being cost-effective compared with surgery (y-axis) for different willingness-to-pay (WTP) thresholds per QALY gained (x-axis). [1A] and [2A] show that all of bootstrapped cost-effect pairs were distributed in the southern quadrants of the CE-planed meaning that the pessary therapy is less costly but could also be less and more effective. [1B] and [2B] indicate a steady probability of 1 that the pessary therapy is cost-effective compared with surgery for different WTP thresholds per QALY gained.

For peer review only - http://bmjopen.bmj.com/site/about/guidelines.xhtml

REFERENCES

1 Aboseif C, Liu P. *Pelvic Organ Prolapse*. StatPearls Publishing 2022. https://www.ncbi.nlm.nih.gov/books/NBK563229/ (accessed 22 August 2022)

2 Harrison BP, Cespedes RD. PELVIC ORGAN PROLAPSE*. *Emergency Medicine Clinics of North America*. 2001;19:781–98.

3 Doaee M, Moradi-Lakeh M, Nourmohammadi A, *et al.* Management of pelvic organ prolapse and quality of life: a systematic review and meta-analysis. *Int Urogynecol J*. 2014;25:153–63.

4 Dieter AA, Wilkins MF, Wu JM. Epidemiological trends and future care needs for pelvic floor disorders. *Current Opinion in Obstetrics and Gynecology*. 2015;27:380–4.

5 Brown HW, Hegde A, Huebner M, *et al.* International urogynecology consultation chapter 1 committee 2: Epidemiology of pelvic organ prolapse: prevalence, incidence, natural history, and service needs. *Int Urogynecol J*. 2022;33:173–87.

6 van Geelen JM, Dwyer PL. Where to for pelvic organ prolapse treatment after the FDA pronouncements? A systematic review of the recent literature. *Int Urogynecol J*. 2013;24:707–18.

7 van der Vaart LR, Vollebregt A, Milani AL, *et al.* Effect of Pessary vs Surgery on Patient-Reported Improvement in Patients With Symptomatic Pelvic Organ Prolapse: A Randomized Clinical Trial. *JAMA*. 2022;328:2312–23.

8 Bugge C, Adams EJ, Gopinath D, *et al.* Pessaries (mechanical devices) for managing pelvic organ prolapse in women. *Cochrane Database of Systematic Reviews*. Published Online First: 2020. doi: 10.1002/14651858.CD004010.pub4

9 van der Vaart L, Vollebregt A, Milani A, *et al.* Pessary or surgery for a symptomatic pelvic organ prolapse: the PEOPLE study, a multicentre prospective cohort study. *BJOG*. 2022;129:820–9.

10 de Albuquerque Coelho SC, de Castro EB, Juliato CRT. Female pelvic organ prolapse using pessaries: systematic review. *Int Urogynecol J*. 2016;27:1797–803.

11 Hullfish KL, Trowbridge ER, Stukenborg GJ. Treatment strategies for pelvic organ prolapse: a cost-effectiveness analysis. *Int Urogynecol J*. 2011;22:507–15.

12 PEOPLE group. Healthcare evaluation in the Netherlands. - PEOPLE Project. 2015. https://www.zorgevaluatienederland.nl/evaluations/people (accessed 11 August 2022)

13 Husereau D, Drummond M, Augustovski F, *et al.* Consolidated Health Economic Evaluation Reporting Standards 2022 (CHEERS 2022) Statement: Updated Reporting Guidance for Health Economic Evaluations. *Value in Health*. 2022;25:3–9.

14 Bump RC, Mattiasson A, Bø K, *et al.* The standardization of terminology of female pelvic organ prolapse and pelvic floor dysfunction. *American Journal of Obstetrics and Gynecology*. 1996;175:10–7.

15 Shumaker SA, Wyman JF, Uebersax JS, *et al.* Health-related quality of life measures for women with urinary incontinence: the Incontinence Impact Questionnaire and the Urogenital Distress Inventory. Continence Program in Women (CPW) Research Group. *Qual Life Res*. 1994;3:291–306.

16 Cundiff GW, Amundsen CL, Bent AE, *et al.* The PESSRI study: symptom relief outcomes of a randomized crossover trial of the ring and Gellhorn pessaries. *American Journal of Obstetrics and Gynecology*. 2007;196:405.e1-405.e8.

For peer review only - http://bmjopen.bmj.com/site/about/guidelines.xhtml

17 Federatie Medisch Specialisten. Prolapse: guideline about the best care for patients with prolapse according to current standards. Utrecht, the Netherlands. 2014. https://richtlijnendatabase.nl/richtlijn/prolaps/prolaps_-_startpagina.html (accessed 22 March 2023)

18 Overview | Urinary incontinence and pelvic organ prolapse in women: management | Guidance | NICE. 2019. https://www.nice.org.uk/guidance/ng123 (accessed 19 December 2023)

19 VWS. Guideline for economic evaluations in healthcare - Dutch National Health Care Institute - Ministerie van Volksgezondheid, Welzijn en Sport. 2016. https://english.zorginstituutnederland.nl/publications/reports/2016/06/16/guideline-for-economic-evaluations-in-healthcare (accessed 23 August 2018)

20 Srikrishna S, Robinson D, Cardozo L. Validation of the Patient Global Impression of Improvement (PGI-I) for urogenital prolapse. *Int Urogynecol J*. 2010;21:523–8.

21 Labrie J, Berghmans BLCM, Fischer K, *et al.* Surgery versus Physiotherapy for Stress Urinary Incontinence. *N Engl J Med*. 2013;369:1124–33.

22 ZonMw. ZonMw. https://www.zonmw.nl/nl/ (accessed 18 June 2022)

23 Drummond MF, Sculpher MJ, Torranc GW. *Methods for the economic evaluation of health care programmes*. 3rd ed. Oxford: Oxford University Press 2005.

24 EuroQol Group. EuroQol - a new facility for the measurement of health-related quality of life. *Health Policy*. 1990;16:199–208.

25 Lamers LM, McDonnell J, Stalmeier PFM, *et al.* The Dutch tariff: results and arguments for an effective design for national EQ-5D valuation studies. *Health Economics*. 2006;15:1121–32.

26 Whitehead SJ, Ali S. Health outcomes in economic evaluation: the QALY and utilities. *Br Med Bull*. 2010;96:5–21.

27 CBS. StatLine - Consumentenprijzen. https://opendata.cbs.nl/#/CBS/nl/dataset/83131NED/table?ts=1660927150933 (accessed 21 August 2022)

28 Kanters TA, Bouwmans CAM, van der Linden N, *et al.* Update of the Dutch manual for costing studies in health care. *PLoS One*. 2017;12. doi: 10.1371/journal.pone.0187477

29 Diagnose Behandeling Combinatie (DBC) open data - NZa. https://www.opendisdata.nl/ (accessed 3 September 2022)

30 iMTA. iMTA Medical Consumption Questionnaire. iMTA. https://www.imta.nl/questionnaires/imcq/ (accessed 6 February 2022)

31 Bouwmans C, Krol M, Severens H, *et al.* The iMTA Productivity Cost Questionnaire: A Standardized Instrument for Measuring and Valuing Health-Related Productivity Losses. *Value in Health*. 2015;18:753–8.

32 Koopmanschap MA, Rutten FFH, van Ineveld BM, *et al.* The friction cost method for measuring indirect costs of disease. *Journal of Health Economics*. 1995;14:171–89.

33 Faria R, Gomes M, Epstein D, *et al.* A Guide to Handling Missing Data in Cost-Effectiveness Analysis Conducted Within Randomised Controlled Trials. *Pharmacoeconomics*. 2014;32:1157.

For peer review only - http://bmjopen.bmj.com/site/about/guidelines.xhtml

34 Schulten SFM, Claas-Quax MJ, Weemhoff M, *et al.* Risk factors for primary pelvic organ prolapse and prolapse recurrence: an updated systematic review and meta-analysis. *American Journal of Obstetrics and Gynecology*. 2022;227:192–208.

35 White IR, Royston P, Wood AM. Multiple imputation using chained equations: Issues and guidance for practice. *Statistics in Medicine*. 2011;30:377–99.

36 Madley-Dowd P, Hughes R, Tilling K, *et al.* The proportion of missing data should not be used to guide decisions on multiple imputation. *Journal of Clinical Epidemiology*. 2019;110:63–73.

37 Little RJA, Rubin DB. *Statistical Analysis with Missing Data*. New York, NY, USA: John Wiley & Sons, Inc. 2014.

38 Gomes M, Grieve R, Nixon R, *et al.* Statistical Methods for Cost-Effectiveness Analyses That Use Data from Cluster Randomized Trials: A Systematic Review and Checklist for Critical Appraisal. *Med Decis Making*. 2012;32:209–20.

39 Manca A, Hawkins N, Sculpher MJ. Estimating mean QALYs in trial-based cost-effectiveness analysis: the importance of controlling for baseline utility. *Health Economics*. 2005;14:487–96.

40 Koullali B, van Kempen LEM, van Zijl MD, *et al.* A multi-centre, non-inferiority, randomised controlled trial to compare a cervical pessary with a cervical cerclage in the prevention of preterm delivery in women with short cervical length and a history of preterm birth – PC study. *BMC Pregnancy Childbirth*. 2017;17:215.

41 Non-Inferiority Clinical Trials to Establish Effectiveness Guidance for Industry. *US Department of Health and Human Services Food and Drug Administration Center for Drug Evaluation and Research (CDER) Center for Biologics Evaluation and Research (CBER)*.

42 Black WC. The CE plane: a graphic representation of cost-effectiveness. *Med Decis Making*. 1990;10:212–4.

43 Bosmans JE, de Bruijne MC, van Hout HPJ, *et al.* Practical guidelines for economic evaluations alongside equivalence trials. *Value Health*. 2008;11:251–8.

44 Fenwick E, Claxton K, Sculpher M. Representing uncertainty: the role of cost-effectiveness acceptability curves. *Health Econ*. 2001;10:779–87.

45 ZIN. Zorginstituut Nederland - Richtlijn voor het uitvoeren van economische evaluaties in de gezondheidszorg. Ministerie van Volksgezondheid, Welzijn en Sport. 2016. https://www.zorginstituutnederland.nl/publicaties/publicatie/2016/02/29/richtlijn-voor-het-uitvoeren-van-economische-evaluaties-in-de-gezondheidszorg (accessed 28 March 2018)

46 Walters SJ, Brazier JE. Comparison of the minimally important difference for two health state utility measures: EQ-5D and SF-6D. *Qual Life Res*. 2005;14:1523–32.

47 Luo N, Johnson JA, Coons SJ. Using Instrument-Defined Health State Transitions to Estimate Minimally Important Differences for Four Preference-Based Health-Related Quality of Life Instruments: *Medical Care*. 2010;48:365–71.

48 Panman C, Wiegersma M, Kollen B, *et al.* Two-year effects and cost-effectiveness of pelvic floor muscle training in mild pelvic organ prolapse: a randomised controlled trial in primary care. *BJOG: An International Journal of Obstetrics & Gynaecology*. 2017;124:511–20.

49 Randomization-Based Evaluations. *Multiple Imputation for Nonresponse in Surveys*. Wiley-Blackwell 2008:113–53. https://doi.org/10.1002/9780470316696.ch4

For peer review only - http://bmjopen.bmj.com/site/about/guidelines.xhtml

50 MacNeil Vroomen J, Eekhout I, Dijkgraaf MG, *et al.* Multiple imputation strategies for zero-inflated cost data in economic evaluations: which method works best? *Eur J Health Econ*. 2016;17:939–50.

51 Ben ÂJ, van Dongen JM, Alili ME, *et al.* The handling of missing data in trial-based economic evaluations: should data be multiply imputed prior to longitudinal linear mixed-model analyses? *Eur J Health Econ*. Published Online First: 26 September 2022. doi: 10.1007/s10198-022-01525-y

Authors' contributions: **Ângela Jornada Ben:** Methodology, Data Curation, Formal analysis, and Writing - Original Draft. **Lisa R van der Vaart:** Methodology, Data Curation, Writing - Review & Editing, and Project administration. **Judith E. Bosmans:** Methodology, Writing - Review & Editing, Supervision. **Jan-Paul W.R. Roovers:** Writing - Review & Editing. **Antoine L. Lagro-Janssen:** Writing - Review & Editing. **Carl H. van der Vaart**: Conceptualization, Methodology, Resources, Funding acquisition, and Writing - Review & Editing. **Astrid Vollebregt**: Conceptualization, Methodology, and Writing - Review & Editing, Supervision. All authors have agreed to the submission of the final paper.

Acknowledgments: PEOPLE group: Milani, F, van Bavel, J., Bon G.G., Bongers M.Y., Bos K., Broekman A.M.W., Dietz, V., van Eijndhoven H.W.F., Hakvoort R.A., Janszen E.W.M., Kluivers K., Link G., Massop-Helmink D.S., van der Ploeg J.M., Sikkema F.M., Spaans W.A., van der Ster A., Verheijen-van de Waarsenburg, K., Vernooij M.M.A., Weemhoff, M., A.E. Weis-Potters.

For peer review only

For peer review only - http://bmjopen.bmj.com/site/about/guidelines.xhtml

TABLE 1. BASELINE CHARACTERISTICS OF PARTICIPANTS

Baseline characteristic	Pessary therapy *n = 218*	Surgery *n = 221*
Age (mean (SD))	64.8 (9.5), n=218	64.7(9.2), n=221
Risk-increasing aspects ¥ (n, %)	71 (32.6), n=218	58 (26.2), n=221
History of gynecological surgery (n, %)	22 (10.1), n=218	28 (12.7), n=221
Family history of prolapse (n, %)	106 (48.6), n=218	107 (49.5), n=216
Parity (median (IQR)	2.0 (2-3), n=215	2.0 (2-3), n=220
Postmenopausal (n, %)	186 (92.5), n=201	185 (90.2), n=205
Duration of symptoms in months (median (IQR)	6 (2-24), n=211	6 (3-24), n=216
Vaginal atrophy (n, %)	106 (56.7), n=187	110 (57.3), n=192
Prolapse stage (n, %)		
II (Moderate)	85 (39.0), n=218	102 (46.2), n=221
≥III (Severe)	133 (61.0), n=218	119 (53.9), n=221
PGI-S score[a] (n, %)		
I (Not severe)	13 (6.3), n=205	9 (4.4), n=205
II (Mild)	48 (23.4), n=205	50 (24.4), n=205
III (Moderate)	99 (48.3), n=205	112 (54.6), n=205
IV (Severe)	45 (22.0), n=205	34 (16.6), n=205
PFDI-20 score[b] (n, %)		
POPDI-6 score	29.5 (19.2), n=210	28.7 (15.6), n=208
CRADI-8 score	13.9 (15.1), n=210	12.1 (12.6), n=208
UDI-6 score	26.0 (22.0), n=209	25.2 (20.0), n=208
PFDI-20 total score	69.3 (45.7), n=209	65.9 (37.7), n=208
EQ-5D utility value[c] (mean (SD))	0.87 (0.15), n=209	0.85 (0.15), n=206

SD = standard deviation. n = number of women. % = proportion. IQR = interquartile range. [a]PGIS = Patient Global Impression of Severity: I (not severe), II (mild), III (moderate), IV (severe). [b]PFDI-20 = Pelvic Floor Distress Inventory: the subscale scores range from 0-100 and the total score ranges from 0 to 300. Higher scores indicate more symptom distress. POPDI-6 = Pelvic Organ Prolapse Distress Inventory. CRADI-8 = Colorectal-Anal Distress Inventory. UDI-6 = Urinary Distress Inventory. [c]EQ-5D utility values: the Dutch EQ-5D tariffs range from -0.33 to 1. [¥]presence of 1 or more comorbidities: smoking, use of antidepressants, obesity, diabetes mellitus, chronic pulmonary disease.

For peer review only

For peer review only - http://bmjopen.bmj.com/site/about/guidelines.xhtml

TABLE 2. EFFECTS AND COSTS BY TREATMENT GROUP AND DIFFERENCE AT 24-MONTH FOLLOW-UP

	**Pessary therapy** ***n = 218***	**Surgery** ***n = 221***	**Unadjusted Difference (95% CI)**
**Effects**			
PGI-I, n (%)	164 (75.1%)	179 (80.8%)	-0.06 (-0.15; 0.04)
QALY, mean (SE)	1.80 (0.02)	1.82 (0.01)	-0.02 (-0.06; 0.02)
**Costs, mean (SE)**			
Intervention costs	178 (0.2)	4640 (0)	-4462 (-4463; -4462)
Primary care costs	18 (2)	15 (2)	3 (-3; 8)
Secondary care costs	3736 (174)	1127 (80)	2609 (2232; 2982)
Healthcare costs	3932 (174)	5782 (80)	-1850 (-2228; -1476)
Absenteeism from paid work	362 (117)	390 (120)	-28 (-338; 290)
Societal costs	4294 (227)	6172 (150)	-1878 (-2395, -1345)

PGI-I = Patient Global Impression of Improvement (1=improvement; 0= no improvement). PGI-I is presented as the difference between groups in the proportion of participants reporting improvement. n = number of participants. % = proportion. SE = standard error. Intervention costs in the pessary group = costs of pessary device and pessary placement consultation at baseline. Intervention costs in the surgery group = DBC costs of surgery at baseline which included one follow-up consultation at 6 weeks. Primary care costs = costs of general practitioner or other healthcare professional consultations apart from the pre-scheduled follow-up consultations because of complaints related to pelvic organ prolapse (POP) symptoms. Secondary care costs = costs of follow-up scheduled consultations with gynecologists attended by patients and extra consultations due to complications, costs of hospital readmissions due to complications, surgeries after pessary, re-surgeries, and costs of pessary change

For peer review only

For peer review only - http://bmjopen.bmj.com/site/about/guidelines.xhtml

TABLE 3. RESULTS OF THE COST-EFFECTIVENESS AND COST-UTILITY ANALYSIS

Effect outcome	ΔE (95% CI)	ΔC (95% CI)	ICER	Proportion of bootstrapped cost-effect pairs in the CE-plane			
				NE	SE	SW	NW
**Main analysis – Healthcare Perspective**							
PGI-I, n=439	-0.05 (-0.14; 0.03)	-1807 (-2172; -1446)	33509	0%	9%	91%	0%
QALY, n=439	-0.03 (-0.07; 0.002)	-1807 (-2172; -1446)	52980	0%	3%	97%	0%
**Main analysis – Societal Perspective**							
PGI-I, n=439	-0.05 (-0.14; 0.03)	-1850 (-2349; -1341)	34295	0%	9%	91%	0%
QALY, n=439	-0.03 (-0.07; 0.002)	-1850 (-2349; -1341)	54223	0%	3%	97%	0%
**Sensitivity analysis 1 – Complete Case Analysis – Healthcare Perspective**							
PGI-I, n=259	-0.02 (-0.11; 0.07)	-1976 (-2460; -1585)	81560	0%	25%	75%	0%
QALY, n=256	-0.01 (-0.05; 0.03)	-1962 (-2470; -1572)	236907	0%	33%	67%	0%
**Sensitivity analysis 1 – Complete Case Analysis – Societal Perspective**							
PGI-I, n=254	-0.02 (-0.11; 0.08)	-1884 (-2499; -1241)	99339	0%	30%	70%	0%
QALY, n=252	-0.005 (-0.05; 0.04)	-1860 (-2500; -1225)	367444	0%	39%	61%	0%
**Sensitivity analysis 2 – Per Protocol Analysis – Healthcare Perspective**							
PGI-I, n=271	-0.13 (-0.25; -0.01)	-4398 (-4583; -4311)	33044	0%	1%	99%	0%
QALY, n=271	-0.01 (-0.05; 0.02)	-4398 (-4583; -4311)	358020	0%	27%	73%	0%
**Sensitivity analysis 2 – Per Protocol Analysis – Societal Perspective**							
PGI-I, n=271	-0.13 (-0.25; -0.01)	-4748 (-5159; -4498)	35676	0%	1%	99%	0%
QALY, n=271	-0.01 (-0.05; 0.02)	-4748 (-5159; -4498)	386539	0%	27%	73%	0%

ΔC= difference in costs in Euros; 95% CI = 95% confidence interval; ΔE= difference in effects; ICER = Incremental Cost-Effectiveness Ratio (€ per unit of effect gained); CE-plane = cost-effectiveness plane showing the difference in costs between pessary therapy and surgery on the y-axis and the difference in effects on the x-axis resulting in four quadrants namely, NE = northeast (pessary therapy more expensive and more effective than surgery); SE = southeast (pessary therapy less expensive and more effective than surgery); SW = southwest (pessary therapy less expensive and less effective than surgery); NW = northwest (pessary therapy more expensive and less effective than surgery).The PGI-I model was adjusted by risk-increasing aspects, and prolapse stage. The QALY model was adjusted by baseline utility values, risk-increasing aspects, and prolapse stage. Healthcare and societal costs models were adjusted by age, menopause state, risk-increasing aspects, and prolapse stage. PGI-I is presented as the difference between groups in the proportion of participants reporting improvement.

For peer review only - http://bmjopen.bmj.com/site/about/guidelines.xhtml

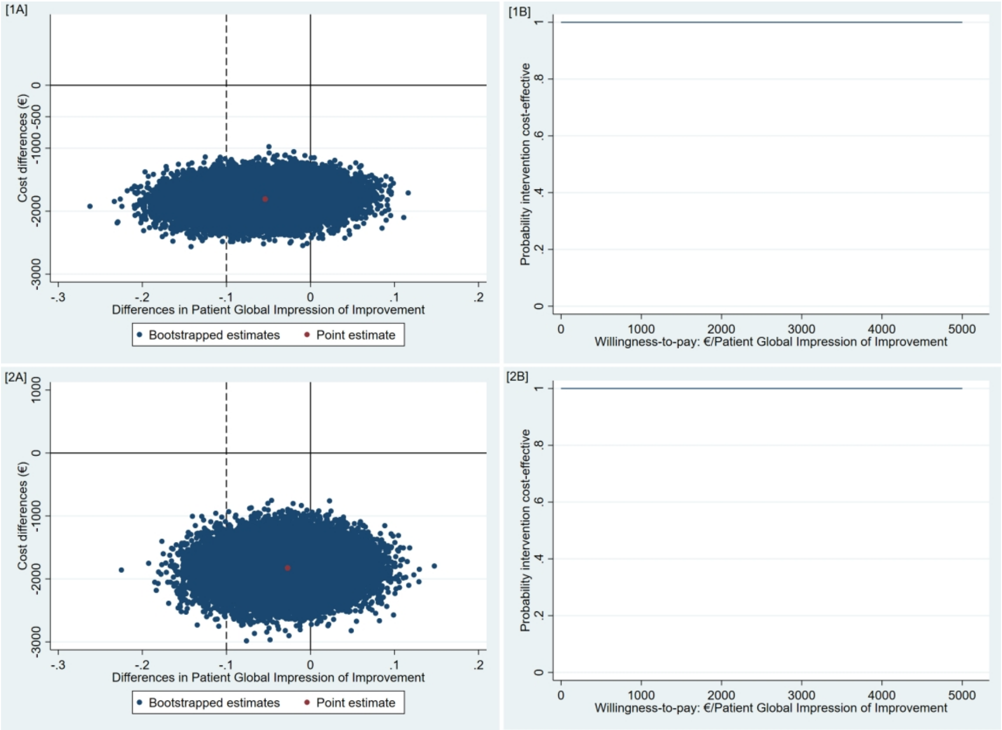

FIGURE 1. COST-EFFECTIVENESS PLANES AND COST-EFFECTIVENESS ACCEPTABILITY CURVES FOR PATIENT GLOBAL IMPRESSION IMPROVEMENT (PGI-I). Cost-effectiveness planes (CE-planes [1A] and [2A]) and cost-effectiveness acceptability curves (CEACs [1B] and [2B]) comparing pessary therapy with surgery for the PGI-I outcome from a healthcare and a societal perspective, respectively. CE-planes show the incremental cost-effectiveness ratio point estimate (ICER, red dot) and the distribution of the 5000 replications of the bootstrapped cost-effective pairs (blue dots). CEACs indicate the probability of pessary therapy being cost-effective compared with surgery (y-axis) for different willingness-to-pay (WTP) thresholds per unit of PGI-I gained (x-axis). The dashed line represents the non-inferiority margin of 10%. [1A] and [2A] show that all of bootstrapped cost-effect pairs were distributed in the southern quadrants of the CE-planed meaning that the pessary therapy is less costly but could also be less and more effective. 83.2% bootstrapped cost-effect pairs are situated on the right of the non-inferiority margin for effects.[1B] and [2B] indicate a steady probability of 1 that the pessary therapy is cost-effective compared with surgery for different WTP thresholds per PGI-I gained. PGI-I is presented as the difference between groups in the proportion of participants reporting improvement.

1712x1249mm (38 x 38 DPI)

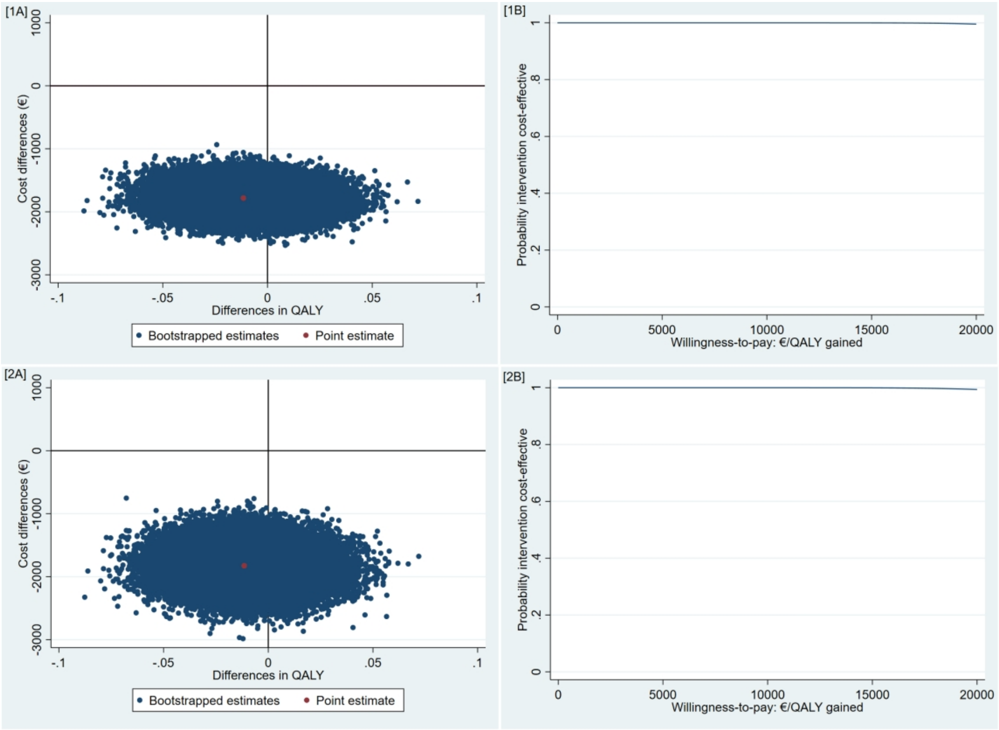

FIGURE 2. COST-EFFECTIVENESS PLANES AND COST-EFFECTIVENESS ACCEPTABILITY CURVES FOR QUALITY-ADJUSTED LIFE-YEARS (QALY). Cost-effectiveness planes (CE-planes [1A] and [2A]) and cost-effectiveness acceptability curves (CEACs [1B] and [2B]) comparing pessary therapy with surgery for QALY from a healthcare and a societal perspective, respectively. CE-planes show the incremental cost-effectiveness ratio point estimate (ICER, red dot) and the distribution of the 5000 replications of the bootstrapped cost-effective pairs (blue dots). CEACs indicate the probability of pessary therapy being cost-effective compared with surgery (y-axis) for different willingness-to-pay (WTP) thresholds per QALY gained (x-axis). [1A] and [2A] show that all of bootstrapped cost-effect pairs were distributed in the southern quadrants of the CE-planed meaning that the pessary therapy is less costly but could also be less and more effective. [1B] and [2B] indicate a steady probability of 1 that the pessary therapy is cost-effective compared with surgery for different WTP thresholds per QALY gained.

1715x1249mm (38 x 38 DPI)

PEOPLE study

Pessary or Surgery for a Symptomatic Pelvic Organ Prolapse

Study protocol

For peer review only

For peer review only - http://bmjopen.bmj.com/site/about/guidelines.xhtml

Table of contents

1. Study protocols 3

1.1 Original study protocol...4

1.2 Final study protocol 55

1.3 Summary of amendments to study protocol.. 107

1.3.1 Detailed summary of all amendments 107

1.3.2 Table with amendments and corresponding section 109

For peer review only - http://bmjopen.bmj.com/site/about/guidelines.xhtml

1. Study protocols

Original study protocol: Version 1.5, November 2014

Final study protocol: Version 1.22, February 2018

For peer review only

1.1 Original study protocol

Pessary or surgery for symptomatic pelvic organ prolapse

Version 1.~~4~~ 5 ~~October~~ November 2014

For peer review only - http://bmjopen.bmj.com/site/about/guidelines.xhtml

PROTOCOL TITLE 'Pessary or surgery for symptomatic pelvic organ prolapse'

**Protocol ID**	**2014 / 1.4**
**Short title**	**Pessary or surgery for symptomatic pelvic organ prolapse**
**EudraCT number**	***Not applicable***
**Version**	**1.~~4~~5**
**Date**	**O~~ctober~~ November 2014**
**Coordinating investigator/project leader**	**Prof. Dr. C.H. van der Vaart, gynaecologist**<br>**University Medical Centre Utrecht**
**Principal investigator(s) (in Dutch: hoofdonderzoeker/ uitvoerder)**	**Dr. A. Vollebregt, gynaecologist**<br>**Spaarne Hospital**<br><br>**M.K. van de Waarsenburg, MD**<br>**University Medical Centre Utrecht**
**Multicenter: per site**	**Prof. Dr. C.H. van der Vaart, UMC Utrecht**<br>**Dr. A.L. Milani, Reinier de Graaf Gasthuis**<br>**Dr. A. Vollebregt, Spaarne Hospital Hoofddorp**<br>**Dr. J.P.W.R. Roovers, Academic Medical Center**<br>**Dr. K.B. Kluivers, Radboud UMC Nijmegen**<br>**Dr. V. Dietz, Catharina Hospital Eindhoven**<br>**Dr. H.W.F. van Eijndhoven, Isala Clinic Zwolle**<br>**Dr. H.S. The, Sint Antonius Hospital Nieuwegein**<br>**Dr. R.P. Schellart, Kennemer Gasthuis Haarlem**<br>**Drs. A.M.W. Broekman, Sint Franciscus Gasthuis Rotterdam**<br>**Drs. J. van Bavel, Amphia Hospital Breda**
**Sponsor (in Dutch: verrichter/opdrachtgever)**	**Prof. Dr. C.H. van der Vaart, gynaecologist**
**Subsidising party**	**ZonMw Project nr 837002525**
**Independent expert (s)**	**Dr. R.P. Zweemer**<br><br>**University Medical Centre Utrecht**

For peer review only

**Laboratory sites <*if applicable*>**	***Not applicable***
**Pharmacy <*if applicable*>**	***Not applicable***

For peer review only

For peer review only - http://bmjopen.bmj.com/site/about/guidelines.xhtml

PROTOCOL SIGNATURE SHEET

Name	Signature	Date
**Head of Department:**<br><br>**Prof. Dr. B.C.J.M. Fauser**<br>**Department of Reproductive Medicine and Gynaecology**<br>**University Medical Centre Utrecht**		
**[Coordinating Investigator/Project leader/Principal Investigator]:**<br><br>**Prof. Dr. C.H. van der Vaart, gynaecologist**<br>**University Medical Centre Utrecht**		

For peer review only

TABLE OF CONTENTS

1. INTRODUCTION AND RATIONALE 9
2. OBJECTIVES 13
3. STUDY DESIGN 14
4. STUDY POPULATION 15
4.1 Population (base) 15
4.2 Inclusion criteria 15
4.3 Exclusion criteria 15
4.4 Sample size calculation 15
5. TREATMENT OF SUBJECTS 17
5.1 Investigational product/treatment 17
5.2 Use of co-intervention (if applicable) 17
5.3 Escape medication (if applicable) 18
6. INVESTIGATIONAL PRODUCT 19
6.1 Name and description of investigational product(s) 19
6.2 Summary of findings from non-clinical studies 19
6.3 Summary of findings from clinical studies 19
6.4 Summary of known and potential risks and benefits 20
6.5 Description and justification of route of administration and dosage 20
6.6 Dosages, dosage modifications and method of administration 20
6.7 Preparation and labelling of Investigational Medicinal Product 20
6.8 Drug accountability 20
7. NON-INVESTIGATIONAL PRODUCT 21
7.1 Name and description of non-investigational product(s) 21
7.2 Summary of findings from non-clinical studies 21
7.3 Summary of findings from clinical studies 21
7.4 Summary of known and potential risks and benefits 21
7.5 Description and justification of route of administration and dosage 21
7.6 Dosages, dosage modifications and method of administration 21
7.7 Preparation and labelling of Non Investigational Medicinal Product 21
7.8 Drug accountability 21
8. METHODS 22
8.1 Study parameters/endpoints 22
8.1.1 Main study parameter/endpoint 22
8.1.2 Secondary study parameters/endpoints (if applicable) 22
8.1.3 Other study parameters (if applicable) 25
8.2 Randomisation, blinding and treatment allocation 25
8.3 Study procedures 26
8.4 Withdrawal of individual subjects 27
8.4.1 Specific criteria for withdrawal (if applicable) 27

For peer review only - http://bmjopen.bmj.com/site/about/guidelines.xhtml

8.5 Replacement of individual subjects after withdrawal 27
8.6 Follow-up of subjects withdrawn from treatment 27
8.7 Premature termination of the study 27
9. SAFETY REPORTING 28
9.1 Section 10 WMO event 28
9.2 AEs, SAEs and SUSARs 28
9.2.1 Adverse events (AEs) 28
9.2.2 Serious adverse events (SAEs) 28
9.2.3 Suspected unexpected serious adverse reactions (SUSARs) 29
9.3 Annual safety report 29
9.4 Follow-up of adverse events 29
9.5 [Data Safety Monitoring Board (DSMB) / Safety Committee] 29
10. STATISTICAL ANALYSIS 30
10.1 Primary study parameter(s) 30
10.2 Secondary study parameter(s) 30
10.3 Other study parameters 31
10.4 Interim analysis (if applicable) 32
11. ETHICAL CONSIDERATIONS 33
11.1 Regulation statement 33
11.2 Recruitment and consent 33
11.3 Objection by minors or incapacitated subjects (if applicable) 33
11.4 Benefits and risks assessment, group relatedness 33
11.5 Compensation for injury 33
11.6 Incentives (if applicable) 33
12. ADMINISTRATIVE ASPECTS, MONITORING AND PUBLICATION 35
12.1 Handling and storage of data and documents 35
12.2 Monitoring and Quality Assurance 35
12.3 Amendments 35
12.4 Annual progress report 35
12.5 End of study report 35
12.6 Public disclosure and publication policy 36
13. STRUCTURED RISK ANALYSIS 37
13.1 Potential issues of concern 37
13.2 Synthesis 37
14. REFERENCES 38
Appendix 1… 42
Appendix 2… 43
Appendix 3… 45
Appendix 4… 46
Appendix 5… 47

For peer review only

LIST OF ABBREVIATIONS AND RELEVANT DEFINITIONS

**ABR**	**ABR form, General Assessment and Registration form, is the application form that is required for submission to the accredited Ethics Committee (In Dutch, ABR = Algemene Beoordeling en Registratie)**
**AE**	**Adverse Event**
**AR**	**Adverse Reaction**
**CA**	**Competent Authority**
**CCMO**	**Central Committee on Research Involving Human Subjects; in Dutch: Centrale Commissie Mensgebonden Onderzoek**
**CV**	**Curriculum Vitae**
**DSMB**	**Data Safety Monitoring Board**
**EU**	**European Union**
**EudraCT**	**European drug regulatory affairs Clinical Trials**
**GCP**	**Good Clinical Practice**
**IB**	**Investigator's Brochure**
**IC**	**Informed Consent**
**IMP**	**Investigational Medicinal Product**
**IMPD**	**Investigational Medicinal Product Dossier**
**METC**	**Medical research ethics committee (MREC); in Dutch: medisch ethische toetsing commissie (METC)**
**(S)AE**	**(Serious) Adverse Event**
**SPC**	**Summary of Product Characteristics (in Dutch: officiële productinfomatie IB1-tekst)**
**Sponsor**	**The sponsor is the party that commissions the organisation or performance of the research, for example a pharmaceutical company, academic hospital, scientific organisation or investigator. A party that provides funding for a study but does not commission it is not regarded as the sponsor, but referred to as a subsidising party.**
**SUSAR**	**Suspected Unexpected Serious Adverse Reaction**
**Wbp**	**Personal Data Protection Act (in Dutch: Wet Bescherming Persoonsgevens)**
**WMO**	**Medical Research Involving Human Subjects Act (in Dutch: Wet Medisch-wetenschappelijk Onderzoek met Mensen**

For peer review only

SUMMARY

Rationale: Moderate to severe pelvic organ prolapse symptoms can be treated with pessary or surgery. Both treatments appear to be effective, but have not been compared directly.

Hypothesis: The strategy of pessary as initial therapy is as effective as direct surgery for moderate to severe POP, but it is associated with lower costs.

Objective: The primary objective is to compare the effectiveness and cost-effectiveness of pessary versus surgery as initial treatment for moderate to severe symptomatic pelvic organ prolapse (POP) in women at two year after initiation of treatment. The secondary objective is the development of a prediction model for failure of pessary use and surgery within 2 years.

Study design: Cohort study with embedded randomized controlled trial.

Study population: Treatment naïve women with POP who present with moderate to severe symptoms.

Intervention (if applicable): Pessary therapy or vaginal POP surgery.

Main study parameters/endpoints:

Primary outcome: Global impression of improvement of POP symptoms at 24 months measured with PGI-I

Secondary outcomes:

- Changes in symptom bother and disease-specific quality of life at 12 and 24 months follow-up
- Changes of sexual function at 12 and 24 months follow-up
- Changes in general quality of life at 3, 6, 12 and 24 months of follow up
- Adverse events/complications related to both treatment strategies during the study period
- Development of prediction model to identify factors for failing of pessary and surgery.
- Costs-effectiveness analyses

Nature and extent of the burden and risks associated with participation, benefit and group relatedness: Both treatment arms are routine treatments in the Netherlands. Patients in the RCT can have the risks of surgery instead of the risks from pessary therapy.

For peer review only - http://bmjopen.bmj.com/site/about/guidelines.xhtml

1. INTRODUCTION AND RATIONALE

Problem definition

Female pelvic organ prolapse (POP) is a common problem in women that negatively affects quality of life. The estimated prevalence of symptomatic POP among women between 45-85 years of age is 8.3 - 11% [1,2]. It is current practice in the Netherlands that the general practitioner (GP) treats the majority of women with POP symptoms. Women with moderate to severe POP symptoms are often referred to a gynecologist for treatment. This study focuses at the subgroup of moderate to severe POP.

Known effective treatment options for moderate to severe POP are pessary or surgery. A pessary has proven its effectiveness in the treatment of symptomatic POP, mainly in cystocele and uterine descent. However, studies are mainly observational in nature and inherently subject to selection and indication bias [3]. In literature, outcomes of pessary therapy are mainly recorded in terms of (dis-) continuation of therapy and to a much lesser extent in terms of symptom relief. The pessary continuation rate is 60% [3]. This is confirmed by a Dutch pilot study in 65 women that showed a satisfaction with pessary in 57% of women and an operation rate of 43% at 12 months follow up [4]. In this study, 80% of women who continued pessary therapy reported much to very much improvement of their POP symptoms at 1 year follow up [4]. Reasons of discontinuation are pressure ulcer, vaginal discharge, discomfort or loss of fitting. These complications are reported to occur in up to 53% of women [5]. Half of them will decide to stop using pessary, but it is unclear which characteristics predict this outcome. Check-up of pessary therapy can be performed by either a general practitioner (GP), gynecologist or by self-management. According to a recent survey 50% percent of gynecologists involved in urogynaecology always offer self-management 40% on indication, and 10% never. Pessary therapy is inexpensive and costs are mainly related to doctor visits and treatment of side effects. In case of self-management costs might even be lower,

Surgery for POP results in much to very much improvement of symptoms in 80% of women and improvement of quality of life [6-9]. An anterior colporraphia is considered the standard procedure for a cystocele, as is the posterior colporraphia for a rectocele. For uterine descent uterus sparing techniques, like sacrospinous hysteropexy (SH) and modified Manchester-Fothergill procedure, or vaginal hysterectomy can be performed [10-12]]. Complications of POP surgery are temporary urinary retention, temporary buttock pain in case of sacrospinous hysteropexy, urinary tract infection, hematoma or dyspareunia [11]. These complications seldom lead to persistent morbidity. The most

For peer review only - http://bmjopen.bmj.com/site/about/guidelines.xhtml

common "complication" is the recurrence of symptomatic POP or de novo stress-incontinence that may lead to additional surgery, pessary therapy, or pelvic floor physiotherapy. As part of a RCT, comparing mesh with fascia plication, we found that
11% of women needed additional surgery after anterior colporraphia at 24 months follow up [7,9]. As in pessary therapy, the characteristics that predict successful or unsuccessful surgical therapy are largely unknown.

The decision which treatment option to choose depends on both patient and doctor's preferences. In our pilot survey 70% of gynecologists informed their patients about the possibility of pessary therapy, but it is unknown how many women actually received a pessary. A recent Dutch study showed that 48% of treatment-naive women preferred surgery, 36% a pessary and 16% had no preference [28]. It is therefore reasonable to assume that at least 50% of treatment naïve women with moderate to severe prolapse symptoms will have surgery as primary treatment.

Although clinical efficacy appears to favor surgery [3], the large variation in study design, outcome measurements and loss to follow up makes any comment on the best treatment option speculative. This is recognized in two recent reviews on the subject that both urge the need for randomized trials comparing surgery and pessary for POP [13,14]. Efficacy can be expressed in terms of clinical outcome but also in terms of cost-effectiveness. It is obvious that surgery (especially hospital costs) is much more expensive than pessary therapy, but the cost-effectiveness of the surgical or pessary strategy has never been assessed. Based on current cohort and case-control studies we hypothesize that a strategy of initial pessary therapy for moderate to severe POP, is more cost-effective than surgery.
We propose to perform a randomized controlled trial to generate evidence for the optimal and most cost-effective primary treatment for moderate to severe POP, including a better a priori patient selection for treatment by identifying factors of failure for pessary therapy or surgery.

Relevance
At present a national multidisciplinary guideline on the diagnosis and treatment of POP is completed. The guideline identifies the lack of evidence with respect to the best treatment option for moderate to severe prolapse, a conclusion that is confirmed by the 2013 Cochrane Collaboration review [13]. In this evidence "vacuum" both doctors and patient
preferences rule, but unfortunately these are not supported by facts. If we look at the available data the following calculation emerges.

For peer review only - http://bmjopen.bmj.com/site/about/guidelines.xhtml

About 60% of women who start pessary therapy in the specialist care setting will continue using it at one year [4,15]. Eighty percent of them will report much to very much improvement, resulting in an overall 48% much to very much improvement. The majority of the 40% of women who are not satisfied with pessary therapy will request or are offered additional surgery. After surgery 80% of women report much to very much improvement of POP symptoms [6]. Combining these percentages, women who originally started with pessary therapy may also expect 80% (48% after initial pessary treatment + 32% after additional surgery =) much to very much improvement. Based on these estimates it is expected that the outcome of both treatment strategies will eventually result in a global improvement of symptoms in 80% of women. With equal clinical outcomes of both strategies the costs needed to obtain these outcomes become crucial. With the exception of a cost calculation based on a Markov model, no direct cost-effectiveness studies on the use of pessary or surgery for POP have been performed [16]. The relevance of this project, with the high prevalence of POP worldwide, associated costs and insufficient evidence, is high. We have searched the www.clinicaltrials.gov database (3th March 2014) on similar studies (comparing pessary with surgery) but none were found.

However, if we were to prove that pessary therapy is more cost-effective then surgical treatment, this does not imply that a trial of pessary should always be undertaken. There is also insufficient evidence on which patient characteristics are associated with failure of pessary treatment or surgery (systematic review). The knowledge on how to predict which women will have a very low chance of success with pessary therapy can further improve effective treatment strategy management. This will contribute to treatment efficacy. This is not only very relevant for the hospital specialist care setting, but this knowledge can also be implemented in general practitioner practice units.

There is very limited evidence on the optimal management strategy for pessary cleaning, both in time interval as well as in who should perform the cleaning. Our study is unique and therefore relevant since self-management is advocated in the study setting. This will not only allow it to obtain data in a standardized way, but also involves the woman in her own management. This involvement is strongly advocated by two major gynecologic patient organizations ('Patienten Gynaecologie Nederland' and the 'Stichting Bekkenbodem Patienten'). These two organizations, as well as the Dutch urogynaecological consortium have identified this study to be highly relevant.

For peer review only - http://bmjopen.bmj.com/site/about/guidelines.xhtml

In line with the report "Medisch Specialistische zorg 20/20" we are heading towards integral health care in which the general physician and medical specialist will work more closely together, using the same treatment protocol for various illnesses. The information and conclusions of this trial will add level I scientific evidence to such an integral protocol and guideline for women with symptomatic POP. This will aid in a better patient selection that will need referral to the specialist. The data on patient's self-management of pessary treatment will supply information for patient instructions, which are relevant for information leaflets on the subject.

For peer review only

2. OBJECTIVES

The aim of this multicenter pragmatic cohort study with embedded randomized controlled non-inferiority trial comparing pessary therapy versus surgery is twofold:

1. To prospectively compare the effectiveness and cost-effectiveness of pessary therapy or surgery as primary treatment of moderate to severe symptomatic cystocele, uterine descent and/or rectocele in women at two year after initiation of treatment.
2. To develop a prediction model for failure of pessary use and surgery within the first 2 years.

For peer review only

For peer review only - http://bmjopen.bmj.com/site/about/guidelines.xhtml

3. STUDY DESIGN

Multicenter pragmatic cohort study with an embedded randomized controlled non-inferiority trial comparing pessary [CE 0086] therapy versus surgery including an economic evaluation. The follow up will be 24 months.

After a short (30 minutes) trial of pessary fitting before randomization into our protocol. This ensures that only women who fit both treatment options enter the randomization procedure. The trial is short and only aims at fitting, not symptom relief. For those women with an unsuccessful pessary fitting baseline characteristics will be recorded to allow analyses of this group.

See also appendix 1.

For peer review only

For peer review only - http://bmjopen.bmj.com/site/about/guidelines.xhtml

4. STUDY POPULATION

4.1 Population (base)

All women with a symptomatic POP will be included.

4.2 Inclusion criteria

In order to be eligible to participate in this study, a subject must meet all of the following criteria:

1. Women with a prolapse stage 2 or more.
2. Women with moderate to severe POP symptoms. Moderate to severe POP symptoms is defined as a prolapse domain score > 33 on the validated Dutch version of the Pelvic Floor Distress Inventory (PFDI-20) [8, 23, 24].
3. Women who have had a successful pessary fitting procedure: for the RCT.
4. Written informed consent.

4.3 Exclusion criteria

A potential subject who meets any of the following criteria will be excluded from participation in this study:

1. Prior urogynaecological (prolapse or incontinence) surgery
2. Probability of future childbearing
3. Insufficient knowledge of the Dutch language
4. Co-morbidity causing increased surgical risks at the discretion of the surgeon
5. Major psychiatric illness
6. Prior pessary use

4.4 Sample size calculation

With 198 women per group, we will have 80% power to reject the null hypothesis that pessary therapy is inferior to surgery, with a 1-sided alpha of 0.05, a non-inferiority margin of 10% and the proportion in the standard group is 80% (NQueryAdvisor). Accounting for 10% loss to follow-up we plan to randomize 436 patients.

The sample size calculation for prediction models is based on the number of failures of pessary or surgical therapy. For each potential predictor in the model we need 10-15 failures. Our pessary group sample size is 198 women. An estimated 40% (80 women) will cross over to surgery and can be regarded as failures. Our sample size is therefore sufficient

For peer review only - http://bmjopen.bmj.com/site/about/guidelines.xhtml

to develop the prediction model for failure of pessary therapy for 6 to 8 items. In the surgery group 20% of women will not be satisfied with the result of treatment. With the same sample size of 198 women, the 40 women who are dissatisfied allow us to study up to 4 potential predictive factors.

For peer review only

For peer review only - http://bmjopen.bmj.com/site/about/guidelines.xhtml

5. TREATMENT OF SUBJECTS

5.1 Investigational product/treatment

Pessary [CE 0086] therapy and surgery both are options for the treatment of a symptomatic POP. Ten large urogynecological units (university hospitals or teaching hospitals) that have worked together in previous consortium studies will participate in this multicenter trial. All participating gynaecologists have fitted at least 100 pessaries and performed more than 100 surgical POP procedures prior to the start of this study.

All pessaries are made of modern silicon material. All types of pessaries, both supportive and occlusive/space filling are allowed according to the judgment of the gynaecologist. A recent randomized trial comparing supportive (ring) and occlusive (Gelhorn) showed no differences [17]. After placing the pessary, all women will receive verbal and written instructions on the self-management of pessary therapy.

The first pessary follow up visit will always be performed by the gynaecologist. In case of self-management the frequency of cleaning is left to her personal judgment, but may not exceed 1 month. If self-management is not possible, women will be seen at 4 months intervals for pessary cleaning and vaginal inspection, preferable by their GP. In case of vaginal atrophy topical estrogens will be advised according to pharmaceutical guidelines. The diagnosis of atrophy is left to the judgment of the treating physician, since no clear definition for atrophy is available yet [18].

All surgical procedures will be performed according to our national guidelines. In this pragmatic trial the decision which technique to use is left, to the discretion of the gynaecologist, within the limitations below [19]. Cystocele repair will consist of conventional anterior colporrhaphy [9]. For uterine descent different techniques are allowed [20]. These techniques can either be uterus sparing (sacrospinous hysteropexy [10], modified Manchester-Fothergill procedure [12] or a abdominal sacrocolpopexy [9]) or a vaginal hysterectomy. Recent studies showed similar effectiveness on both anatomical and functional outcomes for these different techniques [10, 12, 21]. A coexistent stage 2 rectocele repair will be a conventional colporrhaphia posterior. All procedures are performed under general or spinal anesthesia and under antibiotics and thrombosis prophylaxis according to local protocols.

5.2 Use of co-intervention (if applicable)

Not applicable.

For peer review only - http://bmjopen.bmj.com/site/about/guidelines.xhtml

5.3 Escape medication (if applicable)

Not applicable.

For peer review only

For peer review only - http://bmjopen.bmj.com/site/about/guidelines.xhtml

6. INVESTIGATIONAL PRODUCT

6.1 Name and description of investigational product(s)

6.2 Summary of findings from non-clinical studies

Not applicable.

6.3 Summary of findings from clinical studies

Three systematic reviews of the literature were performed by four members of our Dutch urogynaecology consortium (details in appendix 2-4) that concluded:

1. Systematic review on the (cost)effectiveness of pessary use compared to surgery

There are a very limited number of comparative studies on the efficacy of surgery or pessary use for POP. The differences in study population, inclusion criteria, follow-up period, large numbers of loss to follow-up, different outcome measures makes interpretation difficult if not impossible. The two studies that presented data on functional outcome in terms of prolapsed symptom reduction were favorable for surgery (appendix 2).

2. Systematic review of factors influencing pessary fitting and continuation

A systematic review was performed to identify the satisfactory pessary fitting rate and the continuation rate of pessary use. The factors influencing these rates as well as the cross over to prolapse surgery were identified from previous studies (appendix 3).

Summarizing the results show that an estimated 75% of women will have a successful fitting and 59% will continue pessary use at variable follow-up between 3 months and 5 years. In these 18 studies, 8 factors have been tested more than 4 times as prognostic factor of successful pessary use: Stress urinary incontinence was found associated with discontinuation of pessary in 5 out of 7 studies. In 7 out of 10 studies previous prolapse surgery or hysterectomy was associated with less continuation of pessary use. Higher age was related to continuation of pessary use in 3 out of 6 studies, whereas no correlation was found in the other studies. In 1 out of 4 studies sexual activity was related to longer pessary use, whereas in 1 out of 4 related to the choice for surgery. In the two other studies no correlation was found. In one study where the prolapse in a specific vaginal compartment was related to outcome, nor cystocele was related to longer pessary use.

Parity en menopausal status and hormonal replacement were mostly not related to continued pessary use.

For peer review only - http://bmjopen.bmj.com/site/about/guidelines.xhtml

3. Review of factors influencing failure of POP surgery.

A systematic review of factors influencing failure of POP surgery was performed concerning recurrence after surgery (surgery failure). There were 1 case control study, 3 prospective studies and 6 retrospective studies. There were 2298 women included in the studies. Forty-four (44) potential risk factors have been studied, of which 12 risk factors have at least once been identified as statistically significant risk factors in a multivariate logistic regression analysis (appendix 4).

6.4 Summary of known and potential risks and benefits

The present study carries low risks for the participant. Pessary [CE 0086] or surgery is standard care for symptomatic pelvic organ prolapse. Known risks for surgery are blood loss, risk of infection, dyspareunia, urine incontinence or a recurrence of a symptomatic pelvic organ prolapse.

The benefit of the study lies in a better understanding of satisfaction and cost effectiveness.

6.5 Description and justification of route of administration and dosage

Not applicable

6.6 Dosages, dosage modifications and method of administration

Not applicable

6.7 Preparation and labelling of Investigational Medicinal Product

Not applicable

6.8 Drug accountability

Not applicable

For peer review only

7. NON-INVESTIGATIONAL PRODUCT

Not applicable.

7.1 Name and description of non-investigational product(s)

Not applicable.

7.2 Summary of findings from non-clinical studies

Not applicable.

7.3 Summary of findings from clinical studies

Not applicable.

7.4 Summary of known and potential risks and benefits

Not applicable.

7.5 Description and justification of route of administration and dosage

Not applicable.

7.6 Dosages, dosage modifications and method of administration

Not applicable.

7.7 Preparation and labelling of Non Investigational Medicinal Product

Not applicable.

7.8 Drug accountability

Not applicable.

For peer review only

For peer review only - http://bmjopen.bmj.com/site/about/guidelines.xhtml

8. METHODS

8.1 Study parameters/endpoints

8.1.1 Main study parameter/endpoint

The primary outcome of this study is the percentage of women with much or very much improvement of POP symptoms at 2 years follow-up, as measured with the Patient Global Impression of Improvement (PGI-I)scale [22].

PGI-I is a 7-point Likert scale, with scores ranging from very much worse to very much improved. Success is defined as 'much or very much' improvement.

8.1.2 Secondary study parameters/endpoints (if applicable)

1. Changes in symptom bother and quality of life at 12 and 24 months follow up.
2. Changes in sexual function at 12 and 24 months follow up.
3. Changes in general quality of life at 3, 6, 12 and 24 months.
4. Adverse events/complications related to both treatment strategies.
5. Development of prediction model to identify fail factors for pessary and surgery
6. Cost-effectiveness

8.1.3 Other study parameters (if applicable)

Baseline characteristics: Age; ethnicity; alcohol; smoking; number and mode of deliveries; menopausal status; hormone use; drug use; height; weight; co-morbidity (hypertension, diabetes mellitus, COPD, neurological disease, depression, cardiovascular disease); history of gynaecological operations; family history of prolapse; allergies, incontinence and sexual activity.

Physical examination: time, POP-Q, atrophy, stress test, blood loss, excessive discharge.

8.2 Randomisation, blinding and treatment allocation

After written informed consent is obtained, and inclusion and exclusion criteria are assessed, women will be randomly allocated in a 1:1 ratio to either treatment with a pessary or surgical treatment. Randomization will be done web based using ALEA, the software for randomization in clinical trials currently used by most studies in the Dutch consortium for studies on women's health and reproduction studies. The

For peer review only - http://bmjopen.bmj.com/site/about/guidelines.xhtml

randomization sequence will be computer generated using variable blocks of two and four, stratified for centre.

After entering the woman's initials and confirming inclusion criteria on the website, a unique number for randomization will be generated and the allocation code will be disclosed. This unique number cannot be deleted afterwards. This study will be open label because the nature of the intervention meant that masking to the intervention was not possible.

Women who attend the cohort will also be registered in ALEA.

For peer review only

8.3 Study procedures (see also appendix 5)

This study will be performed within the Dutch Urogynaecology Research Consortium, a subdivision of the Dutch Consortium for studies on women's health. Infrastructure (research nurses for counseling and data-monitoring, the use of web-based data entry), expertise on methodology and cost-effectiveness is shared.

1. Symptom bother and disease-specific quality of life are measured with the Pelvic Floor Distress Inventory (PFDI-20) and Pelvic Floor Impact Questionnaire (PFIQ) [23,24]. The validated version of the Dutch PFDI consists of five domains: pelvic organ prolapse, urinary incontinence, overactive bladder, pain, and obstructive micturition. The PFIQ measures the impact of urogenital symptoms on quality of life and consist of five domains: physical functioning, mobility, emotional health, embarrassment and social functioning.

2. Sexual function is measured with the PISQ-R. It is an international disease-specific questionnaire that measures sexual functioning in sexually active and inactive participants [26]. At this time, the Dutch translation is in progress, which will be finished in 2014.

3. Generic quality of life is measured with the EQ-5D and a questionnaire "doktersbezoek".

4. The adverse events of surgery recorded will consist of; direct peri-and postoperative complications (bleeding, pain and infection); interventions for complications; recurrent prolapse; de novo stress urinary incontinence. The adverse events of pessary recorded will consist of; discharge; pain; discomfort; bleeding; involuntary loss of pessary; de novo stress urinary incontinence.

5. The development of a prediction model is separately described in paragraph "data analyses".

6. The economic evaluation is described below.

For peer review only - http://bmjopen.bmj.com/site/about/guidelines.xhtml

ANTICIPATED COST-EFFECTIVENESS/BUDGET IMPACT

Cost differences between the two strategies are mainly the result from differences in costs associated with the initial intervention. Cost of a POP procedure is estimated at 4000 euros direct medical costs, and 4000 euros associated with lost productivity (indirect costs) if a societal perspective is used. The direct cost of pessary use is estimated at 200 euros, including costs for the pessary itself (50 euros) and consultations in the first year (150 euros). The estimated cost differences between the two strategies depend on the extent that women are (and remain) satisfied with the initial procedure (surgical or pessary): in case of dissatisfaction with the procedure, additional costs are generated by a subsequent intervention ((re-)operation, pessary, or pelvic physiotherapy). The flowchart (see appendix 1) illustrates the expected outcomes for each strategy. Based on the assumptions reflected in this flowchart, combined with approximate estimates for unit costs for POP surgery, pessary, GP and specialist visits, the anticipated impact on the annual health care budget as well as societal costs were estimated.

At present, the primary therapy for women presenting with moderate to severe POP is either surgery or pessary. The exact ratio is unknown, but is probably 50/50. If 50% women would receive primary surgery the current medical costs amount to 34 million Euros. If all women would start with pessary therapy, these costs would be 20 million euros, and the potential budget impact would be 14 million Euros/year. As it is not realistic that all women will start with pessary if this strategy proves to be successful, at 85% implementation of the pessary strategy, the annual budget impact will be around 10 million euros. The economic impact to society (including indirect (productivity) costs) will be 28 million euros and 20 million euros, at 100% and 85% implementation, respectively.

Sensitivity analyses showed, that these “base case” results are affected by estimated unit costs for POP surgery (direct and indirect costs) and the satisfaction rate for pessary, relative to surgery, but even the most conservative assumptions would lead to major cost savings for the health care budget (5 million euros) and society (15 million euros).

ECONOMIC EVALUATION

Considering the non-inferiority design of the study, we will not be able to rule out a small but acceptable difference in favor of POP surgery. Consequently, the economic

For peer review only - http://bmjopen.bmj.com/site/about/guidelines.xhtml

evaluation will be setup as a cost-effectiveness analysis, where cost-effectiveness will be expressed as costs per improvement outcome (much or very much improvement on the Patient Global Impression of Improvement (PGI-I)), and the incremental cost-effectiveness ratio as costs saved per additional case of unsatisfactory outcome. We will also perform a cost-effectiveness analysis using QALYs as health outcome, to express the difference between the two strategies in terms of costs (saved) per QALY (lost).

The economic evaluation will therefore encompass a cost-effectiveness analysis (CEA), a cost-utility analysis (CUA) as well as a budget impact analysis (BIA) from a health care budget and a societal perspective, with a time horizon between randomization and 2 years follow up. The primary outcome in the cost-effectiveness analysis will be costs per satisfactory outcome (primary clinical outcome), and the incremental cost-effectiveness ratio will reflect the costs saved per additional case of unsatisfactory outcome. As we hypothesize that pessary as a primary strategy in these patients does not result in more unsatisfactory outcomes, increased use of pessary will result in a decrease in the number of POP surgeries, and associated costs of hospital stay, recovery and (from a societal perspective) productivity loss (non-inferior strategy at lower costs).

Based on data actually observed in the trial, total costs associated with both surgery and pessary as a primary strategy will be estimated. Total costs can be divided into direct medical costs, non-medical costs and indirect costs. Direct medical costs are generated by utilization of primary or secondary health care services (including POP surgery, hospital stay, diagnostic procedures, medication). Non-medical costs are generated by travel expenses, and informal care; and indirect costs result from lost productivity due to absence from work or lost opportunity for non-paid activities. Non-medical and indirect costs are only included in the analysis from a societal perspective.

Resource utilization will be documented in the clinical report form (CRF) and complementary patient questionnaires, based on the Medical Consumption Questionnaire (MCQ) and Productivity Costs Questionnaire (PCQ) [29,30]. In patients for whom complete follow-up is not available, cost and quality-of-life data will be extrapolated using multiple imputations. Unit costs will be based on Dutch guideline

For peer review only - http://bmjopen.bmj.com/site/about/guidelines.xhtml

prices (for primary and secondary health services, informal care and lost productivity), and market prices (for medication)[31,32]

Similarly, the incremental costs per QALY gained will be estimated over a period of two years. Health state utilities to estimate QALYs will be derived from an EQ-5D measurement at discharge, as well as at follow-up assessments. Utility values for EQ-5D scores will be based on UK-estimates (Dolan, 1997). Utility scores will be linearly interpolated, assuming constant increase/decrease between subsequent assessments.

Robustness for sampling uncertainty as well as uncertainty associated with cost estimates and assumptions will be assessed in sensitivity analyses, including: Dutch health states (Lamers, 2005) instead of the UK based model in the main analyses; and varying unit costs for pertinent volumes of health care utilization (e.g. costs of POP surgery, pessary use, productivity costs).
The incremental costs and effects will be depicted in a cost effectiveness plane and cost-effectiveness acceptability curves providing information directly interpretable as the probability of one intervention being cost-effective compared to the alternative given a ceiling ratio that policy makers are willing to invest.

BIA
In a budget impact analysis, study results will be extrapolated to the national level to estimate the total impact on the health care budget per annum for the Netherlands in terms of cost reduction and health outcomes (satisfactory outcomes as well as QALYs). As economic consequences of the intervention are expected to span multiple years, this accumulation of cost (savings) will be reflected in the budget impact analyses.

The Budget Impact Analysis will be executed according to the international ISPOR guidelines [33]. This framework for creating a budget impact model includes formalized guidance about the acquisition and use of data in order to make budget projections. In addition to the societal perspective, the BIA will therefore be also report economic consequences from the perspective of the Dutch budgetary health care framework (BKZ). If the probability of an unsatisfactory outcome exceeds the non-inferiority limit, recommending pessary as primary treatment for all women is not

For peer review only - http://bmjopen.bmj.com/site/about/guidelines.xhtml

feasible, and an economic evaluation/budget impact analysis is not sensible. To estimate costs, we will follow the Handleiding Kosten onderzoek CVZ 2010.

8.4 Withdrawal of individual subjects

Subjects can leave the study at any time for any reason if they wish to do so without any consequences. The investigator can decide to withdraw a subject from the study for urgent medical reasons.

8.4.1 Specific criteria for withdrawal (if applicable)

Not applicable.

8.5 Replacement of individual subjects after withdrawal

We will not replace patients who withdrew informed consent. We will replace patients that are randomized by mistake, for example because of technical errors with online randomization.

8.6 Follow-up of subjects withdrawn from treatment

Patients withdrawn from the intervention but not from informed consent will be followed up.

8.7 Premature termination of the study

This study includes standard care, therefore it is very unlikely that unexpected complications will occur. Therefore premature termination is not applicable.

For peer review only

For peer review only - http://bmjopen.bmj.com/site/about/guidelines.xhtml

9. SAFETY REPORTING

9.1 Section 10 WMO event

In accordance to section 10, subsection 1, of the WMO, the investigator will inform the subjects and the reviewing accredited METC if anything occurs, on the basis of which it appears that the disadvantages of participation may be significantly greater than was foreseen in the research proposal. The study will be suspended pending further review by the accredited METC, except insofar as suspension would jeopardise the subjects' health. The investigator will take care that all subjects are kept informed.

9.2 AEs, SAEs and SUSARs

9.2.1 Adverse events (AEs)

Adverse events are defined as any undesirable experience occurring to a subject during the study, whether or not considered related to the study. All adverse events reported spontaneously by the subject or observed by the investigator or his staff will be recorded. During visits complaints will be questioned systematically.

9.2.2 Serious adverse events (SAEs)

A serious adverse event is any untoward medical occurrence or effect that at any dose:

- results in death;
- is life threatening (at the time of the event);
- requires hospitalisation or prolongation of existing inpatients' hospitalisation (>4 days);
- results in persistent or significant disability or incapacity;
- is a congenital anomaly or birth defect;
- Any other important medical event that may not result in death, be life threatening, or require hospitalization, may be considered a serious adverse experience when, based upon appropriate medical judgement, the event may jeopardize the subject or may require an intervention to prevent one of the outcomes listed above.

SAEs have to be reported when its occurrence appears in two days after the study operations. The investigators in participating centres should inform the coordinating investigator as soon as possible but at least the next working day.

For peer review only - http://bmjopen.bmj.com/site/about/guidelines.xhtml

For peer review only

The sponsor will report the SAEs through the web portal *ToetsingOnline* to the accredited METC that approved the protocol, within 15 days after the sponsor has first knowledge of the serious adverse events.

SAEs that result in death or are life threatening should be reported expedited. The expedited reporting will occur not later than 7 days after the responsible investigator has first knowledge of the adverse event. This is for a preliminary report with another 8 days for completion of the report.

9.2.3 Suspected unexpected serious adverse reactions (SUSARs)

Not applicable.

9.3 Annual safety report

The annual safety report will be combined with the annual progress report (see chapter 12.4).

9.4 Follow-up of adverse events

All AEs will be followed until they have abated, or until a stable situation has been reached. Depending on the event, follow up may require additional tests or medical procedures as indicated, and/or referral to the general physician or a medical specialist.
SAEs need to be reported till end of study within the Netherlands, as defined in the protocol

9.5 Data Safety Monitoring Board (DSMB)

Since both techniques are standard practice, and no major unexpected complications are foreseen, no interim analysis is planned. A Data Safety and Monitoring Board will not be installed, as both procedures are regularly used and acceptable options in current clinical practice.

For peer review only

For peer review only - http://bmjopen.bmj.com/site/about/guidelines.xhtml

10. STATISTICAL ANALYSIS

The results of the study will be reported according to the CONSORT statement.

The main outcomes will be analysed and presented according the intention-to-treat principle. Since in this pragmatic trial we expect that in the pessary strategy group 40% of women will cross over to surgery an additional per protocol analyses is foreseen. This will provide more insight in the effect of surgery after pessary therapy as compared to surgery or pessary therapy alone.

We plan a subgroup analysis for the location of the prolapse: anterior prolapse versus posterior prolapse.

10.1 Primary study parameter(s)

The primary outcome, success (much or very much improvement) or no success (a little better, no change, a little worse, much worse or very much worse) on the PGI-I will be expressed in percentage point differences. Differences between the percentages will be tested using a chi-square test. A p-value <0.05 will be considered statistically significant.

10.2 Secondary study parameter(s)

The PFDI, PFIQ and PISQ-r are all interval scales. Differences between baseline and 12 and 24 months follow up will be assessed using an independent t-test when normality can be assumed, or by non-parametric tests when the data are not normally distributed. Effect sizes will be calculated to estimate the magnitude of changes.

Differences in EQ5-D scores and “ziekteverzuim” between baseline and at 3, 6, 12 and 24 months will be assessed using t-test and further incorporated in the cost-effectiveness analyses.

Imputation statistics will be used or missing data.

(Serious) adverse events will be categorized and chi-square statistics, with calculation of relative risks when appropriate, will be applied in analyses.

Prediction model

A prediction model that uses predefined variables, as potential predictors of failure of pessary therapy, will be developed using multivariable regression analysis. Missing data will be imputed.

For peer review only - http://bmjopen.bmj.com/site/about/guidelines.xhtml

Predictors for failure derived from literature are a large genital hiatus (gh > 4 cm), women being sexually active, age > 65 years, prolapse POP-Q stage 3, previous hysterectomy. If applicable, a prediction rule for the chance of failure of pessary therapy will be constructed, which could be presented as a normogram which could be used to determine the chance of failure on pessary therapy.

Internal validity will be assessed using bootstrapping techniques; shrinkage will be applied to the parameter estimates. Model performance will be assessed with discriminative capacity and calibration. Calibration will be assessed by comparing the mean predicted probability that patients failed on pessary therapy with the mean observed probability that patients failed on pessary therapy. To do so, the total cohort will be split into ten groups based on the deciles of the predicted probability. Per group the mean predicted probability will be calculated as well as the mean observed predicted probability. Discriminative capacity of the model will be assessed with receiver operation characteristics (ROC) analysis and the area under the ROC curve (AUC).

We will also look at factors that could explain failure of surgery. Our systematic review on POP and recurrent POP after surgery showed that 6 preoperative items, eg. POP stage, age, family history, preoperative incontinence, previous POP or incontinence surgery, previous hysterectomy seems to be predictive for recurrence.

Women with previous POP, incontinence surgery or previous hysterectomy are excluded from our study, leaving 4 predefined potential predictive factors. After the 2 year follow-up has been performed, we will reconsider which factors to include in a prognostic model, based on the current literature. We will select predictors from literature with the highest predictive value, where about 1 predictor could be selected for each 10 surgery failures.

Using interaction terms the effect of a differential effect in women with a higher age (>median) or a lower age (<=median), a higher (>25) or lower BMI(<=25) will be assessed for both pessary as well as surgery failures prediction.

10.3 Other study parameters

Not applicable.

For peer review only - http://bmjopen.bmj.com/site/about/guidelines.xhtml

10.4 Interim analysis (if applicable)

Not applicable, because of the non-inferiority design with low risk and the possibility of cross over.

For peer review only

For peer review only - http://bmjopen.bmj.com/site/about/guidelines.xhtml

11. ETHICAL CONSIDERATIONS

11.1 Regulation statement

This study will be conducted according to the principles of the Declaration of Helsinki (version 10, October 2013) and in accordance with the Medical Research Involving Human Subjects Act (WMO) and other guidelines, regulations and Acts.

11.2 Recruitment and consent

Women with symptomatic pelvic organ prolapse who attend the outpatient clinic will be informed about the study by the gynaecologist or nurse. After checking the in- and exclusion criteria the women will receive verbal and written information about the study. If the woman is willing to participate she is asked to sign the informed consent. All women will undergo the pessary fitting test which is part of the standard evaluation during the first visit. All women will be contacted at a minimum interval of 1 week. Those women who failed the initial fitting will be offered surgery and attend the cohort, the women with a succesfull initial fitting will be asked to enroll in the RCT. In case the woman is willing to participate but actively opts for pessary therapy she will be provided with a pessary and enter the cohort.

11.3 Objection by minors or incapacitated subjects (if applicable)

Not applicable.

11.4 Benefits and risks assessment, group relatedness

The present study carries no risks for the participant. Pessary or surgery are standard care for symptomatic pelvic organ prolapse. The benefit of the study lies in a better understanding of satisfaction and cost effectiveness.

11.5 Compensation for injury

The sponsor/investigator has a liability insurance which is in accordance with article 7, subsection 9 of the WMO.

The sponsor (also) has an insurance which is in accordance with the legal requirements in the Netherlands (Article 7 WMO and the Measure regarding Compulsory Insurance for Clinical Research in Humans of 23th June 2003). This insurance provides cover for damage to research subjects through injury or death caused by the study.

For peer review only - http://bmjopen.bmj.com/site/about/guidelines.xhtml

1. € 450.000,-- (i.e. four hundred and fifty thousand Euro) for death or injury for each subject who participates in the Research;
2. € 3.500.000,-- (i.e. three million five hundred thousand Euro) for death or injury for all subjects who participate in the Research;
3. € 5.000.000,-- (i.e. five million Euro) for the total damage incurred by the organisation for all damage disclosed by scientific research for the Sponsor as 'verrichter' in the meaning of said Act in each year of insurance coverage.

The insurance applies to the damage that becomes apparent during the study or within 4 years after the end of the study.

11.6 Incentives (if applicable)

Not applicable.

For peer review only

12. ADMINISTRATIVE ASPECTS, MONITORING AND PUBLICATION

12.1 Handling and storage of data and documents

All data will be stored and will be coded. Only the researchers will be able to link patient ID and research code. The handling of personal data complies with the Dutch Personal Data Protection Act. After cessation of the study, patient material will be stored for a maximum of 15 years.

The case report forms and questionnaires will be filled in online. The head investigator will be able to check all the completed forms and questionnaires.

12.2 Monitoring and Quality Assurance

The monitoring will be coordinated by the Staff Member Clinical Research, quality coordinator of division women and baby en will be executed by a qualified intern monitor. This person is not involved in design and output of this research. The frequency of checking will be every year. The monitoring plan is discussed in section K of the METC dossier.

12.3 Amendments

All substantial amendments will be notified to the METC.

Non-substantial amendments will not be notified to the accredited METC, but will be recorded and filed by the sponsor.

12.4 Annual progress report

The sponsor/investigator will submit a summary of the progress of the trial to the accredited METC once a year. Information will be provided on the date of inclusion of the first subject, numbers of subjects included and numbers of subjects that have completed the trial, serious adverse events/ serious adverse reactions, other problems, and amendments.

12.5 End of study report

The investigator will notify the accredited METC of the end of the study within a period of 8 weeks. The end of the study is defined as the last patient's last visit.

In case the study is ended prematurely, the investigator will notify the accredited METC within 15 days, including the reasons for the premature termination.

For peer review only - http://bmjopen.bmj.com/site/about/guidelines.xhtml

Within one year after the end of the study, the investigator/sponsor will submit a final study report with the results of the study, including any publications/abstracts of the study, to the accredited METC.

12.6 Public disclosure and publication policy

The research findings will be published in peer reviewed journals.

For peer review only

For peer review only - http://bmjopen.bmj.com/site/about/guidelines.xhtml

13. STRUCTURED RISK ANALYSIS

Not applicable because this study is a low risk study concerning standard care.

13.1 Potential issues of concern

Not applicable.

13.2 Synthesis

Not applicable.

For peer review only

For peer review only - http://bmjopen.bmj.com/site/about/guidelines.xhtml

14. REFERENCES

1: Slieker-ten Hove MC et al. The prevalence of pelvic organ prolapse symptoms and signs and their relation with bladder and bowel disorders in a general female population. Int Urogynecol J. 2009;20(9):1037-45
2. Tegerstedt G, Maehle-Schmidt M, Nyrén O, Hammarström M. Prevalence of symptomatic pelvic organ prolapse in a Swedish population. Int Urogynecol J Pelvic Floor Dysfunct. 2005 Nov-Dec;16(6):497-503
3: Lamers BH et al. Pessary treatment for pelvic organ prolapse and health-related quality of life: a review. Int Urogynecol J. 2011;22(6):637-44
4: Broekman BM et al. Prospective pilot study in 65 women on pessary treatment as primary treatment, unpublished data
5:Broens-Oostveen WC, Mom RM, Lagro-Janssen ALM. De genitale prolaps: behnadleing en beloop in vier huisartspraktijken. Ned Tijdschr Geneeskd 2004, 148 (29); 1444-1448
6: Withagen M et al. Sacrospinous hysteropexy or modified manchester for the treatment of pelvic organ prolapse? Int J Gynaecol Obstet. 2013; Abstract Supplement
7: Vollebregt A et al. Cost-utility analysis of trocar-guided transobturator mesh versus anterior colporrhaphy for primary cystocele repair: a randomised trial. [in preparation]
8: Barber MD[1], Walters MD, Bump RC. Short forms of two condition-specific quality-of- life questionnaires for women with pelvic floor disorders (PFDI-20 and PFIQ-7). Am J Obstet Gynecol. 2005 Jul;193(1):103-13.
9: Pollard ME, Eilber KS, Anger JT. Abdominal approaches to pelvic prolapse repairs. Curr Opin Urol. 2013;23(4):306-11.
10: Dietz V et al. Vaginal surgery for uterine descent; which options do we have? A review of the literature. Int J Gynaecol Obstet. 2009;20(3):349-56
11: C. Maher, Feiner B, Baessler K, Schmid C. Surgical management of pelvic organ prolapse in women. Cochrane 2013
12: de Boer TA, Milani AL, Kluivers KB, Withagen MI, Vierhout ME. The effectiveness of surgical correction of uterine prolapse: cervical amputation with uterosacral ligament plication (modified Manchester) versus vaginal hysterectomy with high uterosacral ligament plication. Int Urogynecol J Pelvic Floor Dysfunct. 2009 Nov;20(11):1313-9
13: Bugge C et al. Pessaries (mechanical devices) for pelvic organ prolapse in women. Cochrane database syst rev, 2013;28;2:CD004010
14: van Geelen JM, Dwyer PL. Where to for pelvic organ prolapse treatment after the FDA pronouncements? A systematic review of the recent literature. Int Urogynecol J. 2013 May;24(5):707-18
15: Abdool Z et al. Prospective evaluation of outcome of vaginal pessaries versus surgery in women with symptomatic pelvic organ prolapse. Int Urogynecol J. 2011;22(3):273-8
16: Hullfish KL, Trowbridge ER, Stukenborg GJ. Treatment strategies for pelvic organ prolapse: a cost-effectiveness analysis. Int Urogynecol J. 2011 May;22(5):507-15
17: Cundiff GW, Amundsen CL, Bent AE, Coates KW, Schaffer JI, Strohbehn K, Handa VL. The PESSRI study: symptom relief outcomes of a randomized crossover trial of the ring and Gellhorn pessaries. Am J Obstet Gynecol. 2007 Apr;196(4):405.e1-8
18: Weber MA, Limpens J, Roovers JPWR. Assessment of vaginal atrophy: a review [submitted BJOG]
19: Cobra Ok-Klapper 2012. DCHG 2012
20: Detollenaere RJ, den Boon J, Kluivers KB, Vierhout ME, van Eijndhoven HW.Surgical management of pelvic organ prolapse and uterine descent in the Netherlands. Int Urogynecol J. 2013 May;24(5):781-8
21.Detollenaere RJ, den Boon J, Vierhout ME, van Eijndhoven HW. Uterus preserving surgery versus vaginal hysterectomy in treatment of uterine descent: a systematic review]. Ned Tijdschr Geneeskd. 2011;155(42):A3623

For peer review only - http://bmjopen.bmj.com/site/about/guidelines.xhtml

22: Yalcin I, Bump RC. Validation of two global impression questionnaires for incontinence. Am J Obstet Gynecol. 2003 Jul;189(1):98-101
23: Barber MD, Kuchibhatla MN, Pieper CF, Bump RC. Psychometric evaluation of 2 comprehensive condition-specific quality of life instruments for women with pelvic floor disorders. Am J Obstet Gynecol 2001;185(6):1388–1395.
24: Utomo E, Blok BF, Steensma AB, Korfage IJ. Validation of the Pelvic Floor Distress Inventory (PFDI-20) and Pelvic Floor Impact Questionnaire (PFIQ-7) in a Dutch population. Int Urogynecol J. 2014;25(4):531-44.
25: Euroquol—a new facility for the measurement of health-related quality of life. The EuroQol Group. Health Policy. 1990;16:199-208
26: Rogers RG, Rockwood TH, Constantine ML, Thakar R, Kammerer-Doak DN, Pauls RN, Parekh M, Ridgeway B, Jha S, Pitkin J, Reid F, Sutherland SE, Lukacz ES, Domoney C, Sand P, Davila GW, Espuna Pons ME. A new measure of sexual function in women with pelvic floor disorders (PFD): the Pelvic Organ Prolapse/Incontinence Sexual Questionnaire, IUGA-Revised (PISQ-IR). Int Urogynecol J. 2013 Jul;24(7):1091-103
27: Cabana MD, Rand CS, Powe NR, Wu AW, Wilson MH, Abboud PA, Rubin HR. Why don't physicians follow clinical practice guidelines? A framework for improvement. JAMA. 1999 Oct 20;282(15):1458-65
28: Thys SD, Roovers JP, Geomini PM, Bongers MY. Do patients prefer a pessary or surgery as primary treatment for pelvic organ prolapse. Gynecol Obstet Invest. 2012;74(1):6-12
29: www.imta.nl (C. Bouwmans et al. Handleiding iMTA Medical Cost Questionnaire (iMCQ). Rotterdam: iMTA, Erasmus Universiteit Rotterdam, 2013)
30: www.imta.nl (C. Bouwmans et al. Handleiding iMTA Productivity Cost Questionnaire (iPCQ). Rotterdam: iMTA, Erasmus Universiteit, 2013)
31:Hakkaart- van Roijen, S.S. Tan, C.A.M. Bouwmans: Handleiding voor Kostenonderzoek. Methoden en standaard kostprijzen voor economische evaluaties in de gezondheidszorg. CVZ 2010
32: Oostenbrink JB, Koopmanschap MA, Rutten FF. Standardisation of costs: the Dutch Manual for Costing in economic evaluations. Pharmacoeconomics 2002;20(7):443-454
33: Mauskopf JA, Sullivan SD, Annemans L et al. Principles of good practice for budget impact analysis: report of the ISPOR Task Force on good research practices--budget impact analysis. Value Health 2007;10(5):336-347 -> In BIA, klopt niet, nog vervangen door:

References (Cost) effectiveness of pessary use as compared to surgery systematic review

1: Mamik MM, Rogers RG, Qualls CR, Komesu YM. Goal attainment after treatment in patients with symptomatic pelvic organ prolapse. Am J Obstet Gynecol. 2013 Nov;209(5):488.e1-5.
2: Abdool Z, Thakar R, Sultan AH, Oliver RS. Prospective evaluation of outcome of vaginal pessaries versus surgery in women with symptomatic pelvic organ prolapse. Int Urogynecol J. 2011 Mar;22(3):273-8.
3: Lowenstein L, Gamble T, Sanses TV, van Raalte H, Carberry C, Jakus S, Pham T, Nguyen A, Hoskey K, Kenton K; Fellow's Pelvic Research Network. Changes in sexual function after treatment for prolapse are related to the improvement in body image perception. J Sex Med. 2010 Feb;7(2 Pt 2):1023-8.
4: Barber MD, Walters MD, Cundiff GW; PESSRI Trial Group. Responsiveness of the Pelvic Floor Distress Inventory (PFDI) and Pelvic Floor Impact Questionnaire (PFIQ) in women undergoing vaginal surgery and pessary treatment for pelvic organ prolapse. Am J Obstet Gynecol. 2006 May;194(5):1492-8.

References Systematic review of factors influencing pessary fitting and continuation

For peer review only - http://bmjopen.bmj.com/site/about/guidelines.xhtml

1. Abdool Z, Thakar R, Sultan AH, Oliver RS (2010) Prospective evaluation of outcome of vaginal pessaries versus surgery in women with symptomatic pelvic organ prolapse. Int Urogynecol J Pelvic Floor Dysfunct 16
2. Alperin M, Khan A, Dubina E, Tarnay C, Wu N, Pashos CL, Anger JT.Female Pelvic Med Reconstr Surg. 2013 May-Jun;19(3):142-7.Patterns of pessary care and outcomes for medicare beneficiaries with pelvic organ prolapse.
3. Bai SW, Yoon BS, Kwon JY, Shin JS, Kim SK, Park KH (2005) Survey of the characteristics and satisfaction degree of the patients using a pessary. Int Urogynecol J Pelvic Floor Dysfunct 16 (3):182–186
4. Barber MD, Walters MD, Cundiff GW (2006) Responsiveness of the Pelvic Floor Distress Inventory (PFDI) and Pelvic Floor Impact Questionnaire (PFIQ) in women undergoing vaginal surgery and pessary treatment for pelvic organ prolapse. Am JObstet Gynecol 194(5):1492–149
5. Brincat C, Kenton K, Pat FM, Brubaker L (2004) Sexual activity predicts continued pessary use. Am J Obstet Gynecol 191(1):198–200
6. Broens-Oostveen MC, Mom RM, Lagro-Janssen AL (2004)Genital prolapse; treatment and course in four general practices.Ned Tijdschr Geneeskd 148(29):1444–1448, 17
7. Chan SS, Cheung RY, Yiu KW, Lee LL, Pang AW, Chung TK. Int Urogynecol J. 2012 Aug;23(8):1027-33. Symptoms, quality of life, and factors affecting women's treatment decisions regarding pelvic organ prolapse.
8. Clemons JL, Aguilar VC, Sokol ER, Jackson ND, Myers DL (2004) Patient characteristics that are associated with continued pessary use versus surgery after 1 year. Am J Obstet Gynecol 191(1):159–164
9. Cundiff GW, Amundsen CL, Bent AE, Coates KW, Schaffer JI, Strohbehn K et al (2007) The PESSRI study: symptom relief outcomes of a randomized crossover trial of the ring and Gellhorn pessaries. Am J Obstet Gynecol 196(4):405–408
10. Fernando RJ, Thakar R, Sultan AH, Shah SM, Jones PW (2006) Effect of vaginal pessaries on symptoms associated with pelvic organ prolapse. Obstet Gynecol 108(1):93–99
11. Friedman S, Sandhu KS, Wang C, Mikhail MS, Banks E (2010) Factors influencing long-term pessary use. Int Urogynecol J Pelvic Floor Dysfunct 21(6):673–678
12. Hanson LA, Schulz JA, Flood CG, Cooley B, Tam F (2006) Vaginal pessaries in managing women with pelvic organ prolapse and urinary incontinence: patient characteristics and factors contributing to success. Int Urogynecol J Pelvic Floor Dysfunct 17(2):155–159
13. Jones K,Yang L, Lowder JL, Meyn L, Ellison R, ZyczynskiHMet al (2008) Effect of pessary use on genital hiatus measurements in women with pelvic organ prolapse. Obstet Gynecol 112(3):630–636
14. Geoffrion R, Zhang T, Lee T, Cundiff GW.Female Pelvic Med Reconstr Surg. 2013 Nov-Dec;19(6):339-45. Clinical characteristics associated with unsuccessful pessary fitting outcomes.
15. Komesu YM, Rogers RG, Rode MA, Craig EC, Gallegos KA, Montoya AR et al (2007) Pelvic floor symptom changes incpessary users. Am J Obstet Gynecol 197(6):620–626
16. Kuhn A, Bapst D, Stadlmayr W, Vits K, Mueller MD (2009) Sexual and organ function in patients with symptomatic prolapse: are pessaries helpful? Fertil Steril 91(5):1914–1918
17. Lone F1, Thakar R, Sultan AH, Karamalis G. A 5-year prospective study of vaginal pessary use for pelvic organ prolapse. Int J Gynaecol Obstet. 2011 Jul;114(1):56-9.
18. Maito JM, Quam ZA, Craig E, Danner KA, Rogers RG (2006) Predictors of successful pessary fitting and continued use in a nurse-midwifery pessary clinic. J Midwifery Womens Health 51 (2):78–84
19. Mamik MM, Rogers RG, Qualls CR, Komesu YM. Goal attainment after treatment in patients with symptomatic pelvic organ prolapse. Am J Obstet Gynecol. 2013 Nov;209(5):488.e1-5.
20. Manchana T, Bunyavejchevin S. Impact on quality of life after ring pessary use for pelvic organ prolapse. Int Urogynecol J. 2012 Jul;23(7):873-7.

For peer review only - http://bmjopen.bmj.com/site/about/guidelines.xhtml

21. Markle D, Skoczylas L, Goldsmith C, Noblett K. Patient characteristics associated with a successful pessary fitting. Female Pelvic Med Reconstr Surg. 2011 Sep;17(5):249-52.
22. Mokrzycki ML, Hatangadi SB, Zaccardi JE, Cox S. Preexisting stress urinary incontinence: a predictor of discontinuation with pessary management. J Low Genit Tract Dis. 2001 Oct;5(4):204-7.
23. Mutone MF, Terry C, Hale DS, Benson JT (2005) Factors which influence the short-term success of pessary management of pelvic organ prolapse. Am J Obstet Gynecol 193(1):89–94
24. Nguyen JN, Jones CR. Pessary treatment of pelvic relaxation: factors affecting successful fitting and continued use. J Wound Ostomy Continence Nurs. 2005;32(4):255-61; quiz 262-3.
25. Patel M, Mellen C, O'Sullivan DM, Lasala CA. (2010) Impact ofpessary use on prolapse symptoms, quality of life, and bodyimage. Am J Obstet Gynecol 18
26. Powers K, Lazarou G, Wang A, LaCombe J, Bensinger G.Greston WM et al (2006) Pessary use in advanced pelvic organprolapse. Int Urogynecol J Pelvic Floor Dysfunct 17(2):160–164.

Reference list Systematic review of factors influencing failure of POP surgery

1. Whiteside JL, Weber AM, Meyn LA, Walters MD. Am J Obstet Gynecol. 2004 Nov;191(5):1533-8.factors for prolapse recurrence after vaginal repair.
2. Diez-Itza I, Aizpitarte I, Becerro A. Int Urogynecol J Pelvic Floor Dysfunct. 2007 Nov;18(11):1317-24. Risk factors for the recurrence of pelvic organ prolapse after vaginal surgery: a review at 5 years after surgery.
3. Fialkow MF, Newton KM, Weiss NS. Int Urogynecol J Pelvic Floor Dysfunct. 2008 Nov;19(11):1483-7.Incidence of recurrent pelvic organ prolapse 10 years following primary surgical management: a retrospective cohort study.
4. Jeon MJ, Chung SM, Jung HJ, Kim SK, Bai SW. Gynecol Obstet Invest. 2008;66(4):268-73 Risk factors for the recurrence of pelvic organ prolapse.
5. Dällenbach P, Jungo Nancoz C, Eperon I, Dubuisson JB, Boulvain M. Int Urogynecol J. 2012 Jan;23(1):35-41. Incidence and risk factors for reoperation of surgically treated pelvic organ prolapse.
6. Weemhoff M, Vergeldt TF, Notten K, Serroyen J, Kampschoer PH, Roumen FJ. Int Urogynecol J. 2012 Jan;23(1):65-71. Avulsion of puborectalis muscle and other risk factors for cystocele recurrence: a 2-year follow-up study.
7. Tegerstedt G1, Hammarström M. Acta Obstet Gynecol Scand. 2004 Aug;83(8):758-63. Operation for pelvic organ prolapse: a follow-up study.
8. Medina CA, Candiotti K, Takacs P. Int J Gynaecol Obstet. 2008 May;101(2):184-7. Wide genital hiatus is a risk factor for recurrence following anterior vaginal repair.
9. Salvatore S1, Athanasiou S, Digesu GA, Soligo M, Sotiropoulou M, Serati M, Antsaklis A, Milani R. Neurourol Urodyn. 2009;28(4):301-4. doi: 10.1002/nau.20639. Identification of risk factors for genital prolapse recurrence.
10. Kawasaki A, Corey EG, Laskey RA, Weidner AC, Siddiqui NY, Wu JM. J Reprod Med. 2013 May-Jun;58(5-6):195-9. Obesity as a risk for the recurrence of anterior vaginal wall prolaps after anterior colporrhaphy.

For peer review only - http://bmjopen.bmj.com/site/about/guidelines.xhtml

Study protocol

Appendix 1:

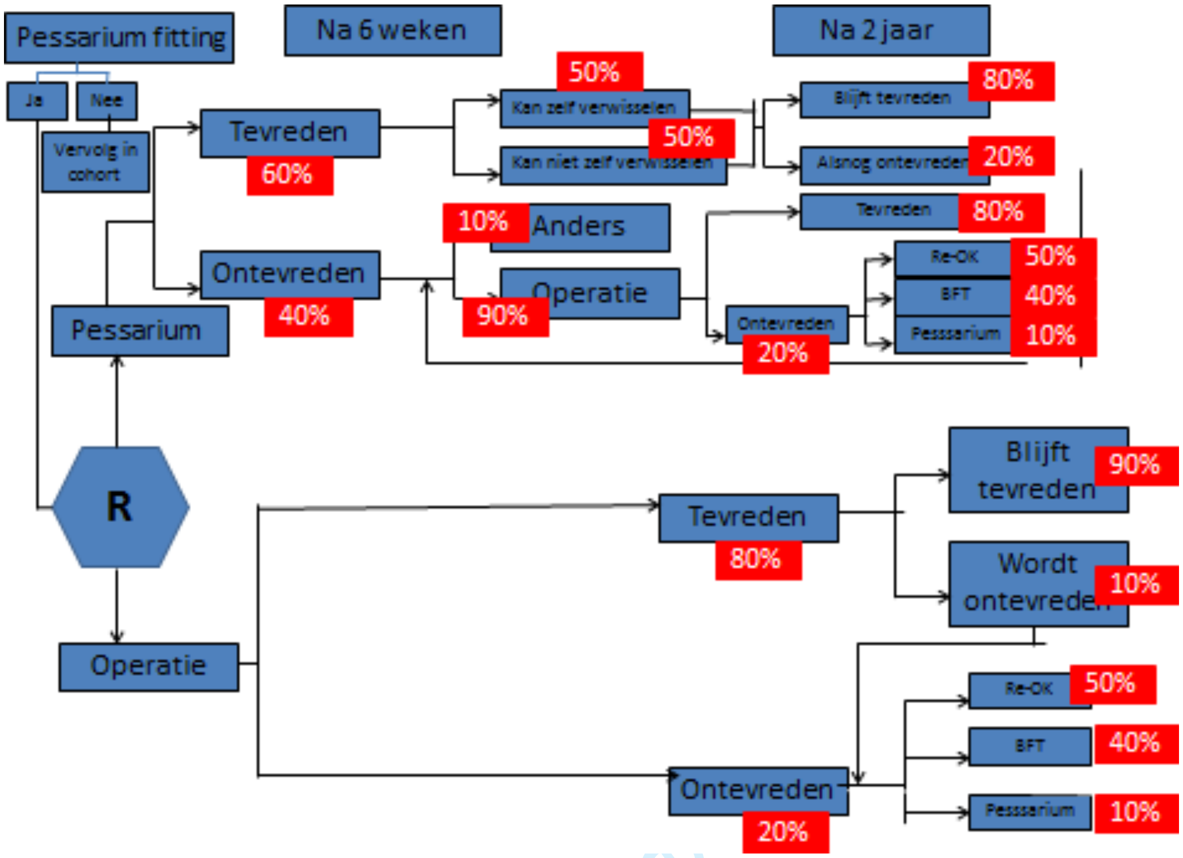

ew only

Study protocol

Appendix 2 Review on (Cost) effectiveness of pessary use as compared to surgery:

For peer review only

For peer review only - http://bmjopen.bmj.com/site/about/guidelines.xhtml

Study protocol

Reference	Study type	Characteristics	Intervention (I)	Controls (C)	Outcome measures and follow-up time	Results
Mamik, 2012<br><br>AJOG 2013:209:488	Design<br>Case-control<br><br>N = 100<br><br>Country<br>US	Aim: compare goal achievement and global improvement between pessary and surgery for POP stage ≥2.<br><br>Inclusion criteria: >18 year old, read and write in English<br><br>Exclusion: not given	Vaginal pessary<br>N = 50	Prolapse surgery<br>N = 50	Primary outcome:<br>Goal attainment<br><br>Secondary:<br>PGI-I<br>PFDI-20<br>PISQ-12<br>Body Image scale<br><br>Follow-up:<br>3 months	Primary outcome:<br>Goal attainment sign. higher score after surgery (8.6 vs 6.4)<br><br>Secondary outcomes<br>PGI-I sign (p=0.04) better improvement after surgery (2.4 vs 1.9 points)<br>PFDI-20 sign (p=0.02) higher change (89 vs 43 points)<br>PISQ-12 and BIS no sign difference<br><br>Additional: 10% crossed over from pessary to surgery within 3 months and 10% referred from surgery after they had been selected as eligible.<br>No follow-up in pessary group is 40% (20/50) and surgery 30% (15/50)
Abdool, 2011	Design<br>Cohort study<br><br>N total = 554<br><br>Country:<br>UK	Aim of the study<br>to evaluate and compare the effectiveness of pessaries and surgery in women with symptomatic pelvic organ prolapse.<br><br>Inclusion criteria<br>- Women referred to a specialist urogynaecology unit with symptomatic POP between June 2002 and May 2007<br><br>Exclusion criteria<br>- Subjects fitted with pessaries for urinary incontinence and those who had concomitant urinary incontinence surgery (e.g. TVT)<br>- Subjects who started in the pessary group but subsequently requested surgery were excluded from	Intervention<br>vaginal pessary<br><br>N = 359	Controls<br>surgery<br><br>N = 195	Primary outcomes:<br>Sheffield POP questionnaire (SPS-Q)<br><br>Secundary outcomes:<br>None<br><br>Follow up:<br>For the surgery and pessary groups 14 months (SD 6.14)<br>and 12 months (SD 3.1), respectively.	Primary outcomes:<br>No difference in functional outcome after 1 year follow-up between groups<br><br>Additional:<br>Only 45% in pessary group en 55% in surgery group responded at 12 months<br>In pessary group 24.7% (89/359) crossed to surgery but were not analyzed<br>In pessary group 7.3% stopped because of other reasons.<br>Selection and patient preference bias<br>The mean age was significantly higher in the pessary group compared to the surgery group (68.4 +/− 13.08 vs 60.4 +/−12.25 years, respectively).

For peer review only - http://bmjopen.bmj.com/site/about/guidelines.xhtml

		**analysis in both the surgery and pessary group.**				

For peer review only - http://bmjopen.bmj.com/site/about/guidelines.xhtml

**Lowenstein 2010**<br><br>**J Sex Med 2010; 7: 1023-**<br><br>**28**	**Design**<br>**Cohort study**<br>**N= 235**<br><br>**Country**<br>**US**	**Aim of the study**<br>**First to evaluate patient-reported outcome, POP symptoms, sexual functioning and body image following treatment of POP. Second to compare surgery with pessary**<br><br>**Inclusion: ≥18 year, ≥ satge 2 POP, complete questionnaire at baseline and at ≥6 months follow up**<br><br>**Exclusion: recurrent UTI, peripheral neuropathy, using pessary at initial presentation or POP surgery < 6 months prior to presentation**	**Intervention**<br>**N = 202 surgery**	**Controls**<br>**N = 33 pessary**	**Primary outcomes**<br>**PFDI-20**<br>**PISQ-12**<br>**Modified Body Image scale**<br><br>**All at six months follow-up**	**Results**<br>**After multivariate analyses, including type of intervention, BMI and difference in Body image were associated with change in total PISQ (sexual functioning) score**<br><br>**In the pessary group there was no significant improvement in sexual functioning as compared to surgery (-2.5 versus +11.5)**<br><br>**Additional:**<br>**No figures presented for pessary and surgery group, with exemption of the Sexual functioning (PISQ-12) result above.**
**Barber, 2006**	**Design**<br>Case-control study<br><br>**N total = 106**<br><br>**Country:** USA	**Aim of the study**<br>**to evaluate the responsiveness of the Pelvic Floor Distress Inventory (PFDI) and Pelvic Floor Impact Questionnaire (PFIQ) in women with pelvic organ prolapse undergoing surgical and nonsurgical management.**<br><br>**Inclusion criteria**<br>**Surgery group:**<br>**Stage III or IV prolapse, were at least 18 years, and scheduled for vaginal prolapse repair.**<br>**Pessary group:**<br>**women with symptomatic pelvic organ prolapse of stage II or greater. (Pessri trial)**<br><br>**Exclusion criteria**<br>**Surgery group:**<br>**- mentally or physically incapable of completing the questionnaires.**<br>**Pessary group:**<br>**- were pregnant, were currently using a pessary, or had vaginal agglutination**	**Intervention**<br>**Pessary in women with stage II or greater POP**<br><br>**N = 42**	**Controls**<br>**Surgery in women with stage III or greater POP**<br><br>**N = 64**	**Primary outcomes:**<br>**PFDI and PFIQ**<br><br>**Secundary outcomes:**<br><br>**Follow up:**<br>**3 months (Pessary group) or 6 months (Surgery group) after initiation of treatment.**	**Primary outcomes:**<br><br>**After controlling for preoperative prolapse stage and baseline HRQOL scores, subjects in the Surgery group had significantly greater improvement in each of the scales of the PFDI and the prolapse and urinary scales of the PFIQ than did the Pessary group.**<br><br>**Scores from each of the scales of the PFDI improved by 14 to 15 points more on average after treatment in the Surgery group than those of the Pessary group (P < .01 for each) after adjusting for the above baseline differences.**<br><br>**Similarly, for the prolapse and urinary scales of the PFIQ, scores improved 13 and 17 points more, respectively, in the Surgery group than the Pessary group after treatment. (P < .05 for each).**<br><br>**Four of 64 (6%) of subjects in the Surgery group had recurrent prolapse develop beyond the hymen by 6 months after surgery. No subjects underwent reoperation for recurrent prolapse during the study period.**<br><br>**Additional:**<br><br>**Difference in follow up**<br>**Selection bias**

For peer review only

		**that precluded pessary insertion.**				

For peer review only - http://bmjopen.bmj.com/site/about/guidelines.xhtml

Appendix 3 Review on risk factors for failure of pessaries:

study		n	FU period	success initial	continued pes	stopped pes	cross over surgery	predict fit	predict contin
Wu 1997	retrospective	110	3 yrs	81 (74%)	43 (39%)		at least 5	nee	ja
Mokrzycki 2001	retrospective	42	3 mth		24 (57%)	18 (43%)	nr	nee	ja
Clemons 2004	prospective	100	1 yr	73 (73%)	43 (43%)		16/73 (27%)	nee	ja
Brincat 2004	retrospective	169	2,5 yr	144 (85%)	82 (60%)	54 (40%)	32/144 (22%)	nee	ja
Mutone 2004	retrospective	407	3 wks	168 (41%)		96 (57%)	nr	ja	ja
Powers 2006	retrospective	32	1 yr			20 (62%)	12 (38%)	nee	ja
Broens-Oostveen 2004	retrospective	192	9 yr			107 (52%)	nr	nee	ja
Nguyen 2005	retrospective	130	1 yr	111 (82%)	85 (65%)		20 (15%)	ja	ja
Hanson 2005	retrospective	1216	3 mth	1043 (86%)	744 (71%)		nr	nee	ja
Fernando 2006	prospective	203	4 mth	nr	153 (75%)		28 (14%)	nee	ja
Barber 2006	prospective	42	3 mth	nr		0	nr	nee	nee
Maito 2006	retrospective	120	6 mth	106 (88%)	92 (77%)		9 (8%)	ja	ja
Cundiff 2007	prospective	85	1 yr	65/71 (92%)			5 (6%)	nee	nee
Komesu 2007	prospective	64	6-12 mth		36 (56%)	28 (44%)	2 (3%)	nee	ja
Jones 2008	prospective	90	3 mth		42 (47%)		nr	nee	ja
Kuhn 2009	prospective	71	3 mth		32 (45%)		nr	nee	nee
Sarma 2009	retrospective	120	1 mth		45 (38%)		18 (15%)	nee	nee
Friedman 2010	retrospective	150	1 yr		115 (77%)	35 (23%)	25 (17%)	nee	ja
Patel 2010	prospective	65	3 mth	54 (83%)	54 (83%)		5 (8%)	nee	nee
Abdool 2010	prospective	359	1 yr	296 (83%)	243 (68%)		31 (9%)	nee	nee
Lone 2011	prospective	246	5 yr	187 (76%)	130 (53%)		70 (28%)	nee	nee
Markle 2011	retrospective	158	1 week	92 (59%)			nr	ja	nee
Chan 2012	prospective	197	1 yr	138 (70%)		59 (30%)	nr	nee	ja
Manchana 2012	retrospective	126	1 yr	77 (61%)	64 (51%)		5 (4%)	nee	nee
Mamik 2013	prospective	50	3 mth				5 (10%)	nee	nee
Geoffrion 2013	retrospective	101	4 wks	79 (78%)			nr	nee	ja
Alperin 2013	retrospective	4019	9 years				12% 1 yr, 24% 9 yrs	nee	nee
Succesfull fitting		3589		2714(76%)					
Continuation		3381			2027 (60%)				

For peer review only - http://bmjopen.bmj.com/site/about/guidelines.xhtml

Appendix 4 Review on risk factors for failure of surgery:

Risk factor	Investigated:	Significant:
Preoperative stage	8	5
Age	8	2
Obesity	7	0
Parity	5	0
Constipation	5	0
Pulmonary disease	5	0
Number of sites involved preoperative	4	1
Menopausal status	4	0
Hysterectomy status	4	0
Concomitent surgery	3	1
Family history	3	1
Complicated delivery	3	0
Diabetes	3	0
Smoking	3	0
Previous incontinence and/or prolapse surgery	2	2
Hiatus genitalis	2	1
Weight	2	1
Any incontinence preoperative	2	1
Delivery mode	2	0
Vaginal delivery	2	0
Hormone replacement therapy	2	0
Previous prolapse surgery	2	0
Surgeons experience	2	0
Abcense of posterior repair	1	1
Sexual activity	1	1
Levator defect	1	1
Height	1	0
Birth weight	1	0
Age at last delivery	1	0
Site of most advanced prolapse	1	0
Surgical approach	1	0
Use of Mesh	1	0
Previous incontinence surgery	1	0
Previous pelvic floor surgery or hysterectomy	1	0
Abdominal hernias	1	0
Cardiovascular disease	1	0
Intense physical exercise	1	0
Heavy lifting	1	0
Heavy lifting or constipation	1	0
Levator muscle contraction	1	0
Weight of the uterus	1	0
Postoperative complications	1	0
Incomplete emptying of bladder	1	0
Fecal incontinence	1	0

For peer review only

Study protocol

Appendix 5 tabel bezoeken, tijdstippen, onderzoeken

Chirurgie en cohort

Contact	Bezoek arts	POPQ	Eq5d doktersbezoek werkverzuim	PFIQ PFDI PISQ PGII PGIS
1. Eerste bezoek	X	X	Eq5D	X (zonder PGII)
2. 6 weken	X	X		
3. 3 maanden			X	
4. 6 maanden			X	
5. 12 maanden	X	X	X	X
6. 24 maanden	X	X	X	X

Pessarium met zelfmanagement

Contact	Bezoek arts	POPQ	Eq5d doktersbezoek werkverzuim	PFIQ PFDI PISQ PGII PGIS
1. Eerste bezoek	X	X	Eq5D	X (zonder PGII)
2. 6 weken	X	X		
3. 3 maanden			X	
4. 6 maanden			X	
5. 12 maanden	X	X	X	X
6. 24 maanden	X	X	X	X

For peer review only

Pessarium zonder zelfmanagement

Contact	Bezoek arts	POPQ	Ringcontrole	Eq5d doktersbezoek werkverzuim	PFIQ PFDI PISQ PGII PGIS
1. Eerste bezoek	X	X		Eq5D	X (zonder PGII)
2. 6 weken	X	X			
3. 3 maanden				X	
4. 4 maanden	X		X		
5. 6 maanden				X	
6. 8 maanden	X		X		
7. 12 maanden	X	X	X	X	X
8. 16 maanden	X		X		
9. 20 maanden	X		X		
10. 24 maanden	X	X	X	X	X

For peer review only

1.2 Final study protocol

For peer review only

Pessary or surgery for symptomatic pelvic organ prolapse

Version 1.~~21~~ 22 ~~April 2017~~February 2018

For peer review only - http://bmjopen.bmj.com/site/about/guidelines.xhtml

Study protocol

PROTOCOL TITLE 'Pessary or surgery for symptomatic pelvic organ prolapse'

**Protocol ID**	**~~2017~~ 2018 / 1.~~21~~22**
**Short title**	**Pessary or surgery for symptomatic pelvic organ prolapse**
**EudraCT number**	***Not applicable***
**Version**	**1.~~21~~22**
**Date**	**April 2017**
**Coordinating investigator/project leader**	**Prof. Dr. C.H. van der Vaart, gynaecologist<br>University Medical Centre Utrecht**
**Principal investigator(s) (in Dutch: hoofdonderzoeker/ uitvoerder)**	**Dr. A. Vollebregt, gynaecologist<br>Spaarne Hospital<br><br>M.K. van de Waarsenburg, MD<br>University Medical Centre Utrecht**
**Multicenter: per site**	**Prof. Dr. C.H. van der Vaart, UMC Utrecht / Bergman Clinics Bilthoven<br>Dr. A.L. Milani, Reinier de Graaf Gasthuis<br>Dr. A. Vollebregt, Spaarne Hospital Hoofddorp<br>Dr. J.P.W.R. Roovers, Academic Medical Center / Bergman Clinics Amsterdam<br>Dr. K.B. Kluivers, Radboud UMC Nijmegen<br>Dr. V. Dietz, Catharina Hospital Eindhoven<br>Dr. H.W.F. van Eijndhoven, Isala Clinic Zwolle<br>Dr. M.M.A. Vernooij, Sint Antonius Hospital**

For peer review only
For peer review only - http://bmjopen.bmj.com/site/about/guidelines.xhtml

Study protocol

	Nieuwegein<br>Dr. R.P. Schellart, Kennemer Gasthuis Haarlem<br>Drs. A.M.W. Broekman, Sint Franciscus Gasthuis Rotterdam<br>Drs. J. van Bavel, Amphia Hospital Breda<br>Dr. M. Weemhoff, Atrium MC Heerlen<br>Dr. ~~G. Link~~W.A. Spaans, Academisch ziekenhuis Maastricht<br>Dr. J.M. van der Ploeg, Martini ziekenhuis Groningen<br>Dr. D. Massop-Helmink, MST Enschede<br>Dr. J.M. Sikkema, ZGT Almelo / Hengelo<br>Dr. L. van der Voet, Deventer ziekenhuis<br>Dr. J.P. de Bruin, Jeroen Bosch ziekenhuis<br>Dr. C. Zeijl, Amstelland ziekenhuis<br>Dr. G.G. Bon, Tergooi ziekenhuis<br>Dr. S. de Vos – Stekelenburg, Albert Schweitzer ziekenhuis<br>Drs. K.L. Bos, Canisius Wilhelmina ziekenhuis<br>Prof. Dr. M.Y. Bongers – Maxima Medisch Centrum<br>Dr. W. Hermes – MCH-Bronovo<br>Dr. E. Janszen – OLVG<br>Dr. T. Huisman - HAGA
Sponsor (in Dutch: verrichter/opdrachtgever)	Prof. Dr. C.H. van der Vaart, gynaecologist<br>University Medical Centre Utrecht
Subsidising party	ZonMw Project nr 837002525
Independent expert (s)	Dr. R.P. Zweemer<br>University Medical Centre Utrecht

For peer review only

**Laboratory sites <*if applicable*>**	***Not applicable***
**Pharmacy <*if applicable*>**	***Not applicable***

For peer review only

PROTOCOL SIGNATURE SHEET

Name	Signature	Date
**Head of Department:**<br><br>**Prof. Dr. B. Veersema**<br>**Department of Reproductive Medicine and Gynaecology**<br>**University Medical Centre Utrecht**		
**Coordinating Investigator/Project leader:**<br><br>**Prof. Dr. C.H. van der Vaart, gynaecologist**		
**M.K. van de Waarsenburg, MD**<br>**University Medical Centre Utrecht**		

For peer review only

TABLE OF CONTENTS

1. INTRODUCTION AND RATIONALE 64
Problem definition 64
Relevance 65
2. OBJECTIVES 68
3. STUDY DESIGN 69
4. STUDY POPULATION 70
4.1 Population (base) 70
4.2 Inclusion criteria 70
4.3 Exclusion criteria 70
4.4 Sample size calculation 70
5. TREATMENT OF SUBJECTS 72
5.1 Investigational product/treatment 72
5.2 Use of co-intervention (if applicable) 73
5.3 Escape medication (if applicable) 73
6. INVESTIGATIONAL PRODUCT 74
6.1 Name and description of investigational product(s) 74
6.2 Summary of findings from non-clinical studies 74
6.3 Summary of findings from clinical studies 74
6.4 Summary of known and potential risks and benefits 75
6.5 Description and justification of route of administration and dosage 75
6.6 Dosages, dosage modifications and method of administration 75
6.7 Preparation and labelling of Investigational Medicinal Product 75
6.8 Drug accountability 75
7. NON-INVESTIGATIONAL PRODUCT 76
7.1 Name and description of non-investigational product(s) 76
7.2 Summary of findings from non-clinical studies 76
7.3 Summary of findings from clinical studies 76
7.4 Summary of known and potential risks and benefits 76
7.5 Description and justification of route of administration and dosage 76
7.6 Dosages, dosage modifications and method of administration 76
7.7 Preparation and labelling of Non Investigational Medicinal Product 76
7.8 Drug accountability 76
8. METHODS 77
8.1 Study parameters/endpoints 77
8.1.1 Main study parameter/endpoint 77
8.1.2 Secondary study parameters/endpoints (if applicable) 77
8.1.3 Other study parameters (if applicable) 77
8.2 Randomisation, blinding and treatment allocation 77
8.3 Study procedures (see also appendix 5) 78

For peer review only - http://bmjopen.bmj.com/site/about/guidelines.xhtml

8.4 Withdrawal of individual subjects 82
8.4.1 Specific criteria for withdrawal (if applicable) 82
8.5 Replacement of individual subjects after withdrawal 82
8.6 Follow-up of subjects withdrawn from treatment 82
8.7 Premature termination of the study 82
9. SAFETY REPORTING9.1 Section 10 WMO event 83
9.2 AEs, SAEs and SUSARs 83
9.2.1 Adverse events (AEs) 83
9.2.2 Serious adverse events (SAEs) 83
9.2.3 Suspected unexpected serious adverse reactions (SUSARs) 84
9.3 Annual safety report 84
9.4 Follow-up of adverse events 84
9.5 Data Safety Monitoring Board (DSMB) 84
10. STATISTICAL ANALYSIS 85
10.1 Primary study parameter(s) 85
10.2 Secondary study parameter(s) 85
10.3 Other study parameters 87
10.4 Interim analysis (if applicable) 87
11. ETHICAL CONSIDERATIONS 88
11.1 Regulation statement 88
11.2 Recruitment and consent 88
11.3 Objection by minors or incapacitated subjects (if applicable) 88
11.4 Benefits and risks assessment, group relatedness 88
11.5 Compensation for injury 88
11.6 Incentives (if applicable) 89
12. ADMINISTRATIVE ASPECTS, MONITORING AND PUBLICATION 90
12.1 Handling and storage of data and documents 90
12.2 Monitoring and Quality Assurance 90
12.3 Amendments 90
12.4 Annual progress report 90
12.5 End of study report 90
12.6 Public disclosure and publication policy 91
13. STRUCTURED RISK ANALYSIS 92
13.1 Potential issues of concern 92
13.2 Synthesis 92
14. REFERENCES 93
Appendix 1: 97
Appendix 2 98
Appendix 3: 102
Appendix 4 103
Appendix 5: 104

For peer review only - http://bmjopen.bmj.com/site/about/guidelines.xhtml

LIST OF ABBREVIATIONS AND RELEVANT DEFINITIONS

**ABR**	**ABR form, General Assessment and Registration form, is the application form that is required for submission to the accredited Ethics Committee (In Dutch, ABR = Algemene Beoordeling en Registratie)**
**AE**	**Adverse Event**
**AR**	**Adverse Reaction**
**CA**	**Competent Authority**
**CCMO**	**Central Committee on Research Involving Human Subjects; in Dutch: Centrale Commissie Mensgebonden Onderzoek**
**CV**	**Curriculum Vitae**
**DSMB**	**Data Safety Monitoring Board**
**EU**	**European Union**
**EudraCT**	**European drug regulatory affairs Clinical Trials**
**GCP**	**Good Clinical Practice**
**IB**	**Investigator's Brochure**
**IC**	**Informed Consent**
**IMP**	**Investigational Medicinal Product**
**IMPD**	**Investigational Medicinal Product Dossier**
**METC**	**Medical research ethics committee (MREC); in Dutch: medisch ethische toetsing commissie (METC)**
**(S)AE**	**(Serious) Adverse Event**
**SPC**	**Summary of Product Characteristics (in Dutch: officiële productinfomatie IB1-tekst)**
**Sponsor**	**The sponsor is the party that commissions the organisation or performance of the research, for example a pharmaceutical company, academic hospital, scientific organisation or investigator. A party that provides funding for a study but does not commission it is not regarded as the sponsor, but referred to as a subsidising party.**
**SUSAR**	**Suspected Unexpected Serious Adverse Reaction**
**Wbp**	**Personal Data Protection Act (in Dutch: Wet Bescherming Persoonsgevens)**
**WMO**	**Medical Research Involving Human Subjects Act (in Dutch: Wet Medisch-wetenschappelijk Onderzoek met Mensen**

For peer review only - http://bmjopen.bmj.com/site/about/guidelines.xhtml

SUMMARY

Rationale: Moderate to severe pelvic organ prolapse symptoms can be treated with pessary or surgery. Both treatments appear to be effective, but have not been compared directly.

Hypothesis: The strategy of pessary as initial therapy is as effective as direct surgery for moderate to severe POP, but it is associated with lower costs.

Objective: The primary objective is to compare the effectiveness and cost-effectiveness of pessary versus surgery as initial treatment for moderate to severe symptomatic pelvic organ prolapse (POP) in women at two year after initiation of treatment. The secondary objective is the development of a prediction model for failure of pessary use and surgery within 2 years.

Study design: Cohort study with embedded randomized controlled trial.

Study population: Treatment naïve women with POP who present with moderate to severe symptoms.

Intervention (if applicable): Pessary therapy or vaginal POP surgery.

Main study parameters/endpoints:

Primary outcome: Global impression of improvement of POP symptoms at 24 months measured with PGI-I

Secondary outcomes:

- Changes in symptom bother and disease-specific quality of life at 12 and 24 months follow-up
- Changes of sexual function at 12 and 24 months follow-up
- Changes in general quality of life at 3, 6, 12 and 24 months of follow up
- Adverse events/complications related to both treatment strategies during the study period
- Development of prediction model to identify factors for failing of pessary and surgery.
- Costs-effectiveness analyses

Nature and extent of the burden and risks associated with participation, benefit and group relatedness: Both treatment arms are routine treatments in the Netherlands. Patients in the RCT can have the risks of surgery instead of the risks from pessary therapy.

For peer review only

1. INTRODUCTION AND RATIONALE

Problem definition

Female pelvic organ prolapse (POP) is a common problem in women that negatively affects quality of life. The estimated prevalence of symptomatic POP among women between 45-85 years of age is 8.3 - 11% [1,2]. It is current practice in the Netherlands that the general practitioner (GP) treats the majority of women with POP symptoms. Women with moderate to severe POP symptoms are often referred to a gynecologist for treatment. This study focuses at the subgroup of moderate to severe POP.

Known effective treatment options for moderate to severe POP are pessary or surgery. A pessary has proven its effectiveness in the treatment of symptomatic POP, mainly in cystocele and uterine descent. However, studies are mainly observational in nature and inherently subject to selection and indication bias [3]. In literature, outcomes of pessary therapy are mainly recorded in terms of (dis-) continuation of therapy and to a much lesser extent in terms of symptom relief. The pessary continuation rate is 60% [3]. This is confirmed by a Dutch pilot study in 65 women that showed a satisfaction with pessary in 57% of womenand an operation rate of 43% at 12 months follow up [4]. In this study, 80% of women who continued pessary therapy reported much to very much improvement of their POP symptomsat 1 year follow up [4]. Reasons of discontinuation are pressure ulcer, vaginal discharge, discomfort or loss of fitting. These complications are reported to occur in up to 53% of women [5]. Half of them will decide to stop using pessary, but it is unclear which characteristics predict this outcome. Check-up of pessary therapy can be performed by either a general practitioner (GP), gynecologist or by self-management. According to a recent survey 50% percent of gynecologists involved in urogynaecology always offer self- management 40% on indication, and 10% never. Pessary therapy is inexpensive and costs are mainly related to doctor visits and treatment of side effects. In case of self-management costs might even be lower,

Surgery for POP results in much to very much improvement of symptoms in 80% of women and improvement of quality of life [6-9]. An anterior colporraphia is considered the standard procedure for a cystocele, as is the posterior colporraphia for a rectocele. For uterinedescent uterus sparing techniques, like sacrospinous hysteropexy (SH) and modified Manchester-Fothergill procedure, or vaginal hysterectomy can be performed [10-12]]. Complications of POP surgery are temporary urinary retention, temporary buttock pain in case of sacrospinous hysteropexy, urinary tract infection, hematoma or dyspareunia [11]. These complications seldom lead to persistent morbidity. The most

For peer review only - http://bmjopen.bmj.com/site/about/guidelines.xhtml

common "complication" is the recurrence of symptomatic POP or de novo stress- incontinence that may lead to additional surgery, pessary therapy, or pelvic floor physiotherapy. As part of a RCT, comparing mesh with fascia plication, we found that
11% of women needed additional surgery after anterior colporraphia at 24 months follow up [7,9]. As in pessary therapy, the characteristics that predict successful or unsuccessfulsurgical therapy are largely unknown.

The decision which treatment option to choose depends on both patient and doctor's preferences. In our pilot survey 70% of gynecologists informed their patients about the possibility of pessary therapy, but it is unknown how many women actually received a pessary. A recent Dutch study showed that 48% of treatment-naive women preferred surgery, 36% a pessary and 16% had no preference [28]. It is therefore reasonable to assume that at least 50% of treatment naïve women with moderate to severe prolapse symptoms will have surgery as primary treatment.

Although clinical efficacy appears to favor surgery [3], the large variation in study design, outcome measurements and loss to follow up makes any comment on the best treatment option speculative. This is recognized in two recent reviews on the subject that both urge the need for randomized trials comparing surgery and pessary for POP [13,14]. Efficacy can be expressed in terms of clinical outcome but also in terms of cost-effectiveness. It is obvious that surgery (especially hospital costs) is much more expensive than pessary therapy, butthe cost-effectiveness of the surgical or pessary strategy has never been assessed. Based on current cohort and case-control studies we hypothesize that a strategy of initial pessary therapy for moderate to severe POP, is more cost-effective than surgery.
We propose to perform a randomized controlled trial to generate evidence for the optimal and most cost-effective primary treatment for moderate to severe POP, including a better a priori patient selection for treatment by identifying factors of failure for pessary therapy or surgery.

Relevance

At present a national multidisciplinary guideline on the diagnosis and treatment of POP is completed. The guideline identifies the lack of evidence with respect to the best treatment option for moderate to severe prolapse, a conclusion that is confirmed by the 2013 Cochrane Collaboration review [13]. In this evidence "vacuum" both doctors and patient preferences rule, but unfortunately these are not supported by facts. If we look at the available data the following

For peer review only - http://bmjopen.bmj.com/site/about/guidelines.xhtml

calculation emerges.

About 60% of women who start pessary therapy in the specialist care setting will continue using it at one year [4,15]. Eighty percent of them will report much to very much improvement, resulting in an overall 48% much to very much improvement. The majority ofthe 40% of women who are not satisfied with pessary therapy will request or are offered additional surgery. After surgery 80% of women report much to very much improvement ofPOP symptoms [6]. Combining these percentages, women who originally started withpessary therapy may also expect 80% (48% after initial pessary treatment + 32% afteradditional surgery =) much to very much improvement. Based on these estimates it isexpected that the outcome of both treatment strategies will eventually result in a globalimprovement of symptoms in 80% of women. With equal clinical outcomes of both strategiesthe costs needed to obtain these outcomes become crucial. With the exception of a cost calculation based on a Markov model, no direct cost-effectiveness studies on the use ofpessary or surgery for POP have been performed [16]. The relevance of this project, with the high prevalence of POP worldwide, associated costs and insufficient evidence, is high. Wehave searched the www.clinicaltrials.gov database (3th March 2014) on similar studies(comparing pessary with surgery) but none were found.

However, if we were to prove that pessary therapy is more cost-effective then surgical treatment, this does not imply that a trial of pessary should always be undertaken. There is also insufficient evidence on which patient characteristics are associated with failure of pessary treatment or surgery (systematic review). The knowledge on how to predict which women will have a very low chance of success with pessary therapy can further improve effective treatment strategy management. This will contribute to treatment efficacy. This is not only very relevant for the hospital specialist care setting, but this knowledge can also be implemented in general practitioner practice units.

There is very limited evidence on the optimal management strategy for pessary cleaning, both in time interval as well as in who should perform the cleaning. Our study is unique and therefore relevant since self-management is advocated in the study setting. This will not only allow it to obtain data in a standardized way, but also involves the woman in her own management. This involvement is strongly advocated by two major gynecologic patient organizations ('Patienten Gynaecologie Nederland' and the 'Stichting Bekkenbodem Patienten'). These two organizations, as well as the Dutch urogynaecological consortium have identified this study to be highly relevant.

For peer review only - http://bmjopen.bmj.com/site/about/guidelines.xhtml

In line with the report "Medisch Specialistische zorg 20/20" we are heading towards integral health care in which the general physician and medical specialist will work more closely together, using the same treatment protocol for various illnesses. The information and conclusions of this trial will add level I scientific evidence to such an integral protocol and guideline for women with symptomatic POP. This will aid in a better patient selection that will need referral to the specialist. The data on patient's self-management of pessary treatment will supply information for patient instructions, which are relevant for information leaflets on the subject.

For peer review only

For peer review only - http://bmjopen.bmj.com/site/about/guidelines.xhtml

2. OBJECTIVES

The aim of this multicenter pragmatic cohort study with embedded randomized controlled non-inferiority trial comparing pessary therapy versus surgery is twofold:

1. To prospectively compare the effectiveness and cost-effectiveness of pessary therapy or surgery as primary treatment of moderate to severe symptomatic cystocele, uterine descent and/or rectocele in women at two year after initiation of treatment, in randomized trial embedded in a preference cohort.
2. To compare the effectiveness between the cohort and randomized trial.
3. To develop a prediction model for failure of pessary use and surgery within the first years.

For peer review only - http://bmjopen.bmj.com/site/about/guidelines.xhtml

3. STUDY DESIGN

Multicenter pragmatic cohort study with an embedded randomized controlled non-inferiority trial comparing pessary [CE 0086] therapy and surgery including an economic evaluation. The follow up will be 24 months.

A short (30 minutes) trial of pessary fitting is part of our protocol. This ensures that only women who fit both treatment options enter the randomization procedure. The trial is short and only aims at fitting, not symptom relief. Women with an unsuccessful pessary fitting will be followed in the cohort fitting failure. In case the woman is willing to participate but actively opts for one of both treatments, she will be followed in a cohort.

See also appendix 1 and 5.

For peer review only

For peer review only - http://bmjopen.bmj.com/site/about/guidelines.xhtml

4. STUDY POPULATION

4.1 Population (base)

All women with a symptomatic POP will be included.

4.2 Inclusion criteria

In order to be eligible to participate in this study, a subject must meet all of the following criteria:

1. Women with a prolapse stage 2 or more.
2. Women with moderate to severe POP symptoms. Moderate to severe POP symptoms is defined as a prolapse domain score > 33 on the validated Dutch version of the Pelvic Floor Distress Inventory (PFDI-20) [8, 23, 24].
3. For the RCT: Women who have had a successful pessary fitting procedure.
4. Written informed consent.

4.3 Exclusion criteria

A potential subject who meets any of the following criteria will be excluded from participation in this study:

1. Prior urogynaecological (prolapse or incontinence) surgery
2. Probability of future childbearing
3. Insufficient knowledge of the Dutch language
4. Co-morbidity causing increased surgical risks at the discretion of the surgeon
5. Major psychiatric illness
6. Prior pessary use

4.4 Sample size calculation

With 198 women per group, we will have 80% power to reject the null hypothesis that pessary therapy is inferior to surgery, with a 1-sided alpha of 0.05, a non-inferiority margin of 10% and the proportion in the standard group is 80% (NQueryAdvisor). Accounting for 10% loss to follow-up we plan to randomize 436 patients.

The sample size calculation for prediction models is based on the number of failures of pessary or surgical therapy. For each potential predictor in the model we need 10-15 failures. Our pessary group sample size is 198 women. An estimated 40% (80 women) will cross over to surgery and can be regarded as failures. Our sample size is therefore sufficient

For peer review only - http://bmjopen.bmj.com/site/about/guidelines.xhtml

to develop the prediction model for failure of pessary therapy for 6 to 8 items. In the surgery group 20% of women will not be satisfied with the result of treatment. With the same sample size of 198 women, the 40 women who are dissatisfied allow us to study up to 4 potential predictive factors.

In the cohort we include all patients who are willing to collaborate on this research but have a preference for one of both therapies. We now assume that 70% of the eligible patients object participation in the RCT, and that 90% of them is nevertheless willing to participate in the cohort.

For peer review only

For peer review only - http://bmjopen.bmj.com/site/about/guidelines.xhtml

5. TREATMENT OF SUBJECTS

5.1 Investigational product/treatment

Pessary [CE 0086] therapy and surgery both are options for the treatment of a symptomatic POP. Ten large urogynecological units (university hospitals or teaching hospitals) that have worked together in previous consortium studies will participate in this multicenter trial. All participating gynaecologists have fitted at least 100 pessaries and performed more than 100 surgical POP procedures prior to the start of this study.

All pessaries are made of modern silicon material. All types of pessaries, both supportive and occlusive/space filling are allowed according to the judgment of the gynaecologist. A recent randomized trial comparing supportive (ring) and occlusive (Gelhorn) showed no differences [17]. After placing the pessary, all women will receive verbal and written instructions on the self-management of pessary therapy.

The first pessary follow up visit will always be performed by the gynaecologist. In case of self-management the frequency of cleaning is left to her personal judgment, but may not exceed 4 months. If self-management is not possible, women will be seen at 4 months intervals for pessary cleaning and vaginal inspection, preferable by their GP. In case of vaginal atrophy topical estrogens will be advised according to pharmaceutical guidelines. The diagnosis of atrophy is left to the judgment of the treating physician, since no clear definition for atrophy is available yet [18].

All surgical procedures will be performed according to our national guidelines. In this pragmatic trial the decision which technique to use is left, to the discretion of the gynaecologist, within the limitations below [19]. Cystocele repair will consist of conventional anterior colporrhaphy [9]. For uterine descent different techniques are allowed [20]. These techniques can either be uterus sparing (sacrospinous hysteropexy [10], modified Manchester-Fothergill procedure [12] or a abdominal sacrocolpopexy [9]) or a vaginal hysterectomy. Recent studies showed similar effectiveness on both anatomical and functional outcomes for these different techniques [10, 12, 21]. A coexistent stage 2 rectocele repair will be a conventional colporrhaphia posterior. All procedures are performed under general or spinal anesthesia and under antibiotics and thrombosis prophylaxis according to local protocols.

For peer review only - http://bmjopen.bmj.com/site/about/guidelines.xhtml

5.2 Use of co-intervention (if applicable)

Not applicable.

5.3 Escape medication (if applicable)

Not applicable.

For peer review only

For peer review only - http://bmjopen.bmj.com/site/about/guidelines.xhtml

6. INVESTIGATIONAL PRODUCT

6.1 Name and description of investigational product(s)

6.2 Summary of findings from non-clinical studies

Not applicable.

6.3 Summary of findings from clinical studies

Three systematic reviews of the literature were performed by four members of our Dutch urogynaecology consortium (details in appendix 2-4) that concluded:

1. Systematic review on the (cost)effectiveness of pessary use compared to surgery

There are a very limited number of comparative studies on the efficacy of surgery or pessary use for POP. The differences in study population, inclusion criteria, follow-up period, large numbers of loss to follow-up, different outcome measures makes interpretation difficult if not impossible. The two studies that presented data on functional outcome in terms of prolapsed symptom reduction were favorable for surgery (appendix 2).

2. Systematic review of factors influencing pessary fitting and continuation

A systematic review was performed to identify the satisfactory pessary fitting rate and the continuation rate of pessary use. The factors influencing these rates as well as the cross over to prolapse surgery were identified from previous studies (appendix 3).

Summarizing the results show that an estimated 75% of women will have a successful fitting and 59% will continue pessary use at variable follow-up between 3 months and 5 years. In these 18 studies, 8 factors have been tested more than 4 times as prognostic factor of successful pessary use: Stress urinary incontinence was found associated with discontinuation of pessary in 5 out of 7 studies. In 7 out of 10 studies previous prolapse surgery or hysterectomy was associated with less continuation of pessary use. Higher age was related to continuation of pessary use in 3 out of 6 studies, whereas no correlation was found in the other studies. In 1 out of 4 studies sexual activity was related to longer pessary use, whereas in 1 out of 4 related to the choice for surgery. In the two other studies no correlation was found. In one study where the prolapse in a specific vaginal compartment was related to outcome, nor cystocele was related to longer pessary use.

Parity en menopausal status and hormonal replacement were mostly not related to continued pessary use.

For peer review only - http://bmjopen.bmj.com/site/about/guidelines.xhtml

3. Review of factors influencing failure of POP surgery.

A systematic review of factors influencing failure of POP surgery was performed concerning recurrence after surgery (surgery failure). There were 1 case control study, 3 prospective studies and 6 retrospective studies. There were 2298 women included in the studies. Forty-four (44) potential risk factors have been studied, of which 12 risk factors have at least once been identified as statistically significant risk factors in a multivariate logistic regression analysis (appendix 4).

6.4 Summary of known and potential risks and benefits

The present study carries low risks for the participant. Pessary [CE 0086] or surgery is standard care for symptomatic pelvic organ prolapse. Known risks for surgery are blood loss, risk of infection, dyspareunia, urine incontinence or a recurrence of a symptomatic pelvic organ prolapse.

The benefit of the study lies in a better understanding of satisfaction and cost effectiveness.

6.5 Description and justification of route of administration and dosage

Not applicable

6.6 Dosages, dosage modifications and method of administration

Not applicable

6.7 Preparation and labelling of Investigational Medicinal Product

Not applicable

6.8 Drug accountability

Not applicable

For peer review only

7. NON-INVESTIGATIONAL PRODUCT

Not applicable.

7.1 Name and description of non-investigational product(s)

Not applicable.

7.2 Summary of findings from non-clinical studies

Not applicable.

7.3 Summary of findings from clinical studies

Not applicable.

7.4 Summary of known and potential risks and benefits

Not applicable.

7.5 Description and justification of route of administration and dosage

Not applicable.

7.6 Dosages, dosage modifications and method of administration

Not applicable.

7.7 Preparation and labelling of Non Investigational Medicinal Product

Not applicable.

7.8 Drug accountability

Not applicable.

For peer review only

For peer review only - http://bmjopen.bmj.com/site/about/guidelines.xhtml

8. METHODS

8.1 Study parameters/endpoints

8.1.1 Main study parameter/endpoint

The primary outcome of this study is the percentage of women with much or very much improvement of POP symptoms at 2 years follow-up, as measured with the Patient Global Impression of Improvement (PGI-I) scale [22].
PGI-I is a 7-point Likert scale, with scores ranging from very much worse to very much improved. Success is defined as 'much or very much' improvement.

8.1.2 Secondary study parameters/endpoints (if applicable)

1. Changes in symptom bother and quality of life at 12 and 24 months follow up.
2. Changes in sexual function at 12 and 24 months follow up.
3. Changes in general quality of life at 3, 6, 12 and 24 months.
4. Adverse events/complications related to both treatment strategies.
5. Development of prediction model to identify fail factors for pessary and surgery
6. Cost-effectiveness

8.1.3 Other study parameters (if applicable)

Baseline characteristics: Age; ethnicity; allergies; smoking; obstetric history including number and mode of deliveries; menopausal status; hormone use; use of medication; height; weight; co-morbidity (diabetes mellitus, COPD); history of gynaecological operations; family history of prolapse; duration of complaints;.
Physical examination: time, POP-Q, atrophy, vulvar deviations, stress test.
Brand pessary, type of surgery.

8.2 Randomisation, blinding and treatment allocation

After written informed consent is obtained, and inclusion and exclusion criteria are assessed, women will be randomly allocated in a 1:1 ratio to either treatment with a pessary or surgical treatment. Randomization will be done web based using ALEA, the software for randomization in clinical trials currently used by most studies in the Dutch consortium for studies on women's health and reproduction studies. The randomization sequence will be computer generated using variable blocks of two and four, stratified for centre.
After entering the woman's initials and confirming inclusion criteria on the website, a

For peer review only - http://bmjopen.bmj.com/site/about/guidelines.xhtml

unique number for randomization will be generated and the allocation code will be disclosed. This unique number cannot be deleted afterwards. This study will be open label because the nature of the intervention meant that masking to the intervention was not possible. Women who attend the cohort fitting failure will also be registered in ALEA.

In case the woman is willing to participate but actively opts for one of both treatments, she will also be registered in ALEA.

All groups will have the same data collection and follow up as displayed in appendix 5. We expect differences in the study parameters between RCT and cohort, in effectivity, satisfaction and cost effectivity.

8.3 Study procedures (see also appendix 5)

This study will be performed within the Dutch Urogynaecology Research Consortium, a subdivision of the Dutch Consortium for studies on women's health. Infrastructure (research nurses for counseling and data-monitoring, the use of web-based data entry), expertise on methodology and cost-effectiveness is shared.

1. Symptom bother and disease-specific quality of life are measured with the Pelvic Floor Distress Inventory (PFDI-20) and Pelvic Floor Impact Questionnaire (PFIQ) [23,24]. The validated version of the Dutch PFDI consists of five domains: pelvic organ prolapse, urinary incontinence, overactive bladder, pain, and obstructive micturition. The PFIQ measures the impact of urogenital symptoms on quality of life and consist of five domains: physical functioning, mobility, emotional health, embarrassment and social functioning.

2. Sexual function is measured with the PISQ-R. It is an international disease-specific questionnaire that measures sexual functioning in sexually active and inactive participants [26].

3. Generic quality of life is measured with the EQ-5D and a questionnaire "doktersbezoek".

4. The adverse events of surgery recorded will consist of; direct peri-and postoperative complications (bleeding, pain and infection); interventions for complications; recurrent prolapse; de novo stress urinary incontinence. The adverse events of pessary recorded will consist of; discharge; pain; discomfort; bleeding; involuntary loss of pessary; de novo stress urinary incontinence.

5. The development of a prediction model is separately described in paragraph "data analyses".

6. The economic evaluation is described below.

For peer review only - http://bmjopen.bmj.com/site/about/guidelines.xhtml

ANTICIPATED COST-EFFECTIVENESS/BUDGET IMPACT

Cost differences between the two strategies are mainly the result from differences in costs associated with the initial intervention. Cost of a POP procedure is estimated at 4000 euros direct medical costs, and 4000 euros associated with lost productivity (indirect costs) if a societal perspective is used. The direct cost of pessary use is estimated at 200 euros, including costs for the pessary itself (50 euros) and consultations in the first year (150 euros). The estimated cost differences between the two strategies depend on the extent that women are (and remain) satisfied with the initial procedure (surgical or pessary): in case of dissatisfaction with the procedure, additional costs are generated by a subsequent intervention ((re-)operation, pessary, or pelvic physiotherapy). The flowchart (see appendix 1) illustrates the expected outcomes for each strategy. Based on the assumptions reflected in this flowchart, combined with approximate estimates for unit costs for POP surgery, pessary, GP and specialist visits, the anticipated impact on the annualhealth care budget as well as societal costs were estimated.

At present, the primary therapy for women presenting with moderate to severe POP is either surgery or pessary. The exact ratio is unknown, but is probably 50/50. If 50% women would receive primary surgery the current medical costs amount to 34 million Euros. If all women would start with pessary therapy, these costs would be 20 million euros, and the potential budget impact would be 14 million Euros/year. Asit is not realistic that all women will start with pessary if this strategy proves to be successful, at 85% implementation of the pessary strategy, the annual budgetimpact will be around 10 million euros. The economic impact to society (including indirect (productivity) costs) will be 28 million euros and 20 million euros, at 100% and 85% implementation, respectively.

Sensitivity analyses showed, that these "base case" results are affected by estimated unit costs for POP surgery (direct and indirect costs) and the satisfaction rate for pessary, relative to surgery, but even the most conservative assumptions would lead to major cost savings for the health care budget (5 million euros) and society (15 million euros).

ECONOMIC EVALUATION

The economic evaluation will be based on the randomized trial. Considering the non-inferiority design of the study, we will not be able to rule out a small but acceptable

For peer review only - http://bmjopen.bmj.com/site/about/guidelines.xhtml

difference in favor of POP surgery. Consequently, the economic evaluation will be setup as a cost-effectiveness analysis, where cost-effectiveness will be expressed as costs per improvement outcome (much or very much improvement on the Patient Global Impression of Improvement (PGI-I)), and the incremental cost-effectiveness ratio as costs saved per additional case of unsatisfactory outcome. We will also perform a cost-effectiveness analysis using QALYs as health outcome, to express the difference between the two strategies in terms of costs (saved) per QALY (lost).

The economic evaluation will therefore encompass a cost-effectiveness analysis (CEA), a cost-utility analysis (CUA) as well as a budget impact analysis (BIA) from a health care budget and a societal perspective, with a time horizon between randomization and 2 years follow up. The primary outcome in the cost-effectiveness analysis will be costs per satisfactory outcome (primary clinical outcome), and the incremental cost-effectiveness ratio will reflect the costs saved per additional case of unsatisfactory outcome. As we hypothesize that pessary as a primary strategy in these patients does not result in more unsatisfactory outcomes, increased use of pessary will result in a decrease in the number of POP surgeries, and associated costs of hospital stay, recovery and (from a societal perspective) productivity loss (non-inferior strategy at lower costs).

Based on data actually observed in the trial, total costs associated with both surgery and pessary as a primary strategy will be estimated. Total costs can be divided into direct medical costs, non-medical costs and indirect costs. Direct medical costs are generated by utilization of primary or secondary health care services (including POP surgery, hospital stay, diagnostic procedures, medication). Non-medical costs are generated by travel expenses, and informal care; and indirect costs result from lost productivity due to absence from work or lost opportunity for non-paid activities. Non-medical and indirect costs are only included in the analysis from a societal perspective.

Resource utilization will be documented in the clinical report form (CRF) and complementary patient questionnaires, based on the Medical Consumption Questionnaire (MCQ) and Productivity Costs Questionnaire (PCQ) [29,30]. In patients for whom complete follow-up is not available, cost and quality-of-life datawill be extrapolated using multiple imputations. Unit costs will be based on Dutch guideline prices (for primary and secondary health services, informal care and lost productivity),

For peer review only - http://bmjopen.bmj.com/site/about/guidelines.xhtml

and market prices (for medication)[31,32]

Similarly, the incremental costs per QALY gained will be estimated over a period of two years. Health state utilities to estimate QALYs will be derived from an EQ-5D measurement at discharge, as well as at follow-up assessments. Utility values for EQ-5D scores will be based on UK-estimates (Dolan, 1997). Utility scores will be linearly interpolated, assuming constant increase/decrease between subsequent assessments.

Robustness for sampling uncertainty as well as uncertainty associated with cost estimates and assumptions will be assessed in sensitivity analyses, including: Dutch health states (Lamers, 2005) instead of the UK based model in the main analyses; and varying unit costs for pertinent volumes of health care utilization (e.g. costs of POP surgery, pessary use, productivity costs).
The incremental costs and effects will be depicted in a cost effectiveness plane and cost-effectiveness acceptability curves providing information directly interpretable as the probability of one intervention being cost-effective compared to the alternative given a ceiling ratio that policy makers are willing to invest.

BIA
In a budget impact analysis, study results will be extrapolated to the national level to estimate the total impact on the health care budget per annum for the Netherlands in terms of cost reduction and health outcomes (satisfactory outcomes as well as QALYs). As economic consequences of the intervention are expected to spanmultiple years, this accumulation of cost (savings) will be reflected in the budget impact analyses.

The Budget Impact Analysis will be executed according to the international ISPOR guidelines [33]. This framework for creating a budget impact model includes formalized guidance about the acquisition and use of data in order to make budget projections. In addition to the societal perspective, the BIA will therefore be also report economic consequences from the perspective of the Dutch budgetary health care framework (BKZ). If the probability of an unsatisfactory outcome exceeds the non-inferiority limit, recommending pessary as primary treatment for all women isnot feasible, and an economic evaluation/budget impact analysis is not sensible. To estimate costs, we will follow the Handleiding Kosten onderzoek CVZ 2010.

For peer review only - http://bmjopen.bmj.com/site/about/guidelines.xhtml

8.4 Withdrawal of individual subjects

Subjects can leave the study at any time for any reason if they wish to do so without any consequences. The investigator can decide to withdraw a subject from the study for urgent medical reasons.

8.4.1 Specific criteria for withdrawal (if applicable)

Not applicable.

8.5 Replacement of individual subjects after withdrawal

We will not replace patients who withdrew informed consent. We will replace patients that are randomized by mistake, for example because of technical errors with online randomization.

8.6 Follow-up of subjects withdrawn from treatment

Patients withdrawn from the intervention but not from informed consent will be followed up.

8.7 Premature termination of the study

This study includes standard care, therefore it is very unlikely that unexpected complications will occur. Therefore premature termination is not applicable.

For peer review only

9. SAFETY REPORTING

9.1

Temporary halt for reasons of subject safety (section 9.1, CCMO Template Research Protocol)

In accordance to section 10, subsection 4, of the WMO, the sponsor will suspend the study if there is sufficient ground that continuation of the study will jeopardise subject health or safety. The sponsor will notify the accredited METC with undue delay of a temporary halt including the reason for such an action. The study will be suspended pending further review by the accredited METC. The investigator will take care that all subjects are kept informed.

Temporary halt and (prematurely) end of study report (section 12.5, CCMO Template Research Protocol)

The sponsor will notify the METC immediately of a temporary halt of the study, including the reason of such an action.

9.2 AEs, SAEs and SUSARs

9.2.1 Adverse events (AEs)

Adverse events are defined as any undesirable experience occurring to a subject during the study, whether or not considered related to the study. All adverse events reported spontaneously by the subject or observed by the investigator or his staff will be recorded. During visits complaints will be questioned systematically.

9.2.2 Serious adverse events (SAEs)

A serious adverse event is any untoward medical occurrence or effect that at any dose:

- results in death;
- is life threatening (at the time of the event);
- requires hospitalisation or prolongation of existing inpatients' hospitalisation (>4 days);
- results in persistent or significant disability or incapacity;
- is a congenital anomaly or birth defect;
- Any other important medical event that may not result in death, be life threatening, or require hospitalization, may be considered a serious adverse experience when,

For peer review only - http://bmjopen.bmj.com/site/about/guidelines.xhtml

based upon appropriate medical judgement, the event may jeopardize the subject or may require an intervention to prevent one of the outcomes listed above.

SAEs have to be reported when its occurrence appears in two days after the study operations. The investigators in participating centres should inform the coordinating investigator as soon as possible but at least the next working day.

The sponsor will report the SAEs through the web portal *ToetsingOnline* to the accredited METC that approved the protocol, within 15 days after the sponsor has first knowledge of the serious adverse events.

SAEs that result in death or are life threatening should be reported expedited. The expedited reporting will occur not later than 7 days after the responsible investigator has first knowledge of the adverse event. This is for a preliminary report with another 8 days for completion of the report.

9.2.3 Suspected unexpected serious adverse reactions (SUSARs)

Not applicable.

9.3 Annual safety report

The annual safety report will be combined with the annual progress report (see chapter 12.4).

9.4 Follow-up of adverse events

All AEs will be followed until they have abated, or until a stable situation has been reached. Depending on the event, follow up may require additional tests or medical procedures as indicated, and/or referral to the general physician or a medical specialist.

SAEs need to be reported till end of study within the Netherlands, as defined in the protocol

9.5 Data Safety Monitoring Board (DSMB)

Since both techniques are standard practice, and no major unexpected complications are foreseen, no interim analysis is planned. A Data Safety and Monitoring Board will not be installed, as both procedures are regularly used and acceptable options in current clinical practice.

For peer review only - http://bmjopen.bmj.com/site/about/guidelines.xhtml

10. STATISTICAL ANALYSIS

The results of the study will be reported according to the CONSORT statement.

The main outcomes will be analysed and presented according the intention-to-treat principle. Since in this pragmatic trial we expect that in the pessary strategy group 40% of women will cross over to surgery an additional per protocol analyses is foreseen. This will provide more insight in the effect of surgery after pessary therapy as compared to surgery or pessary therapy alone.

We plan a subgroup analysis for the location of the prolapse: anterior prolapse versus posterior prolapse.

The cohort with patients treated according their preference will be analysed separately from the randomized trial, and presented in the same manuscript, which will provide insight into the generalizability of the results.

10.1 Primary study parameter(s)

The primary outcome, success (much or very much improvement) or no success (a little better, no change, a little worse, much worse or very much worse) on the PGI-I will be expressed in percentage point differences. Differences between the percentages will be tested using a chi-square test. A p-value <0.05 will be considered statistically significant.

For the cohort study results will be presented separately, and the same analyses will be done. Differences between the trial arm and the cohort arm will be tested using the chi-square test, to determine the generalizability of the results.

10.2 Secondary study parameter(s)

The PFDI, PFIQ and PISQ-r are all interval scales. Differences between baseline and 12 and 24 months follow up will be assessed using an independent t-test when normality can be assumed, or by non-parametric tests when the data are not normally distributed. Effect sizes will be calculated to estimate the magnitude of changes.

Differences in EQ5-D scores and "ziekteverzuim" between baseline and at 3, 6, 12 and 24 months will be assessed using t-test and further incorporated in the cost-effectiveness analyses.

Imputation statistics will be used or missing data.

(Serious) adverse events will be categorized and chi-square statistics, with calculation of relative risks when appropriate, will be applied in analyses.

For peer review only - http://bmjopen.bmj.com/site/about/guidelines.xhtml

Prediction model

A prediction model that uses predefined variables, as potential predictors of failure of pessary therapy, will be developed using multivariable regression analysis.Missing data will be imputed.

Predictors for failure derived from literature are a large genital hiatus (gh > 4 cm), women being sexually active, age > 65 years, prolapse POP-Q stage 3, previous hysterectomy. If applicable, a prediction rule for the chance of failure of pessary therapy will be constructed, which could be presented as a normogram which could be used to determine the chance of failure on pessary therapy.

Internal validity will be assessed using bootstrapping techniques; shrinkage will be applied to the parameter estimates. Model performance will be assessed with discriminative capacity and calibration. Calibration will be assessed by comparing the mean predicted probability that patients failed on pessary therapy with the mean observed probability that patients failed on pessary therapy. To do so, the total cohort will be split into ten groups based on the deciles of the predicted probability. Per group the mean predicted probability will be calculated as well as the mean observed predicted probability. Discriminative capacity of the model will be assessed with receiver operation characteristics (ROC) analysis and the area under the ROC curve (AUC).

We will also look at factors that could explain failure of surgery. Our systematic review on POP and recurrent POP after surgery showed that 6 preoperative items, eg. POP stage, age, family history, preoperative incontinence, previous POP or incontinence surgery, previous hysterectomy seems to be predictive for recurrence.

Women with previous POP or incontinence surgery are excluded from our study, leaving 4 predefined potential predictive factors. After the 2 year follow-up has been performed, we will reconsider which factors to include in a prognostic model, based on the current literature. We will select predictors from literature with the highest predictive value, where about 1 predictor could be selected for each 10 surgery failures.

Using interaction terms the effect of a differential effect in women with a higher age (>median) or a lower age (<=median), a higher (>25) or lower BMI(<=25) will be assessed for both pessary as well as surgery failures prediction.

For peer review only - http://bmjopen.bmj.com/site/about/guidelines.xhtml

10.3 Other study parameters

Not applicable.

10.4 Interim analysis (if applicable)

Not applicable, because of the non-inferiority design with low risk and the possibility of cross over.

For peer review only

11. ETHICAL CONSIDERATIONS

11.1 Regulation statement

This study will be conducted according to the principles of the Declaration of Helsinki (version 10, October 2013) and in accordance with the Medical Research InvolvingHuman Subjects Act (WMO) and other guidelines, regulations and Acts.

11.2 Recruitment and consent

Women with symptomatic pelvic organ prolapse who attend the outpatient clinic will be informed about the study by the gynaecologist or nurse. After checking the in- and exclusion criteria the women will receive verbal and written information about the study. If the woman is willing to participate she is asked to sign the informed consent. All women will undergo the pessary fitting test which is part of the standard evaluation. All women will be contacted at a minimum interval of 1 week. Those women who failed the initial fitting will be offered surgery and attend the cohort fitting failure, the women with a succesfull initial fitting will be asked to enroll in the RCT. In case the woman is willing to participate but actively opts for one of both treatments, she can attend the cohort. Her motivation is requested. In case the women is not willing to participate, she will be registred as "refuser".

11.3 Objection by minors or incapacitated subjects (if applicable)

Not applicable.

11.4 Benefits and risks assessment, group relatedness

The present study carries no risks for the participant. Pessary or surgery are standard care for symptomatic pelvic organ prolapse. The benefit of the study lies in a better understanding of satisfaction and cost effectiveness.

11.5 Compensation for injury

The sponsor/investigator has a liability insurance which is in accordance with article 7, subsection 9 of the WMO.

The sponsor (also) has an insurance which is in accordance with the legal requirements in the Netherlands (Article 7 WMO and the Measure regarding Compulsory Insurance for Clinical Research in Humans of 23th June 2003). This insurance provides cover for

For peer review only - http://bmjopen.bmj.com/site/about/guidelines.xhtml

damage to research subjects through injury or death caused by the study.

1. € 450.000,-- (i.e. four hundred and fifty thousand Euro) for death or injury for each subject who participates in the Research;
2. € 3.500.000,-- (i.e. three million five hundred thousand Euro) for death or injury for all subjects who participate in the Research;
3. € 5.000.000,-- (i.e. five million Euro) for the total damage incurred by the organisation for all damage disclosed by scientific research for the Sponsor as 'verrichter' in the meaning of said Act in each year of insurance coverage.

The insurance applies to the damage that becomes apparent during the study or within 4 years after the end of the study.

11.6 Incentives (if applicable)

Not applicable.

For peer review only

12. ADMINISTRATIVE ASPECTS, MONITORING AND PUBLICATION

12.1 Handling and storage of data and documents

All data will be stored and will be coded. Only the researchers will be able to link patient ID and research code. The handling of personal data complies with the Dutch Personal Data Protection Act. After cessation of the study, patient material will be stored for a maximum of 15 years.

The case report forms and questionnaires will be filled in online. The head investigator will be able to check all the completed forms and questionnaires.

12.2 Monitoring and Quality Assurance

The monitoring will be coordinated by the Dutch Consortium and will be executed by a qualified intern monitor. This person is not involved in design and output of this research. The frequency of checking will be every year. The monitoring plan is discussed in section K of the METC dossier.

12.3 Amendments

All substantial amendments will be notified to the METC.

Non-substantial amendments will not be notified to the accredited METC, but will be recorded and filed by the sponsor.

12.4 Annual progress report

The sponsor/investigator will submit a summary of the progress of the trial to the accredited METC once a year. Information will be provided on the date of inclusion of the first subject, numbers of subjects included and numbers of subjects that have completed the trial, serious adverse events/ serious adverse reactions, other problems, and amendments.

12.5 End of study report

The investigator will notify the accredited METC of the end of the study within a period of 8 weeks. The end of the study is defined as the last patient’s last visit.

In case the study is ended prematurely, the investigator will notify the accredited METC within 15 days, including the reasons for the premature termination.

For peer review only - http://bmjopen.bmj.com/site/about/guidelines.xhtml

Within one year after the end of the study, the investigator/sponsor will submit a final study report with the results of the study, including any publications/abstracts of the study,to the accredited METC.

12.6 Public disclosure and publication policy

The research findings will be published in peer reviewed journals.

For peer review only

For peer review only - http://bmjopen.bmj.com/site/about/guidelines.xhtml

13. STRUCTURED RISK ANALYSIS

Not applicable because this study is a low risk study concerning standard care.

13.1 Potential issues of concern

Not applicable.

13.2 Synthesis

Not applicable.

For peer review only

For peer review only - http://bmjopen.bmj.com/site/about/guidelines.xhtml

14. REFERENCES

1: Slieker-ten Hove MC et al. The prevalence of pelvic organ prolapse symptoms and signs and their relation with bladder and bowel disorders in a general female population. Int Urogynecol J. 2009;20(9):1037-45
2. Tegerstedt G, Maehle-Schmidt M, Nyrén O, Hammarström M. Prevalence of symptomatic pelvic organ prolapse in a Swedish population. Int Urogynecol J Pelvic Floor Dysfunct. 2005 Nov-Dec;16(6):497-503
3: Lamers BH et al. Pessary treatment for pelvic organ prolapse and health-related quality of life: a review. Int Urogynecol J. 2011;22(6):637-44
4: Broekman BM et al. Prospective pilot study in 65 women on pessary treatment as primary treatment, unpublished data
5:Broens-Oostveen WC, Mom RM, Lagro-Janssen ALM. De genitale prolaps: behnadleingen beloop in vier huisartspraktijken. Ned Tijdschr Geneeskd 2004, 148 (29); 1444-1448
6: Withagen M et al. Sacrospinous hysteropexy or modified manchester for the treatment of pelvic organ prolapse? Int J Gynaecol Obstet. 2013; Abstract Supplement
7: Vollebregt A et al. Cost-utility analysis of trocar-guided transobturator mesh versus anterior colporrhaphy for primary cystocele repair: a randomised trial. [in preparation]
8: Barber MD[1], Walters MD, Bump RC. Short forms of two condition-specific quality-of-life questionnaires for women with pelvic floor disorders (PFDI-20 and PFIQ-7). Am J Obstet Gynecol. 2005 Jul;193(1):103-13.
9: Pollard ME, Eilber KS, Anger JT. Abdominal approaches to pelvic prolapse repairs. Curr Opin Urol. 2013;23(4):306-11.
10: Dietz V et al. Vaginal surgery for uterine descent; which options do we have? A review of the literature. Int J Gynaecol Obstet. 2009;20(3):349-56
11: C. Maher, Feiner B, Baessler K, Schmid C. Surgical management of pelvic organprolapse in women. Cochrane 2013
12: de Boer TA, Milani AL, Kluivers KB, Withagen MI, Vierhout ME. The effectiveness of surgical correction of uterine prolapse: cervical amputation with uterosacral ligament plication (modified Manchester) versus vaginal hysterectomy with high uterosacral ligament plication. Int Urogynecol J Pelvic Floor Dysfunct. 2009 Nov;20(11):1313-9
13: Bugge C et al. Pessaries (mechanical devices) for pelvic organ prolapse in women. Cochrane database syst rev, 2013;28;2:CD004010
14: van Geelen JM, Dwyer PL. Where to for pelvic organ prolapse treatment after the FDA pronouncements? A systematic review of the recent literature. Int Urogynecol J. 2013 May;24(5):707-18
15: Abdool Z et al. Prospective evaluation of outcome of vaginal pessaries versus surgery in women with symptomatic pelvic organ prolapse. Int Urogynecol J. 2011;22(3):273-8
16: Hullfish KL, Trowbridge ER, Stukenborg GJ. Treatment strategies for pelvic organ prolapse: a cost-effectiveness analysis. Int Urogynecol J. 2011 May;22(5):507-15
17: Cundiff GW, Amundsen CL, Bent AE, Coates KW, Schaffer JI, Strohbehn K, Handa VL. The PESSRI study: symptom relief outcomes of a randomized crossover trial of the ring and Gellhorn pessaries. Am J Obstet Gynecol. 2007 Apr;196(4):405.e1-8
18: Weber MA, Limpens J, Roovers JPWR. Assessment of vaginal atrophy: a review [submitted BJOG]
19: Cobra Ok-Klapper 2012. DCHG 2012
20: Detollenaere RJ, den Boon J, Kluivers KB, Vierhout ME, van Eijndhoven HW.Surgical management of pelvic organ prolapse and uterine descent in the Netherlands. Int Urogynecol J. 2013 May;24(5):781-8
21.Detollenaere RJ, den Boon J, Vierhout ME, van Eijndhoven HW. Uterus preservingsurgery versus vaginal hysterectomy in treatment of uterine descent: a systematic review]. Ned

For peer review only - http://bmjopen.bmj.com/site/about/guidelines.xhtml

Tijdschr Geneeskd. 2011;155(42):A3623
22: Yalcin I, Bump RC. Validation of two global impression questionnaires for incontinence. Am J Obstet Gynecol. 2003 Jul;189(1):98-101
23: Barber MD, Kuchibhatla MN, Pieper CF, Bump RC. Psychometric evaluation of 2 comprehensive condition-specific quality of life instruments for women with pelvic floor disorders. Am J Obstet Gynecol 2001;185(6):1388–1395.
24: Utomo E, Blok BF, Steensma AB, Korfage IJ. Validation of the Pelvic Floor Distress Inventory (PFDI-20) and Pelvic Floor Impact Questionnaire (PFIQ-7) in a Dutch population. Int Urogynecol J. 2014;25(4):531-44.
25: Euroquol—a new facility for the measurement of health-related quality of life. The EuroQol Group. Health Policy. 1990;16:199-208
26: Rogers RG, Rockwood TH, Constantine ML, Thakar R, Kammerer-Doak DN, Pauls RN, Parekh M, Ridgeway B, Jha S, Pitkin J, Reid F, Sutherland SE, Lukacz ES, Domoney C, Sand P, Davila GW, Espuna Pons ME. A new measure of sexual function in women with pelvic floor disorders (PFD): the Pelvic Organ Prolapse/Incontinence Sexual Questionnaire, IUGA-Revised (PISQ-IR). Int Urogynecol J. 2013 Jul;24(7):1091-103
27: Cabana MD, Rand CS, Powe NR, Wu AW, Wilson MH, Abboud PA, Rubin HR. Why don't physicians follow clinical practice guidelines? A framework for improvement. JAMA. 1999 Oct 20;282(15):1458-65
28: Thys SD, Roovers JP, Geomini PM, Bongers MY. Do patients prefer a pessary or surgery as primary treatment for pelvic organ prolapse. Gynecol Obstet Invest. 2012;74(1):6-12
29: www.imta.nl (C. Bouwmans et al. Handleiding iMTA Medical Cost Questionnaire (iMCQ). Rotterdam: iMTA, Erasmus Universiteit Rotterdam, 2013)
30: www.imta.nl (C. Bouwmans et al. Handleiding iMTA Productivity Cost Questionnaire (iPCQ). Rotterdam: iMTA, Erasmus Universiteit, 2013)
31:Hakkaart- van Roijen, S.S. Tan, C.A.M. Bouwmans: Handleiding voor Kostenonderzoek. Methoden en standaard kostprijzen voor economische evaluaties in de gezondheidszorg. CVZ 2010
32: Oostenbrink JB, Koopmanschap MA, Rutten FF. Standardisation of costs: the Dutch Manual for Costing in economic evaluations. Pharmacoeconomics 2002;20(7):443-454
33: Mauskopf JA, Sullivan SD, Annemans L et al. Principles of good practice for budget impact analysis: report of the ISPOR Task Force on good research practices--budget impact analysis. Value Health 2007;10(5):336-347 -> In BIA, klopt niet, nog vervangen door:

References (Cost) effectiveness of pessary use as compared to surgery systematic review

1: Mamik MM, Rogers RG, Qualls CR, Komesu YM. Goal attainment after treatment in patients with symptomatic pelvic organ prolapse. Am J Obstet Gynecol. 2013 Nov;209(5):488.e1-5.
2: Abdool Z, Thakar R, Sultan AH, Oliver RS. Prospective evaluation of outcome of vaginal pessaries versus surgery in women with symptomatic pelvic organ prolapse. Int Urogynecol J. 2011 Mar;22(3):273-8.
3: Lowenstein L, Gamble T, Sanses TV, van Raalte H, Carberry C, Jakus S, Pham T, Nguyen A, Hoskey K, Kenton K; Fellow's Pelvic Research Network. Changes in sexual function after treatment for prolapse are related to the improvement in body image perception. J Sex Med. 2010 Feb;7(2 Pt 2):1023-8.
4: Barber MD, Walters MD, Cundiff GW; PESSRI Trial Group. Responsiveness of the Pelvic Floor Distress Inventory (PFDI) and Pelvic Floor Impact Questionnaire (PFIQ) in women undergoing vaginal surgery and pessary treatment for pelvic organ prolapse. Am J Obstet Gynecol. 2006 May;194(5):1492-8.

References Systematic review of factors influencing pessary fitting and continuation

1.Abdool Z, Thakar R, Sultan AH, Oliver RS (2010) Prospective evaluation of outcome of

For peer review only - http://bmjopen.bmj.com/site/about/guidelines.xhtml

vaginal pessaries versus surgery in women with symptomatic pelvic organ prolapse. Int Urogynecol J Pelvic Floor Dysfunct 16

2. Alperin M, Khan A, Dubina E, Tarnay C, Wu N, Pashos CL, Anger JT.Female Pelvic Med Reconstr Surg. 2013 May-Jun;19(3):142-7.Patterns of pessary care and outcomes for medicare beneficiaries with pelvic organ prolapse.
3. Bai SW, Yoon BS, Kwon JY, Shin JS, Kim SK, Park KH (2005) Survey of the characteristics and satisfaction degree of the patients using a pessary. Int Urogynecol J Pelvic Floor Dysfunct 16 (3):182–186
4. Barber MD, Walters MD, Cundiff GW (2006) Responsiveness of the Pelvic Floor Distress Inventory (PFDI) and Pelvic Floor Impact Questionnaire (PFIQ) in women undergoing vaginal surgery and pessary treatment for pelvic organ prolapse. Am JObstet Gynecol 194(5):1492–149
5. Brincat C, Kenton K, Pat FM, Brubaker L (2004) Sexual activity predicts continued pessary use. Am J Obstet Gynecol 191(1):198–200
6. Broens-Oostveen MC, Mom RM, Lagro-Janssen AL (2004)Genital prolapse; treatment and course in four general practices.Ned Tijdschr Geneeskd 148(29):1444–1448, 17
7. Chan SS, Cheung RY, Yiu KW, Lee LL, Pang AW, Chung TK. Int Urogynecol J. 2012 Aug;23(8):1027-33. Symptoms, quality of life, and factors affecting women's treatment decisions regarding pelvic organ prolapse.
8. Clemons JL, Aguilar VC, Sokol ER, Jackson ND, Myers DL (2004) Patient characteristics that are associated with continued pessary use versus surgery after 1 year. Am J Obstet Gynecol 191(1):159–164
9. Cundiff GW, Amundsen CL, Bent AE, Coates KW, Schaffer JI, Strohbehn K et al (2007) The PESSRI study: symptom relief outcomes of a randomized crossover trial of the ring and Gellhorn pessaries. Am J Obstet Gynecol 196(4):405–408
10. Fernando RJ, Thakar R, Sultan AH, Shah SM, Jones PW (2006) Effect of vaginal pessaries on symptoms associated with pelvic organ prolapse. Obstet Gynecol 108(1):93–99
11. Friedman S, Sandhu KS, Wang C, Mikhail MS, Banks E (2010) Factors influencing long-term pessary use. Int Urogynecol J Pelvic Floor Dysfunct 21(6):673–678
12. Hanson LA, Schulz JA, Flood CG, Cooley B, Tam F (2006) Vaginal pessaries in managing women with pelvic organ prolapse and urinary incontinence: patient characteristics and factors contributing to success. Int Urogynecol J Pelvic Floor Dysfunct 17(2):155–159
13. Jones K,Yang L, Lowder JL, Meyn L, Ellison R, ZyczynskiHMet al (2008) Effect of pessary use on genital hiatus measurements in women with pelvic organ prolapse. Obstet Gynecol 112(3):630–636
14. Geoffrion R, Zhang T, Lee T, Cundiff GW.Female Pelvic Med Reconstr Surg. 2013 Nov-Dec;19(6):339-45. Clinical characteristics associated with unsuccessful pessary fitting outcomes.
15. Komesu YM, Rogers RG, Rode MA, Craig EC, Gallegos KA, Montoya AR et al (2007) Pelvic floor symptom changes incpessary users. Am J Obstet Gynecol 197(6):620–626
16. Kuhn A, Bapst D, Stadlmayr W, Vits K, Mueller MD (2009) Sexual and organ function in patients with symptomatic prolapse: are pessaries helpful? Fertil Steril 91(5):1914–1918
17. Lone F1, Thakar R, Sultan AH, Karamalis G. A 5-year prospective study of vaginal pessary use for pelvic organ prolapse. Int J Gynaecol Obstet. 2011 Jul;114(1):56-9.
18. Maito JM, Quam ZA, Craig E, Danner KA, Rogers RG (2006) Predictors of successful pessary fitting and continued use in a nurse-midwifery pessary clinic. J Midwifery Womens Health 51 (2):78–84
19. Mamik MM, Rogers RG, Qualls CR, Komesu YM. Goal attainment after treatment in patients with symptomatic pelvic organ prolapse. Am J Obstet Gynecol. 2013 Nov;209(5):488.e1-5.
20. Manchana T, Bunyavejchevin S. Impact on quality of life after ring pessary use for pelvic organ prolapse. Int Urogynecol J. 2012 Jul;23(7):873-7.
21. Markle D, Skoczylas L, Goldsmith C, Noblett K. Patient characteristics associated with a

For peer review only - http://bmjopen.bmj.com/site/about/guidelines.xhtml

successful pessary fitting. Female Pelvic Med Reconstr Surg. 2011 Sep;17(5):249-52.
22. Mokrzycki ML, Hatangadi SB, Zaccardi JE, Cox S. Preexisting stress urinary incontinence: a predictor of discontinuation with pessary management. J Low Genit TractDis. 2001 Oct;5(4):204-7.
23. Mutone MF, Terry C, Hale DS, Benson JT (2005) Factors which influence the short-term success of pessary management of pelvic organ prolapse. Am J Obstet Gynecol 193(1):89–94
24. Nguyen JN, Jones CR. Pessary treatment of pelvic relaxation: factors affecting successful fitting and continued use. J Wound Ostomy Continence Nurs. 2005;32(4):255-61; quiz 262-3.
25. Patel M, Mellen C, O'Sullivan DM, Lasala CA. (2010) Impact ofpessary use on prolapse symptoms, quality of life, and bodyimage. Am J Obstet Gynecol 18
26. Powers K, Lazarou G, Wang A, LaCombe J, Bensinger G.Greston WM et al (2006) Pessary use in advanced pelvic organprolapse. Int Urogynecol J Pelvic Floor Dysfunct17(2):160–164.

Reference list Systematic review of factors influencing failure of POP surgery

1. Whiteside JL, Weber AM, Meyn LA, Walters MD. Am J Obstet Gynecol. 2004 Nov;191(5):1533-8.factors for prolapse recurrence after vaginal repair.
2. Diez-Itza I, Aizpitarte I, Becerro A. Int Urogynecol J Pelvic Floor Dysfunct. 2007 Nov;18(11):1317-24. Risk factors for the recurrence of pelvic organ prolapse after vaginal surgery: a review at 5 years after surgery.
3. Fialkow MF, Newton KM, Weiss NS. Int Urogynecol J Pelvic Floor Dysfunct. 2008 Nov;19(11):1483-7.Incidence of recurrent pelvic organ prolapse 10 years following primary surgical management: a retrospective cohort study.
4. Jeon MJ, Chung SM, Jung HJ, Kim SK, Bai SW. Gynecol Obstet Invest. 2008;66(4):268- 73 Risk factors for the recurrence of pelvic organ prolapse.
5. Dällenbach P, Jungo Nancoz C, Eperon I, Dubuisson JB, Boulvain M. Int Urogynecol J. 2012 Jan;23(1):35-41. Incidence and risk factors for reoperation of surgically treated pelvic organ prolapse.
6. Weemhoff M, Vergeldt TF, Notten K, Serroyen J, Kampschoer PH, Roumen FJ. Int Urogynecol J. 2012 Jan;23(1):65-71. Avulsion of puborectalis muscle and other risk factors for cystocele recurrence: a 2-year follow-up study.
7. Tegerstedt G1, Hammarström M. Acta Obstet Gynecol Scand. 2004 Aug;83(8):758-63. Operation for pelvic organ prolapse: a follow-up study.
8. Medina CA, Candiotti K, Takacs P. Int J Gynaecol Obstet. 2008 May;101(2):184-7. Wide genital hiatus is a risk factor for recurrence following anterior vaginal repair.
9. Salvatore S1, Athanasiou S, Digesu GA, Soligo M, Sotiropoulou M, Serati M, Antsaklis A, Milani R. Neurourol Urodyn. 2009;28(4):301-4. doi: 10.1002/nau.20639. Identification of risk factors for genital prolapse recurrence.
10. Kawasaki A, Corey EG, Laskey RA, Weidner AC, Siddiqui NY, Wu JM. J Reprod Med. 2013 May-Jun;58(5-6):195-9. Obesity as a risk for the recurrence of anterior vaginal wall prolaps after anterior colporrhaphy.

For peer review only - http://bmjopen.bmj.com/site/about/guidelines.xhtml

Appendix 1:

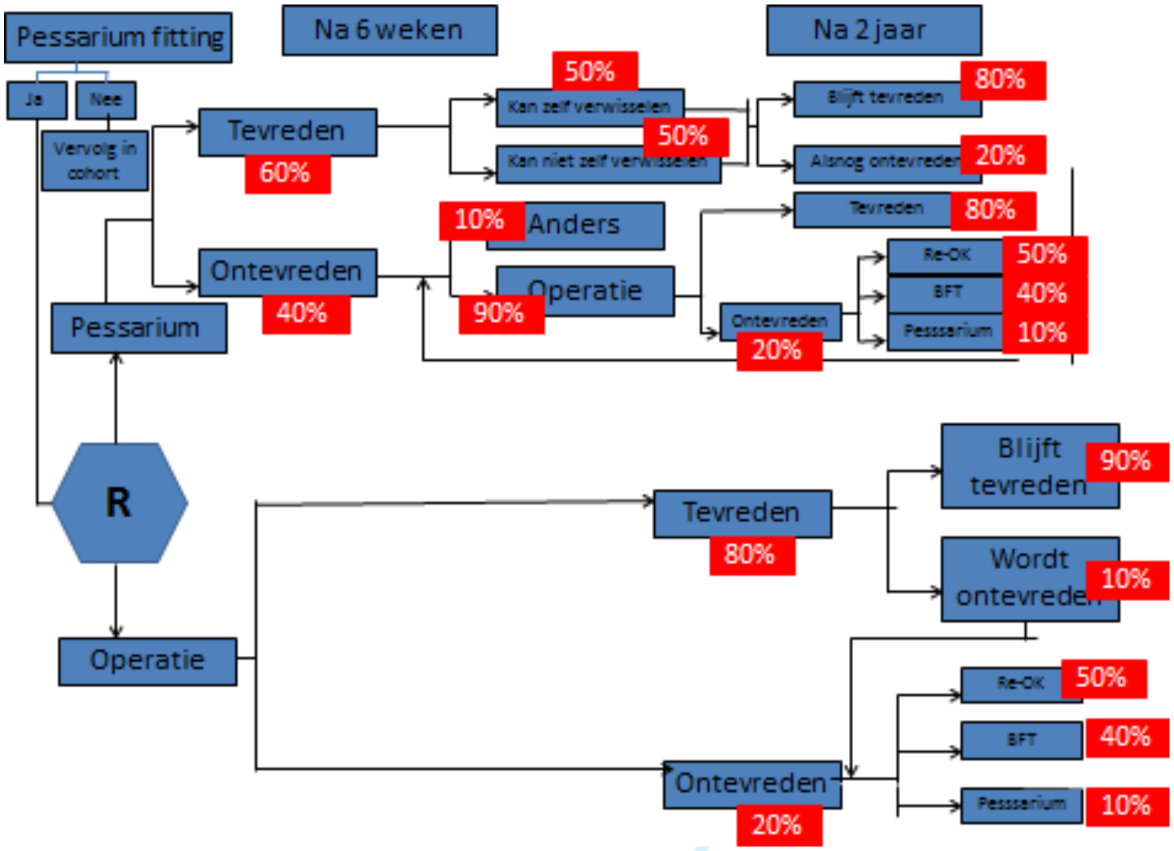

For peer review only - http://bmjopen.bmj.com/site/about/guidelines.xhtml

Appendix 2 Review on (Cost) effectiveness of pessary use as compared to surgery:

Reference	Study type	Characteristics	Intervention (I)	Controls (C)	Outcome measures and follow-uptime	Results
**Mamik, 2012**<br><br>**AJOG 2013:209:488**	**Design**<br>**Case-control**<br>**N = 100**<br>**Country**<br>**US**	**Aim: compare goal achievement and global improvement between pessary and surgery for [illegible] stage ≥2.**<br><br>**Inclusion criteria: >18 year old, read and write in English**<br><br>**Exclusion: not given**	**Vaginal pessary**<br>**N = 50**	**Prolapse surgery**<br>**N = 50**	**Primary outcome:**<br>**Goal attainment**<br><br>**Secondary:**<br>**PGI-I**<br>**PFDI-20**<br>**PISQ-12**<br>**Body Image scale**<br><br>**Follow-up: 3 months**	**Primary outcome:**<br>**Goal attainment sign. higher score after surgery (8.6 vs 6.4)**<br>**Secondary outcomes**<br>**PGI-I sign (p=0.04) better improvement after surgery (2.4vs 1.9 points)**<br>**PFDI-20 sign (p=0.02) higher change (89 vs 43 points)**<br>**PISQ-12 and BIS no sign difference**<br><br>**Additional: 10% crossed over from pessary to surgery within 3 months a[illegible]% referred from surgery after they had been selected as eligible.**<br>**No follow-up in pessary group is 40% (20/50) and surgery 30% [illegible]**
**Abdool, 2011**	**Design**<br>**Cohort study**<br><br>**N total = [illegible]**<br><br>**Country:**<br>**UK**	**Aim of the study**<br>**to evaluate and compare the effectiveness of pessariesand surgery in women with symptomatic pelvic organ prolapse.**<br>**Inclusion criteria**<br>**- Women referred to a specialist urogynaecology unit with symptomatic [illegible] between June 2002 andMay 2007**	**Intervention vaginal pessary**<br>**N = 359**	**Controls surgery**<br>**N = 195**	**Primary outcomes:**<br>**Sheffield POP questionnaire [illegible]**<br><br>**Secundary outcomes:**<br>**None**<br>**Follow up:**<br>**For the surgery and pessary groups 14 months(SD 6.14) and 12 months (SD 3.1), respectively.**	**Primary outcomes:**<br>**No difference in functional outcome after 1 year follow-up between groups**<br>**Additional:**<br>**Only 45% in pessary group en 55% in surgery group responded at 12monthsIn pessary group 24.7% (89/359) crossed to surgery but were not analyzed In pessary group 7.3% stopped because of other reasons.**<br>**Selection and patient preference bias**<br>**The mean age was significantly higher in the pessary group compared to the surgerygroup (68.4 +/− 13.08 vs 60.4 +/−12.25 years, respectively).**

For peer review only - http://bmjopen.bmj.com/site/about/guidelines.xhtml

Study protocol

		**Exclusion criteria**<br>· Subjects fitted with pessaries for urinary incontinence and those who had concomitant urinary incontinence surgery (e.g. TVT)<br>· Subjects who started inthe pessary group but subsequently requested surgery were excluded from analysis in both the surgery and pessary group.				

For peer review only

Study protocol

Lowenstein 2010<br><br>J Sex Med 2010; 7:-1023<br><br>28	Design<br>Cohort study<br>N= 235<br><br>Country<br>US	Aim of the study<br>First to evaluate patient-reported outcome, POP symptoms, sexual functioning and body image following treatment of P[illegible]<br>Second to compare surgery with pessary<br><br>Inclusion: ≥18 year, ≥ satge 2 POP, complete questionnaire at baseline and at ≥6 months follow up<br><br>Exclusion: recurrent UTI, peripheral neuropathy, using pessary at initial presentationor POP surgery < 6 months prior to presentation	Intervention<br>N = 202 surgery	Controls<br>N = 33 pessary	Primary outcomes<br>PFDI-20<br>PISQ-12<br>Modified Body Image scale<br>All at six months follow-up	Results<br>After multivariate analyses, including type of intervention, BMI and differencein Body image were associated with change in total PISQ (sexual functioning) score<br><br>In the pessary group there was no significant improvement in sexual functioningas compared to surgery (-2.5 versus +11.5)<br>Additional:<br>No figures presented for pessary and surgery group, with exemption oftheSexual functioning (PISQ-12) result above.

For peer review only - http://bmjopen.bmj.com/site/about/guidelines.xhtml

Study protocol

**Barber, 2006**	**Design** Case-control study<br>**Ntotal=106Country:** USA	Aim of the studyto evaluate the responsiveness of the PelvicFloor Distress Inventory (PFDI) andelicvPFloor Impact Questionnaire (PFIQ) in women with pelvic organ prolapse undergoingsurgical and nonsurgical management.<br><br>Inclusion criteria<br>Surgery group:<br>Stage III or IV prolapse, wereat least 18 years, and scheduled for vaginal prolapse repair.<br>Pessary group:<br>women with symptomatic pelvic organ prolapse oftsageII or greater. (Pessri trial)<br><br>Exclusion criteriaSurgery group:<br>· mentally or physically incapable of completingthe questionnaires.<br>Pessary group:<br>· were pregnant, were currently using a pessary, orhad vaginal agglutination that precluded pessary insertion.	**Intervention**<br>**Pessary in women with stage II or greater POP**<br>**N = 42**	**Controls**<br>**Surgery in womenwithstage III or greater POP**<br>**N = 64**	**Primary outcomes:**<br>**PFDI and PFIQ**<br>**Secundary outcomes:**<br><br>**Follow up:**<br>**3 months (Pessary group)or 6 months (Surgery group) after initiationoftreatment.**	**Primary outcomes:**<br><br>**After controlling for preoperative prolapse stage and baseline HRQOLscores,subjects in the Surgery group had significantly greater improvement in each of the scales of the PFDI and the prolapse and urinary scales of the PFIQ than did the Pessary group.**<br>**Scores from each of the scales of the PFDI improved by 14 to 15**<br>**points more on average after treatment in the Surgery group than those ofthe Pessary group (P < .01 for each) after adjusting for the above baseline differences.**<br><br>**Similarly, for the prolapse and urinary scales of the PFIQ, scores improved13and 17 points more, respectively, in the Surgery group than the Pessary group after treatment. (P < .05 for each).**<br><br>**Four of 64 (6%) of subjects in the Surgery group had recurrent prolapsedevelopbeyond the hymen by 6 months after surgery. No subjects underwent reoperation for recurrent prolapse during the study period.**<br>**Additional:**<br><br>**Difference in followup**<br>**Selection bias**

For peer review only - http://bmjopen.bmj.com/site/about/guidelines.xhtml

Appendix 3 Review on risk factors for failure of pessaries:

study		n	FU period	success initial	continued pes	stopped pes	cross over surgery	predict fit	predict contin
Wu 1997	retrospective	110	3 yrs	81 (74%)	43 (39%)		at least 5	nee	ja
Mokrzycki 2001	retrospective	42	3 mth		24 (57%)	18 (43%)	nr	nee	ja
Clemons 2004	prospective	100	1 yr	73 (73%)	43 (43%)		16/73 (27%)	nee	ja
Brincat 2004	retrospective	169	2,5 yr	144 (85%)	82 (60%)	54 (40%)	32/144 (22%)	nee	ja
Mutone 2004	retrospective	407	3 wks	168 (41%)		96 (57%)	nr	ja	ja
Powers 2006	retrospective	32	1 yr			20 (62%)	12 (38%)	nee	ja
Broens-Oostveen 2004	retrospective	192	9 yr			107 (52%)	nr	nee	ja
Nguyen 2005	retrospective	130	1 yr	111 (82%)	85 (65%)		20 (15%)	ja	ja
Hanson 2005	retrospective	1216	3 mth	1043 (86%)	744 (71%)		nr	nee	ja
Fernando 2006	prospective	203	4 mth	nr	153 (75%)		28 (14%)	nee	ja
Barber 2006	prospective	42	3 mth	nr		0	nr	nee	nee
Maito 2006	retrospective	120	6 mth	106 (88%)	92 (77%)		9 (8%)	ja	ja
Cundiff 2007	prospective	85	1 yr	65/71 (92%)			5 (6%)	nee	nee
Komesu 2007	prospective	64	6-12 mth		36 (56%)	28 (44%)	2 (3%)	nee	ja
Jones 2008	prospective	90	3 mth		42 (47%)		nr	nee	ja
Kuhn 2009	prospective	71	3 mth		32 (45%)		nr	nee	nee
Sarma 2009	retrospective	120	1 mth		45 (38%)		18 (15%)	nee	nee
Friedman 2010	retrospective	150	1 yr		115 (77%)	35 (23%)	25 (17%)	nee	ja
Patel 2010	prospective	65	3 mth	54 (83%)	54 (83%)		5 (8%)	nee	nee
Abdool 2010	prospective	359	1 yr	296 (83%)	243 (68%)		31 (9%)	nee	nee
Lone 2011	prospective	246	5 yr	187 (76%)	130 (53%)		70 (28%)	nee	nee
Markle 2011	retrospective	158	1 week	92 (59%)			nr	ja	nee
Chan 2012	prospective	197	1 yr	138 (70%)		59 (30%)	nr	nee	ja
Manchana 2012	retrospective	126	1 yr	77 (61%)	64 (51%)		5 (4%)	nee	nee
Mamik 2013	prospective	50	3 mth				5 (10%)	nee	nee
Geoffrion 2013	retrospective	101	4 wks	79 (78%)			nr	nee	ja
Alperin 2013	retrospective	4019	9 years				12% 1 yr, 24% 9 yrs	nee	nee
Succesfull fitting		3589		2714(76%)					
Continuation		3381			2027 (60%)				

For peer review only - http://bmjopen.bmj.com/site/about/guidelines.xhtml

Appendix 4 Review on risk factors for failure of surgery:

Risk factor	Investigated:	Significant:
Preoperative stage	8	5
Age	8	2
Obesity	7	0
Parity	5	0
Constipation	5	0
Pulmonary disease	5	0
Number of sites involved preoperative	4	1
Menopausal status	4	0
Hysterectomy status	4	0
Concomitent surgery	3	1
Family history	3	1
Complicated delivery	3	0
Diabetes	3	0
Smoking	3	0
Previous incontinence and/or prolapse surgery	2	2
Hiatus genitalis	2	1
Weight	2	1
Any incontinence preoperative	2	1
Delivery mode	2	0
Vaginal delivery	2	0
Hormone replacement therapy	2	0
Previous prolapse surgery	2	0
Surgeons experience	2	0
Abcense of posterior repair	1	1
Sexual activity	1	1
Levator defect	1	1
Height	1	0
Birth weight	1	0
Age at last delivery	1	0
Site of most advanced prolapse	1	0
Surgical approach	1	0
Use of Mesh	1	0
Previous incontinence surgery	1	0
Previous pelvic floor surgery or hysterectomy	1	0
Abdominal hernias	1	0
Cardiovascular disease	1	0
Intense physical exercise	1	0
Heavy lifting	1	0
Heavy lifting or constipation	1	0
Levator muscle contraction	1	0
Weight of the uterus	1	0
Postoperative complications	1	0
Incomplete emptying of bladder	1	0
Fecal incontinence	1	0

For peer review only

Appendix 5 diagram/tabel bezoeken, tijdstippen, onderzoeken

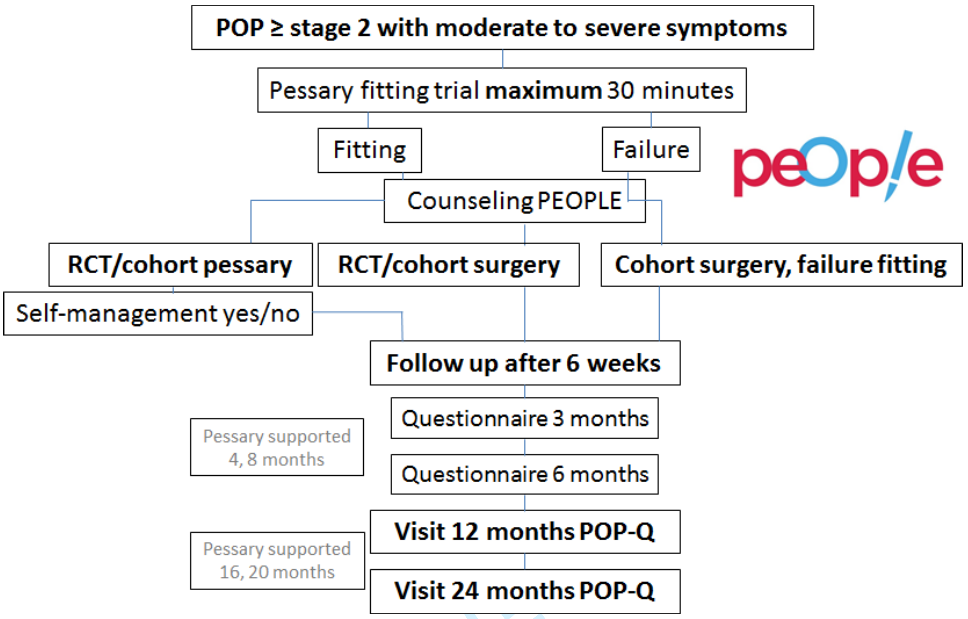

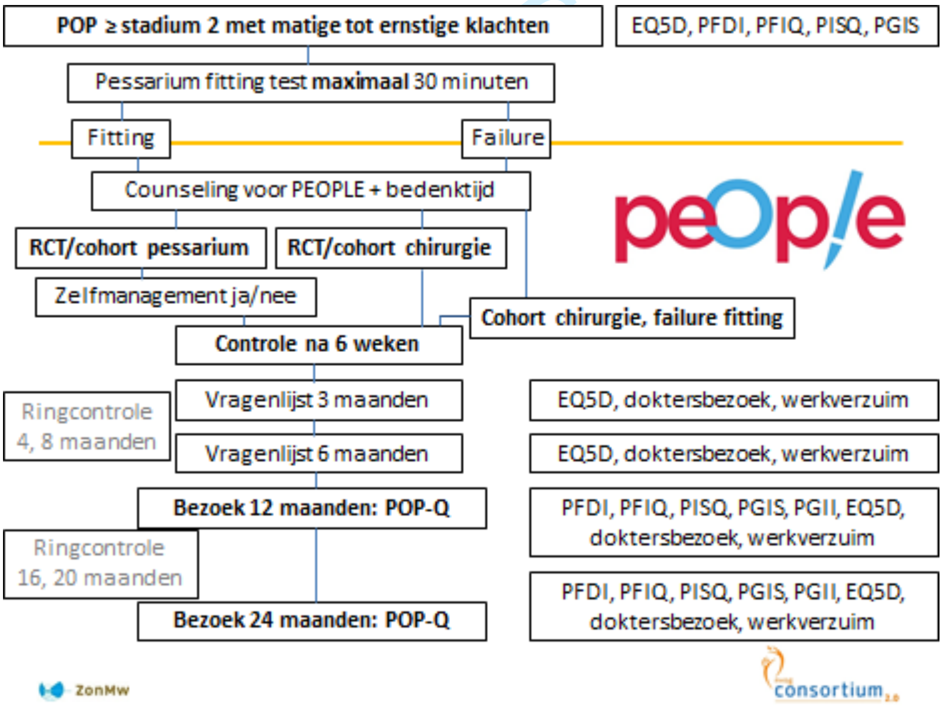

Chirurgie en cohort fitting failure

**Contact**	**Bezoek arts**	**POPQ**	**Eq5d doktersbezoek werkverzuim**	**PFIQ PFDI PISQ PGII PGIS**

1. Eerste bezoek	X	X	Eq5D	X (zonder PGII)
2. 6 weken	X			
3. 3 maanden			X	
4. 6 maanden			X	
5. 12 maanden	X	X	X	X
6. 24 maanden	X	X	X	X

Pessarium met zelfmanagement

Contact	Bezoek arts	POPQ	Eq5d doktersbezoek werkverzuim	PFIQ PFDI PISQ PGII PGIS
1. Eerste bezoek	X	X	Eq5D	X (zonder PGII)
2. 6 weken	X			
3. 3 maanden			X	
4. 6 maanden			X	
5. 12 maanden	X	X	X	X
6. 24 maanden	X	X	X	X

For peer review only

Pessarium zonder zelfmanagement

Contact	Bezoek arts	POPQ	Ringcontrole	Eq5d doktersbezoek werkverzuim	PFIQ PFDI PISQ PGII PGIS
1. Eerste bezoek	X	X		Eq5D	X (zonder PGII)
2. 6 weken	X				
3. 3 maanden				X	
4. 4 maanden	X		X		
5. 6 maanden				X	
6. 8 maanden	X		X		
7. 12 maanden	X	X	X	X	X
8. 16 maanden	X		X		
9. 20 maanden	X		X		
10. 24 maanden	X	X	X	X	X

For peer review only

1.3 Summary of amendment to study protocol

The main change in the final version is the addition of an observational cohort performed alongside the RCT. We added this observational cohort since many women refused to participate in the RCT due to treatment preference. In case a woman was willing to participate in the study but actively opted for one of two treatment options she was followed in the observational cohort. The same study parameters and follow-up were used in both the trial and observational cohort. See section 2, section 3, section 4.4, section 8.2, section 10, section 11.2

1.3.1 Detailed summary of all amendments

1. Addition of multiple centers for participation.

Added centers:

- Atrium MC Heerlen
- Academisch ziekenhuis Maastricht
- Martini ziekenhuis Groningen
- MST Enschede
- ZGT Almelo / Hengelo
- Deventer ziekenhuis
- Jeroen Bosch ziekenhuis
- Amstelland ziekenhuis
- Tergooi ziekenhuis
- Albert Schweitzer ziekenhuis
- Canisius Wilhelmina ziekenhuis
- Maxima Medisch Centrum
- MCH-Bronovo
- OLVG
- HAGA

2. Change in investigators at the following participating centers:
   - St. Antonius hospital. S. The was replaced by E. Vernooij
   - Canisius hospital. C.F. van Heteren was replaced by K.L. Bos
   - Maastricht University center (MUMC): G. Link was replaced by W.A. Spaans

3. Change in Head of Department of Reproductive Medicine and Gynaecology.

4. Change in Objective.

An observational cohort was added since many women refused to participate in the trial due to treatment preference. At first, women were asked to participate in the trial. In case the woman is willing to participate but actively opts for one of both treatments, she will be followed in a cohort 'own choice'.

For peer review only

5. Change in study design.
In the first version it is noted that for women with an unsuccessful pessary fitting only baseline characteristics will be recorded. However, these women will be followed in the cohort fitting failure with the same follow-up as for the trial (24-months). Appendix 5 has been noted in more detail.

6. Addition in sample size calculation for observational cohort.
Since we added an observational cohort with women who made their own choice of treatment, we added this to the section sample size calculation. In the cohort we include all patients who are willing to collaborate on this research but have a preference for one of both therapies. We now assume that 70% of the eligible patients object participation in the RCT, and that 90% of them is nevertheless willing to participate in the cohort.

7. Change in self-management of pessary treatment.
In case self-management was performed, women were advised to change their pessary every 4-months, instead of every 1 month.

8. Observational cohort is added in randomization section.
In case the woman is willing to participate but actively opts for one of both treatments, she will also be registered in ALEA. All groups will have the same data collection and follow up as displayed in appendix 5.

9. Observational cohort added in statistical analysis section.
The cohort with patients treated according their preference will be analyzed separately from the randomized trial. The same analysis will be done.

10. Change in exclusion criteria.
Women with a previous hysterectomy were only excluded in case the indication for the hysterectomy was a prolapse.

11. Observational cohort added in recruitment.
In case the woman is willing to participate but actively opts for one of both treatments, she can attend the cohort.

12. Change in monitoring
At first, the monitoring was coordinated by the Staff Member Clinical Research, quality coordinator of division women and baby. Later on, the monitoring was conducted by the Dutch consortium and was executed by a qualified intern monitor.

13. POP-Q only performed at 12- and 24-months follow-up, not at 6 weeks visit.
Demonstrated in the tables listed in appendix 5.

For peer review only - http://bmjopen.bmj.com/site/about/guidelines.xhtml

1.3.2 Table with amendments and corresponding section

**Amendment**	**Corresponding section in the final version 1.22**
1. Addition of multiple centers for participation	First table with project information
2. Change in investigators	First table with project information
3. Change in Head of Department	Protocol signature sheet
4. Change in objective	Section 2
5. Change in study design	Section 3
6. Addition in sample size calculation for observational cohort	Section 4.4
7. Change in self-management of pessary treatment	Section 5.1
8. Observational cohort is added in randomization section	Section 8.2
9. Observational cohort added in statistical analysis section	Section 10
10. Change in exclusion criteria	Section 10.2
11. Observational cohort added in recruitment	Section 11.2
12. Change in monitoring	Section 12.2
13. POP-Q only performed at 12- and 24-months	Appendix 5

For peer review only

For peer review only

For peer review only - http://bmjopen.bmj.com/site/about/guidelines.xhtml

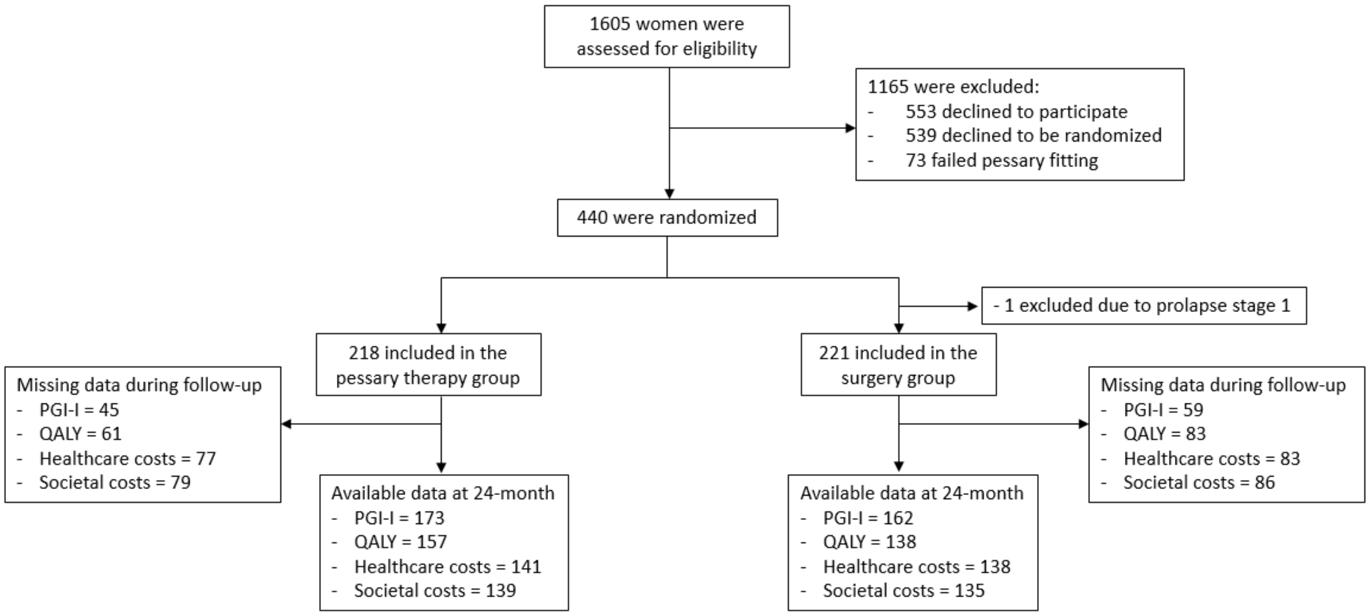

SUPPLEMENTARY Figure 1. FLOW DIAGRAM. Inclusion and available data at 24-month follow-up.

For peer review only

SUPPLEMENTARY TABLE 1. RESOURCES AND UNIT COSTS

Resources	Unit costs	Year	Reference
Pessary device			
Milex®	€64	2022	Market price: bol.com
Arabin®	€73	2022	Market price: bol.com
Other brand (average)	€68	2022	Market price: bol.com
Pessary placement	€109	2022	Dutch costing manual[1]
Surgery			
Sacrospinous hysteropexy (care product 149999033)	€5835	2022	DBC[2]
Sacrospinous fixation (care product 149999047)	€4640	2022	DBC[2]
Manchester–Fothergill procedure (care product 149999047)	€4640	2022	DBC[2]
Abdominal sacrocolpopexy (care product 149999033)	€5835	2022	DBC[2]
Sacrocervicopexy care product 149999033)	€5835	2022	DBC[2]
Vaginal hysterectomy (care product 149999047)	€4640	2022	DBC[2]
Average surgical procedures costs (used as WTP threshold)	€5237	2022	DBC[2]
**Other resources**			
General practitioner consultation	€39	2022	Dutch costing manual[1]
Other healthcare professional consultation at primary care	€39	2022	Dutch costing manual[1]
Medical specialist consultation at secondary care	€109	2022	Dutch costing manual[1]
Hospital readmission (1 day)	€568	2022	Dutch costing manual[1]
Paid working hour for women	€38	2022	Dutch costing manual[1]

DBC: Diagnosis Treatment Combination, in Dutch *Diagnose Behandeling CombinatieI.*

References:

1 Kanters TA, Bouwmans CAM, van der Linden N, *et al.* Update of the Dutch manual for costing studies in health care. *PLoS One* 2017;**12**. doi:10.1371/journal.pone.0187477

2 Diagnose Behandeling Combinatie (DBC) open data - NZa. https://www.opendisdata.nl/ (accessed 3 Sep 2022).

For peer review only

CHEERS 2022 Checklist

Topic	No.	Item	Location where item is reported
**Title**			
	1	Identify the study as an economic evaluation and specify the interventions being compared.	Page 1, 1st paragraph
**Abstract**			
	2	Provide a structured summary that highlights context, key methods, results, and alternative analyses.	Page 2
**Introduction**			
**Background and objectives**	3	Give the context for the study, the study question, and its practical relevance for decision making in policy or practice.	Page 4
**Methods**			
**Health economic analysis plan**	4	Indicate whether a health economic analysis plan was developed and where available.	Page 5, Study design, 1st paragraph
**Study population**	5	Describe characteristics of the study population (such as age range, demographics, socioeconomic, or clinical characteristics).	Page 5, Study population, 2nd paragraph
**Setting and location**	6	Provide relevant contextual information that may influence findings.	Page 6, Setting and location, 1st paragraph
**Comparators**	7	Describe the interventions or strategies being compared and why chosen.	Page 6, Comparators, Pessary therapy, 2nd paragraph and Surgery, 3rd paragraph
**Perspective**	8	State the perspective(s) adopted by the study and why chosen.	Page 7, Study perspective, time horizon, and discount rate, 1st paragraph
**Time horizon**	9	State the time horizon for the study and why appropriate.	Page 7, Study perspective, time horizon, and discount rate, 1st paragraph

For peer review only - http://bmjopen.bmj.com/site/about/guidelines.xhtml

Topic	No.	Item	Location where item is reported
**Discount rate**	10	Report the discount rate(s) and reason chosen.	Page 7, Study perspective, time horizon, and discount rate, 1st paragraph
**Selection of outcomes**	11	Describe what outcomes were used as the measure(s) of benefit(s) and harm(s).	Page 7, Outcomes, Health outcomes, 2nd and 3rd paragraph
**Measurement of outcomes**	12	Describe how outcomes used to capture benefit(s) and harm(s) were measured.	Page 7, Outcomes, Health outcomes, 2nd and 3rd paragraph
**Valuation of outcomes**	13	Describe the population and methods used to measure and value outcomes.	Page 7, Outcomes, Health outcomes, 2nd and 3rd paragraph
**Measurement and valuation of resources and costs**	14	Describe how costs were valued.	Page 8-9, Cost outcomes
**Currency, price date, and conversion**	15	Report the dates of the estimated resource quantities and unit costs, plus the currency and year of conversion.	Page 9, 1st paragraph, last sentence
**Rationale and description of model**	16	If modelling is used, describe in detail and why used. Report if the model is publicly available and where it can be accessed.	Page 9, Cost-effectiveness analysis, 3rd paragraph
**Analytics and assumptions**	17	Describe any methods for analysing or statistically transforming data, any extrapolation methods, and approaches for validating any model used.	Page 9-10, Cost-effectiveness analysis
**Characterising heterogeneity**	18	Describe any methods used for estimating how the results of the study vary for subgroups.	Page 10, Sensitivity analysis
**Characterising distributional effects**	19	Describe how impacts are distributed across different individuals or adjustments made to reflect priority populations.	Page 9, 3rd paragraph and Page 10, 1st paragraph
**Characterising uncertainty**	20	Describe methods to characterise any sources of uncertainty in the analysis.	Page 10, Sensitivity analysis
**Approach to engagement with patients and others affected by the study**	21	Describe any approaches to engage patients or service recipients, the general public, communities, or stakeholders (such as clinicians or payers) in the design of the study.	Page 11, Patient and Public Involvement

Topic	No.	Item	Location where item is reported
**Results**			
**Study parameters**	22	Report all analytic inputs (such as values, ranges, references) including uncertainty or distributional assumptions.	Page 12, Participants, 1st paragraph
**Summary of main results**	23	Report the mean values for the main categories of costs and outcomes of interest and summarise them in the most appropriate overall measure.	Page 12-13, Effectiveness, Costs
**Effect of uncertainty**	24	Describe how uncertainty about analytic judgments, inputs, or projections affect findings. Report the effect of choice of discount rate and time horizon, if applicable.	Page 13, Cost-effectiveness analysis. Page 14, Sensitivity analysis
**Effect of engagement with patients and others affected by the study**	25	Report on any difference patient/service recipient, general public, community, or stakeholder involvement made to the approach or findings of the study	Not Applicable
**Discussion**			
**Study findings, limitations, generalisability, and current knowledge**	26	Report key findings, limitations, ethical or equity considerations not captured, and how these could affect patients, policy, or practice.	Page 15-17
**Other relevant information**			
**Source of funding**	27	Describe how the study was funded and any role of the funder in the identification, design, conduct, and reporting of the analysis	Page 3
**Conflicts of interest**	28	Report authors conflicts of interest according to journal or International Committee of Medical Journal Editors requirements.	Page 3

From: Husereau D, Drummond M, Augustovski F, et al. Consolidated Health Economic Evaluation Reporting Standards 2022 (CHEERS 2022) Explanation and Elaboration: A Report of the ISPOR CHEERS II Good Practices Task Force. Value Health 2022;25. doi:10.1016/j.jval.2021.10.008
